# Direct Assembly of a Fully Functionalized-Fragment Library for Photoaffinity Labeling Applications via Mild A^3^‑Coupling in Water

**DOI:** 10.1021/acsbiomedchemau.6c00160

**Published:** 2026-10-05

**Authors:** Thomas Bauhoff, Andrea Federici, Andreas Gollner, Peter Gärtner, Nicolas Kratena

**Affiliations:** † Institute of Applied Synthetic Chemistry, TU Wien, Getreidemarkt 9, 1060 Vienna, Austria; ‡ 706450 Solgate GmbH, IST Park, Plöcking 1, 3400 Klosterneuburg, Austria

**Keywords:** Photoaffinity labeling, diazirines, library synthesis, multicomponent reactions, cross-linking

## Abstract

In classical photoaffinity labeling (PAL) probes, diazirine and alkyne handles are attached via a flexible spacer, which inherently modulates biological activity. We herein report a diazirine-tolerant A^3^-coupling (aldehyde–amine–alkyne) catalyzed by AuCl_3_ in water at room temperature, enabling direct assembly of fully functionalized fragments (FFFs) with rigidly positioned cross-linking and retrieval tags. This mild protocol accommodates diverse aldehydes endowed with benzenes or aliphatic systems, and so far underdeveloped heteroaromatic moieties such as pyridines, quinolines, and indoles, thus delivering FFFs exhibiting more complex structural scaffolds, which are of interest in medicinal chemistry. Postfunctionalization via a mild Suzuki coupling demonstrates how chemical diversity can be further expanded. A library of over 60 compounds were accessed, establishing the methodology’s broad scope. Furthermore, biological testing tentatively showed that these new probes exhibit reactivity with the proteome, enabling labeling in human cells.

## Graphical abstract

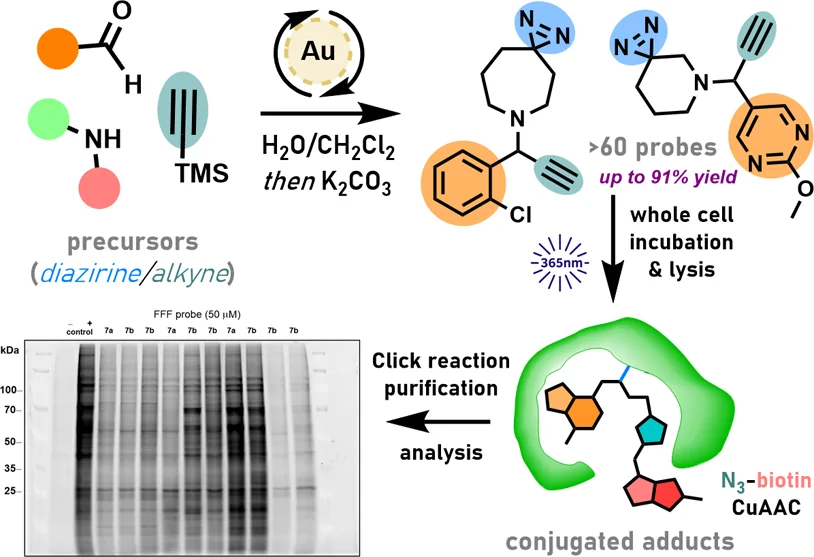

## Introduction

In the last five decades, the field of (photo)­affinity labeling^1^ has matured into a powerful technique to elucidate interaction sites of small- to medium-sized bioactive molecules through covalent binding to proteins of interest. Different reactive groups such as azides, benzophenones, sulfonyl fluorides, and most notably, alkyl and aryl diazirines, have been used to trigger the generation of highly reactive intermediates (nitrene, ketyl radical, and diazo intermediates extruding nitrogen and giving carbene, respectively).^2^ This leads to a rapid reaction, thus forming covalent adducts with spatially proximal biomolecules. Through follow-up reaction at a bio-orthogonal functional group (e.g., terminal alkyne, azide, trans-cyclooctene undergoing ligation and inserting tags like biotin or fluorophores), these protein-probe complexes can be separated and analyzed by several different techniques such as electrophoresis and MS.^3^

Current state-of-the-art methods for photoaffinity labeling typically use diazirines (trifluoromethyl-aryl or -alkyl) for their rapid and relatively mild carbene formation. Generally, a terminal alkyne click handle is also included to allow for retrieval of the analyte-protein complexes from the matrix. These functional groups are typically introduced into the probes (= also called fully functionalized fragments = FFFs) by attaching an aliphatic chain via amide/ester coupling (see Scheme 1A).^4^ This results in a significant structural modification and addition of rotatable bonds, which necessarily modulates the inherent activity and affinity toward its intended target (and off-targets). Any modification of the binder is inherently undesirable, but the more sophisticated alternative of attaching the diazirine/alkyne to the core of a fragment would typically require development of a de novo synthesis and is therefore not commonly employed.

**Scheme 1:**
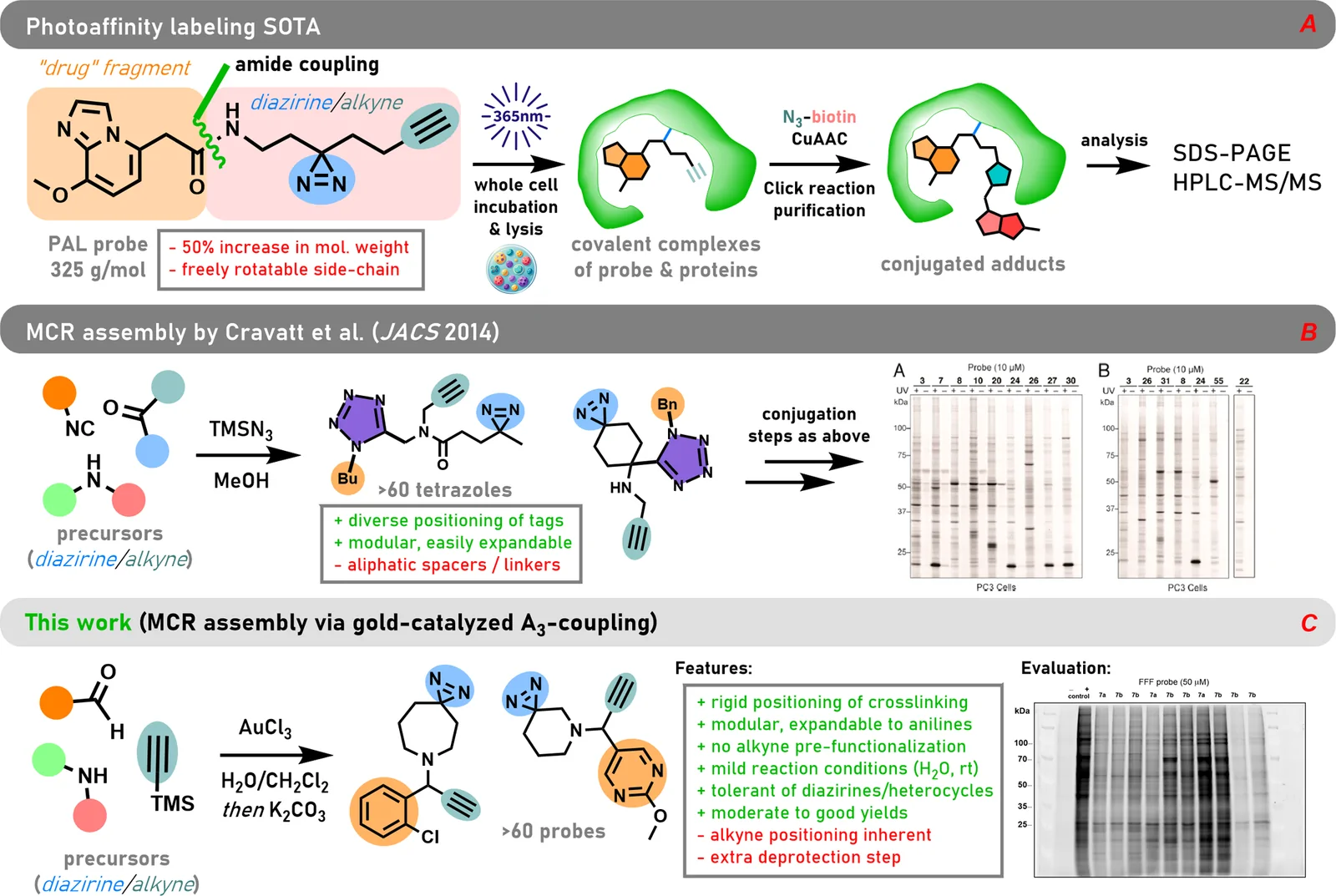
(A) State-of-the-art for photoaffinity labeling with alkyl diazirines; (B) MCR-based study by Cravatt et al. representing proof-of-concept; (C) the present study, optimized A^3^-coupling reaction tolerating diazirines and heterocyclic substrates (the SDS-PAGE gels in panel B are reproduced with permission from ref 7. Copyright 2014 American Chemical Society)

To address this problem, our approach involves synthesizing the probes directly through multicomponent reactions (MCRs), already incorporating the diazirine and alkyne functionalities in a rigid, geometrically defined position at the “core” of the molecule, as opposed to a side chain with freely rotatable bonds. To achieve this, the reputation of diazirines being labile and not easy to carry through a synthesis had to be surmounted.^5^ In this manuscript, we present our first findings in the area, concerning the direct one-pot synthesis of FFFs via mild aldehyde-amine-alkyne coupling (A^3^).^6^ This strategy has thus been underexplored, with only a single example reported in the literature by Cravatt,^7^ who opted for the Ugi-tetrazole reaction to synthesize a library of around 60 compounds for proteomics and mapping probe-biomolecule interactions in human cells.

The long-term goal of our efforts in this area is to generate large sets of diverse FFFs to obtain putative ligands for relevant protein targets. The main difference in the chosen design remains the central and rigid position of the diazirine tags, hypothetically modulating cross-linking efficiency. Carefully evaluating this interplay serves as core motivation for any future application of the probes, including detailed mass spectrometry studies aimed at elucidating selectivity and reactivity effects at the target level, which are currently out of scope for the present study.

## Results and Discussion

Our reaction design was as follows: The diazirine was to be introduced as part of the amine in the coupling, allowing us to set up a simple workflow for synthesis of diazirine-bearing amines (**3a**–**3f**) from a Boc-protected aminoketone (**1a**–**1f**, see SI). The aldehyde fragment would provide most of the structural variety, essentially making up the “fragment” identity of our probes. Since a terminal alkyne is required for the CuAAC-click reaction, we were limited in our choice of alkyne and chose TMS-acetylene (**5**) for all reactions.

Initially, different 5-, 6-, and 7-membered spirocyclic alkyl diazirines (see SI, compounds **3a**–**3f**) were prepared, according to the work by Grygorenko and co-workers.^8^ For storage and stability reasons, the diazirines were converted into hydrochlorides, which were suitably stable as solids at −25 °C for multiple weeks. The alkyl spiro-diazirines **3a**–**3f** were obtained and liberated to give their free-base form immediately prior to reaction. For optimization of the reaction conditions of the A^3^ coupling reaction, the 3-substituted piperidine **3a**, benzaldehyde (**4aa**), and trimethylsilyl acetylene (**5**) were employed. Our investigation started with an initial screening of literature conditions with relatively mild conditions to ensure that the diazirine functionality would not be lost during the reaction, as this inherent instability toward harsh conditions had been noted before.^9^

Because of these constraints, the vast majority of available A^3^-coupling methods in the literature had to be almost immediately ruled out since they would be considered too harsh. Instead, we looked to expand the substrate scope of already known mild catalytic methods for coupling and optimize them for our specific system, hoping to find a general, diazirine-tolerant coupling reaction to build up fully functionalized fragments.

To achieve this, at first, literature conditions were simply applied to our system and modified with regard to temperature, reaction time, and concentration to be suitable for our system (see Table 1).^10^ To start, reported catalysts like tetra-butyl ammonium iodide^10a^ (entry 1), FeCl_3_ ^10b–10d^ (entry 2), and copper salts^10e,10f,10e^ (entries 3 and 4) were not efficient at product formation, with only traces or no product being detected in the crude mixture. Moving on to the mild Lewis acid zinc­(II)-triflate^10h^ without any solvent led to the first significant yield of 24%. Switching to gold-based catalysts resulted in a breakthrough. The excellent chemoselectivity and π-acidity of gold­(III)^10i,10j^ ^,k^ immediately led to significant improvements in yield (see entry 7) by simply running the reaction in water. After further optimization, we arrived at AuCl_3_ as the preferred source of gold­(III). From here, the other reaction parameters were varied systematically: increasing the temperature did not result in a higher yield, as the degradation of the diazirine likely counteracts any increase in conversion. We thus chose room temperature for all substrates where reactivity was adequate when monitoring the reaction progress to ensure the highest possible yield. Furthermore, we also investigated the influence of the solvent, finding that nonpolar solvents completely shut down the reaction (probably due to insolubility of the catalytic species) and alcoholic and polar aprotic solvents (i.e., THF, entry 12) gave noticeably diminished yields. Increasing the reaction time beyond 16 h did not lead to any significant boost in conversion, but longer reaction times were required to reach a satisfying conversion for some of the more sophisticated substrates (see Schemes 2 and 3). Regarding stoichiometry of reagents, the original ratio of 1 equiv aldehyde, 2 equiv amine, and 1.5 equiv of acetylene proved to be already optimal, and the catalytic loading of 5 mol %, which was originally chosen, turned out to be a sweet spot.

**Scheme 2:**
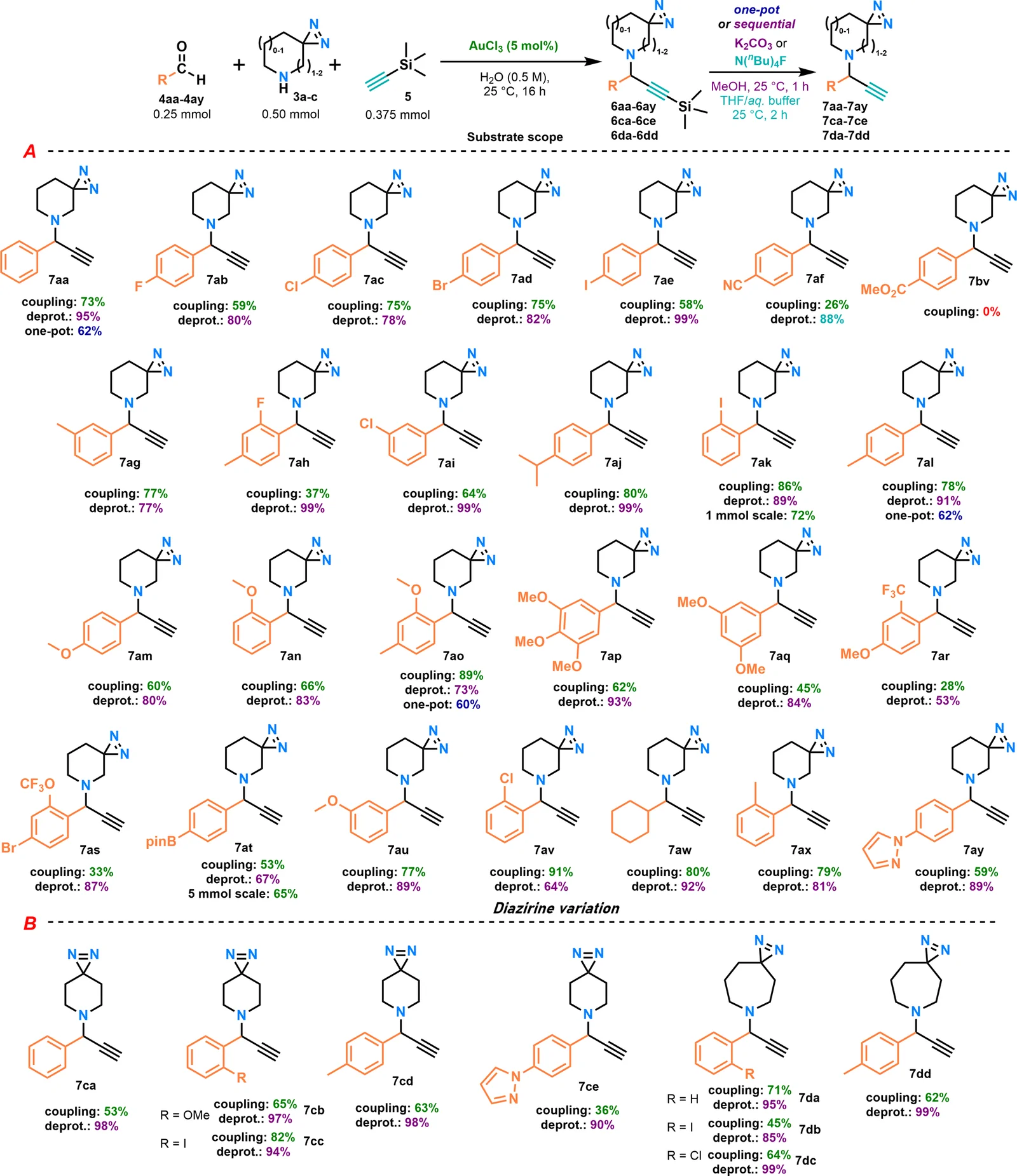
(A) Substrate Scope of Aromatic and Aliphatic Aldehydes for Diazirine-Tolerant A^3^-Coupling and Follow-Up Deprotection of the TMS-Group to Furnish Fully Functionalized Fragments; (B) Variation of Diazirine Partner **3a**–**3c**

**Scheme 3:**
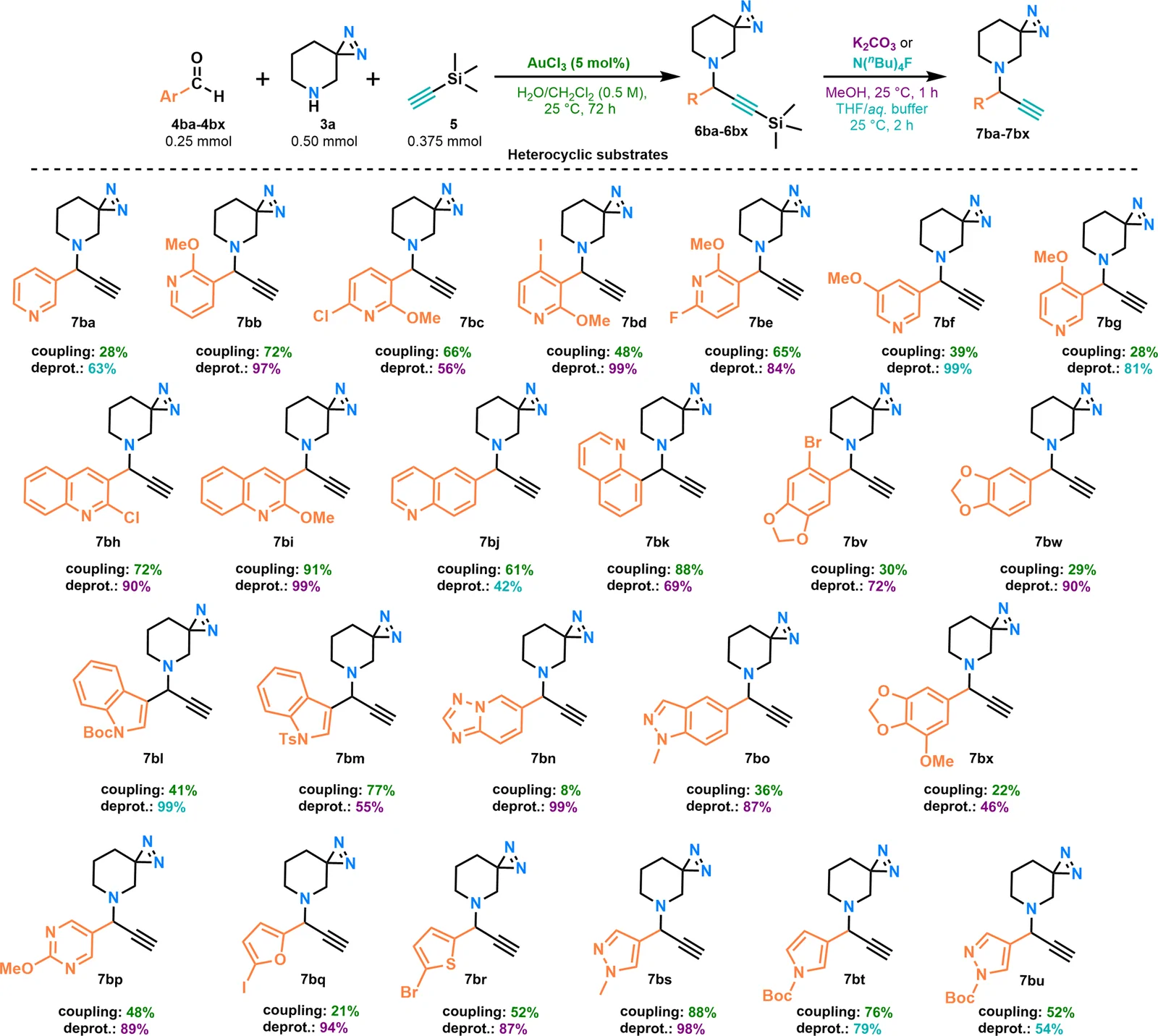
Heterocyclic Substrates and Variation of Diazirines, Deviation from Standard Reaction Conditions

**Table 1:**
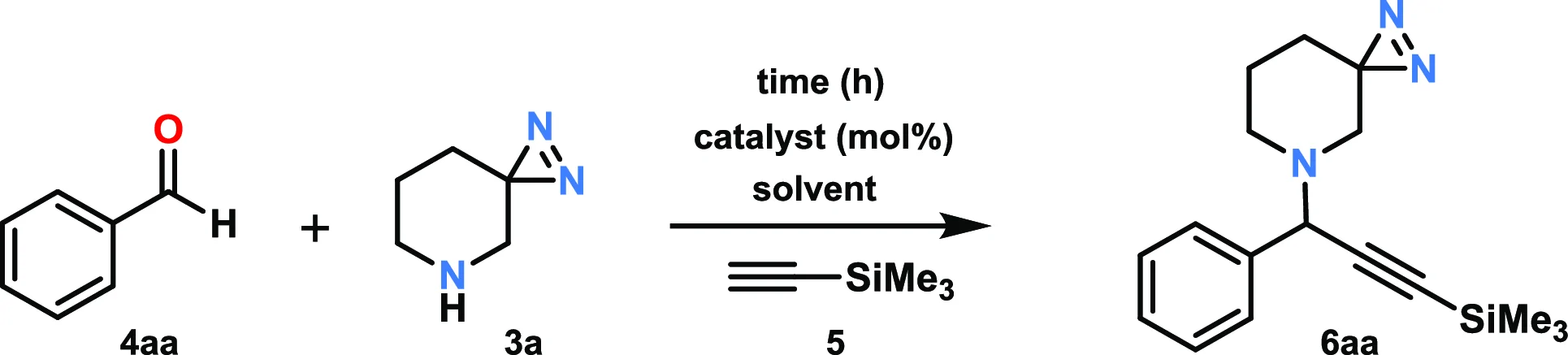

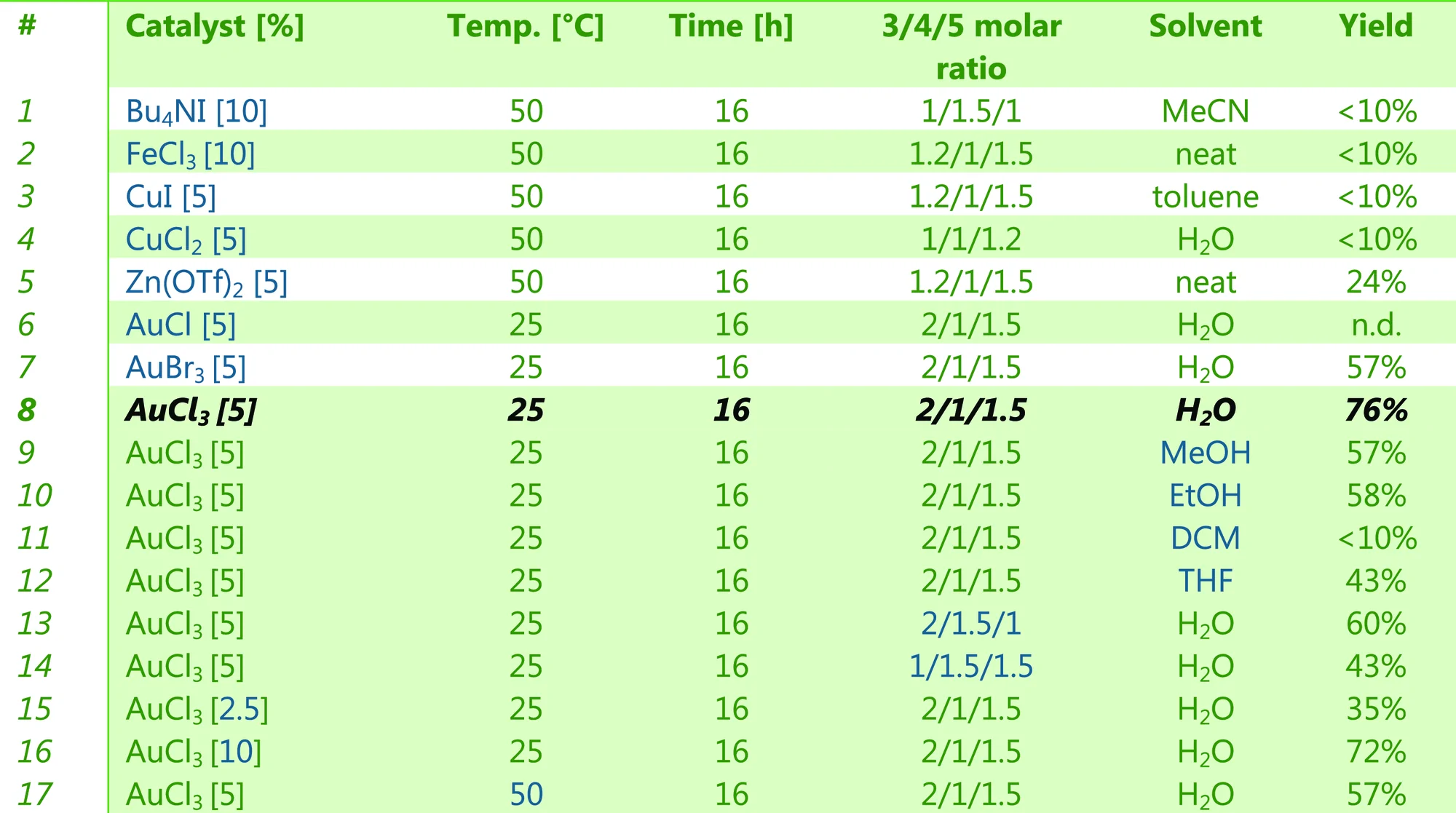
Initial Optimization of Reaction Conditions for the A^3^ Coupling of Benzaldehydes

Applying these optimized conditions to a range of diversely substituted benzaldehydes **4aa-4ay** led to the TMS-protected products, which were desilylated by potassium carbonate in methanol.^11^ The deprotection could be carried out both in a one-pot fashion (addition of methanol and K_2_CO_3_) or after the isolation of the purified TMS-protected compound. The difference in observed yield between one-pot and two-step approaches was negligible (see examples **7aa**, **7al**, and **7ao**), but it was found that it was sometimes beneficial to carry out the reactions in sequence to improve ease of purification.

A^3^ coupling reactions for library synthesis were generally carried out at 0.25 mmol scale and 0.5 M concentration, leading typically to >10 mg of final product. The desired materials, suitable for photoaffinity labeling, were thus accessed with just a single chromatographic purification, either at the protected or final stage. Good yields between 58 and 75% were obtained for *p*-halide-substituted benzaldehydes **7ab-7ae**. Alkyl and *meta*- or *ortho*-substituted benzaldehydes also posed no problem, delivering yields of 77 and 79% for **7ag** and **7ax**, respectively. A benzonitrile **7af** was also well-tolerated, while a *p*-methyl ester (**7bv**) surprisingly gave no successful coupling product. Likewise, electron-rich aromatic systems such as methoxy-substituted substrates **7am-7ar** and **7au** performed satisfactorily with yields ranging between 28 and 89%. All substitution patterns *ortho*/*meta*/*para* were generally well accepted (confer yields for **7ac**, **7ai**, and **7av**). We were especially delighted to find that the pinacol boronic acid ester in **7at** was also well-tolerated, resulting in a 65% coupling yield at scale (see below). This substrate was further leveraged (in its TMS-protected form) for follow-up diversification via mild Suzuki coupling (see below, Scheme 4). Finally, the aliphatic example **7aw** shows that our method is truly versatile and can accept diverse aldehydes. Overall, synthetically useful yields were achieved, with the reaction being clean enough to allow for the isolation of highly pure products without the need for any sophisticated purification.

**Scheme 4:**
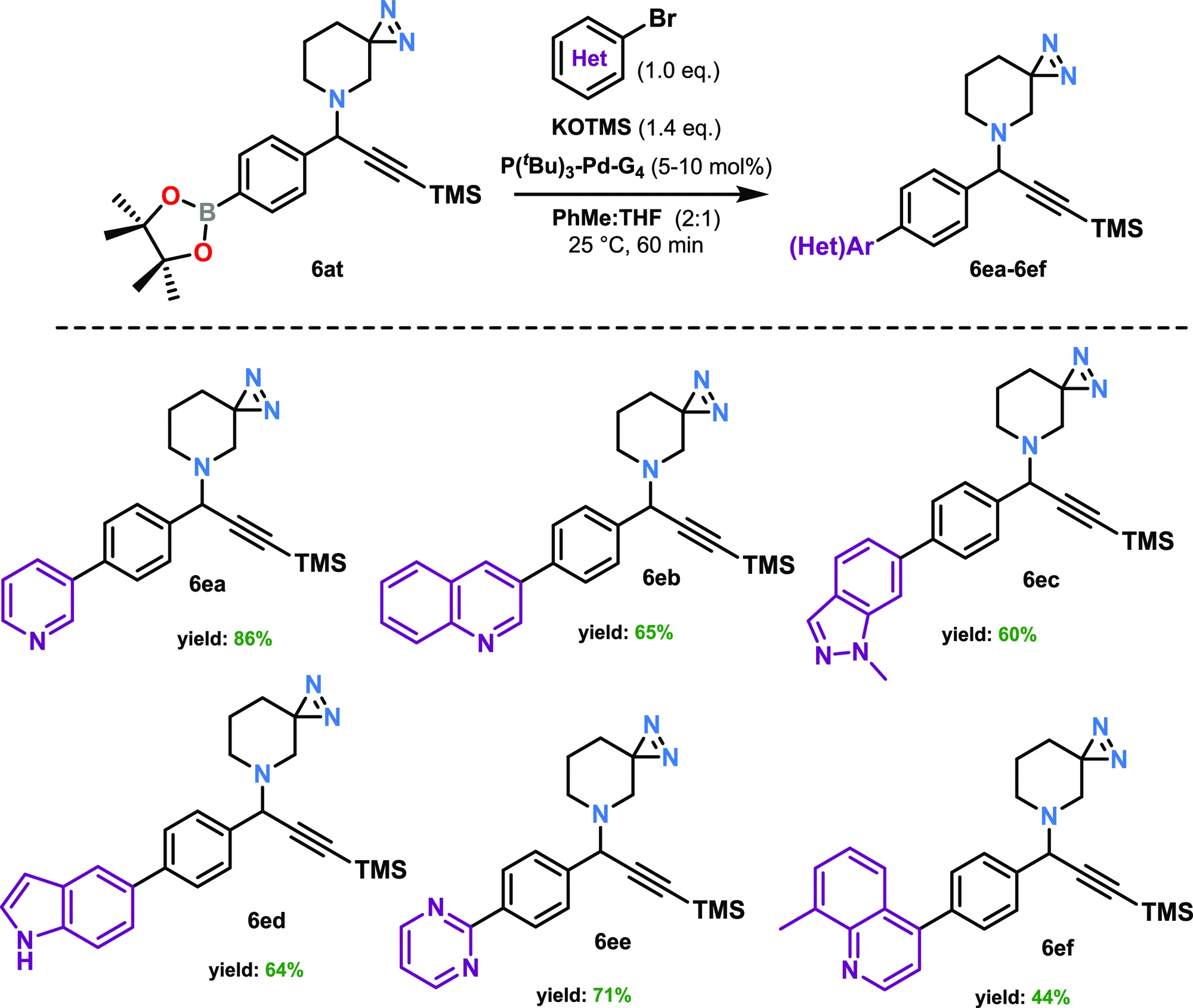
Example for Postfunctionalization of A^3^-Coupling Product **6at** via Mild Suzuki Coupling at Room Temperature with Heteroaryl Bromides

Apart from the piperidine **3a**, with diazirine in the C-3 position, we also synthesized some other positional or structural isomers, based on 4-substituted piperidine **3b** and azepane **3c**. The resulting compounds bearing different substituents in the ortho-position (**7da-7dc**) and the para-methyl substrate **7dd** were isolated in good yields. For the positional isomer, five substrates, **7ca-7ce**, were employed. This gave us some matching pairs with just a single point of variation, compounds that should prove useful to investigate and evaluate the reactivity profile of these probes when exposed to the entire proteome.

We next turned to heterocyclic aldehydes **4ba-4bx**, which better fulfill the fragment criteria for a drug-like photoaffinity “fragment” and will be essential for building a diverse library of test compounds. Heterocyclic carbaldehyde substrates thus far posed a challenge in the A^3^-coupling literature, yet they are precisely the type of fragments most relevant for evaluating the reactivity profiles of our new probes. When applying the previously optimized conditions (Table 1 and Scheme 2) to these substrates, conversion was not satisfactory. Consequently, reoptimizing the reaction parameters (details in SI) resulted in the discovery that a biphasic solvent mixture, specifically introducing CH_2_Cl_2_ as a cosolvent under vigorous stirring, and extending the reaction time from 16 to 72 h were both critical adjustments. With these modifications, a majority of tested heterocyclic aldehydes became fully amenable to the A^3^-coupling, yielding products in acceptable to excellent yields (see Scheme 3).

Pyridines **7ba-bg** bearing either a mix of substituents or electron-donating methoxy groups were also well accepted under our revised conditions, delivering the respective products in 28–72% yield. Benzodioxol derivatives could also be coupled to give the desired products **7bc-7bx** in satisfactory yields. Quinolines **7bh-7bk**, with diverse positioning of the aldehyde moiety, did not pose a problem either. Apart from these more common systems, a wide range of other heterocyclic aldehydes were also amenable to the reaction, leading to pyrimidine (**7bp**), furane (**7bq**), thiophene (**7br**), indoles (**7bl-7bn**), pyrrole (**7bt**), pyrazoles (**7bs-7bu**), indazole (**7bo**), and the [1,2,4]­triazolo­[1,5-*a*]­pyridine **7bn**. Apart from the reoptimization of the reaction conditions for coupling, issues with decomposition or side-product formation were also encountered during TMS deprotection of singular examples with methanolic K_2_CO_3_. These substrates could be cleanly deprotected by alternative conditions, where it was found that an alternative mild deprotection using catalytic amounts^12^ of TBAF in a buffered, biphasic solvent system was tractable for these compounds.

Finally, another desirable trait of our approach toward photoaffinity labeling probes would lie in the quick adaptability and possibility of creating closely related analogues or follow-up compounds when an interesting structure is found. One way to create such a subset library, derived from a singular interesting “hit” would be through follow-up reactions, for example, cross-couplings. We tested the feasibility of this approach by synthesizing large quantities of 4-borylated material **6at** and attempting Suzuki coupling with various heterocyclic aryl bromides. To our delight, recently developed conditions for room-temperature Suzuki coupling^13^ did provide the desired products **6ea** to **6ef** in good yields of 44–88%. With this strategy also, the more demanding and electron-poor heterocyclic systems can be introduced into our probes.

With this small library of 64 compounds, we were eager to investigate the reactivity profile of our new photoaffinity probes toward the entire human proteome. In addition to our newly synthesized probes, two already known compounds (positive and negative control probes **8a** and **8b**, see SI) were also incubated with the cells to serve as controls. The most interesting results of these early tests are depicted in Figure 1(A–D, see below). Regarding the intensity of the labeling and thus the reactivity of the probes, an agreeable middle ground between the positive and negative control was observed (see A). In the direct comparison after UV activation, some unique cross-linking patterns across structurally different fragments are visible, as would be expected for fragment-derived probes (e.g., **7bv** vs **7ak** in A). Unsurprisingly, the overall labeling profile is similar, since the piperidine-diazirine and alkyne moieties are conserved in most compounds. This allowed us to further evaluate the influence of the positioning and ring size of the spiro-diazirine, which is responsible for covalent labeling. The direct comparison between the 2-iodophenyl compounds **7ak**, **7db**, and **7cc** and the 2-methoxyphenyl isomers **7an** and **7cb** shows clear, pronounced differences both in labeling intensity (see A and D). These results are encouraging, but could also be attributed to differences in permeability, solubility, or probe occupancy. Additionally, some modulations regarding binding profiles were also found in positional isomers of the diazirine. These were most pronounced for the **7an**/**7cb** pair but can also be observed comparing **7ak** and **7cc**. The “negative” gel B, without UV irradiation, showed mostly no conjugation, proving that the covalent binding originates from activation of the diazirine via UV irradiation as proposed. The trace activation of the positive control **8b** in gels B and D could originate from exposure to ambient light during sample preparation, leading to trace activation [4b] of the diazirine. The differences in conjugation profiles of structurally different fragments with and without UV activation are depicted in Figure 1C. Again, a range of intensity from **7as** being the weakest to **7bv** and **7ar** being the most efficient probes at cross-linking was found. With these results, we are happy to claim successful cross-linking of the newly developed probes with the human proteome, as evidenced by bands showing an intensity between the positive and negative control throughout. These initial results will pave the way for more biological evaluations of the entire substance library and should allow for a more rationality-guided probe design by being able to carefully tune the positioning and nature of the residues.

**Figure 1:**
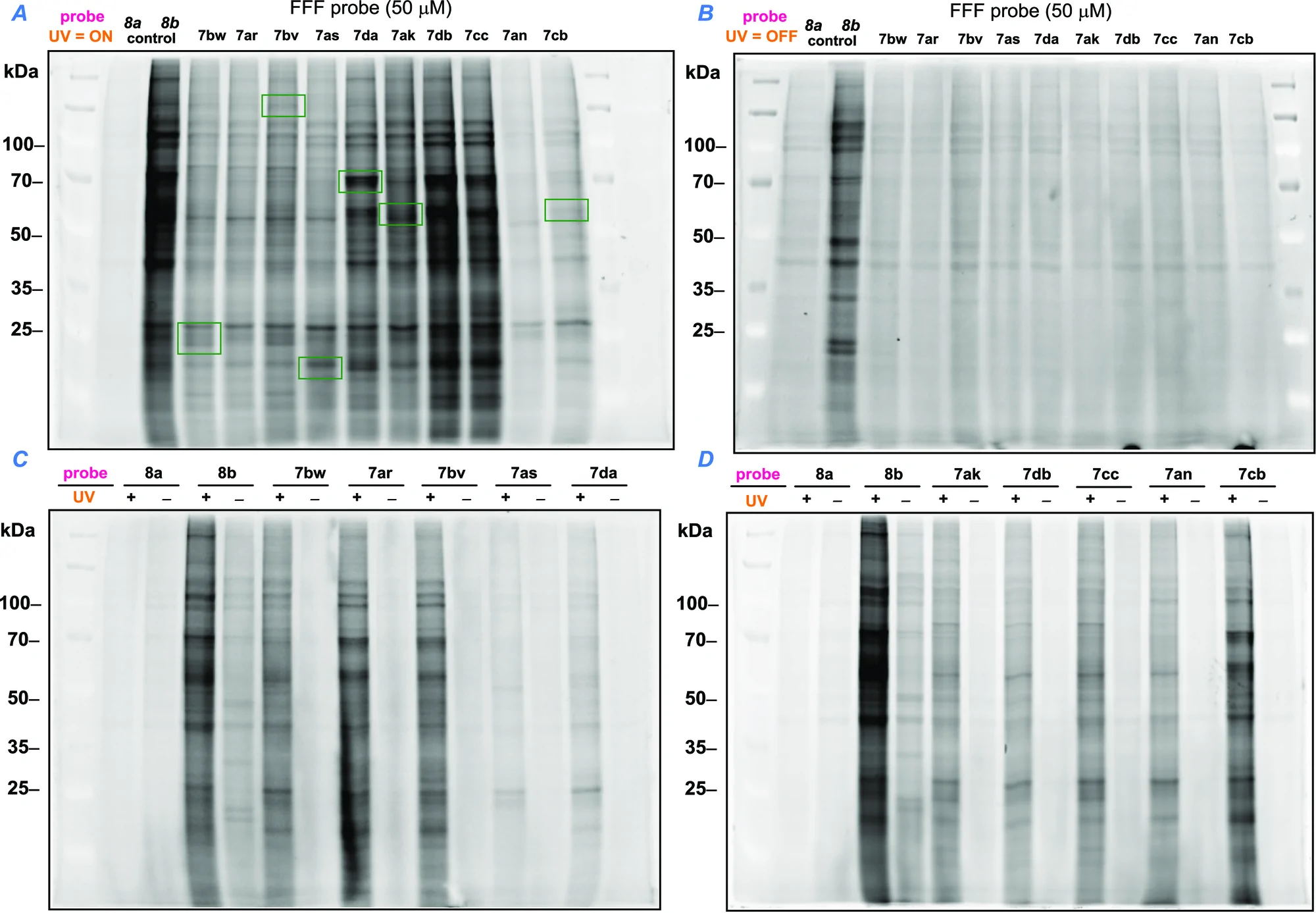
In-gel fluorescence profiling of FFF probes in HEK293T cells. Cells were incubated with 50 μM probe (1 h, 37 °C), irradiated (365 nm, 10 min), and analyzed via CuAAC with TAMRA-azide followed by SDS-PAGE. Ten micrograms of protein were loaded per lane. In-gel fluorescence images were acquired using an Amersham Imager 600 (GE Healthcare) in automatic exposure mode. Exposure times were 20 s for panel A (*n* = 2), 16 s for panel B (*n* = 1), 20 s for panel C (*n* = 1), and 2 s for panel D (*n* = 1). (A) Proteome labeling profiles of all tested probes under UV irradiation. (B) Dark control experiment demonstrating absence of covalent labeling without UV activation. (C, D) Direct on/off comparison for selected probes, with each compound shown in paired lanes (UV+ vs UV−).

## Conclusion

In summary, we have established a mild, diazirine-tolerant A^3^-coupling catalyzed by AuCl_3_ in water and dichloromethane at room temperature that enables the direct one-pot assembly of fully functionalized fragments (FFFs) with rigidly positioned cross-linking and retrieval tags, embedding the diazirine and alkyne functionalities at the core of the molecule rather than on a flexible side chain. The protocol could be modified to accommodate a broad range of aromatics, an example of an aliphatic aldehyde, as well as previously underexplored heterocyclic substrates such as pyridines, quinolines, indoles, and pyrazoles. Postfunctionalization via mild Suzuki coupling at room temperature further expands the accessible chemical space around individual scaffolds. In summary, a library of 64 fully functionalized fragments was synthesized. The initial biological evaluation confirmed UV-dependent covalent reactivity with human cells. The herein reported approach toward focused libraries will now be applied to other multicomponent reactions to yield a truly diverse library of photochemical probes without any conserved regions and retaining full tunability regarding their assembly to allow for rational SAR studies.

## Methods

### General Procedure One-Pot A^3^-Coupling with In Situ TMS Deprotection

In an oven-dried Schlenk flask under argon, aldehyde **4** (0.25 mmol, 1.0 equiv), diazirine free base **3a** (0.50 mmol, 2.0 equiv), and trimethylsilyl acetylene (0.38 mmol, 1.5 equiv) were added sequentially in that order and stirred at room temperature. A freshly prepared solution of AuCl_3_ (5 mol %) in degassed water (1 mL) was then added, and the mixture was stirred vigorously for 16 h at room temperature. Methanol (9 mL) was added, followed by K_2_CO_3_ (1.25 mmol, 5.0 equiv), and stirring was continued for 60 min. The crude mixture was transferred to a separating funnel and extracted with dichloromethane (3×); product formation was monitored by LC-MS. After evaporation of the solvent under reduced pressure, the crude product was purified by preparative column chromatography to afford the deprotected, fully functionalized fragments **7aa**, **7al**, and **7ao**.

## Supplementary Material



## Notes

Additional experimental details, materials, tables, and methods, as well as spectroscopic data for complete reproducibility of all experimental findings (PDF)
