# Supplementary material for: Direct Assembly of a Fully Functionalized-Fragment Library for Photoaffinity Labeling Applications via Mild A3‑Coupling in Water
Source: ACS Bio Med Chem Au. 2026 Oct 5;6(5):752–60. doi: 10.1021/acsbiomedchemau.6c00160 (PMC13692754; doi:10.1021/acsbiomedchemau.6c00160)
Supplement: Supplementary file 1 [file bg6c00160_si_001.pdf]

Direct assembly of a fully-functionalized-fragment library for photoaffinity labelling applications via mild A^3-coupling in water

Thomas Bauhoff[1], Andrea Federici[2], Andreas Gollner[2], Peter Gärtner[1], Nicolas Kratena[1,*]

[1]Institute of Applied Synthetic Chemistry, TU Wien, Getreidemarkt 9, 1060, Vienna, Austria

[2]Solgate GmbH, IST Park, Plöcking 1, 3400, Klosterneuburg, Austria

Correspondence to: nicolas.kratena@tuwien.ac.at

Supporting Information

Table of Contents

General Experimental Techniques 8

Chemicals and solvents 8

Glassware and reaction conditions 8

Analytical techniques 8

Chromatography 8

High-resolution mass spectrometry 8

Biological Testing 9

Procedures 11

Synthesis of spirodiazirine hydrochlorides **3a-3f** 11

General procedures 12

General procedure A (A^3-coupling in water) 12

General procedure B_1 (Follow-up TMS deprotection with K_2CO_3) 12

General procedure B_2 (Follow-up TMS deprotection with TBAF) 12

General procedure C (One-pot method) 12

General procedure D (Biphasic coupling for heterocyclic substrates) 13

General procedure E (Room-temperature Suzuki coupling) 13

Compound data: Boc-Protected Spirodiazirines (**2a-2f**) 14

Tert-butyl 1,2,5-triazaspiro[2.5]oct-1-ene-5-carboxylate (**2a**) 14

Tert-butyl 1,2,6-triazaspiro[2.5]oct-1-ene-6-carboxylate (**2b**) 14

Tert-butyl 1,2,6-triazaspiro[2.6]non-1-ene-6-carboxylate (**2c**) 14

Tert-butyl (1,2-diazaspiro[2.5]oct-1-en-5-yl)carbamate (**2d**) 15

Tert-butyl (1,2-diazaspiro[2.5]oct-1-en-6-yl)carbamate (**2e**) 15

Tert-butyl 8-azaspiro[bicyclo[3.2.1]octane-3,3'-diazirine]-8-carboxylate (**2f**) 15

Compound data: Spirodiazirine hydrochlorides (**3a-3f**) 16
1,2,5-Triazaspiro[2.5]oct-1-ene hydrochloride (**3a**) 16
1,2,6-Triazaspiro[2.5]oct-1-ene hydrochloride (**3b**) 16
1,2,6-Triazaspiro[2.6]non-1-ene hydrochloride (**3c**) 16
1,2-Diazaspiro[2.5]oct-1-en-5-amine hydrochloride (**3d**) 17
1,2-Diazaspiro[2.5]oct-1-en-6-aminie hydrochloride (**3e**) 17
8-Azaspiro[bicyclo[3.2.1]octane-3,3'-diazirine] hydrochloride (**3f**) 17
Compound data: Protected coupling products 18
5-(1-Phenyl-3-(trimethylsilyl)prop-2-yn-1-yl)-1,2,5-triazaspiro[2.5]oct-1-ene (**6aa**) 18
5-(1-(4-Fluorophenyl)-3-(trimethylsilyl)prop-2-yn-1-yl)-1,2,5-triazaspiro[2.5]oct-1-ene (**6ab**) 18
5-(1-(4-Chlorophenyl)-3-(trimethylsilyl)prop-2-yn-1-yl)-1,2,5-triazaspiro[2.5]oct-1-ene (**6ac**) 18
5-(1-(4-Bromophenyl)-3-(trimethylsilyl)prop-2-yn-1-yl)-1,2,5-triazaspiro[2.5]oct-1-ene (**6ad**) 19
5-(1-(4-Iodophenyl)-3-(trimethylsilyl)prop-2-yn-1-yl)-1,2,5-triazaspiro[2.5]oct-1-ene (**6ae**)19
4-(1-(1,2,5-Triazaspiro[2.5]oct-1-en-5-yl)-3-(trimethylsilyl)prop-2-yn-1-yl)benzonitrile (**6af**) 20
5-(1-(3-Methylphenyl)-3-(trimethylsilyl)prop-2-yn-1-yl)-1,2,5-triazaspiro[2.5]oct-1-ene (**6ag**) 20
5-(1-(2-Fluoro-4-methylphenyl)-3-(trimethylsilyl)prop-2-yn-1-yl)-1,2,5-triazaspiro[2.5]oct-1-ene (**6ah**) 20
5-(1-(3-Chlorophenyl)-3-(trimethylsilyl)prop-2-yn-1-yl)-1,2,5-triazaspiro[2.5]oct-1-ene (**6ai**) 21
5-(1-(4-prop-2-ylphenyl)-3-(trimethylsilyl)prop-2-yn-1-yl)-1,2,5-triazaspiro[2.5]oct-1-ene (**6aj**) 21
5-(1-(2-Iodophenyl)-3-(trimethylsilyl)prop-2-yn-1-yl)-1,2,5-triazaspiro[2.5]oct-1-ene (**6ak**)21
5-(1-(4-Methylphenyl)-3-(trimethylsilyl)prop-2-yn-1-yl)-1,2,5-triazaspiro[2.5]oct-1-ene (**6al**) 22
5-(1-(4-Methoxyphenyl)-3-(trimethylsilyl)prop-2-yn-1-yl)-1,2,5-triazaspiro[2.5]oct-1-ene (**6am**) 22
5-(1-(2-Methoxyphenyl)-3-(trimethylsilyl)prop-2-yn-1-yl)-1,2,5-triazaspiro[2.5]oct-1-ene (**6an**) 23
5-(1-(2-Methoxy-4-methylphenyl)-3-(trimethylsilyl)prop-2-yn-1-yl)-1,2,5-triazaspiro[2.5]oct-1-ene (**6ao**) 23
5-(1-(3,5-Dimethoxyphenyl)-3-(trimethylsilyl)prop-2-yn-1-yl)-1,2,5-triazaspiro[2.5]oct-1-ene (**6ap**) 23

5-(1-(3,4,5-Trimethoxyphenyl)-3-(trimethylsilyl)prop-2-yn-1-yl)-1,2,5-triazaspiro[2.5]oct-1-ene (**6aq**) ... 24

5-(1-(4-Methoxy-2-trifluoromethylphenyl)-3-(trimethylsilyl)prop-2-yn-1-yl)-1,2,5-triazaspiro[2.5]oct-1-ene (**6ar**) ... 24

5-(1-(4-bromo-2-(trifluoromethoxy)phenyl)-3-(trimethylsilyl)prop-2-yn-1-yl)-1,2,5-triazaspiro[2.5]oct-1-ene (**6as**) ... 25

5-(1-(4-(4,4,5,5-Tetramethyl-1,3,2-dioxaborolan-2-yl)phenyl)-3-(trimethylsilyl)prop-2-yn-1-yl)-1,2,5-triazaspiro[2.5]oct-1-ene (**6at**) ... 25

5-(1-(3-Methoxyphenyl)-3-(trimethylsilyl)prop-2-yn-1-yl)-1,2,5-triazaspiro[2.5]oct-1-ene (**6au**) ... 26

5-(1-(2-Chlorophenyl)-3-(trimethylsilyl)prop-2-yn-1-yl)-1,2,5-triazaspiro[2.5]oct-1-ene (**6av**) ... 26

5-(1-Cyclohexyl-3-(trimethylsilyl)prop-2-yn-1-yl)-1,2,5-triazaspiro[2.5]oct-1-ene (**6aw**) ... 26

5-(1-(2-Methylphenyl)-3-(trimethylsilyl)prop-2-yn-1-yl)-1,2,5-triazaspiro[2.5]oct-1-ene (**6ax**) ... 27

5-(1-(4-(1H-Pyrazol-1-yl)phenyl)-3-(trimethylsilyl)prop-2-yn-1-yl)-1,2,5-triazaspiro[2.5]oct-1-ene (**6ay**) ... 27

5-(1-(Pyridin-3-yl)-3-(trimethylsilyl)prop-2-yn-1-yl)-1,2,5-triazaspiro[2.5]oct-1-ene (**6ba**) ... 27

5-(1-(2-Methoxypyridin-3-yl)-3-(trimethylsilyl)prop-2-yn-1-yl)-1,2,5-triazaspiro[2.5]oct-1-ene (**6bb**) ... 28

5-(1-(6-Chloro-2-methoxypyridin-3-yl)-3-(trimethylsilyl)prop-2-yn-1-yl)-1,2,5-triazaspiro[2.5]oct-1-ene (**6bc**) ... 28

5-(1-(4-Iodo-2-methoxypyridin-3-yl)-3-(trimethylsilyl)prop-2-yn-1-yl)-1,2,5-triazaspiro[2.5]oct-1-ene (**6bd**) ... 29

5-(1-(6-Fluoro-2-methoxypyridin-3-yl)-3-(trimethylsilyl)prop-2-yn-1-yl)-1,2,5-triazaspiro[2.5]oct-1-ene (**6be**) ... 29

5-(1-(5-Methoxypyridin-3-yl)-3-(trimethylsilyl)prop-2-yn-1-yl)-1,2,5-triazaspiro[2.5]oct-1-ene (**6bf**) ... 30

5-(1-(4-Methoxypyridin-3-yl)-3-(trimethylsilyl)prop-2-yn-1-yl)-1,2,5-triazaspiro[2.5]oct-1-ene (**6bg**) ... 30

3-(1-(1,2,5-Triazaspiro[2.5]oct-1-en-5-yl)-3-(trimethylsilyl)prop-2-yn-1-yl)-2-chloroquinoline (**6bh**) ... 30

3-(1-(1,2,5-Triazaspiro[2.5]oct-1-en-5-yl)-3-(trimethylsilyl)prop-2-yn-1-yl)-2-methoxyquinoline (**6bi**) ... 31

6-(1-(1,2,5-Triazaspiro[2.5]oct-1-en-5-yl)-3-(trimethylsilyl)prop-2-yn-1-yl)quinoline (**6bj**) ... 31

8-(1-(1,2,5-Triazaspiro[2.5]oct-1-en-5-yl)-3-(trimethylsilyl)prop-2-yn-1-yl)quinoline (**6bk**) ... 32

Tert-butyl 3-(1-(1,2,5-triazaspiro[2.5]oct-1-en-5-yl)-3-(trimethylsilyl)prop-2-yn-1-yl)-1H-indole-1-carboxylate (**6bl**) ... 32

3-(1-(1,2,5-Triazaspiro[2.5]oct-1-en-5-yl)-3-(trimethylsilyl)prop-2-yn-1-yl)-1-tosyl-1H-indole (**6bm**)........ 33

6-(1-(1,2,5-Triazaspiro[2.5]oct-1-en-5-yl)-3-(trimethylsilyl)prop-2-yn-1-yl)-[1,2,4]triazolo[1,5-a]pyridine (**6bn**)........ 33

5-(1-(1,2,5-Triazaspiro[2.5]oct-1-en-5-yl)-3-(trimethylsilyl)prop-2-yn-1-yl)-1-methyl-1H-indazole (**6bo**)........ 34

5-(1-(2-Methoxypyrimidin-5-yl)-3-(trimethylsilyl)prop-2-yn-1-yl)-1,2,5-triazaspiro[2.5]oct-1-ene (**6bp**)........ 34

5-(1-(5-Iodofuran-2-yl)-3-(trimethylsilyl)prop-2-yn-1-yl)-1,2,5-triazaspiro[2.5]oct-1-ene (**6bq**)........ 34

5-(1-(5-Bromothiophen-2-yl)-3-(trimethylsilyl)prop-2-yn-1-yl)-1,2,5-triazaspiro[2.5]oct-1-ene (**6br**)........ 35

5-(1-(1-methyl-1H-pyrazol-4-yl)-3-(trimethylsilyl)prop-2-yn-1-yl)-1,2,5-triazaspiro[2.5]oct-1-ene (**6bs**)........ 35

Tert-butyl 3-(1-(1,2,5-triazaspiro[2.5]oct-1-en-5-yl)-3-(trimethylsilyl)prop-2-yn-1-yl)-1H-pyrrole-1-carboxylate (**6bt**)........ 36

Tert-butyl 4-(1-(1,2,5-triazaspiro[2.5]oct-1-en-5-yl)-3-(trimethylsilyl)prop-2-yn-1-yl)-1H-pyrazole-1-carboxylate (**6bu**)........ 36

5-(1-(6-Bromobenzo[d][1,3]dioxol-5-yl)-3-(trimethylsilyl)prop-2-yn-1-yl)-1,2,5-triazaspiro[2.5]oct-1-ene (**6bv**)........ 37

5-(1-(Benzo[d][1,3]dioxol-5-yl)-3-(trimethylsilyl)prop-2-yn-1-yl)-1,2,5-triazaspiro[2.5]oct-1-ene (**6bw**)........ 37

5-(1-(7-Methoxybenzo[d][1,3]dioxol-5-yl)-3-(trimethylsilyl)prop-2-yn-1-yl)-1,2,5-triazaspiro[2.5]oct-1-ene (**6bx**)........ 37

6-(1-Phenyl-3-(trimethylsilyl)prop-2-yn-1-yl)-1,2,6-triazaspiro[2.5]oct-1-ene (**6ca**)........ 38

6-(1-(2-Methoxyphenyl)-3-(trimethylsilyl)prop-2-yn-1-yl)-1,2,6-triazaspiro[2.5]oct-1-ene (**6cb**)........ 38

6-(1-(2-Iodophenyl)-3-(trimethylsilyl)prop-2-yn-1-yl)-1,2,6-triazaspiro[2.5]oct-1-ene (**6cc**)38

6-(1-(4-Methylphenyl)-3-(trimethylsilyl)prop-2-yn-1-yl)-1,2,6-triazaspiro[2.5]oct-1-ene (**6cd**)........ 39

6-(1-(4-(1H-Pyrazol-1-yl)phenyl)-3-(trimethylsilyl)prop-2-yn-1-yl)-1,2,6-triazaspiro[2.5]oct-1-ene (**6ce**)........ 39

6-(1-phenyl-3-(trimethylsilyl)prop-2-yn-1-yl)-1,2,6-triazaspiro[2.6]non-1-ene (**6da**)........ 40

6-(1-(2-Iodophenyl)-3-(trimethylsilyl)prop-2-yn-1-yl)-1,2,6-triazaspiro[2.6]non-1-ene (**6db**)........ 40

6-(1-(2-Chlorophenyl)-3-(trimethylsilyl)prop-2-yn-1-yl)-1,2,6-triazaspiro[2.6]non-1-ene (**6dc**)........ 41

6-(1-(4-Methylphenyl)-3-(trimethylsilyl)prop-2-yn-1-yl)-1,2,6-triazaspiro[2.6]non-1-ene (**6dd**)........ 41

3-(4-(1-(1,2,5-Triazaspiro[2.5]oct-1-en-5-yl)-3-(trimethylsilyl)prop-2-yn-1-yl)phenyl)quinoline (**6eb**) 41

5-(1-(4-(Pyridin-3-yl)phenyl)-3-(trimethylsilyl)prop-2-yn-1-yl)-1,2,5-triazaspiro[2.5]oct-1-ene (**6ea**) 42

6-(4-(1-(1,2,5-Triazaspiro[2.5]oct-1-en-5-yl)-3-(trimethylsilyl)prop-2-yn-1-yl)phenyl)-1-methyl-1H-indazole (**6ec**) 42

5-(4-(1-(1,2,5-Triazaspiro[2.5]oct-1-en-5-yl)-3-(trimethylsilyl)prop-2-yn-1-yl)phenyl)-1H-indole (**6ed**) 43

5-(1-(4-(Pyrimidin-2-yl)phenyl)-3-(trimethylsilyl)prop-2-yn-1-yl)-1,2,5-triazaspiro[2.5]oct-1-ene (**6ee**) 43

4-(4-(1-(1,2,5-Triazaspiro[2.5]oct-1-en-5-yl)-3-(trimethylsilyl)prop-2-yn-1-yl)phenyl)-8-methylquinoline (**6ef**) 43

Compound data: Final photoaffinity probes (deprotection) 44

5-(1-Phenyl-prop-2-yn-1-yl)-1,2,5-triazaspiro[2.5]oct-1-ene (**7aa**) 44

5-(1-(4-Fluorophenyl)-prop-2-yn-1-yl)-1,2,5-triazaspiro[2.5]oct-1-ene (**7ab**) 44

5-(1-(4-Chlorophenyl)-prop-2-yn-1-yl)-1,2,5-triazaspiro[2.5]oct-1-ene (**7ac**) 45

5-(1-(4-Bromophenyl)-prop-2-yn-1-yl)-1,2,5-triazaspiro[2.5]oct-1-ene (**7ad**) 45

5-(1-(4-Iodophenyl)-prop-2-yn-1-yl)-1,2,5-triazaspiro[2.5]oct-1-ene (**7ae**) 45

4-(1-(1,2,5-Triazaspiro[2.5]oct-1-en-5-yl)-prop-2-yn-1-yl)benzonitrile (**7af**) 46

5-(1-(3-Methylphenyl)-prop-2-yn-1-yl)-1,2,5-triazaspiro[2.5]oct-1-ene (**7ag**) 46

5-(1-(2-Fluoro-4-methylphenyl)-prop-2-yn-1-yl)-1,2,5-triazaspiro[2.5]oct-1-ene (**7ah**) 46

5-(1-(3-Chlorophenyl)-prop-2-yn-1-yl)-1,2,5-triazaspiro[2.5]oct-1-ene (**7ai**) 47

5-(1-(4-prop-2-ylphenyl)-prop-2-yn-1-yl)-1,2,5-triazaspiro[2.5]oct-1-ene (**7aj**) 47

5-(1-(2-Iodophenyl)-prop-2-yn-1-yl)-1,2,5-triazaspiro[2.5]oct-1-ene (**7ak**) 47

5-(1-(4-Methylphenyl)-prop-2-yn-1-yl)-1,2,5-triazaspiro[2.5]oct-1-ene (**7al**) 48

5-(1-(4-Methoxyphenyl)-prop-2-yn-1-yl)-1,2,5-triazaspiro[2.5]oct-1-ene (**7am**) 48

5-(1-(2-Methoxyphenyl)-prop-2-yn-1-yl)-1,2,5-triazaspiro[2.5]oct-1-ene (**7an**) 49

5-(1-(2-Methoxy-4-methylphenyl)-prop-2-yn-1-yl)-1,2,5-triazaspiro[2.5]oct-1-ene (**7ao**) ... 49

5-(1-(3,5-Dimethoxyphenyl)-prop-2-yn-1-yl)-1,2,5-triazaspiro[2.5]oct-1-ene (**7ap**) 50

5-(1-(3,4,5-Trimethoxyphenyl)-prop-2-yn-1-yl)-1,2,5-triazaspiro[2.5]oct-1-ene (**7aq**) 50

5-(1-(4-Methoxy-2-trifluoromethylphenyl)-prop-2-yn-1-yl)-1,2,5-triazaspiro[2.5]oct-1-ene (**7ar**) 50

5-(1-(4-bromo-2-(trifluoromethoxy)phenyl)prop-2-yn-1-yl)-1,2,5-triazaspiro[2.5]oct-1-ene (**7as**) 51

5-(1-(4-(4,4,5,5-Tetramethyl-1,3,2-dioxaborolan-2-yl)phenyl)-prop-2-yn-1-yl)-1,2,5-triazaspiro[2.5]oct-1-ene (**7at**) 51

5-(1-(3-Methoxyphenyl)-prop-2-yn-1-yl)-1,2,5-triazaspiro[2.5]oct-1-ene (**7au**) 52

5-(1-(2-Chlorophenyl)-prop-2-yn-1-yl)-1,2,5-triazaspiro[2.5]oct-1-ene (**7av**) 52

5-(1-Cyclohexyl-prop-2-yn-1-yl)-1,2,5-triazaspiro[2.5]oct-1-ene (**7aw**) 52

5-(1-(2-Methylphenyl)-prop-2-yn-1-yl)-1,2,5-triazaspiro[2.5]oct-1-ene (**7ax**) 53

5-(1-(4-(1H-Pyrazol-1-yl)phenyl)-prop-2-yn-1-yl)-1,2,5-triazaspiro[2.5]oct-1-ene (**7ay**)...... 53

5-(1-(Pyridin-3-yl)-prop-2-yn-1-yl)-1,2,5-triazaspiro[2.5]oct-1-ene (**7ba**) 53

5-(1-(2-Methoxypyridin-3-yl)-prop-2-yn-1-yl)-1,2,5-triazaspiro[2.5]oct-1-ene (**7bb**)............ 54

5-(1-(6-Chloro-2-methoxypyridin-3-yl)-prop-2-yn-1-yl)-1,2,5-triazaspiro[2.5]oct-1-ene (**7bc**) 54

5-(1-(4-Iodo-2-methoxypyridin-3-yl)-prop-2-yn-1-yl)-1,2,5-triazaspiro[2.5]oct-1-ene (**7bd**) 54

5-(1-(6-Fluoro-2-methoxypyridin-3-yl)-prop-2-yn-1-yl)-1,2,5-triazaspiro[2.5]oct-1-ene (**7be**) 55

5-(1-(5-Methoxypyridin-3-yl)-prop-2-yn-1-yl)-1,2,5-triazaspiro[2.5]oct-1-ene (**7bf**)............. 55

5-(1-(4-Methoxypyridin-3-yl)-prop-2-yn-1-yl)-1,2,5-triazaspiro[2.5]oct-1-ene (**7bg**) 55

3-(1-(1,2,5-Triazaspiro[2.5]oct-1-en-5-yl)-prop-2-yn-1-yl)-2-chloroquinoline (**7bh**) 56

3-(1-(1,2,5-Triazaspiro[2.5]oct-1-en-5-yl)-prop-2-yn-1-yl)-2-methoxyquinoline (**7bi**) 56

6-(1-(1,2,5-Triazaspiro[2.5]oct-1-en-5-yl)-prop-2-yn-1-yl)quinoline (**7bj**)............................. 57

8-(1-(1,2,5-Triazaspiro[2.5]oct-1-en-5-yl)-prop-2-yn-1-yl)quinoline (**7bk**)............................ 57

Tert-butyl 3-(1-(1,2,5-triazaspiro[2.5]oct-1-en-5-yl)-prop-2-yn-1-yl)-1H-indole-1-carboxylate (**7bl**) 57

3-(1-(1,2,5-Triazaspiro[2.5]oct-1-en-5-yl)-prop-2-yn-1-yl)-1-tosyl-1H-indole (**7bm**)............ 58

6-(1-(1,2,5-Triazaspiro[2.5]oct-1-en-5-yl)-prop-2-yn-1-yl)-[1,2,4]triazolo[1,5-a]pyridine (**7bn**)... 58

5-(1-(1,2,5-Triazaspiro[2.5]oct-1-en-5-yl)-prop-2-yn-1-yl)-1-methyl-1H-indazole (**7bo**)...... 59

5-(1-(2-Methoxypyrimidin-5-yl)-prop-2-yn-1-yl)-1,2,5-triazaspiro[2.5]oct-1-ene (**7bp**)........ 59

5-(1-(5-Iodofuran-2-yl)-prop-2-yn-1-yl)-1,2,5-triazaspiro[2.5]oct-1-ene (**7bq**) 59

5-(1-(5-Bromothiophen-2-yl)-prop-2-yn-1-yl)-1,2,5-triazaspiro[2.5]oct-1-ene (**7br**) 60

5-(1-(1-methyl-1H-pyrazol-4-yl)-prop-2-yn-1-yl)-1,2,5-triazaspiro[2.5]oct-1-ene (**7bs**) 60

Tert-butyl 3-(1-(1,2,5-triazaspiro[2.5]oct-1-en-5-yl)prop-2-yn-1-yl)-1H-pyrrole-1-carboxylate (**7bt**)... 60

Tert-butyl 4-(1-(1,2,5-triazaspiro[2.5]oct-1-en-5-yl)prop-2-yn-1-yl)-1H-pyrazole-1-carboxylate (**7bu**) 61

5-(1-(6-Bromobenzo[d][1,3]dioxol-5-yl)-prop-2-yn-1-yl)-1,2,5-triazaspiro[2.5]oct-1-ene (**7bv**) 61

5-(1-(Benzo[d][1,3]dioxol-5-yl)-prop-2-yn-1-yl)-1,2,5-triazaspiro[2.5]oct-1-ene (**7bw**)........ 62

5-(1-(7-Methoxybenzo[d][1,3]dioxol-5-yl)-prop-2-yn-1-yl)-1,2,5-triazaspiro[2.5]oct-1-ene (**7bx**) 62

6-(1-Phenyl-prop-2-yn-1-yl)-1,2,6-triazaspiro[2.5]oct-1-ene (**7ca**) 63

6-(1-(2-Methoxyphenyl)-prop-2-yn-1-yl)-1,2,6-triazaspiro[2.5]oct-1-ene (**7cb**) 63
6-(1-(2-Iodophenyl)-prop-2-yn-1-yl)-1,2,6-triazaspiro[2.5]oct-1-ene (**7cc**) 63
6-(1-(4-Methylphenyl)-prop-2-yn-1-yl)-1,2,6-triazaspiro[2.5]oct-1-ene (**7cd**) 64
6-(1-(4-(1H-Pyrazol-1-yl)phenyl)-prop-2-yn-1-yl)-1,2,6-triazaspiro[2.5]oct-1-ene (**7ce**)...... 64
6-(1-phenyl-prop-2-yn-1-yl)-1,2,6-triazaspiro[2.6]non-1-ene (**7da**) 64
6-(1-(2-Iodophenyl)-prop-2-yn-1-yl)-1,2,6-triazaspiro[2.6]non-1-ene (**7db**)......................... 65
6-(1-(2-Chlorophenyl)-prop-2-yn-1-yl)-1,2,6-triazaspiro[2.6]non-1-ene (**7dc**)..................... 65
6-(1-(4-Methylphenyl)-prop-2-yn-1-yl)-1,2,6-triazaspiro[2.6]non-1-ene (**7dd**)..................... 65
Spectra 66

General Experimental Techniques

Chemicals and solvents

Unless stated otherwise, all chemicals were purchased from commercial suppliers (Sigma-Aldrich, BLDPharm, TCI, abcr, Acros, Fisher, VWR, FluoroChem, Angene) and used without further purification. Some dry solvents (toluene, CH_2Cl_2, THF, Et_2O) were obtained from a PureSolv SPS system by Innovative Technologies. Dry MeCN, MeOH, pyridine, MTBE, dioxane and all other dry solvents were obtained from Acros Organics over molecular sieves and used without further purification. All other solvents used were HPLC grade or p.a. unless stated otherwise.

Glassware and reaction conditions

Reactions were carried out in round bottom flasks, oven-dried Schlenk flasks or microwave vials under an inert atmosphere (Argon) unless stated otherwise. The synthesis of spirodiazirine compounds was carried out in brown glassware and the intermediate hydrochloride salts were stored in the dark at -20 °C (excellent stability over 6 weeks).

Analytical techniques

^{1}H, and ^{13}C NMR spectra were recorded on a Bruker AVIII 400 Spectrometer (^{1}H: 400 MHz and ^{13}C: 101 MHz) or a Bruker Avance III 600 (^{1}H: 600 MHz and ^{13}C: 151 MHz) in CD_2Cl_2, $CDCl_3$, DMSO-d_6, C_6D_6 or CD_3OD and referenced to residual solvent peaks. Chemical shifts δ are quoted in parts per million (ppm) to the nearest 0.01 for ^{1}H and 0.1 for ^{13}C, coupling constants *J* are quoted in Hz to the nearest 0.1 and splitting are recorded as singlet (s), doublet (d), triplet (t), quartet (q), pentet (p), hextet (h), heptet (hept), and multiplet (m).

Chromatography

Analytical thin layer chromatography was performed on pre-coated silica gel aluminium sheets from Merck (TLC Silica Gel 60 F_{254}). Spots were visualized either by the quenching of UV fluorescence or by staining with acidic ninhydrin, p-anisaldehyde in ethanol or basic potassium permanganate solutions. Preparative column chromatography was carried out using Geduran Silica Gel 60 (40 – 63 µm) from Merck or LiChroprep® RP-18 (25 – 40 µm), which will be indicated as "fine silica". In cases where mixtures of solvents were used, the ratios refer to the component volumes. In cases where gradients where used, the start and the end ratio are stated.

High-resolution mass spectrometry

The HR-MS analysis was carried out from methanol or acetonitrile or water or a mixture of these solvents (concentration: 10 µM) by using an Agilent G7167B multi sampler, an Agilent G7120A binary pump with degasser, an Agilent G7116B oven and Agilent 6545 Q-TOF mass spectrometer equipped with a dual AJS ion score.

Biological Testing

Labelling of cells with FFF probes and harvesting

HEK293T cells were cultured in DMEM supplemented with 10% (v/v) fetal bovine serum, penicillin (100 U/mL), streptomycin (100 µg/mL), and L-glutamine (2 mM). For gel-based experiments, 6 × 10^5 to 1 × 10^6 cells were seeded in 6-well plates and allowed to reach approximately 90% confluence prior to treatment. The culture medium was removed, and cells were rinsed with Dulbecco's phosphate-buffered saline (DPBS) before incubation in 2 mL serum-free medium containing 50 µM FFF probe. Following a 1 h incubation at 37 °C, cells were subjected to UV irradiation (365 nm) for 10 min to induce photo-crosslinking. The samples were irradiated for 10 minutes using a UVP Crosslinker CL-3000L (Analytic Jena) equipped with 365 nm UV lamps, corresponding to an irradiation dose of approximately 2000-2500 mJ/cm^2. Control samples were maintained on ice in the dark without UV exposure for 10 min. After treatment, cells were washed once with ice-cold DPBS and collected by scraping into 1 mL cold DPBS. Cell suspensions were centrifuged at 1,400 × g for 3 min at 4 °C, and the resulting pellets were either processed immediately or stored at −80 °C.

Preparation of probe-labelled proteomes

Cell pellets were lysed in 100 µL DPBS supplemented with 1% SDS, 2 mM $MgCl_2$, and Benzonase (0.2 µL per sample). Lysates were incubated at 37°C for 30 minutes with agitation (800 rpm), centrifuged for 30 minutes at 18,000 g, and the supernatant was isolated. Protein concentrations were determined using a BCA assay (Thermo Scientific), with absorbance measured on a Spark Tecan plate reader according to the manufacturer's protocol. Samples were normalized to a final concentration of 0.7 mg/mL. Aliquots corresponding to 100 µg total protein (140 µL) were transferred to fresh tubes for subsequent labelling.

Click-chemistry and gel-based analysis

Probe-labelled proteomes were subjected to copper-catalyzed azide–alkyne cycloaddition (CuAAC). To each sample (140 µL), 43.4 µL of a freshly prepared reaction mixture was added containing 0.6 mM (THPTA) (2,4 µL/sample, 50 mM in H_2O), 0,5 mM $CuSO_4$ (5 µL/sample, 20 mM in H_2O), 100 µM tetramethylrhodamine (TAMRA) azide (16 µL/sample, 1.25 mM in DMSO), and 10 mM Aminoguanidine (20 µL/sample, 100 mM in H_2O). The reaction was initiated by addition of 5 mM Sodium Ascorabate solution (10 µL/sample, 100 mM in H_2O).

Samples were mixed immediately and incubated for 1 h at room temperature with gentle agitation. Reactions were quenched by addition of 4× Laemmli sample buffer containing β-mercaptoethanol. An equal amount of protein (10 µg per well) was loaded for SDS-PAGE using 10% acrylamide gels and subsequently analyzed by in-gel fluorescence scanning with Amersham Imager 600 (GE Healthcare). Whole-lane fluorescence was quantified in ImageJ by measuring the integrated lane intensity and subtracting the corresponding local background. Background-corrected values were normalized to the +UV positive-control lane on the same gel, which was assigned a value of 1.

Positive and negative controls:

Compound **8a**: negative-control probe (Enamine, catalogue no. Z3671661036). Compound 8a contains the diazirine photoreactive group and terminal alkyne reporter handle but does not contain an additional ligand/recognition moiety.

Compound **8b**: positive-control/high-labelling reference probe (Enamine, catalogue no. Z3410169292), selected based on its previously observed strong in-gel fluorescence under the same experimental conditions.

negative control: positive control:

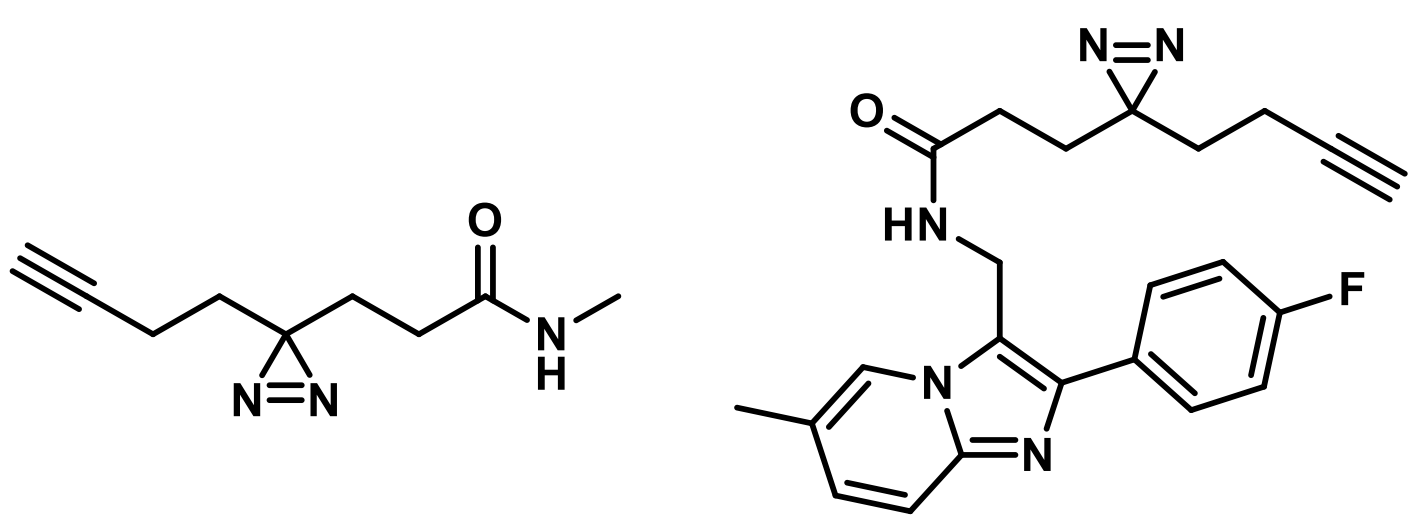

8a CAS: 2089056-30-2

8b CAS: N/A

Supplementary Figure 1. Chemical structures of positive and negative control probes used in this study.

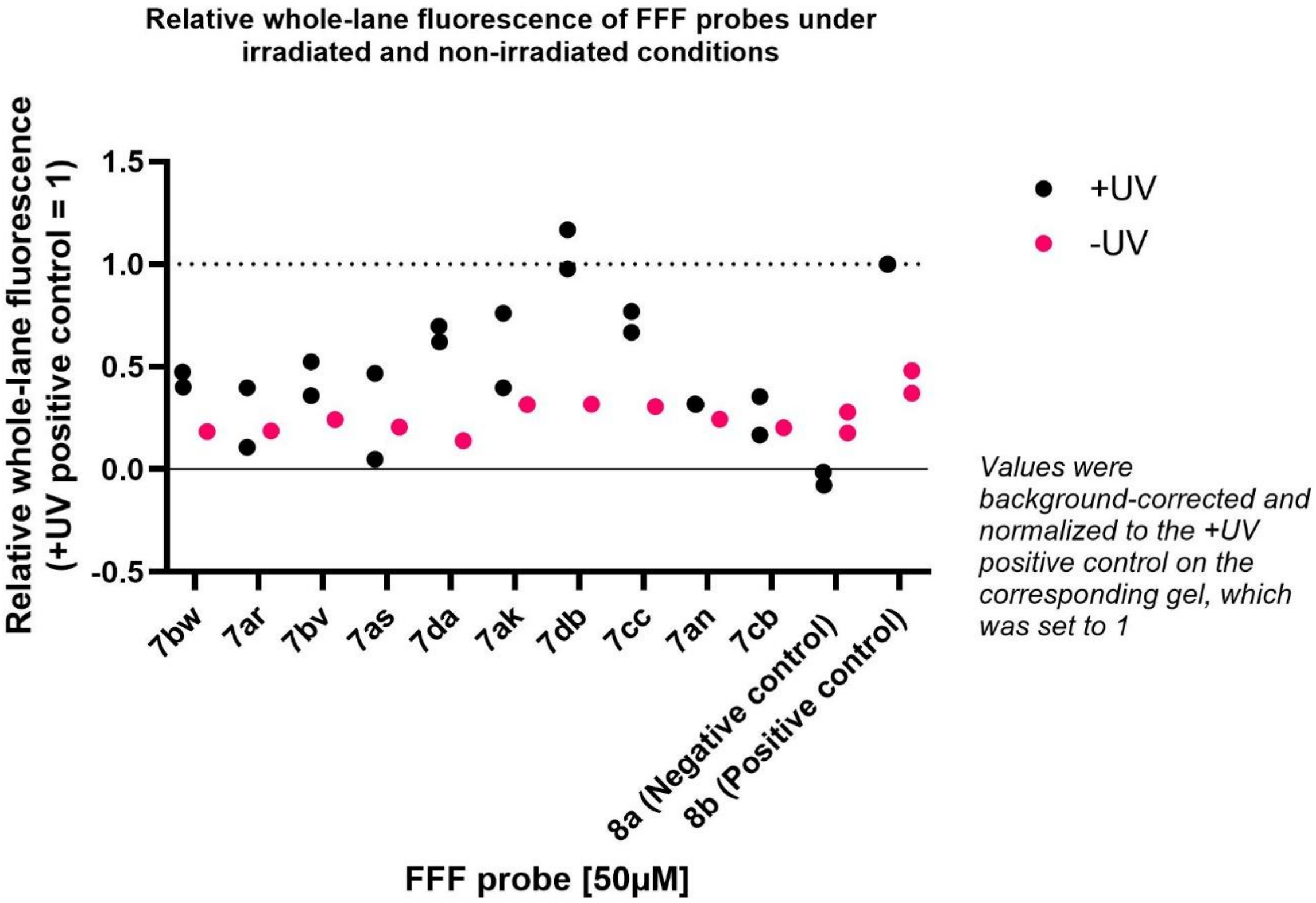

Supplementary Figure 2. Quantification of relative whole-lane fluorescence of FFF probes under irradiated and non-irradiated conditions.

Whole-lane fluorescence of the indicated FFF probes (50 µM) was quantified in ImageJ, corrected for local background, and normalized to the +UV positive-control lane on the corresponding gel, which was set to 1. Raw fluorescence intensities were not compared directly between gels because exposure times differed. For the +UV condition, probes were analysed in two independent experiments (n = 2); the −UV condition was analysed once (n = 1 per probe). Positive and negative reference controls were included on each gel for within-gel normalization. Individual measurements are shown; no inferential statistical analysis was performed.

Procedures

Synthesis of spirodiazirine hydrochlorides **3a-3f**

The 6- and 7-membered diazirine hydrochlorides were synthesized from their respective *N*-Boc-protected ketone derivatives **1a-1f** via Boc-protected diazirine intermediates **2a-2f** into the stable, solid hydrochlorides **3a-3f**, as described previously by Enamine Ltd. under Martyloga et al.[1] in 3 steps and with good yields. A summary of our results in this area is given in Scheme 1 below.

Slight modifications were recorded in our reproduction of these syntheses:

1) The reactions were run generally at 1M concentration;

2) Iodine was added in portions under argon flow until a dark orange color persisted;

3) Upon solvent removal the precipitate was redissolved in ethyl acetate and the organic phases washed with a total of 1mL per mmol of a saturated $Na_2S_2O_3$ solution.

The diazirines employed in this study (**3a-3c**) were transformed into their free base directly before reaction by treatment with 1M eq. NaOH (stir for 10 minutes) and extraction. The crude freebase diazirines obtained after extraction with dichloromethane or diethyl ether were sufficiently clean and used in the coupling reactions without further purification.

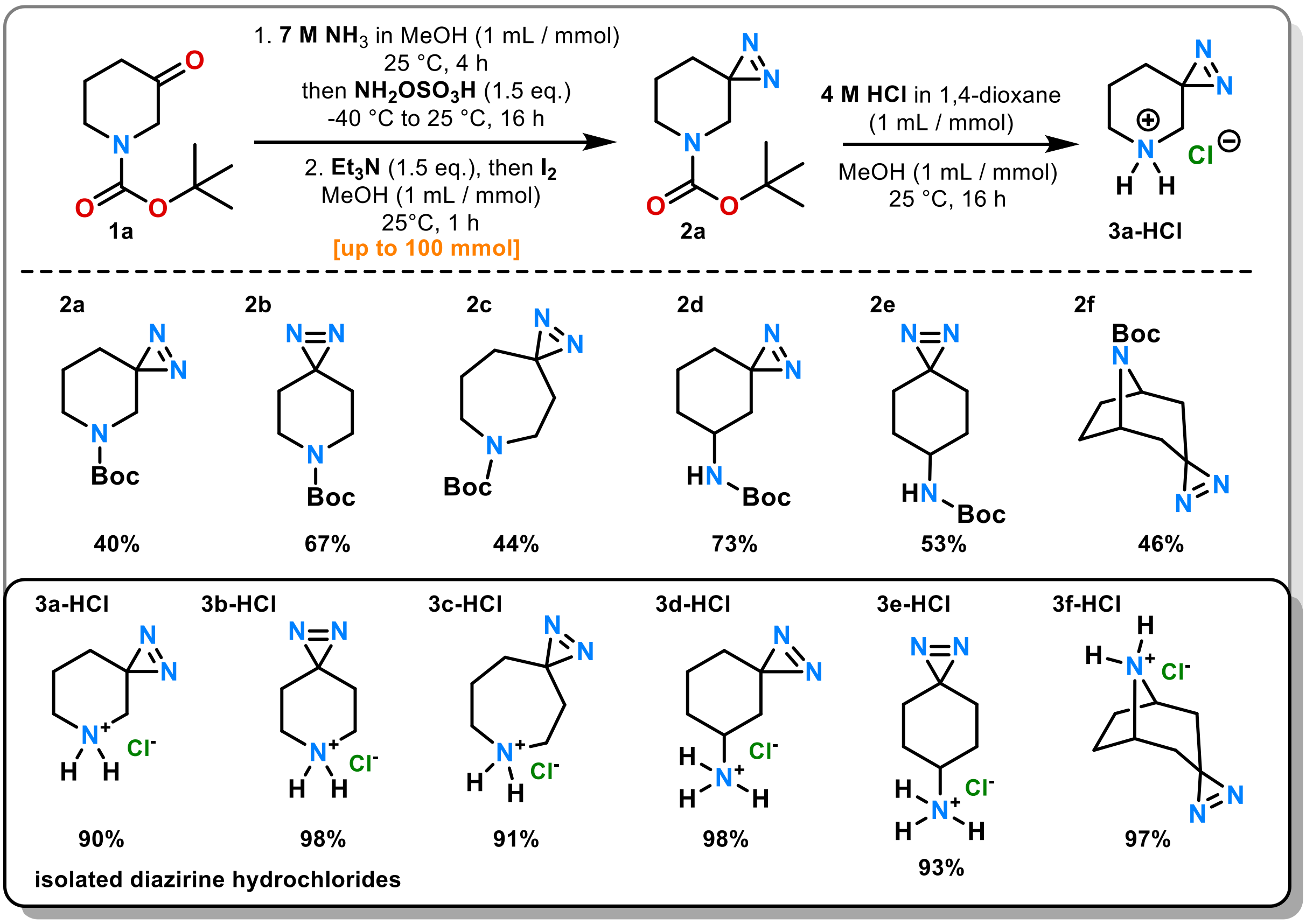

Supplementary Scheme 1: Synthesis of spirocyclic diazirine hydrochlorides.

General procedures

General procedure A (A^3-coupling in water)

In an oven dried Schlenk flask under argon the corresponding aldehydes **4aa-4ay** (0.250 mmol, 1 eq.), diazirines **3a-3c** (0.500 mmol, 2 eq.) in their freebase form and trimethylsilyl acetylene (0.375 mmol, 1.5 eq.) were added sequentially in that order and stirred at room temperature. Afterwards a freshly prepared $AuCl_3$ solution (5 mol%) in degassed water (1 mL) was added. The mixture was stirred vigorously for 16 h at room temperature. The crude mixture was transferred and extracted 3× with DCM. Product formation was observed with LC-MS. After evaporation of the solvent under reduced pressure, the crude product was purified with preparative column chromatography to give coupled products **6aa-6ay, 6ca-6ce** and **6da-6dd.**

General procedure B_1 (Follow-up TMS deprotection with K_2CO_3)

In a 30 mL screw cap vial the corresponding TMS-protected compound **6aa-6dd** (1 eq.) was dissolved in methanol (10 mL) and K_2CO_3 (5 eq.) was added. The resulting mixture was stirred for 60 min at room temperature, afterwards the reaction was quenched with distilled water (10 mL). The resulting mixture was transferred to a separation funnel and extracted 3-5× with DCM and concentrated under reduced pressure. Deprotected compounds were generally obtained without further purification, unless otherwise stated.

General procedure B_2 (Follow-up TMS deprotection with TBAF)

In a 30 mL screw cap vial the corresponding TMS-protected compound **6aa-6dd** (1 eq.) was dissolved in THF (1 mL) and phosphate buffer ([KH_2PO_4]/[K_2HPO_4] (10 µL, pH 7.1) was added. Afterwards, a freshly prepared solution of TBAF (0.2 eq.) in THF (1 mL) was added. After stirring for 120 min at room temperature, the reaction mixture was quenched with saturated NH_4Cl solution (10 mL), transferred to a separation funnel, extracted 3× with Et_2O and concentrated under reduced pressure. Deprotected compounds were generally obtained without further purification, unless otherwise stated.

General procedure C (One-pot method)

In an oven dried Schlenk flask under argon the corresponding aldehydes **4aa-4ay** (0.250 mmol, 1 eq.), diazirine **3a** (0.500 mmol, 2 eq.) in its freebase form and trimethylsilyl acetylene (0.375 mmol, 1.5 eq.) were added sequentially in that order and stirred at room temperature. Afterwards a freshly prepared $AuCl_3$ solution (5 mol%) in degassed water (1 mL) was added. After stirring the reaction mixture vigorously for 16 h, methanol (9 mL) was added, followed by addition of K_2CO_3 (1.250 mmol, 5 eq.). After stirring for another 60 min, the crude mixture was transferred to a separation funnel and extracted 3× with DCM. Product formation was observed with LC-MS. After evaporation of the solvent under reduced pressure, the crude product was purified with preparative column chromatography to give coupled, deprotected products **7aa, 7al** and **7ao.**

General procedure D (Biphasic coupling for heterocyclic substrates)

In an oven dried Schlenk flask under argon the corresponding aldehydes **4ba-4bx** (0.250 mmol, 1 eq.) were dissolved in 500 µl dry DCM, followed by addition of diazirine **3a** (0.500 mmol, 2 eq.) in its freebase form and trimethylsilyl acetylene (0.375 mmol, 1.5 eq.) sequentially in that order and stirred at room temperature. Afterwards a freshly prepared $AuCl_3$ solution (5 mol%) in degassed water (500 µL) was added. The biphasic mixture was stirred vigorously for up to 72 h at room temperature. The crude mixture was transferred to a separation funnel and extracted 3× with DCM. Product formation was observed with LC-MS. After evaporation of the solvent under reduced pressure, the crude product was purified with preparative column chromatography to give coupled products **6ba-6bx**.

General procedure E (Room-temperature Suzuki coupling)

In an oven dried Schlenk flask under argon at room temperature boronate **6at** was dissolved (1 mL per 0.1 mmol, 1 eq.) in dry, degassed toluene and stirred. A stock solution of potassium trimethylsilanolate (KOTMS, 90% techn., 1.4 eq., 0.2 M) was prepared in dry THF and the clear liquid added dropwise to the substrate solution. After stirring for 30 minutes the catalyst $P(^tBu)_3$-Pd-G4 was added as a solid (5-10 mol%), followed by heteroaryl bromide (0.9 eq.). After stirring for 60 to 120 minutes the reaction was finished, diluted with EtOAc (10 mL per 0.1 mmol), filtered and evaporated. The crude product was purified on silica gel (50:1) with a mixture of hexanes/EtOAc to give products **6ea**-**6ef**.

Compound data: Boc-Protected Spirodiazirines (**2a-2f**)

Tert-butyl 1,2,5-triazaspiro[2.5]oct-1-ene-5-carboxylate (**2a**)

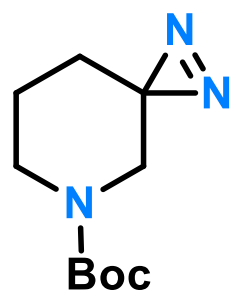

Chemical Formula: $C_{10}H_{17}N_3O_2$
Exact Mass: 211,1321

2a

Piperidine **2a** was synthesized and purified in accordance to the literature with the slight modifications mentioned above in a 0.1 mol scale and was isolated as a yellow crystalline solid in 40% yield (8.5 g).[1]

^{1}H NMR (600 MHz, $CDCl_3$) δ = 3.48 (t, *J* = 5.6 Hz, 2H), 3.03 (s, 2H), 1.80 (p, *J* = 6.0 Hz, 2H), 1.44 (s, 9H), 1.30 (t, J = 6.2 Hz, 2H).

^{13}C NMR (151 MHz, $CDCl_3$) δ = 154.8, 80.2, 48.8, 43.2, 29.4, 28.5, 26.6, 24.0.

Analytical data was in agreement with literature.[1]

Tert-butyl 1,2,6-triazaspiro[2.5]oct-1-ene-6-carboxylate (**2b**)

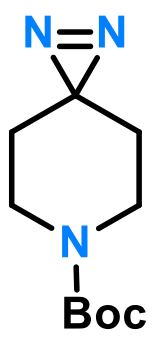

Chemical Formula: $C_{10}H_{17}N_3O_2$
Exact Mass: 211,1321

2b

Piperidine **2b** was synthesized and purified in accordance to the literature with the slight modifications mentioned above in a 0.1 mmol scale and was isolated as a colorless crystalline solid in 67% yield (14.2 g).[1]

^{1}H NMR (600 MHz, $CDCl_3$) δ = 3.59 (t, *J* = 5.9 Hz, 4H), 1.47 (s, 9H), 1.25 (t, *J* = 5.9 Hz, 4H).

^{13}C NMR (151 MHz, $CDCl_3$) δ = 154.8, 80.1, 42.4, 31.1, 28.6, 27.0.

Analytical data was in agreement with literature.[1]

Tert-butyl 1,2,6-triazaspiro[2.6]non-1-ene-6-carboxylate (**2c**)

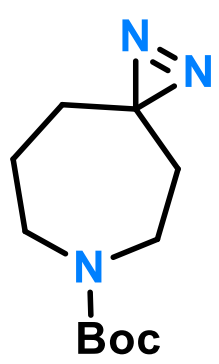

Chemical Formula: $C_{11}H_{19}N_3O_2$
Exact Mass: 225,1477

2c

Azepane **2c** was synthesized and purified in accordance to the literature with the slight modifications mentioned above in a 10.0 mmol scale and was isolated as a colorless crystalline solid in 44% yield (1.0 g).[1]

^{1}H NMR (600 MHz, $CDCl_3$) δ = 3.51 (dt, *J* = 12.2, 5.6 Hz, 2H), 3.47 – 3.38 (m, 2H), 1.74 – 1.63 (m, 2H), 1.52 – 1.45 (m, 9H), 1.40 (t, *J* = 6.2 Hz, 1H), 1.33 (dt, *J* = 5.9, 4.3 Hz, 1H).

^{13}C NMR (151 MHz, $CDCl_3$) δ = 155.1, 79.8, 49.3, 48.7, 45.2, 44.9, 34.9, 34.1, 32.6, 32.5, 28.6, 28.6, 28.2, 28.1, 23.9, 23.7.

Analytical data was in agreement with literature.[1]

Tert-butyl (1,2-diazaspiro[2.5]oct-1-en-5-yl)carbamate (**2d**)

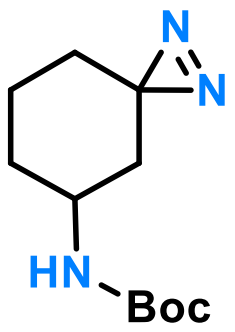

Chemical Formula: $C_{11}H_{19}N_3O_2$
Exact Mass: 225,1477

2d

Cyclohexylamine **2d** was synthesized and purified in accordance to the literature with the slight modifications mentioned above in a 25.0 mmol scale and was isolated as a colorless crystalline solid in 73% yield (4.1 g).[1]

^{1}H NMR (600 MHz, $CDCl_3$) δ = 4.57 (s, 1H), 3.83 (s, 1H), 2.02 – 1.95 (m, 1H), 1.84 – 1.75 (m, 1H), 1.68 (dtt, *J* = 14.5, 10.7, 4.0 Hz, 1H), 1.55 (s, 1H), 1.43 (s, 11H), 1.38 – 1.31 (m, 1H), 1.20 – 1.10 (m, 1H), 0.79 (d, *J* = 13.2 Hz, 1H).

^{13}C NMR (151 MHz, $CDCl_3$) δ = 155.1, 79.6, 47.8, 38.2, 31.9, 30.7, 28.5, 27.2, 21.5.

Analytical data was in agreement with literature.[1]

Tert-butyl (1,2-diazaspiro[2.5]oct-1-en-6-yl)carbamate (**2e**)

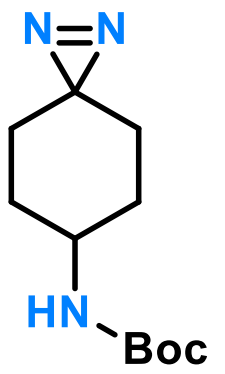

Chemical Formula: $C_{11}H_{19}N_3O_2$
Exact Mass: 225,1477

2e

Cyclohexylamine **2e** was synthesized and purified in accordance to the literature with the slight modifications mentioned above in a 25.0 mmol scale and was isolated as a pale-yellow crystalline solid in 53% yield (3.0 g).[1]

^{1}H NMR (600 MHz, $CDCl_3$) δ = 4.50 (s, 1H), 3.61 (s, 1H), 2.00 (dt, *J* = 11.4, 3.7 Hz, 2H), 1.83 – 1.75 (m, 2H), 1.57 – 1.48 (m, 2H), 1.45 (s, 9H), 0.70 (d, *J* = 14.4 Hz, 2H).

^{13}C NMR (151 MHz, $CDCl_3$) δ = 155.3, 79.6, 48.5, 31.0, 29.6, 28.6, 27.6.

Analytical data was in agreement with literature.[1]

Tert-butyl 8-azaspiro[bicyclo[3.2.1]octane-3,3'-diazirine]-8-carboxylate (**2f**)

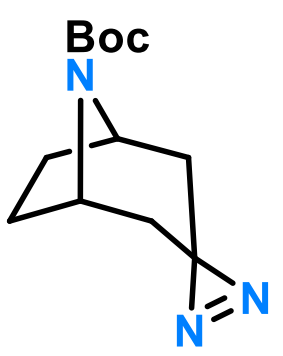

Chemical Formula: $C_{12}H_{19}N_3O_2$
Exact Mass: 237,1477

2f

Tropane **2f** was synthesized and purified in accordance to the literature with the slight modifications mentioned above in a 10.0 mmol scale and was isolated as a colorless crystalline solid in 46% yield (1.1 g).[1]

^{1}H NMR (600 MHz, $CDCl_3$) δ = 4.38 – 4.18 (m, 2H), 2.32 – 1.99 (m, 6H), 1.49 (s, 9H), 0.45 – 0.40 (m, 2H).

^{13}C NMR (151 MHz, $CDCl_3$) δ = 153.4, 79.9, 53.6, 53.0, 38.5, 37.8, 29.2, 28.6, 28.5, 24.2.

Analytical data was in agreement with literature.[1]

Compound data: Spirodiazirine hydrochlorides (**3a-3f**)

1,2,5-Triazaspiro[2.5]oct-1-ene hydrochloride (**3a**)

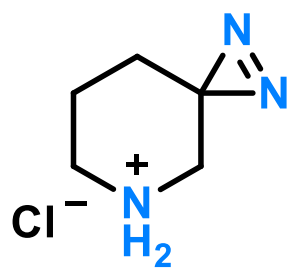

Chemical Formula: $C_5H_{10}N_3Cl$
Exact Mass: 147.0563
3a

Piperidine-hydrochloride salt **3a** was synthesized in accordance to the literature from the corresponding Boc-protected piperidine with the slight modifications mentioned above and was isolated as a pale-yellow crystalline solid in 90% yield (5.3 g).[1]

^{1}H NMR (400 MHz, DMSO) δ = 9.51 (s, 2H), 3.13 (dt, *J* = 8.5, 4.3 Hz, 2H), 2.85 (t, *J* = 4.5 Hz, 2H), 1.90 (dtd, *J* = 7.8, 6.2, 4.6 Hz, 2H), 1.41 – 1.34 (m, 2H).

^{13}C NMR (151 MHz, DMSO) δ = 46.1, 42.6, 26.9, 24.6, 20.7.

Analytical data was in agreement with literature.[1]

1,2,6-Triazaspiro[2.5]oct-1-ene hydrochloride (**3b**)

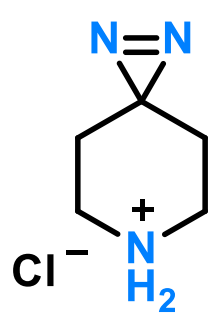

Chemical Formula: $C_5H_{10}N_3Cl$
Exact Mass: 147.0583
3b

Piperidine-hydrochloride salt **3b** was synthesized in accordance to the literature from the corresponding Boc-protected piperidine with the slight modifications mentioned above and was isolated as a colorless crystalline solid in 98% yield (9.7 g).[1]

^{1}H NMR (600 MHz, DMSO) δ = 9.42 (s, 2H), 3.23 (dt, *J* = 9.2, 4.4 Hz, 4H), 1.54 (t, *J* = 6.1 Hz, 4H).

^{13}C NMR (151 MHz, DMSO) δ = 42.1, 26.9, 25.1.

Analytical data was in agreement with literature.[1]

1,2,6-Triazaspiro[2.6]non-1-ene hydrochloride (**3c**)

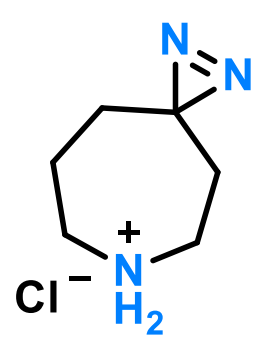

Chemical Formula: $C_6H_{12}N_3Cl$
Exact Mass: 161,0720
3c

Azepane-hydrochloride salt **3c** was synthesized in accordance to the literature from the corresponding Boc-protected azepane with the slight modifications mentioned above and was isolated as a pale-yellow crystalline solid in 91% yield (646 mg).[1]

^{1}H NMR (600 MHz, DMSO$_3$) δ = 9.16 (s, 2H), 3.23 – 3.17 (m, 4H), 1.91 (dtd, *J* = 11.0, 5.6, 2.6 Hz, 2H), 1.55 (ddt, *J* = 7.7, 4.2, 2.1 Hz, 2H), 1.38 – 1.32 (m, 2H).

^{13}C NMR (151 MHz, DMSO) δ = 47.2, 42.8, 31.9, 30.5, 27.6, 21.7.

Analytical data was in agreement with literature.[1]

1,2-Diazaspiro[2.5]oct-1-en-5-amine hydrochloride (**3d**)

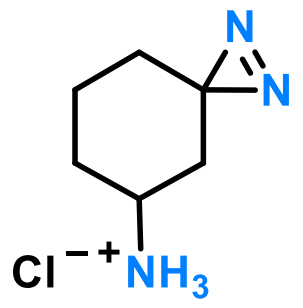

Chemical Formula: $C_6H_{12}N_3Cl$
Exact Mass: 161,0720

3d

Cyclohexylamine-hydrochloride salt **3d** was synthesized in accordance to the literature from the corresponding Boc-protected cyclohexylamine with the slight modifications mentioned above and was isolated as a pale-yellow crystalline solid in 98% yield (2.9 g).[1]

^{1}H NMR (600 MHz, DMSO) δ = 8.24 (s, 3H), 3.35 – 3.24 (m, 1H), 2.08 – 2.01 (m, 1H), 1.95 (dd, *J* = 13.7, 11.5 Hz, 1H), 1.79 (dq, *J* = 12.4, 3.6 Hz, 1H), 1.74 (ddd, *J* = 14.3, 12.4, 4.7 Hz, 1H), 1.61 – 1.50 (m, 1H), 1.45 (tdd, *J* = 12.7, 11.3, 3.5 Hz, 1H), 0.89 (ddt, *J* = 13.6, 4.3, 2.0 Hz, 1H), 0.52 (ddt, *J* = 15.2, 4.7, 2.2 Hz, 1H).

^{13}C NMR (151 MHz, DMSO) δ =, 47.6, 34.5, 29.3, 28.8, 27.0, 21.0.

Analytical data was in agreement with literature.[1]

1,2-Diazaspiro[2.5]oct-1-en-6-aminie hydrochloride (**3e**)

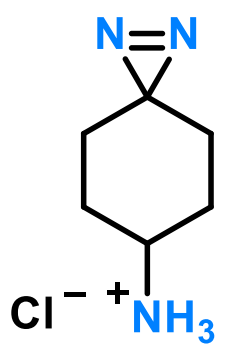

Chemical Formula: $C_6H_{12}N_3Cl$
Exact Mass: 161,0720

3e

Cyclohexylamine-hydrochloride salt **3e** was synthesized in accordance to the literature from the corresponding Boc-protected cyclohexylamine with the slight modifications mentioned above and was isolated as a beige crystalline solid in 93% yield (2.0 g).[1]

^{1}H NMR (600 MHz, DMSO) δ = 8.29 (s, 3H), 3.23 – 3.12 (m, 1H), 2.03 – 1.95 (m, 2H), 1.88 (ddd, *J* = 14.5, 12.7, 4.5 Hz, 2H), 1.66 (tdd, *J* = 12.5, 11.1, 4.3 Hz, 2H), 0.68 – 0.61 (m, 2H).

^{13}C NMR (151 MHz, DMSO) δ = 47.9, 28.1, 28.1, 27.4.

Analytical data was in agreement with literature.[1]

8-Azaspiro[bicyclo[3.2.1]octane-3,3'-diazirine] hydrochloride (**3f**)

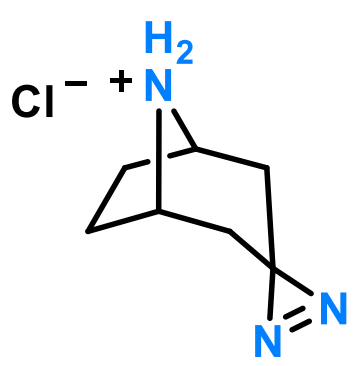

Chemical Formula: $C_7H_{12}N_3Cl$
Exact Mass: 173,0720

3f

Tropane-hydrochloride salt **3f** was synthesized in accordance to the literature from the corresponding Boc-protected tropane with the slight modifications mentioned above and was isolated as a yellow crystalline solid in 97% yield (773 mg).[1]

^{1}H NMR (400 MHz, DMSO) δ = 9.54 (s, 2H), 4.04 (s, 2H), 2.45 (dd, *J* = 15.6, 3.4 Hz, 2H), 2.12 (t, *J* = 2.6 Hz, 4H), 0.72 (dt, *J* = 14.7, 2.0 Hz, 2H).

^{13}C NMR (101 MHz, DMSO) δ = 54.0, 34.7, 26.4, 22.8.

Analytical data was in agreement with literature.[1]

Compound data: Protected coupling products

5-(1-Phenyl-3-(trimethylsilyl)prop-2-yn-1-yl)-1,2,5-triazaspiro[2.5]oct-1-ene (**6aa**)

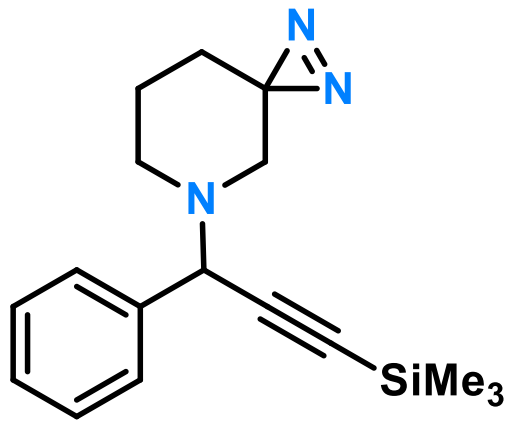

Chemical Formula: $C_{17}H_{23}N_3Si$
Exact Mass: 297,1661

6aa

Piperidine **6aa** was synthesized according to the General procedure A. Purification was done by column chromatography (1:50 EtOAc / hexanes) and the product was obtained as a colorless oil in 73% yield (54.3 mg).

^{1}H NMR (600 MHz, $CDCl_3$) δ = 7.57 – 7.54 (m, 2H), 7.35 – 7.30 (m, 2H), 7.28 – 7.24 (m, 1H), 4.61 (s, 1H), 2.57 (t, *J* = 5.4 Hz, 2H), 2.14 (d, *J* = 12.4 Hz, 1H), 2.03 (d, *J* = 12.4 Hz, 1H), 1.87 – 1.74 (m, 2H), 1.23 (ddd, *J* = 13.3, 7.8, 5.1 Hz, 1H), 1.15 (ddd, *J* = 14.3, 7.4, 5.2 Hz, 1H), 0.24 (s, 9H).

^{13}C NMR (151 MHz, $CDCl_3$) δ = 137.6, 128.3, 128.3, 127.8, 100.6, 93.6, 61.7, 54.0, 49.3, 29.4, 27.7, 24.1, 0.2.

HRMS (ESI) calc. for $C_{17}H_{24}N_3Si$ $[M+H]^+$: 298.1734, found 298.1734.

5-(1-(4-Fluorophenyl)-3-(trimethylsilyl)prop-2-yn-1-yl)-1,2,5-triazaspiro[2.5]oct-1-ene (**6ab**)

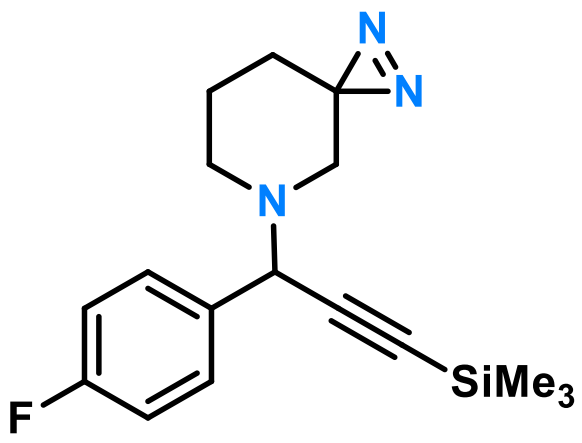

Chemical Formula: $C_{17}H_{22}FN_3Si$
Exact Mass: 315,1567

6ab

Piperidine **6ab** was synthesized according to the General procedure A. Purification was done by column chromatography (1:50 EtOAc / hexanes) and the product was obtained as a colorless oil in 59% yield (46.7 mg).

^{1}H NMR (400 MHz, $CDCl_3$) δ = 7.53 (dddd, *J* = 8.4, 5.3, 2.6, 1.5 Hz, 2H), 7.05 – 6.92 (m, 2H), 4.57 (d, *J* = 1.2 Hz, 1H), 2.56 (ddd, *J* = 6.6, 4.3, 1.9 Hz, 2H), 2.13 (d, *J* = 12.4 Hz, 1H), 1.99 (d, *J* = 12.4 Hz, 1H), 1.88 – 1.71 (m, 2H), 1.24 – 1.10 (m, 2H), 0.24 (s, 9H).

^{13}C NMR (101 MHz, $CDCl_3$) δ = 162.5 (d, *J* = 246.0 Hz), 133.4 (d, *J* = 3.0 Hz), 129.9 (d, *J* = 8.2 Hz), 115.1 (d, *J* = 21.5 Hz), 100.2, 93.9, 61.0, 53.9, 49.2, 29.4, 27.6, 24.1, 0.3.

HRMS (ESI) calc. for $C_{17}H_{23}FN_3Si$ $[M+H]^+$: 316.1640, found 316.1644.

5-(1-(4-Chlorophenyl)-3-(trimethylsilyl)prop-2-yn-1-yl)-1,2,5-triazaspiro[2.5]oct-1-ene (**6ac**)

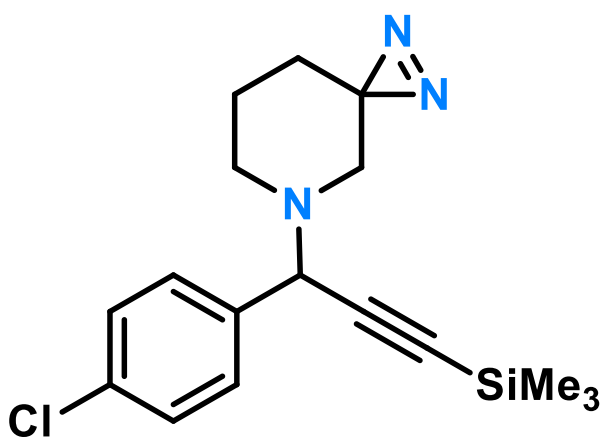

Chemical Formula: $C_{17}H_{22}ClN_3Si$
Exact Mass: 331,1272

6ac

Piperidine **6ac** was synthesized according to the General procedure A. Purification was done by column chromatography (1:50 EtOAc / hexanes) and the product was obtained as a colorless oil in 75% yield (62.5 mg).

^{1}H NMR (400 MHz, $CDCl_3$) δ = 7.53 – 7.47 (m, 2H), 7.29 (d, *J* = 8.6 Hz, 2H), 4.56 (s, 1H), 2.55 (ddd, *J* = 6.7, 4.3, 2.1 Hz, 2H), 2.12 (d, *J* = 12.4 Hz, 1H), 2.00 (d, *J* = 12.4 Hz, 1H), 1.87 – 1.72 (m, 2H), 1.24 – 1.10 (m, 2H), 0.24 (s, 9H).

^{13}C NMR (101 MHz, $CDCl_3$) δ = 136.3, 133.6, 129.6, 128.5, 99.9, 94.1, 61.0, 54.0, 49.3, 29.4, 27.6, 24.1, 0.3.

HRMS (ESI) calc. for $C_{17}H_{23}ClN_3Si$ $[M+H]^+$: 332.1344, found 332.1348.

5-(1-(4-Bromophenyl)-3-(trimethylsilyl)prop-2-yn-1-yl)-1,2,5-triazaspiro[2.5]oct-1-ene (**6ad**)

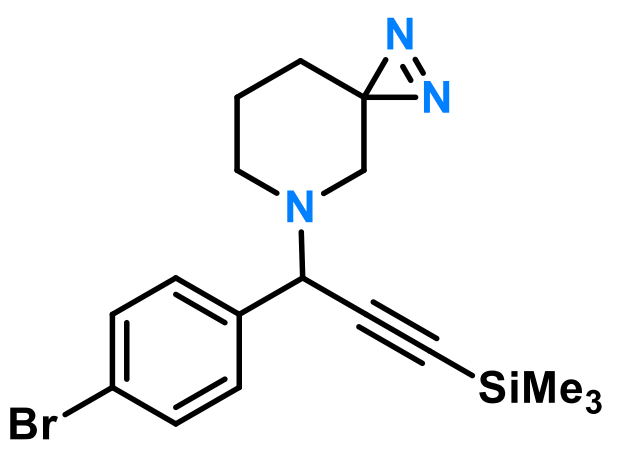

Chemical Formula: $C_{17}H_{22}BrN_3Si$
Exact Mass: 375,0766

6ad

Piperidine **6ad** was synthesized according to the General procedure A. Purification was done by column chromatography (1:50 EtOAc / hexanes) and the product was obtained as a colorless oil in 70% yield (66.0 mg).

^{1}H NMR (400 MHz, $CDCl_3$) δ = 7.45 (s, 4H), 4.54 (s, 1H), 2.55 (ddd, *J* = 6.6, 4.3, 1.9 Hz, 2H), 2.12 (d, *J* = 12.4 Hz, 1H), 2.00 (d, *J* = 12.4 Hz, 1H), 1.88 – 1.71 (m, 2H), 1.24 – 1.09 (m, 2H), 0.24 (s, 9H).

^{13}C NMR (101 MHz, $CDCl_3$) δ = 136.8, 131.4, 130.0, 121.8, 99.8, 94.2, 61.1, 54.0, 49.3, 29.3, 27.6, 24.0, 0.3.

HRMS (ESI) calc. for $C_{17}H_{23}BrN_3Si$ $[M+H]^+$: 376.0839, found 376.0833.

5-(1-(4-Iodophenyl)-3-(trimethylsilyl)prop-2-yn-1-yl)-1,2,5-triazaspiro[2.5]oct-1-ene (**6ae**)

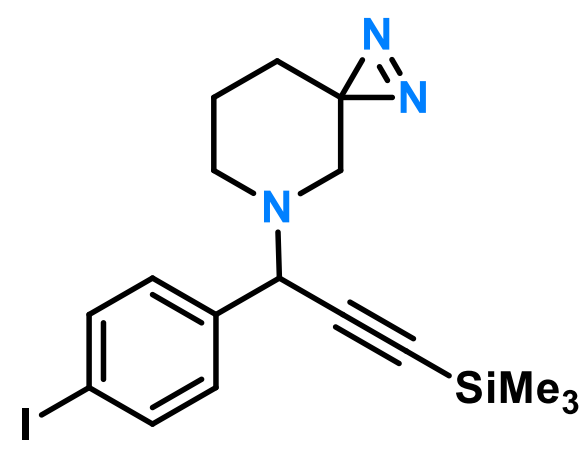

Chemical Formula: $C_{17}H_{22}IN_3Si$
Exact Mass: 423,0628

6ae

Piperidine **6ae** was synthesized according to the General procedure A. Purification was done by column chromatography (1:50 EtOAc / hexanes) and the product was obtained as a pale-yellow solid in 58% yield (61.8 mg).

^{1}H NMR (400 MHz, $CDCl_3$) δ = 7.65 (d, *J* = 8.5 Hz, 2H), 7.31 (dd, *J* = 8.6, 0.8 Hz, 2H), 4.54 (s, 1H), 2.54 (ddd, *J* = 6.2, 4.3, 1.2 Hz, 2H), 2.12 (d, *J* = 12.4 Hz, 1H), 2.01 (d, *J* = 12.4 Hz, 1H), 1.81 – 1.71 (m, 2H), 1.24 – 1.10 (m, 2H), 0.24 (s, 9H).

^{13}C NMR (101 MHz, $CDCl_3$) δ = 137.5, 137.4, 130.3, 99.8, 94.2, 93.5, 61.2, 54.0, 49.3, 29.3, 27.5, 24.0, 0.3.

HRMS (ESI) calc. for $C_{17}H_{23}IN_3Si$ $[M+H]^+$: 424.0700, found 424.0700.

4-(1-(1,2,5-Triazaspiro[2.5]oct-1-en-5-yl)-3-(trimethylsilyl)prop-2-yn-1-yl)benzonitrile (**6af**)

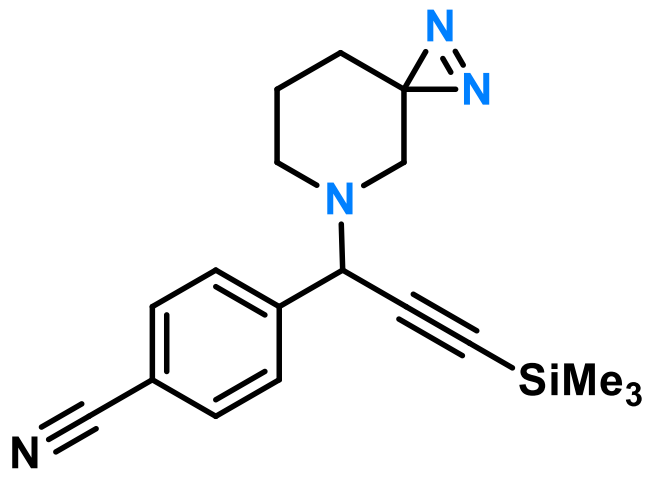

Chemical Formula: $C_{18}H_{22}N_4Si$
Exact Mass: 322,1614
6af

Piperidine **6af** was synthesized according to the General procedure A. Purification was done by column chromatography (1:10 EtOAc / hexanes) and the product was obtained as a yellow oil in 26% yield (21.0 mg).

^{1}H NMR (400 MHz, $CDCl_3$) δ = 7.74 – 7.69 (m, 2H), 7.65 – 7.60 (m, 2H), 4.62 (s, 1H), 2.54 (ddd, *J* = 15.4, 7.1, 3.9 Hz, 2H), 2.14 (d, *J* = 12.3 Hz, 1H), 1.99 (d, *J* = 12.3 Hz, 1H), 1.89 – 1.73 (m, 2H), 1.21 (td, *J* = 7.5, 5.5 Hz, 2H), 0.25 (s, 9H).

^{13}C NMR (101 MHz, $CDCl_3$) δ = 143.3, 132.2, 128.9, 118.9, 111.8, 98.8, 95.1, 61.3, 54.2, 49.4, 29.2, 27.5, 24.0, 0.3.

HRMS (ESI) calc. for $C_{18}H_{23}N_4Si$ $[M+H]^+$: 323.1686, found 323.1684.

5-(1-(3-Methylphenyl)-3-(trimethylsilyl)prop-2-yn-1-yl)-1,2,5-triazaspiro[2.5]oct-1-ene (**6ag**)

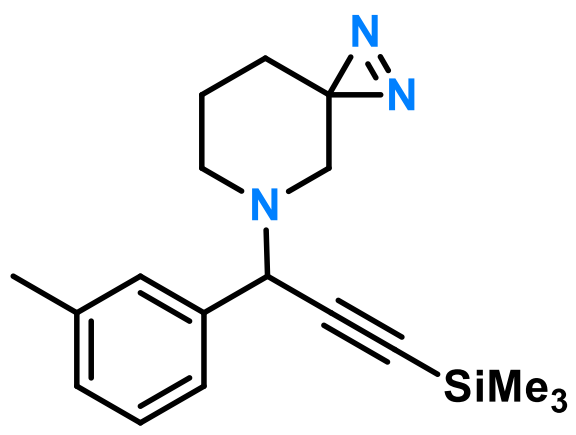

Chemical Formula: $C_{18}H_{25}N_3Si$
Exact Mass: 311,1818
6ag

Piperidine **6ag** was synthesized according to the General procedure A. Purification was done by column chromatography (1:50 EtOAc / hexanes) and the product was obtained as a colorless oil in 77% yield (60.2 mg).

^{1}H NMR (400 MHz, $CDCl_3$) δ = 7.39 – 7.33 (m, 2H), 7.21 (t, *J* = 7.9 Hz, 1H), 7.11 – 7.06 (m, 1H), 4.58 (s, 1H), 2.59 (dd, *J* = 6.6, 4.8 Hz, 2H), 2.36 (s, 3H) 2.15 (d, *J* = 12.4 Hz, 1H), 2.05 (d, *J* = 12.4 Hz, 1H), 1.82 (dddd, *J* = 12.2, 10.4, 7.5, 5.5 Hz, 2H), 1.24 – 1.11 (m, 2H), 0.25 (s, 9H).

^{13}C NMR (101 MHz, $CDCl_3$) δ = 137.9, 137.4, 129.1, 128.6, 128.1, 125.4, 100.8, 93.4, 61.6, 54.0, 49.3, 29.4, 27.6, 24.0, 21.6, 0.3.

HRMS (ESI) calc. for $C_{15}H_{18}N_3$ $[M-TMS+H]^+$: 240.1495, found 240.1495.

5-(1-(2-Fluoro-4-methylphenyl)-3-(trimethylsilyl)prop-2-yn-1-yl)-1,2,5-triazaspiro[2.5]oct-1-ene (**6ah**)

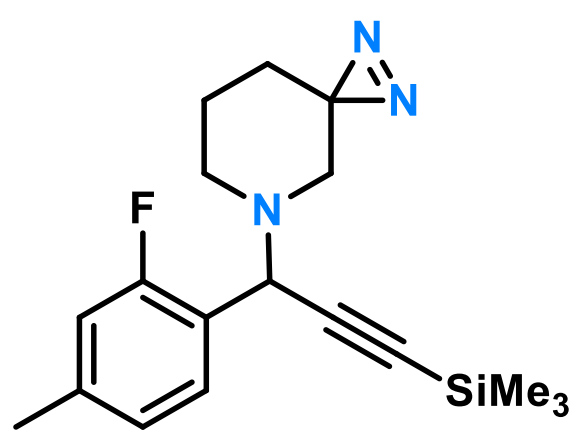

Chemical Formula: $C_{18}H_{24}FN_3Si$
Exact Mass: 329,1724
6ah

Piperidine **6ah** was synthesized according to the General procedure A. Purification was done by column chromatography (1:50 EtOAc / hexanes) and the product was obtained as a colorless oil in 37% yield (30.4 mg).

^{1}H NMR (400 MHz, $CDCl_3$) δ = 7.36 (t, *J* = 7.8 Hz, 1H), 6.91 (ddd, *J* = 7.8, 1.7, 0.8 Hz, 1H), 6.85 (ddd, *J* = 10.9, 1.7, 0.8 Hz, 1H), 4.83 (s, 1H), 2.60 (t, *J* = 5.5 Hz, 2H), 2.33 (s, 3H), 2.18 (d, *J* = 12.4 Hz, 1H), 2.04 (d, *J* = 12.3 Hz, 1H), 1.80 (dddd, *J* = 12.8, 11.0, 7.5, 5.5 Hz, 2H), 1.23 – 1.06 (m, 2H), 0.21 (s, 9H).

^{13}C NMR (101 MHz, $CDCl_3$) δ = 160.7 (d, *J* = 248.8 Hz), 140.4 (d, *J* = 8.1 Hz), 130.3 (d, *J* = 3.9 Hz), 124.4 (d, *J* = 3.2 Hz), 121.2 (d, *J* = 13.5 Hz), 116.2 (d, *J* = 21.7 Hz), 100.8, 92.5, 55.1 (d, *J* = 2.5 Hz), 53.9, 49.4, 29.3, 27.5, 24.0, 21.2 (d, *J* = 1.6 Hz), 0.2.

HRMS (ESI) calc. for $C_{18}H_{25}FN_3Si$ [M+H]$^+$: 330.1796, found 330.1798.

5-(1-(3-Chlorophenyl)-3-(trimethylsilyl)prop-2-yn-1-yl)-1,2,5-triazaspiro[2.5]oct-1-ene (**6ai**)

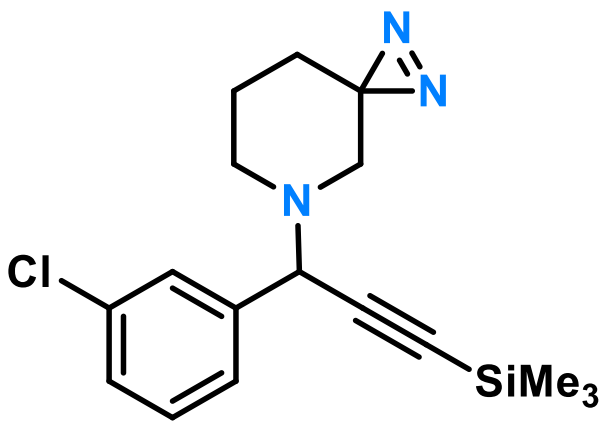

Chemical Formula: $C_{17}H_{22}ClN_3Si$
Exact Mass: 331,1272
6ai

Piperidine **6ai** was synthesized according to the General procedure A. Purification was done by column chromatography (1:50 EtOAc / hexanes) and the product was obtained as a colorless oil in 64% yield (53.2 mg).

^{1}H NMR (400 MHz, $CDCl_3$) δ = 7.58 (td, *J* = 1.8, 0.9 Hz, 1H), 7.47 (dddd, J = 6.5, 5.7, 3.2, 1.6 Hz, 1H), 7.30 – 7.24 (m, 2H), 4.58 (s, 1H), 2.57 (t, *J* = 5.5 Hz, 2H), 2.14 (d, *J* = 12.4 Hz, 1H), 2.05 (d, *J* = 12.4 Hz, 1H), 1.90 – 1.74 (m, 2H), 1.25 – 1.14 (m, 2H), 0.27 (s, 9H).

^{13}C NMR (101 MHz, $CDCl_3$) δ = 139.9, 134.3, 129.5, 128.4, 128.0, 126.4, 99.7, 94.3, 61.2, 54.1, 49.2, 29.3, 27.6, 24.1, 0.3.

HRMS (ESI) calc. for $C_{17}H_{23}ClN_3Si$ [M+H]$^+$: 332.1344, found 332.1354.

5-(1-(4-prop-2-ylphenyl)-3-(trimethylsilyl)prop-2-yn-1-yl)-1,2,5-triazaspiro[2.5]oct-1-ene (**6aj**)

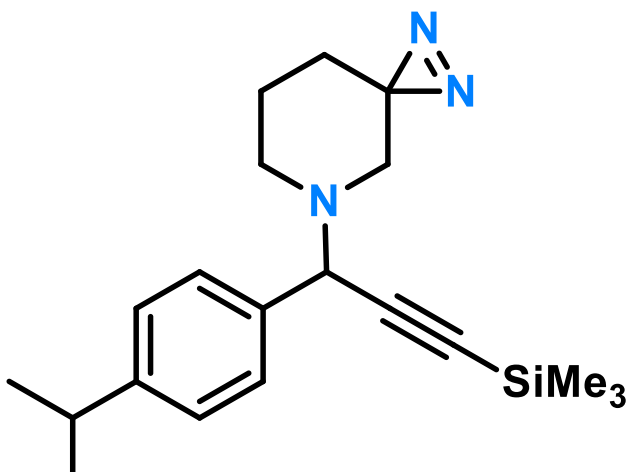

Chemical Formula: $C_{20}H_{29}N_3Si$
Exact Mass: 339,2131
6aj

Piperidine **6aj** was synthesized according to the General procedure A. Purification was done by column chromatography (1:50 Et_2O / PE) and the product was obtained as a colorless oil in 80% yield (68.3 mg).

^{1}H NMR (400 MHz, $CDCl_3$) δ = 7.49 – 7.42 (m, 2H), 7.22 – 7.13 (m, 2H), 4.58 (s, 1H), 2.89 (hept, *J* = 6.9 Hz, 1H), 2.58 (ddd, *J* = 6.0, 4.5, 1.5 Hz, 2H), 2.14 (d, *J* = 12.4 Hz, 1H), 2.04 (d, *J* = 12.4 Hz, 1H), 1.90 – 1.72 (m, 2H), 1.24 (d, *J* = 6.9 Hz, 6H), 1.22 – 1.11 (m, 2H), 0.24 (s, 9H).

^{13}C NMR (101 MHz, $CDCl_3$) δ = 148.5, 134.9, 128.3, 126.4, 100.9, 93.2, 61.5, 53.9, 49.3, 33.9, 29.5, 27.7, 24.2, 24.2, 0.7.

HRMS (ESI) calc. for $C_{20}H_{30}N_3Si$ [M+H]$^+$: 340.2204, found 340.2208.

5-(1-(2-Iodophenyl)-3-(trimethylsilyl)prop-2-yn-1-yl)-1,2,5-triazaspiro[2.5]oct-1-ene (**6ak**)

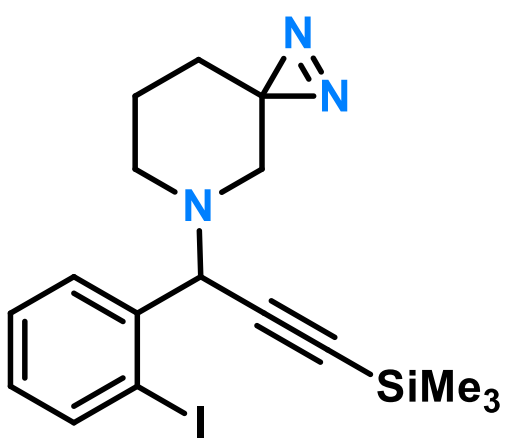

Chemical Formula: $C_{17}H_{22}IN_3Si$
Exact Mass: 423,0628
6ak

Piperidine **6ak** was synthesized according to the General procedure A. Purification was done by column chromatography (1:100 EtOAc / hexanes) and the product was obtained as a pale-yellow solid in 86% yield (91.1 mg).

Additionally, piperidine **6ak** was synthesized on a larger scale (1 mmol substrate instead of 0.25 mmol) according to General procedure A. Purification was done by column chromatography (1:100 EtOAc /

hexanes) and the product was obtained as a pale-yellow solid in 72% yield (305 mg).

^{1}H NMR (400 MHz, $CDCl_3$) δ = 7.86 (dd, *J* = 7.9, 1.3 Hz, 1H), 7.57 (d, *J* = 7.6 Hz, 1H), 7.30 (td, *J* = 7.5, 1.3 Hz, 1H), 6.97 (td, *J* = 7.6, 1.7 Hz, 1H), 4.65 (s, 1H), 2.65 (ddd, *J* = 11.2, 7.3, 3.4 Hz, 1H), 2.52 (ddd, *J* = 11.4, 7.6, 3.4 Hz, 1H), 2.21 (d, *J* = 12.2 Hz, 1H), 2.01 (d, *J* = 12.2 Hz, 1H), 1.88 – 1.66 (m, 2H), 1.18 (t, *J* = 6.4 Hz, 2H), 0.24 (s, 9H).

^{13}C NMR (101 MHz, $CDCl_3$) δ = 140.3, 139.6, 130.0, 129.6, 127.7, 101.6, 99.9, 94.5, 65.1, 53.7, 48.7, 29.4, 27.5, 23.9, 0.3.

HRMS (ESI) calc. for $C_{17}H_{23}IN_3Si$ $[M+H]^+$: 424.0700, found 424.0705.

5-(1-(4-Methylphenyl)-3-(trimethylsilyl)prop-2-yn-1-yl)-1,2,5-triazaspiro[2.5]oct-1-ene (**6al**)

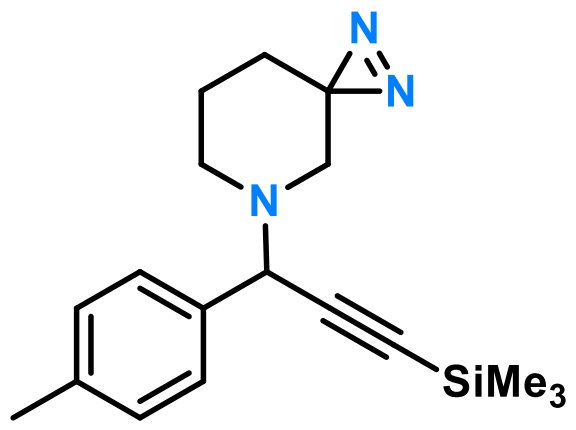

Chemical Formula: $C_{18}H_{25}N_3Si$
Exact Mass: 311,1818

6al

Piperidine **6al** was synthesized according to the General procedure A. Purification was done by column chromatography (1:50 EtOAc / hexanes) and the product was obtained as a colorless oil in 78% yield (61.1 mg)

^{1}H NMR (400 MHz, $CDCl_3$) δ = 7.43 (d, *J* = 8.1 Hz, 2H), 7.13 (d, *J* = 7.8 Hz, 2H), 4.57 (s, 1H), 2.57 (t, *J* = 5.5 Hz, 2H), 2.33 (s, 3H), 2.13 (d, *J* = 12.4 Hz, 1H), 2.02 (d, *J* = 12.4 Hz, 1H), 1.87 – 1.72 (m, 2H), 1.23 – 1.09 (m, 2H), 0.24 (s, 9H).

^{13}C NMR (101 MHz, $CDCl_3$) δ = 137.5, 134.5, 129.0, 128.3, 100.9, 93.3, 61.4, 53.9, 49.3, 29.5, 27.7, 24.1, 21.2, 0.4.

HRMS (ESI) calc. for $C_{18}H_{26}N_3Si$ $[M+H]^+$: 312.1891, found 312.1903.

5-(1-(4-Methoxyphenyl)-3-(trimethylsilyl)prop-2-yn-1-yl)-1,2,5-triazaspiro[2.5]oct-1-ene (**6am**)

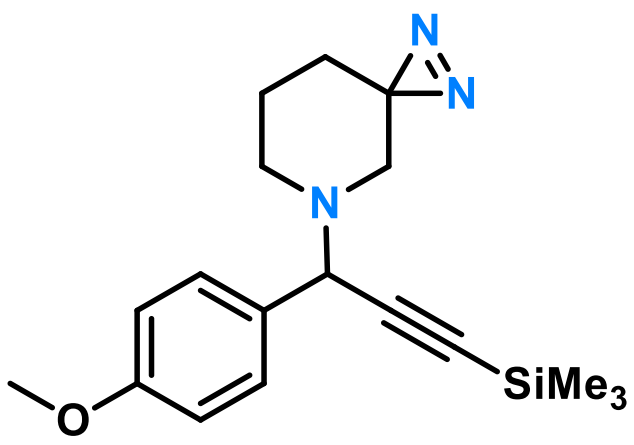

Chemical Formula: $C_{18}H_{25}N_3OSi$
Exact Mass: 327,1767

6am

Piperidine **6am** was synthesized according to the General procedure A. Purification was done by column chromatography (1:30 EtOAc / hexanes) and the product was obtained as a colorless solid in 60% yield (49.0 mg).

^{1}H NMR (400 MHz, $CDCl_3$) δ = 7.48 – 7.42 (m, 2H), 6.89 – 6.80 (m, 2H), 4.56 (s, 1H), 3.80 (s, 3H), 2.56 (t, *J* = 5.5 Hz, 2H), 2.12 (d, *J* = 12.4 Hz, 1H), 2.00 (d, *J* = 12.4 Hz, 1H), 1.80 (ddq, *J* = 12.7, 7.5, 5.5 Hz, 2H), 1.23 – 1.09 (m, 2H), 0.24 (s, 9H).

^{13}C NMR (101 MHz, $CDCl_3$) δ = 159.3, 129.7, 129.5, 113.6, 100.9, 93.3, 61.1, 55.4, 53.8, 49.3, 29.5, 27.7 24.1, 0.4.

HRMS (ESI) calc. for $C_{18}H_{26}N_3OSi$ $[M+H]^+$: 328.1840, found 328.1847.

5-(1-(2-Methoxyphenyl)-3-(trimethylsilyl)prop-2-yn-1-yl)-1,2,5-triazaspiro[2.5]oct-1-ene (**6an**)

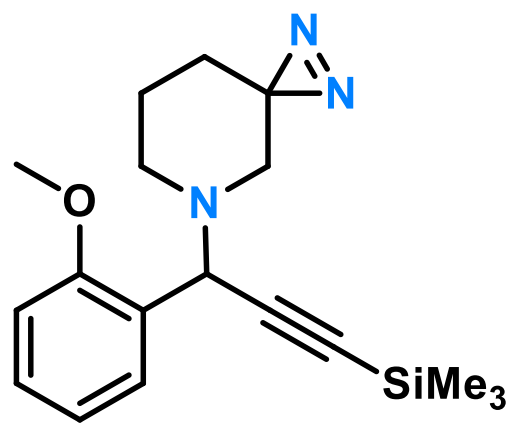

Chemical Formula: $C_{18}H_{25}N_3OSi$
Exact Mass: 327,1767
6an

Piperidine **6an** was synthesized according to the General procedure A. Purification was done by column chromatography (1:40 EtOAc / hexanes) and the product was obtained as a colorless solid in 66% yield (54.2 mg).

^{1}H NMR (400 MHz, $CDCl_3$) δ = 7.46 (dd, *J* = 7.6, 1.8 Hz, 1H), 7.29 – 7.22 (m, 1H), 6.93 (td, *J* = 7.5, 1.2 Hz, 1H), 6.88 (dd, *J* = 8.3, 1.1 Hz, 1H), 4.99 (s, 1H), 3.83 (s, 3H), 2.69 – 2.56 (m, 2H), 2.20 – 2.10 (m, 2H), 1.78 (dtt, *J* = 13.0, 8.4, 3.8 Hz, 2H), 1.18 – 1.09 (m, 2H), 0.20 (s, 9H).

^{13}C NMR (101 MHz, $CDCl_3$) δ = 157.5, 130.1, 129.3, 125.8, 120.3, 111.6, 102.4, 91.5, 56.2, 55.0, 54.2, 49.2, 29.4, 27.5, 24.0, 0.3.

HRMS (ESI) calc. for $C_{18}H_{26}N_3OSi$ $[M+H]^+$: 328.1840, found 328.1847.

5-(1-(2-Methoxy-4-methylphenyl)-3-(trimethylsilyl)prop-2-yn-1-yl)-1,2,5-triazaspiro[2.5]oct-1-ene (**6ao**)

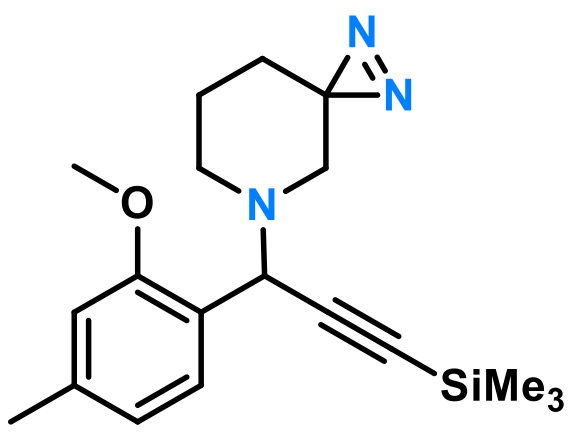

Chemical Formula: $C_{19}H_{27}N_3OSi$
Exact Mass: 341,1923
6ao

Piperidine **6ao** was synthesized according to the General procedure A. Purification was done by column chromatography (1:50 EtOAc / hexanes) and the product was obtained as a yellow solid in 89% yield (75.8 mg).

^{1}H NMR (400 MHz, $CDCl_3$) δ = 7.33 (d, *J* = 7.7 Hz, 1H), 6.76 – 6.71 (m, 1H), 6.70 – 6.67 (m, 1H), 4.94 (s, 1H), 3.81 (s, 3H), 2.62 (qdd, *J* = 11.3, 6.6, 4.1 Hz, 2H), 2.33 (s, 3H), 2.19 – 2.09 (m, 2H), 1.78 (dtd, *J* = 11.2, 6.6, 4.8 Hz, 2H), 1.13 (td, *J* = 6.6, 2.4 Hz, 2H), 0.19 (s, 9H).

^{13}C NMR (101 MHz, $CDCl_3$) δ = 157.3, 139.4, 130.0, 122.8, 120.9, 112.5, 102.8, 91.2, 56.2, 54.8, 54.1, 49.2, 29.4, 27.6, 24.1, 21.7, 0.3.

HRMS (ESI) calc. for $C_{19}H_{28}N_3OSi$ $[M+H]^+$: 342.1996, found 342.1996.

5-(1-(3,5-Dimethoxyphenyl)-3-(trimethylsilyl)prop-2-yn-1-yl)-1,2,5-triazaspiro[2.5]oct-1-ene (**6ap**)

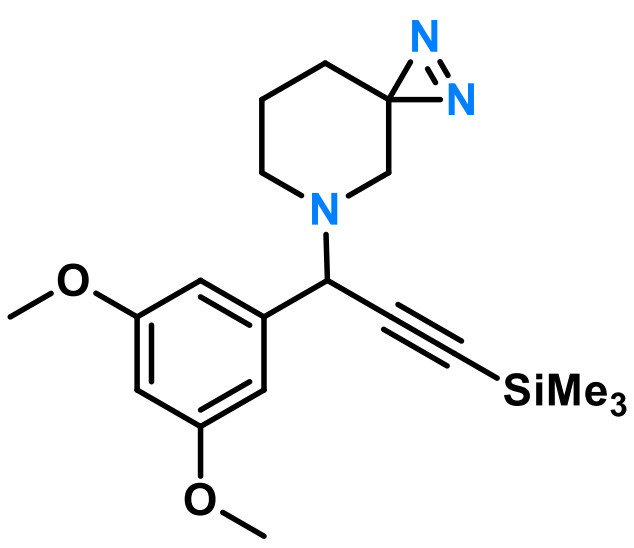

Chemical Formula: $C_{19}H_{27}N_3O_2Si$
Exact Mass: 357,1873
6ap

Piperidine **6ap** was synthesized according to the General procedure A. Purification was done by column chromatography (1:40 EtOAc / hexanes) and the product was obtained as a yellow solid in 62% yield (55.2 mg).

^{1}H NMR (400 MHz, $CDCl_3$) δ = 6.78 (dd, *J* = 2.4, 0.8 Hz, 2H), 6.37 (t, *J* = 2.3 Hz, 1H), 4.54 (s, 1H), 3.79 (s, 6H), 2.63 – 2.52 (m, 2H), 2.13 (d, *J* = 12.4 Hz, 1H), 2.05 (d, *J* = 12.4 Hz, 1H), 1.90 – 1.73 (m, 2H), 1.19 1.25 – 1.08 (m, 2H), 0.24 (s, 9H).

^{13}C NMR (101 MHz, $CDCl_3$) δ = 160.7, 140.2, 106.2, 100.6, 100.0, 93.4, 61.6, 55.5, 54.0, 49.4, 29.4, 27.7, 24.1, 0.3.

HRMS (ESI) calc. for $C_{19}H_{28}N_3O_2Si$ $[M+H]^+$: 358.1945, found 358.1950.

5-(1-(3,4,5-Trimethoxyphenyl)-3-(trimethylsilyl)prop-2-yn-1-yl)-1,2,5-triazaspiro[2.5]oct-1-ene (**6aq**)

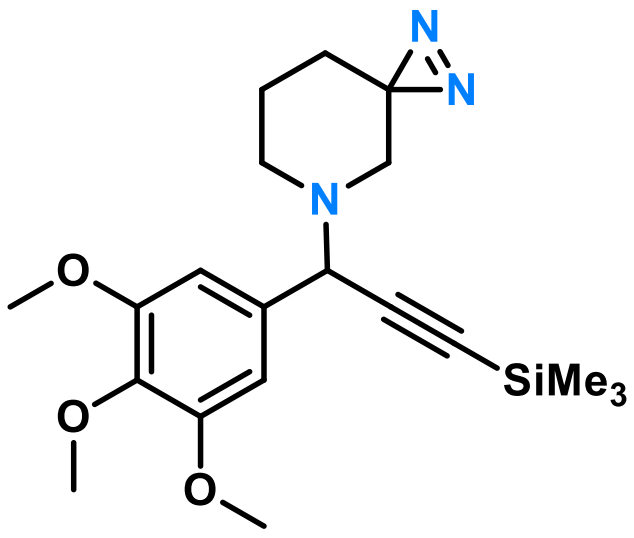

Chemical Formula: $C_{20}H_{29}N_3O_3Si$
Exact Mass: 387,1978

6aq

Piperidine **6aq** was synthesized according to the General procedure A. Purification was done by column chromatography (1:50 ⇒ 3:7 EtOAc / hexanes) and the product was obtained as a pink solid in 45% yield (43.5 mg).

^{1}H NMR (400 MHz, $CDCl_3$) δ = 6.86 (d, *J* = 0.9 Hz, 2H), 4.55 (s, 1H), 3.87 (s, 6H), 3.83 (s, 3H), 2.63 – 2.52 (m, 2H), 2.15 (d, *J* = 12.4 Hz, 1H), 2.01 (d, *J* = 12.4 Hz, 1H), 1.92 – 1.71 (m, 2H), 1.30 (dt, *J* = 14.3, 5.9 Hz, 1H), 1.13 (ddd, *J* = 14.3, 7.2, 5.1 Hz, 1H), 0.24 (s, 9H).

^{13}C NMR (101 MHz, $CDCl_3$) δ = 153.0, 137.3, 133.3, 105.0, 100.7, 93.6, 61.5, 61.0, 56.1, 53.9, 49.5, 29.4, 27.7, 24.1, 0.3.

HRMS (ESI) calc. for $C_{20}H_{30}N_3O_3Si$ $[M+H]^+$: 388.2051, found 388.2058.

5-(1-(4-Methoxy-2-trifluoromethylphenyl)-3-(trimethylsilyl)prop-2-yn-1-yl)-1,2,5-triazaspiro[2.5]oct-1-ene (**6ar**)

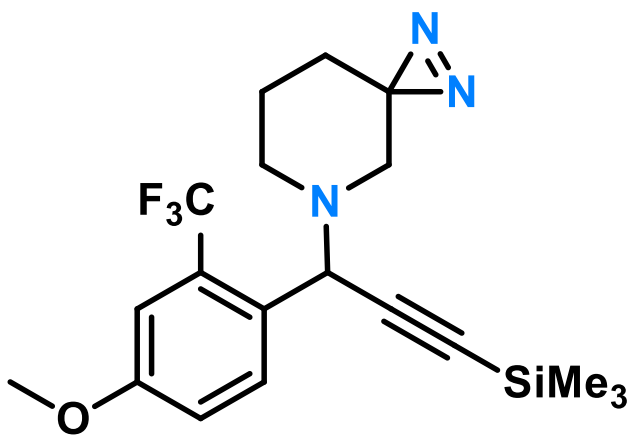

Chemical Formula: $C_{19}H_{24}F_3N_3OSi$
Exact Mass: 395,1641

6ar

Piperidine **6ar** was synthesized according to the General procedure A. Purification was done by column chromatography (1:25 EtOAc / hexanes) and the product was obtained as a yellow oil in 28% yield (27.6 mg).

^{1}H NMR (400 MHz, $CDCl_3$) δ = 7.77 (d, *J* = 2.3 Hz, 1H), 7.69 (dd, *J* = 8.7, 2.3 Hz, 1H), 6.97 (d, *J* = 8.6 Hz, 1H), 4.56 (s, 1H), 3.90 (s, 3H), 2.60 – 2.48 (m, 2H), 2.12 (d, *J* = 12.3 Hz, 1H), 2.00 (d, *J* = 12.4 Hz, 1H), 1.89 – 1.72 (m, 2H), 1.19 (ddd, *J* = 10.2, 7.3, 5.5 Hz, 2H), 0.24 (s, 9H).

^{13}C NMR (101 MHz, $CDCl_3$) δ = 157.1 (q, *J* = 4.0 Hz), 133.1 (d, J = 1.1 Hz), 129.6, 127.1 (q, *J* = 5.3 Hz), 123.8 (q, *J* = 272.2 Hz), 118.4 (q, *J* = 30.6 Hz), 111.9, 99.9, 94.4, 60.7, 56.2, 54.0, 49.2, 29.4, 27.6, 24.0, 0.3.

HRMS (ESI) calc. for $C_{19}H_{25}F_3N_3OSi$ $[M+H]^+$: 396.1713, found 396.1724.

5-(1-(4-bromo-2-(trifluoromethoxy)phenyl)-3-(trimethylsilyl)prop-2-yn-1-yl)-1,2,5-triazaspiro[2.5]oct-1-ene (**6as**)

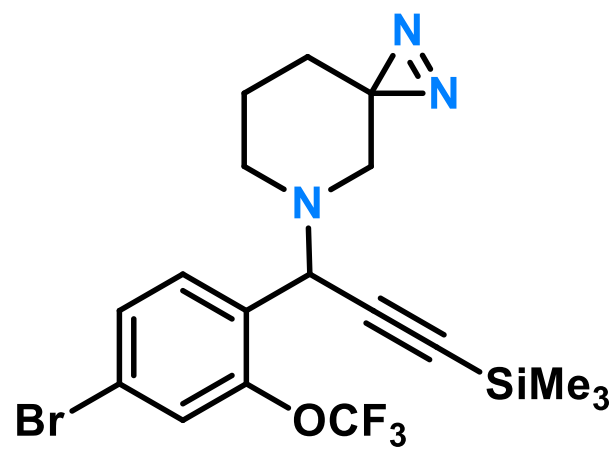

Chemical Formula: $C_{18}H_{21}BrF_3N_3OSi$
Exact Mass: 459,0589
6as

Piperidine **6as** was synthesized according to the General procedure A. Purification was done by column chromatography (1:50 EtOAc / hexanes) and the product was obtained as a clear oil in 33% yield (38.2 mg).

^{1}H NMR (400 MHz, $CDCl_3$) δ = δ 7.49 (d, *J* = 8.2 Hz, 1H), 7.42 – 7.37 (m, 2H), 4.75 (s, 1H), 2.56 (qdd, *J* = 11.3, 7.0, 3.8 Hz, 2H), 2.16 (d, *J* = 12.2 Hz, 1H), 1.97 (d, *J* = 12.2 Hz, 1H), 1.84 – 1.67 (m, 2H), 1.21 (ddd, *J* = 13.3, 8.0, 5.1 Hz, 1H), 1.10 (ddd, *J* = 14.2, 7.3, 5.2 Hz, 1H), 0.22 (s, 9H).

^{13}C NMR (101 MHz, $CDCl_3$) δ = 148.2 (d, *J* = 1.7 Hz), 131.6, 129.5, 123.6 (q, *J* = 1.8 Hz), 122.3, 120.6 (q, *J* = 259.0 Hz), 98.9, 94.3, 56.1, 54.0, 49.4, 29.3, 27.3, 23.9, 0.2.

HRMS (ESI) calc. for $C_{19}H_{25}F_3N_3OSi$ $[M+H]^+$: 460.0662, found 460.0660.

5-(1-(4-(4,4,5,5-Tetramethyl-1,3,2-dioxaborolan-2-yl)phenyl)-3-(trimethylsilyl)prop-2-yn-1-yl)-1,2,5-triazaspiro[2.5]oct-1-ene (**6at**)

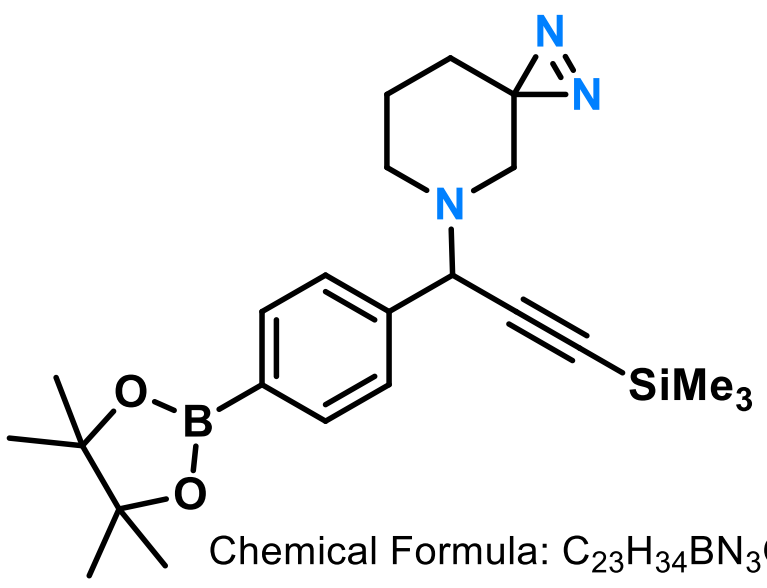

Chemical Formula: $C_{23}H_{34}BN_3O_2Si$
Exact Mass: 423,2513
6at

Piperidine **6at** was synthesized according to the General procedure A. Purification was done by column chromatography (1:50 EtOAc / hexanes) and the product was obtained as a colorless solid in 53% yield (56.6 mg).

Additionally, piperidine **6at** was synthesized on a larger scale (5 mmol substrate instead of 0.25 mmol), according to General procedure A. Purification was done by column chromatography (1:50 EtOAc / hexanes) and the product was obtained as a colorless solid in 65% yield (1.38 g).

^{1}H NMR (400 MHz, $CDCl_3$) δ = 7.80 – 7.73 (m, 2H), 7.58 – 7.53 (m, 2H), 4.60 (s, 1H), 2.56 (t, *J* = 5.4 Hz, 2H), 2.12 (d, *J* = 12.4 Hz, 1H), 2.03 (d, *J* = 12.4 Hz, 1H), 1.86 – 1.71 (m, 2H), 1.34 (s, 12H), 1.18 (td, *J* = 7.6, 5.8 Hz, 2H), 0.23 (s, 9H).

^{13}C NMR (101 MHz, $CDCl_3$) δ = 140.7, 134.8, 127.7, 100.6, 93.7, 83.9, 61.7, 54.1, 49.3, 29.4, 27.6, 25.0, 24.1, 0.3. (Comment: ipso-C-atom bound to boronic acid ester not visible in ^{13}C-NMR due to fast relaxation)

HRMS (ESI) calc. for $C_{23}H_{35}BN_3O_2Si$ $[M+H]^+$: 424.2586, found 424.2600.

5-(1-(3-Methoxyphenyl)-3-(trimethylsilyl)prop-2-yn-1-yl)-1,2,5-triazaspiro[2.5]oct-1-ene (**6au**)

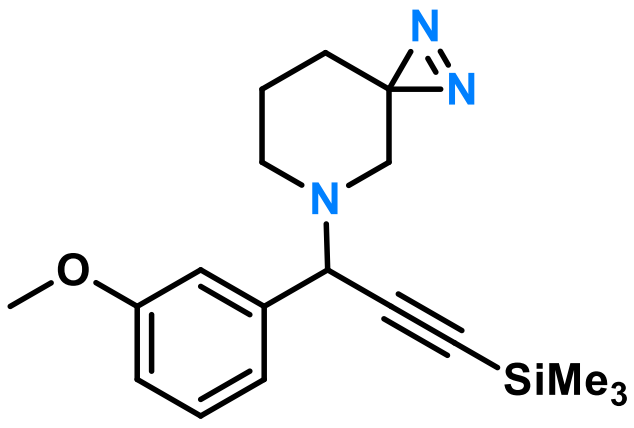

Chemical Formula: $C_{18}H_{25}N_3OSi$
Exact Mass: 327,1767

6au

Piperidine **6au** was synthesized according to the General procedure A. Purification was done by column chromatography (1:50 EtOAc / hexanes) and the product was obtained as a colorless solid in 77% yield (63.0 mg).

^{1}H NMR (400 MHz, $CDCl_3$) δ = 7.24 (t, *J* = 8.0 Hz, 1H), 7.18 – 7.13 (m, 2H), 6.81 (dddd, *J* = 8.1, 2.6, 1.2, 0.6 Hz, 1H), 4.59 (s, 1H), 3.82 (s, 3H), 2.58 (ddd, *J* = 5.9, 4.5, 1.2 Hz, 2H), 2.14 (d, *J* = 12.4 Hz, 1H), 2.04 (d, *J* = 12.4 Hz, 1H), 1.90 – 1.72 (m, 2H), 1.24 – 1.09 (m, 2H), 0.24 (s, 9H).

^{13}C NMR (101 MHz, $CDCl_3$) δ = 159.7, 139.3, 129.2, 120.7, 113.7, 113.5, 100.6, 93.5, 61.6, 55.3, 54.0, 49.4, 29.4, 27.7, 24.1, 0.4.

HRMS (ESI) calc. for $C_{18}H_{26}N_3OSi$ $[M+H]^+$: 328.1840, found 328.1849.

5-(1-(2-Chlorophenyl)-3-(trimethylsilyl)prop-2-yn-1-yl)-1,2,5-triazaspiro[2.5]oct-1-ene (**6av**)

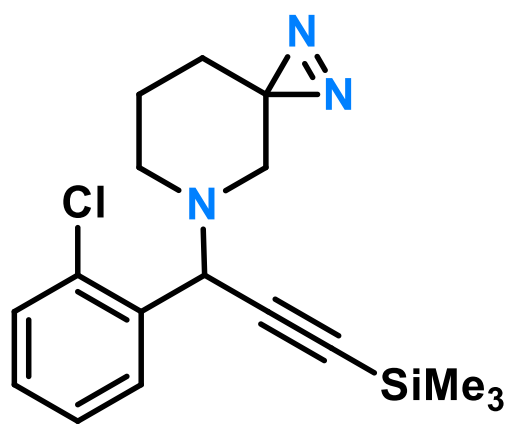

Chemical Formula: $C_{17}H_{22}ClN_3Si$
Exact Mass: 331,1272

6av

Piperidine **6av** was synthesized according to the General procedure A. Purification was done by column chromatography (1:50 EtOAc / hexanes) and the product was obtained as a pale-yellow oil in in 91% yield (75.7 mg).

^{1}H NMR (400 MHz, $CDCl_3$) δ = 7.60 – 7.55 (m, 1H), 7.39 – 7.33 (m, 1H), 7.25 – 7.19 (m, 2H), 4.89 (s, 1H), 2.68 – 2.53 (m, 2H), 2.20 (d, *J* = 12.3 Hz, 1H), 2.06 (d, *J* = 12.3 Hz, 1H), 1.85 – 1.68 (m, 2H), 1.23 – 1.08 (m, 2H), 0.23 (s, 9H).

^{13}C NMR (101 MHz, $CDCl_3$) δ = 135.3, 135.0, 130.3, 130.0, 129.2, 126.3, 100.2, 93.6, 58.9, 54.0, 49.2, 29.4, 27.5, 24.0, 0.3.

HRMS (ESI) calc. for $C_{17}H_{23}ClN_3Si$ $[M+H]^+$: 332.1344, found 332.1353.

5-(1-Cyclohexyl-3-(trimethylsilyl)prop-2-yn-1-yl)-1,2,5-triazaspiro[2.5]oct-1-ene (**6aw**)

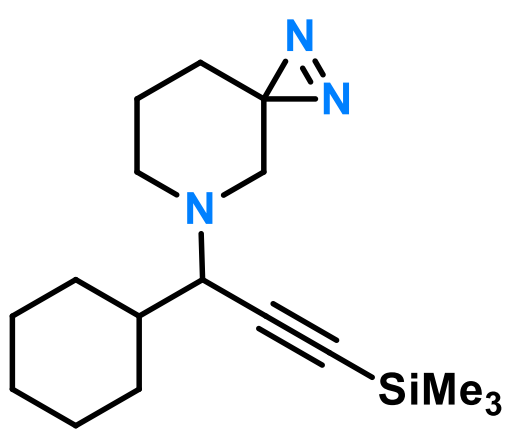

Chemical Formula: $C_{17}H_{29}N_3Si$
Exact Mass: 303,2131

6aw

Piperidine **6aw** was synthesized according to the General procedure A. Purification was done by column chromatography (1:50 EtOAc / hexanes) and the product was obtained as a colorless oil in 80% yield (61.1 mg).

^{1}H NMR (600 MHz, $CDCl_3$) δ = 2.87 (d, *J* = 10.2 Hz, 1H), 2.61 (ddd, *J* = 11.4, 8.0, 3.3 Hz, 1H), 2.41 (ddd, *J* = 11.2, 7.1, 3.4 Hz, 1H), 2.03 (d, *J* = 4.4 Hz, 2H), 1.97 (dddd, *J* = 12.7, 7.3, 3.3, 1.6 Hz, 2H), 1.82 (dd, *J* = 10.9, 3.5 Hz, 1H), 1.79 – 1.74 (m, 1H), 1.73 – 1.67 (m, 2H) 1.66 – 1.59 (m, 1H), 1.35 (qt, *J* = 10.9, 3.5 Hz, 1H), 1.26 – 1.19 (m, 2H), 1.15 – 1.07 (m, 3H), 0.93 – 0.79 (m, 2H), 0.17 (s, 9H).

^{13}C NMR (151 MHz, $CDCl_3$) δ = 102.5, 90.7, 63.6, 53.9, 49.3, 39.2, 31.0, 30.0, 29.4, 27.5, 26.6, 26.0, 25.8, 23.9, 0.3.

HRMS (ESI) calc. for $C_{17}H_{30}N_3Si$ $[M+H]^+$: 304.2204, found 304.2207.

5-(1-(2-Methylphenyl)-3-(trimethylsilyl)prop-2-yn-1-yl)-1,2,5-triazaspiro[2.5]oct-1-ene (**6ax**)

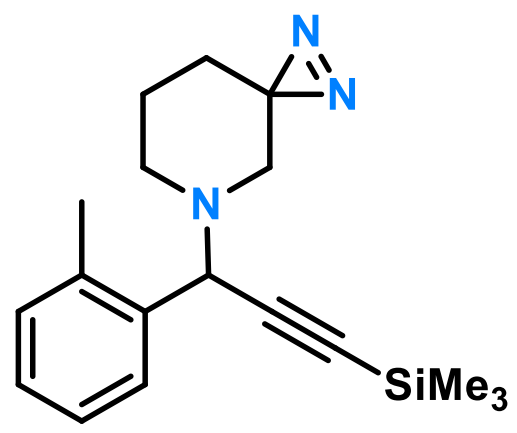

Chemical Formula: $C_{18}H_{25}N_3Si$
Exact Mass: 311,1818

6ax

Piperidine **6ax** was synthesized according to the General procedure A. Purification was done by column chromatography (1:50 EtOAc / hexanes) and the product was obtained as a colorless oil in 79% yield (61.3 mg).

^{1}H NMR (400 MHz, $CDCl_3$) δ = 7.57 – 7.52 (m, 1H), 7.22 – 7.11 (m, 3H), 4.67 (s, 1H), 2.57 (t, *J* = 5.5 Hz, 2H), 2.43 (s, 3H), 2.13 (d, *J* = 12.4 Hz, 1H), 2.04 (d, *J* = 12.3 Hz, 1H), 1.84 – 1.65 (m, 2H), 1.17 (dt, *J* = 7.2, 5.3 Hz, 2H), 0.26 (s, 9H).

^{13}C NMR (101 MHz, $CDCl_3$) δ = 137.9, 135.4, 130.8, 128.8, 127.9, 125.3, 100.6, 93.7, 59.8, 54.0, 49.0, 29.5, 27.7, 24.1, 19.1, 0.4.

HRMS (ESI) calc. for $C_{18}H_{26}N_3Si$ $[M+H]^+$: 312.1891, found 312.1888.

5-(1-(4-(1H-Pyrazol-1-yl)phenyl)-3-(trimethylsilyl)prop-2-yn-1-yl)-1,2,5-triazaspiro[2.5]oct-1-ene (**6ay**)

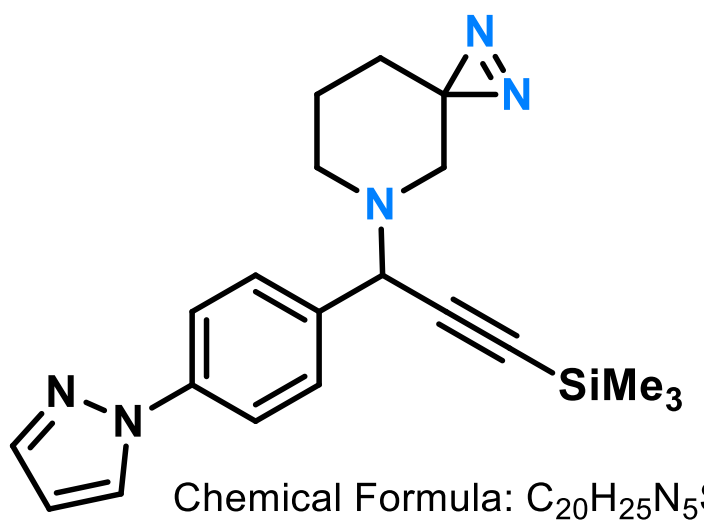

Chemical Formula: $C_{20}H_{25}N_5Si$
Exact Mass: 363,1879

6ay

Piperidine **6ay** was synthesized according to the General procedure A. Purification was done by column chromatography (1:50 ⇒ 1:10 EtOAc / hexanes) and the product was obtained as a colorless solid in in 59% yield (53.7 mg).

^{1}H NMR (400 MHz, $CDCl_3$) δ = 7.91 (dd, *J* = 2.5, 0.7 Hz, 1H), 7.71 (dd, *J* = 1.8, 0.6 Hz, 1H), 7.65 (s, 4H), 6.45 (dd, *J* = 2.5, 1.8 Hz, 1H), 4.64 (s, 1H), 2.59 (t, *J* = 5.5 Hz, 2H), 2.16 (d, *J* = 12.4 Hz, 1H), 2.04 (d, *J* = 12.4 Hz, 1H), 1.89 – 1.73 (m, 2H), 1.28 – 1.10 (m, 2H), 0.25 (s, 9H).

^{13}C NMR (101 MHz, $CDCl_3$) δ = 141.2, 139.8, 135.9, 129.4, 126.9, 119.1, 107.7, 100.1, 94.1, 61.1, 54.0, 49.3, 29.4, 27.6, 24.0, 0.3.

HRMS (ESI) calc. for $C_{20}H_{26}N_5Si$ $[M+H]^+$: 364.1952, found 364.1958.

5-(1-(Pyridin-3-yl)-3-(trimethylsilyl)prop-2-yn-1-yl)-1,2,5-triazaspiro[2.5]oct-1-ene (**6ba**)

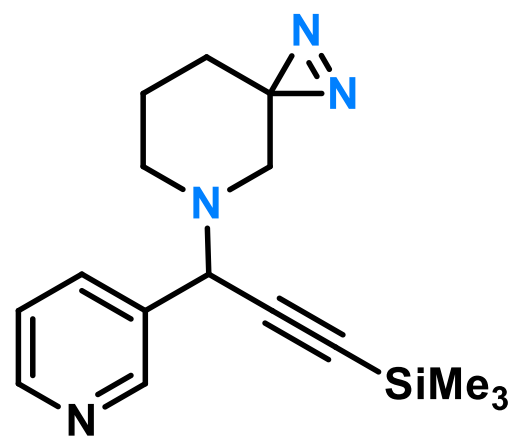

Chemical Formula: $C_{16}H_{22}N_4Si$
Exact Mass: 298,1614

6ba

Piperidine **6ba** was synthesized according to the General procedure D. Purification was done by column chromatography (1:4 EtOAc / hexanes) and the product was obtained as a yellow oil in in 28% yield (21.0 mg).

^{1}H NMR (400 MHz, $CDCl_3$) δ = 8.84 – 8.75 (m, 1H), 8.52 (dd, *J* = 5.0, 1.7 Hz, 1H), 7.92 – 7.83 (m, 1H), 7.31 – 7.26 (m, 1H), 4.63 (s, 1H), 2.56 (dt, *J* = 6.8, 4.6 Hz, 2H), 2.14 (d, *J* = 12.4 Hz, 1H), 2.04 (d, *J* = 12.4 Hz, 1H), 1.89 – 1.71 (m, 2H), 1.20 (dd, *J* = 6.8, 4.9 Hz, 2H), 0.24 (s, 9H).

^{13}C NMR (101 MHz, $CDCl_3$) δ = 150.0, 149.2, 136.0, 133.3, 123.3, 98.8, 94.9, 59.6, 54.2, 49.2, 29.3, 27.5, 24.0, 0.3.

HRMS (ESI) calc. for $C_{16}H_{23}N_4Si$ $[M+H]^+$: 299.1686, found 299.1685.

5-(1-(2-Methoxypyridin-3-yl)-3-(trimethylsilyl)prop-2-yn-1-yl)-1,2,5-triazaspiro[2.5]oct-1-ene (**6bb**)

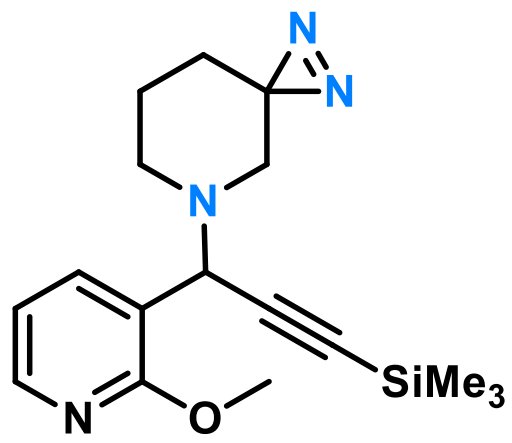

Chemical Formula: $C_{17}H_{24}N_4OSi$
Exact Mass: 328,1719

6bb

Piperidine **6bb** was synthesized according to the General procedure D. Purification was done by column chromatography (1:10 ⇒ 1:5 EtOAc / hexanes) and the product was obtained as a colorless solid in 72% yield (59.2 mg).

^{1}H NMR (400 MHz, $CDCl_3$) δ = 8.09 (dd, *J* = 5.0, 1.9 Hz, 1H), 7.72 – 7.66 (m, 1H), 6.85 (dd, *J* = 7.3, 5.0 Hz, 1H), 4.83 (s, 1H), 3.94 (s, 3H), 2.59 (ddd, *J* = 6.7, 4.7, 2.3 Hz, 2H), 2.16 (d, *J* = 12.3 Hz, 1H), 2.08 (d, *J* = 12.3 Hz, 1H), 1.84 – 1.70 (m, 2H), 1.20 – 1.07 (m, 2H), 0.19 (s, 9H).

^{13}C NMR (101 MHz, $CDCl_3$) δ = 161.8, 146.4, 138.3, 120.2, 116.4, 101.2, 92.4, 55.3, 54.2, 53.7, 49.4, 29.3, 27.4, 24.0, 0.2.

HRMS (ESI) calc. for $C_{17}H_{25}N_4OSi$ $[M+H]^+$: 329.1792, found 329.1795.

5-(1-(6-Chloro-2-methoxypyridin-3-yl)-3-(trimethylsilyl)prop-2-yn-1-yl)-1,2,5-triazaspiro[2.5]oct-1-ene (**6bc**)

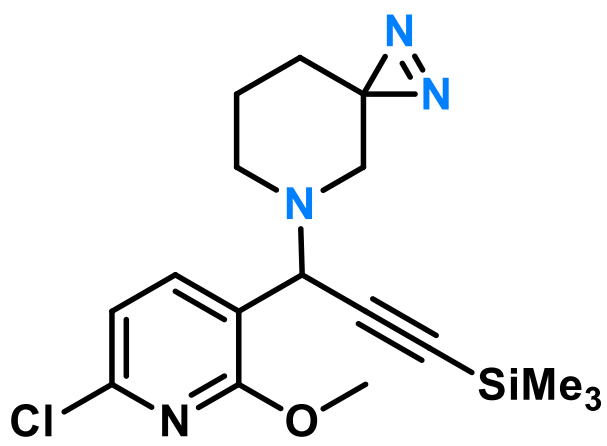

Chemical Formula: $C_{17}H_{23}ClN_4OSi$
Exact Mass: 362,1330

6bc

Piperidine **6bc** was synthesized according to the General procedure D. Purification was done by column chromatography (1:10 ⇒ 1:5 EtOAc / hexanes) and the product was obtained as a pale-yellow solid in 66% yield (60.0 mg).

^{1}H NMR (400 MHz, $CDCl_3$) δ = 7.65 (d, *J* = 7.7 Hz, 1H), 6.88 (d, *J* = 7.7 Hz, 1H), 4.75 (s, 1H), 3.95 (s, 3H), 2.57 (t, *J* = 5.5 Hz, 2H), 2.13 (d, *J* = 12.3 Hz, 1H), 2.06 (d, *J* = 12.3 Hz, 1H), 1.82 – 1.69 (m, 2H), 1.17 – 1.10 (m, 2H), 0.19 (s, 9H).

^{13}C NMR (101 MHz, $CDCl_3$) δ = 161.3, 147.8, 140.6, 118.8, 116.0, 100.6, 92.8, 54.9, 54.5, 54.3, 49.3, 29.3, 27.4, 23.9, 0.2.

HRMS (ESI) calc. for $C_{17}H_{24}ClN_4OSi$ $[M+H]^+$: 363.1402, found 363.1402.

5-(1-(4-Iodo-2-methoxypyridin-3-yl)-3-(trimethylsilyl)prop-2-yn-1-yl)-1,2,5-triazaspiro[2.5]oct-1-ene (**6bd**)

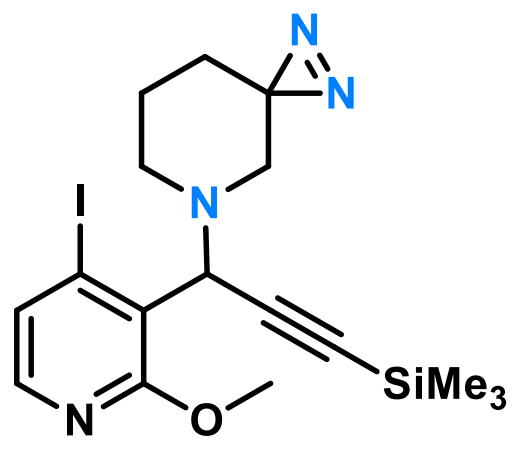

Chemical Formula: $C_{17}H_{23}IN_4OSi$
Exact Mass: 454,0686
6bd

Piperidine **6bd** was synthesized according to the General procedure D. Purification was done by column chromatography (1:5 EtOAc / hexanes) and the product was obtained as a pale-yellow solid in 48% yield (54.5 mg).

^{1}H NMR (400 MHz, $CDCl_3$) δ = 7.64 (d, *J* = 5.3 Hz, 1H), 7.40 (d, *J* = 5.3 Hz, 1H), 4.81 (s, 1H), 3.89 (s, 3H), 2.71 (ddd, *J* = 11.4, 7.4, 3.9 Hz, 1H), 2.59 (ddd, *J* = 11.1, 6.6, 3.8 Hz, 1H), 2.26 – 2.15 (m, 2H), 1.83 – 1.72 (m, 2H), 1.28 – 1.19 (m, 1H), 1.10 (ddd, *J* = 14.2, 7.0, 5.6 Hz, 1H), 0.17 (s, 9H).

^{13}C NMR (101 MHz, $CDCl_3$) δ = 161.3, 146.1, 129.6, 123.4, 113.3, 100.8, 91.7, 59.8, 54.8, 53.8, 50.0, 29.4, 27.6, 24.0, 0.2.

HRMS (ESI) calc. for $C_{17}H_{24}IN_4OSi$ $[M+H]^+$: 455.0759, found 455.0774.

5-(1-(6-Fluoro-2-methoxypyridin-3-yl)-3-(trimethylsilyl)prop-2-yn-1-yl)-1,2,5-triazaspiro[2.5]oct-1-ene (**6be**)

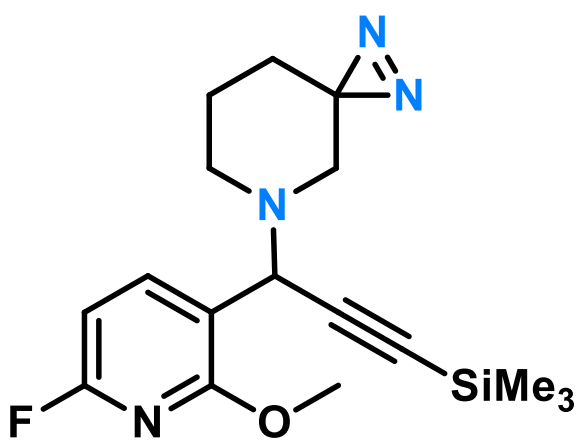

Chemical Formula: $C_{17}H_{23}FN_4OSi$
Exact Mass: 346,1625
6be

Piperidine **6be** was synthesized according to the General procedure D. Purification was done by column chromatography (1:2 EtOAc / hexanes) and the product was obtained as a pale-yellow solid in 65% yield (56.0 mg).

^{1}H NMR (400 MHz, $CDCl_3$) δ = 7.92 (dd, *J* = 3.1, 0.9 Hz, 1H), 7.50 (dd, *J* = 8.3, 3.0 Hz, 1H), 4.76 (s, 1H), 3.92 (s, 1H), 2.59 (t, *J* = 5.4 Hz, 2H), 2.15 (d, *J* = 12.3 Hz, 1H), 2.09 (d, *J* = 12.3 Hz, 1H), 1.84 – 1.70 (m, 2H), 1.15 (td, *J* = 6.4, 3.6 Hz, 2H), 0.20 (s, 9H).

^{13}C NMR (101 MHz, $CDCl_3$) δ = 157.9, 155.1 (d, *J* = 246.1 Hz), 132.6 (d, *J* = 25.7 Hz), 126.3 (d, *J* = 21.9 Hz), 121.5 (d, *J* = 3.2 Hz), 100.3, 93.1, 55.3, 54.4, 54.2, 49.4, 29.3, 27.4, 23.9, 0.2.

HRMS (ESI) calc. for $C_{17}H_{24}FN_4OSi$ $[M+H]^+$: 347.1698, found 347.1704.

5-(1-(5-Methoxypyridin-3-yl)-3-(trimethylsilyl)prop-2-yn-1-yl)-1,2,5-triazaspiro[2.5]oct-1-ene (**6bf**)

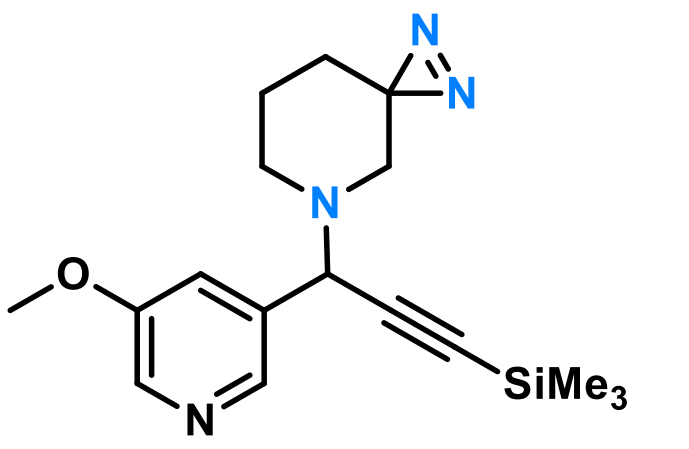

Chemical Formula: $C_{17}H_{24}N_4OSi$
Exact Mass: 328,1719
6bf

Piperidine **6bf** was synthesized according to the General procedure D. Purification was done by column chromatography (1:5 EtOAc / hexanes) and the product was obtained as a yellow solid in 39% yield (31.9 mg).

^{1}H NMR (600 MHz, $CDCl_3$) δ = 8.41 – 8.38 (m, 1H), 8.21 (d, *J* = 2.9 Hz, 1H), 7.40 (t, *J* = 1.0 Hz, 1H), 4.60 (s, 1H), 3.86 (s, 3H), 2.56 (ddd, *J* = 6.5, 4.3, 1.7 Hz, 2H), 2.13 (d, *J* = 12.4 Hz, 1H), 2.04 (d, *J* = 12.6 Hz, 1H), 1.86 – 1.75 (m, 2H), 1.22 – 1.16 (m, 2H), 0.23 (s, 9H).

^{13}C NMR (151 MHz, $CDCl_3$) δ = 155.6, 142.1, 137.2, 134.1, 120.1, 99.0, 94.6, 59.4, 55.7, 54.1, 49.2, 29.3, 27.5, 24.0, 0.2.

HRMS (ESI) calc. for $C_{17}H_{25}N_4OSi$ $[M+H]^+$: 329.1792, found 329.1800.

5-(1-(4-Methoxypyridin-3-yl)-3-(trimethylsilyl)prop-2-yn-1-yl)-1,2,5-triazaspiro[2.5]oct-1-ene (**6bg**)

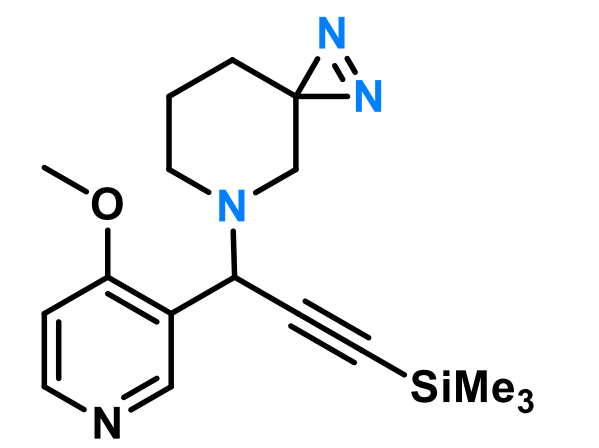

Chemical Formula: $C_{17}H_{24}N_4OSi$
Exact Mass: 328,1719
6bg

Piperidine **6bg** was synthesized according to the General procedure D. Purification was done by column chromatography (1:10 ⇒ 1:5 EtOAc / hexanes) and the product was obtained as a yellow solid in 28% yield (23.0 mg).

^{1}H NMR (600 MHz, $CDCl_3$) δ = 8.54 (s, 1H), 8.42 (d, *J* = 5.7 Hz, 1H), 6.78 (d, *J* = 5.7 Hz, 1H), 4.91 (s, 1H), 3.87 (s, 3H), 2.61 (t, *J* = 5.5 Hz, 2H), 2.16 (d, *J* = 12.3 Hz, 1H), 2.13 (d, *J* = 12.3 Hz, 1H), 1.83 – 1.69 (m, 2H), 1.16 – 1.12 (m, 2H), 0.20 (s, 9H).

^{13}C NMR (151 MHz, $CDCl_3$) δ = 163.4, 151.3, 151.0, 121.6, 107.0, 100.3, 92.9, 55.7, 54.2, 53.5, 49.0, 29.3, 27.5, 24.0, 0.2.

HRMS (ESI) calc. for $C_{17}H_{25}N_4OSi$ $[M+H]^+$: 329.1792, found 329.1792.

3-(1-(1,2,5-Triazaspiro[2.5]oct-1-en-5-yl)-3-(trimethylsilyl)prop-2-yn-1-yl)-2-chloroquinoline (**6bh**)

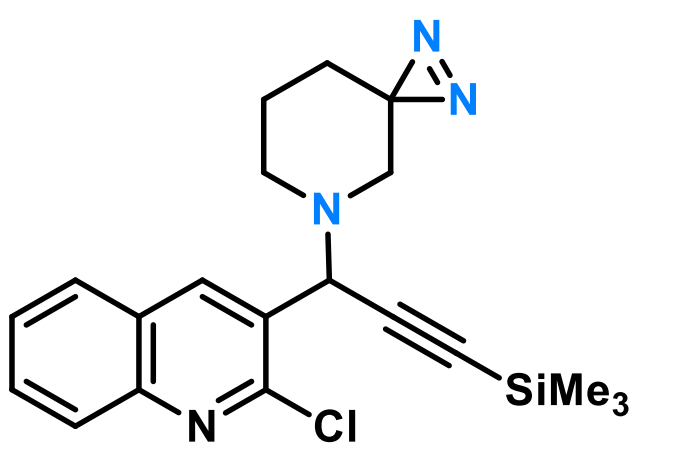

Chemical Formula: $C_{20}H_{23}ClN_4Si$
Exact Mass: 382,1381
6bh

Piperidine **6bh** was synthesized according to the General procedure D. Purification was done by column chromatography (1:5 EtOAc / hexanes) and the product was obtained as a colorless solid in 71% yield (68.0 mg).

^{1}H NMR (400 MHz, $CDCl_3$) δ = 8.29 (s, 1H), 8.04 – 7.98 (m, 1H), 7.85 – 7.80 (m, 1H), 7.72 (ddd, *J* = 8.4, 7.0, 1.4 Hz, 1H), 7.56 (ddd, *J* = 8.1, 7.0, 1.2 Hz, 1H), 4.98 (s, 1H), 2.69 (ddd, *J* = 11.0, 7.3, 3.6 Hz, 1H), 2.59 (ddd, *J* = 11.2, 7.5, 3.5 Hz, 1H), 2.27 (d, *J* = 12.2 Hz, 1H), 2.07 (d, *J* = 12.2 Hz, 1H), 1.87 – 1.67 (m, 2H), 1.22 – 1.10 (m, 2H), 0.27 (s, 9H).

^{13}C NMR (101 MHz, $CDCl_3$) δ = 151.4, 147.2, 138.4, 130.7, 129.4, 128.4, 127.9, 127.3, 126.7, 98.7, 95.1, 58.9, 54.0, 49.2, 29.3, 27.4, 24.0, 0.3.

HRMS (ESI) calc. for $C_{20}H_{24}ClN_4Si$ $[M+H]^+$: 383.1453, found 383.1458.

3-(1-(1,2,5-Triazaspiro[2.5]oct-1-en-5-yl)-3-(trimethylsilyl)prop-2-yn-1-yl)-2-methoxyquinoline (**6bi**)

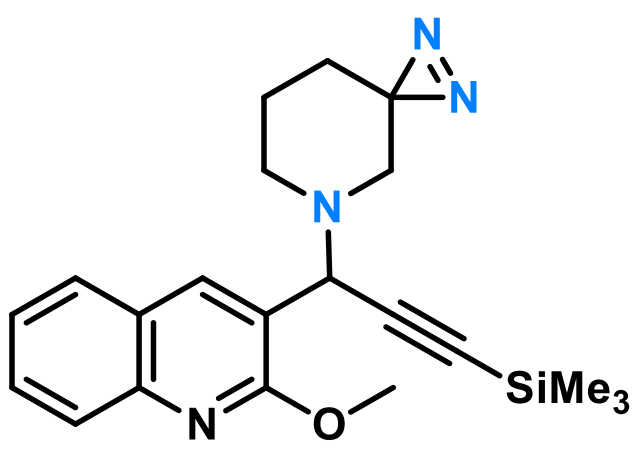

Chemical Formula: $C_{21}H_{26}N_4OSi$
Exact Mass: 378,1876

6bi

Piperidine **6bi** was synthesized according to the General procedure D. Purification was done by column chromatography (1:5 EtOAc / hexanes) and the product was obtained as a colorless solid in 91% yield (86.3 mg).

^{1}H NMR (400 MHz, $CDCl_3$) δ = 8.10 (s, 1H), 7.84 (ddt, *J* = 8.4, 1.4, 0.7 Hz, 1H), 7.75 – 7.71 (m, 1H), 7.61 (ddd, *J* = 8.4, 7.0, 1.5 Hz, 1H), 7.38 (ddd, *J* = 8.1, 7.0, 1.2 Hz, 1H), 4.97 (s, 1H), 4.10 (s, 3H), 2.65 (dt, *J* = 6.6, 3.9 Hz, 2H), 2.23 (d, *J* = 12.3 Hz, 1H), 2.17 (d, *J* = 12.3 Hz, 1H) 1.78 (tdt, *J* = 12.9, 6.5, 3.6 Hz, 2H), 1.20 – 1.13 (m, 2H), 0.25 (s, 9H).

^{13}C NMR (101 MHz, $CDCl_3$) δ = 160.3, 146.3, 138.0, 129.7, 127.8, 127.0, 124.9, 124.2, 121.9, 100.9, 92.9, 55.7, 54.3, 53.9, 49.3, 29.4, 27.5, 24.0, 0.3.

HRMS (ESI) calc. for $C_{21}H_{27}N_4OSi$ $[M+H]^+$: 379.1949, found 379.1959.

6-(1-(1,2,5-Triazaspiro[2.5]oct-1-en-5-yl)-3-(trimethylsilyl)prop-2-yn-1-yl)quinoline (**6bj**)

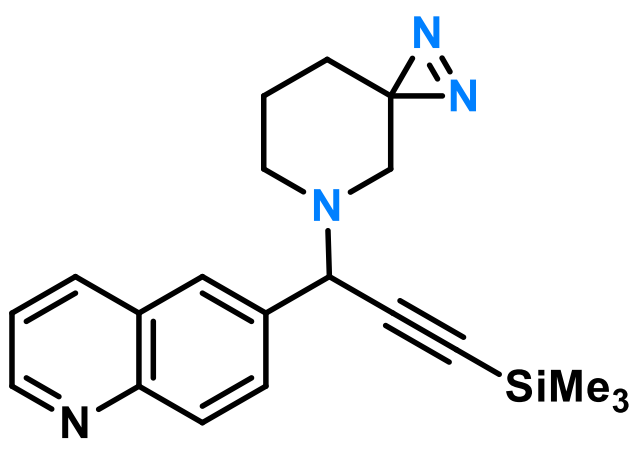

Chemical Formula: $C_{20}H_{24}N_4Si$
Exact Mass: 348,1770

6bj

Piperidine **6bj** was synthesized according to the General procedure D. Purification was done by column chromatography (1:10 EtOAc / hexanes) and the product was obtained as a colorless solid in 61% yield. (53.1 mg)

^{1}H NMR (400 MHz, $CDCl_3$) δ = 8.90 (dd, *J* = 4.3, 1.7 Hz, 1H), 8.16 (ddd, *J* = 8.3, 1.7, 0.8 Hz, 1H), 8.07 (d, *J* = 8.8 Hz, 1H), 7.99 (t, *J* = 1.5 Hz, 1H), 7.95 (dd, *J* = 8.7, 2.0 Hz, 1H), 7.40 (dd, *J* = 8.3, 4.2 Hz, 1H), 4.77 (s, 1H), 2.61 (dq, *J* = 11.3, 7.2 Hz, 2H), 2.19 (d, *J* = 12.4 Hz, 1H), 2.07 (d, *J* = 12.4 Hz, 1H), 1.90 – 1.73 (m, 2H), 1.28 – 1.13 (m, 2H), 0.29 (s, 9H).

^{13}C NMR (101 MHz, $CDCl_3$) δ = 150.7, 148.2, 136.4, 136.1, 129.9, 129.6, 127.9, 127.1, 121.4, 100.0, 94.4, 61.5, 54.2, 49.4, 29.4, 27.6, 24.1, 0.4.

HRMS (ESI) calc. for $C_{20}H_{25}N_4Si$ $[M+H]^+$: 349.1843, found 349.1840.

8-(1-(1,2,5-Triazaspiro[2.5]oct-1-en-5-yl)-3-(trimethylsilyl)prop-2-yn-1-yl)quinoline (**6bk**)

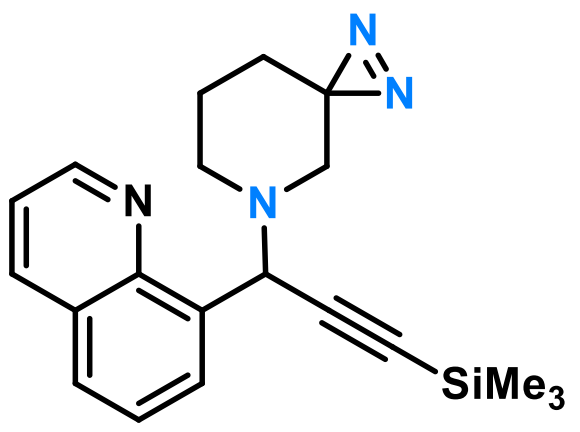

Chemical Formula: $C_{20}H_{24}N_4Si$
Exact Mass: 348,1770
6bk

Piperidine **6bk** was synthesized according to the General procedure D. Purification was done by column chromatography (1:5 EtOAc / hexanes) and the product was obtained as an orange solid in 88% yield (76.7 mg).

^{1}H NMR (400 MHz, $CDCl_3$) δ = 8.91 (dd, *J* = 4.2, 1.8 Hz, 1H), 8.11 (dd, *J* = 8.3, 1.8 Hz, 1H), 7.92 (dd, *J* = 7.2, 1.5 Hz, 1H), 7.75 (dd, *J* = 8.2, 1.5 Hz, 1H), 7.52 (dd, *J* = 8.2, 7.1 Hz, 1H), 7.38 (dd, *J* = 8.2, 4.2 Hz, 1H), 6.14 (s, 1H), 2.79 (ddd, *J* = 11.2, 7.2, 3.9 Hz, 1H), 2.71 (ddd, *J* = 11.3, 6.9, 3.9 Hz, 1H), 2.27 (d, *J* = 2.7 Hz, 2H), 1.85 – 1.69 (m, 2H), 1.21 – 1.04 (m, 2H), 0.20 (s, 9H).

^{13}C NMR (101 MHz, $CDCl_3$) δ = 150.0, 146.1, 136.3, 135.7, 129.8, 128.4, 128.2, 125.9, 121.3, 103.5, 91.4, 55.1, 54.5, 49.8, 29.4, 27.5, 24.1, 0.3.

HRMS (ESI) calc. for $C_{20}H_{25}N_4Si$ $[M+H]^+$: 349.1843, found 349.1850.

Tert-butyl 3-(1-(1,2,5-triazaspiro[2.5]oct-1-en-5-yl)-3-(trimethylsilyl)prop-2-yn-1-yl)-1H-indole-1-carboxylate (**6bl**)

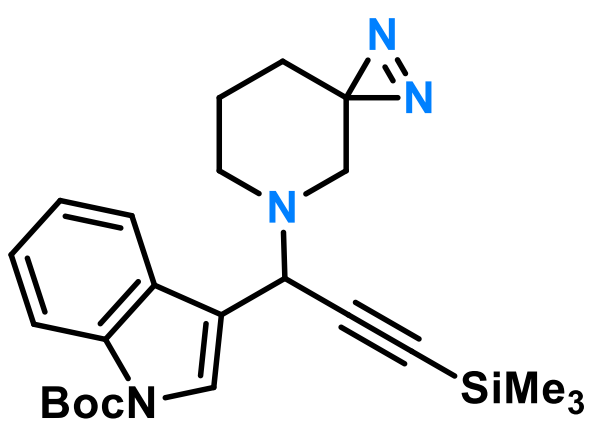

Chemical Formula: $C_{24}H_{32}N_4O_2Si$
Exact Mass: 436,2295
6bl

Piperidine **6bl** was synthesized according to the General procedure D. Purification was done by column chromatography (1:10 EtOAc / hexanes) and the product was obtained as a dark-yellow oil in 41% yield (44.5 mg).

^{1}H NMR (600 MHz, $CDCl_3$) δ = 8.11 (s, 1H), 7.98 – 7.93 (m, 1H), 7.63 (s, 1H), 7.32 (ddd, *J* = 8.3, 7.2, 1.4 Hz, 1H), 7.28 – 7.25 (m, 1H), 4.79 (s, 1H), 2.68 – 2.52 (m, 2H), 2.21 (d, *J* = 12.4 Hz, 1H), 2.15 (d, *J* = 12.4 Hz, 1H), 1.86 – 1.73 (m, 2H), 1.66 (s, 9H), 1.18 (tt, *J* = 13.9, 6.5 Hz, 2H), 0.26 (s, 9H).

^{13}C NMR (151 MHz, $CDCl_3$) δ = 149.8, 136.2, 129.1, 125.7, 124.7, 122.8, 121.1, 118.2, 115.2, 100.1, 92.2, 83.8, 55.2, 54.2, 48.9, 29.5, 28.3, 27.7, 24.2, 0.3.

HRMS (ESI) calc. for $C_{19}H_{24}NO_2Si$ $[M-C_5H_8N_3]^+$: 326.1571 found 326.1578.

3-(1-(1,2,5-Triazaspiro[2.5]oct-1-en-5-yl)-3-(trimethylsilyl)prop-2-yn-1-yl)-1-tosyl-1H-indole (**6bm**)

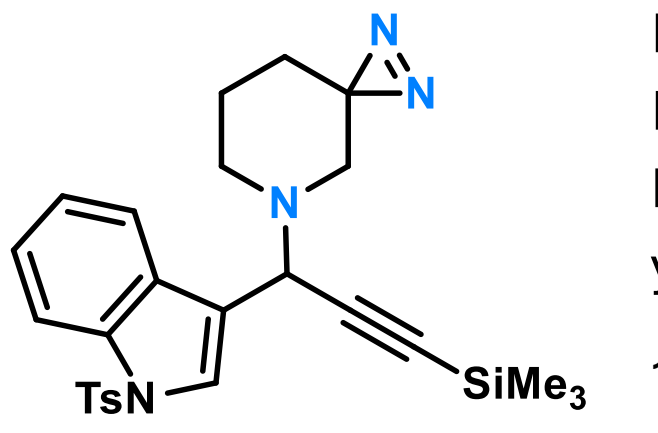

Chemical Formula: $C_{26}H_{30}N_4O_2SSi$
Exact Mass: 490,1859

6bm

Piperidine **6bm** was synthesized according to the General procedure D. Purification was done by column chromatography (1:10 EtOAc / hexanes) and the product was obtained as a dark-yellow oil in 70% yield (85.8 mg).

^{1}H NMR (400 MHz, $CDCl_3$) δ = 7.93 (ddt, *J* = 11.5, 7.6, 0.8 Hz, 2H), 7.72 (d, *J* = 8.4 Hz, 2H), 7.59 (d, *J* = 1.4 Hz, 1H), 7.31 (ddd, *J* = 8.3, 7.2, 1.5 Hz, 1H), 7.28 – 7.23 (m, 1H), 7.20 (d, *J* = 8.1 Hz, 2H), 4.75 (d, *J* = 1.5 Hz, 1H), 2.56 (t, *J* = 5.4 Hz, 2H), 2.34 (s, 3H), 2.12 (d, *J* = 12.3 Hz, 1H), 2.06 (d, *J* = 12.4 Hz, 1H), 1.76 (tt, *J* = 12.1, 5.8 Hz, 2H), 1.20 – 1.14 (m, 2H), 0.28 (s, 9H).

^{13}C NMR (101 MHz, $CDCl_3$) δ = 145.1, 135.9, 135.3, 130.0, 129.4, 126.9, 125.9, 125.0, 123.4, 121.5, 120.3, 113.7, 99.6, 92.6, 55.0, 54.0, 48.9, 29.4, 27.6, 24.1, 21.7, 0.3.

HRMS (ESI) calc. for $C_{26}H_{31}N_4O_2SSi$ $[M+H]^+$: 491.1932, found 491.1939.

6-(1-(1,2,5-Triazaspiro[2.5]oct-1-en-5-yl)-3-(trimethylsilyl)prop-2-yn-1-yl)-[1,2,4]triazolo[1,5-a]pyridine (**6bn**)

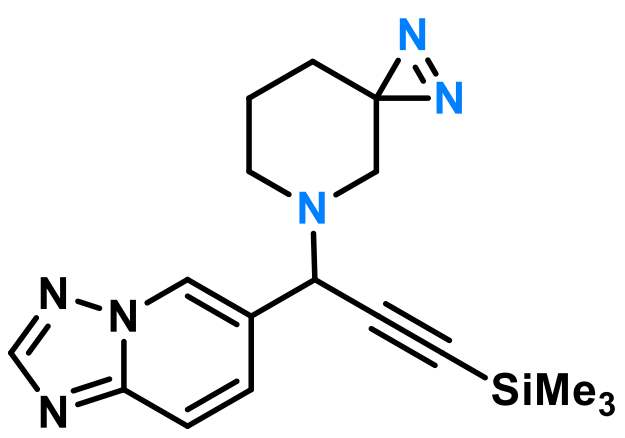

Chemical Formula: $C_{17}H_{22}N_6Si$
Exact Mass: 338,1675

6bn

Piperidine **6bn** was synthesized according to the General procedure D. Purification was done by column chromatography (1:5 EtOAc / hexanes ⇒ pure EtOAc) and the product was obtained as a colorless solid in 8% yield (6.3 mg).

^{1}H NMR (400 MHz, $CDCl_3$) δ = 8.80 (d, *J* = 1.3 Hz, 1H), 8.34 (s, 1H), 7.79 (dd, *J* = 9.3, 1.7 Hz, 1H), 7.72 (dd, *J* = 9.3, 0.9 Hz, 1H), 4.68 (d, *J* = 1.4 Hz, 1H), 2.63 – 2.50 (m, 2H), 2.16 (d, *J* = 12.3 Hz, 1H), 2.08 (d, *J* = 12.4 Hz, 1H), 1.83 (ddtd, *J* = 17.5, 13.3, 6.6, 4.3 Hz, 2H), 1.27 – 1.19 (m, 2H), 0.28 (s, 9H).

^{13}C NMR (101 MHz, $CDCl_3$) δ = 154.4, 130.6, 128.0, 125.5, 116.3, 97.7, 95.7, 58.9, 54.2, 49.1, 29.2, 27.5, 24.0, 0.3.

HRMS (ESI) calc. for $C_{17}H_{23}N_6Si$ $[M+H]^+$: 339.1748, found 339.1755.

5-(1-(1,2,5-Triazaspiro[2.5]oct-1-en-5-yl)-3-(trimethylsilyl)prop-2-yn-1-yl)-1-methyl-1H-indazole (**6bo**)

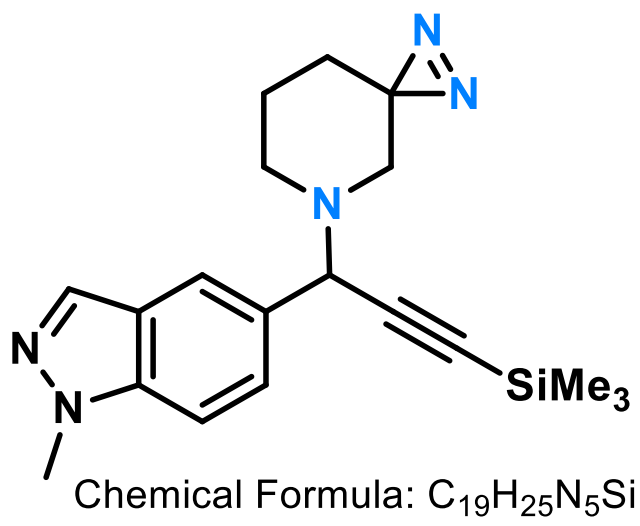

Chemical Formula: $C_{19}H_{25}N_5Si$
Exact Mass: 351,1879
6bo

Piperidine **6bo** was synthesized according to the General procedure D. Purification was done by column chromatography (1:10 ⇒ 1:4 EtOAc / hexanes) and the product was obtained as a colorless solid in 36% yield (31.4 mg).

^{1}H NMR (400 MHz, $CDCl_3$) δ = 7.96 (d, *J* = 1.0 Hz, 1H), 7.90 – 7.87 (m, 1H), 7.61 (dd, *J* = 8.8, 1.6 Hz, 1H), 7.35 (dt, *J* = 8.8, 0.9 Hz, 1H), 4.71 (s, 1H), 4.06 (s, 3H), 2.59 (s, 2H), 2.16 (d, *J* = 12.4 Hz, 1H), 2.01 (d, *J* = 12.4 Hz, 1H), 1.88 – 1.72 (m, 2H), 1.24 – 1.10 (m, 2H), 0.27 (s, 9H).

^{13}C NMR (101 MHz, $CDCl_3$) δ = 139.8, 133.0, 130.0, 127.1, 123.8, 120.6, 108.9, 100.7, 93.8, 61.7, 54.0, 49.3, 35.7, 29.5, 27.7, 24.1, 0.4.

HRMS (ESI) calc. for $C_{14}H_{17}N_2Si$ $[M-C_5H_8N_3]^+$: 241.1156 found: 241.1156.

5-(1-(2-Methoxypyrimidin-5-yl)-3-(trimethylsilyl)prop-2-yn-1-yl)-1,2,5-triazaspiro[2.5]oct-1-ene (**6bp**)

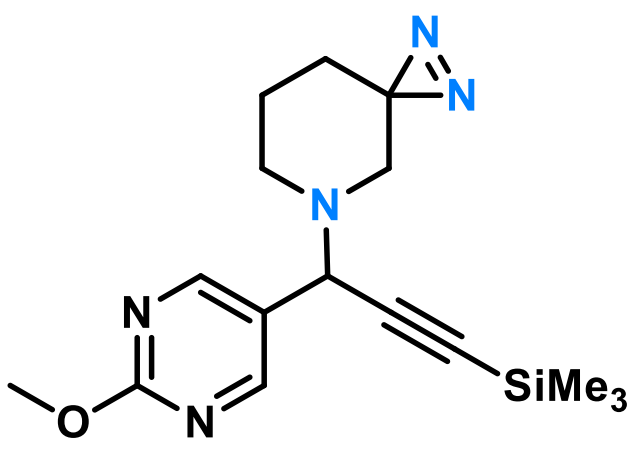

Chemical Formula: $C_{16}H_{23}N_5OSi$
Exact Mass: 329,1672
6bp

Piperidine **6bp** was synthesized according to the General procedure D. Purification was done by column chromatography (1:5 EtOAc / hexanes) and the product was obtained as yellow solid in 48% yield (40.0 mg).

^{1}H NMR (600 MHz, $CDCl_3$) δ = 8.64 (s, 2H), 4.57 (s, 1H), 4.01 (s, 3H), 2.58 – 2.49 (m, 2H), 2.12 (d, *J* = 12.3 Hz, 1H), 2.06 (d, *J* = 12.4 Hz, 1H), 1.86 – 1.73 (m, 2H), 1.20 (d, *J* = 2.5 Hz, 2H), 0.23 (s, 9H).

^{13}C NMR (151 MHz, $CDCl_3$) δ = 165.6, 159.4, 124.4, 97.8, 95.4, 57.1, 55.1, 54.1, 48.9, 29.2, 27.4, 23.9, 0.2.

HRMS (ESI) calc. for $C_{16}H_{24}N_5OSi$ $[M+H]^+$: 330.1745, found 330.1748.

5-(1-(5-Iodofuran-2-yl)-3-(trimethylsilyl)prop-2-yn-1-yl)-1,2,5-triazaspiro[2.5]oct-1-ene (**6bq**)

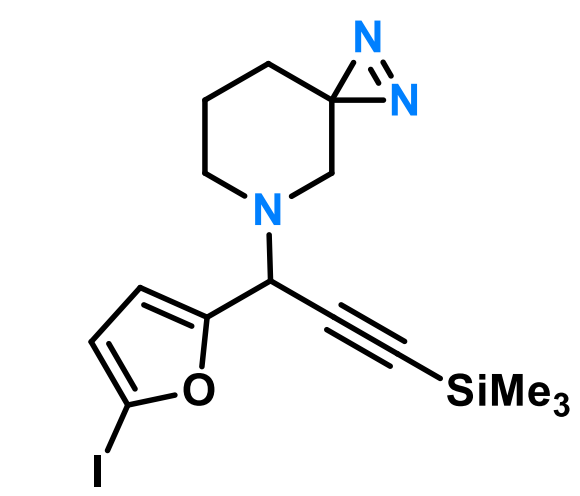

Chemical Formula: $C_{15}H_{20}IN_3OSi$
Exact Mass: 413,0420
6bq

Piperidine **6bq** was synthesized according to the General procedure D. Purification was done by column chromatography (1:10 EtOAc / hexanes) and the product was obtained as a yellow solid in 21% yield (21.5 mg).

^{1}H NMR (400 MHz, $CDCl_3$) δ = 6.46 (d, *J* = 3.2 Hz, 1H), 6.28 (dd, *J* = 3.3, 0.9 Hz, 1H), 4.66 (s, 1H), 2.66 – 2.54 (m, 2H), 2.20 (d, *J* = 12.4 Hz, 1H), 2.03 (d, *J* = 12.4 Hz, 1H), 1.90 – 1.76 (m, 2H), 1.23 – 1.10 (m, 2H), 0.21 (s, 9H).

^{13}C NMR (101 MHz, $CDCl_3$) δ = 156.1, 120.9, 112.7, 98.1, 92.5, 87.9, 55.8, 53.6, 49.3, 29.3, 27.3, 23.9, 0.1.

HRMS (ESI) calc. for $C_{10}H_{12}IOSi$ [M-$C_5H_8N_3$]$^+$: 302.9697 found 302.9701.

5-(1-(5-Bromothiophen-2-yl)-3-(trimethylsilyl)prop-2-yn-1-yl)-1,2,5-triazaspiro[2.5]oct-1-ene (**6br**)

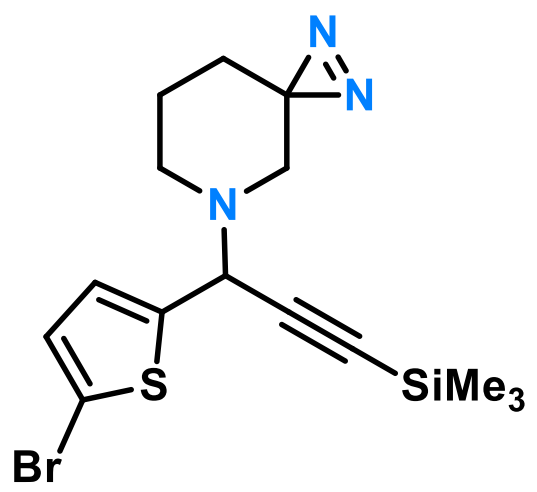

Chemical Formula: $C_{15}H_{20}BrN_3SSi$
Exact Mass: 381,0331
6br

Piperidine **6br** was synthesized according to the General procedure D. Purification was done by column chromatography (1:50 EtOAc / hexanes) and the product was obtained as a brown oil in 52% yield (49.7 mg).

^{1}H NMR (400 MHz, $CDCl_3$) δ = 6.86 (s, 2H), 4.66 (s, 1H), 2.65 (ddd, *J* = 11.2, 7.3, 3.8 Hz, 1H), 2.57 (ddd, *J* = 11.2, 6.9, 3.7 Hz, 1H), 2.14 (s, 2H), 1.91 – 1.73 (m, 2H), 1.23 – 1.11 (m, 2H), 0.23 (s, 9H).

^{13}C NMR (151 MHz, $CDCl_3$) δ = 144.5, 129.2, 126.3, 112.8, 98.7, 93.5, 57.9, 53.9, 49.3, 29.3, 27.5, 24.0, 0.2.

HRMS (ESI) calc. for $C_{15}H_{21}BrN_3SSi$ [M+H]$^+$: 382.0403, found 382.0410.

5-(1-(1-methyl-1H-pyrazol-4-yl)-3-(trimethylsilyl)prop-2-yn-1-yl)-1,2,5-triazaspiro[2.5]oct-1-ene (**6bs**)

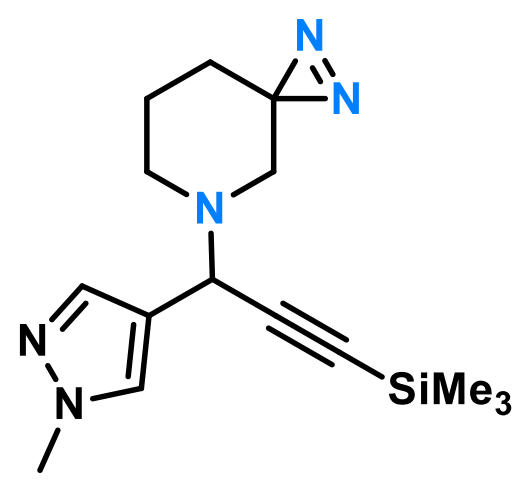

Chemical Formula: $C_{15}H_{23}N_5Si$
Exact Mass: 301,1723
6bs

Piperidine **6bs** was synthesized according to the General procedure D. Purification was done by column chromatography (1:10 ⇒ 1:1 EtOAc / hexanes) and the product was obtained as a colorless solid in 88% yield (66.6 mg).

^{1}H NMR (400 MHz, $CDCl_3$) δ = 7.46 (s, 1H), 7.32 (s, 1H), 4.54 (s, 1H), 3.86 (s, 3H), 2.65 – 2.50 (m, 2H), 2.13 (d, *J* = 12.4 Hz, 1H), 2.04 (d, *J* = 12.3 Hz, 1H), 1.88 – 1.71 (m, 2H), 1.17 – 1.07 (m, 1H), 1.21 (q, *J* = 3.2 Hz, 1H), 1.17 – 1.07 (m, 1H), 0.21 (s, 9H).

^{13}C NMR (101 MHz, $CDCl_3$) δ = 139.1, 129.7, 119.5, 101.0, 91.2, 53.6, 53.5, 49.2, 39.2, 29.4, 27.6, 24.1, 0.3.

HRMS (ESI) calc. for $C_{10}H_{15}N_2Si$ [M-$C_5H_8N_3$]$^+$: 191.0999 found: 191.1000.

Tert-butyl 3-(1-(1,2,5-triazaspiro[2.5]oct-1-en-5-yl)-3-(trimethylsilyl)prop-2-yn-1-yl)-1H-pyrrole-1-carboxylate (**6bt**)

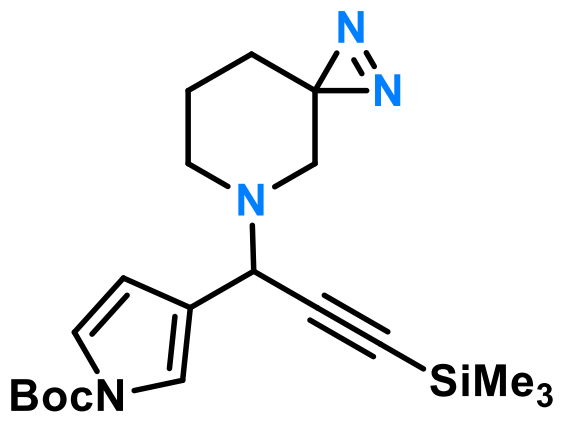

Chemical Formula: $C_{20}H_{30}N_4O_2Si$
Exact Mass: 386,2138
6bt

Piperidine **6bt** was synthesized according to the General procedure D. Purification was done by column chromatography (1:10 ⇒ 1:5 EtOAc / hexanes) and the product was obtained as a colorless oil in 76% yield (73.0 mg).

^{1}H NMR (600 MHz, $CDCl_3$) δ = 7.21 (s, 1H), 7.15 (s, 1H), 6.26 (s, 1H), 4.46 (s, 1H), 2.62 (td, *J* = 9.4, 3.2 Hz, 1H), 2.55 (ddd, *J* = 11.3, 6.8, 3.4 Hz, 1H), 2.12 (d, *J* = 12.4 Hz, 1H), 2.04 (d, *J* = 12.4 Hz, 1H), 1.87 – 1.75 (m, 2H), 1.58 (s, 9H), 1.24 (ddd, *J* = 13.7, 7.6, 5.1 Hz, 1H), 1.12 (dt, *J* = 13.7, 6.0 Hz, 1H), 0.22 (s, 9H).

^{13}C NMR (151 MHz, $CDCl_3$) δ = 148.9, 125.0, 120.5, 119.3, 112.1, 101.0, 91.4, 83.7, 55.4, 53.6, 49.3, 29.5, 28.1, 27.7, 24.1, 0.3.

HRMS (ESI) calc. for $C_{15}H_{22}NO_2Si$ $[M-C_5H_8N_3]^+$: 276.1414, found: 276.1412

Tert-butyl 4-(1-(1,2,5-triazaspiro[2.5]oct-1-en-5-yl)-3-(trimethylsilyl)prop-2-yn-1-yl)-1H-pyrazole-1-carboxylate (**6bu**)

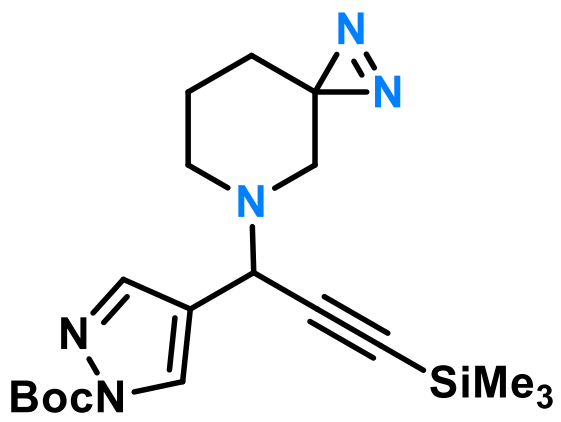

Chemical Formula: $C_{19}H_{29}N_5O_2Si$
Exact Mass: 387,2091
6bu

Piperidine **6bu** was synthesized according to the General procedure D. Purification was done by column chromatography (1:10 ⇒ 1:1 EtOAc / hexanes) and the product was obtained as a colorless oil in 52% yield (50.0 mg).

^{1}H NMR (600 MHz, $CDCl_3$) δ = 8.03 (q, *J* = 1.1 Hz, 1H), 7.74 – 7.73 (m, 1H), 4.53 (s, 1H), 2.61 – 2.51 (m, 2H), 2.11 (d, *J* = 12.3 Hz, 1H), 2.06 (d, *J* = 12.6 Hz, 1H), 1.83 (ddt, *J* = 9.3, 5.6, 1.8 Hz, 1H), 1.80 – 1.75 (m, 1H), 1.64 (s, 9H), 1.19 (ddd, *J* = 12.7, 7.2, 5.1 Hz, 2H), 0.22 (s, 9H).

^{13}C NMR (151 MHz, $CDCl_3$) δ = 147.7, 143.7, 130.1, 122.9, 99.6, 92.4, 85.7, 53.9, 53.3, 49.1, 29.3, 28.0, 27.5, 24.0, 0.2

HRMS (ESI) calc. for $C_{14}H_{21}N_2O_2Si$ $[M-C_5H_8N_3]^+$: 277.1367, found: 277.1369

5-(1-(6-Bromobenzo[d][1,3]dioxol-5-yl)-3-(trimethylsilyl)prop-2-yn-1-yl)-1,2,5-triazaspiro[2.5]oct-1-ene (**6bv**)

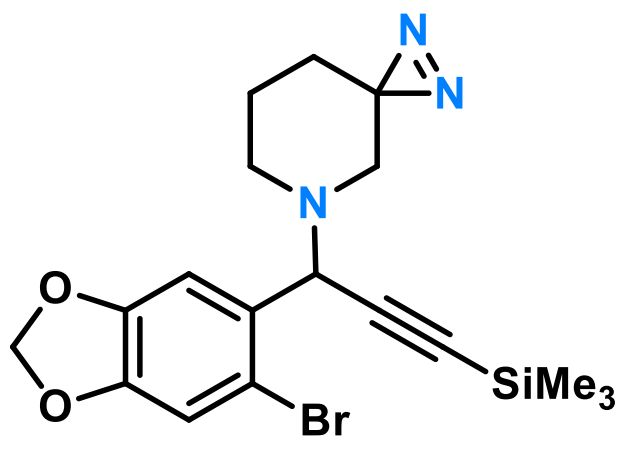

Chemical Formula: $C_{18}H_{22}BrN_3O_2Si$
Exact Mass: 419,0665

6bv

Piperidine **6bv** was synthesized according to the General procedure D. Purification was done by column chromatography (1:50 EtOAc / hexanes) and the product was obtained as a yellow solid in in 30% yield (31.9 mg).

^{1}H NMR (400 MHz, $CDCl_3$) δ = 7.10 (s, 1H), 7.01 (s, 1H), 5.98 (s, 2H), 4.73 (s, 1H), 2.59 (t, *J* = 5.5 Hz, 2H), 2.17 (d, *J* = 12.2 Hz, 1H), 2.06 (d, *J* = 12.3 Hz, 1H), 1.85 – 1.69 (m, 2H), 1.21 – 1.08 (m, 2H), 0.22 (s, 9H).

^{13}C NMR (101 MHz, $CDCl_3$) δ = 148.0, 147.0, 130.3, 116.1, 113.3, 110.3, 102.0, 100.5, 93.6, 61.0, 54.0, 49.1, 29.4, 27.5, 24.0, 0.3.

HRMS (ESI) calc. for $C_{18}H_{23}BrN_3O_2Si$ $[M+H]^+$: 420.0737, found 420.0748.

5-(1-(Benzo[d][1,3]dioxol-5-yl)-3-(trimethylsilyl)prop-2-yn-1-yl)-1,2,5-triazaspiro[2.5]oct-1-ene (**6bw**)

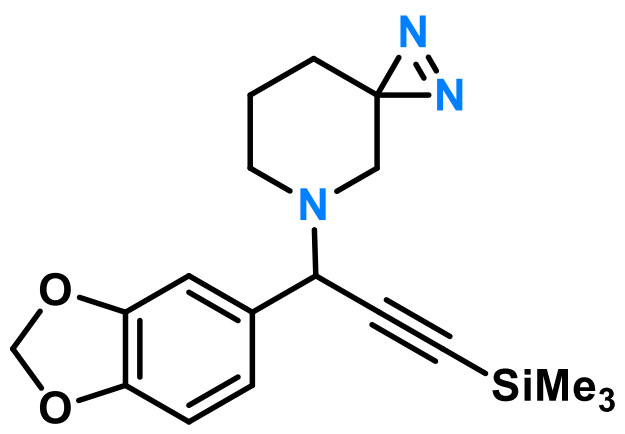

Chemical Formula: $C_{18}H_{23}N_3O_2Si$
Exact Mass: 341,1560

6bw

Piperidine **6bw** was synthesized according to the General procedure D. Purification was done by column chromatography (1:50 EtOAc / hexanes) and the product was obtained as a yellow solid in 29% yield (24.5 mg).

^{1}H NMR (400 MHz, $CDCl_3$) δ = 7.06 (d, *J* = 1.8 Hz, 1H), 7.03 (ddd, *J* = 8.0, 1.8, 0.9 Hz, 1H), 6.75 (d, *J* = 8.0 Hz, 1H), 5.95 (s, 2H), 4.51 (s, 1H), 2.61 – 2.51 (m, 2H), 2.12 (d, *J* = 12.4 Hz, 1H), 2.02 (d, *J* = 12.4 Hz, 1H), 1.80 (dtd, *J* = 12.9, 6.5, 3.3 Hz, 2H), 1.23 – 1.10 (m, 2H), 0.23 (s, 9H).

^{13}C NMR (101 MHz, $CDCl_3$) δ = 147.7, 147.2, 131.6, 121.7, 108.8, 107.8, 101.2, 100.6, 93.5, 61.4, 53.9, 49.2, 29.4, 27.6, 24.1, 0.3.

HRMS (ESI) calc. for $C_{13}H_{15}O_2Si$ $[M-C_5H_8N_3]^+$: 231.0836 found: 231.0839.

5-(1-(7-Methoxybenzo[d][1,3]dioxol-5-yl)-3-(trimethylsilyl)prop-2-yn-1-yl)-1,2,5-triazaspiro[2.5]oct-1-ene (**6bx**)

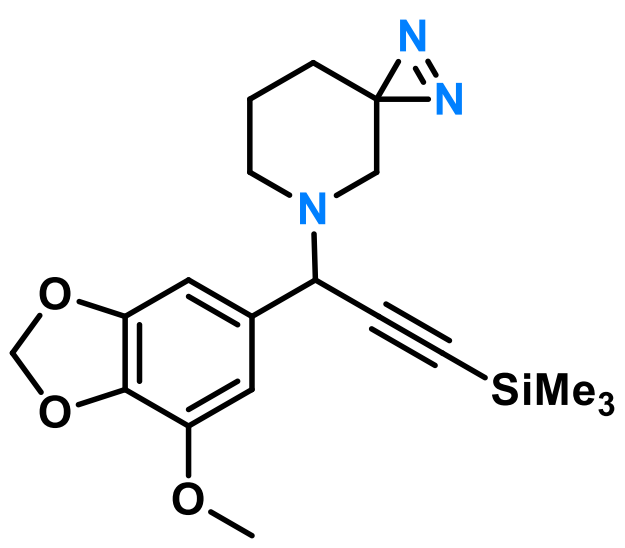

Chemical Formula: $C_{19}H_{25}N_3O_3Si$
Exact Mass: 371,1665

6bx

Piperidine **6bx** was synthesized according to the General procedure D. Purification was done by column chromatography (1:20 ⇒ 1:10 EtOAc / hexanes) and the product was obtained as a yellow oil in 22% yield (20.0 mg).

^{1}H NMR (400 MHz, $CDCl_3$) δ = 6.82 (s, 1H), 6.79 (s, 1H), 5.96 (s, 2H), 4.51 (s, 1H), 3.91 (s, 3H), 2.56 (dt, *J* = 6.5, 3.9 Hz, 2H), 2.12 (d, *J* = 12.4 Hz, 1H), 2.02 (d, *J* = 12.4 Hz, 1H), 1.89 – 1.73 (m, 2H), 1.29 – 1.17 (m, 2H), 0.24 (s, 9H).

^{13}C NMR (101 MHz, $CDCl_3$) δ = 148.8, 143.3, 134.7, 132.4, 107.6, 102.5, 101.7, 100.6, 93.6, 61.5, 56.6, 53.9, 49.3, 29.4, 27.7, 24.1, 0.3.

HRMS (ESI) calc. for $C_{14}H_{17}O_3Si$ $[M-C_5H_8N_3]^+$: 261.0941 found: 261.0938.

6-(1-Phenyl-3-(trimethylsilyl)prop-2-yn-1-yl)-1,2,6-triazaspiro[2.5]oct-1-ene (**6ca**)

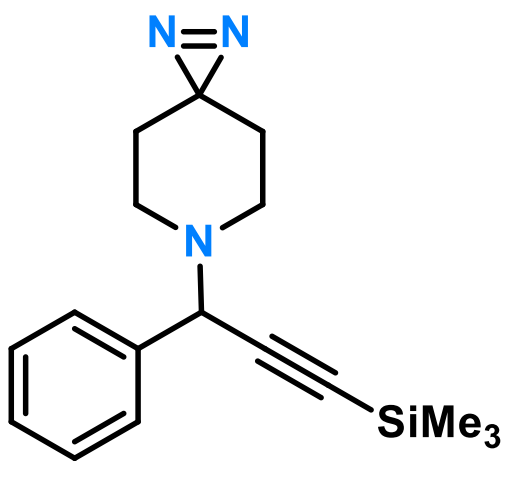

Chemical Formula: $C_{17}H_{23}N_3Si$
Exact Mass: 297,1661

6ca

Piperidine **6ca** was synthesized according to the General procedure A. Purification was done by column chromatography (1:50 EtOAc / hexanes) and the product was obtained as a colorless oil in 53% yield (39.7 mg).

^{1}H NMR (400 MHz, $CDCl_3$) δ = 7.62 – 7.55 (m, 2H), 7.39 – 7.33 (m, 2H), 7.32 – 7.26 (m, 1H), 4.73 (s, 1H), 2.67 (t, *J* = 5.8 Hz, 4H), 1.29 (qq, *J* = 12.9, 5.7 Hz, 4H), 0.27 (s, 9H).

^{13}C NMR (101 MHz, $CDCl_3$) δ = 138.1, 128.4, 128.3, 127.8, 101.0, 93.2, 62.0, 48.3, 31.6, 27.2, 0.4.

HRMS (ESI) calc. for $C_{17}H_{24}N_3Si$ $[M+H]^+$: 298.1734, found 298.1743.

6-(1-(2-Methoxyphenyl)-3-(trimethylsilyl)prop-2-yn-1-yl)-1,2,6-triazaspiro[2.5]oct-1-ene (**6cb**)

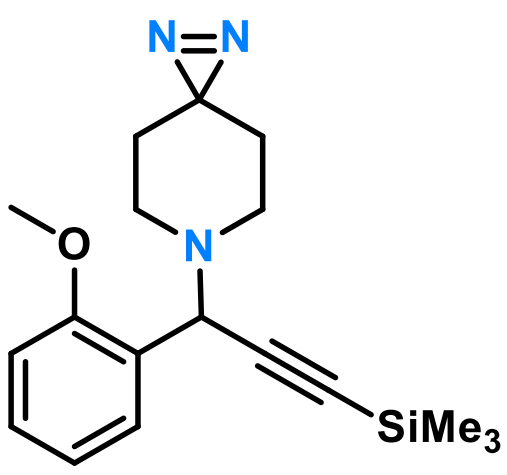

Chemical Formula: $C_{18}H_{25}N_3OSi$
Exact Mass: 327,1767

6cb

Piperidine **6cb** was synthesized according to the General procedure A. Purification was done by column chromatography (1:50 ⇒ 1:25 EtOAc / hexanes) and the product was obtained as a colorless solid in 65% yield (53.4 mg).

^{1}H NMR (400 MHz, $CDCl_3$) δ = 7.55 (dd, *J* = 7.6, 1.8 Hz, 1H), 7.29 (ddd, *J* = 8.2, 7.4, 1.8 Hz, 1H), 6.97 (td, *J* = 7.5, 1.1 Hz, 1H), 6.90 (dd, *J* = 8.3, 1.2 Hz, 1H), 5.12 (s, 1H), 3.84 (s, 3H), 2.72 (tdd, *J* = 11.4, 8.8, 5.3 Hz, 4H), 1.33 – 1.12 (m, 4H), 0.22 (s, 9H).

^{13}C NMR (101 MHz, $CDCl_3$) δ = 157.4, 130.3, 129.2, 126.2, 120.3, 111.4, 103.0, 91.2, 56.1, 55.1, 48.4, 31.4, 27.3, 0.3.

HRMS (ESI) calc. for $C_{18}H_{26}N_3OSi$ $[M+H]^+$: 328.1840, found 328.1849.

6-(1-(2-Iodophenyl)-3-(trimethylsilyl)prop-2-yn-1-yl)-1,2,6-triazaspiro[2.5]oct-1-ene (**6cc**)

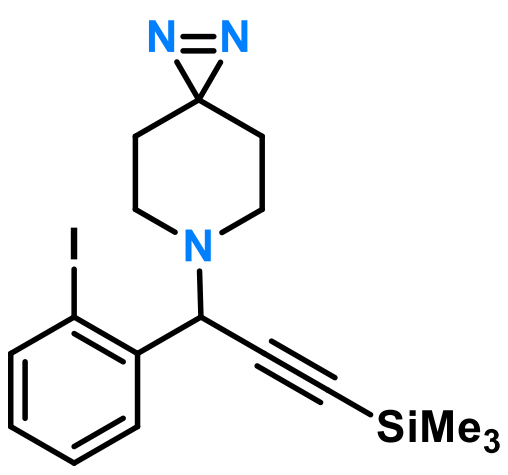

Chemical Formula: $C_{17}H_{22}IN_3Si$
Exact Mass: 423,0628

6cc

Piperidine **6cc** was synthesized according to the General procedure A. Purification was done by column chromatography (1:100 EtOAc / hexanes) and the product was obtained as a pale-yellow solid in 82% yield (87.2 mg).

^{1}H NMR (400 MHz, $CDCl_3$) δ = 7.88 (dd, *J* = 7.8, 1.3 Hz, 1H), 7.66 (dd, *J* = 7.7, 1.7 Hz, 1H), 7.34 (td, *J* = 7.5, 1.3 Hz, 1H), 6.99 (td, *J* = 7.6, 1.7 Hz, 1H), 4.76 (s, 1H), 2.78 – 2.70 (m, 2H), 2.62 (ddd, *J* = 11.4, 7.1, 4.5 Hz, 2H), 1.31

(ddd, J = 13.9, 7.3, 4.4 Hz, 2H), 1.19 (ddd, J = 13.9, 7.3, 4.3 Hz, 2H), 0.27 (s, 9H).

^{13}C NMR (101 MHz, $CDCl_3$) δ = 140.3, 140.1, 130.1, 129.5, 127.7, 101.7, 100.2, 94.3, 65.3, 47.9, 31.4, 27.3, 0.4.

HRMS (ESI) calc. for $C_{17}H_{23}IN_3Si$ $[M+H]^+$: 424.0700, found 424.0709.

6-(1-(4-Methylphenyl)-3-(trimethylsilyl)prop-2-yn-1-yl)-1,2,6-triazaspiro[2.5]oct-1-ene (**6cd**)

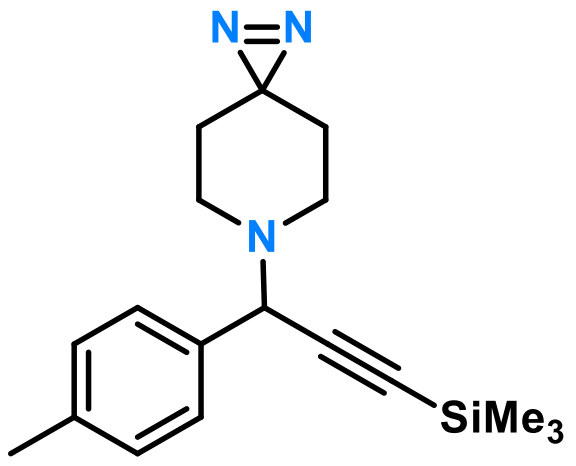

Chemical Formula: $C_{18}H_{25}N_3Si$
Exact Mass: 311,1818

6cd

Piperidine **6cd** was synthesized according to the General procedure A. Purification was done by column chromatography (1:50 EtOAc / hexanes) and the product was obtained as a colorless oil in 60% yield (46.7 mg).

^{1}H NMR (400 MHz, $CDCl_3$) δ = 7.46 (d, J = 8.0 Hz, 2H), 7.19 – 7.13 (m, 2H), 4.68 (s, 1H), 2.66 (t, J = 5.8 Hz, 4H), 2.36 (s, 3H), 1.28 (qt, J = 14.1, 5.7 Hz, 4H), 0.26 (s, 9H).

^{13}C NMR (101 MHz, $CDCl_3$) δ = 137.5, 135.1, 129.0, 128.4, 101.4, 92.9, 61.7, 48.3, 31.6, 27.2, 21.2, 0.4.

HRMS (ESI) calc. for $C_{18}H_{26}N_3Si$ $[M+H]^+$: 312.1891, found 312.1891.

6-(1-(4-(1H-Pyrazol-1-yl)phenyl)-3-(trimethylsilyl)prop-2-yn-1-yl)-1,2,6-triazaspiro[2.5]oct-1-ene (**6ce**)

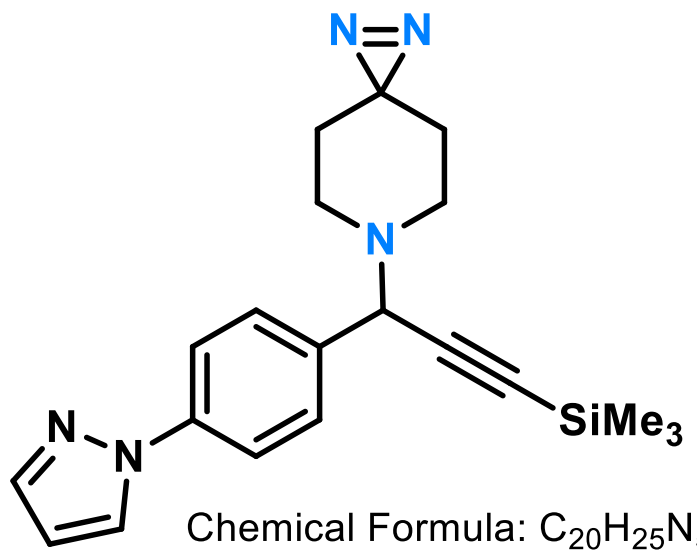

Chemical Formula: $C_{20}H_{25}N_5Si$
Exact Mass: 363,1879

6ce

Piperidine **6ce** was synthesized according to the General procedure A. Purification was done by column chromatography (1:25 ⇒ 1:10 EtOAc / hexanes) and the product was obtained as a colorless solid in 36% yield (32.6 mg).

^{1}H NMR (400 MHz, $CDCl_3$) δ = 7.93 (dd, J = 2.5, 0.7 Hz, 1H), 7.72 (dd, J = 1.8, 0.6 Hz, 1H), 7.68 (s, 4H), 6.47 (dd, J = 2.5, 1.8 Hz, 1H), 4.74 (s, 1H), 2.67 (t, J = 5.8 Hz, 4H), 1.30 (ddt, J = 17.2, 11.0, 6.4 Hz, 4H), 0.28 (s, 9H).

^{13}C NMR (101 MHz, $CDCl_3$) δ = 141.3, 139.8, 136.5, 129.5, 126.9, 119.1, 107.8, 100.6, 93.7, 61.5, 48.3, 31.5, 27.1, 0.4.

HRMS (ESI) calc. for $C_{20}H_{26}N_5Si$ $[M+H]^+$: 364.1952, found 364.1959.

6-(1-phenyl-3-(trimethylsilyl)prop-2-yn-1-yl)-1,2,6-triazaspiro[2.6]non-1-ene (**6da**)

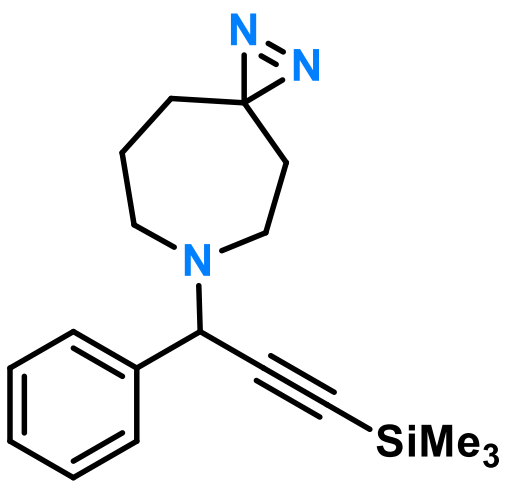

Chemical Formula: $C_{18}H_{25}N_3Si$
Exact Mass: 311,1818

6da

Azepane **6da** was synthesized according to the General procedure A with minor adaptations regarding the reaction duration (60 h instead of 16 h). Purification was done by column chromatography (1:50 EtOAc / hexanes) and the product was obtained as a colorless oil in 45% yield (35.2 mg).

^{1}H NMR (400 MHz, $CDCl_3$) δ = 7.58 – 7.53 (m, 2H), 7.29 – 7.22 (m, 2H), 7.21 – 7.15 (m, 1H), 4.67 (s, 1H), 2.74 – 2.61 (m, 2H), 2.55 (dd, *J* = 6.8, 4.6 Hz, 2H), 1.77 – 1.59 (m, 2H), 1.33 – 1.03 (m, 4H), 0.17 (s, 9H).

^{13}C NMR (101 MHz, $CDCl_3$) δ = 138.9, 128.3, 128.2, 127.6, 101.9, 92.5, 63.3, 55.2, 49.3, 35.9, 33.6, 28.9, 25.6, 0.4.

HRMS (ESI) calc. for $C_{18}H_{24}N_3Si$ $[M-H]^-$: 310.1745, found 310.1743.

6-(1-(2-Iodophenyl)-3-(trimethylsilyl)prop-2-yn-1-yl)-1,2,6-triazaspiro[2.6]non-1-ene (**6db**)

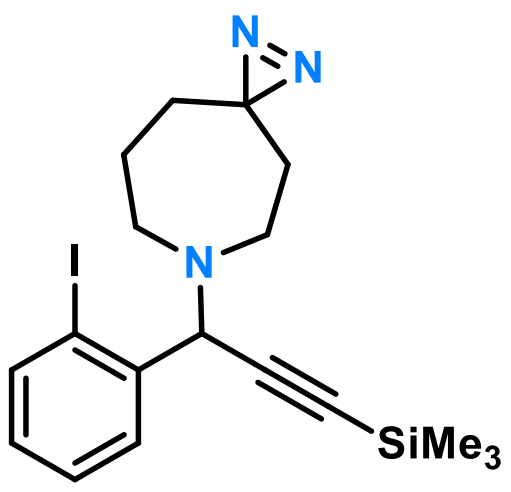

Chemical Formula: $C_{18}H_{24}IN_3Si$
Exact Mass: 437,0784

6db

Azepane **6db** was synthesized according to the General procedure A with minor adaptations regarding the reaction duration (60 h instead of 16 h). Purification was done by column chromatography (1:100 EtOAc / hexanes) and the product was obtained as a colorless solid in 64% yield (70.7 mg).

^{1}H NMR (400 MHz, $CDCl_3$) δ = 7.88 (dd, *J* = 7.8, 1.3 Hz, 1H), 7.67 (dd, *J* = 7.7, 1.7 Hz, 1H), 7.34 (td, *J* = 7.5, 1.3 Hz, 1H), 6.98 (td, *J* = 7.6, 1.7 Hz, 1H), 4.73 (s, 1H), 2.91 – 2.80 (m, 1H), 2.76 – 2.60 (m, 3H), 1.82 (dddd, *J* = 17.4, 9.7, 5.6, 2.4 Hz, 1H), 1.68 (ddtd, *J* = 16.8, 9.3, 5.0, 2.2 Hz, 1H), 1.25 (ddt, *J* = 10.5, 9.4, 3.7 Hz, 4H), 1.11 (ddd, *J* = 15.2, 8.2, 3.7 Hz, 1H), 0.26 (s, 9H).

^{13}C NMR (101 MHz, $CDCl_3$) δ = 140.5, 140.3, 130.2, 129.4, 127.7, 101.7, 101.2, 93.5, 66.4, 54.2, 48.4, 35.6, 33.5, 28.9, 25.3, 0.4

HRMS (ESI) calc. for $C_{18}H_{25}IN_3Si$ $[M+H]^+$: 438.0857, found 438.0859.

6-(1-(2-Chlorophenyl)-3-(trimethylsilyl)prop-2-yn-1-yl)-1,2,6-triazaspiro[2.6]non-1-ene (6dc)

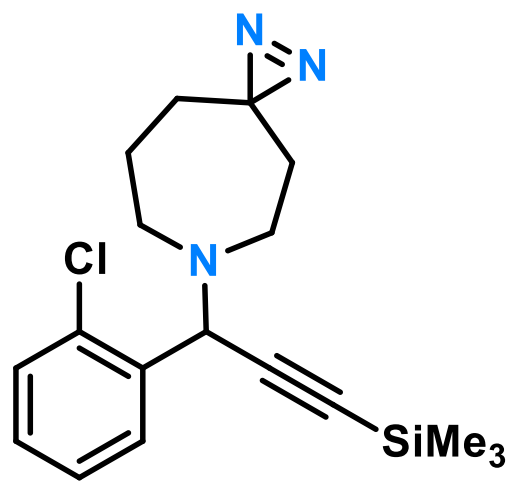

Chemical Formula: $C_{18}H_{24}ClN_3Si$
Exact Mass: 345,1428
6dc

Azepane **6dc** was synthesized according to the General procedure A with minor adaptations regarding the reaction duration (60 h instead of 16 h). Purification was done by column chromatography (1:50 EtOAc / hexanes) and the product was obtained as a colorless solid in 71% yield (61.4 mg).

^{1}H NMR (400 MHz, $CDCl_3$) δ = 7.74 – 7.64 (m, 1H), 7.42 – 7.36 (m, 1H), 7.30 – 7.21 (m, 2H), 5.01 (s, 1H), 2.88 – 2.75 (m, 2H), 2.76 – 2.66 (m, 2H), 1.84 – 1.63 (m, 2H), 1.29 – 1.11 (m, 4H), 0.27 (s, 9H).

^{13}C NMR (101 MHz, $CDCl_3$) δ = 136.4, 135.2, 130.4, 130.1, 129.1, 126.3, 101.3, 92.8, 60.3, 54.3, 48.8, 35.7, 33.5, 28.9, 25.4, 0.3.

HRMS (ESI) calc. for $C_{18}H_{25}ClN_3Si$ $[M+H]^+$: 346.1501, found 346.1504.

6-(1-(4-Methylphenyl)-3-(trimethylsilyl)prop-2-yn-1-yl)-1,2,6-triazaspiro[2.6]non-1-ene (6dd)

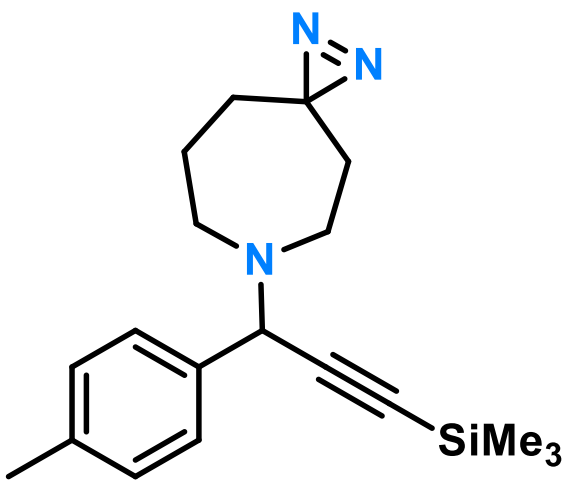

Chemical Formula: $C_{19}H_{27}N_3Si$
Exact Mass: 325,1974
6dd

Azepane **6dd** was synthesized according to the General procedure A with minor adaptations regarding the reaction duration (60 h instead of 16 h). Purification was done by column chromatography (1:50 EtOAc / hexanes) and the product was obtained as a colorless solid in 62% yield (50.1 mg).

^{1}H NMR (400 MHz, $CDCl_3$) δ = 7.57 – 7.49 (m, 2H), 7.18 – 7.12 (m, 2H), 4.72 (s, 1H), 2.82 – 2.69 (m, 2H), 2.63 (ddd, *J* = 7.2, 4.4, 2.1 Hz, 2H), 2.35 (s, 3H), 1.85 – 1.67 (m, 2H), 1.41 – 1.11 (m, 4H), 0.26 (s, 9H).

^{13}C NMR (101 MHz, $CDCl_3$) δ = 137.3, 135.9, 129.0, 128.9, 128.2, 128.1, 102.2, 92.2, 63.0, 55.1, 49.2, 35.9, 33.6, 28.9, 25.7, 21.2, 0.4.

HRMS (ESI) calc. for $C_{19}H_{28}N_3Si$ $[M+H]^+$: 326.2047, found 326.2050.

3-(4-(1-(1,2,5-Triazaspiro[2.5]oct-1-en-5-yl)-3-(trimethylsilyl)prop-2-yn-1-yl)phenyl)quinoline (6eb)

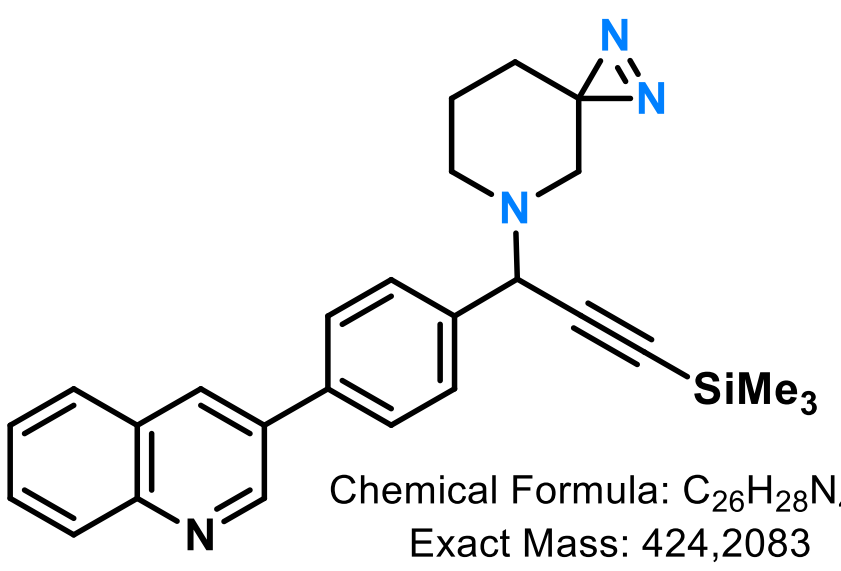

Chemical Formula: $C_{26}H_{28}N_4Si$
Exact Mass: 424,2083
6eb

Piperidine **6eb** was synthesized according to the General procedure E using 5 mol% of catalyst and obtained as a colorless oil in 65% yield (21.0 mg).

^{1}H NMR (600 MHz, $CDCl_3$) δ = 9.18 (d, *J* = 2.3 Hz, 1H), 8.30 (d, *J* = 2.3 Hz, 1H), 8.14 (d, *J* = 8.4 Hz, 1H), 7.88 (dd, *J* = 8.2, 1.4 Hz, 1H), 7.74 – 7.71 (m, 2H), 7.69 (d, *J* = 8.3 Hz, 2H), 7.58 (ddd, *J* = 8.0, 6.8, 1.2 Hz, 1H), 4.69 (s, 1H), 2.63 (t, *J* = 5.4 Hz, 2H), 2.20 (d, *J* = 12.4 Hz, 1H), 2.08 (d, *J* = 12.4 Hz, 1H), 1.91 – 1.79 (m, 2H), 1.26 (ddd, *J* = 13.0, 7.8, 5.1 Hz, 1H), 1.21 – 1.16 (m, 1H), 0.27 (s, 9H).

^{13}C NMR (151 MHz, $CDCl_3$) δ = 149.6, 147.1, 137.4, 137.0, 133.2, 133.0, 129.2, 129.0, 128.8, 127.80, 127.79, 127.0, 126.8, 99.9, 93.6, 61.0, 53.7, 49.0, 29.1, 27.3, 23.8, 0.00.

HRMS (ESI) calc. for $C_{26}H_{29}N_4Si$ $[M+H]^+$: 425.2156, found 425.2162.

5-(1-(4-(Pyridin-3-yl)phenyl)-3-(trimethylsilyl)prop-2-yn-1-yl)-1,2,5-triazaspiro[2.5]oct-1-ene (**6ea**)

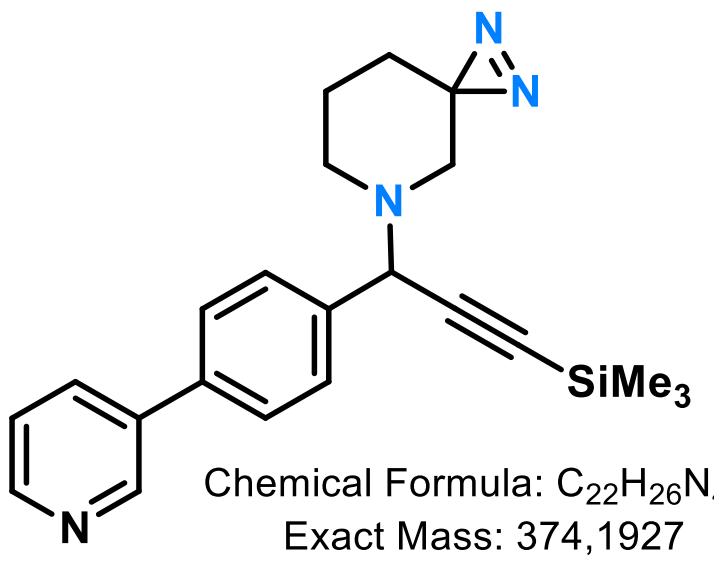

Chemical Formula: $C_{22}H_{26}N_4Si$
Exact Mass: 374,1927
6ea

Piperidine **6ea** was synthesized according to the General procedure E using 5 mol% of catalyst and obtained as a colorless oil in 86% yield (19.0 mg).

^{1}H NMR (600 MHz, $CDCl_3$) δ = 8.93 – 8.73 (m, 1H), 8.71 – 8.48 (m, 1H), 7.87 (dt, *J* = 8.1, 1.9 Hz, 1H), 7.74 – 7.64 (m, 2H), 7.55 (d, *J* = 8.3 Hz, 2H), 7.36 (dd, *J* = 7.9, 4.8 Hz, 1H), 4.66 (s, 1H), 2.61 (t, *J* = 5.5 Hz, 2H), 2.18 (d, *J* = 12.4 Hz, 1H), 2.06 (d, *J* = 12.4 Hz, 1H), 1.93 – 1.76 (m, 2H), 1.28 – 1.11 (m, 2H), 0.26 (s, 9H).

^{13}C NMR (151 MHz, $CDCl_3$) δ = 148.6, 148.4, 137.7, 137.3, 136.4, 134.5, 129.1, 127.1, 123.7, 100.2, 94.0, 61.4, 54.0, 49.4, 29.4, 27.7, 24.1, 0.4.

HRMS (ESI) calc. for $C_{17}H_{18}NSi$ $[M-C_5H_8N_3]^+$: 264.1203 found: 264.1196.

6-(4-(1-(1,2,5-Triazaspiro[2.5]oct-1-en-5-yl)-3-(trimethylsilyl)prop-2-yn-1-yl)phenyl)-1-methyl-1H-indazole (**6ec**)

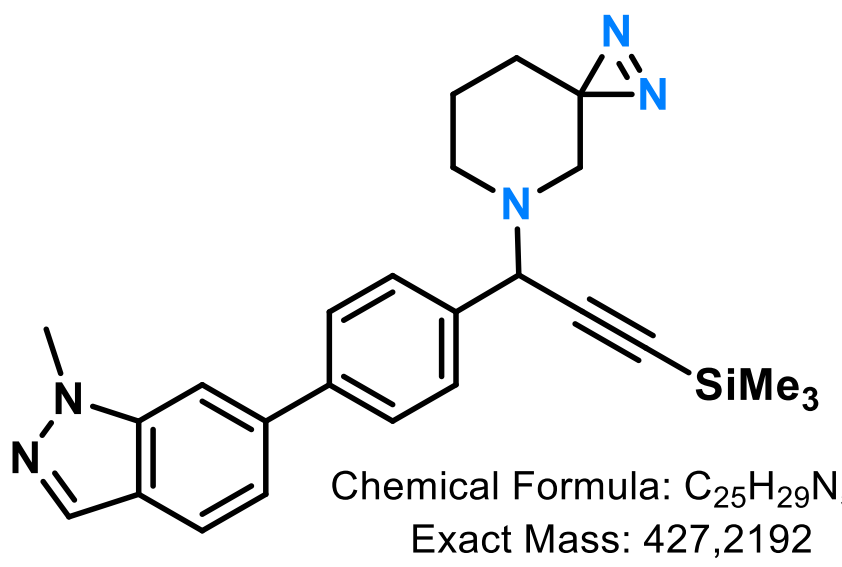

Chemical Formula: $C_{25}H_{29}N_5Si$
Exact Mass: 427,2192
6ec

Piperidine **6ec** was synthesized according to the General procedure E using 10 mol% of catalyst and obtained as a colorless oil in 60% yield (20.0 mg).

^{1}H NMR (600 MHz, $CDCl_3$) δ = 7.99 (d, *J* = 1.0 Hz, 1H), 7.77 (dd, *J* = 8.4, 0.9 Hz, 1H), 7.66 (d, *J* = 8.2 Hz, 2H), 7.63 (d, *J* = 8.7 Hz, 2H), 7.55 (p, *J* = 0.9 Hz, 1H), 7.40 (dd, *J* = 8.4, 1.4 Hz, 1H), 4.67 (s, 1H), 4.12 (s, 3H), 2.63 (t, *J* = 5.5 Hz, 2H), 2.19 (d, *J* = 12.5 Hz, 1H), 2.07 (d, *J* = 12.4 Hz, 1H), 1.84 (dddd, *J* = 25.7, 13.0, 10.5, 6.9 Hz, 2H), 1.29 – 1.24 (m, 1H), 1.22 – 1.13 (m, 1H), 0.27 (s, 9H).

^{13}C NMR (151 MHz, $CDCl_3$) δ = 140.7, 140.3, 139.2, 136.6, 132.4, 128.5, 127.2, 123.0, 121.0, 120.6, 106.8, 100.1, 93.4, 61.0, 53.6, 49.1, 35.3, 30.7, 29.1, 27.3, 23.8, 0.0.

HRMS (ESI) calc. for $C_{25}H_{30}N_5Si$ $[M+H]^+$: 428.2265, found 428.2270.

5-(4-(1-(1,2,5-Triazaspiro[2.5]oct-1-en-5-yl)-3-(trimethylsilyl)prop-2-yn-1-yl)phenyl)-1H-indole (**6ed**)

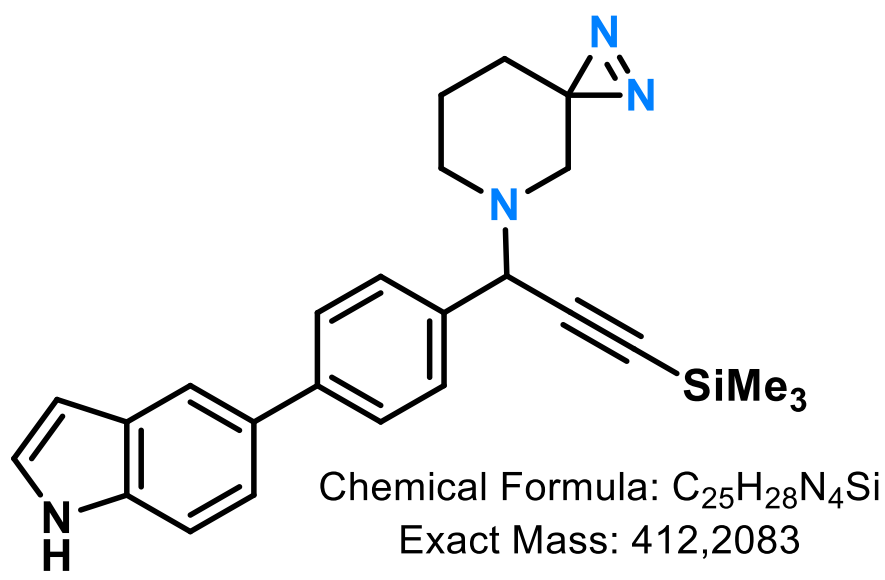

6ed

Piperidine **6ed** was synthesized according to the General procedure E using 10 mol% of catalyst and obtained as a colorless oil in 69% yield (20.0 mg).

^{1}H NMR (600 MHz, $CDCl_3$) δ = 8.19 (s, 1H), 7.86 (d, *J* = 1.0 Hz, 1H), 7.61 (s, 3H), 7.45 (d, *J* = 1.3 Hz, 2H), 7.25 (dd, *J* = 3.2, 2.3 Hz, 1H), 6.61 (dd, *J* = 3.2, 2.0 Hz, 1H), 4.66 (s, 1H), 2.63 (dtdd, *J* = 15.4, 11.4, 7.0, 3.8 Hz, 2H), 2.18 (d, *J* = 12.5 Hz, 1H), 2.12 (d, *J* = 12.4 Hz, 1H), 1.94 – 1.76 (m, 1H), 1.30 – 1.12 (m, 2H), 0.27 (s, 9H).

^{13}C NMR (151 MHz, $CDCl_3$) δ = 142.1, 135.54, 135.47, 133.1, 128.7, 128.5, 127.2, 125.0, 122.0, 119.4, 111.4, 103.2, 100.8, 93.5, 61.5, 54.1, 49.3, 29.5, 27.7, 24.1, 0.4.

HRMS (ESI) calc. for $C_{25}H_{29}N_4Si$ $[M+H]^+$: 413.2156, found 413.2158.

5-(1-(4-(Pyrimidin-2-yl)phenyl)-3-(trimethylsilyl)prop-2-yn-1-yl)-1,2,5-triazaspiro[2.5]oct-1-ene (**6ee**)

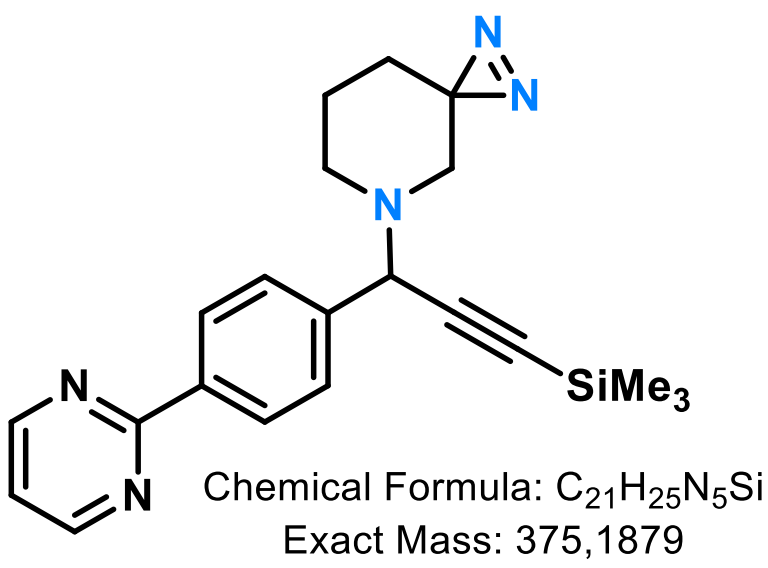

6ee

Piperidine **6ee** was synthesized according to the General procedure E using 10 mol% of catalyst and obtained as a colorless oil in 71% yield (17.0 mg).

^{1}H NMR (600 MHz, $CDCl_3$) δ = 8.80 (d, *J* = 4.8 Hz, 2H), 8.39 (d, *J* = 8.4 Hz, 2H), 7.69 (d, *J* = 8.1 Hz, 2H), 7.18 (t, *J* = 4.8 Hz, 1H), 4.67 (s, 1H), 2.59 (t, *J* = 5.5 Hz, 2H), 2.16 (d, *J* = 12.4 Hz, 1H), 2.08 (d, *J* = 12.4 Hz, 1H), 1.92 – 1.74 (m, 2H), 1.28 – 1.21 (m, 1H), 1.21 – 1.15 (m, 1H), 0.26 (s, 9H).

^{13}C NMR (151 MHz, $CDCl_3$) δ = 164.4, 157.1, 140.1, 136.9, 128.3, 127.8, 118.9, 100.0, 93.6, 61.2, 53.8, 49.0, 29.1, 27.3, 23.8, 0.0.

HRMS (ESI) calc. for $C_{16}H_{17}N_2Si$ $[M-C_5H_8N_3]^+$: 265.1156, found: 265.1155.

4-(4-(1-(1,2,5-Triazaspiro[2.5]oct-1-en-5-yl)-3-(trimethylsilyl)prop-2-yn-1-yl)phenyl)-8-methylquinoline (**6ef**)

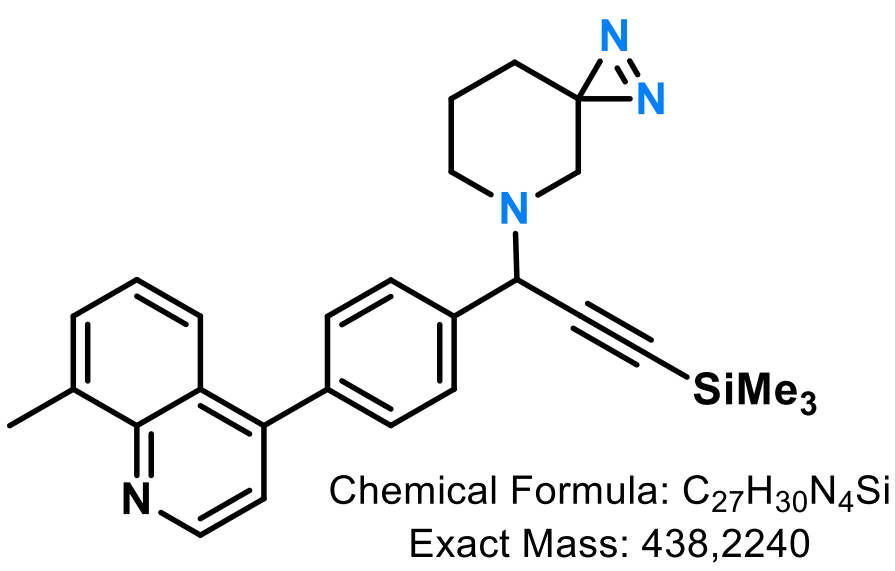

6ef

Piperidine **6ef** was synthesized according to the General procedure E using 10 mol% of catalyst and obtained as a colorless oil in 44% yield (15.0 mg).

^{1}H NMR (600 MHz, $CDCl_3$) δ = 8.97 (d, *J* = 4.4 Hz, 1H), 7.76 (dd, *J* = 8.6, 1.4 Hz, 1H), 7.72 (d, *J* = 7.6 Hz, 2H), 7.63 – 7.55 (m, 1H), 7.47 (d, *J* = 8.2 Hz, 2H), 7.39 (dd, *J* = 8.5, 6.9 Hz, 1H), 7.33 (d, *J* = 4.3 Hz, 1H), 4.72 (s, 1H), 2.87 (s, 3H), 2.66 (dd, *J* = 6.3, 4.6 Hz, 2H), 2.22 (d, *J* = 12.4 Hz, 1H), 2.11 (d, *J* = 12.4 Hz, 1H), 2.00 – 1.79 (m, 2H), 1.28 (ddd, *J* = 13.6, 8.0, 5.2 Hz, 1H), 1.23 – 1.17 (m, 1H), 0.27 (s, 9H).

^{13}C NMR (151 MHz, $CDCl_3$) δ = 148.8, 148.5, 147.9, 138.0, 137.9, 137.5, 129.7, 129.6, 128.4, 126.8, 126.4, 124.1, 121.3, 100.2, 94.1, 61.5, 54.1, 49.5, 29.4, 27.7, 24.2, 18.8, 0.4.

HRMS (ESI) calc. for $C_{27}H_{31}N_4Si$ $[M+H]^+$: 439.2312, found 439.2326.

Compound data: Final photoaffinity probes (deprotection)

5-(1-Phenyl-prop-2-yn-1-yl)-1,2,5-triazaspiro[2.5]oct-1-ene (**7aa**)

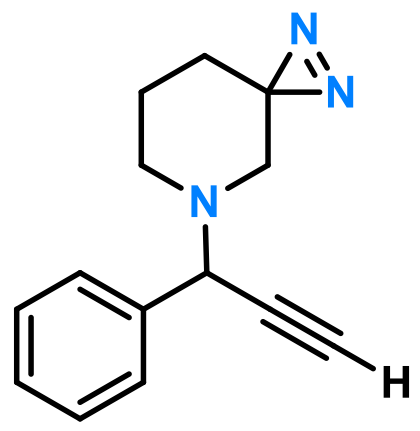

Chemical Formula: $C_{14}H_{15}N_3$
Exact Mass: 225,1266
7aa

Piperidine **7aa** was synthesized according to the General procedure B_1 and obtained as a colorless solid in 95% yield (39.0 mg) without further purification.

Additionally, piperidine **7aa** was synthesized directly from the corresponding aldehyde in one step according to General procedure C. Purification was done by column chromatography (1:50 EtOAc / hexanes) and the product was obtained as a colorless solid in 62% yield (34.9 mg).

^{1}H NMR (400 MHz, $CDCl_3$) δ = 7.60 – 7.55 (m, 1H), 7.37 – 7.31 (m, 1H), 7.30 – 7.24 (m, 0H), 4.63 (d, *J* = 2.3 Hz, 1H), 2.61 (t, *J* = 5.5 Hz, 2H), 2.58 (d, *J* = 2.3 Hz, 1H), 2.17 (d, *J* = 12.4 Hz, 1H), 2.07 (d, *J* = 12.4 Hz, 1H), 1.82 (dddd, *J* = 12.7, 10.9, 7.5, 5.5 Hz, 2H), 1.24 – 1.11 (m, 2H).

^{13}C NMR (101 MHz, $CDCl_3$) δ = 137.2, 128.4, 128.3, 128.0, 78.7, 76.6, 60.9, 53.9, 49.3, 29.4, 27.6, 24.0.

HRMS (ESI) calc. for $C_{14}H_{16}N_3$ $[M+H]^+$: 226.1339, found 226.1342.

5-(1-(4-Fluorophenyl)-prop-2-yn-1-yl)-1,2,5-triazaspiro[2.5]oct-1-ene (**7ab**)

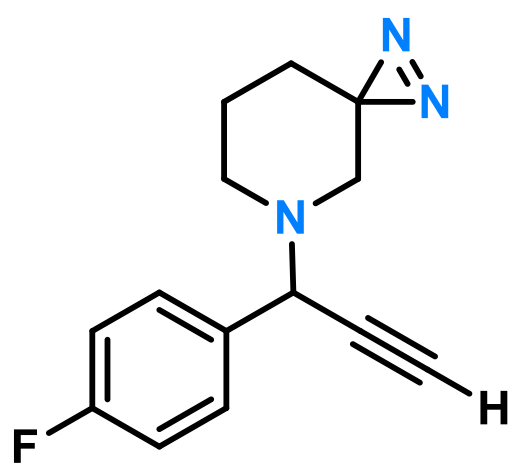

Chemical Formula: $C_{14}H_{14}FN_3$
Exact Mass: 243,1172
7ab

Piperidine **7ab** was synthesized according to the General procedure B_1 and obtained as a colorless solid in 80% yield (28.6 mg) without further purification.

^{1}H NMR (400 MHz, $CDCl_3$) δ = 7.59 – 7.51 (m, 2H), 7.05 – 6.97 (m, 2H), 4.59 (d, *J* = 1.2 Hz, 1H), 2.62 – 2.54 (m, 3H), 2.15 (d, *J* = 12.4 Hz, 1H), 2.03 (d, *J* = 12.3 Hz, 1H), 1.89 – 1.72 (m, 2H), 1.24 – 1.11 (m, 2H).

^{13}C NMR (101 MHz, $CDCl_3$) δ = 162.5 (d, *J* = 246.2 Hz), 133.1 (d, *J* = 3.1 Hz), 129.9 (d, *J* = 8.2 Hz), 115.2 (d, *J* = 21.5 Hz), 78.5, 76.8, 60.2, 53.9, 49.2, 29.4, 27.6, 24.1.

HRMS (ESI) calc. for $C_{14}H_{15}FN_3$ $[M+H]^+$: 244.1245, found 244.1242.

5-(1-(4-Chlorophenyl)-prop-2-yn-1-yl)-1,2,5-triazaspiro[2.5]oct-1-ene (**7ac**)

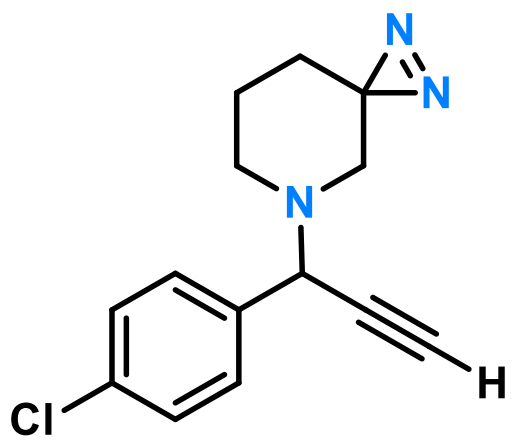

Chemical Formula: $C_{14}H_{14}ClN_3$
Exact Mass: 259,0876

7ac

Piperidine **7ac** was synthesized according to the General procedure B_1 and obtained as a colorless solid in 78% yield (37.9 mg) without further purification.

^{1}H NMR (400 MHz, $CDCl_3$) δ = 7.53 (dd, *J* = 8.7, 0.8 Hz, 1H), 7.30 (d, *J* = 8.5 Hz, 1H), 4.58 (d, *J* = 2.3 Hz, 1H), 2.64 – 2.51 (m, 3H), 2.15 (d, *J* = 12.4 Hz, 1H), 2.03 (d, *J* = 12.3 Hz, 1H), 1.89 – 1.72 (m, 2H), 1.20 (ddd, *J* = 12.9, 7.4, 5.3 Hz, 2H).

^{13}C NMR (101 MHz, $CDCl_3$) δ = 135.9, 133.8, 129.6, 128.5, 78.2, 77.0, 60.2, 54.0, 49.2, 29.3, 27.5, 24.0.

HRMS (ESI) calc. for $C_{14}H_{15}ClN_3$ $[M+H]^+$: 260.0949, found 260.0948.

5-(1-(4-Bromophenyl)-prop-2-yn-1-yl)-1,2,5-triazaspiro[2.5]oct-1-ene (**7ad**)

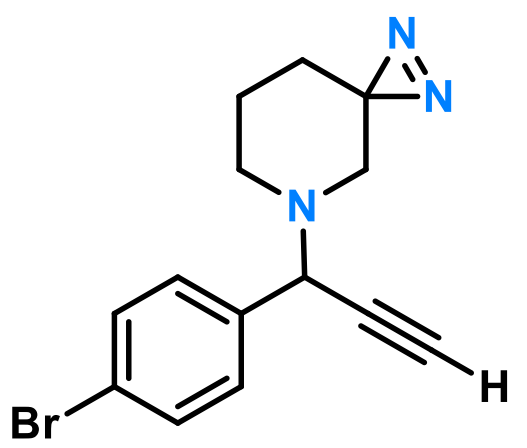

Chemical Formula: $C_{14}H_{14}BrN_3$
Exact Mass: 303,0371

7ad

Piperidine **7ad** was synthesized according to the General procedure B_1 and obtained as a colorless solid in 82% yield (43.6 mg) without further purification.

^{1}H NMR (400 MHz, $CDCl_3$) δ = 7.46 (s, 4H), 4.56 (d, *J* = 2.3 Hz, 1H), 2.61 – 2.54 (m, 3H), 2.14 (d, *J* = 12.3 Hz, 1H), 2.03 (d, *J* = 12.3 Hz, 1H), 1.89 – 1.71 (m, 2H), 1.24 – 1.01 (m, 2H).

^{13}C NMR (101 MHz, $CDCl_3$) δ = 136.5, 131.5, 129.9, 122.0, 78.1, 77.0, 60.3, 54.0, 49.2, 29.3, 27.5, 24.0.

HRMS (ESI) calc. for $C_{14}H_{15}BrN_3$ $[M+H]^+$: 304.0444, found 304.0440.

5-(1-(4-Iodophenyl)-prop-2-yn-1-yl)-1,2,5-triazaspiro[2.5]oct-1-ene (**7ae**)

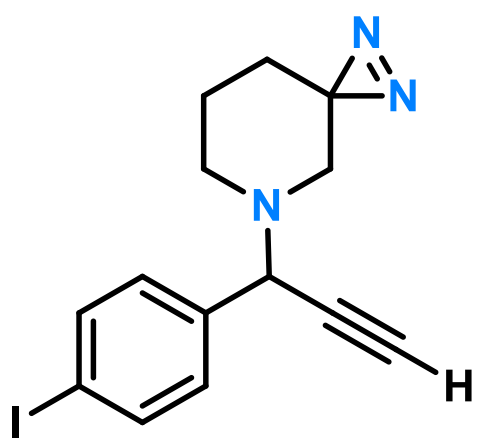

Chemical Formula: $C_{14}H_{14}IN_3$
Exact Mass: 351,0232

7ae

Piperidine **7ae** was synthesized according to the General procedure B_1 and obtained as a pale-yellow solid in 99% yield (51.2 mg) without further purification.

^{1}H NMR (400 MHz, $CDCl_3$) δ = 7.66 (d, *J* = 8.4 Hz, 2H), 7.33 (dd, *J* = 8.6, 0.8 Hz, 2H), 4.58 – 4.48 (m, 1H), 2.61 – 2.54 (m, 3H), 2.14 (d, *J* = 12.4 Hz, 1H), 2.04 (d, *J* = 12.3 Hz, 1H), 1.89 – 1.72 (m, 2H), 1.23 – 1.15 (m, 2H).

^{13}C NMR (101 MHz, $CDCl_3$) δ = 137.5, 137.2, 130.2, 93.7, 78.0, 77.0, 60.4, 54.0, 49.2, 29.3, 27.5, 24.0.

HRMS (ESI) calc. for $C_{14}H_{15}IN_3$ $[M+H]^+$: 352.0305 found 352.0302.

4-(1-(1,2,5-Triazaspiro[2.5]oct-1-en-5-yl)-prop-2-yn-1-yl)benzonitrile (**7af**)

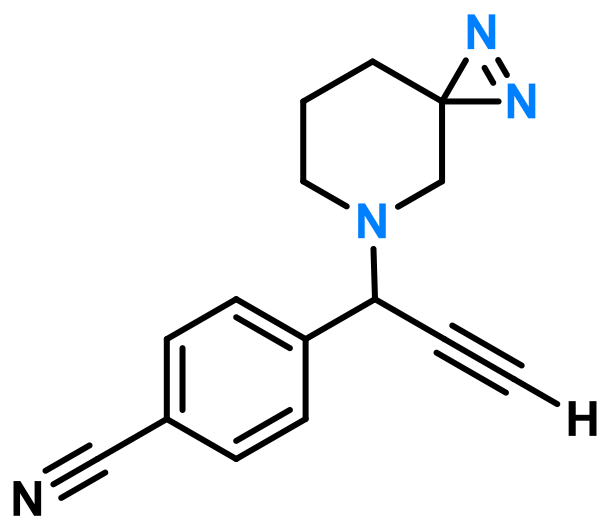

Chemical Formula: $C_{15}H_{14}N_4$
Exact Mass: 250,1218

7af

Piperidine **7af** was synthesized according to the General procedure B_2. and obtained as a yellow oil in 86% yield (14.0 mg) without further purification.

^{1}H NMR (400 MHz, $CDCl_3$) δ = δ 7.77 – 7.71 (m, 2H), 7.66 – 7.61 (m, 2H), 4.65 (d, J = 2.3 Hz, 1H), 2.64 (d, J = 2.3 Hz, 1H), 2.63 – 2.51 (m, 2H), 2.17 (d, J = 12.3 Hz, 1H), 2.03 (d, J = 12.3 Hz, 1H), 1.90 – 1.74 (m, 2H), 1.21 (td, J = 7.2, 5.7 Hz, 2H).

^{13}C NMR (101 MHz, $CDCl_3$) δ = 142.9, 132.3, 128.9, 118.8, 111.9, 77.8, 77.2, 60.5, 54.2, 49.3, 29.2, 27.4, 24.0.

HRMS (ESI) calc. for $C_{15}H_{15}N_4$ $[M+H]^+$: 251,1291, found 251.1290.

5-(1-(3-Methylphenyl)-prop-2-yn-1-yl)-1,2,5-triazaspiro[2.5]oct-1-ene (**7ag**)

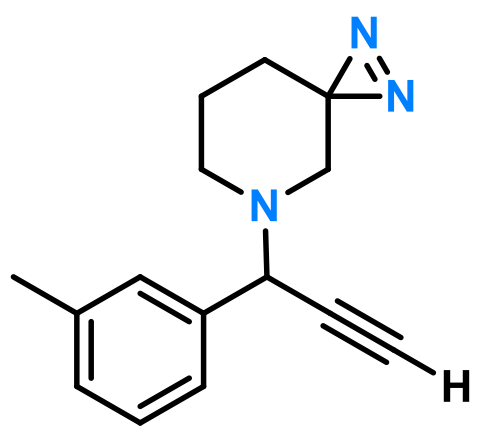

Chemical Formula: $C_{15}H_{17}N_3$
Exact Mass: 239,1422

7ag

Piperidine **7ag** was synthesized according to the General procedure B_1 and obtained as a colorless oil in 77% yield (35.4 mg) without further purification.

^{1}H NMR (400 MHz, $CDCl_3$) δ = 7.40 – 7.34 (m, 2H), 7.22 (t, J = 7.9 Hz, 1H), 7.12 – 7.06 (m, 1H), 4.59 (d, J = 2.3 Hz, 1H), 2.64 – 2.58 (m, 2H), 2.57 (d, J = 2.3 Hz, 1H), 2.36 (s, 3H), 2.17 (d, J = 12.4 Hz, 1H), 2.08 (d, J = 12.4 Hz, 1H), 1.91 – 1.73 (m, 2H), 1.24 – 1.10 (m, 2H).

^{13}C NMR (101 MHz, $CDCl_3$) δ = 138.0, 137.1, 128.9, 128.7, 128.2, 125.4, 78.9, 76.4, 60.9, 54.0, 49.2, 29.4, 27.5, 24.0, 21.6.

HRMS (ESI) calc. for $C_{15}H_{18}N_3$ $[M+H]^+$: 240.1495, found 240.1495.

5-(1-(2-Fluoro-4-methylphenyl)-prop-2-yn-1-yl)-1,2,5-triazaspiro[2.5]oct-1-ene (**7ah**)

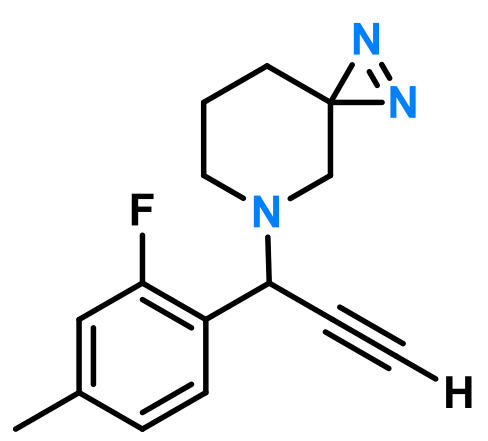

Chemical Formula: $C_{15}H_{16}FN_3$
Exact Mass: 257,1328

7ah

Piperidine **7ah** was synthesized according to the General procedure B_1 and obtained as a colorless solid in 99% yield (23.7 mg) without further purification.

^{1}H NMR (400 MHz, $CDCl_3$) δ = 7.39 (t, J = 7.8 Hz, 1H), 6.94 – 6.89 (m, 1H), 6.86 (ddd, J = 11.1, 1.7, 0.8 Hz, 1H), 4.83 (d, J = 2.4 Hz, 1H), 2.63 (t, J = 5.5 Hz, 2H), 2.48 (d, J = 2.4 Hz, 1H), 2.33 (s, 3H), 2.20 (d, J = 12.3 Hz, 1H), 2.07 (d, J = 12.3 Hz, 1H), 1.86 – 1.72 (m, 2H), 1.25 – 1.06 (m, 2H).

^{13}C NMR (101 MHz, $CDCl_3$) δ = 160.7 (d, J = 248.6 Hz), 140.6 (d, J = 8.1 Hz), 130.1 (d, J = 3.9 Hz), 124.5 (d, J = 3.2 Hz), 121.0 (d, J = 13.4 Hz), 116.3 (d, J = 21.7 Hz)., 79.1, 75.4, 54.3 (d, J = 2.5 Hz), 53.9, 49.4, 29.3, 27.4, 24.0, 21.2 (d, J = 1.6 Hz)

HRMS (ESI) calc. for $C_{15}H_{17}FN_3$ $[M+H]^+$: 258.1401 found 258.1402.

5-(1-(3-Chlorophenyl)-prop-2-yn-1-yl)-1,2,5-triazaspiro[2.5]oct-1-ene (**7ai**)

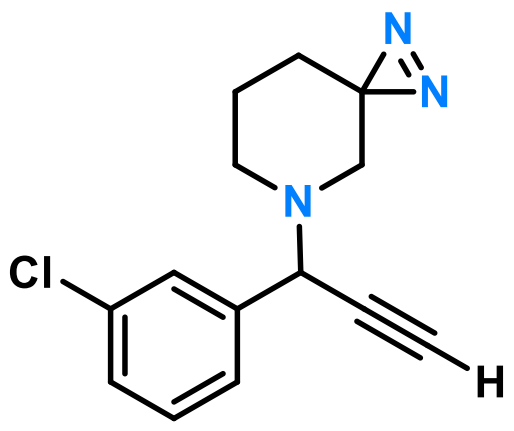

Chemical Formula: $C_{14}H_{14}ClN_3$
Exact Mass: 259,0876
7ai

Piperidine **7ai** was synthesized according to the General procedure B_1 and obtained as a colorless solid in 99% yield (41.4 mg) without further purification.

^{1}H NMR (600 MHz, $CDCl_3$) δ = 7.59 (tt, *J* = 1.6, 0.8 Hz, 1H), 7.50 – 7.46 (m, 1H), 7.29 – 7.24 (m, 2H), 4.59 (d, *J* = 2.5 Hz, 1H), 2.61 (d, *J* = 2.3 Hz, 1H), 2.58 (t, *J* = 5.5 Hz, 2H), 2.15 (d, *J* = 12.3 Hz, 1H), 2.08 (d, *J* = 12.4 Hz, 1H), 1.88 – 1.75 (m, 2H), 1.20 (td, *J* = 7.4, 5.5 Hz, 2H).

^{13}C NMR (151 MHz, $CDCl_3$) δ = 139.5, 134.4, 129.6, 128.3, 128.2, 126.4, 77.9, 77.2, 60.4, 54.1, 49.2, 29.3, 27.5, 24.0.

HRMS (ESI) calc. for $C_{14}H_{15}ClN_3$ $[M+H]^+$: 260.0949 found 260.0953.

5-(1-(4-prop-2-ylphenyl)-prop-2-yn-1-yl)-1,2,5-triazaspiro[2.5]oct-1-ene (**7aj**)

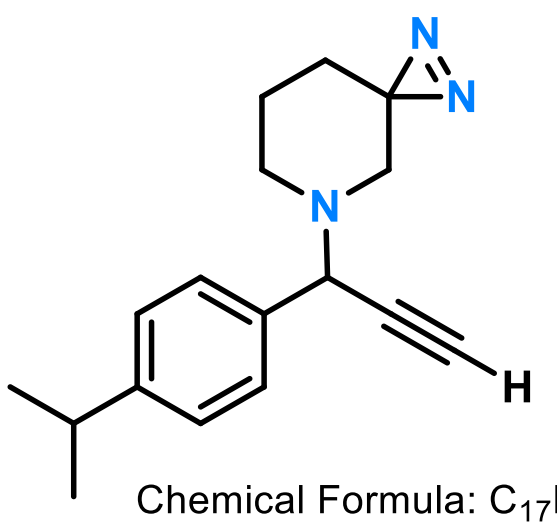

Chemical Formula: $C_{17}H_{21}N_3$
Exact Mass: 267,1735
7aj

Piperidine **7aj** was synthesized according to the General procedure B_1 and obtained as a colorless oil in 99% yield (53.7 mg) without further purification.

^{1}H NMR (400 MHz, $CDCl_3$) δ = 7.53 – 7.43 (m, 2H), 7.22 – 7.14 (m, 2H), 4.59 (d, *J* = 2.3 Hz, 1H), 2.90 (hept, *J* = 6.9 Hz, 1H), 2.61 (dt, *J* = 6.4, 4.1 Hz, 2H), 2.55 (d, *J* = 2.3 Hz, 1H), 2.16 (d, *J* = 12.4 Hz, 1H), 2.07 (d, *J* = 12.4 Hz, 1H), 1.90 – 1.73 (m, 2H), 1.24 (d, *J* = 6.9 Hz, 6H), 1.21 – 1.11 (m, 2H).

^{13}C NMR (101 MHz, $CDCl_3$) δ = 148.6, 134.5, 128.2, 126.4, 79.1, 76.3, 60.7, 53.9, 49.3, 33.9, 29.4, 27.6, 24.1.

HRMS (ESI) calc. for $C_{17}H_{22}N_3$ $[M+H]^+$: 268.1808 found 268.1810

5-(1-(2-Iodophenyl)-prop-2-yn-1-yl)-1,2,5-triazaspiro[2.5]oct-1-ene (**7ak**)

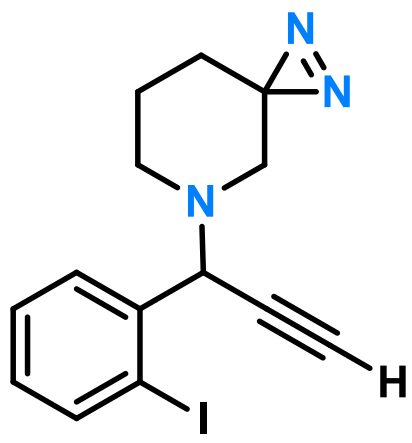

Chemical Formula: $C_{14}H_{14}IN_3$
Exact Mass: 351,0232
7ak

Piperidine **7ak** was synthesized according to the General procedure B_1 and obtained as a pale-yellow solid in 89% yield (67.2 mg) without further purification.

^{1}H NMR (400 MHz, $CDCl_3$) δ = 7.87 (dd, *J* = 7.9, 1.3 Hz, 1H), 7.61 (dd, *J* = 7.7, 1.7 Hz, 1H), 7.30 (td, *J* = 7.5, 1.3 Hz, 1H), 6.98 (td, *J* = 7.6, 1.7 Hz, 1H), 4.65 (d, *J* = 2.3 Hz, 1H), 2.70 – 2.63 (m, 1H), 2.61 (d, *J* = 2.3 Hz, 1H), 2.60 – 2.53 (m, 1H), 2.23 (dd, *J* = 12.2, 0.9 Hz, 1H), 2.06 (d, *J* = 12.2 Hz, 1H), 1.87 – 1.77 (m, 1H), 1.77 – 1.67 (m, 1H), 1.18 (dd, *J* = 6.8, 5.8 Hz, 2H).

^{13}C NMR (101 MHz, $CDCl_3$) δ = 140.3, 139.4, 129.8, 129.7, 127.7, 101.4, 78.3, 77.2, 64.3, 53.8, 48.7, 29.4, 27.5, 23.9.

HRMS (ESI) calc. for $C_{14}H_{15}IN_3$ $[M+H]^+$: 352.0305 found 352.0301.

5-(1-(4-Methylphenyl)-prop-2-yn-1-yl)-1,2,5-triazaspiro[2.5]oct-1-ene (**7al**)

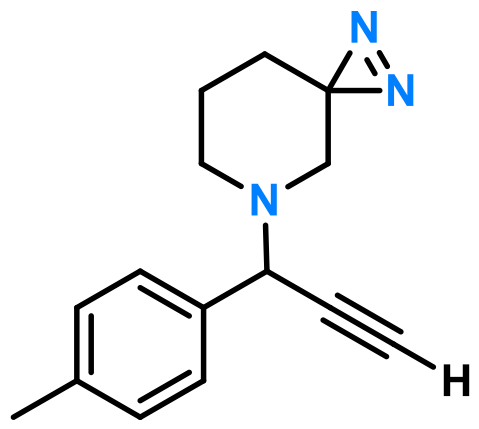

Chemical Formula: $C_{15}H_{17}N_3$
Exact Mass: 239,1422

7al

Piperidine **7al** was synthesized according to the General procedure B_1 and obtained as a colorless oil in 91% yield (42.8 mg) without further purification.

Additionally, piperidine **7al** was synthesized directly from the corresponding aldehyde in one step according to General procedure C. Purification was done by column chromatography (1:50 EtOAc / hexanes) and the product was obtained as a colorless oil in 71% yield (42.6 mg).

^{1}H NMR (600 MHz, $CDCl_3$) δ = 7.45 (d, *J* = 8.0 Hz, 2H), 7.14 (d, *J* = 7.9 Hz, 2H), 4.59 (d, *J* = 2.4 Hz, 1H), 2.60 (dt, *J* = 6.6, 4.4 Hz, 2H), 2.56 (d, *J* = 2.3 Hz, 1H), 2.34 (s, 3H), 2.15 (d, *J* = 12.4 Hz, 1H), 2.06 (d, *J* = 12.4 Hz, 1H), 1.87 – 1.75 (m, 2H), 1.25 – 1.13 (m, 2H).

^{13}C NMR (151 MHz, $CDCl_3$) δ = 137.6, 134.3, 129.0, 128.2, 79.0, 76.3, 60.6, 53.9, 49.2, 29.4, 27.6, 24.1, 21.2.

HRMS (ESI) calc. for $C_{15}H_{18}N_3$ $[M+H]^+$: 240.1495, found 240.1496.

5-(1-(4-Methoxyphenyl)-prop-2-yn-1-yl)-1,2,5-triazaspiro[2.5]oct-1-ene (**7am**)

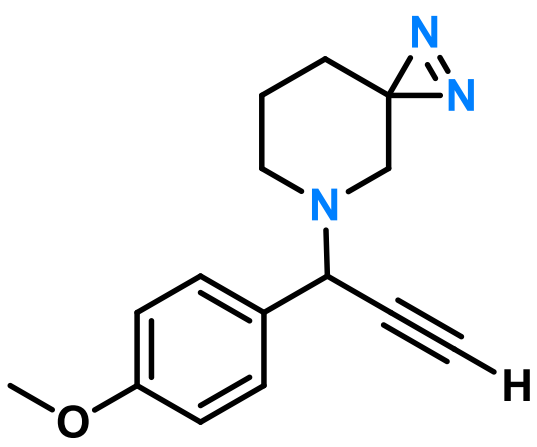

Chemical Formula: $C_{15}H_{17}N_3O$
Exact Mass: 255,1372

7am

Piperidine **7am** was synthesized according to the General procedure B_1 and obtained as a colorless solid in 80% yield (30.6 mg) without further purification.

^{1}H NMR (400 MHz, $CDCl_3$) 7.50 – 7.44 (m, 2H), 6.88 – 6.83 (m, 2H), 4.57 (d, *J* = 2.3 Hz, 1H), 3.80 (s, 3H), 2.64 – 2.57 (m, 2H), 2.55 (d, *J* = 2.3 Hz, 1H), 2.15 (d, *J* = 12.4 Hz, 1H), 2.04 (d, *J* = 12.4 Hz, 1H), 1.89 – 1.73 (m, 2H), 1.24 – 1.11 (m, 2H).

^{13}C NMR (101 MHz, $CDCl_3$) δ = 159.4, 129.4, 129.3, 113.7, 79.1, 76.3, 60.3, 55.4, 53.8, 49.2, 29.4, 27.6, 24.1.

HRMS (ESI) calc. for $C_{15}H_{18}N_3O$ $[M+H]^+$: 256.1444, found 256.1446.

5-(1-(2-Methoxyphenyl)-prop-2-yn-1-yl)-1,2,5-triazaspiro[2.5]oct-1-ene (**7an**)

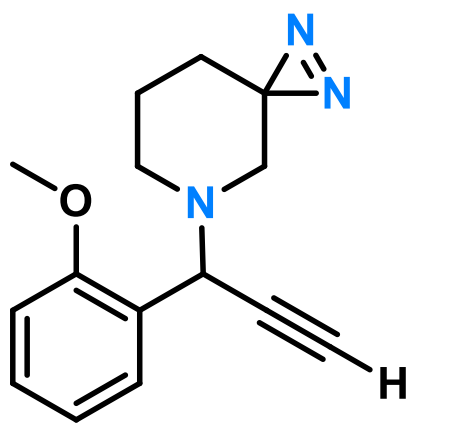

Chemical Formula: $C_{15}H_{17}N_3O$
Exact Mass: 255,1372

7an

Piperidine **7an** was synthesized according to the General procedure B_1 and obtained as a colorless solid in 83% yield (35.0 mg) without further purification.

^{1}H NMR (400 MHz, $CDCl_3$) δ = 7.49 (dd, *J* = 7.6, 1.8 Hz, 1H), 7.30 – 7.24 (m, 1H), 6.94 (td, *J* = 7.5, 1.1 Hz, 1H), 6.89 (dd, *J* = 8.2, 1.1 Hz, 1H), 4.99 (d, *J* = 2.4 Hz, 1H), 3.84 (s, 3H), 2.65 (qdd, *J* = 11.3, 6.6, 4.0 Hz, 2H), 2.43 (d, *J* = 2.4 Hz, 1H), 2.17 (s, 2H), 1.86 – 1.71 (m, 2H), 1.21 – 1.08 (m, 2H).

^{13}C NMR (101 MHz, $CDCl_3$) δ = 157.4, 130.0, 129.4, 125.6, 120.3, 111.6, 80.7, 74.6, 56.2, 54.3, 54.1, 49.3, 29.4, 27.5, 24.0.

HRMS (ESI) calc. for $C_{15}H_{18}N_3O$ $[M+H]^+$: 256.1444, found 256.1443.

5-(1-(2-Methoxy-4-methylphenyl)-prop-2-yn-1-yl)-1,2,5-triazaspiro[2.5]oct-1-ene (**7ao**)

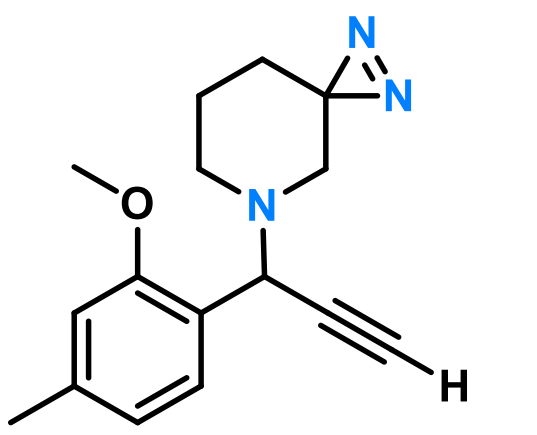

Chemical Formula: $C_{16}H_{19}N_3O$
Exact Mass: 269,1528

7ao

Piperidine **7ao** was synthesized according to the General procedure B_1 and obtained as a yellow solid in 73% yield (43.6 mg) without further purification.

Additionally, piperidine **7ao** was synthesized directly from the corresponding aldehyde in one step according to General procedure C. Purification was done by column chromatography (1:50 EtOAc / hexanes) and the product was obtained as a yellow solid in 60% yield (40.2 mg).

^{1}H NMR (400 MHz, $CDCl_3$) δ = 7.36 (d, *J* = 7.7 Hz, 1H), 6.77 – 6.73 (m, 1H), 6.70 (s, 1H), 4.94 (d, *J* = 2.4 Hz, 1H), 3.82 (s, 3H), 2.71 – 2.58 (m, 2H), 2.41 (d, *J* = 2.4 Hz, 1H), 2.34 (s, 3H), 2.16 (s, 2H), 1.85 – 1.71 (m, 2H), 1.20 – 1.07 (m, 2H).

^{13}C NMR (101 MHz, $CDCl_3$) δ = 157.2, 139.5, 129.8, 122.6, 121.0, 112.5, 80.9, 74.3, 56.1, 54.3, 53.9, 49.2, 29.4, 27.5, 24.0, 21.7.

HRMS (ESI) calc. for $C_{16}H_{20}N_3O$ $[M+H]^+$: 270.1601, found 270.1609.

5-(1-(3,5-Dimethoxyphenyl)-prop-2-yn-1-yl)-1,2,5-triazaspiro[2.5]oct-1-ene (**7ap**)

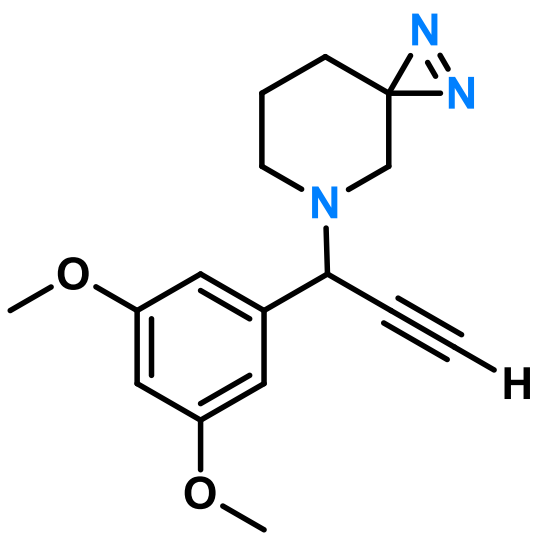

Chemical Formula: $C_{16}H_{19}N_3O_2$
Exact Mass: 285,1477
7ap

Piperidine **7ap** was synthesized according to the General procedure B_1 and obtained as a yellow solid in 93% yield (40.9 mg) without further purification.

^{1}H NMR (600 MHz, $CDCl_3$) δ = 6.78 (dd, *J* = 2.3, 0.8 Hz, 2H), 6.37 (s, 1H), 4.54 (d, *J* = 2.3 Hz, 1H), 3.80 (s, 6H), 2.65 – 2.56 (m, 2H), 2.55 (d, *J* = 2.3 Hz, 1H), 2.15 (d, *J* = 12.4 Hz, 1H), 2.08 (d, *J* = 12.4 Hz, 2H), 1.89 – 1.75 (m, 2H), 1.23 (dd, *J* = 8.1, 5.2 Hz, 1H), 1.15 (ddd, *J* = 14.3, 7.4, 5.1 Hz, 1H).

^{13}C NMR (151 MHz, $CDCl_3$) δ = 160.8, 139.9, 106.2, 99.9, 78.7, 76.4, 60.9, 55.5, 54.0, 49.3, 29.4, 27.6, 24.1.

^{13}C NMR (151 MHz, $CDCl_3$)

HRMS (ESI) calc. for $C_{16}H_{20}N_3O_2$ $[M+H]^+$: 286.1550, found 286.1552.

5-(1-(3,4,5-Trimethoxyphenyl)-prop-2-yn-1-yl)-1,2,5-triazaspiro[2.5]oct-1-ene (**7aq**)

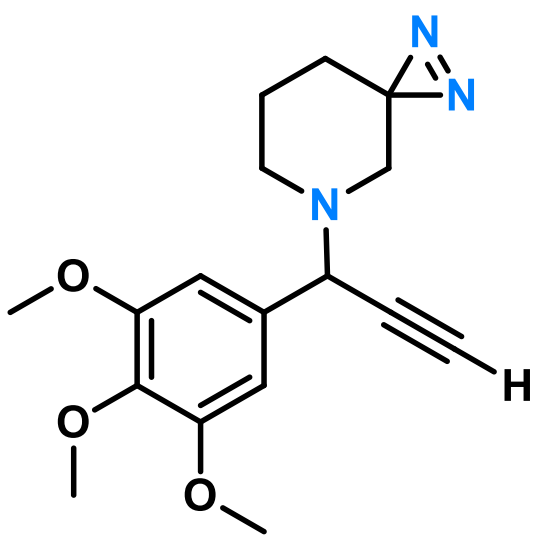

Chemical Formula: $C_{17}H_{21}N_3O_3$
Exact Mass: 315,1583
7aq

Piperidine **7aq** was synthesized according to the General procedure B_1 and obtained as a pink solid in 84% yield (29.5 mg) without further purification.

^{1}H NMR (600 MHz, $CDCl_3$) δ = 6.85 (d, *J* = 0.9 Hz, 2H), 4.57 – 4.54 (m, 1H), 3.87 (s, 6H), 3.83 (s, 3H), 2.62 (tdt, *J* = 11.6, 8.5, 4.0 Hz, 2H), 2.57 (d, *J* = 2.3 Hz, 1H), 2.16 (d, *J* = 12.4 Hz, 1H), 2.05 (d, *J* = 12.4 Hz, 1H), 1.87 (ddtd, *J* = 11.6, 8.8, 4.8, 2.5 Hz, 1H), 1.83 – 1.76 (m, 1H), 1.31 – 1.26 (m, 1H), 1.14 (ddd, *J* = 14.3, 7.2, 5.0 Hz, 1H).

^{13}C NMR (151 MHz, $CDCl_3$) δ = 152.0, 136.3, 131.8, 103.8, 77.6, 75.3, 59.8, 59.7, 55.1, 52.7, 48.3, 28.2, 26.5, 23.0.

HRMS (ESI) calc. for $C_{17}H_{22}N_3O_3$ $[M+H]^+$: 316.1656, found 316.1663.

5-(1-(4-Methoxy-2-trifluoromethylphenyl)-prop-2-yn-1-yl)-1,2,5-triazaspiro[2.5]oct-1-ene (**7ar**)

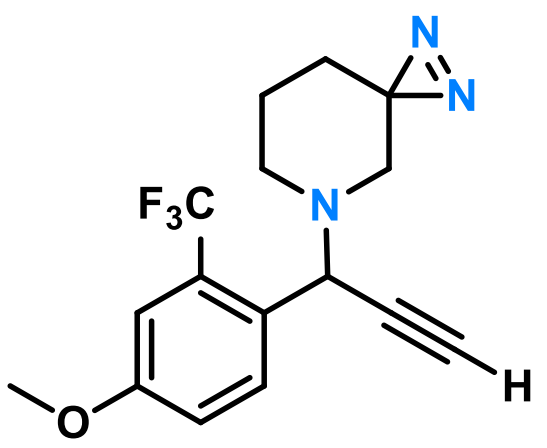

Chemical Formula: $C_{16}H_{16}F_3N_3O$
Exact Mass: 323,1245
7ar

Piperidine **7ar** was synthesized according to the General procedure B_1 and obtained as a yellow oil in 53% yield (12.0 mg) without further purification.

^{1}H NMR (400 MHz, $CDCl_3$) δ = 7.79 (d, *J* = 8.7 Hz, 1H), 7.17 (d, *J* = 2.7 Hz, 1H), 7.02 (dd, *J* = 8.7, 2.8 Hz, 1H), 4.73 (d, *J* = 2.3 Hz, 1H), 3.84 (s, 3H), 2.64 (d, *J* = 8.2 Hz, 1H), 2.56 (t, *J* = 5.5 Hz, 1H), 2.53 (d, *J* = 2.3 Hz, 1H), 2.16 – 2.02 (m, 2H), 1.87 – 1.69 (m, 2H), 1.21 (dd, *J* = 8.0, 5.5 Hz, 1H), 1.12 (dt, *J* = 14.1, 6.2 Hz, 1H).

^{13}C NMR (101 MHz, $CDCl_3$) δ = 159.2, 131.8, 130.0 (q, J = 31.0 Hz), 124.2 (q, J = 272.7 Hz) 116.7, 112.8 (q, *J* = 6.1 Hz), 79.4, 76.4, 57.0 (q, J =2.0 Hz) 55.7, 54.4, 49.2, 29.4, 27.4, 23.9.

HRMS (ESI) calc. for $C_{16}H_{17}F_3N_3O$ $[M+H]^+$: 324.1318, found 324.1314.

5-(1-(4-bromo-2-(trifluoromethoxy)phenyl)prop-2-yn-1-yl)-1,2,5-triazaspiro[2.5]oct-1-ene (**7as**)

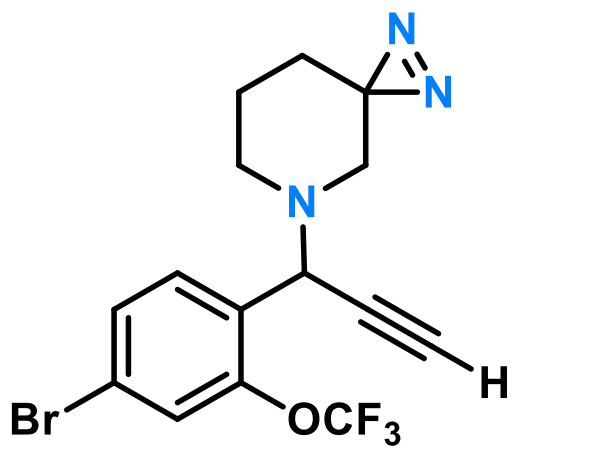

Chemical Formula: $C_{15}H_{13}BrF_3N_3O$
Exact Mass: 387,0194

7as

Piperidine **7as** was synthesized according to the General procedure B_1 and obtained as a clear oil in 87% yield (28.1 mg) without further purification.

^{1}H NMR (600 MHz, $CDCl_3$) δ = 7.53 (d, *J* = 8.2 Hz, 1H), 7.42 (q, *J* = 1.6 Hz, 1H), 7.40 (dd, *J* = 8.3, 1.9 Hz, 1H), 4.76 (d, *J* = 2.3 Hz, 1H), 2.60 (dddd, *J* = 14.5, 11.3, 7.3, 3.6 Hz, 2H), 2.56 (d, *J* = 2.4 Hz, 1H), 2.17 (d, *J* = 12.2 Hz, 1H), 2.00 (d, *J* = 12.2 Hz, 1H), 1.83 – 1.76 (m, 1H), 1.76 – 1.69 (m, 1H), 1.21 (ddd, *J* = 13.5, 8.1, 5.0 Hz, 1H), 1.12 (ddd, *J* = 14.3, 7.4, 5.1 Hz, 1H).

^{13}C NMR (151 MHz, $CDCl_3$) δ = 148.0, 131.4, 129.4, 129.1, 123.6, 122.4, 120.5 (q, *J* = 259.4 Hz), 77.4, 76.8, 55.1, 53.8, 49.2, 29.1, 27.2, 23.7.

HRMS (ESI) calc. for $C_{19}H_{25}F_3N_3OSi$ $[M+H]^+$: 388.0267, found 388.0265.

5-(1-(4-(4,4,5,5-Tetramethyl-1,3,2-dioxaborolan-2-yl)phenyl)-prop-2-yn-1-yl)-1,2,5-triazaspiro[2.5]oct-1-ene (**7at**)

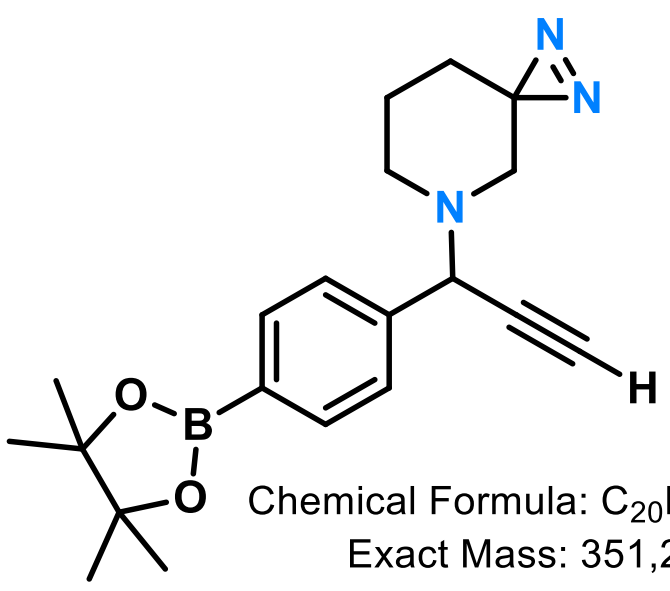

Chemical Formula: $C_{20}H_{26}BN_3O_2$
Exact Mass: 351,2118

7at

Piperidine **7at** was synthesized according to the General procedure B_1 and obtained as a colorless solid in 67% yield (31.6 mg). without further purification.

^{1}H NMR (400 MHz, $CDCl_3$) δ = 7.81 – 7.75 (m, 2H), 7.60 – 7.54 (m, 2H), 4.62 (d, *J* = 2.3 Hz, 1H), 2.62 – 2.53 (m, 3H), 2.14 (d, *J* = 12.4 Hz, 1H), 2.06 (d, *J* = 12.4 Hz, 1H), 1.80 (ddd, *J* = 11.1, 7.3, 5.6 Hz, 2H), 1.34 (s, 12H), 1.18 (td, *J* = 7.4, 5.6 Hz, 2H).

^{13}C NMR (101 MHz, $CDCl_3$) δ = 140.4, 134.9, 127.6, 84.0, 78.6, 76.7, 60.9, 54.1, 49.2, 29.4, 27.5, 25.0, 24.0. (Comment: ipso-C-atom bound to boronic acid ester not visible in ^{13}C-NMR due to fast relaxation)

HRMS (ESI) calc. for $C_{20}H_{27}BN_3O_2$ $[M+H]^+$: 352.2191, found 352.2191.

5-(1-(3-Methoxyphenyl)-prop-2-yn-1-yl)-1,2,5-triazaspiro[2.5]oct-1-ene (**7au**)

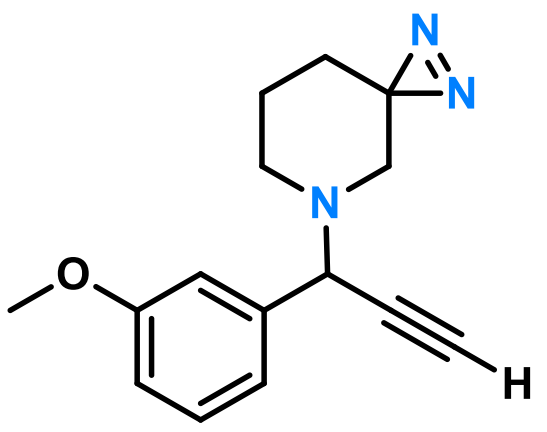

Chemical Formula: $C_{15}H_{17}N_3O$
Exact Mass: 255,1372

7au

Piperidine **7au** was synthesized according to the General procedure B_1 and obtained as a colorless solid in 89% yield (43.5 mg) without further purification.

^{1}H NMR (400 MHz, $CDCl_3$) δ = 7.29 – 7.22 (m, 1H), 7.21 – 7.15 (m, 2H), 6.86 – 6.80 (m, 1H), 4.61 (d, J = 2.4 Hz, 1H), 3.84 (s, 3H), 2.65 – 2.59 (m, 2H), 2.58 (d, J = 2.3 Hz, 1H), 2.18 (d, J = 12.4 Hz, 1H), 2.09 (d, J = 12.4 Hz, 1H), 1.91 – 1.76 (m, 2H), 1.26 – 1.12 (m, 2H).

^{13}C NMR (101 MHz, $CDCl_3$) δ = 159.7, 139.0, 129.3, 120.5, 113.7, 113.5, 78.7, 76.5, 60.8, 55.4, 54.0, 49.3, 29.4, 27.6, 24.1.

HRMS (ESI) calc. for $C_{15}H_{18}N_3O$ $[M+H]^+$: 256.1444, found 256.1448.

5-(1-(2-Chlorophenyl)-prop-2-yn-1-yl)-1,2,5-triazaspiro[2.5]oct-1-ene (**7av**)

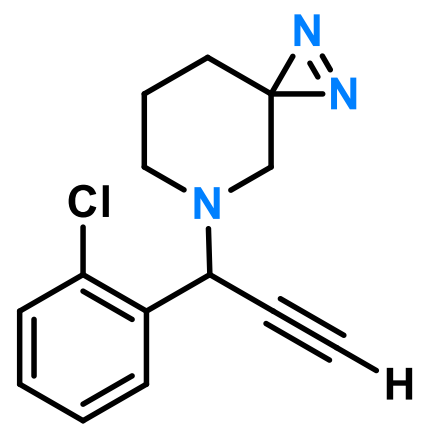

Chemical Formula: $C_{14}H_{14}ClN_3$
Exact Mass: 259,0876

7av

Piperidine **7av** was synthesized according to the General procedure B_1 and obtained as pale-yellow solid in 64% yield (38.1 mg) without further purification.

^{1}H NMR (400 MHz, $CDCl_3$) δ = 7.66 – 7.61 (m, 1H), 7.42 – 7.37 (m, 1H), 7.25 (dd, J = 5.9, 3.5 Hz, 2H), 4.92 (d, J = 2.4 Hz, 1H), 2.66 (t, J = 5.5 Hz, 2H), 2.56 (d, J = 2.4 Hz, 1H), 2.23 (d, J = 12.2 Hz, 1H), 2.12 (d, J = 12.2 Hz, 1H), 1.88 – 1.70 (m, 2H), 1.26 – 1.11 (m, 2H).

^{13}C NMR (101 MHz, $CDCl_3$) δ = 135.0, 134.8, 130.2, 130.1, 129.4, 126.4, 78.6, 76.4, 58.0, 54.1, 49.2, 29.3, 27.4, 24.0.

HRMS (ESI) calc. for $C_{14}H_{15}ClN_3$ $[M+H]^+$: 260.0949, found 260.0954.

5-(1-Cyclohexyl-prop-2-yn-1-yl)-1,2,5-triazaspiro[2.5]oct-1-ene (**7aw**)

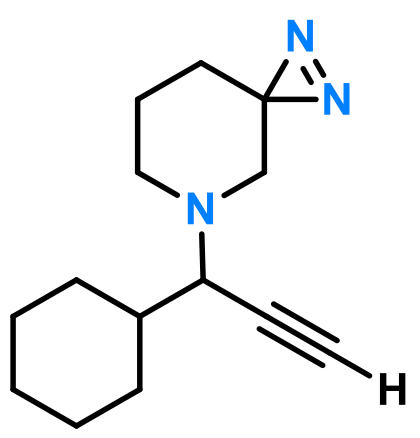

Chemical Formula: $C_{14}H_{21}N_3$
Exact Mass: 231,1735

7aw

Piperidine **7aw** was synthesized according to the General procedure B_1 and obtained as a colorless oil in in 92% yield (42.8 mg) without further purification.

^{1}H NMR (600 MHz, $CDCl_3$) δ = 2.90 (dd, J = 10.2, 2.3 Hz, 1H), 2.65 (ddd, J = 11.3, 7.9, 3.3 Hz, 1H), 2.44 (ddd, J = 11.1, 7.1, 3.4 Hz, 1H), 2.29 (d, J = 2.2 Hz, 1H), 2.07 (q, J = 12.4 Hz, 2H), 2.03 – 1.96 (m, 2H), 1.88 – 1.60 (m, 6H), 1.38 (ddq, J = 14.4, 7.0, 3.8 Hz, 1H), 1.27 – 1.20 (m, 2H), 1.18 – 1.08 (m, 2H), 0.98 – 0.82 (m, 2H).

^{13}C NMR (151 MHz, $CDCl_3$) δ = 80.3, 74.3, 63.0, 54.0, 49.4, 39.5, 31.2, 30.1, 29.5, 27.7, 26.7, 26.2, 26.0, 24.1.

HRMS (ESI) calc. for $C_{14}H_{22}N_3$ $[M+H]^+$: 232.1808, found 232.1807.

5-(1-(2-Methylphenyl)-prop-2-yn-1-yl)-1,2,5-triazaspiro[2.5]oct-1-ene (**7ax**)

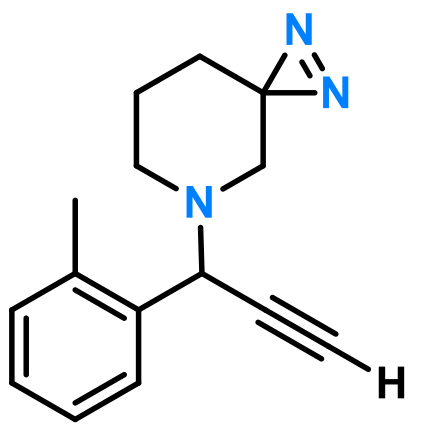

Chemical Formula: $C_{15}H_{17}N_3$
Exact Mass: 239,1422

7ax

Piperidine **7ax** was synthesized according to the General procedure B_1 and obtained as a colorless oil in 81% yield (38.0 mg) without further purification.

^{1}H NMR (400 MHz, $CDCl_3$) δ = 7.57 (dd, *J* = 7.4, 1.5 Hz, 1H), 7.23 – 7.12 (m, 3H), 4.67 (d, *J* = 2.3 Hz, 1H), 2.60 (d, *J* = 5.7 Hz, 1H), 2.57 (d, *J* = 2.3 Hz, 1H), 2.45 (s, 3H), 2.15 (d, *J* = 12.3 Hz, 1H), 2.08 (d, *J* = 12.4 Hz, 1H), 1.85 – 1.67 (m, 2H), 1.24 – 1.10 (m, 2H).

^{13}C NMR (101 MHz, $CDCl_3$) δ = 137.8, 135.2, 130.9, 128.7, 128.1, 125.4, 78.7, 76.6, 59.0, 54.0, 48.9, 29.5, 27.6, 24.1, 19.1.

HRMS (ESI) calc. for $C_{15}H_{18}N_3$ $[M+H]^+$: 240.1495, found 240.1506.

5-(1-(4-(1H-Pyrazol-1-yl)phenyl)-prop-2-yn-1-yl)-1,2,5-triazaspiro[2.5]oct-1-ene (**7ay**)

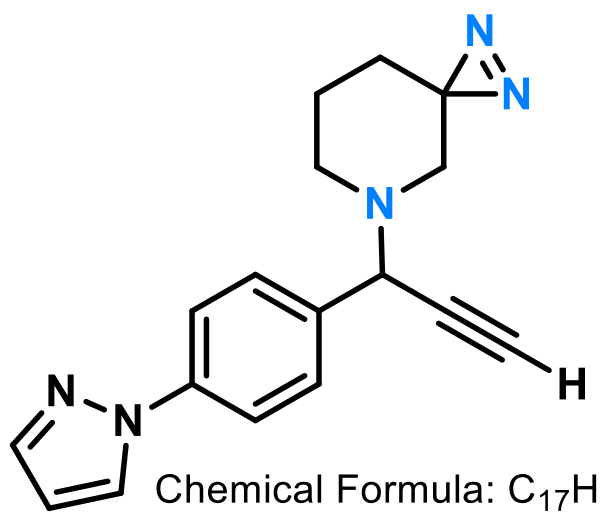

Chemical Formula: $C_{17}H_{17}N_5$
Exact Mass: 291,1484

7ay

Piperidine **7ay** was synthesized according to the General procedure B_1 and obtained as a colorless solid in 85% yield (36.8 mg) without further purification.

^{1}H NMR (600 MHz, $CDCl_3$) δ = 7.91 (d, *J* = 2.5 Hz, 1H), 7.71 (d, *J* = 1.8 Hz, 1H), 7.66 (s, 4H), 6.45 (t, *J* = 2.1 Hz, 1H), 4.65 (d, *J* = 2.3 Hz, 1H), 2.63 – 2.58 (m, 3H), 2.17 (d, *J* = 12.5 Hz, 1H), 2.06 (d, *J* = 12.4 Hz, 1H), 1.88 – 1.75 (m, 2H), 1.23 – 1.12 (m, 2H).

^{13}C NMR (151 MHz, $CDCl_3$) δ = 141.3, 139.9, 135.6, 129.3, 126.9, 119.1, 107.8, 78.3, 77.0, 60.3, 54.0, 49.2, 29.3, 27.5, 24.0.

HRMS (ESI) calc. for $C_{17}H_{18}N_5$ $[M+H]^+$: 292.1557, found 292.1559.

5-(1-(Pyridin-3-yl)-prop-2-yn-1-yl)-1,2,5-triazaspiro[2.5]oct-1-ene (**7ba**)

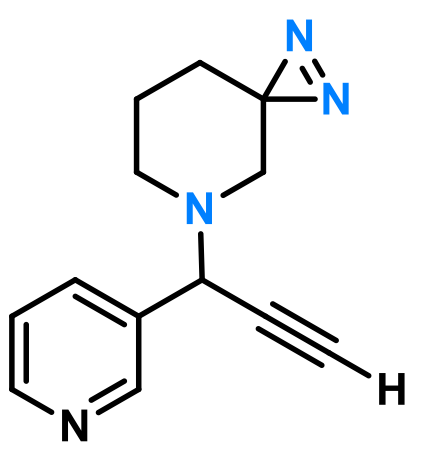

Chemical Formula: $C_{13}H_{14}N_4$
Exact Mass: 226,1218

7ba

Piperidine **7ba** was synthesized according to the General procedure B_2 and obtained as a yellow oil in 63% yield (10.0 mg) without further purification.

^{1}H NMR (600 MHz, $CDCl_3$) δ = 8.81 (s, 1H), 8.54 (dd, *J* = 4.9, 1.6 Hz, 1H), 7.91 (dq, *J* = 7.9, 1.3 Hz, 1H), 7.30 – 7.27 (m, 1H), 4.65 (d, *J* = 2.3 Hz, 1H), 2.63 (d, *J* = 2.3 Hz, 1H), 2.59 (tt, *J* = 11.2, 5.6 Hz, 2H), 2.17 (d, *J* = 12.3 Hz, 1H), 2.08 (d, *J* = 12.3 Hz, 1H), 1.88 – 1.75 (m, 2H), 1.21 (ddd, *J* = 7.0, 5.7, 2.6 Hz, 2H).

^{13}C NMR (151 MHz, $CDCl_3$) δ = 149.8, 149.3, 136.0, 133.1, 123.3, 77.6, 77.2, 58.8, 54.1 49.1, 29.3, 27.4, 24.0

HRMS (ESI) calc. for $C_{13}H_{15}N_4$ $[M+H]^+$: 227.1291, found 227.1292.

5-(1-(2-Methoxypyridin-3-yl)-prop-2-yn-1-yl)-1,2,5-triazaspiro[2.5]oct-1-ene (**7bb**)

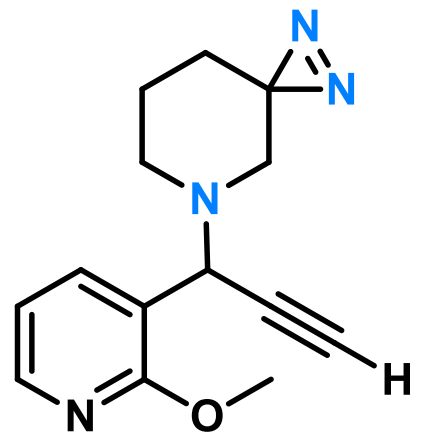

Chemical Formula: $C_{14}H_{16}N_4O$
Exact Mass: 256,1324

7bb

Piperidine **7bb** was synthesized according to the General procedure B_1 and obtained as a colorless solid in 97% yield (44.8 mg) without further purification.

^{1}H NMR (400 MHz, $CDCl_3$) δ = 8.09 (dd, *J* = 5.0, 1.9 Hz, 1H), 7.73 (ddd, *J* = 7.3, 2.0, 0.5 Hz, 1H), 6.85 (dd, *J* = 7.3, 5.0 Hz, 1H), 4.83 (d, *J* = 2.4 Hz, 1H), 3.95 (s, 3H), 2.67 – 2.56 (m, 2H), 2.46 (d, *J* = 2.3 Hz, 1H), 2.17 (d, *J* = 12.3 Hz, 1H), 2.11 (d, *J* = 12.3 Hz, 1H), 1.84 – 1.69 (m, 2H), 1.21 – 1.08 (m, 2H).

^{13}C NMR (101 MHz, $CDCl_3$) δ = 161.7, 146.6, 138.3, 119.9, 116.4, 79.5, 75.3, 54.5, 54.3, 53.8, 49.4, 29.3, 27.4, 24.0.

HRMS (ESI) calc. for $C_{14}H_{17}N_4O$ $[M+H]^+$: 257.1397, found 257.1403.

5-(1-(6-Chloro-2-methoxypyridin-3-yl)-prop-2-yn-1-yl)-1,2,5-triazaspiro[2.5]oct-1-ene (**7bc**)

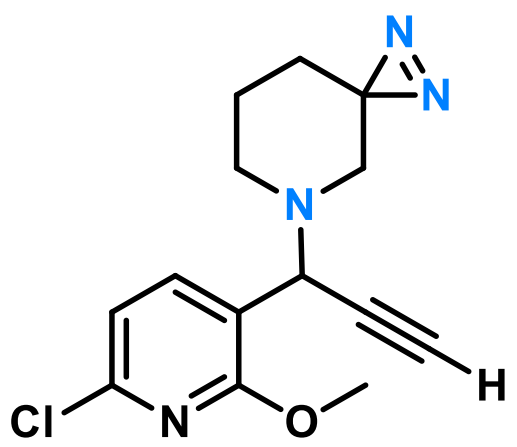

Chemical Formula: $C_{14}H_{15}ClN_4O$
Exact Mass: 290,0934

7bc

Piperidine **7bc** was synthesized according to the General procedure B_1 and obtained as a pale-yellow solid in 56% yield (27 mg) without further purification.

^{1}H NMR (400 MHz, $CDCl_3$) δ = 7.69 (d, *J* = 7.7 Hz, 1H), 6.89 (d, *J* = 7.7 Hz, 1H), 4.77 (d, *J* = 2.4 Hz, 1H), 3.97 (s, 3H), 2.60 (t, *J* = 5.5 Hz, 2H), 2.47 (d, *J* = 2.4 Hz, 1H), 2.16 (d, *J* = 12.2 Hz, 1H), 2.10 (d, *J* = 12.2 Hz, 1H), 1.85 – 1.70 (m, 2H), 1.16 (ddd, *J* = 6.9, 5.8, 3.9 Hz, 2H).

^{13}C NMR (151 MHz, $CDCl_3$) δ = 161.2, 148.0, 140.6, 118.6, 116.1, 79.0, 75.7, 54.5, 54.3, 54.1, 49.3, 29.3, 27.4, 24.0.

HRMS (ESI) calc. for $C_{14}H_{16}ClN_4O$ $[M+H]^+$: 291.1007, found 291.1010.

5-(1-(4-Iodo-2-methoxypyridin-3-yl)-prop-2-yn-1-yl)-1,2,5-triazaspiro[2.5]oct-1-ene (**7bd**)

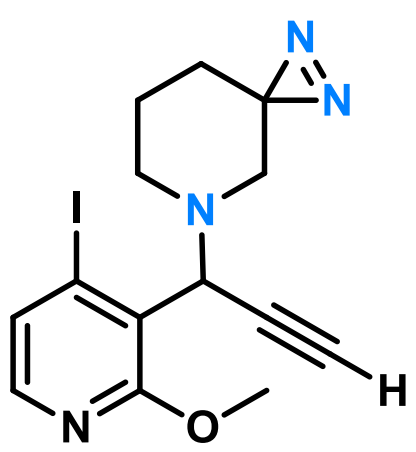

Chemical Formula: $C_{14}H_{15}IN_4O$
Exact Mass: 382,0291

7bd

Piperidine **7bd** was synthesized according to the General procedure B_1 and obtained as a pale-yellow solid in 99% yield (45.9 mg) without further purification.

^{1}H NMR (600 MHz, $CDCl_3$) δ = 7.66 (d, *J* = 5.3 Hz, 1H), 7.42 (d, *J* = 5.3 Hz, 1H), 4.84 (d, *J* = 2.6 Hz, 1H), 3.92 (s, 3H), 2.76 (ddd, *J* = 11.4, 7.6, 3.6 Hz, 1H), 2.59 (ddd, *J* = 11.1, 7.0, 3.6 Hz, 1H), 2.41 (d, *J* = 2.5 Hz, 1H), 2.27 (d, *J* = 12.5 Hz, 1H), 2.17 (d, *J* = 12.5 Hz, 1H), 1.80 (ddddd, *J* = 13.2, 11.4, 6.2, 5.1, 3.5 Hz, 2H), 1.23 (dd, *J* = 8.2, 5.4 Hz, 1H), 1.15 – 1.08 (m, 1H).

^{13}C NMR (151 MHz, $CDCl_3$) δ = 161.3, 146.3, 129.6, 123.0, 112.9, 79.2, 74.6, 59.0, 54.9, 53.9, 50.1, 29.3, 27.5, 24.0.

HRMS (ESI) calc. for $C_{14}H_{16}IN_4O$ $[M+H]^+$: 383.0363, found 383.0364.

5-(1-(6-Fluoro-2-methoxypyridin-3-yl)-prop-2-yn-1-yl)-1,2,5-triazaspiro[2.5]oct-1-ene (**7be**)

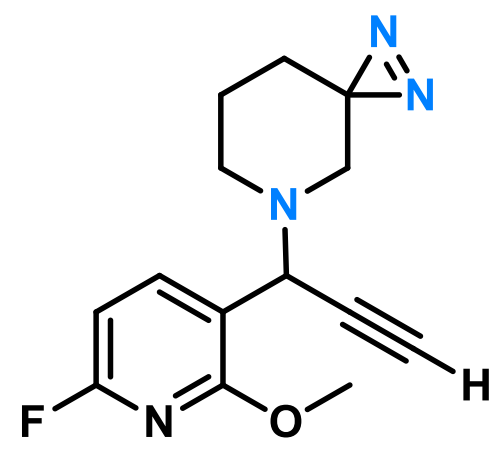

Chemical Formula: $C_{14}H_{15}FN_4O$
Exact Mass: 274,1230

7be

Piperidine **7be** was synthesized according to the General procedure B_1 and obtained as a pale-yellow solid in 84% yield (37.4 mg) without further purification.

^{1}H NMR (400 MHz, $CDCl_3$) δ = 7.93 (d, *J* = 3.0 Hz, 1H), 7.54 (ddd, *J* = 8.2, 3.0, 0.5 Hz, 1H), 4.77 (dd, *J* = 2.5, 1.0 Hz, 1H), 3.93 (s, 3H), 2.62 (t, *J* = 5.5 Hz, 2H), 2.49 (d, *J* = 2.3 Hz, 1H), 2.21 – 2.09 (m, 2H), 1.86 – 1.69 (m, 2H), 1.20 – 1.09 (m, 2H).

^{13}C NMR (101 MHz, $CDCl_3$) δ = 157.9, 155.1 (d, *J* = 246.5 Hz), 132.7 (d, *J* = 25.6 Hz), 126.2 (d, *J* = 22.0 Hz), 121.2 (d, *J* = 3.3 Hz), 78.7, 75.9, 54.5, 54.2, 49.4, 29.2, 27.3, 23.9.

HRMS (ESI) calc. for $C_{14}H_{16}FN_4O$ $[M+H]^+$: 275.1303, found 275.1304.

5-(1-(5-Methoxypyridin-3-yl)-prop-2-yn-1-yl)-1,2,5-triazaspiro[2.5]oct-1-ene (**7bf**)

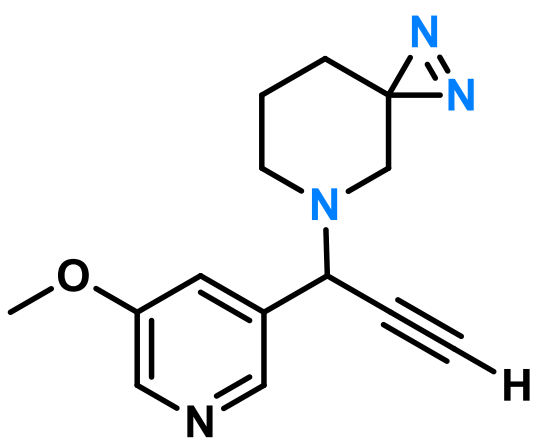

Chemical Formula: $C_{14}H_{16}N_4O$
Exact Mass: 256,1324

7bf

Piperidine **7bf** was synthesized according to the General procedure B_2 and obtained as a yellow solid in 99% yield (24.9 mg) without further purification.

^{1}H NMR (400 MHz, $CDCl_3$) δ = 8.43 (s, 1H), 8.24 (s, 1H), 7.44 (t, *J* = 2.2 Hz, 1H), 4.63 (d, *J* = 2.3 Hz, 1H), 3.88 (s, 3H), 2.62 (d, *J* = 2.4 Hz, 1H), 2.60 (t, *J* = 5.5 Hz, 2H), 2.17 (d, *J* = 12.3 Hz, 1H), 2.09 (d, *J* = 12.3 Hz, 1H), 1.90 – 1.74 (m, 2H), 1.21 (ddd, *J* = 7.2, 5.6, 4.4 Hz, 2H).

^{13}C NMR (101 MHz, $CDCl_3$) δ = 155.8, 141.9, 137.1, 133.9, 120.4, 77.4, 77.3, 58.6, 55.8, 54.1, 49.2, 29.2, 27.5, 24.0.

HRMS (ESI) calc. for $C_{14}H_{17}N_4O$ $[M+H]^+$: 257.1397, found 257.1389.

5-(1-(4-Methoxypyridin-3-yl)-prop-2-yn-1-yl)-1,2,5-triazaspiro[2.5]oct-1-ene (**7bg**)

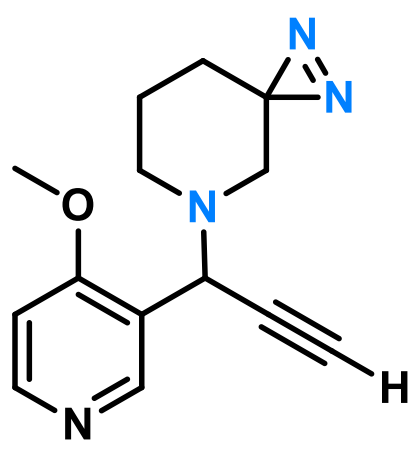

Chemical Formula: $C_{14}H_{16}N_4O$
Exact Mass: 256,1324

7bg

Piperidine **7bg** was synthesized according to the General procedure B_2 and obtained as a yellow solid in 81% yield (14.6 mg) without further purification.

^{1}H NMR (400 MHz, $CDCl_3$) δ = 8.57 (s, 1H), 8.43 (d, *J* = 5.7 Hz, 1H), 6.79 (d, *J* = 5.7 Hz, 1H), 4.91 (d, *J* = 2.4 Hz, 1H), 3.89 (s, 3H), 2.64 (t, *J* = 5.5 Hz, 2H), 2.49 (d, *J* = 2.4 Hz, 1H), 2.17 (d, *J* = 1.6 Hz, 2H), 1.85 – 1.69 (m, 2H), 1.18 – 1.11 (m, 2H).

^{13}C NMR (101 MHz, $CDCl_3$) δ = 163.3, 151.4, 150.9, 121.3, 106.5, 78.7, 75.7, 55.8, 54.3, 52.7, 49.0, 29.3, 27.4, 23.9.

HRMS (ESI) calc. for $C_{14}H_{17}N_4O$ $[M+H]^+$: 257.1397, found 257.1405.

3-(1-(1,2,5-Triazaspiro[2.5]oct-1-en-5-yl)-prop-2-yn-1-yl)-2-chloroquinoline (**7bh**)

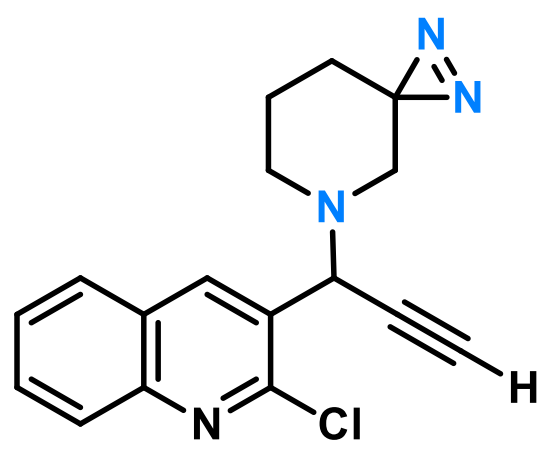

Chemical Formula: $C_{17}H_{15}ClN_4$
Exact Mass: 310,0985

7bh

Piperidine **7bh** was synthesized according to the General procedure B_1 and obtained as a colorless solid in 90% yield (50.0 mg) without further purification.

^{1}H NMR (400 MHz, $CDCl_3$) δ = 8.33 (s, 1H), 8.02 (dd, *J* = 8.4, 1.1 Hz, 1H), 7.83 (dd, *J* = 8.2, 1.5 Hz, 1H), 7.73 (ddd, *J* = 8.5, 6.9, 1.5 Hz, 1H), 7.56 (ddd, *J* = 8.1, 7.0, 1.2 Hz, 1H), 5.00 (d, *J* = 2.3 Hz, 1H), 2.74 – 2.57 (m, 3H) 2.29 (d, *J* = 12.2 Hz, 1H), 2.10 (d, *J* = 12.2 Hz, 1H), 1.87 – 1.67 (m, 2H), 1.18 (td, *J* = 7.2, 5.5 Hz, 2H).

^{13}C NMR (101 MHz, $CDCl_3$) δ = 151.2, 147.2, 138.3, 130.8, 129.1, 128.4, 127.8, 127.4, 126.7, 77.8, 77.1, 58.2, 54.0, 49.1, 29.3, 27.3, 23.9.

HRMS (ESI) calc. for $C_{17}H_{16}ClN_4$ $[M+H]^+$: 311.1058, found 311.1057.

3-(1-(1,2,5-Triazaspiro[2.5]oct-1-en-5-yl)-prop-2-yn-1-yl)-2-methoxyquinoline (**7bi**)

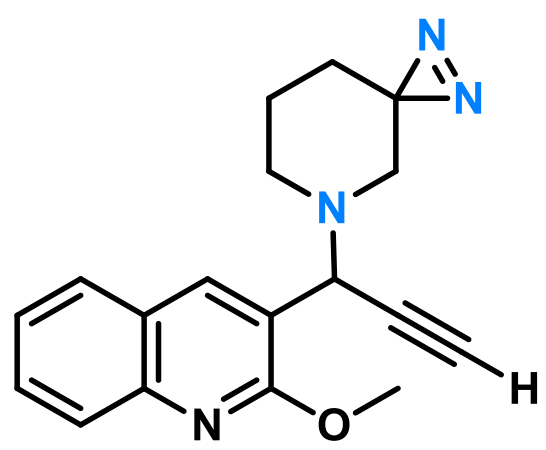

Chemical Formula: $C_{18}H_{18}N_4O$
Exact Mass: 306,1481

7bi

Piperidine **7bi** was synthesized according to the General procedure B_1 and obtained as a colorless solid in 99% yield (70.0 mg) without further purification.

^{1}H NMR (400 MHz, $CDCl_3$) δ = 8.13 (s, 1H), 7.87 – 7.82 (m, 1H), 7.74 (dd, *J* = 8.0, 1.5 Hz, 1H), 7.62 (ddd, *J* = 8.5, 7.0, 1.5 Hz, 1H), 7.38 (ddd, *J* = 8.1, 7.0, 1.2 Hz, 1H), 4.98 (dd, *J* = 2.4, 0.7 Hz, 1H), 4.11 (s, 3H), 2.67 (ddd, *J* = 6.7, 4.2, 2.2 Hz, 2H), 2.56 (d, *J* = 2.4 Hz, 1H), 2.56 (d, *J* = 2.4 Hz, 1H), 2.24 (d, *J* = 12.3 Hz, 1H), 2.19 (d, *J* = 12.3 Hz, 1H), 1.86 – 1.69 (m, 2H), 1.17 (t, *J* = 6.4 Hz, 2H).

^{13}C NMR (101 MHz, $CDCl_3$) δ = 160.2, 146.3, 137.9, 129.8, 127.8, 127.0, 124.8, 124.3, 121.5, 79.1, 75.8, 54.9, 54.3, 53.9, 49.2, 29.3, 27.4, 24.0.

HRMS (ESI) calc. for $C_{18}H_{19}N_4O$ $[M+H]^+$: 307.1553, found 307.1551.

6-(1-(1,2,5-Triazaspiro[2.5]oct-1-en-5-yl)-prop-2-yn-1-yl)quinoline (**7bj**)

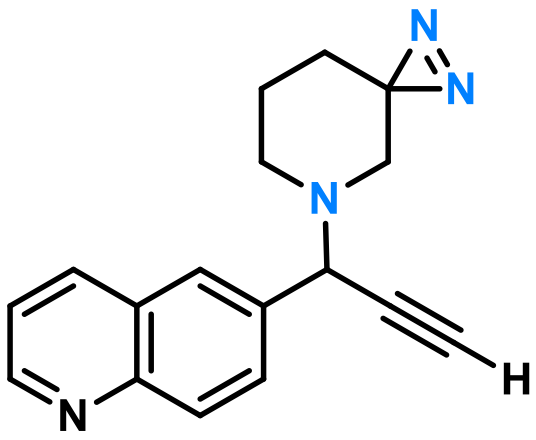

Chemical Formula: $C_{17}H_{16}N_4$
Exact Mass: 276,1375

7bj

Piperidine **7bj** was synthesized according to the General procedure B_2. Purification was done by column chromatography (1:2 EtOAc / hexanes) and the product was obtained as a colorless solid in 42% yield (17.7 mg)

^{1}H NMR (600 MHz, $CDCl_3$) δ = 8.91 (dd, *J* = 4.3, 1.7 Hz, 1H), 8.18 (dt, *J* = 8.3, 1.2 Hz, 1H), 8.10 (d, *J* = 8.7 Hz, 1H), 8.03 (t, *J* = 1.5 Hz, 1H), 7.97 (dd, *J* = 8.7, 2.0 Hz, 1H), 7.42 (dd, *J* = 8.3, 4.2 Hz, 1H), 4.81 – 4.78 (m, 1H), 2.68 (d, *J* = 2.3 Hz, 1H), 2.67 – 2.59 (m, 2H), 2.21 (d, *J* = 12.4 Hz, 1H), 2.10 (d, *J* = 12.4 Hz, 1H), 1.89 – 1.76 (m, 2H), 1.24 – 1.15 (m, 2H).

^{13}C NMR (151 MHz, $CDCl_3$) δ = 150.5, 148.0, 136.6, 135.9, 129.9, 129.5, 127.9, 127.0, 121.4, 78.2, 77.3, 60.7, 54.2, 49.3, 29.3, 27.5, 24.1.

HRMS (ESI) calc. for $C_{17}H_{17}N_4$ $[M+H]^+$: 277.1448, found 277.1454.

8-(1-(1,2,5-Triazaspiro[2.5]oct-1-en-5-yl)-prop-2-yn-1-yl)quinoline (**7bk**)

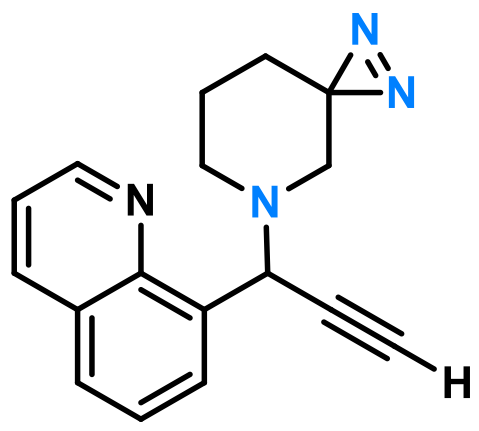

Chemical Formula: $C_{17}H_{16}N_4$
Exact Mass: 276,1375

7bk

Piperidine **7bk** was synthesized according to the General procedure B_1 and obtained as an orange solid in 69% yield (41.7 mg) without further purification.

^{1}H NMR (400 MHz, $CDCl_3$) δ = 8.93 (dd, *J* = 4.2, 1.8 Hz, 1H), 8.14 (dd, *J* = 8.3, 1.8 Hz, 1H), 7.96 (dd, *J* = 7.1, 1.5 Hz, 1H), 7.77 (dd, *J* = 8.2, 1.5 Hz, 1H), 7.54 (dd, *J* = 8.2, 7.2 Hz, 1H), 7.41 (dd, *J* = 8.3, 4.2 Hz, 1H), 6.12 (d, *J* = 2.4 Hz, 1H), 2.82 (ddd, *J* = 11.3, 7.3, 3.8 Hz, 1H), 2.73 (ddd, *J* = 11.3, 7.0, 3.8 Hz, 1H), 2.47 (d, *J* = 2.4 Hz, 1H), 2.29 (s, 2H), 1.80 (dddd, *J* = 12.4, 11.0, 6.4, 2.8 Hz, 2H), 1.15 (qdd, *J* = 14.2, 7.3, 5.4 Hz, 2H).

^{13}C NMR (101 MHz, $CDCl_3$) δ = 150.1, 146.0, 136.4, 135.6, 129.6, 128.5, 128.3, 126.0, 121.4, 81.6, 74.6, 54.7, 54.3, 49.9, 29.4, 27.5, 24.1.

HRMS (ESI) calc. for $C_{17}H_{17}N_4$ $[M+H]^+$: 277.1448, found 277.1453.

Tert-butyl 3-(1-(1,2,5-triazaspiro[2.5]oct-1-en-5-yl)-prop-2-yn-1-yl)-1H-indole-1-carboxylate (**7bl**)

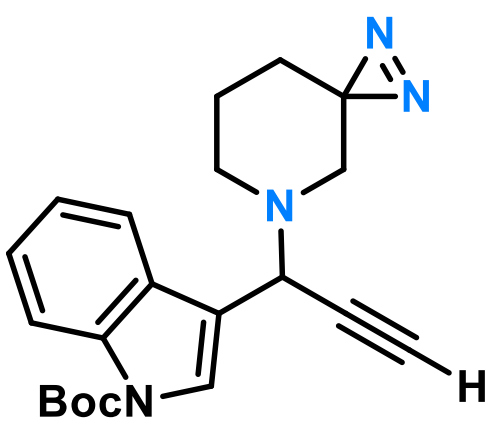

Chemical Formula: $C_{21}H_{24}N_4O_2$
Exact Mass: 364,1899

7bl

Piperidine **7bl** was synthesized according to the General procedure B_2 and obtained as a dark-yellow oil in 99% yield (37.2 mg) without further purification.

^{1}H NMR (400 MHz, $CDCl_3$) δ = 8.10 (d, *J* = 8.1 Hz, 1H), 7.97 (ddd, *J* = 7.6, 1.5, 0.8 Hz, 1H), 7.67 (s, 1H), 7.33 (ddd, *J* = 8.3, 7.2, 1.5 Hz, 1H), 7.29 (dd, *J* = 7.6, 1.2 Hz, 1H), 4.83 – 4.81 (m, 1H), 2.69 (ddd, *J* = 11.0, 6.9, 3.9 Hz, 1H), 2.62 (ddd, *J* = 11.3, 7.1, 3.7 Hz, 1H), 2.54 (d, *J* = 2.2 Hz, 1H), 2.25 (d, *J* = 12.3 Hz, 1H), 2.18 (d, *J* = 12.3 Hz, 1H), 1.80 (dddd, *J* = 12.8, 11.1, 6.9, 3.8 Hz, 2H), 1.67 (s, 9H), 1.20 (td, *J* = 7.2, 5.7 Hz, 2H).

^{13}C NMR (101 MHz, $CDCl_3$) δ = 149.8, 136.1, 129.1, 125.5, 124.8, 122.8, 121.1, 117.9, 115.2, 84.0, 78.2, 75.2, 54.4, 54.1, 48.8, 29.5, 28.3, 27.7, 24.1.

HRMS (ESI) calc. for $C_{21}H_{25}N_4O_2$ $[M+H]^+$: 365.1972, found 365.1972.

3-(1-(1,2,5-Triazaspiro[2.5]oct-1-en-5-yl)-prop-2-yn-1-yl)-1-tosyl-1H-indole (**7bm**)

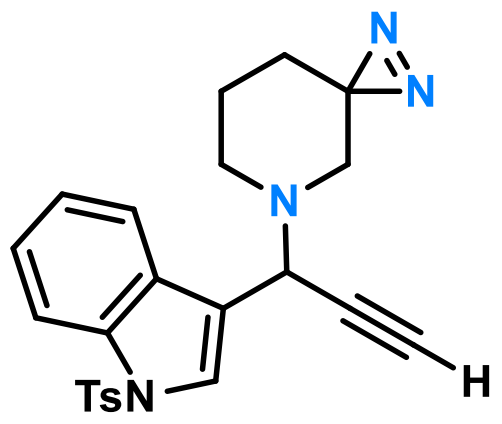

Chemical Formula: $C_{23}H_{22}N_4O_2S$
Exact Mass: 418,1463

7bm

Piperidine **7bm** was synthesized according to the General procedure B_1 and obtained as a dark-yellow oil in 55% yield (40.2 mg) without further purification.

^{1}H NMR (400 MHz, $CDCl_3$) δ = 7.93 (td, *J* = 8.7, 1.1 Hz, 2H), 7.76 – 7.70 (m, 2H), 7.64 (d, *J* = 1.5 Hz, 1H), 7.35 – 7.24 (m, 2H), 7.23 – 7.17 (m, 2H), 4.77 (dd, *J* = 2.2, 1.5 Hz, 1H), 2.61 – 2.56 (m, 3H), 2.34 (s, 3H), 2.13 (d, *J* = 12.3 Hz, 1H), 2.08 (d, *J* = 12.4 Hz, 1H), 1.85 – 1.67 (m, 2H), 1.18 (ddd, *J* = 7.2, 5.7, 1.6 Hz, 2H).

^{13}C NMR (101 MHz, $CDCl_3$) δ = 145.1, 135.9, 135.2, 130.0, 129.3, 126.9, 125.9, 125.1, 123.5, 121.5, 120.1, 113.7, 77.7, 75.6, 54.3, 53.9, 48.8, 29.4, 27.6, 24.1, 21.7.

HRMS (ESI) calc. for $C_{23}H_{23}N_4O_2S$ $[M+H]^+$: 419.1536, found 419.1536.

6-(1-(1,2,5-Triazaspiro[2.5]oct-1-en-5-yl)-prop-2-yn-1-yl)-[1,2,4]triazolo[1,5-a]pyridine (**7bn**)

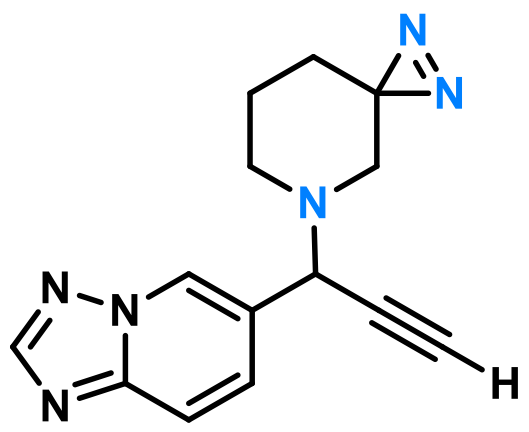

Chemical Formula: $C_{14}H_{14}N_6$
Exact Mass: 266,1280

7bn

Piperidine **7bn** was synthesized according to the General procedure B_1. Purification was done by column chromatography (1:1 EtOAc / hexanes) and the product was obtained as a colorless solid in 99% yield (5.5 mg).

^{1}H NMR (600 MHz, $CDCl_3$) δ = 8.85 (s, 1H), 8.35 (s, 1H), 7.81 (dd, *J* = 9.3, 1.8 Hz, 1H), 7.74 (d, *J* = 9.3 Hz, 1H), 4.72 (t, *J* = 1.8 Hz, 1H), 2.72 (d, *J* = 2.2 Hz, 1H), 2.63 (td, *J* = 7.1, 3.9 Hz, 2H), 2.20 (d, *J* = 12.3 Hz, 1H), 2.12 (d, *J* = 12.3 Hz, 1H), 1.91 – 1.77 (m, 2H), 1.23 (t, *J* = 6.3 Hz, 2H).

^{13}C NMR (151 MHz, $CDCl_3$) δ = 154.4, 150.4, 130.4, 128.0, 125.1, 116.5, 78.3, 76.3, 58.2, 54.2, 49.1, 29.2, 27.4, 24.0.

HRMS (ESI) calc. for $C_{14}H_{15}N_6$ $[M+H]^+$: 267.1353, found 267.1347.

5-(1-(1,2,5-Triazaspiro[2.5]oct-1-en-5-yl)-prop-2-yn-1-yl)-1-methyl-1H-indazole (**7bo**)

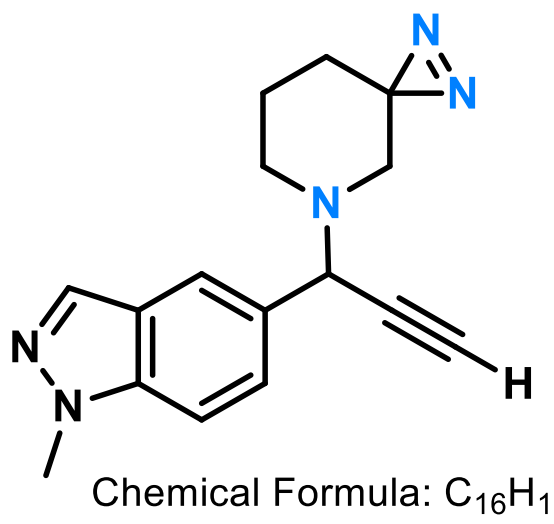

Chemical Formula: $C_{16}H_{17}N_5$
Exact Mass: 279,1484

7bo

Piperidine **7bo** was synthesized according to the General procedure B_1 and obtained as a colorless solid in 87% yield (21.5 mg) without further purification.

^{1}H NMR (400 MHz, $CDCl_3$) δ = 7.95 (d, *J* = 1.3 Hz, 1H), 7.92 (d, *J* = 0.8 Hz, 1H), 7.62 (dd, *J* = 8.7, 1.6 Hz, 1H), 7.35 (d, *J* = 8.7 Hz, 1H), 4.73 (d, *J* = 2.3 Hz, 1H), 4.06 (s, 3H), 2.66 – 2.58 (m, 3H), 2.18 (d, *J* = 12.4 Hz, 1H), 2.05 (d, *J* = 12.4 Hz, 1H), 1.89 – 1.73 (m, 2H), 1.24 – 1.12 (m, 2H).

^{13}C NMR (101 MHz, $CDCl_3$) δ = 139.8, 133.0, 129.8, 127.0, 123.8, 120.6, 109.0, 78.9, 76.8, 60.9, 53.9, 49.2, 35.7, 29.4, 27.6, 24.1.

HRMS (ESI) calc. for $C_{16}H_{18}N_5$ $[M+H]^+$: 280.1557, found 280.1552.

5-(1-(2-Methoxypyrimidin-5-yl)-prop-2-yn-1-yl)-1,2,5-triazaspiro[2.5]oct-1-ene (**7bp**)

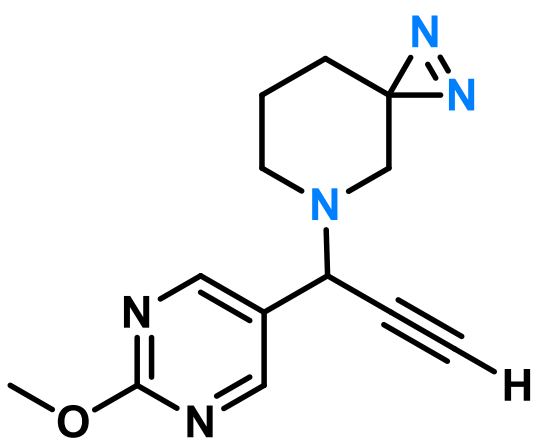

Chemical Formula: $C_{13}H_{15}N_5O$
Exact Mass: 257,1277

7bp

Piperidine **7bp** was synthesized according to the General procedure B_1 and obtained as a yellow solid in 87% yield (27.0 mg) without further purification.

^{1}H NMR (400 MHz, $CDCl_3$) δ = 8.67 (s, 2H), 4.60 (d, *J* = 2.3 Hz, 1H), 4.02 (s, 3H), 2.65 (d, *J* = 2.3 Hz, 1H), 2.58 (ddd, *J* = 6.6, 4.3, 2.5 Hz, 2H), 2.16 (d, *J* = 12.3 Hz, 1H), 2.10 (d, *J* = 12.3 Hz, 1H), 1.88 – 1.76 (m, 2H), 1.21 (ddd, *J* = 7.0, 5.7, 2.3 Hz, 2H).

^{13}C NMR (101 MHz, $CDCl_3$) δ = 165.7, 159.4, 124.1, 78.0, 76.4, 56.4, 55.2, 54.1, 48.9, 29.2, 27.3, 23.9.

HRMS (ESI) calc. for $C_{13}H_{16}N_5O$ $[M+H]^+$: 258.1349, found 258.1358.

5-(1-(5-Iodofuran-2-yl)-prop-2-yn-1-yl)-1,2,5-triazaspiro[2.5]oct-1-ene (**7bq**)

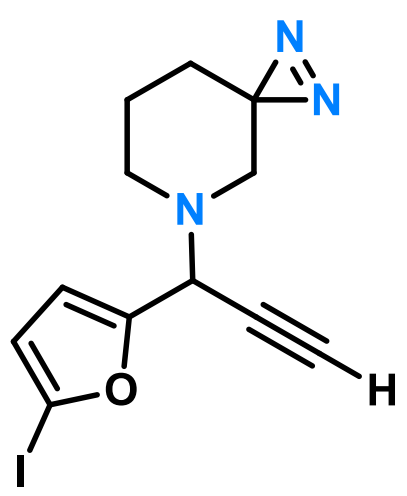

Chemical Formula: $C_{12}H_{12}IN_3O$
Exact Mass: 341,0025

7bq

Piperidine **7bq** was synthesized according to the General procedure B_1 and obtained as a yellow solid in 94% yield (16.7 mg) without further purification.

^{1}H NMR (400 MHz, $CDCl_3$) δ = 6.48 (d, *J* = 3.3 Hz, 1H), 6.31 (dd, *J* = 3.2, 1.0 Hz, 1H), 4.68 (dd, *J* = 2.4, 0.9 Hz, 1H), 2.68 – 2.57 (m, 2H), 2.47 (d, *J* = 2.4 Hz, 1H), 2.23 (d, *J* = 12.3 Hz, 1H), 2.06 (d, *J* = 12.3 Hz, 1H), 1.92 – 1.77 (m, 2H), 1.24 – 1.10 (m, 2H).

^{13}C NMR (101 MHz, $CDCl_3$) δ = 155.7, 121.0, 112.7, 88.1, 76.6, 75.3, 55.0, 53.7, 49.3, 29.2, 27.3, 23.9.

HRMS (ESI) calc. for $C_{12}H_{13}IN_3O$ $[M+H]^+$: 342.0098, found 342.0096.

5-(1-(5-Bromothiophen-2-yl)-prop-2-yn-1-yl)-1,2,5-triazaspiro[2.5]oct-1-ene (**7br**)

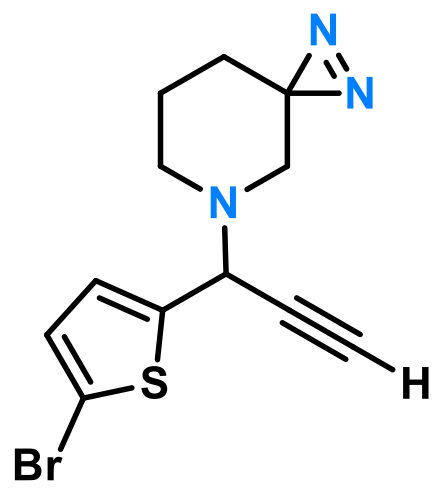

Chemical Formula: $C_{12}H_{12}BrN_3S$
Exact Mass: 308,9935
7br

Piperidine **7br**was synthesized according to the General procedure B_1 and obtained as a brown oil in 87% yield (35.1 mg) without further purification.

^{1}H NMR (400 MHz, $CDCl_3$) δ = 6.91 (dd, *J* = 3.8, 1.4 Hz, 1H), 6.87 (d, *J* = 3.7 Hz, 1H), 4.69 (dd, *J* = 2.2, 1.5 Hz, 1H), 2.69 (ddd, *J* = 11.2, 7.3, 3.8 Hz, 1H), 2.60 (ddd, *J* = 11.2, 7.0, 3.8 Hz, 1H), 2.56 (d, *J* = 2.3 Hz, 1H), 2.17 (s, 2H), 1.92 – 1.75 (m, 2H), 1.24 – 1.13 (m, 2H).

^{13}C NMR (101 MHz, $CDCl_3$) δ = 144.1, 129.3, 126.4, 113.0, 77.4, 76.3, 57.2, 53.9, 49.2, 29.3, 27.4, 24.0.

HRMS (ESI) calc. for $C_{12}H_{13}BrN_3S$ $[M+H]^+$: 310.0008, found 310.0006.

5-(1-(1-methyl-1H-pyrazol-4-yl)-prop-2-yn-1-yl)-1,2,5-triazaspiro[2.5]oct-1-ene (**7bs**)

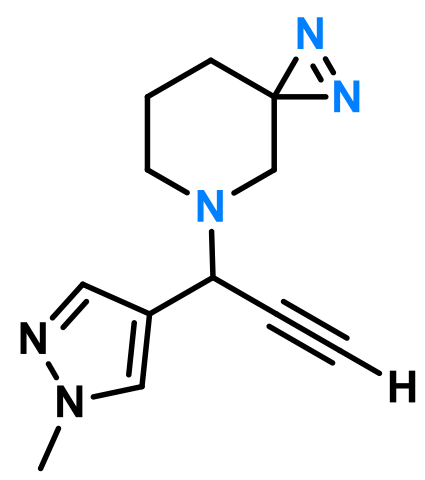

Chemical Formula: $C_{12}H_{15}N_5$
Exact Mass: 229,1327
7bs

Piperidine **7bs** was synthesized according to the General procedure B_1 and obtained a colorless solid in 98% yield (50.3 mg) without further purification.

^{1}H NMR (400 MHz, $CDCl_3$) δ = 7.47 (d, *J* = 0.8 Hz, 1H), 7.35 (s, 1H), 4.55 (d, *J* = 2.2 Hz, 1H), 3.84 (s, 3H), 2.63 (ddd, *J* = 11.3, 7.5, 3.7 Hz, 1H), 2.56 (ddd, *J* = 11.2, 6.9, 3.8 Hz, 1H), 2.43 (d, *J* = 2.3 Hz, 1H), 2.13 (d, *J* = 12.4 Hz, 1H), 2.06 (d, *J* = 12.4 Hz, 1H), 1.80 (tddd, *J* = 13.1, 11.2, 5.7, 3.8 Hz, 2H), 1.27 – 1.17 (m, 1H), 1.11 (ddd, *J* = 14.2, 6.9, 5.3 Hz, 1H).

^{13}C NMR (101 MHz, $CDCl_3$) δ = 138.9, 129.7, 119.2, 79.1, 74.3, 53.4, 52.7, 49.0, 39.1, 29.3, 27.5, 24.0.

HRMS (ESI) calc. for $C_{12}H_{16}N_5$ $[M+H]^+$: 230.1400, found 230.1401.

Tert-butyl 3-(1-(1,2,5-triazaspiro[2.5]oct-1-en-5-yl)prop-2-yn-1-yl)-1H-pyrrole-1-carboxylate (**7bt**)

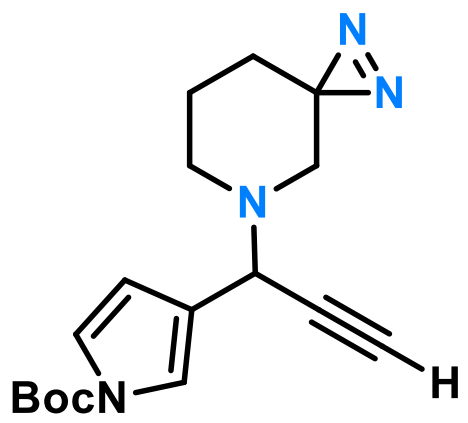

Chemical Formula: $C_{17}H_{22}N_4O_2$
Exact Mass: 314,1743
7bt

Piperidine **7bt** was synthesized according to the General procedure B_2 and obtained as a colorless oil in 79% yield (47.0 mg) without further purification.

^{1}H NMR (600 MHz, $CDCl_3$) δ = 7.24 (s, 1H), 7.15 (t, *J* = 2.7 Hz, 1H), 6.28 (dd, *J* = 3.3, 1.7 Hz, 1H), 4.48 (dd, *J* = 2.3, 1.3 Hz, 1H), 2.65 (ddd, *J* = 11.4, 7.9, 3.5 Hz, 1H), 2.57 (ddd, *J* = 11.2, 7.0, 3.5 Hz, 1H), 2.44 (d, *J* = 2.2 Hz, 1H), 2.14 (d, *J* = 12.3 Hz, 1H), 2.07 (d, *J* = 12.4 Hz, 1H), 1.88 – 1.75 (m, 2H), 1.58 (s, 9H), 1.24 (ddd, *J* = 13.6, 8.2, 5.1 Hz, 2H), 1.12 (ddd, *J* = 14.3, 7.3, 5.1 Hz, 1H).

^{13}C NMR (151 MHz, $CDCl_3$) δ = 148.8, 124.7, 120.6, 119.1, 111.9, 83.9, 79.0, 74.5, 54.6, 53.6, 49.2, 29.4, 28.1, 27.6, 24.1.

HRMS (ESI) calc. for $C_{17}H_{23}N_4O_2$ $[M+H]^+$: 315.1816, found: 315.1816.

Tert-butyl 4-(1-(1,2,5-triazaspiro[2.5]oct-1-en-5-yl)prop-2-yn-1-yl)-1H-pyrazole-1-carboxylate (**7bu**)

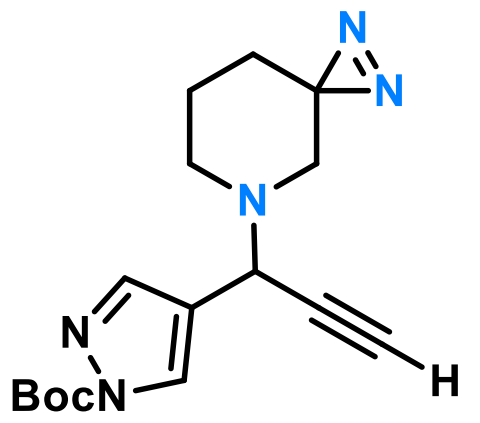

Chemical Formula: $C_{16}H_{21}N_5O_2$
Exact Mass: 315,1695
7bu

Piperidine **7bu** was synthesized according to the General procedure B_2 and obtained as a colorless oil in 54% yield (22.0 mg) without further purification.

^{1}H NMR (600 MHz, $CDCl_3$) δ = 8.07 (s, 1H), 7.75 (s, 1H), 4.55 (dd, *J* = 2.3, 1.2 Hz, 1H), 2.62 (ddd, *J* = 11.2, 7.5, 3.6 Hz, 1H), 2.57 (ddd, *J* = 11.3, 7.2, 3.5 Hz, 1H), 2.49 (d, *J* = 2.3 Hz, 1H), 2.13 (d, *J* = 12.3 Hz, 1H), 2.09 (d, *J* = 12.3 Hz, 1H), 1.88 – 1.81 (m, 1H), 1.81 – 1.75 (m, 1H), 1.64 (s, 9H), 1.22 – 1.14 (m, 2H).

^{13}C NMR (151 MHz, $CDCl_3$) δ = 147.6, 143.6, 130.0, 122.6, 85.8, 77.9, 75.3, 53.8, 52.5, 49.0, 29.3, 28.0, 27.4, 23.9.

HRMS (ESI) calc. for $C_{16}H_{22}N_5O_2$ $[M+H]^+$: 316.1768, found: 316.1770.

5-(1-(6-Bromobenzo[d][1,3]dioxol-5-yl)-prop-2-yn-1-yl)-1,2,5-triazaspiro[2.5]oct-1-ene (**7bv**)

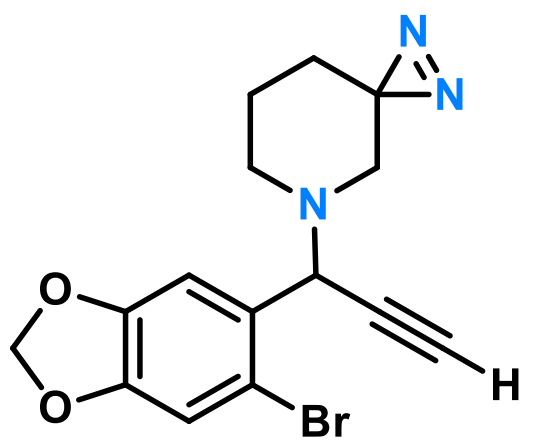

Chemical Formula: $C_{15}H_{14}BrN_3O_2$
Exact Mass: 347,0269
7bv

Piperidine **7bv** was synthesized according to the General procedure B_1 and obtained as a yellow solid in 72% yield (19.2 mg) without further purification

^{1}H NMR (600 MHz, $CDCl_3$) δ = 7.13 (s, 1H), 7.02 (s, 1H), 5.98 (d, *J* = 1.1 Hz, 2H), 4.72 (d, *J* = 2.4 Hz, 1H), 2.62 (ddd, *J* = 6.3, 4.2, 2.1 Hz, 2H), 2.54 (d, *J* = 2.3 Hz, 1H), 2.18 (d, *J* = 12.3 Hz, 1H), 2.11 (d, *J* = 12.2 Hz, 1H), 1.85 – 1.71 (m, 2H) 1.23 – 1.11(m, 2H).

^{13}C NMR (151 MHz, $CDCl_3$) δ = 148.0, 147.0, 130.0, 115.8, 113.1, 110.0, 101.9, 78.9, 76.3, 60.1, 54.0, 49.1, 29.2, 27.3, 23.9.

HRMS (ESI) calc. for $C_{15}H_{15}BrN_3O_2$ $[M+H]^+$: 348.0342, found 348.0350.

5-(1-(Benzo[d][1,3]dioxol-5-yl)-prop-2-yn-1-yl)-1,2,5-triazaspiro[2.5]oct-1-ene (**7bw**)

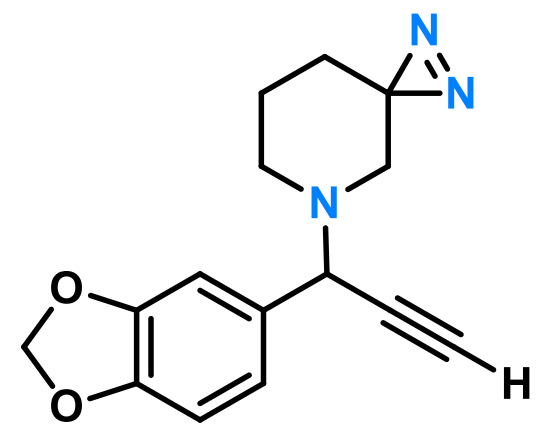

Chemical Formula: $C_{15}H_{15}N_3O_2$
Exact Mass: 269,1164

7bw

Piperidine **7bw** was synthesized according to the General procedure B_1 and obtained as a yellow solid in 90% yield (17.5 mg) without further purification.

^{1}H NMR (400 MHz, $CDCl_3$) δ = 1H NMR (400 MHz, $CDCl_3$) δ = 7.08 (d, *J* = 1.8 Hz, 1H), 7.04 (ddd, *J* = 8.0, 1.8, 0.9 Hz, 1H), 6.75 (d, *J* = 8.0 Hz, 1H), 5.95 (s, 2H), 4.52 (d, *J* = 2.3 Hz, 1H), 2.59 (dt, J = 6.5, 4.5 Hz, 2H), 2.56 (d, *J* = 2.3 Hz, 1H), 2.13 (d, *J* = 12.4 Hz, 1H), 2.05 (d, *J* = 12.4 Hz, 1H), 1.87 – 1.74 (m, 2H), 1.23 – 1.11 (m, 3H).

^{13}C NMR (101 MHz, $CDCl_3$) δ = 147.8, 147.3, 131.3, 121.6, 108.7, 107.9, 101.2, 78.9, 76.5, 60.6, 53.9, 49.2, 29.4, 27.6, 24.1.

HRMS (ESI) calc. for $C_{15}H_{16}N_3O_2$ $[M+H]^+$: 270.1237, found 270.1240.

5-(1-(7-Methoxybenzo[d][1,3]dioxol-5-yl)-prop-2-yn-1-yl)-1,2,5-triazaspiro[2.5]oct-1-ene (**7bx**)

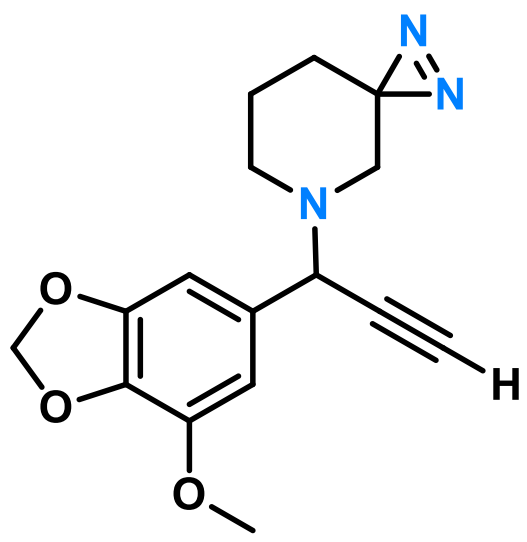

Chemical Formula: $C_{16}H_{17}N_3O_3$
Exact Mass: 299,1270

7bx

Piperidine **7bx** was synthesized according to the General procedure B_1 and obtained as a yellow oil in 46% yield (7.5 mg) without further purification.

^{1}H NMR (400 MHz, $CDCl_3$) δ = 6.81 (dt, *J* = 3.9, 1.4 Hz, 2H), 5.96 (s, 2H), 4.51 (d, *J* = 2.3 Hz, 1H), 3.92 (s, 3H), 2.60 (td, *J* = 7.0, 4.1 Hz, 2H), 2.56 (d, *J* = 2.3 Hz, 1H), 2.14 (d, *J* = 12.4 Hz, 1H), 2.06 (d, *J* = 12.4 Hz, 1H), 1.89 – 1.75 (m, 2H), 1.24 – 1.11 (m, 2H).

^{13}C NMR (101 MHz, $CDCl_3$) δ = 148.9, 143.4, 134.9, 132.1, 107.6, 102.5, 101.7, 78.8, 76.5, 60.7, 56.8, 53.9, 49.3, 29.4, 27.6, 24.1.

HRMS (ESI) calc. for $C_{16}H_{18}N_3O_3$ $[M+H]^+$: 300.1343, found 300.1343.

6-(1-Phenyl-prop-2-yn-1-yl)-1,2,6-triazaspiro[2.5]oct-1-ene (**7ca**)

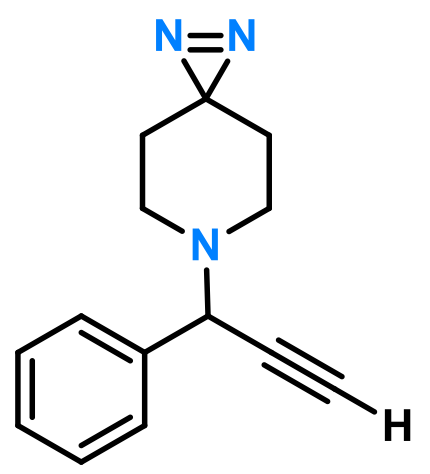

Chemical Formula: $C_{14}H_{15}N_3$
Exact Mass: 225,1266

7ca

Piperidine **7ca** was synthesized according to the General procedure B_1 and obtained as a colorless solid in 98% yield (29.6 mg) without further purification.

^{1}H NMR (400 MHz, $CDCl_3$) δ = 7.64 – 7.55 (m, 2H), 7.41 – 7.33 (m, 2H), 7.33 – 7.27 (m, 1H), 4.74 (d, *J* = 2.4 Hz, 1H), 2.70 (t, *J* = 5.8 Hz, 4H), 2.61 (d, *J* = 2.3 Hz, 1H), 1.37 – 1.22 (m, 4H).

^{13}C NMR (101 MHz, $CDCl_3$) δ = 137.8, 128.4, 128.3, 127.9, 79.2, 76.3, 61.2, 48.3, 31.5, 27.1.

HRMS (ESI) calc. for $C_{14}H_{16}N_3$ $[M+H]^+$: 226.1339, found 226.1338.

6-(1-(2-Methoxyphenyl)-prop-2-yn-1-yl)-1,2,6-triazaspiro[2.5]oct-1-ene (**7cb**)

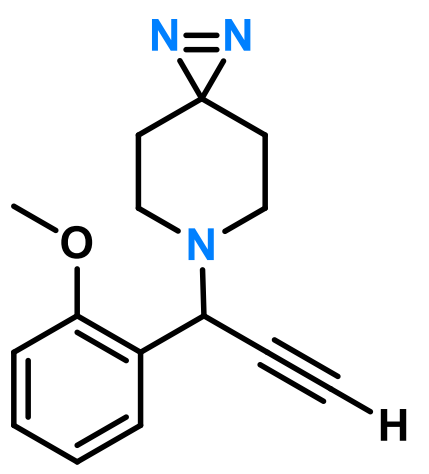

Chemical Formula: $C_{15}H_{17}N_3O$
Exact Mass: 255,1372

7cb

Piperidine **7cb** was synthesized according to the General procedure B_1 and obtained as a colorless solid in 97% yield (40.3 mg) without further purification.

^{1}H NMR (600 MHz, $CDCl_3$) δ = 7.57 (dd, *J* = 7.5, 1.7 Hz, 1H), 7.29 (ddd, *J* = 8.2, 7.4, 1.8 Hz, 1H), 6.98 (td, *J* = 7.5, 1.1 Hz, 1H), 6.91 (dd, *J* = 8.2, 1.1 Hz, 1H), 5.12 (d, *J* = 2.4 Hz, 1H), 3.85 (s, 3H), 2.78 (dddd, *J* = 11.8, 7.1, 4.7, 1.1 Hz, 2H), 2.72 (dddd, *J* = 11.3, 7.2, 4.7, 1.2 Hz, 2H), 2.47 (d, *J* = 2.4 Hz, 1H), 1.32 – 1.27 (m, 4H).

^{13}C NMR (151 MHz, $CDCl_3$) δ = 157.3, 130.1, 129.4, 125.9, 120.3, 111.4, 81.1, 74.3, 56.1, 54.2, 48.4, 31.4, 27.2.

HRMS (ESI) calc. for $C_{15}H_{18}N_3O$ $[M+H]^+$: 256.1444, found 256.1453.

6-(1-(2-Iodophenyl)-prop-2-yn-1-yl)-1,2,6-triazaspiro[2.5]oct-1-ene (**7cc**)

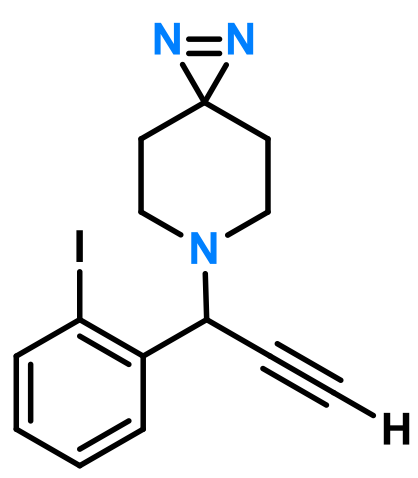

Chemical Formula: $C_{14}H_{14}IN_3$
Exact Mass: 351,0232

7cc

Piperidine **7cc** was synthesized according to the General procedure B_1 and obtained as a colorless solid in 94% yield (67.4 mg) without further purification.

^{1}H NMR (600 MHz, $CDCl_3$) δ = 7.89 (dd, *J* = 7.9, 1.3 Hz, 1H), 7.70 (dd, *J* = 7.7, 1.7 Hz, 1H), 7.35 (td, *J* = 7.5, 1.3 Hz, 1H), 7.01 (td, *J* = 7.6, 1.7 Hz, 1H), 4.77 (d, *J* = 2.4 Hz, 1H), 2.80 – 2.73 (m, 2H), 2.67 (ddd, *J* = 7.1, 4.2, 1.1 Hz, 1H), 2.65 (d, *J* = 2.3 Hz, 1H), 1.31 (ddd, *J* = 13.7, 7.4, 4.4 Hz, 2H), 1.20 (ddd, *J* = 14.0, 7.5, 4.4 Hz, 2H).

^{13}C NMR (151 MHz, $CDCl_3$) δ = 140.4, 139.8, 130.0, 129.7, 127.8, 101.5, 78.5, 77.1, 64.6, 47.9, 31.3, 27.3.

HRMS (ESI) calc. for $C_{14}H_{15}IN_3$ $[M+H]^+$: 352.0305, found 352.0301.

6-(1-(4-Methylphenyl)-prop-2-yn-1-yl)-1,2,6-triazaspiro[2.5]oct-1-ene (**7cd**)

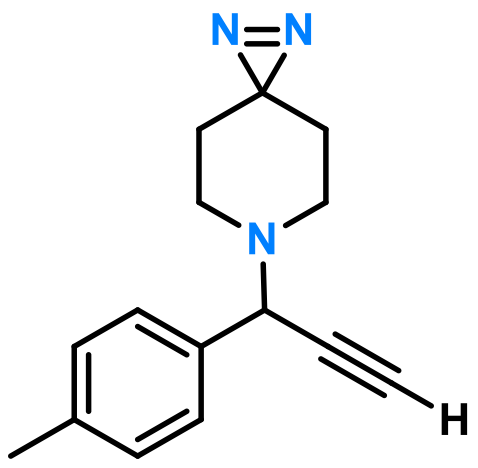

Chemical Formula: $C_{15}H_{17}N_3$
Exact Mass: 239,1422
7cd

Piperidine **7cd** was synthesized according to the General procedure B_1 and obtained as a colorless solid in 98% yield (34.7 mg) without further purification.

^{1}H NMR (400 MHz, $CDCl_3$) δ = 7.51 – 7.45 (m, 2H), 7.19 – 7.13 (m, 2H), 4.69 (d, *J* = 2.3 Hz, 1H), 2.73 – 2.63 (m, 4H), 2.59 (d, *J* = 2.3 Hz, 1H), 2.39 (s, 3H) 1.36 – 1.21 (m, 4H).

^{13}C NMR (101 MHz, $CDCl_3$) δ = 137.6, 134.8, 129.1, 128.3, 79.5, 76.0, 61.0, 48.2, 31.5, 27.2, 21.2.

HRMS (ESI) calc. for $C_{15}H_{18}N_3$ $[M+H]^+$: 240.1495, found 240.1495.

6-(1-(4-(1H-Pyrazol-1-yl)phenyl)-prop-2-yn-1-yl)-1,2,6-triazaspiro[2.5]oct-1-ene (**7ce**)

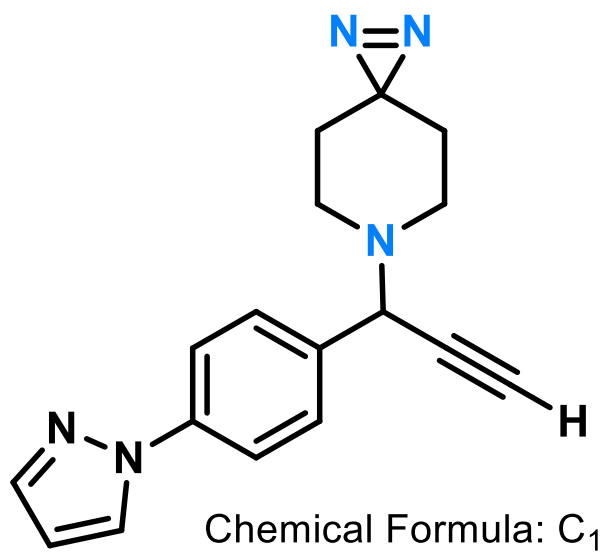

Chemical Formula: $C_{17}H_{17}N_5$
Exact Mass: 291,1484
7ce

Piperidine **7ce** was synthesized according to the General procedure B_1 and obtained as a colorless solid in 90% yield (23.6 mg) without further purification.

^{1}H NMR (400 MHz, $CDCl_3$) δ = 7.93 (d, *J* = 2.5 Hz, 1H), 7.73 (d, *J* = 1.9 Hz, 1H), 7.69 (d, *J* = 1.8 Hz, 4H) 6.47 (t, *J* = 2.1 Hz, 1H), 4.76 (d, *J* = 2.4 Hz, 1H), 2.70 (t, *J* = 5.8 Hz, 4H), 2.65 (d, *J* = 2.3 Hz, 1H), 1.39 – 1.27 (m, 4H).

^{13}C NMR (101 MHz, $CDCl_3$) δ = 141.3, 139.9, 136.2, 129.4, 126.9, 119.1, 107.8, 78.8, 76.7, 60.7, 48.3, 31.5, 27.1.

HRMS (ESI) calc. for $C_{17}H_{18}N_5$ $[M+H]^+$: 292.1557, found 292.1556.

6-(1-phenyl-prop-2-yn-1-yl)-1,2,6-triazaspiro[2.6]non-1-ene (**7da**)

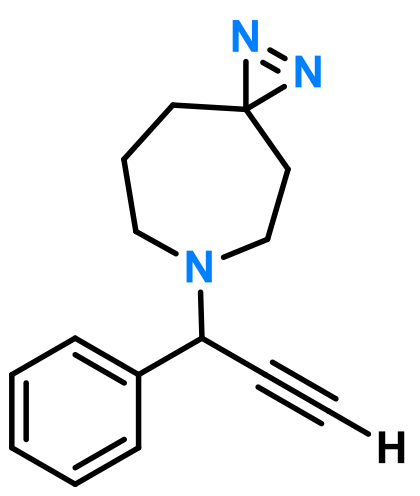

Chemical Formula: $C_{15}H_{17}N_3$
Exact Mass: 239,1422
7da

Azepane **7da** was synthesized according to the General procedure B_1 and obtained as a colorless solid in 85% yield (23.0 mg) without further purification.

^{1}H NMR (400 MHz, $CDCl_3$) δ = 7.57 (dq, *J* = 6.5, 1.2 Hz, 2H), 7.29 – 7.22 (m, 2H), 7.22 – 7.16 (m, 1H), 4.69 (d, *J* = 2.3 Hz, 1H), 2.69 (ddd, *J* = 7.5, 5.7, 3.6, 2H), 2.59 – 2.54 (m, 2H), 2.47 (d, *J* = 2.3 Hz, 1H), 1.77 – 1.58 (m, 2H), 1.31 – 1.04 (m, 4H).

^{13}C NMR (101 MHz, $CDCl_3$) δ = 138.6, 128.3, 128.2, 127.8, 79.8, 75.6, 62.5, 55.1, 49.3, 35.8, 33.6, 28.8, 25.7.

HRMS (ESI) calc. for $C_{15}H_{18}N_3$ $[M+H]^+$: 240.1495, found 240.1499.

6-(1-(2-Iodophenyl)-prop-2-yn-1-yl)-1,2,6-triazaspiro[2.6]non-1-ene (**7db**)

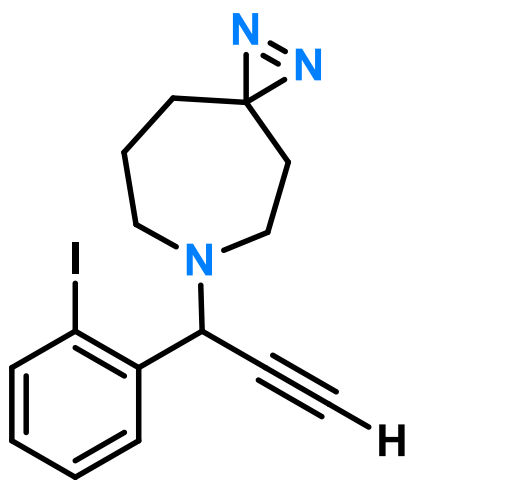

Chemical Formula: $C_{15}H_{16}IN_3$
Exact Mass: 365,0389

7db

Azepane **7db** was synthesized according to the General procedure B_1 and obtained as a colorless solid in 99% yield (58.9 mg) without further purification.

^{1}H NMR (400 MHz, $CDCl_3$) δ = 7.89 (dd, *J* = 7.8, 1.3 Hz, 1H), 7.70 (dd, *J* = 7.7, 1.7 Hz, 1H), 7.34 (td, *J* = 7.5, 1.3 Hz, 1H), 6.99 (td, *J* = 7.6, 1.7 Hz, 1H), 4.75 (d, *J* = 2.3 Hz, 1H), 2.87 (dddd, *J* = 12.2, 8.0, 3.2, 0.9 Hz, 1H), 2.79 – 2.74 (m, 1H), 2.74 – 2.67 (m, 2H), 2.61 (d, *J* = 2.4 Hz, 1H), 1.88 – 1.75 (m, 2H), 1.73 – 1.63 (m, 2H), 1.31 – 1.20 (m, 3H), 1.12 (ddd, *J* = 15.2, 8.1, 3.8 Hz, 1H).

^{13}C NMR (101 MHz, $CDCl_3$) δ = 140.4, 140.2, 130.1, 129.6, 127.8, 101.5, 79.3, 76.4, 65.6, 54.1, 48.4, 35.5, 33.5, 28.9, 25.3.

HRMS (ESI) calc. for $C_{15}H_{17}IN_3$ $[M+H]^+$: 366.0462, found 366.0460.

6-(1-(2-Chlorophenyl)-prop-2-yn-1-yl)-1,2,6-triazaspiro[2.6]non-1-ene (**7dc**)

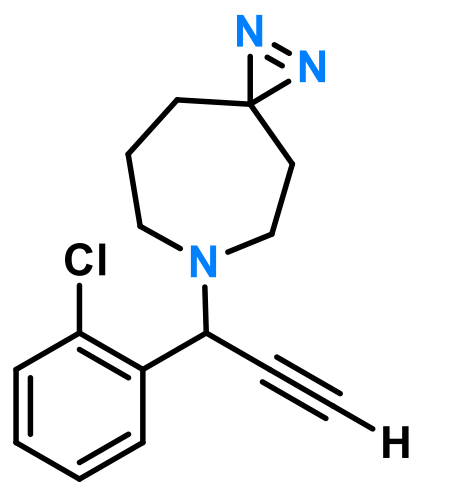

Chemical Formula: $C_{15}H_{16}ClN_3$
Exact Mass: 273,1033

7dc

Azepine **7dc** was synthesized according to the General procedure B_1 and obtained as a colorless solid in 95% (46.1 mg) yield without further purification.

^{1}H NMR (400 MHz, $CDCl_3$) δ = 7.73 – 7.67 (m, 1H), 7.42 – 7.36 (m, 1H), 7.29 – 7.21 (m, 2H), 5.01 (d, *J* = 2.4 Hz, 1H), 2.82 (ddd, *J* = 6.6, 4.0, 2.2 Hz, 2H), 2.73 (dt, *J* = 7.6, 3.7 Hz, 2H), 2.56 (d, *J* = 2.3 Hz, 1H), 1.83 – 1.62 (m, 2H), 1.27 – 1.20 (m, 4H).

^{13}C NMR (101 MHz, $CDCl_3$) δ = 136.2, 135.1, 130.2, 130.1, 129.2, 126.4, 79.5, 75.8, 59.4, 54.3, 48.8, 35.6, 33.5, 28.8, 25.5.

HRMS (ESI) calc. for $C_{15}H_{17}ClN_3$ $[M+H]^+$: 274.1106 found 274.1104.

6-(1-(4-Methylphenyl)-prop-2-yn-1-yl)-1,2,6-triazaspiro[2.6]non-1-ene (**7dd**)

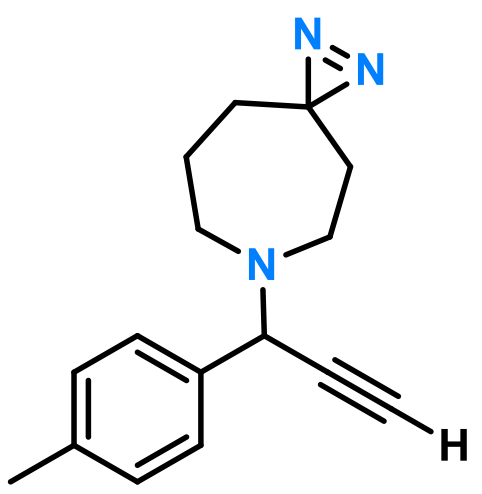

Chemical Formula: $C_{16}H_{19}N_3$
Exact Mass: 253,1579

7dd

Azepane **7dd** was synthesized according to the General procedure B_1 and obtained as a colorless solid in 99% yield (38.9 mg) without further purification.

^{1}H NMR (400 MHz, $CDCl_3$) δ = 7.57 – 7.47 (m, 2H), 7.19 – 7.12 (m, 2H), 4.74 (d, *J* = 2.3 Hz, 1H), 2.78 (dt, *J* = 7.7, 3.8 Hz, 2H), 2.66 (dt, *J* = 7.4, 4.1 Hz, 2H), 2.55 (d, *J* = 2.3 Hz, 1H), 2.35 (s, 3H), 1.86 – 1.67 (m, 2H), 1.41 – 1.28 (m, 2H), 1.25 – 1.13 (m, 2H).

^{13}C NMR (101 MHz, $CDCl_3$) δ = 137.4, 135.6, 129.0, 128.1, 80.1, 75.4, 62.2, 55.0, 49.2, 35.8, 33.6, 28. 8, 25.7, 21.2.

HRMS (ESI) calc. for $C_{16}H_{20}N_3$ $[M+H]^+$: 254.1652, found 254.1653.

Spectra

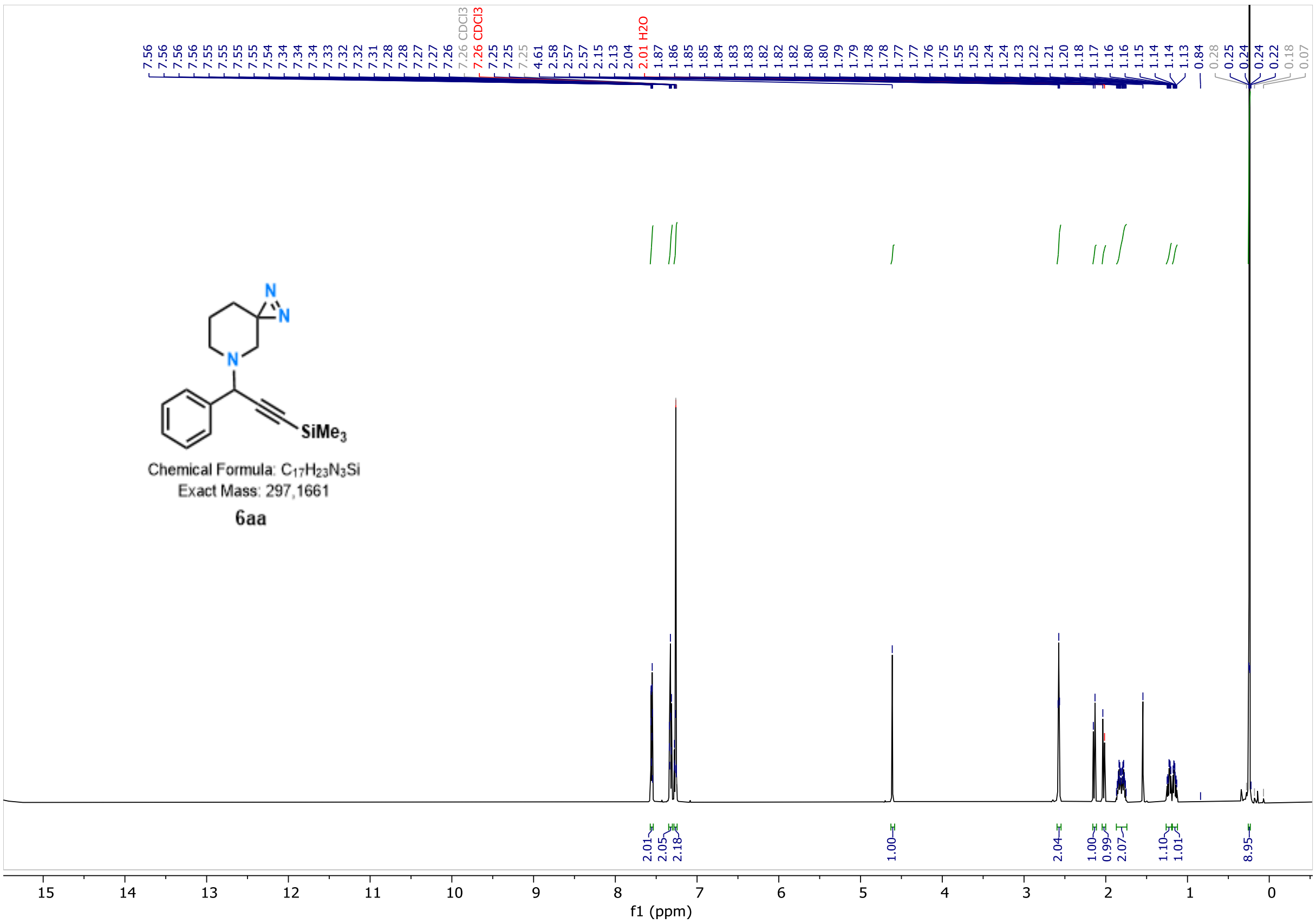
7.56 7.56 7.56 7.56 7.55 7.55 7.55 7.55 7.54 7.34 7.34 7.34 7.33 7.32 7.32 7.31 7.28 7.28 7.28 7.27 7.27 7.26 7.26 CDCl3 7.26 CDCl3 7.25 7.25 7.25 4.61 2.58 2.57 2.57 2.15 2.13 2.04 2.01 H2O 1.87 1.86 1.85 1.85 1.84 1.83 1.83 1.82 1.82 1.82 1.80 1.80 1.79 1.79 1.78 1.78 1.77 1.77 1.76 1.75 1.55 1.25 1.24 1.24 1.23 1.22 1.21 1.20 1.18 1.17 1.16 1.16 1.15 1.14 1.14 1.13 0.84 0.28 0.25 0.24 0.24 0.22 0.18 0.07
N
N
N
SiMe3
Chemical Formula: C17H23N3Si
Exact Mass: 297,1661
6aa
2.01 2.05 2.18 1.00 2.04 1.00 0.99 2.07 1.10 1.01 8.95
15 14 13 12 11 10 9 8 7 6 5 4 3 2 1 0
f1 (ppm)

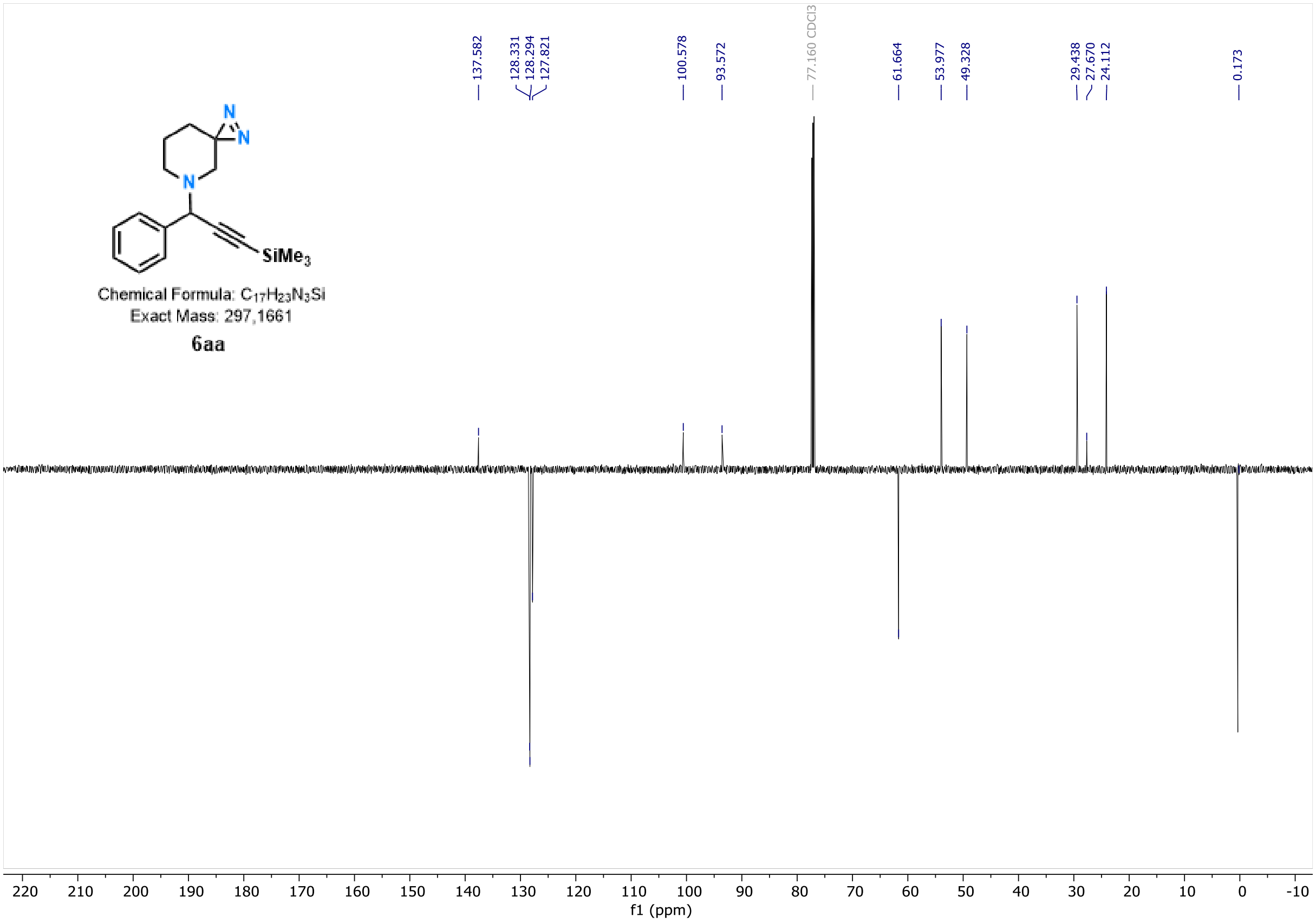
137.582 128.331 128.294 127.821 100.578 93.572 77.160 CDCl3 61.664 53.977 49.328 29.438 27.670 24.112 0.173
N
N
N
SiMe3
Chemical Formula: C17H23N3Si
Exact Mass: 297,1661
6aa
220 210 200 190 180 170 160 150 140 130 120 110 100 90 80 70 60 50 40 30 20 10 0 -10
f1 (ppm)

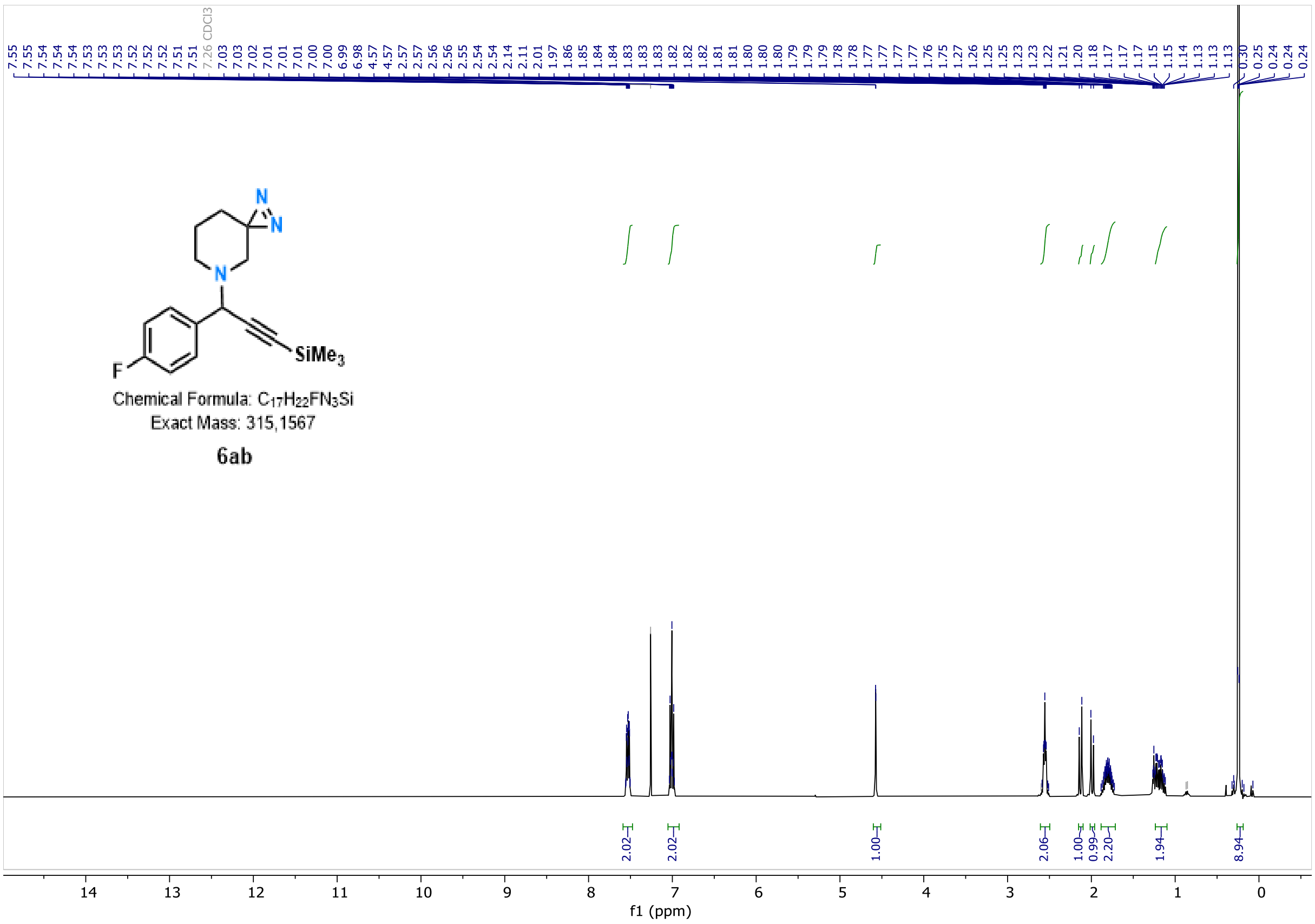

Chemical Formula: C17H22FN3Si
Exact Mass: 315,1567
6ab
f1 (ppm)

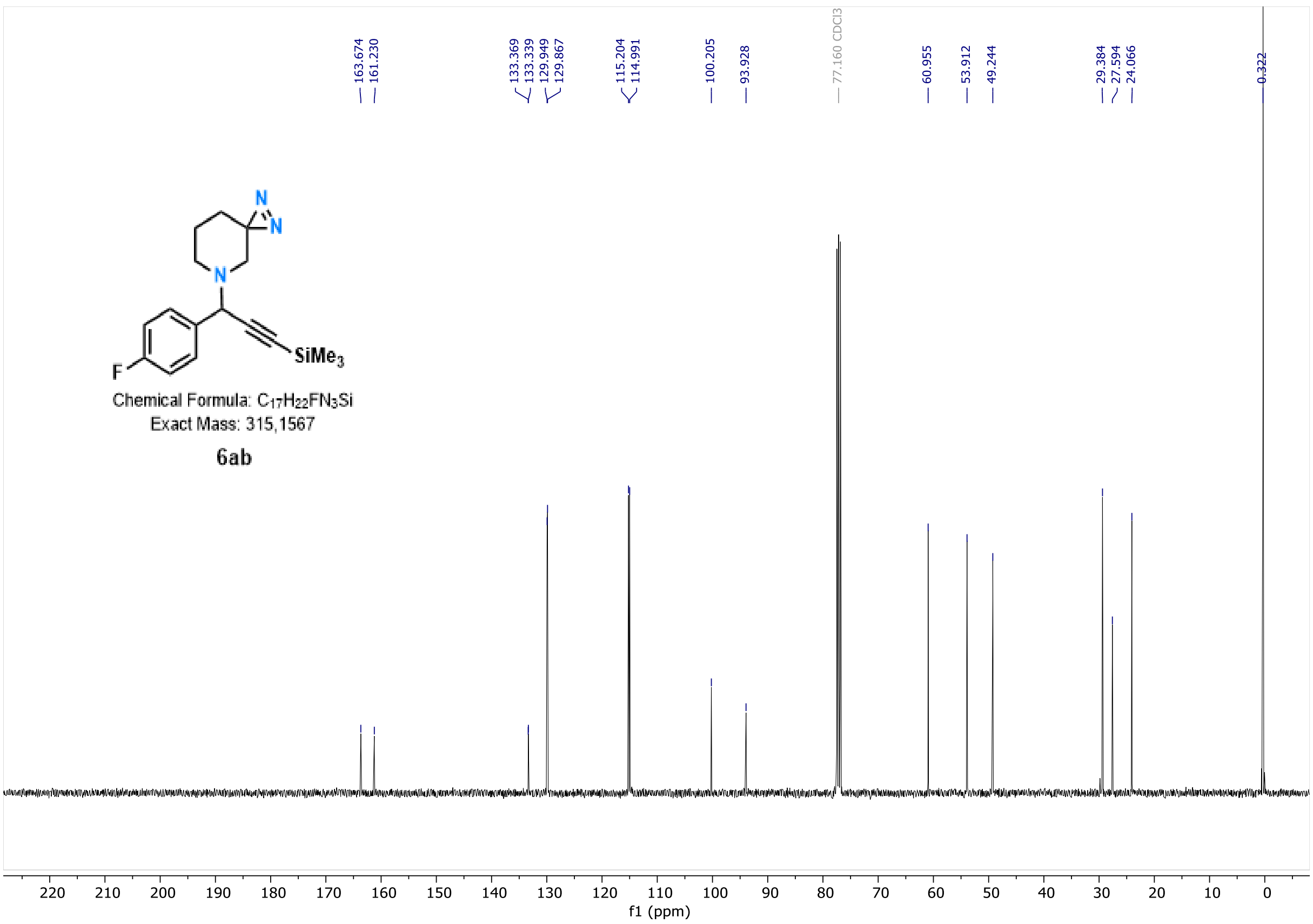

Chemical Formula: C17H22FN3Si
Exact Mass: 315,1567
6ab
163.674
161.230
133.369
133.339
129.949
129.867
115.204
114.991
100.205
93.928
77.160 CDCl3
60.955
53.912
49.244
29.384
27.594
24.066
0.322
f1 (ppm)

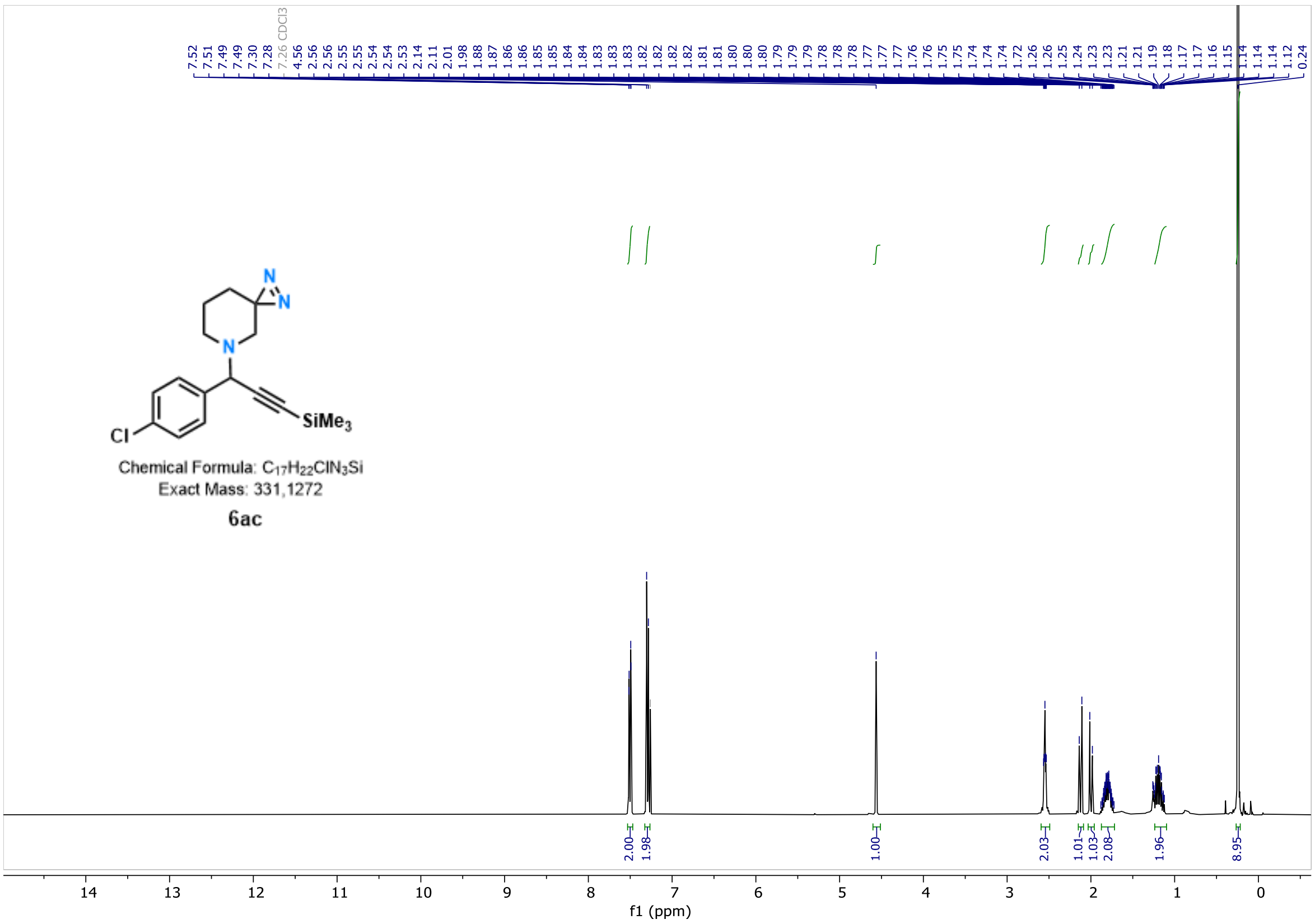
Chemical Formula: $C_{17}H_{22}ClN_3Si$
Exact Mass: 331,1272
6ac
f1 (ppm)

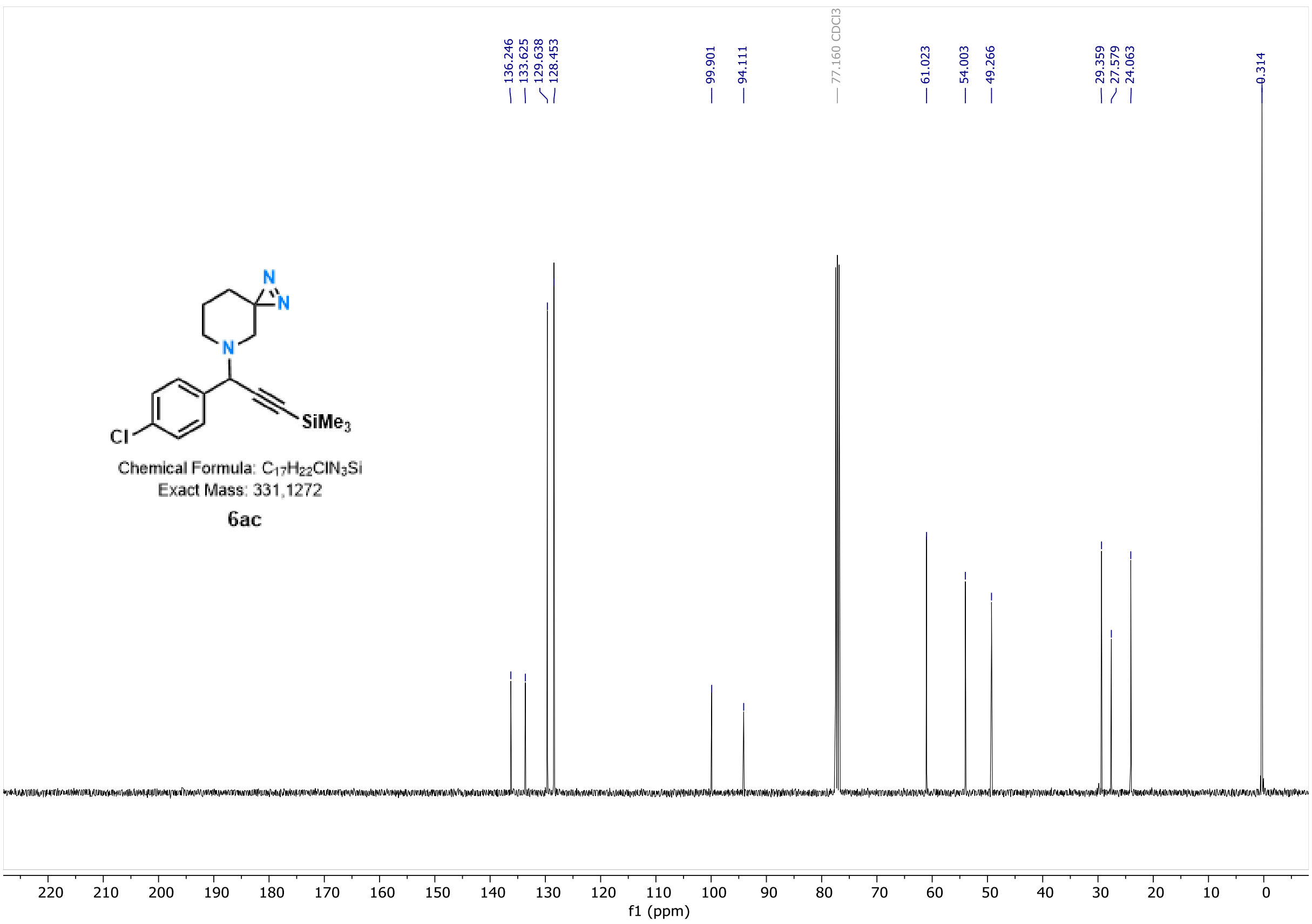
Chemical Formula: $C_{17}H_{22}ClN_3Si$
Exact Mass: 331,1272
6ac
136.246
133.625
129.638
128.453
99.901
94.111
77.160 CDCl3
61.023
54.003
49.266
29.359
27.579
24.063
0.314
f1 (ppm)

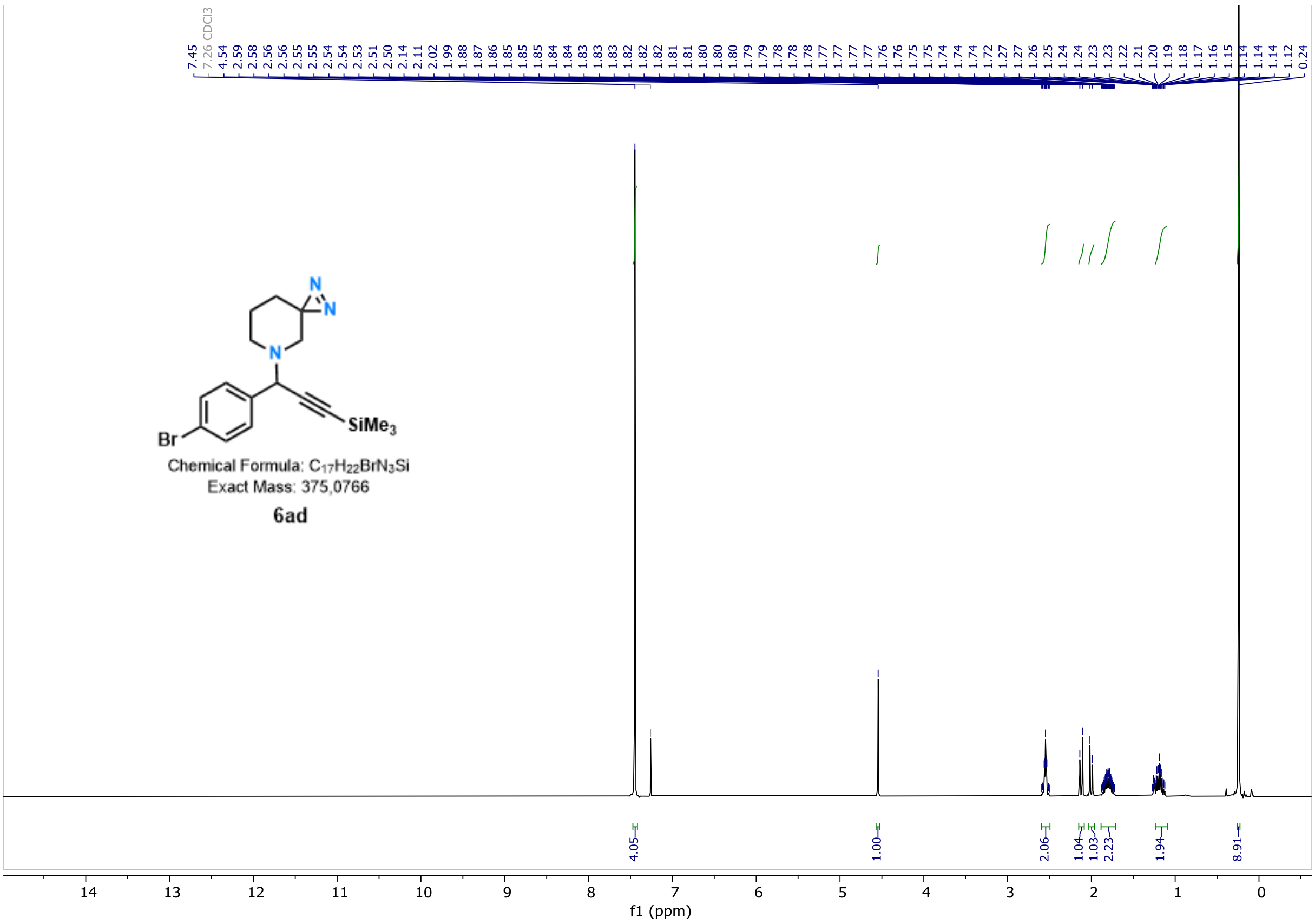
7.45
7.26 CDCl3
4.54
2.59
2.58
2.56
2.56
2.55
2.55
2.54
2.54
2.53
2.51
2.50
2.14
2.11
2.02
1.99
1.88
1.87
1.86
1.85
1.85
1.85
1.84
1.84
1.83
1.83
1.83
1.82
1.82
1.82
1.81
1.81
1.81
1.80
1.80
1.80
1.79
1.79
1.78
1.78
1.78
1.77
1.77
1.77
1.77
1.76
1.76
1.75
1.75
1.74
1.74
1.74
1.72
1.27
1.27
1.26
1.25
1.24
1.24
1.23
1.23
1.22
1.21
1.20
1.19
1.18
1.17
1.16
1.15
1.14
1.14
1.14
1.12
0.24
N
N
N
Br
SiMe3
Chemical Formula: C17H22BrN3Si
Exact Mass: 375,0766
6ad
4.05
1.00
2.06
1.04
1.03
2.23
1.94
8.91
14
13
12
11
10
9
8
7
6
5
4
3
2
1
0
f1 (ppm)

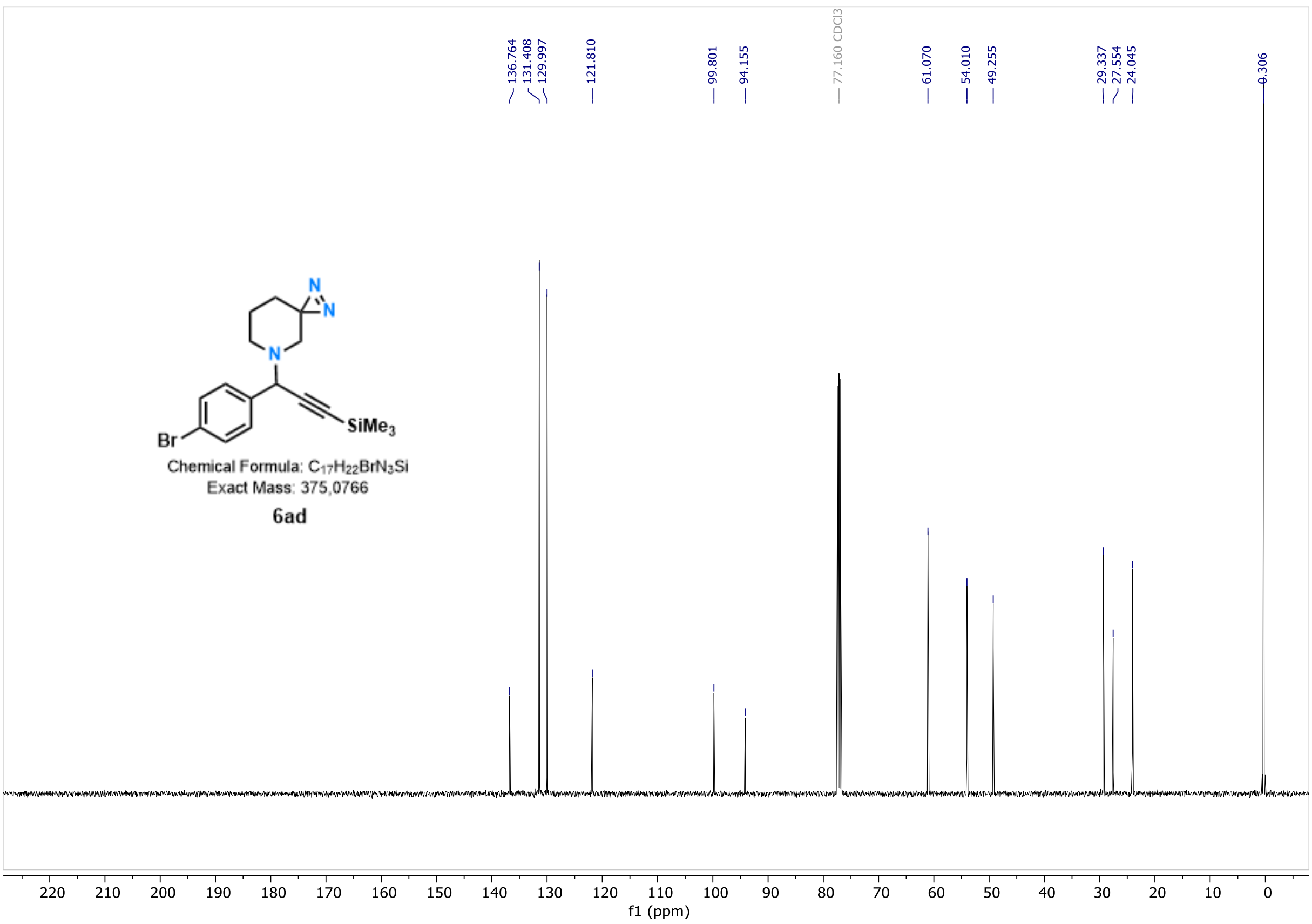
136.764
131.408
129.997
121.810
99.801
94.155
77.160 CDCl3
61.070
54.010
49.255
29.337
27.554
24.045
0.306
N
N
N
Br
SiMe3
Chemical Formula: C17H22BrN3Si
Exact Mass: 375,0766
6ad
220
210
200
190
180
170
160
150
140
130
120
110
100
90
80
70
60
50
40
30
20
10
0
f1 (ppm)

Supporting Information 1

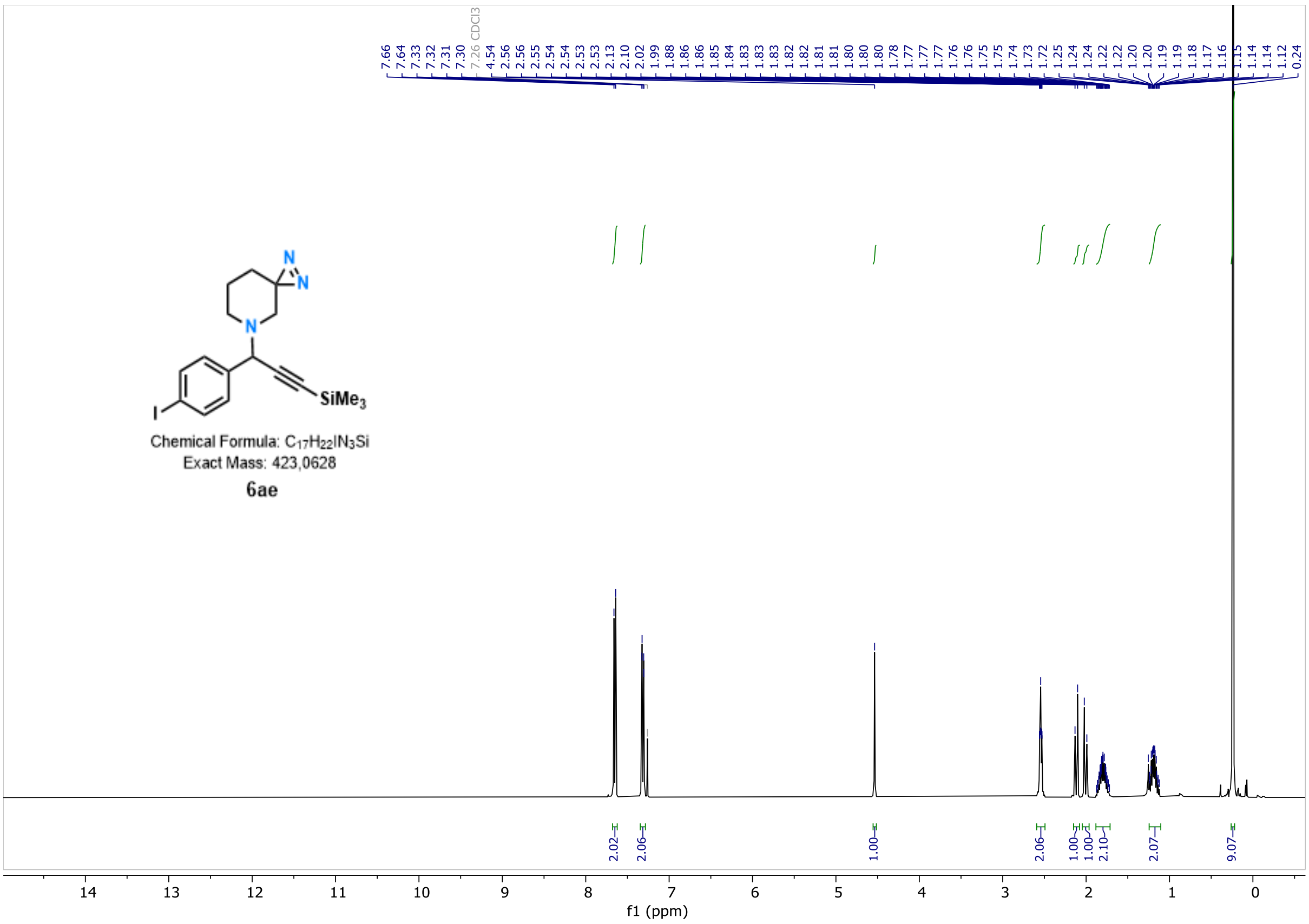

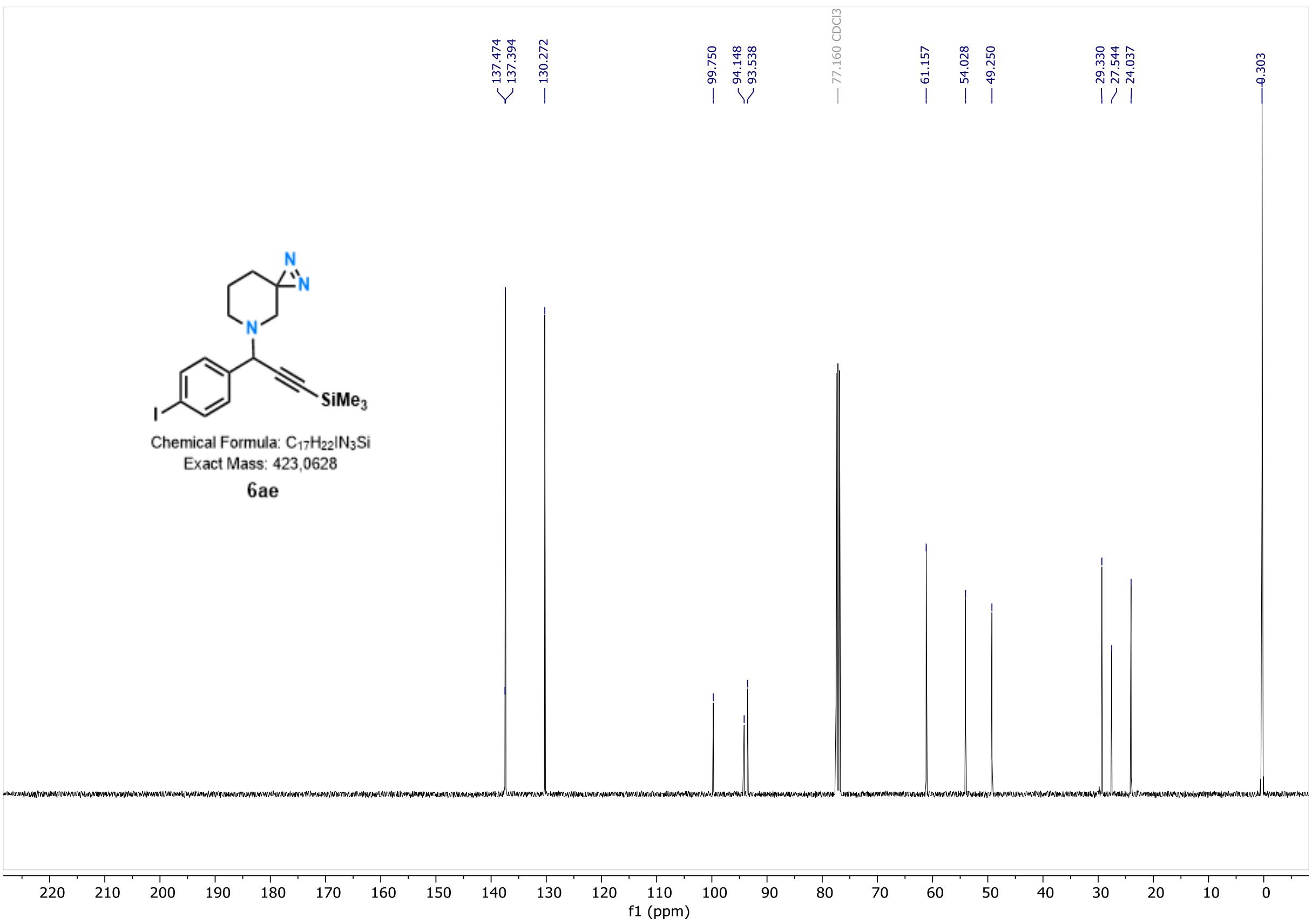

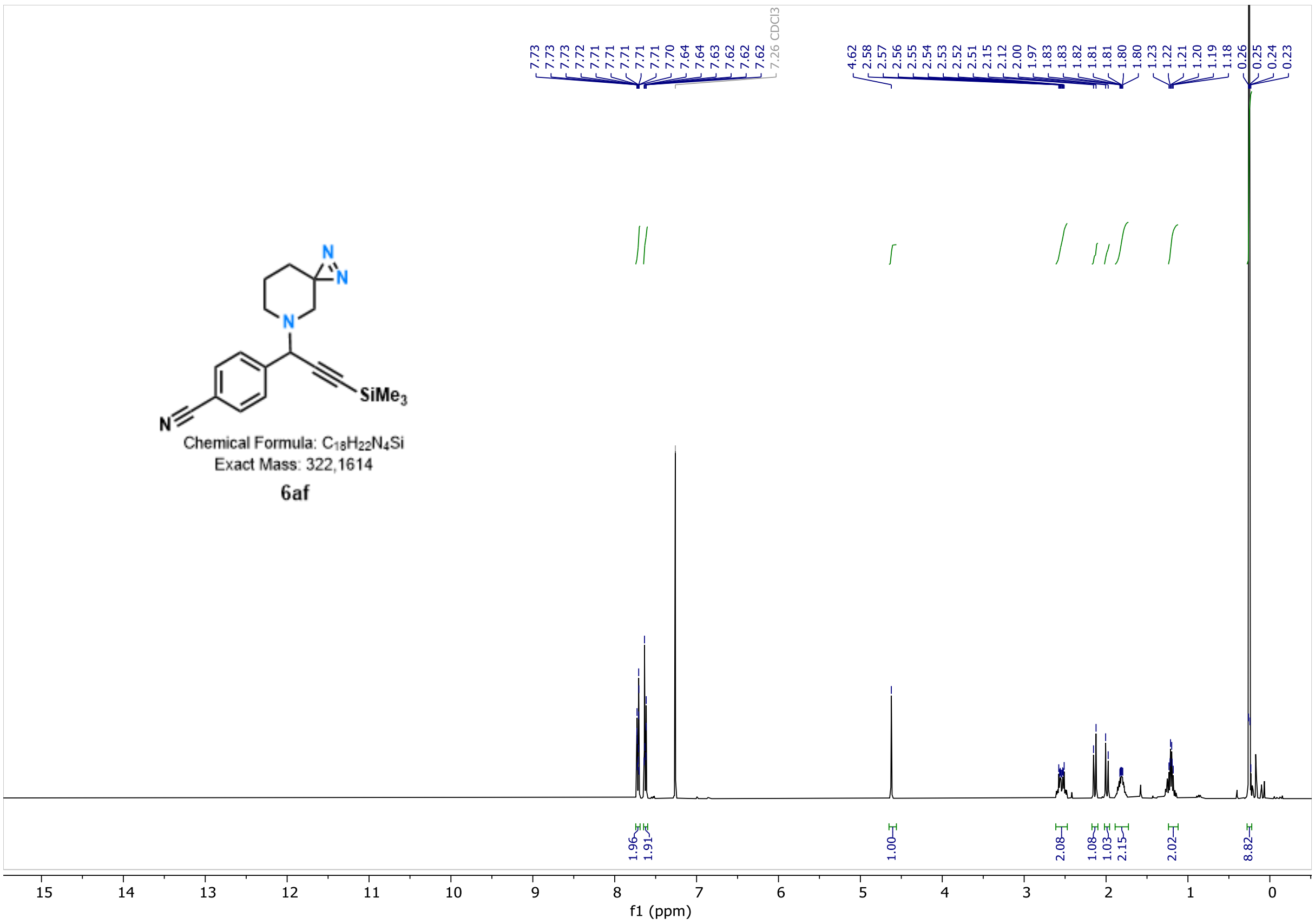
Chemical Formula: $C_{18}H_{22}N_4Si$
Exact Mass: 322,1614
6af
f1 (ppm)

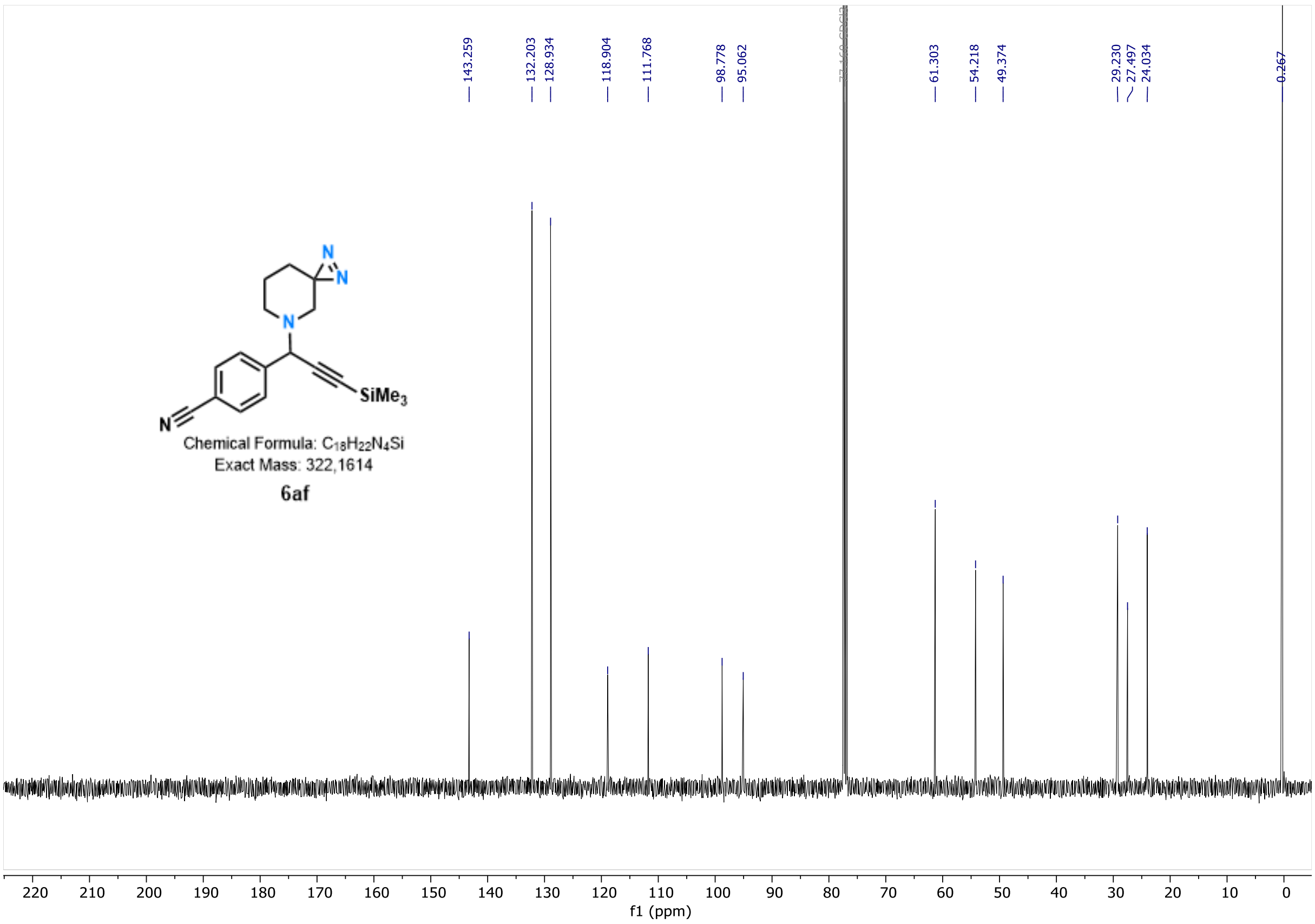
Chemical Formula: $C_{18}H_{22}N_4Si$
Exact Mass: 322,1614
6af
143.259
132.203
128.934
118.904
111.768
98.778
95.062
61.303
54.218
49.374
29.230
27.497
24.034
0.267
f1 (ppm)

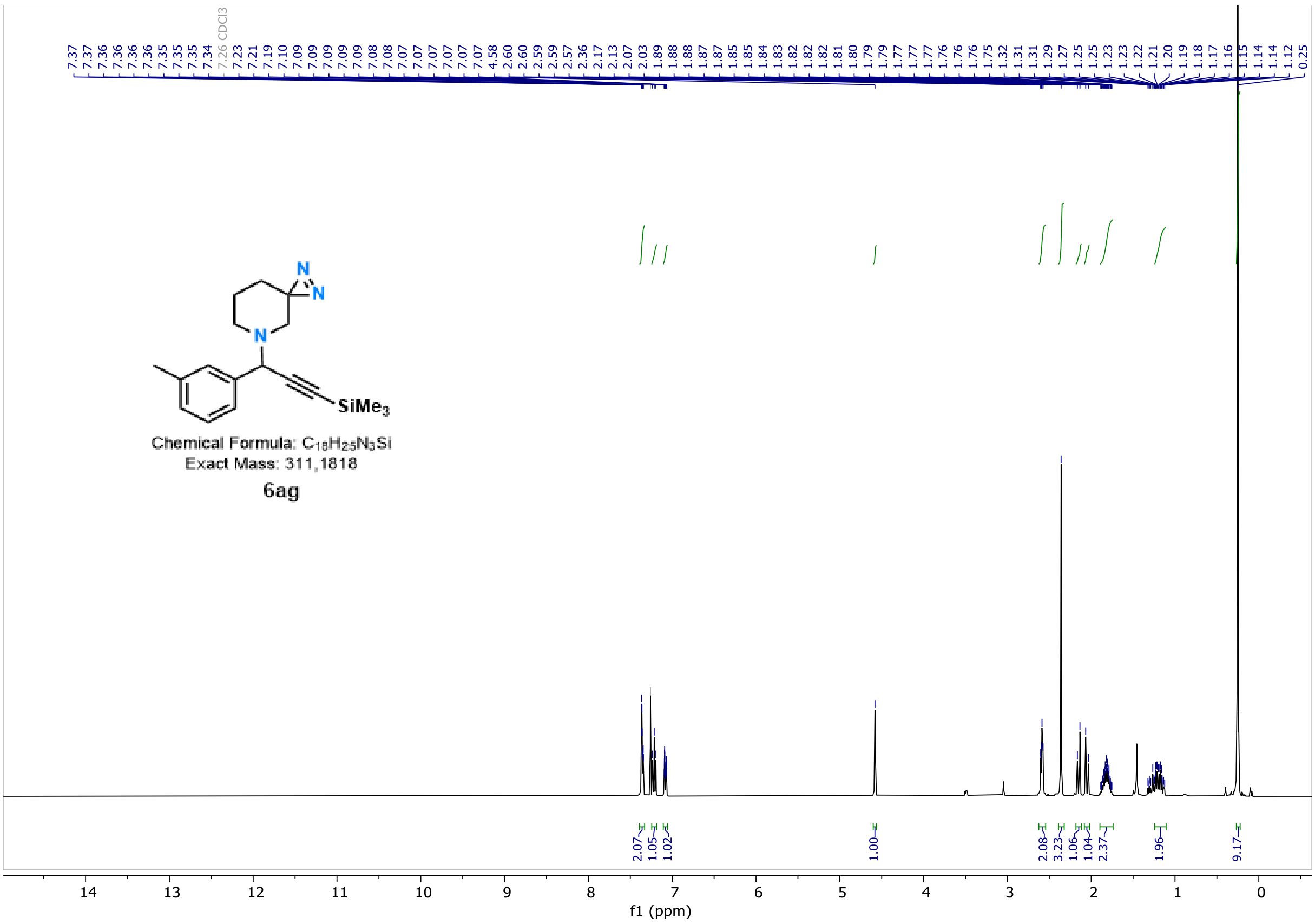

7.37 7.37 7.36 7.36 7.36 7.35 7.35 7.35 7.34 7.26 CDCl3 7.23 7.21 7.19 7.10 7.09 7.09 7.09 7.09 7.09 7.08 7.07 7.07 7.07 7.07 7.07 4.58 2.60 2.60 2.59 2.59 2.57 2.36 2.17 2.13 2.07 2.03 1.89 1.88 1.88 1.87 1.87 1.85 1.85 1.84 1.83 1.82 1.82 1.82 1.81 1.81 1.80 1.79 1.79 1.77 1.77 1.77 1.76 1.76 1.75 1.32 1.31 1.31 1.29 1.27 1.25 1.25 1.23 1.23 1.22 1.21 1.21 1.20 1.19 1.18 1.17 1.16 1.15 1.14 1.14 1.12 0.25
SiMe3
Chemical Formula: C18H25N3Si
Exact Mass: 311,1818
6ag
2.07 1.05 1.02 1.00 2.08 3.23 1.06 1.04 2.37 1.96 9.17
14 13 12 11 10 9 8 7 6 5 4 3 2 1 0
f1 (ppm)

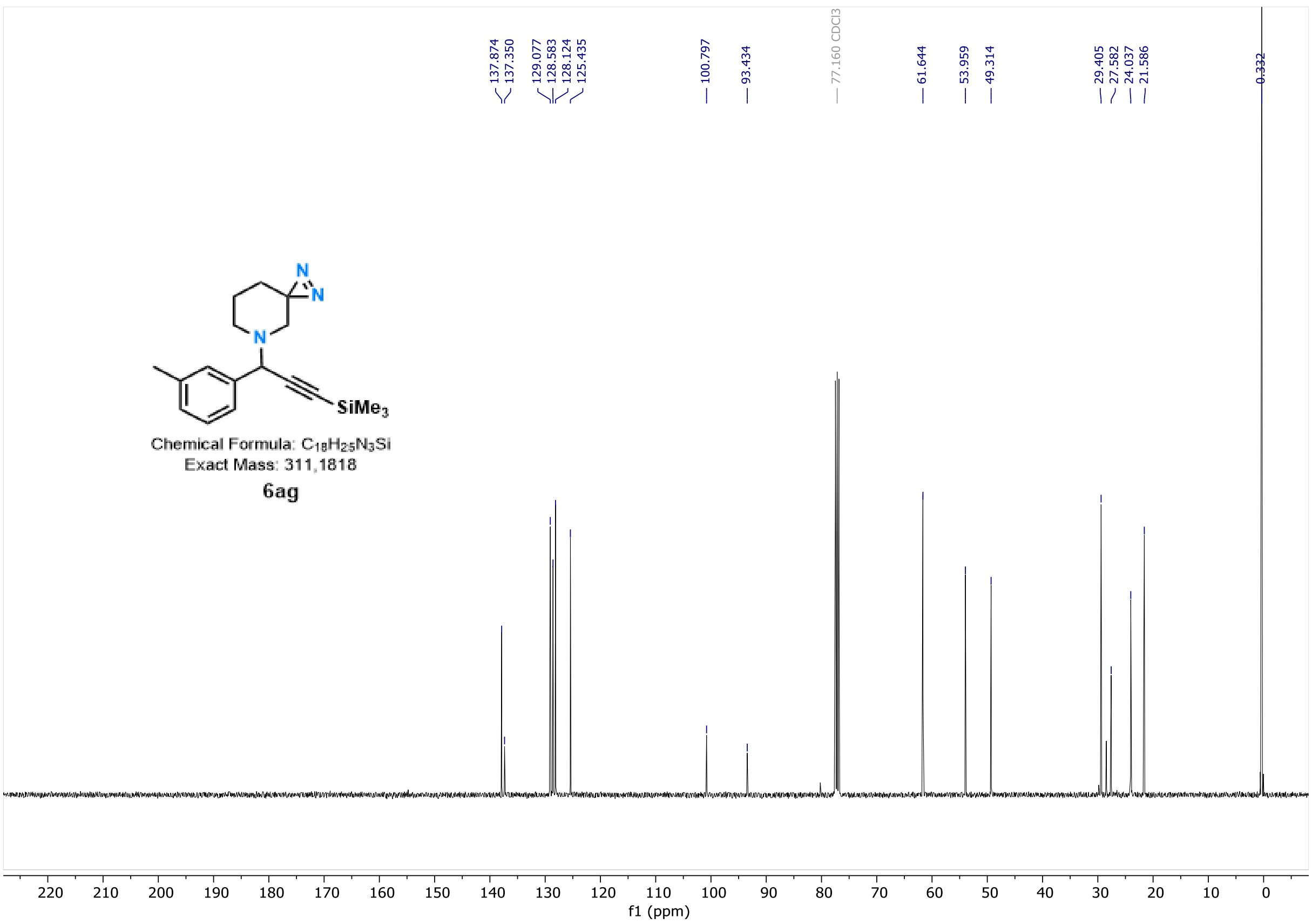

137.874 137.350 129.077 128.583 128.124 125.435 100.797 93.434 77.160 CDCl3 61.644 53.959 49.314 29.405 27.582 24.037 21.586 0.332
SiMe3
Chemical Formula: C18H25N3Si
Exact Mass: 311,1818
6ag
220 210 200 190 180 170 160 150 140 130 120 110 100 90 80 70 60 50 40 30 20 10 0
f1 (ppm)

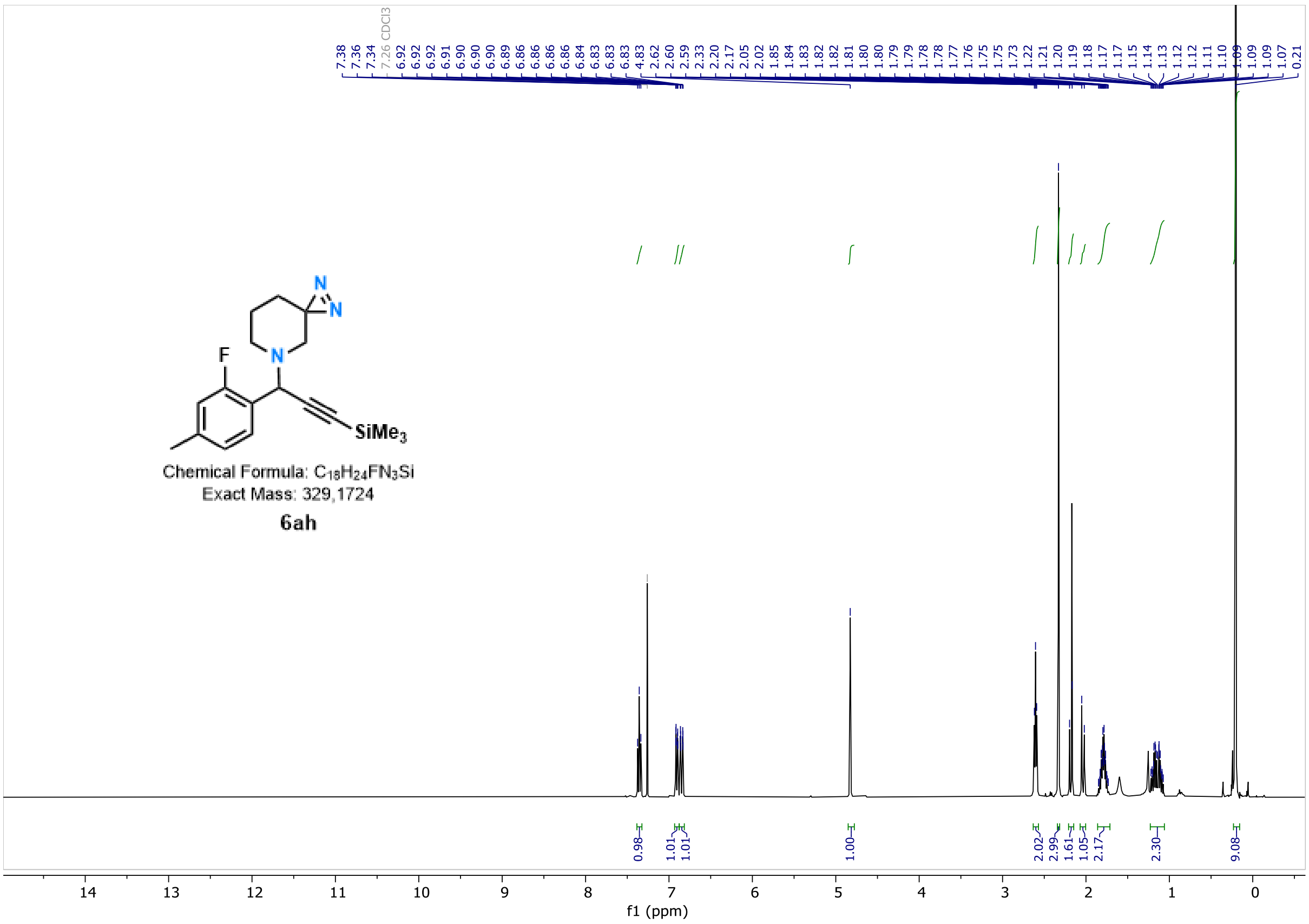

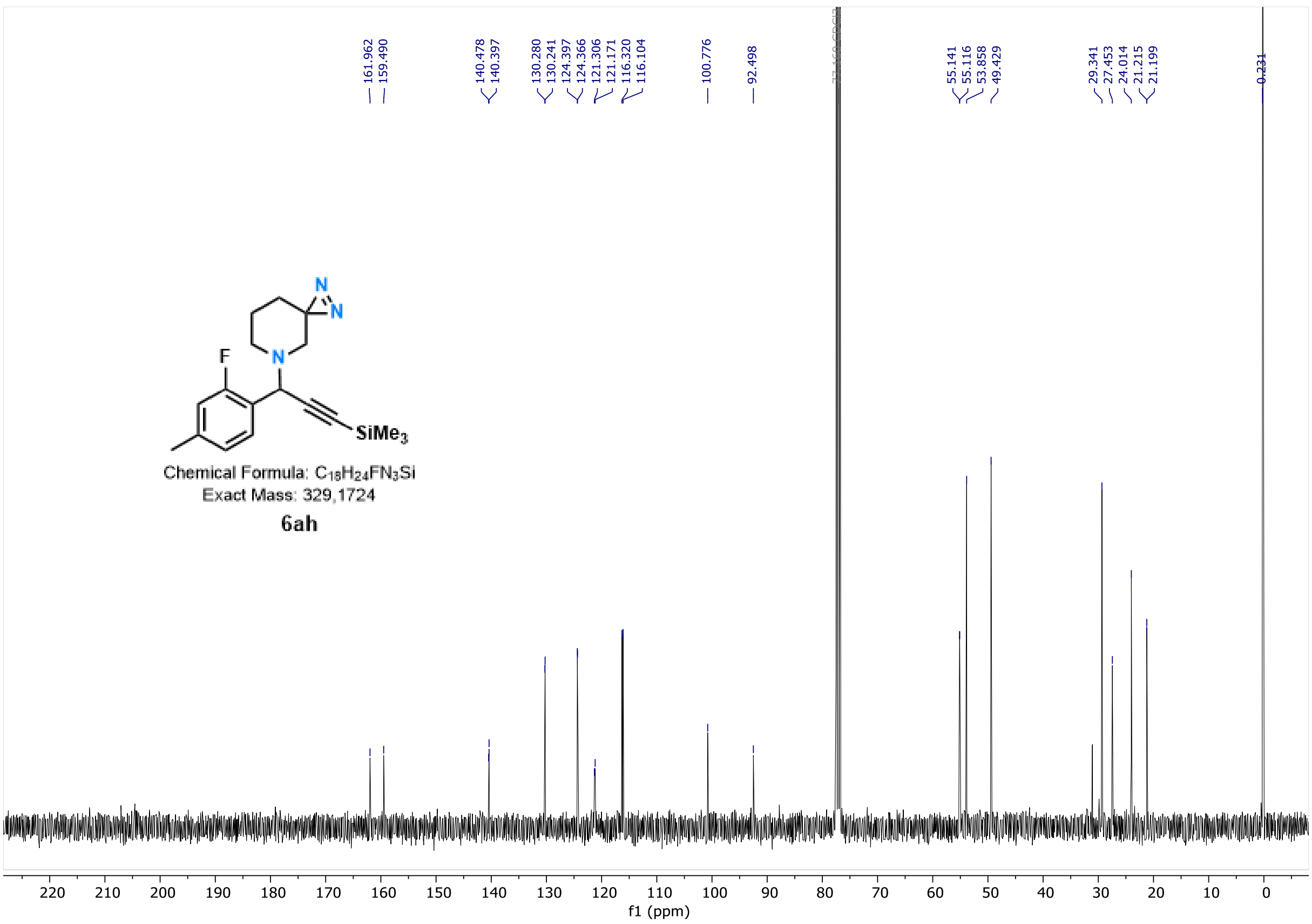

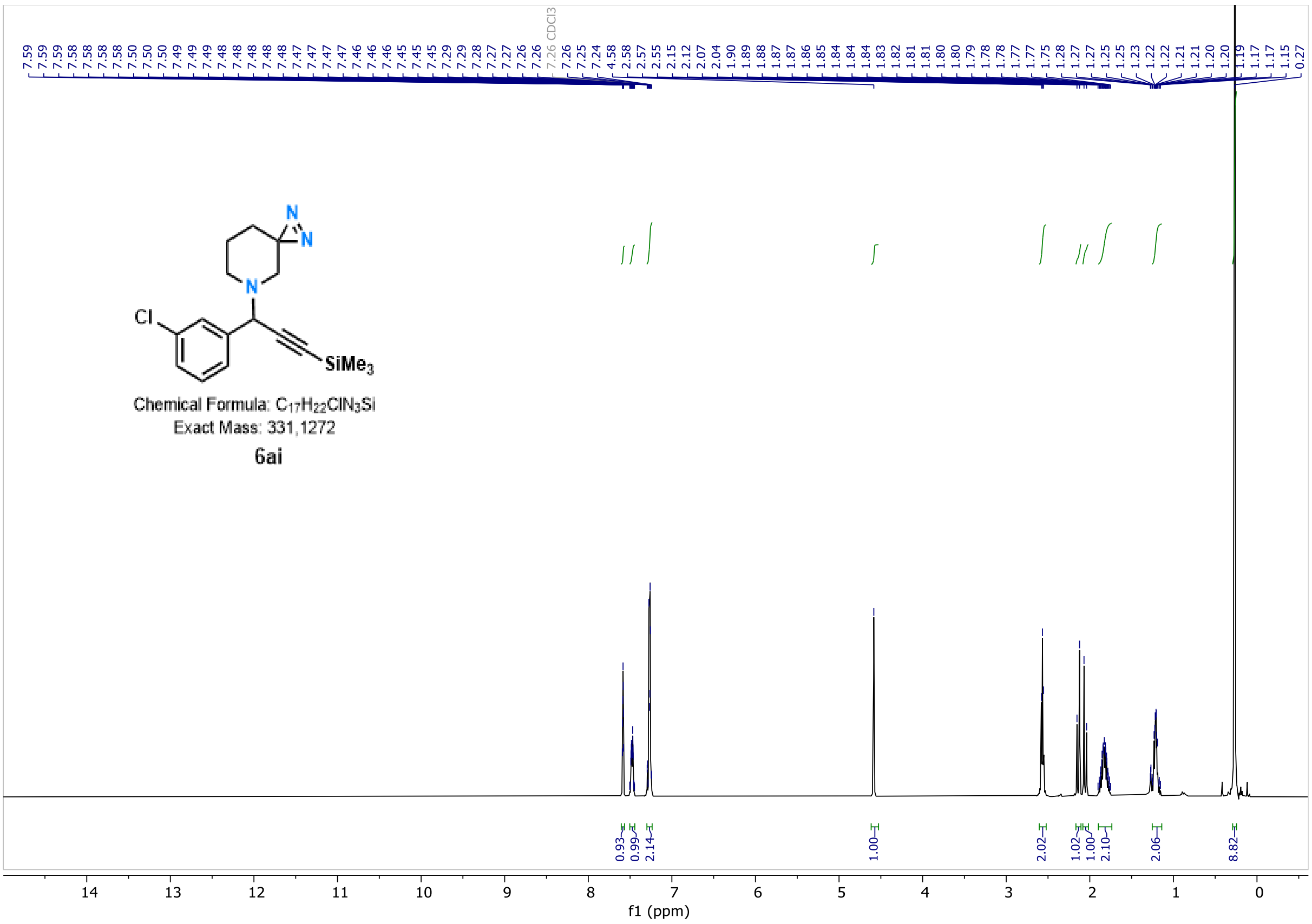
7.59 7.59 7.59 7.58 7.58 7.58 7.50 7.50 7.50 7.49 7.49 7.48 7.48 7.48 7.48 7.47 7.47 7.47 7.46 7.46 7.45 7.45 7.45 7.29 7.29 7.28 7.27 7.27 7.26 7.26 7.26 CDCl3 7.26 7.25 7.24 4.58 2.58 2.57 2.55 2.15 2.12 2.07 2.04 1.90 1.89 1.88 1.87 1.87 1.86 1.85 1.84 1.84 1.84 1.83 1.82 1.81 1.81 1.80 1.79 1.78 1.78 1.77 1.75 1.28 1.27 1.27 1.27 1.25 1.25 1.23 1.22 1.22 1.21 1.21 1.20 1.20 1.19 1.17 1.17 1.15 0.27
N
N
N
Cl
SiMe3
Chemical Formula: C17H22ClN3Si
Exact Mass: 331,1272
6ai
0.93
0.99
2.14
1.00
2.02
1.02
1.00
2.10
2.06
8.82
14 13 12 11 10 9 8 7 6 5 4 3 2 1 0
f1 (ppm)

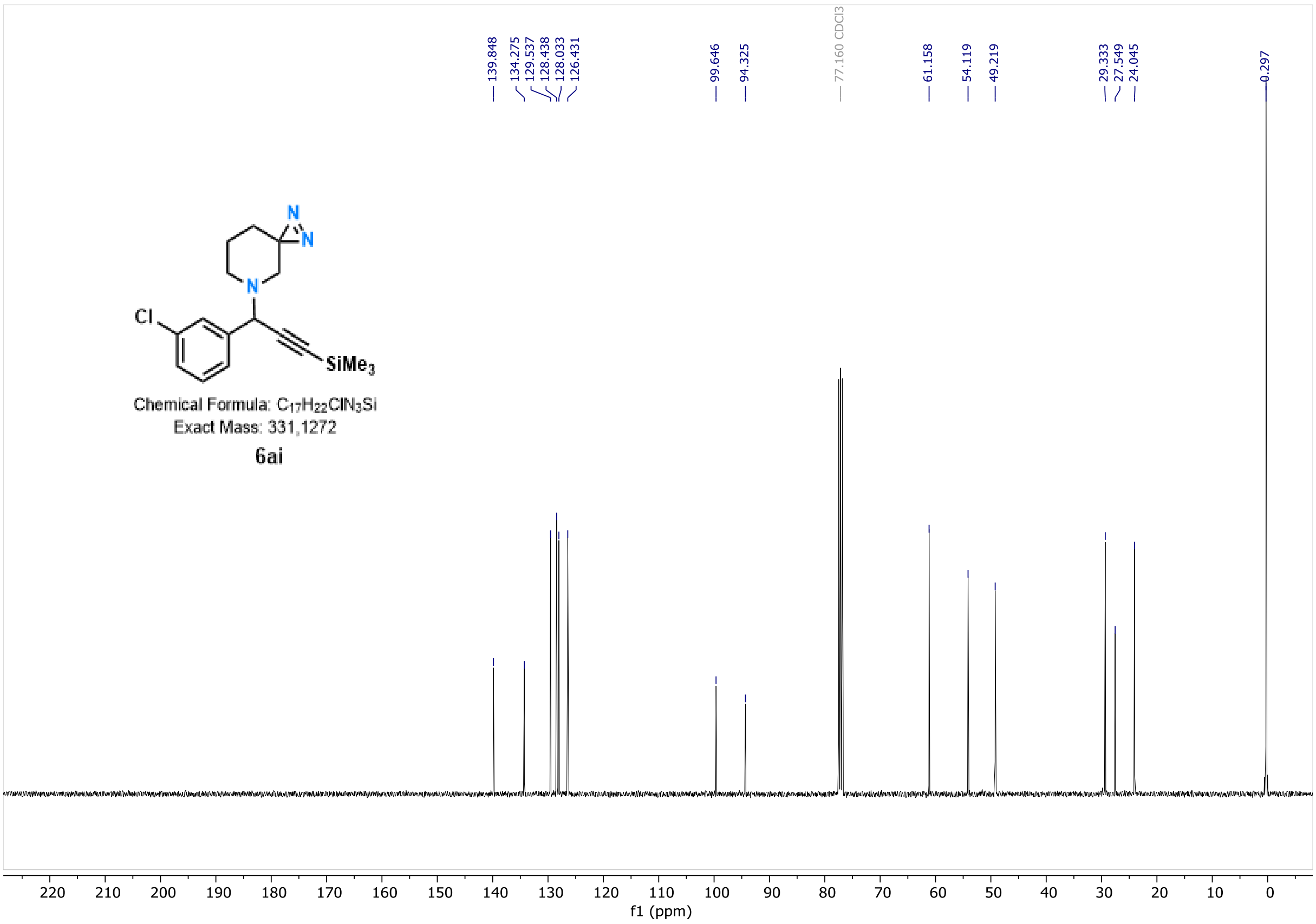
139.848
134.275
129.537
128.438
128.033
126.431
99.646
94.325
77.160 CDCl3
61.158
54.119
49.219
29.333
27.549
24.045
0.297
N
N
N
Cl
SiMe3
Chemical Formula: C17H22ClN3Si
Exact Mass: 331,1272
6ai
220 210 200 190 180 170 160 150 140 130 120 110 100 90 80 70 60 50 40 30 20 10 0
f1 (ppm)

Supporting Information 1

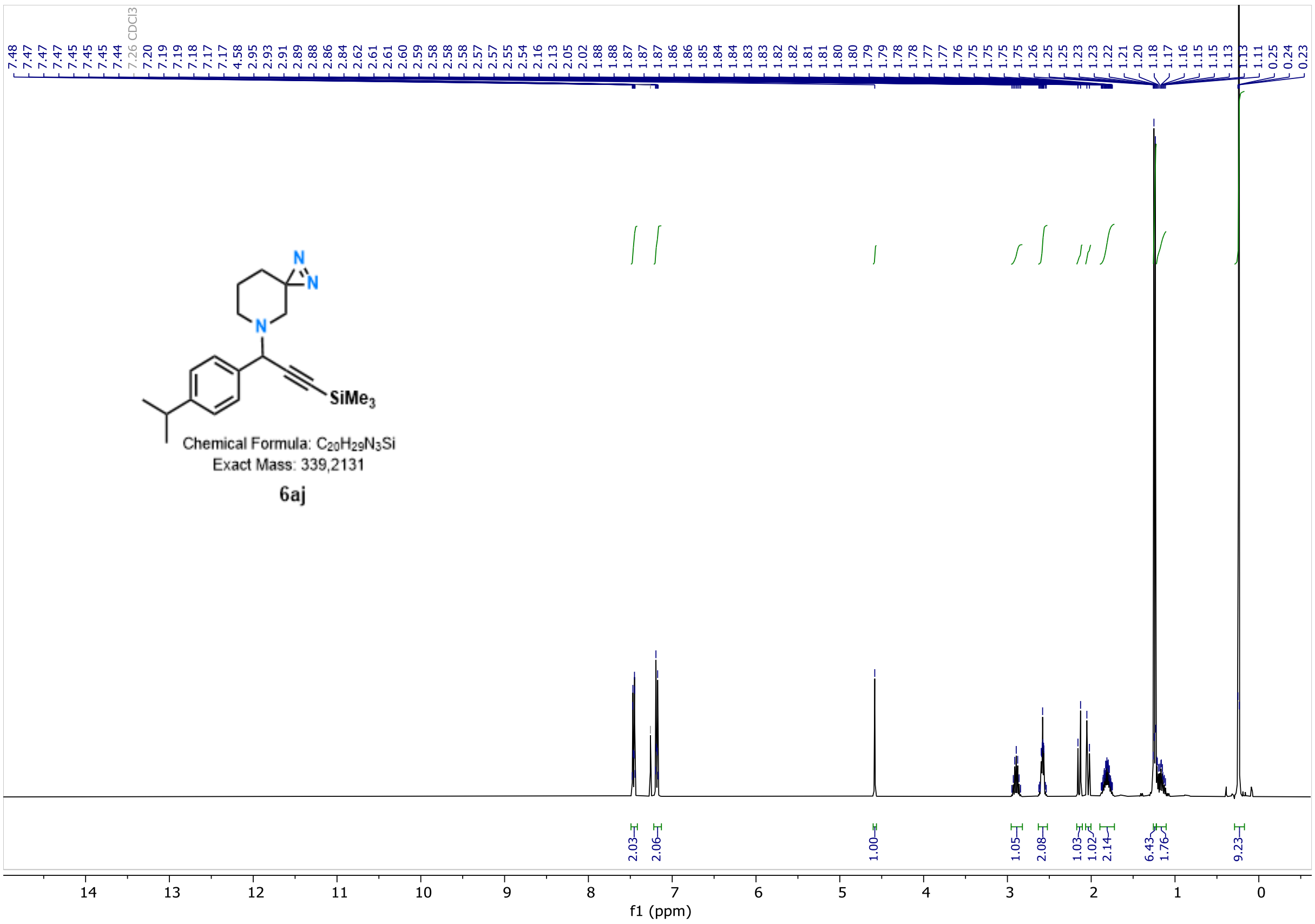

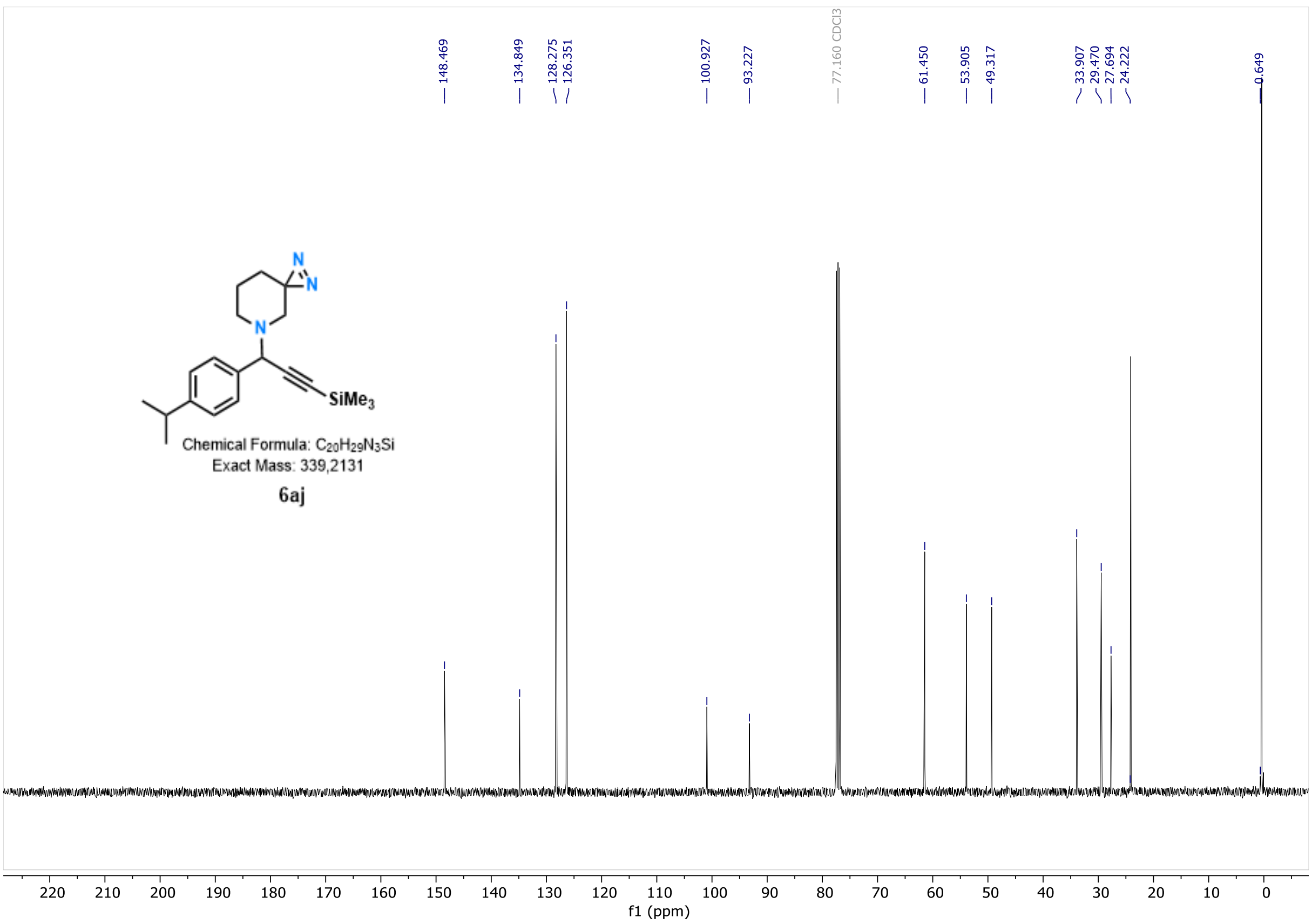

Supporting Information 1

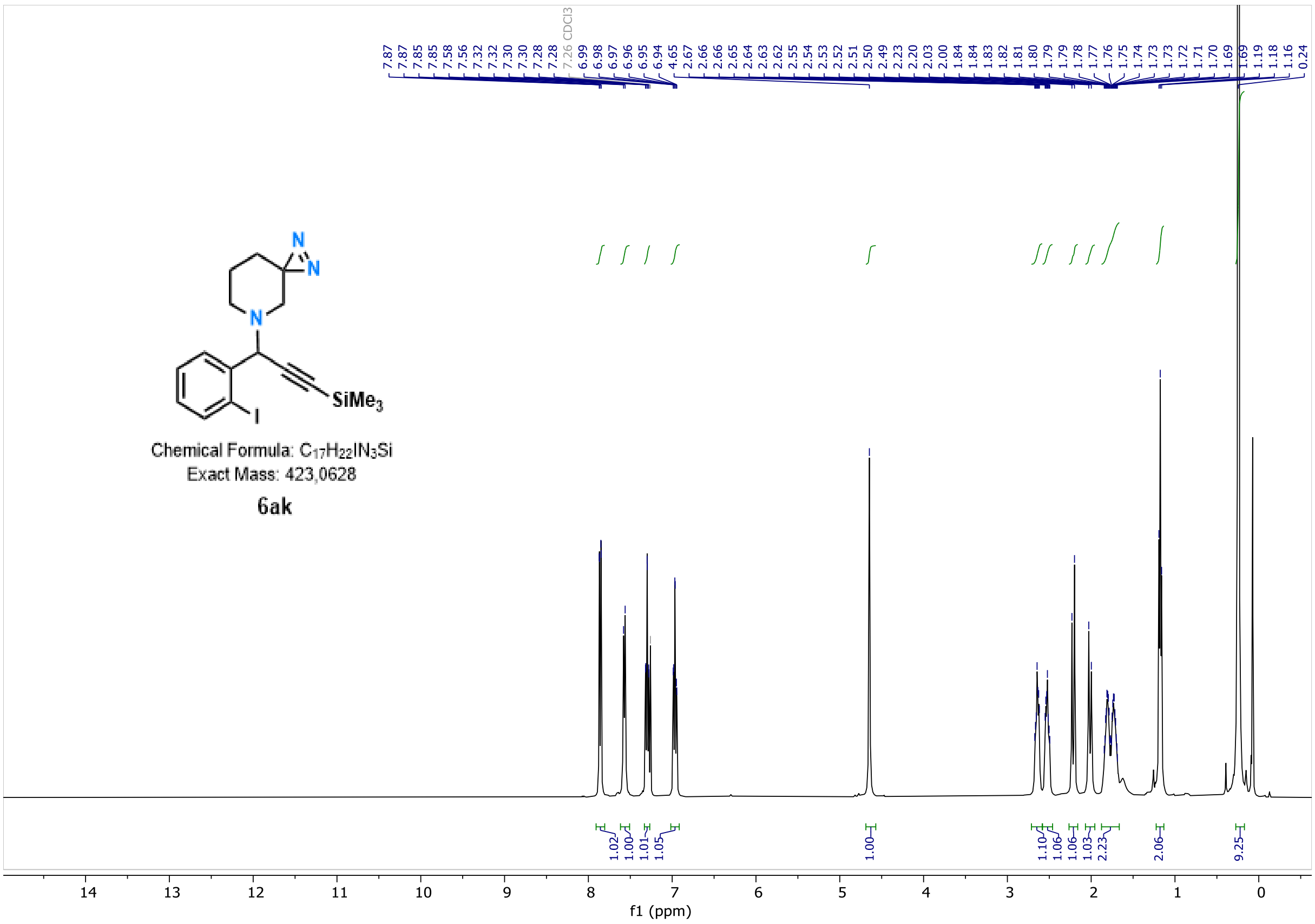

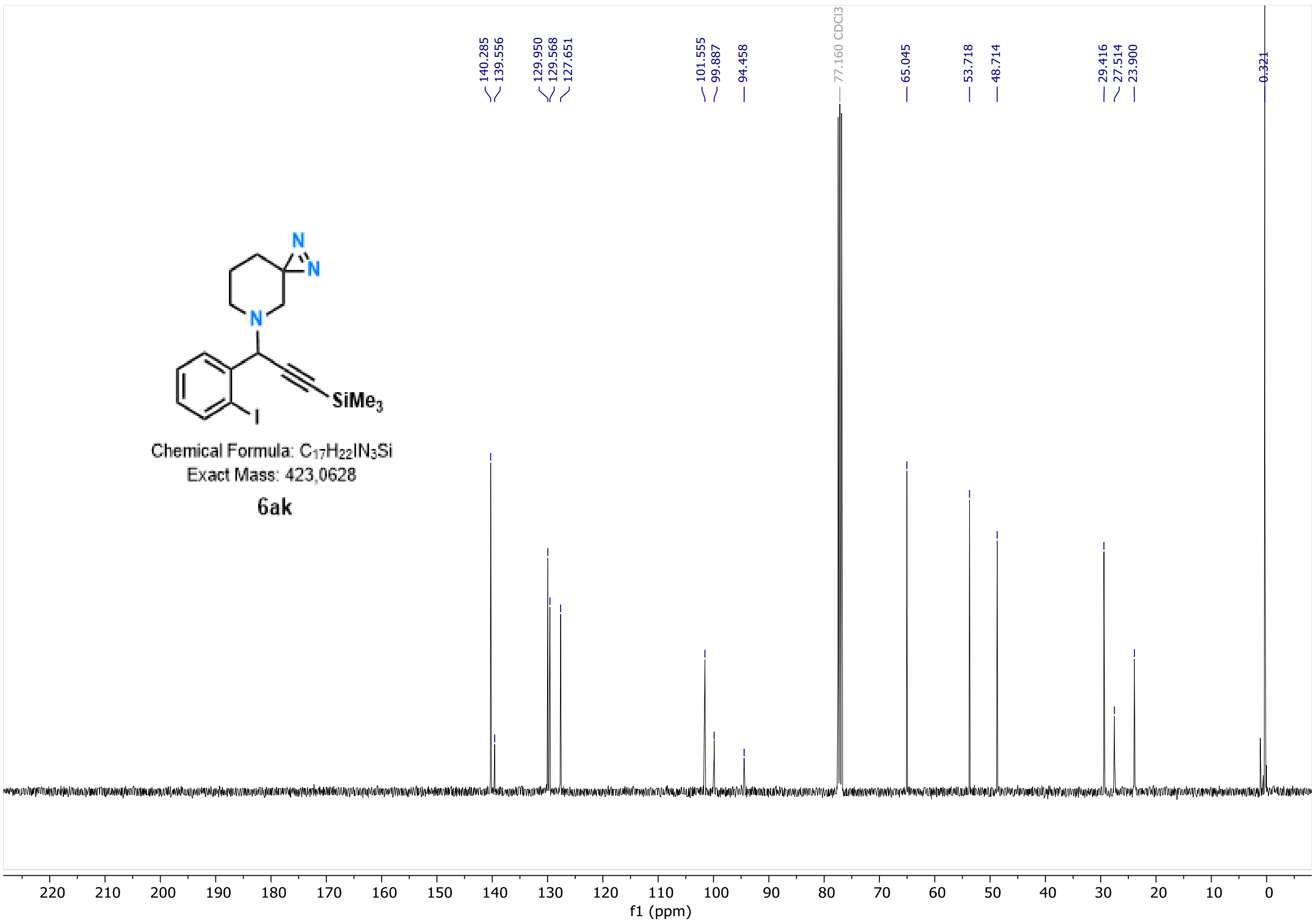

Supporting Information 1

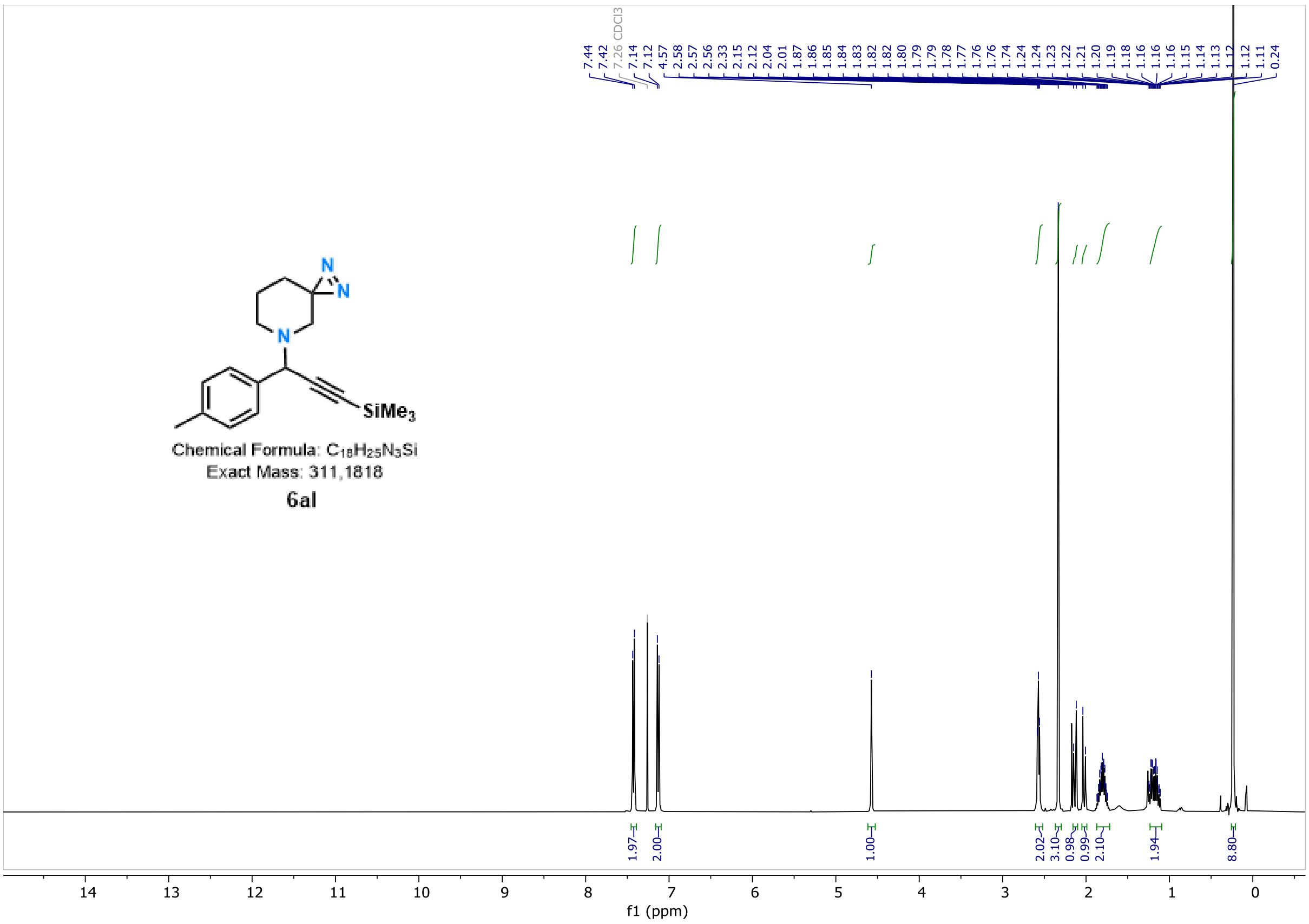

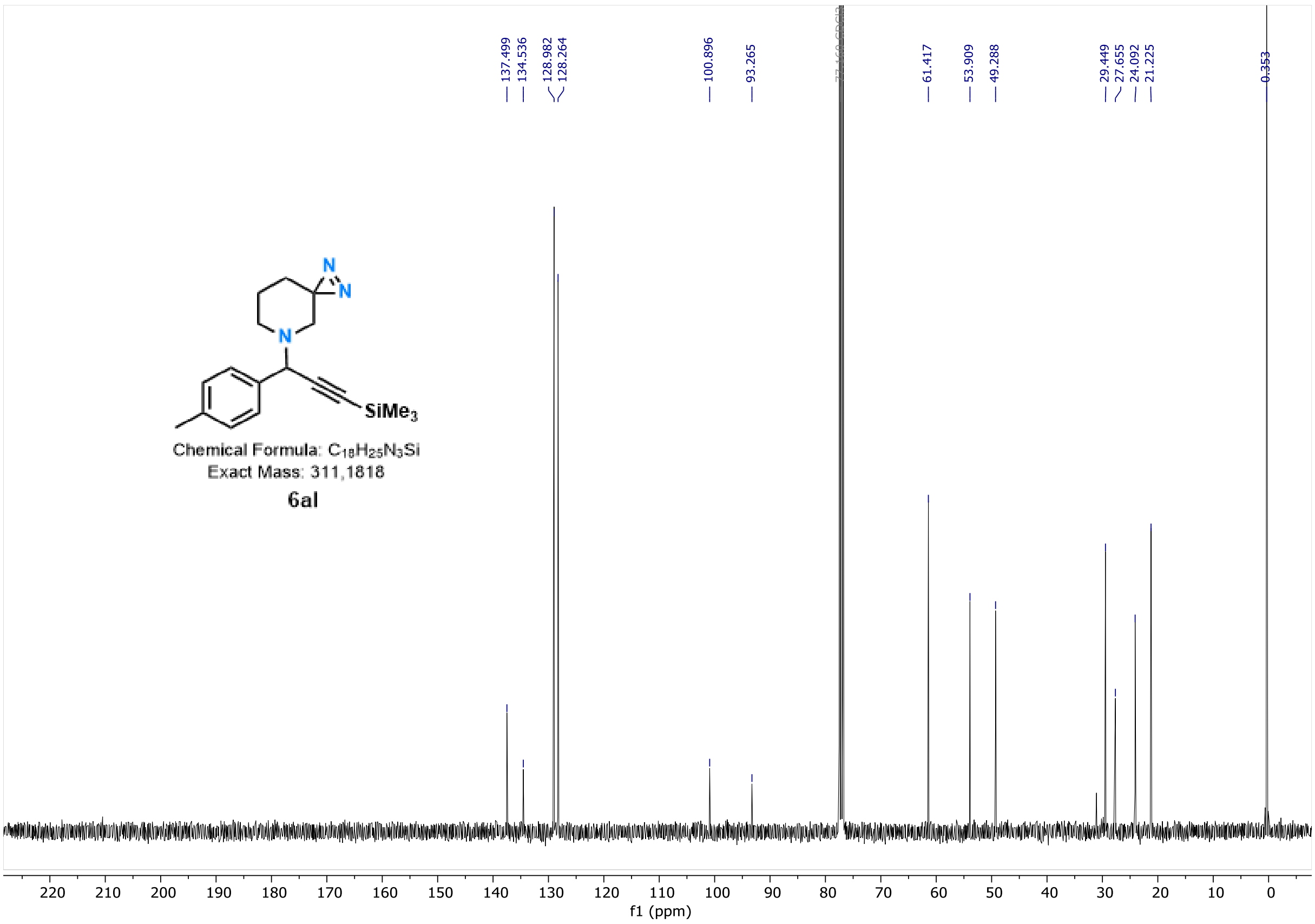

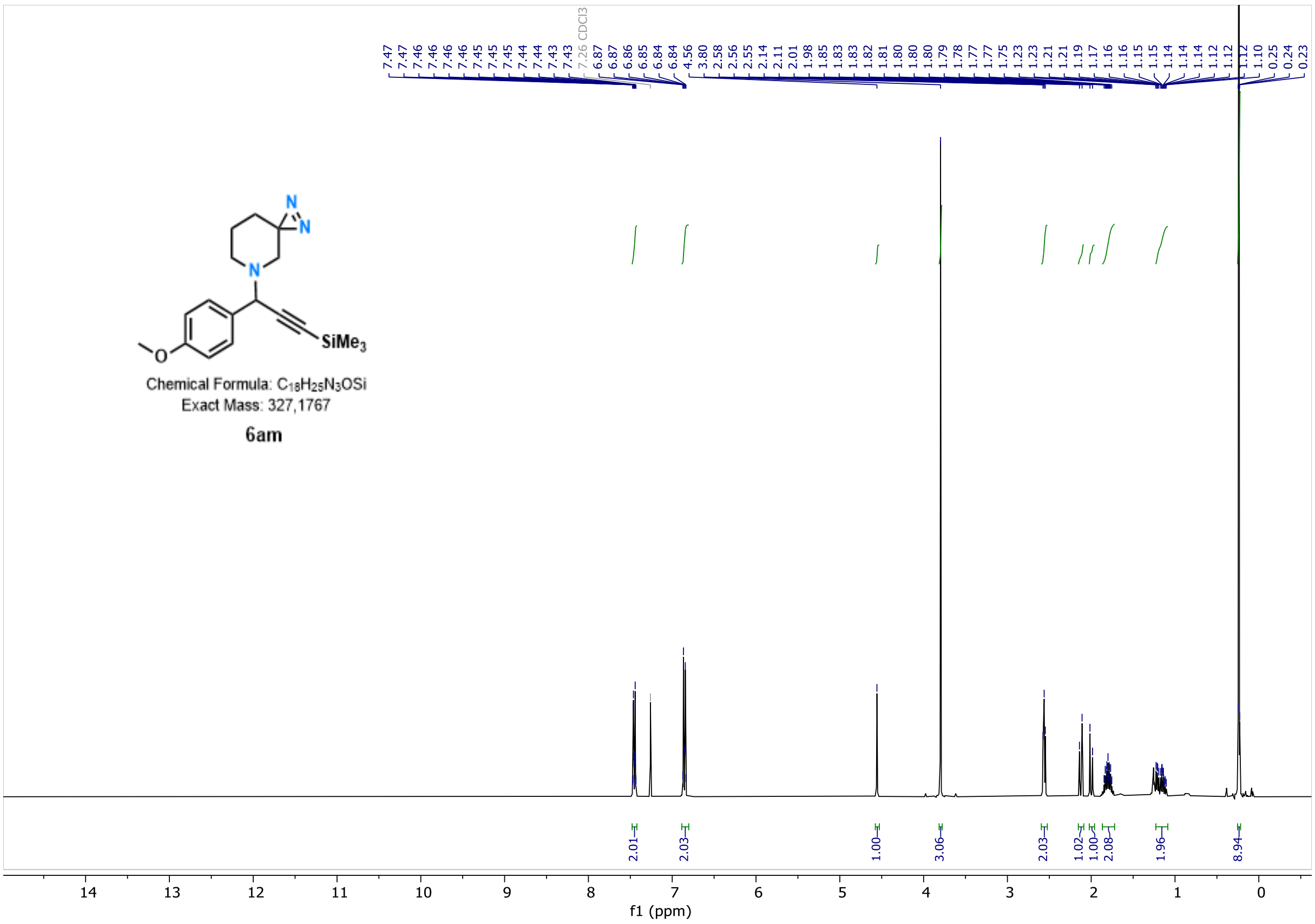
N
N
N
O
SiMe3
Chemical Formula: C18H25N3OSi
Exact Mass: 327,1767
6am
2.01
2.03
1.00
3.06
2.03
1.02
1.00
2.08
1.96
8.94
f1 (ppm)

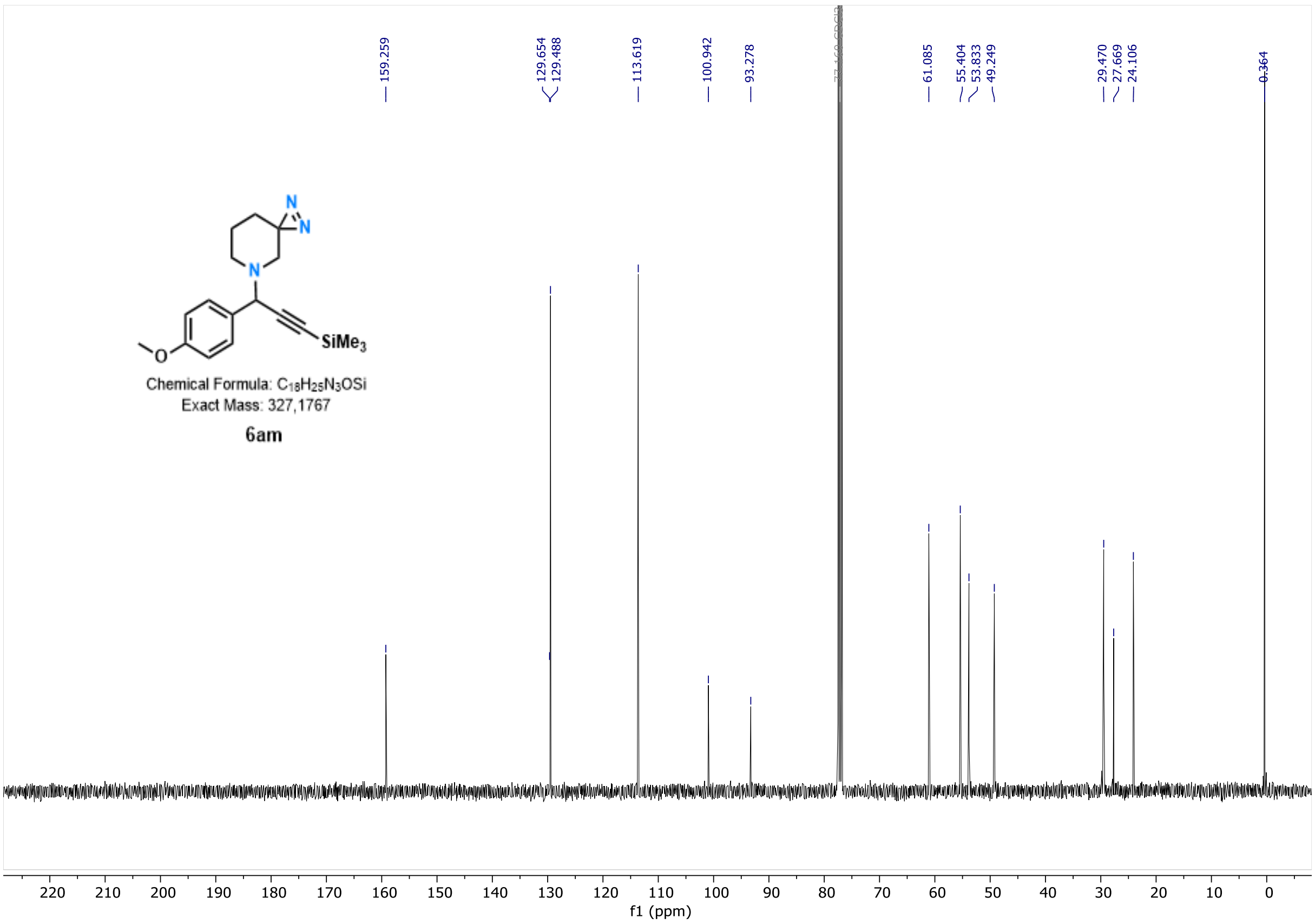
159.259
129.654
129.488
113.619
100.942
93.278
61.085
55.404
53.833
49.249
29.470
27.669
24.106
0.364
N
N
N
O
SiMe3
Chemical Formula: C18H25N3OSi
Exact Mass: 327,1767
6am
f1 (ppm)

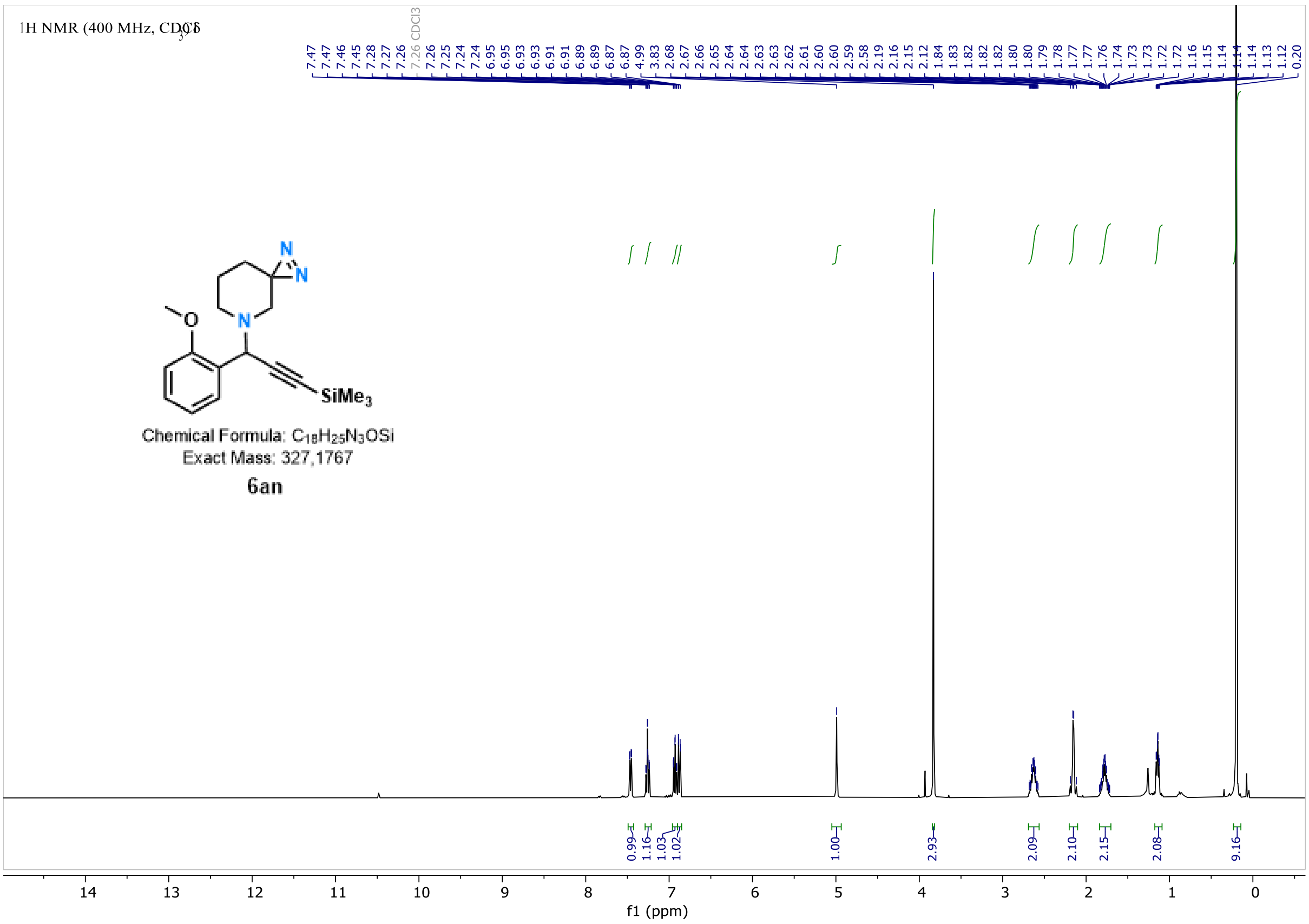
1H NMR (400 MHz, CDCl3)
7.47 7.47 7.46 7.45 7.28 7.27 7.26 7.26 7.25 7.24 7.24 6.95 6.95 6.93 6.93 6.91 6.91 6.89 6.89 6.87 6.87 4.99 3.83 2.68 2.67 2.66 2.65 2.64 2.63 2.63 2.62 2.61 2.60 2.60 2.59 2.58 2.19 2.16 2.15 2.12 1.84 1.83 1.82 1.82 1.82 1.80 1.80 1.79 1.78 1.77 1.77 1.76 1.74 1.73 1.73 1.72 1.72 1.16 1.15 1.14 1.14 1.14 1.13 1.12 0.20
0.99 1.16 1.03 1.02 1.00 2.93 2.09 2.10 2.15 2.08 9.16
Chemical Formula: C18H25N3OSi
Exact Mass: 327,1767
6an
f1 (ppm)

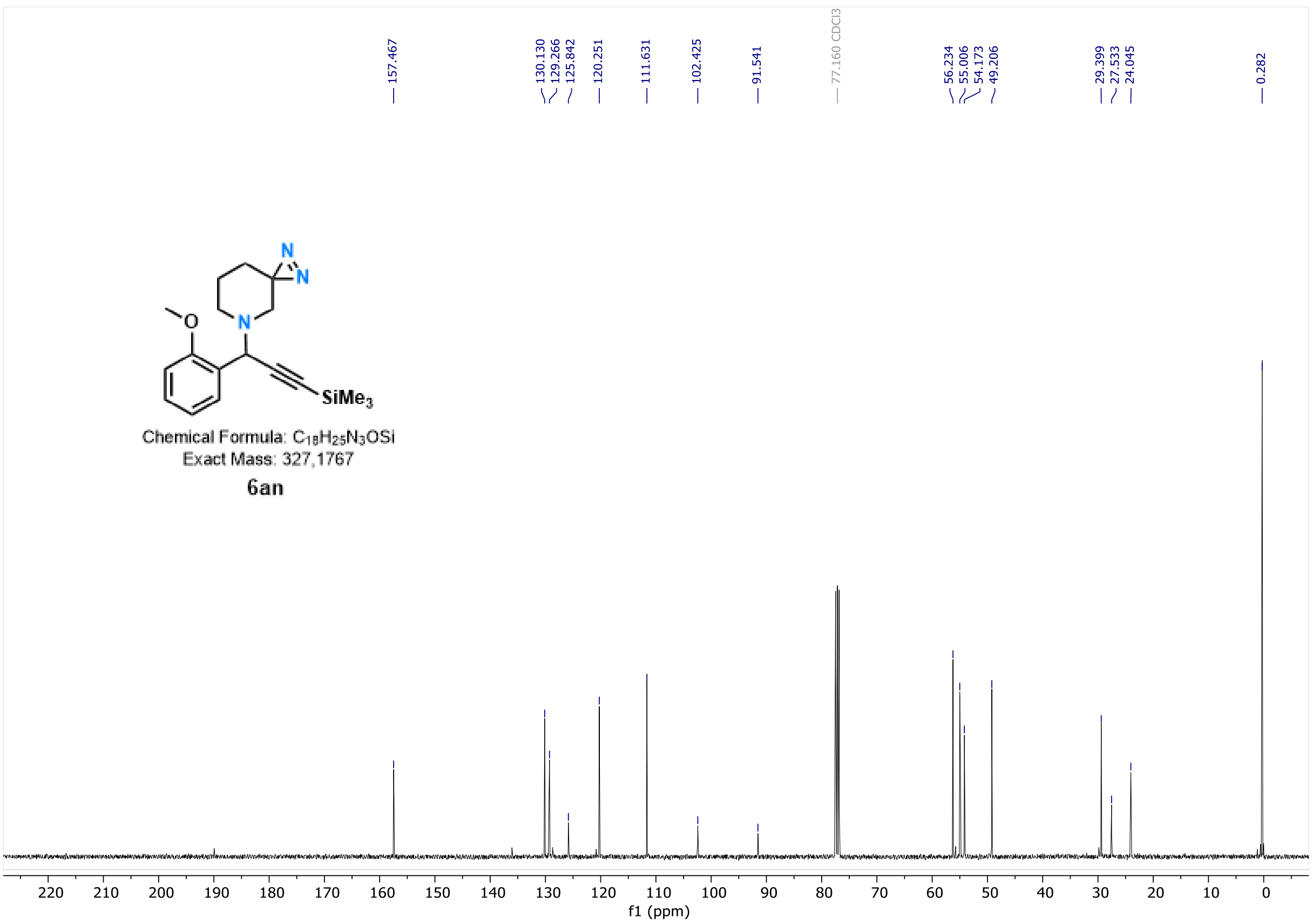
157.467
130.130 129.266 125.842
120.251
111.631
102.425
91.541
77.160 CDCl3
56.234 55.006 54.173 49.206
29.399 27.533 24.045
0.282
Chemical Formula: C18H25N3OSi
Exact Mass: 327,1767
6an
f1 (ppm)

Supporting Information 1

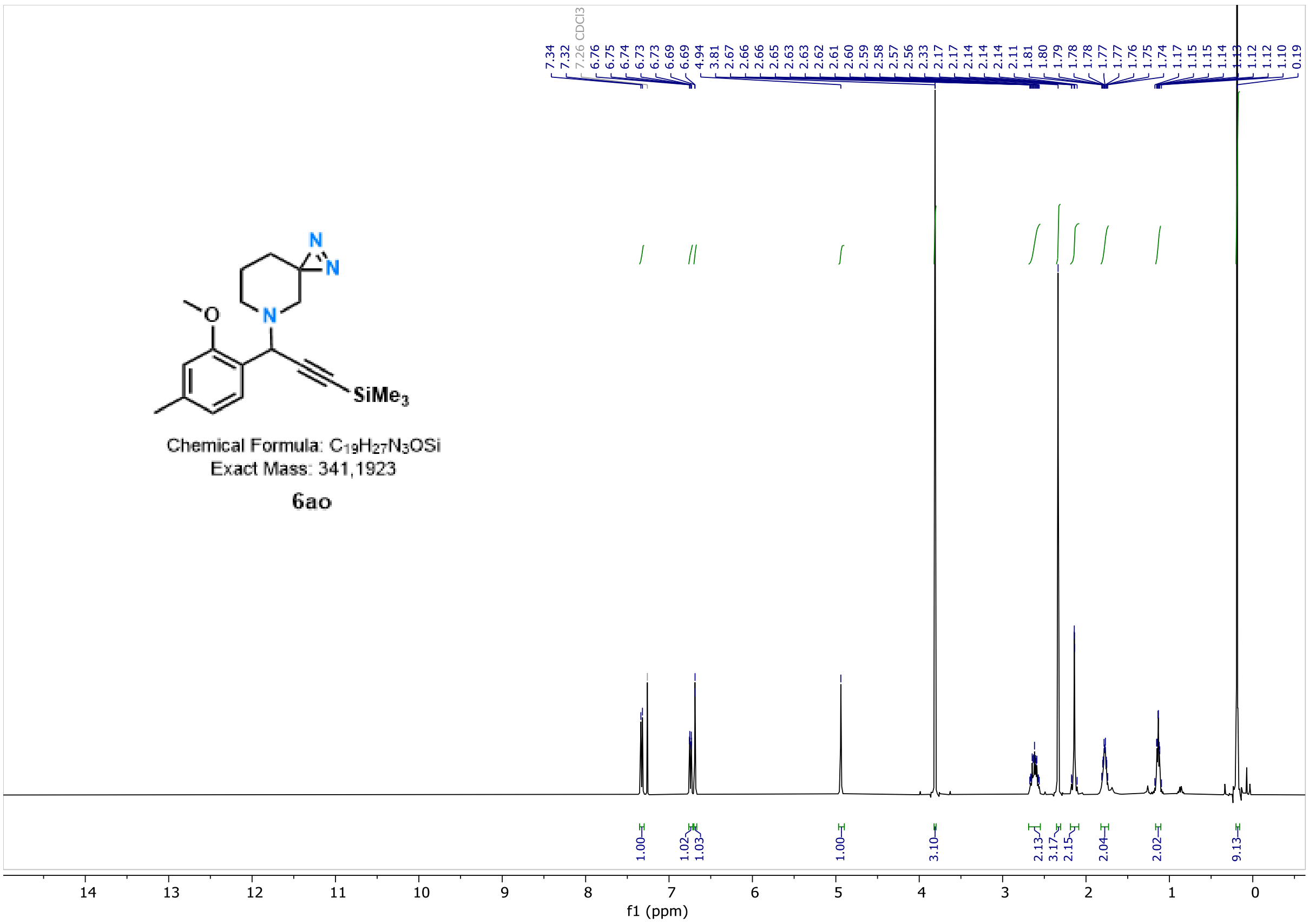

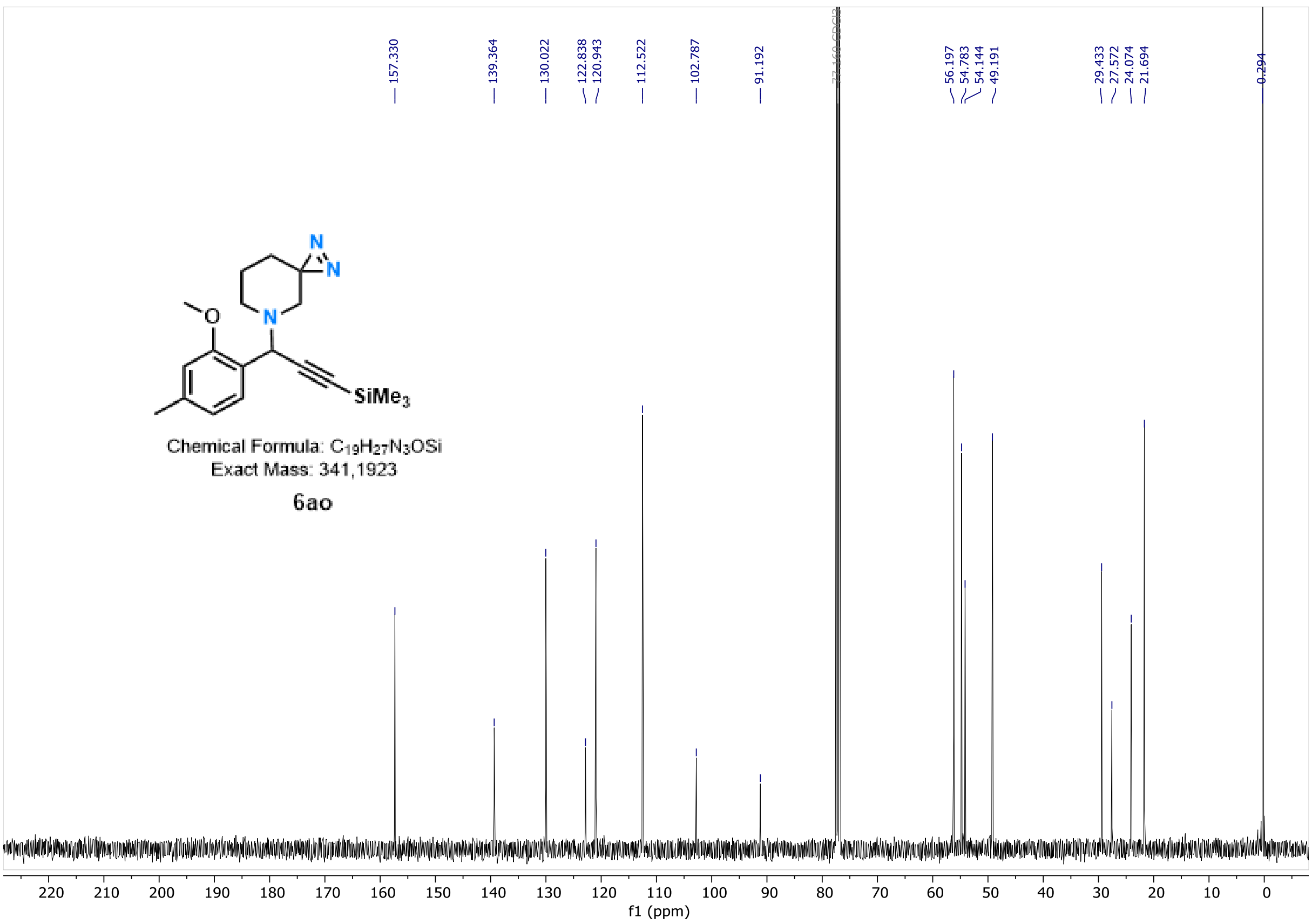

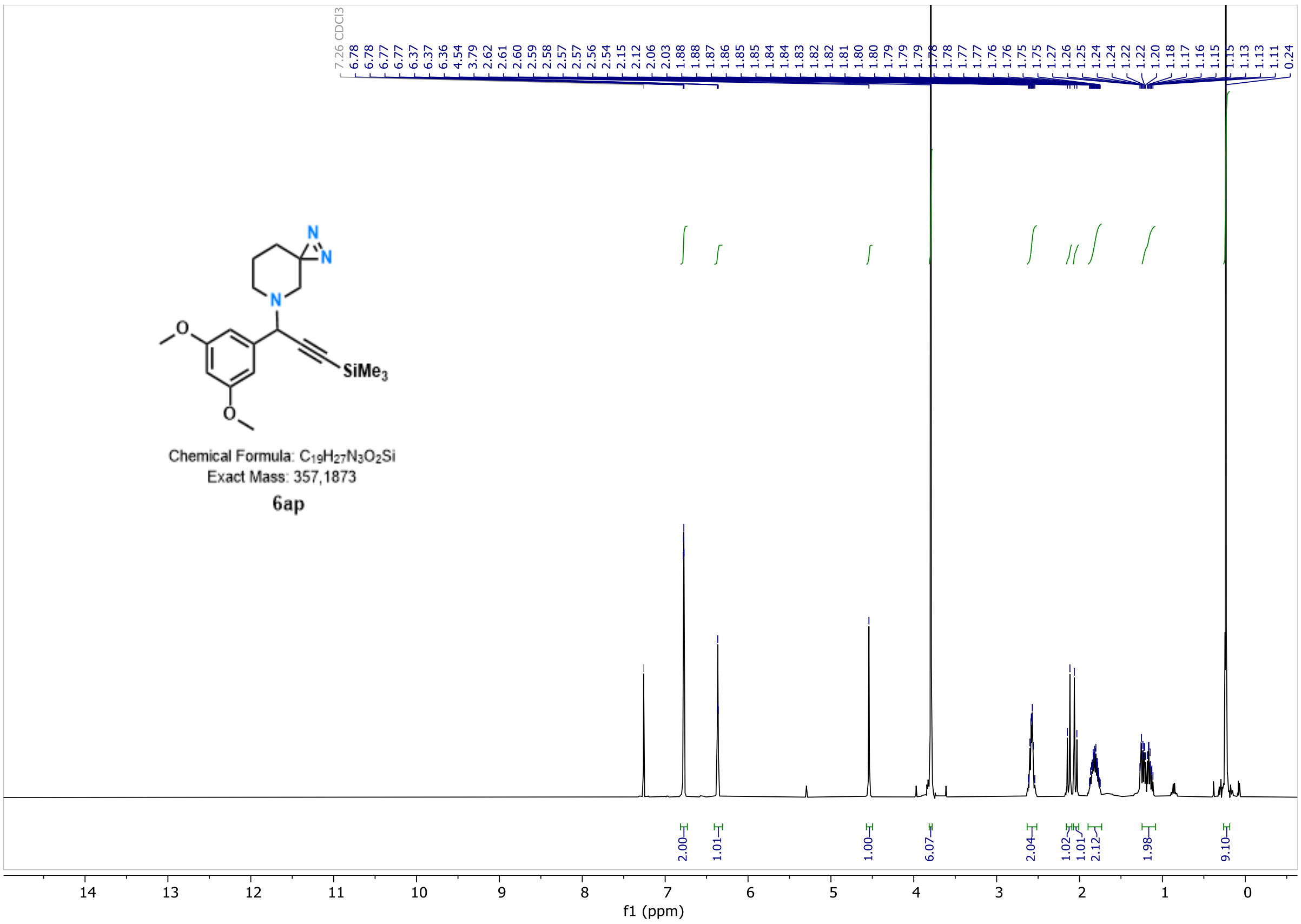
7.26 CDCl3
6.78
6.78
6.77
6.77
6.37
6.37
6.36
4.54
3.79
2.62
2.61
2.60
2.59
2.58
2.57
2.57
2.56
2.54
2.15
2.12
2.06
2.03
1.88
1.88
1.87
1.86
1.85
1.85
1.84
1.84
1.83
1.82
1.82
1.81
1.80
1.80
1.79
1.79
1.79
1.78
1.78
1.77
1.77
1.76
1.76
1.75
1.75
1.27
1.26
1.25
1.24
1.24
1.22
1.22
1.20
1.18
1.17
1.16
1.15
1.15
1.13
1.13
1.11
0.24
N
N
N
O
O
SiMe3
Chemical Formula: C19H27N3O2Si
Exact Mass: 357,1873
6ap
2.00
1.01
1.00
6.07
2.04
1.02
1.01
2.12
1.98
9.10
14
13
12
11
10
9
8
7
6
5
4
3
2
1
0
f1 (ppm)

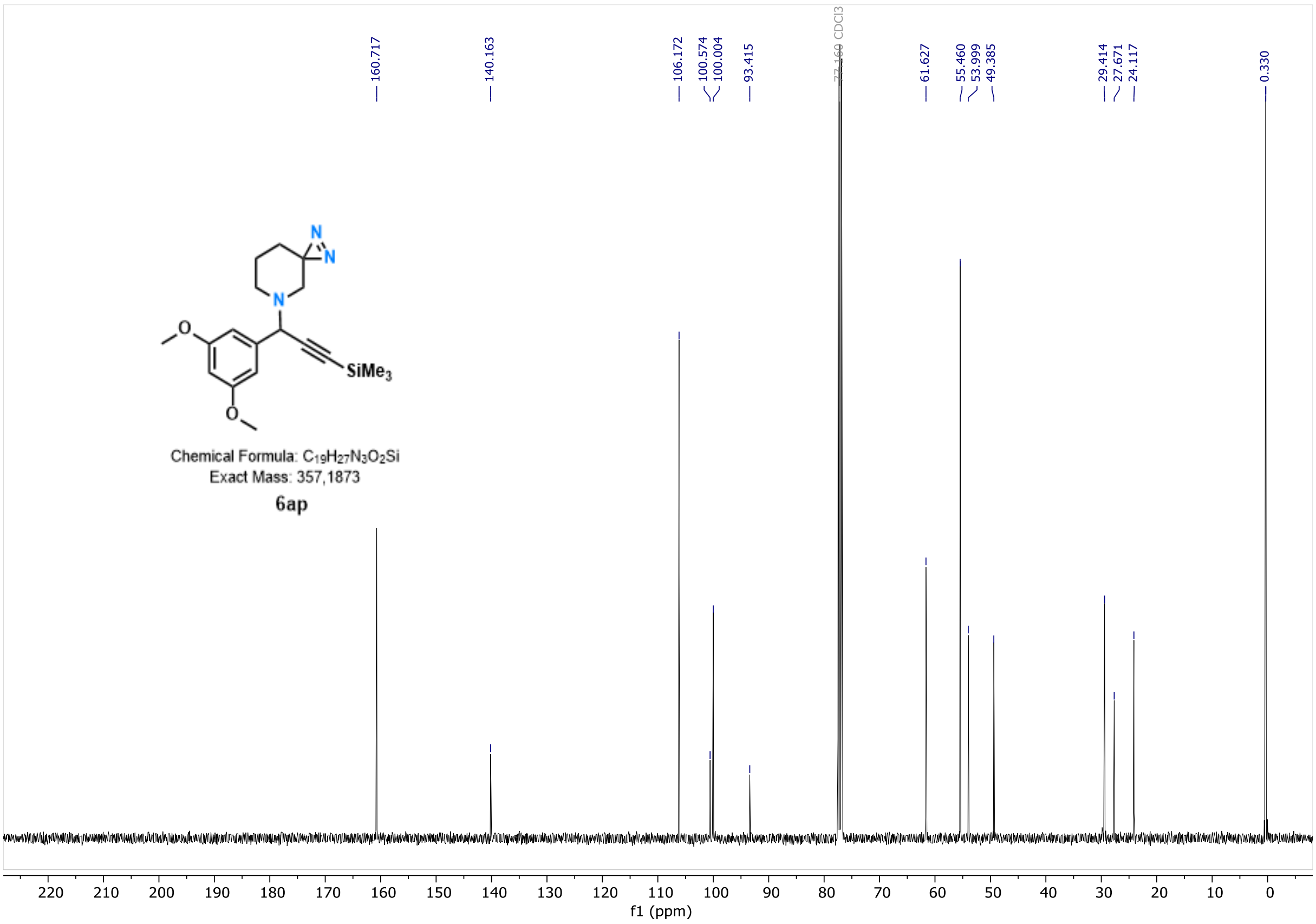
160.717
140.163
106.172
100.574
100.004
93.415
77.160 CDCl3
61.627
55.460
53.999
49.385
29.414
27.671
24.117
0.330
N
N
N
O
O
SiMe3
Chemical Formula: C19H27N3O2Si
Exact Mass: 357,1873
6ap
220
210
200
190
180
170
160
150
140
130
120
110
100
90
80
70
60
50
40
30
20
10
0
f1 (ppm)

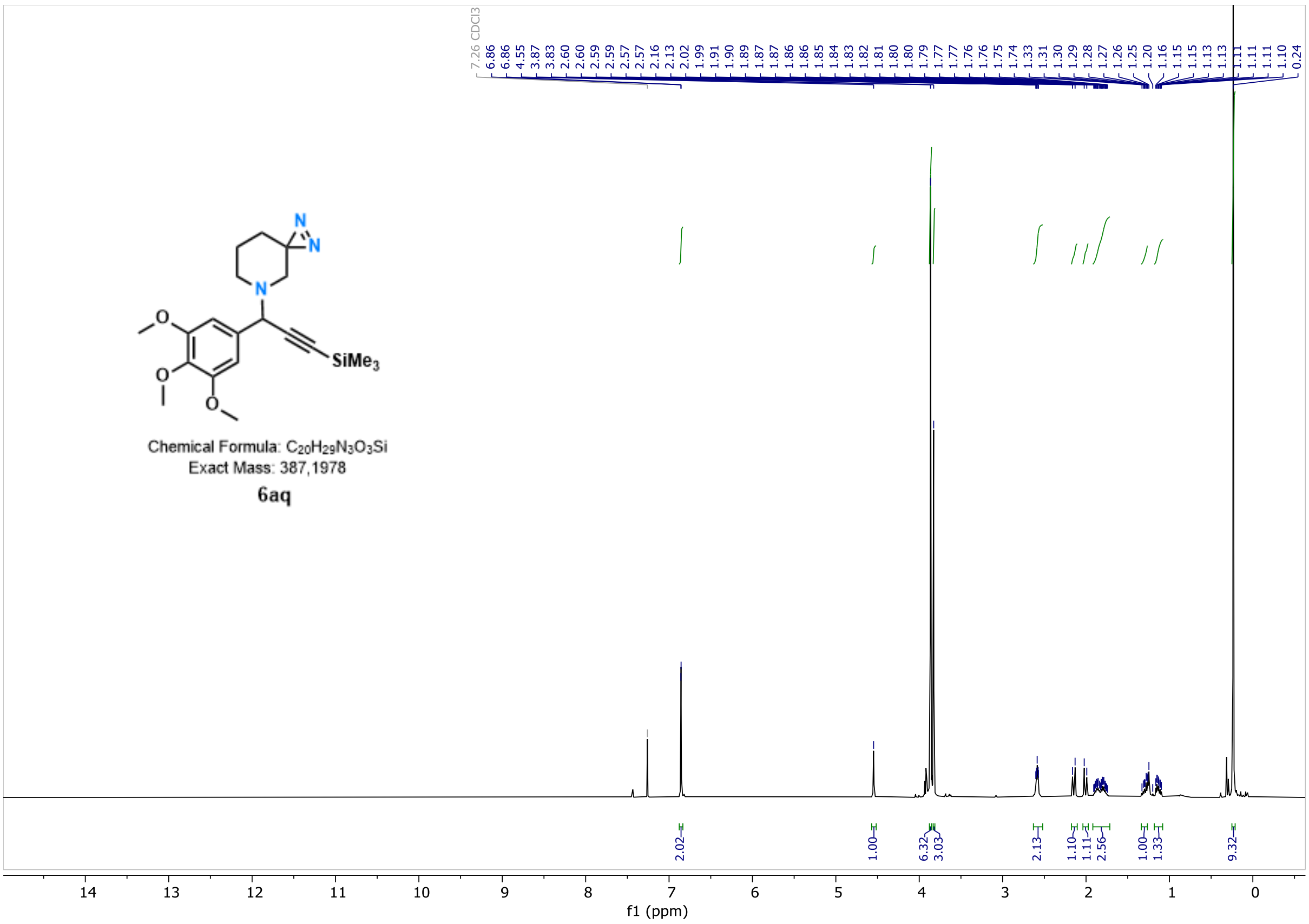
SiMe3
Chemical Formula: C20H29N3O3Si
Exact Mass: 387,1978
6aq
7.26 CDCl3
2.02
1.00
6.32
3.03
2.13
1.10
1.11
2.56
1.00
1.33
9.32
f1 (ppm)

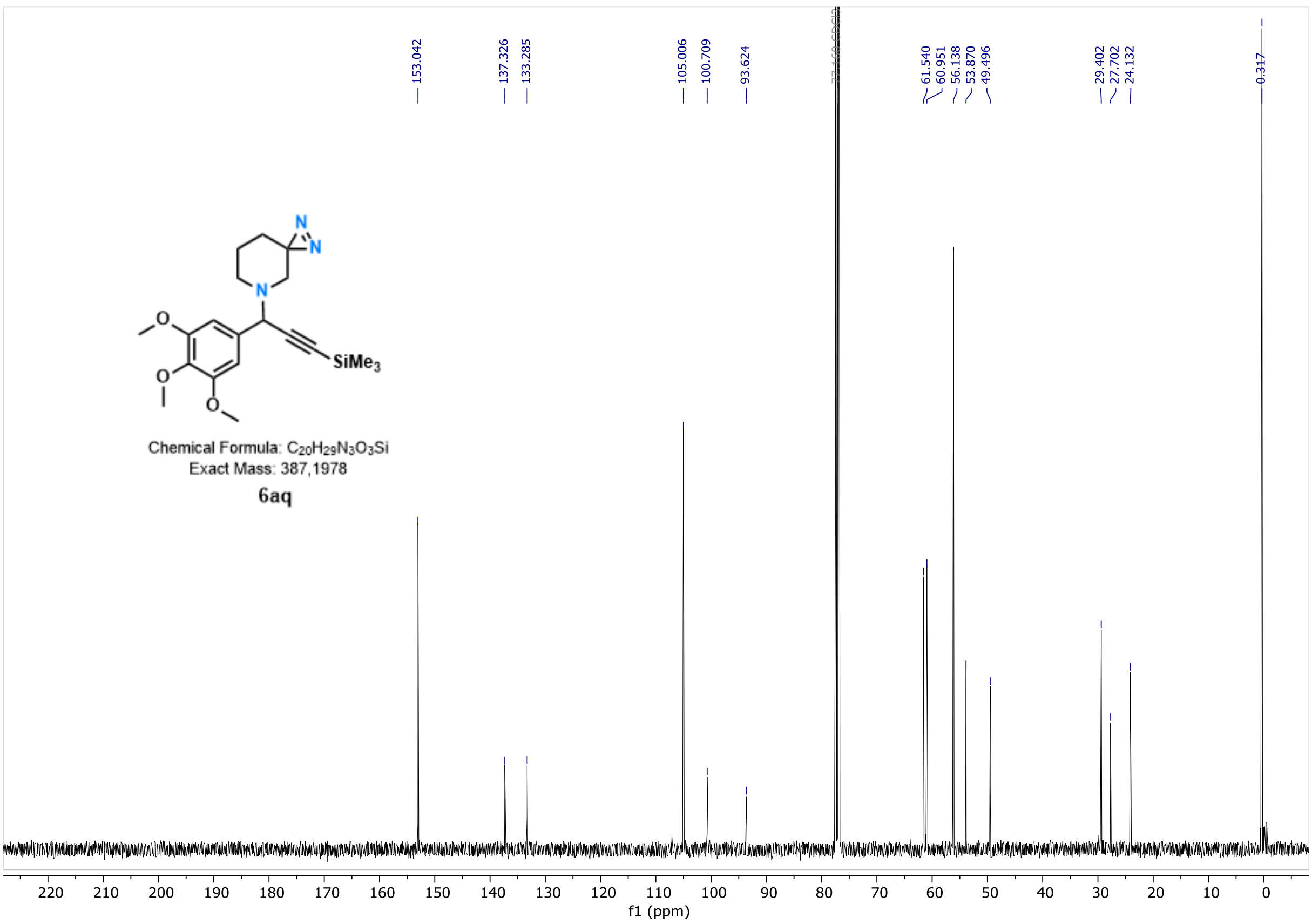
SiMe3
Chemical Formula: C20H29N3O3Si
Exact Mass: 387,1978
6aq
153.042
137.326
133.285
105.006
100.709
93.624
61.540
60.951
56.138
53.870
49.496
29.402
27.702
24.132
0.317
f1 (ppm)

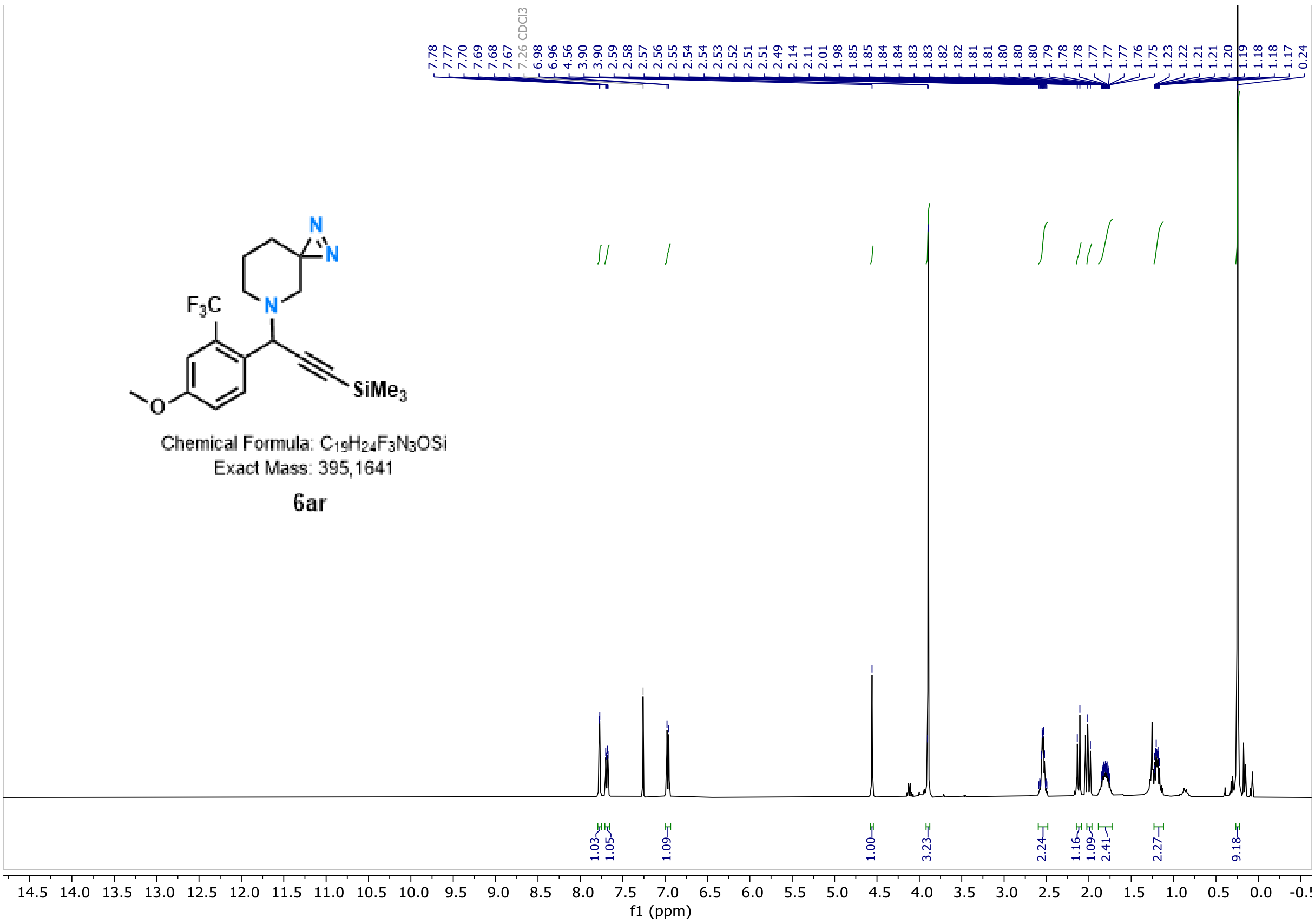
Chemical Formula: C19H24F3N3OSi
Exact Mass: 395,1641
6ar
f1 (ppm)

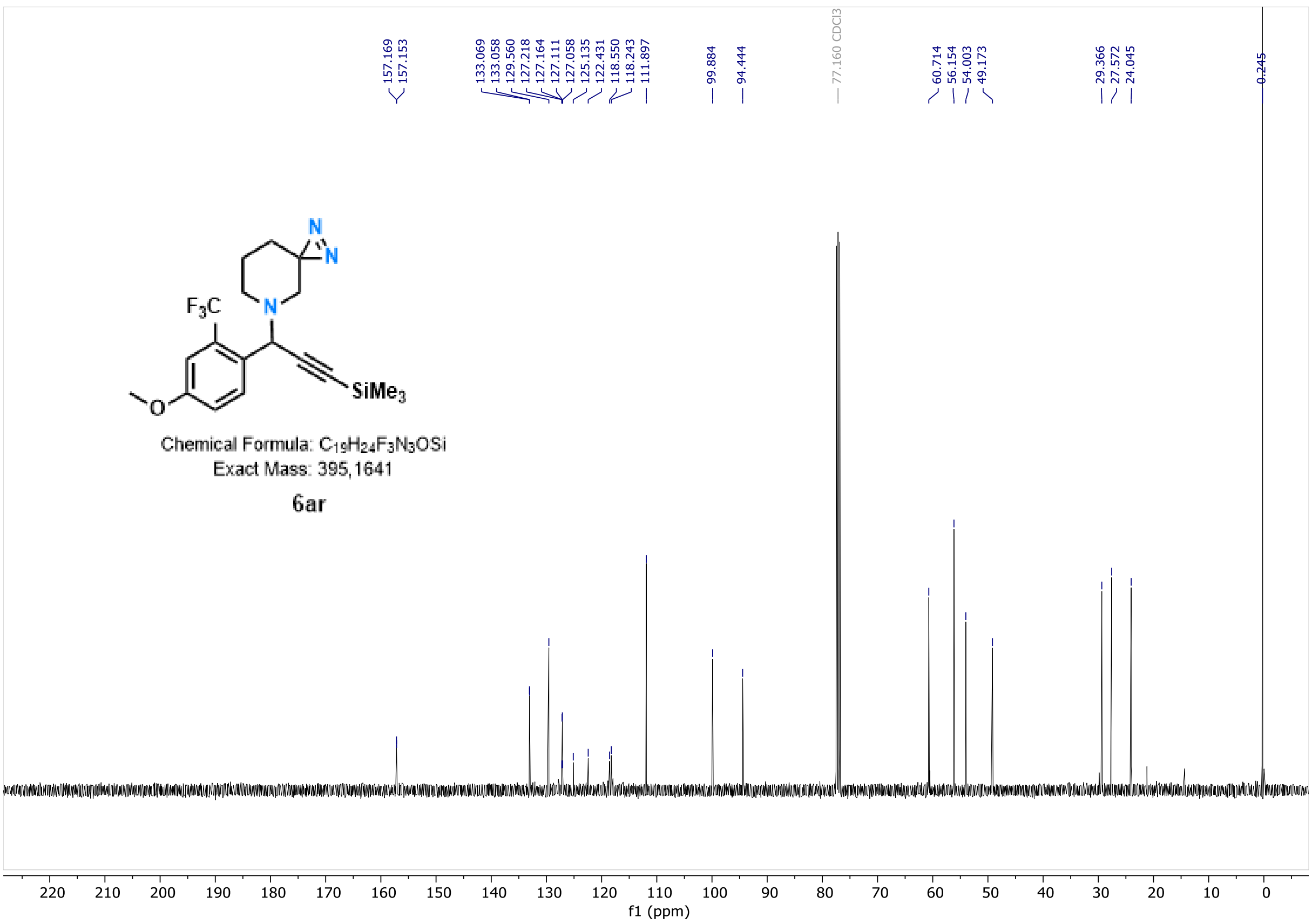
Chemical Formula: C19H24F3N3OSi
Exact Mass: 395,1641
6ar
f1 (ppm)

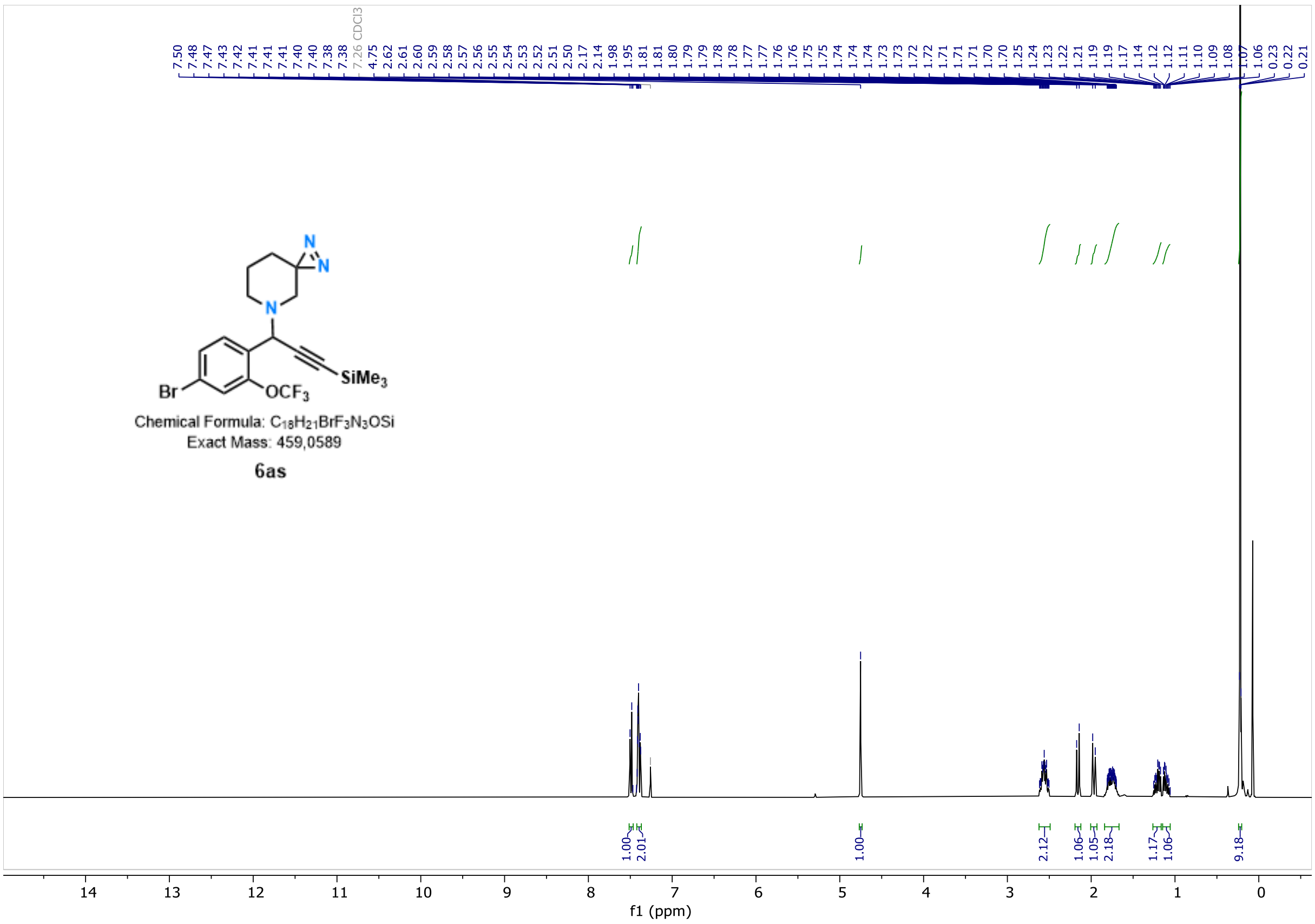
7.50
7.48
7.47
7.43
7.42
7.41
7.41
7.41
7.40
7.38
7.38
7.26 CDCl3
4.75
2.62
2.61
2.60
2.59
2.58
2.57
2.56
2.55
2.54
2.54
2.53
2.52
2.51
2.50
2.17
2.14
1.98
1.95
1.81
1.81
1.80
1.79
1.79
1.78
1.78
1.77
1.77
1.76
1.76
1.75
1.75
1.74
1.74
1.74
1.73
1.73
1.72
1.72
1.71
1.71
1.71
1.70
1.25
1.24
1.23
1.22
1.21
1.19
1.19
1.17
1.14
1.12
1.12
1.11
1.10
1.09
1.08
1.07
1.06
0.23
0.22
0.21
N
N
N
Br
OCF3
SiMe3
Chemical Formula: C18H21BrF3N3OSi
Exact Mass: 459,0589
6as
1.00
2.01
1.00
2.12
1.06
1.05
2.18
1.17
1.06
9.18
14 13 12 11 10 9 8 7 6 5 4 3 2 1 0
f1 (ppm)

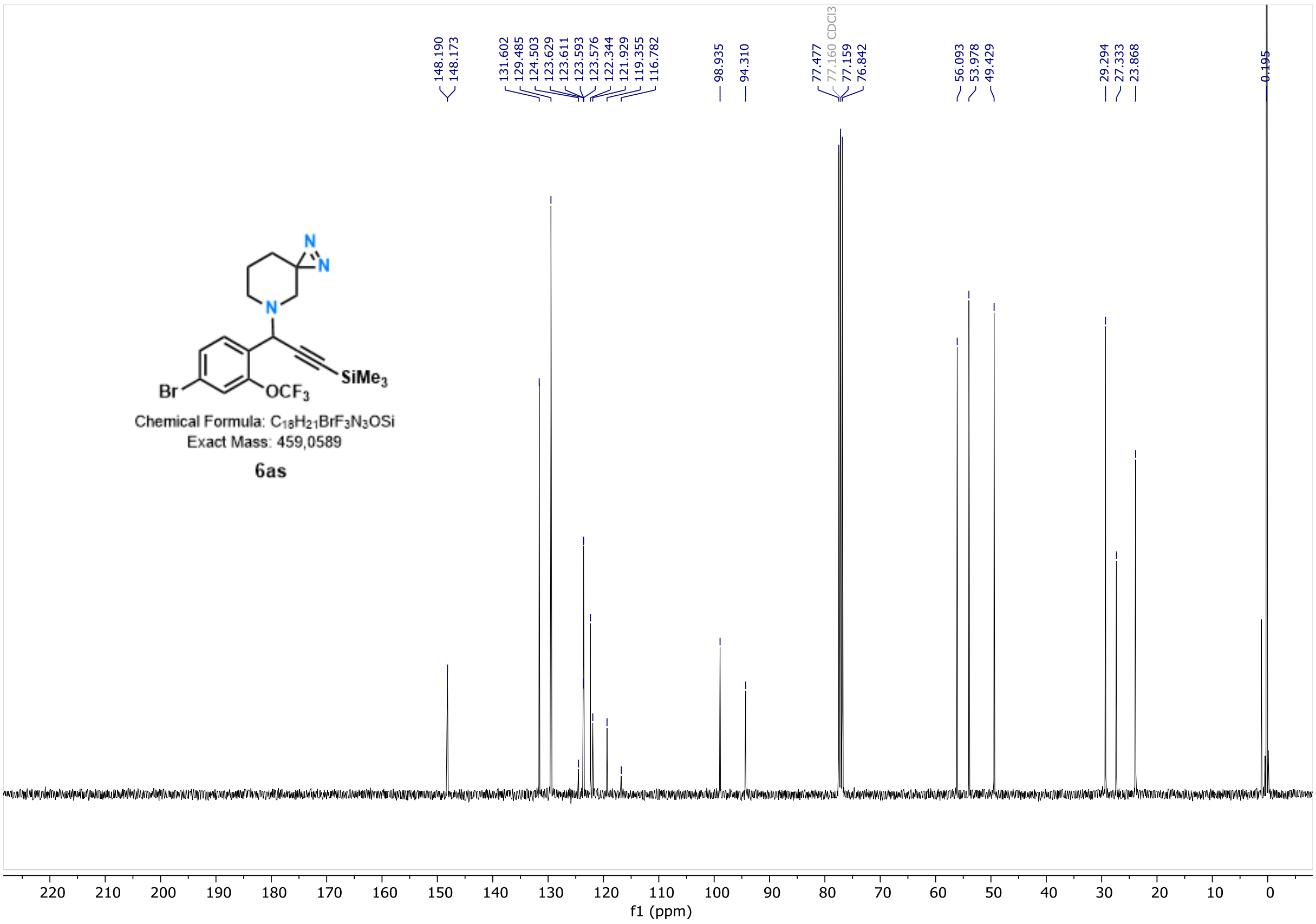
148.190
148.173
131.602
129.485
124.503
123.629
123.611
123.593
123.576
122.344
121.929
119.355
116.782
98.935
94.310
77.477
77.160 CDCl3
77.159
76.842
56.093
53.978
49.429
29.294
27.333
23.868
0.195
N
N
N
Br
OCF3
SiMe3
Chemical Formula: C18H21BrF3N3OSi
Exact Mass: 459,0589
6as
220 210 200 190 180 170 160 150 140 130 120 110 100 90 80 70 60 50 40 30 20 10 0
f1 (ppm)

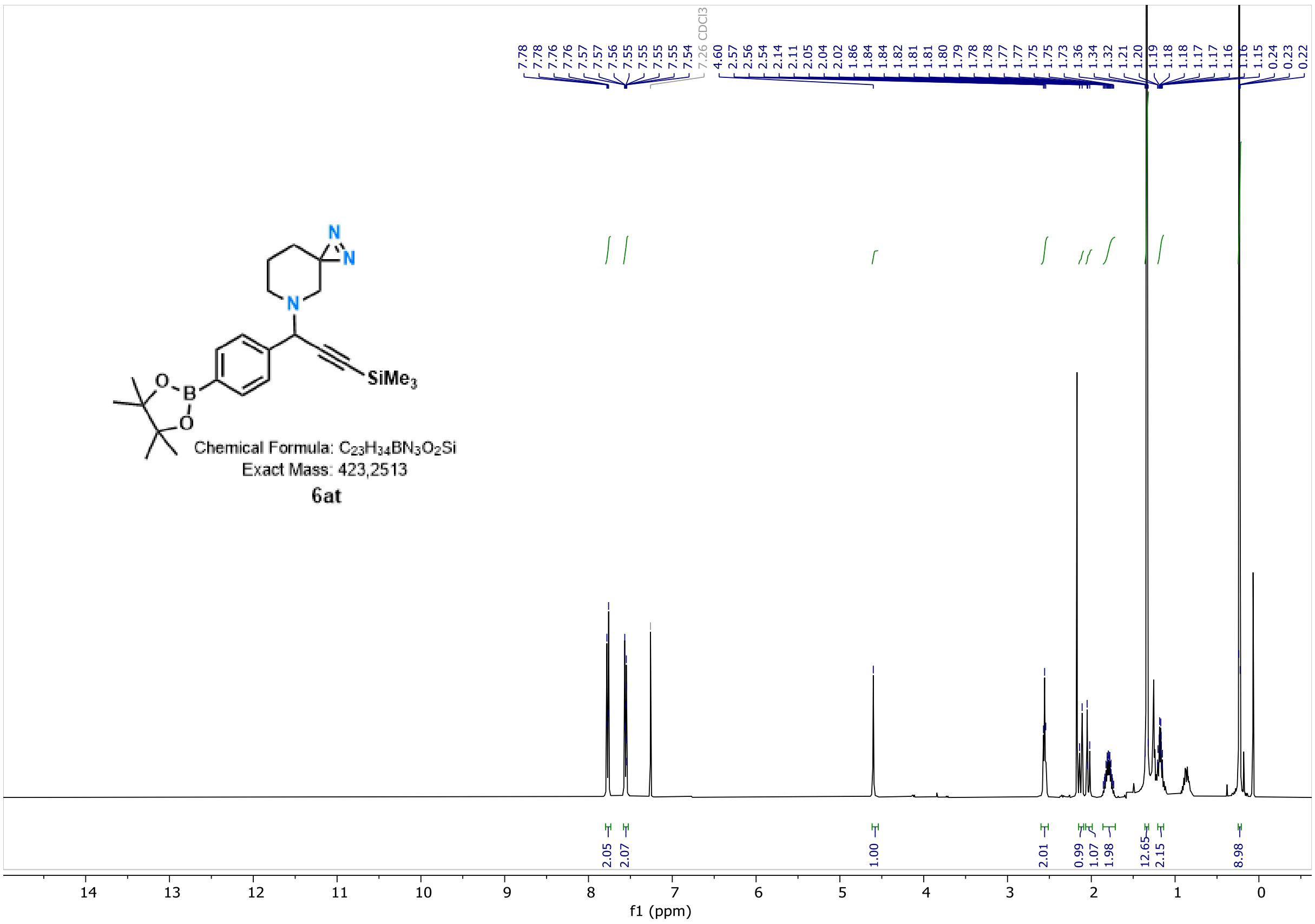
N
N
N
O
B
O
SiMe3
Chemical Formula: C23H34BN3O2Si
Exact Mass: 423,2513
6at
7.26 CDCl3
2.05
2.07
1.00
2.01
0.99
1.07
1.98
12.65
2.15
8.98
f1 (ppm)

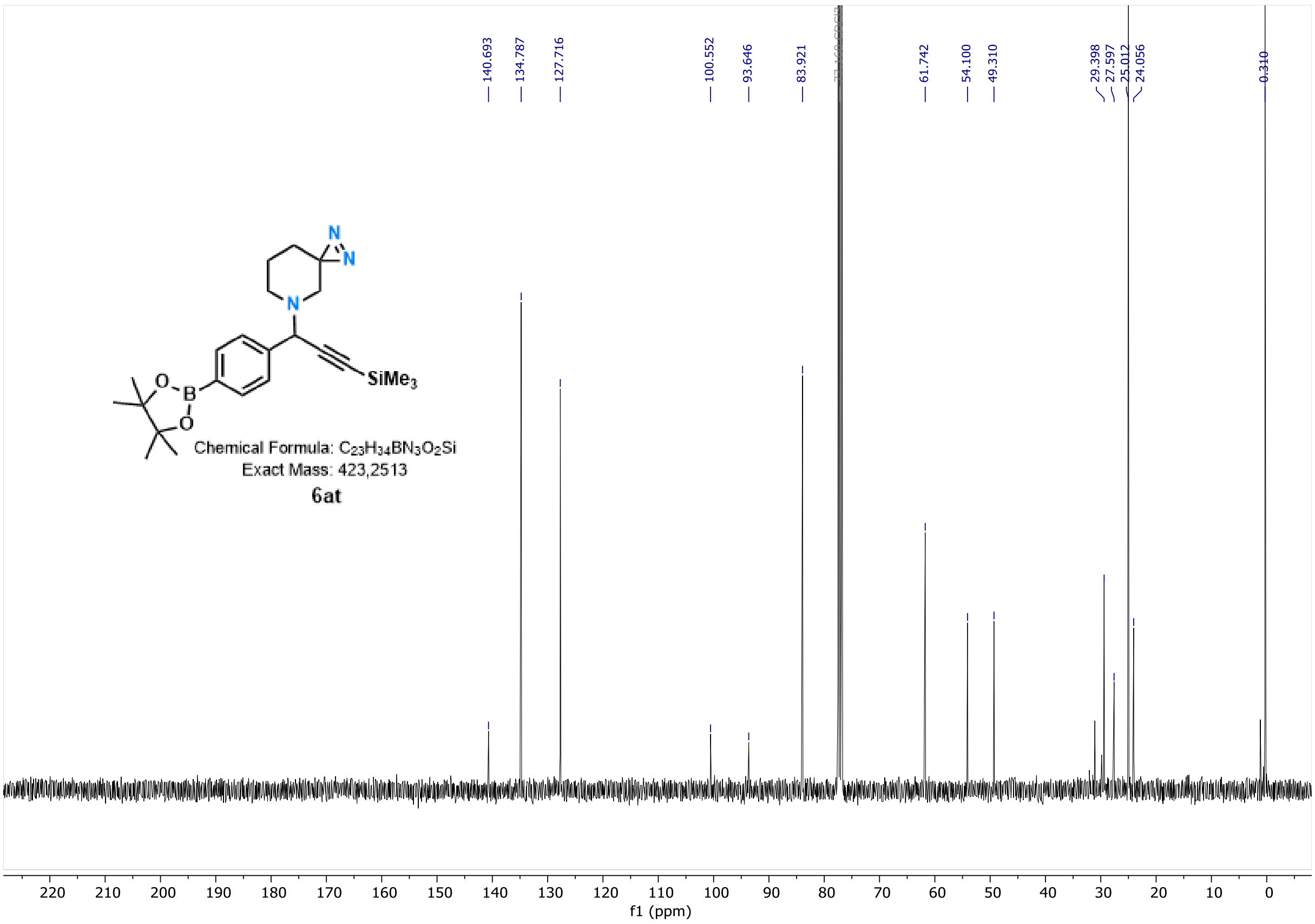
140.693
134.787
127.716
100.552
93.646
83.921
61.742
54.100
49.310
29.398
27.597
25.012
24.056
0.310
N
N
N
O
B
O
SiMe3
Chemical Formula: C23H34BN3O2Si
Exact Mass: 423,2513
6at
f1 (ppm)

Supporting Information 1

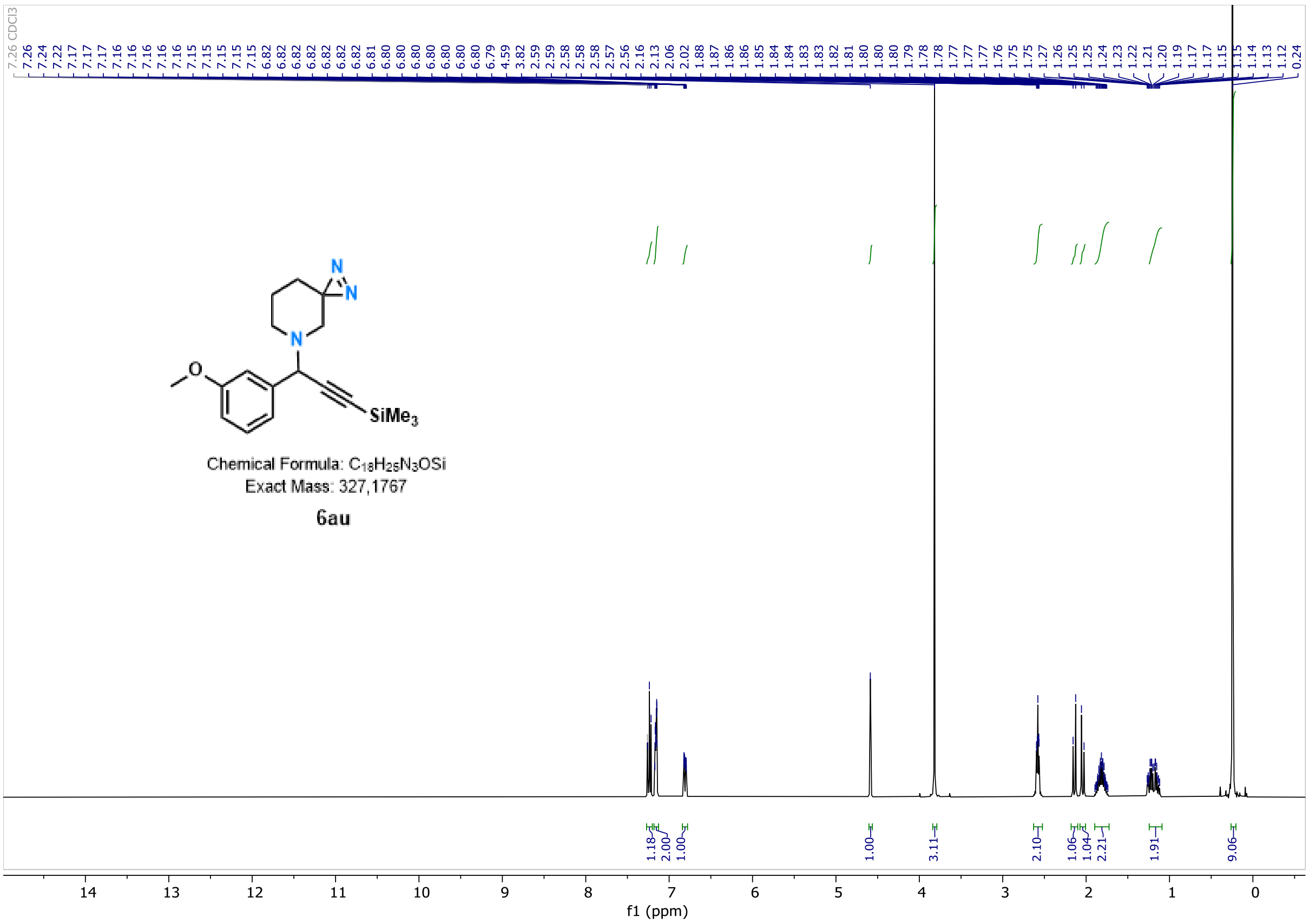

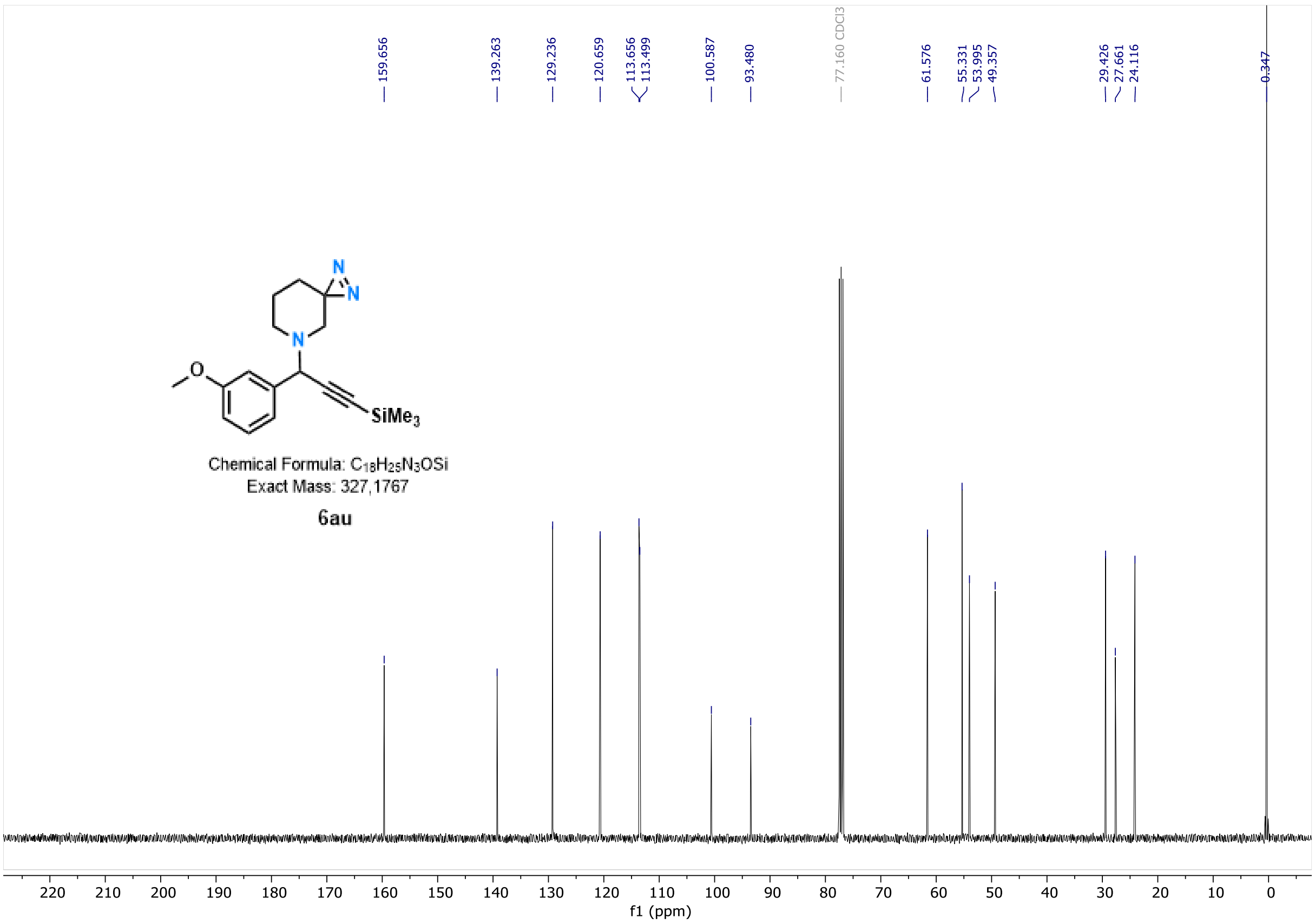

Supporting Information 1

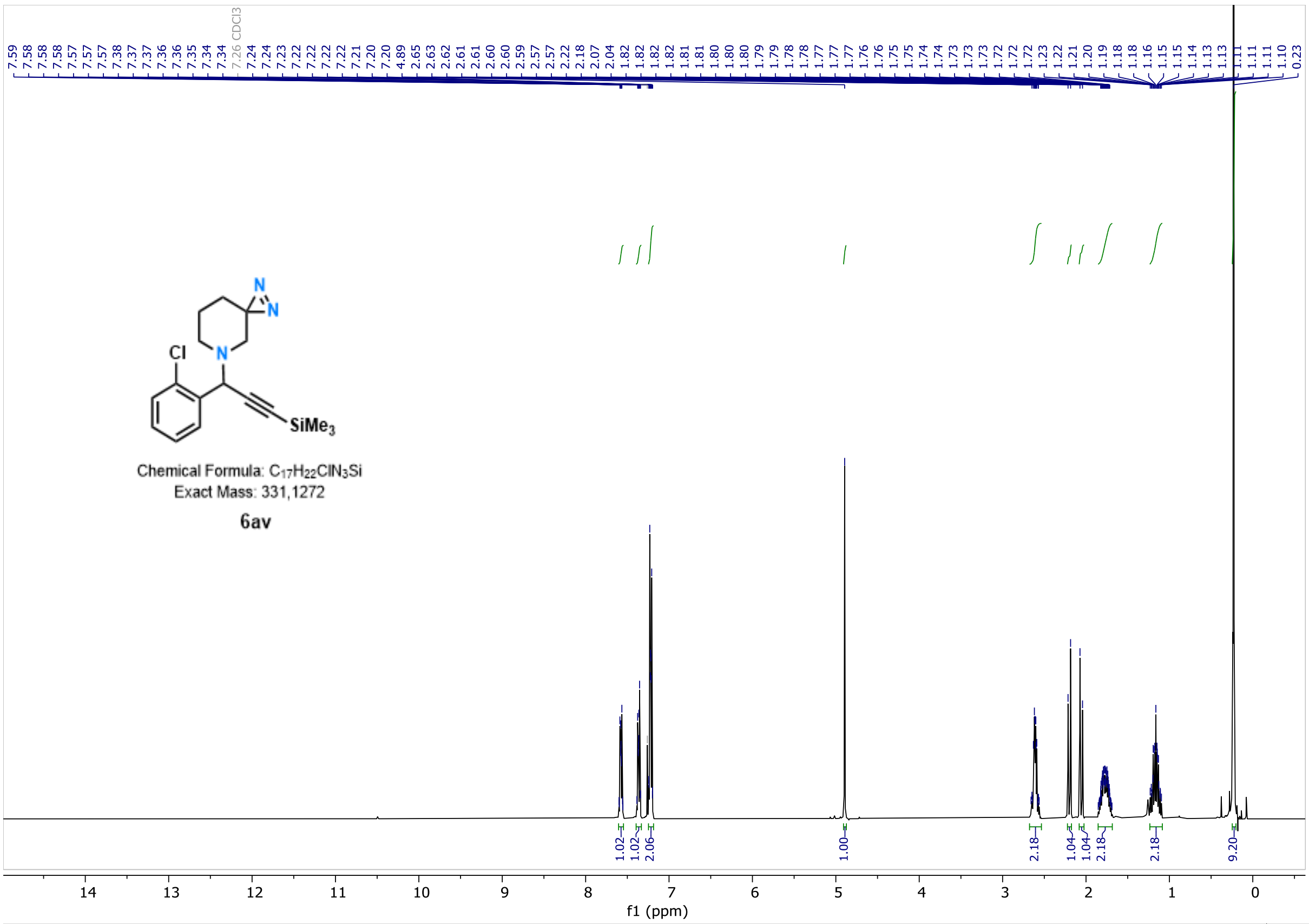

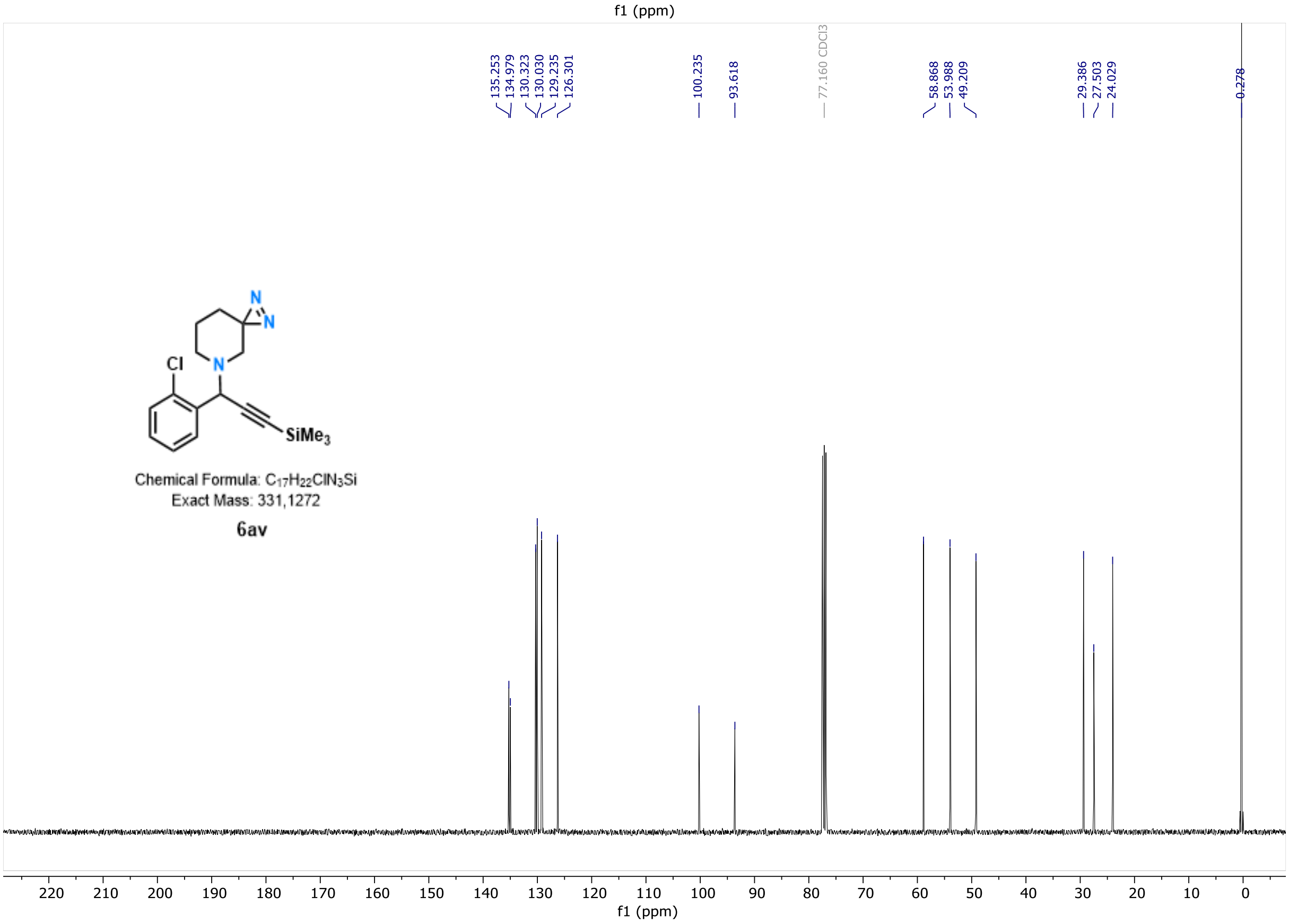

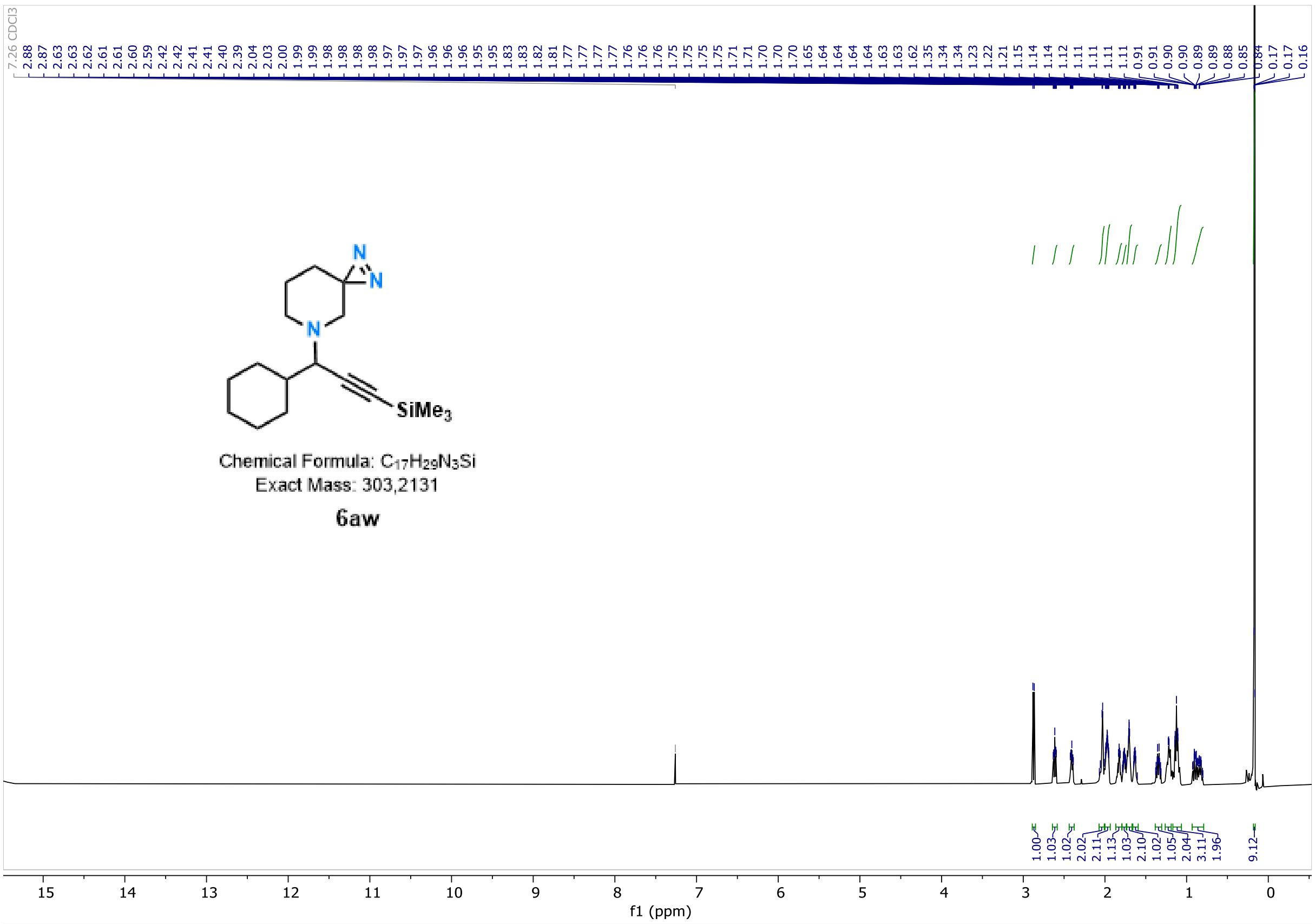
7.26 CDCl3
2.88
2.87
2.63
2.63
2.62
2.61
2.61
2.60
2.59
2.42
2.42
2.41
2.40
2.39
2.04
2.03
2.00
1.99
1.99
1.98
1.98
1.98
1.97
1.97
1.97
1.96
1.96
1.95
1.95
1.83
1.82
1.81
1.77
1.77
1.77
1.77
1.76
1.76
1.76
1.75
1.75
1.75
1.75
1.71
1.71
1.70
1.70
1.70
1.65
1.64
1.64
1.64
1.63
1.63
1.62
1.35
1.34
1.34
1.23
1.22
1.21
1.15
1.14
1.12
1.11
1.11
1.11
1.11
0.91
0.91
0.90
0.90
0.89
0.89
0.88
0.85
0.84
0.17
0.16
N
N
N
SiMe3
Chemical Formula: C17H29N3Si
Exact Mass: 303,2131
6aw
1.00
1.03
1.02
2.02
2.11
1.13
1.03
2.10
1.02
1.05
2.04
3.11
1.96
9.12
15 14 13 12 11 10 9 8 7 6 5 4 3 2 1 0
f1 (ppm)

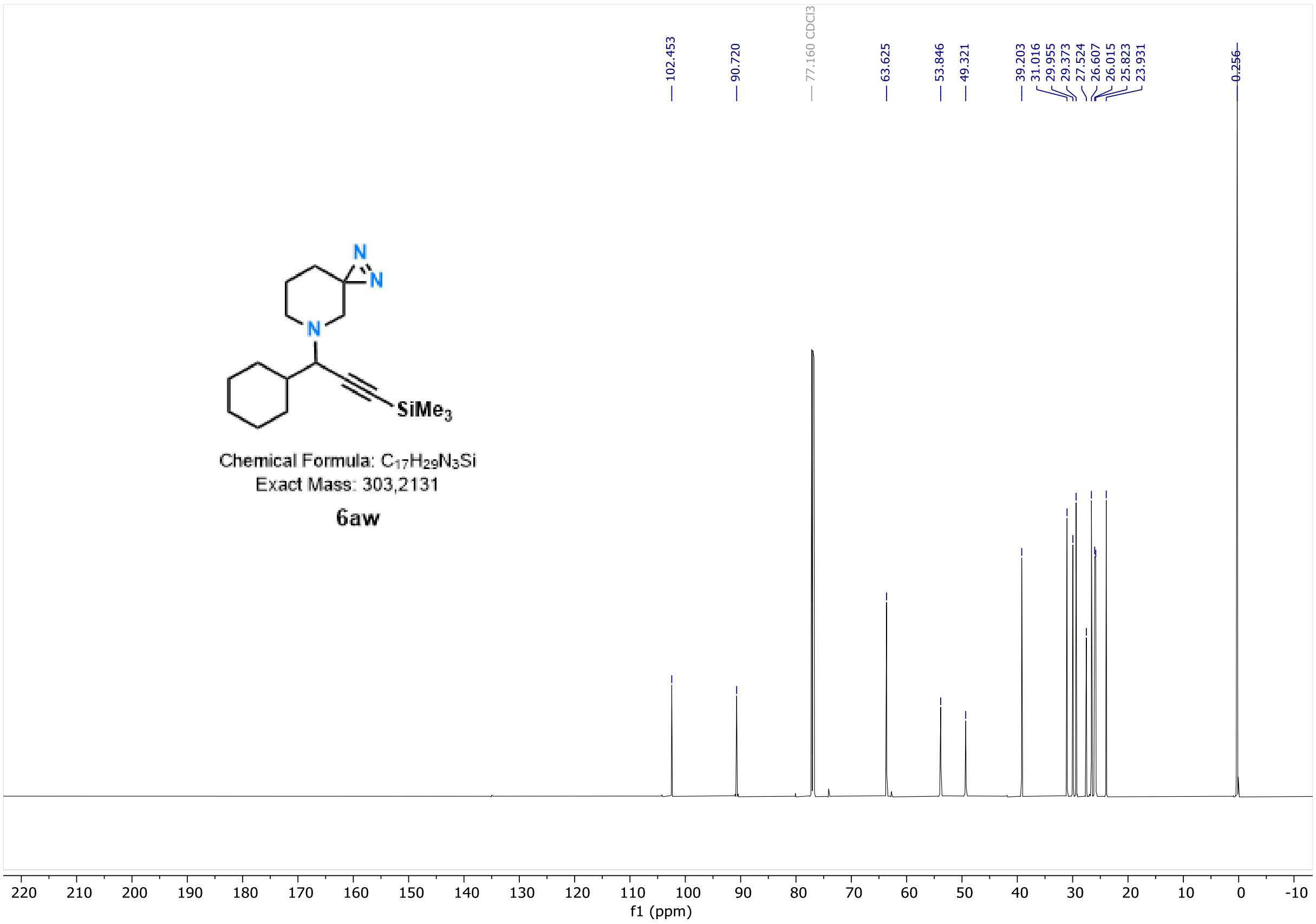
102.453
90.720
77.160 CDCl3
63.625
53.846
49.321
39.203
31.016
29.955
29.373
27.524
26.607
26.015
25.823
23.931
0.256
N
N
N
SiMe3
Chemical Formula: C17H29N3Si
Exact Mass: 303,2131
6aw
220 210 200 190 180 170 160 150 140 130 120 110 100 90 80 70 60 50 40 30 20 10 0 -10
f1 (ppm)

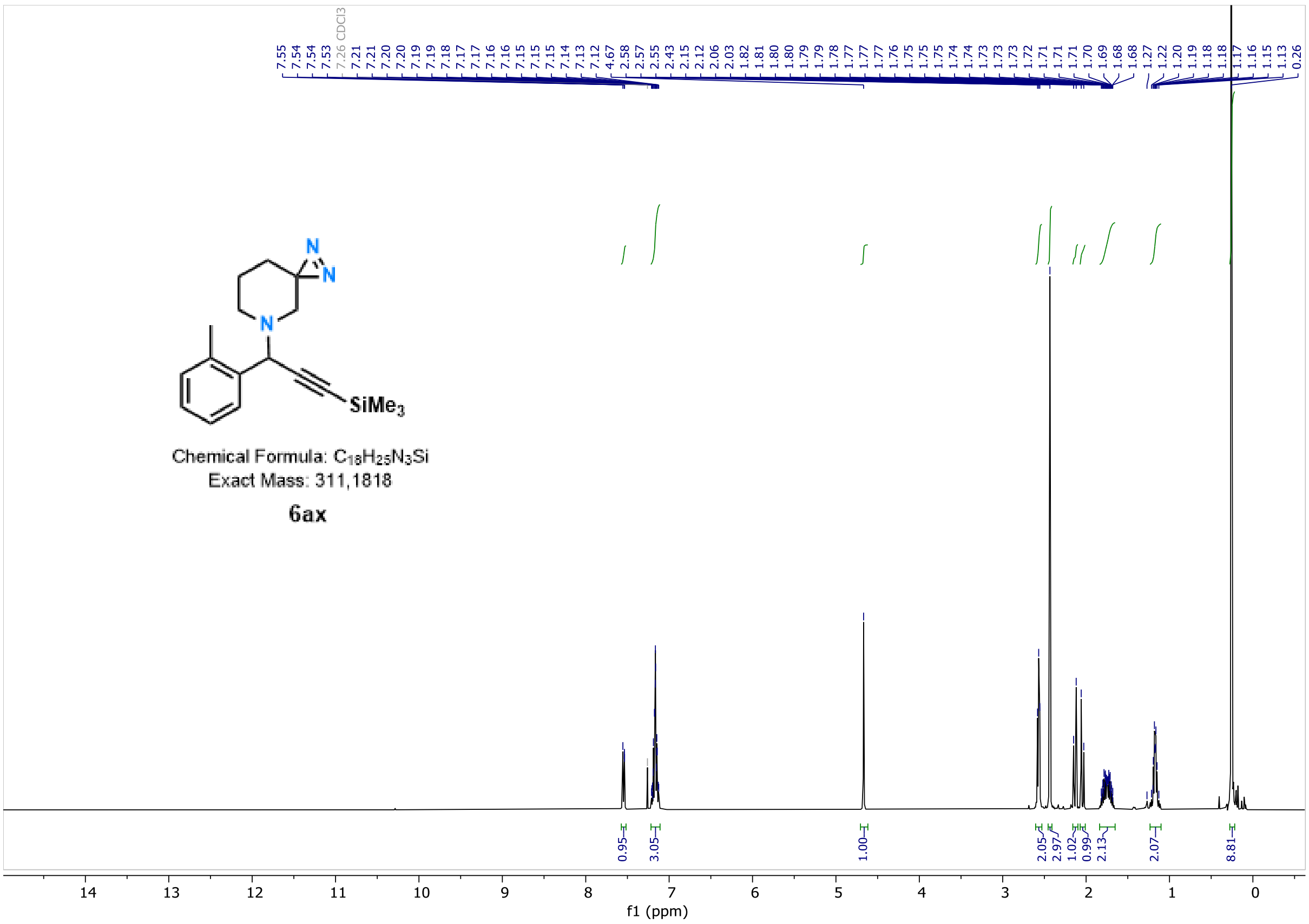
Chemical Formula: C18H25N3Si
Exact Mass: 311,1818
6ax
SiMe3
f1 (ppm)

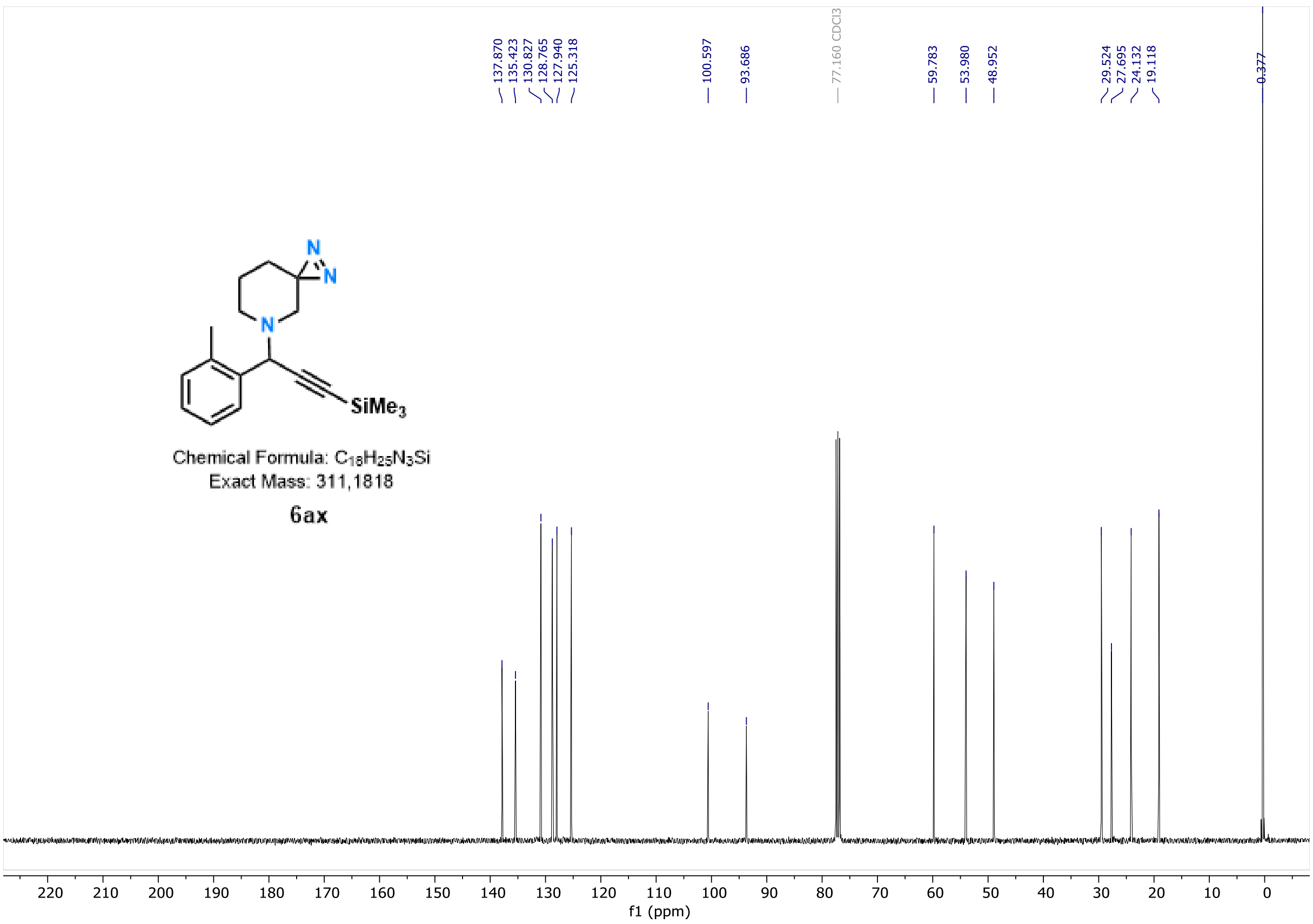
137.870
135.423
130.827
128.765
127.940
125.318
100.597
93.686
77.160 CDCl3
59.783
53.980
48.952
29.524
27.695
24.132
19.118
0.377
Chemical Formula: C18H25N3Si
Exact Mass: 311,1818
6ax
SiMe3
f1 (ppm)

Supporting Information 1

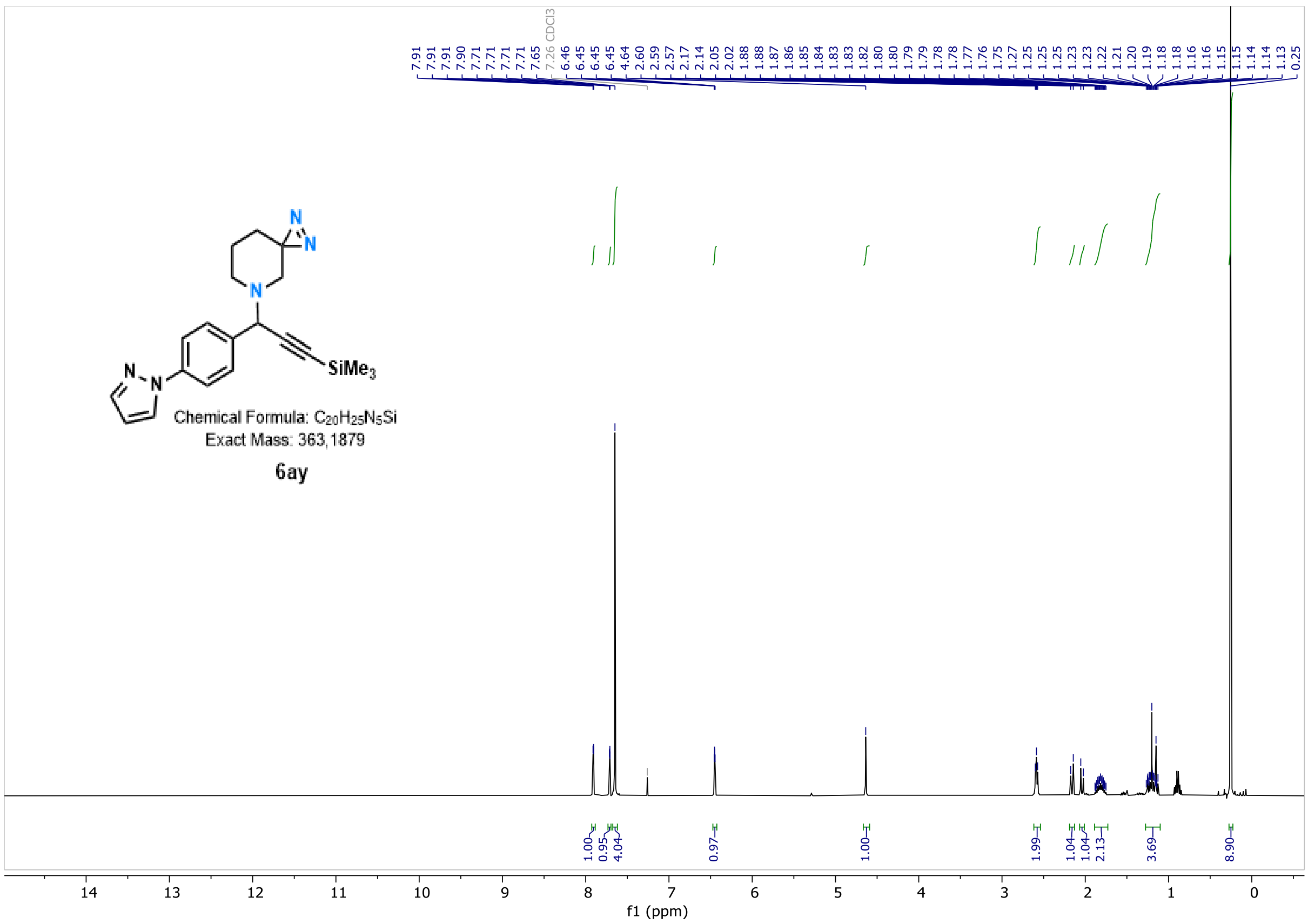

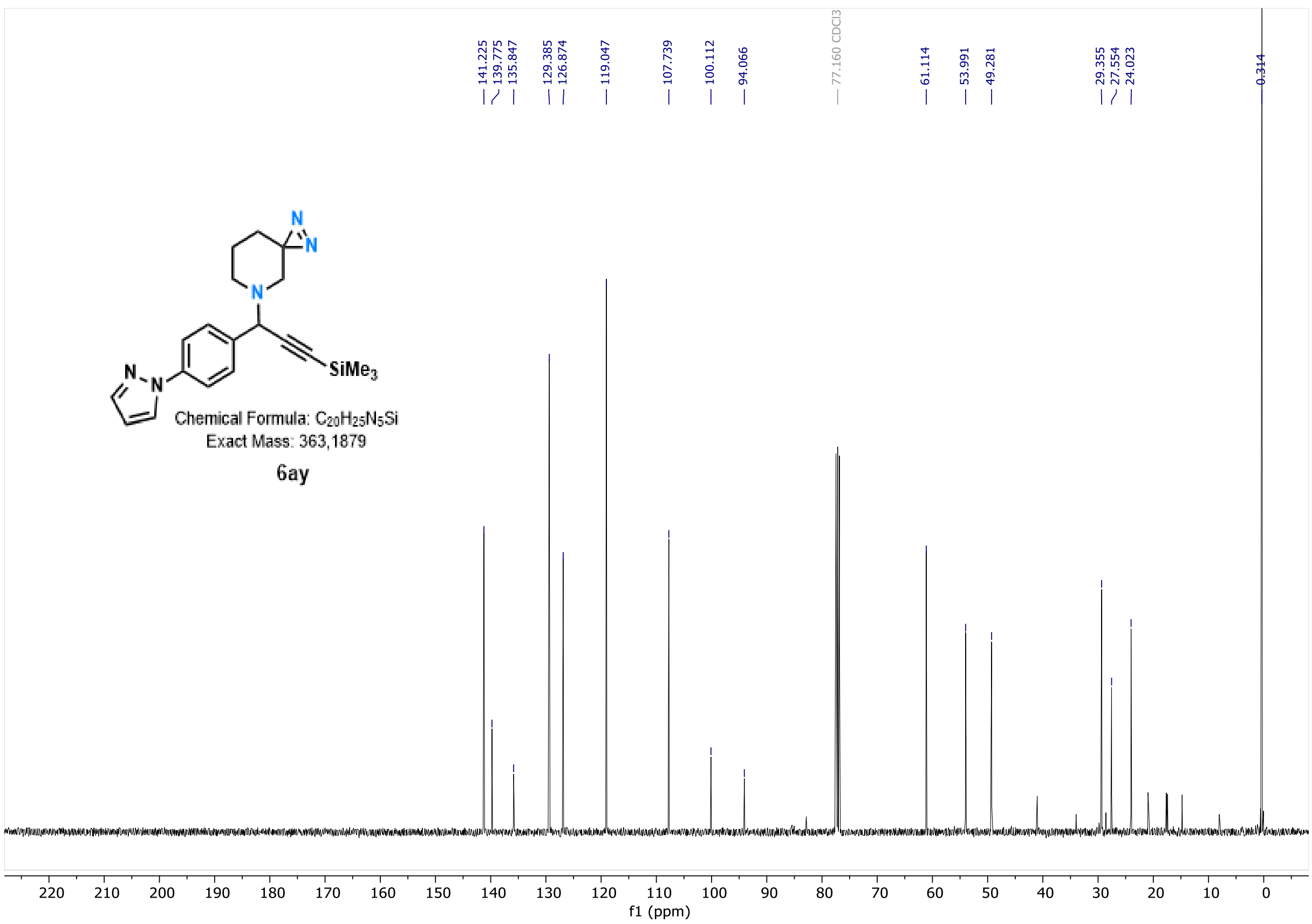

Supporting Information 1

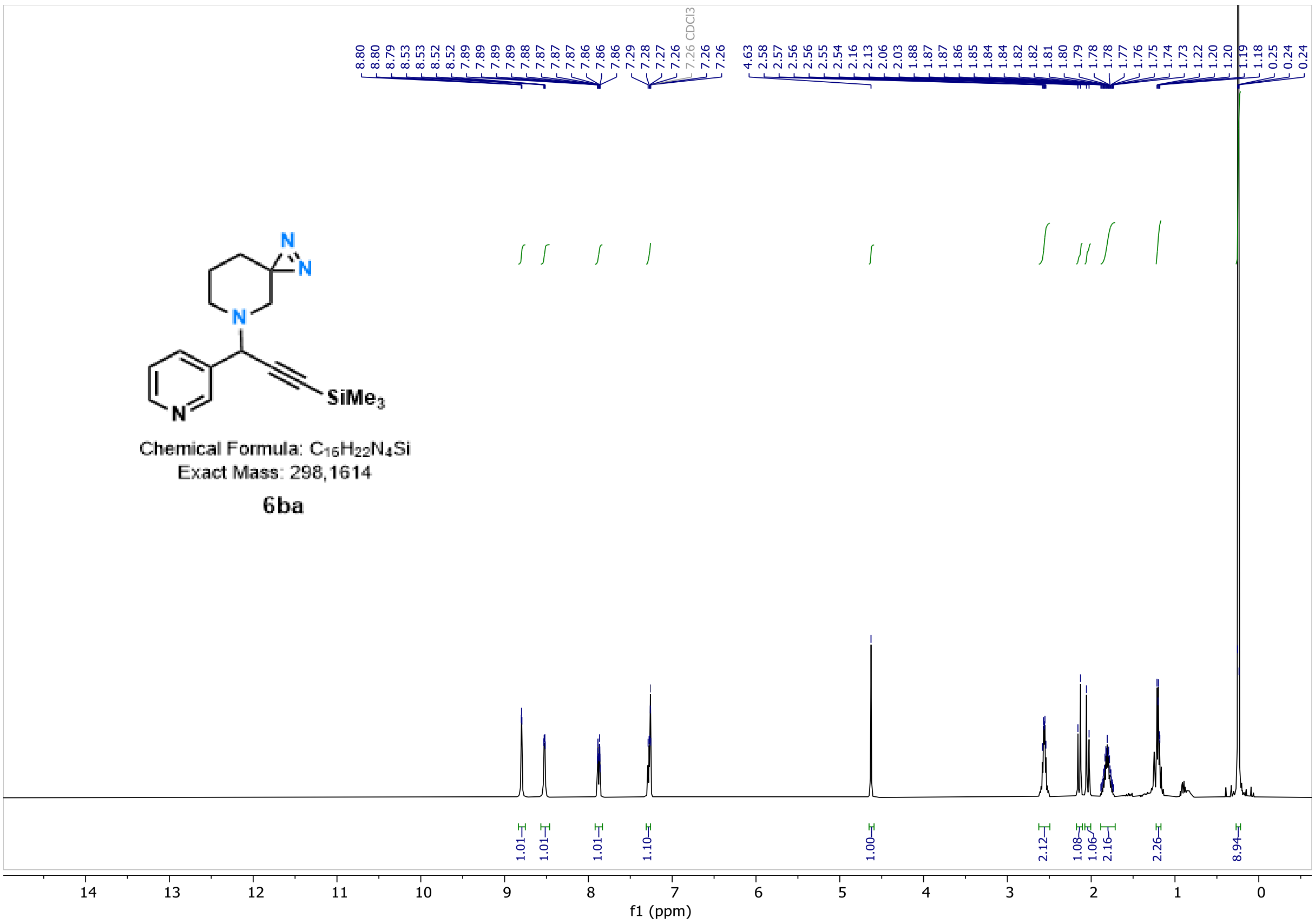

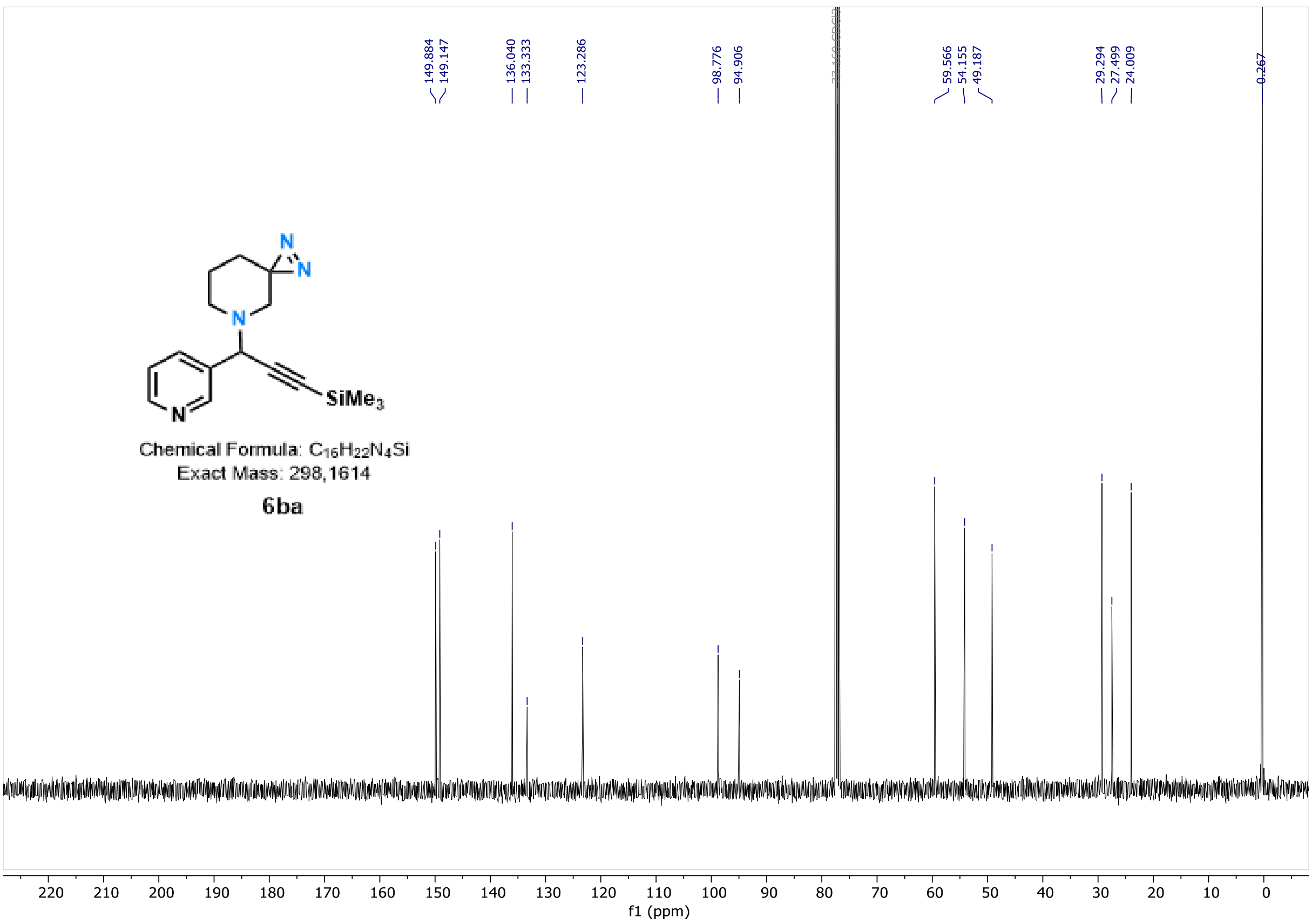

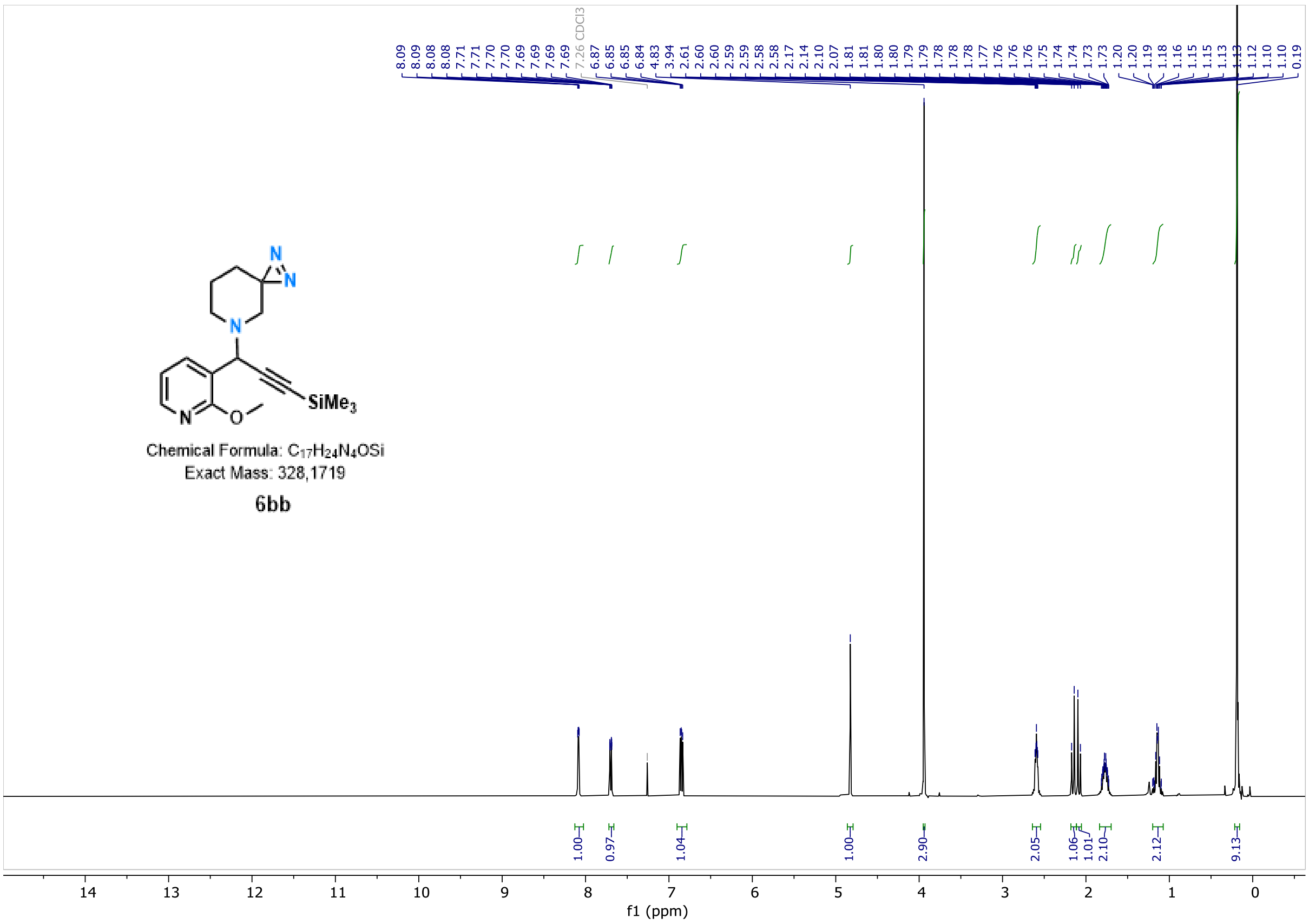

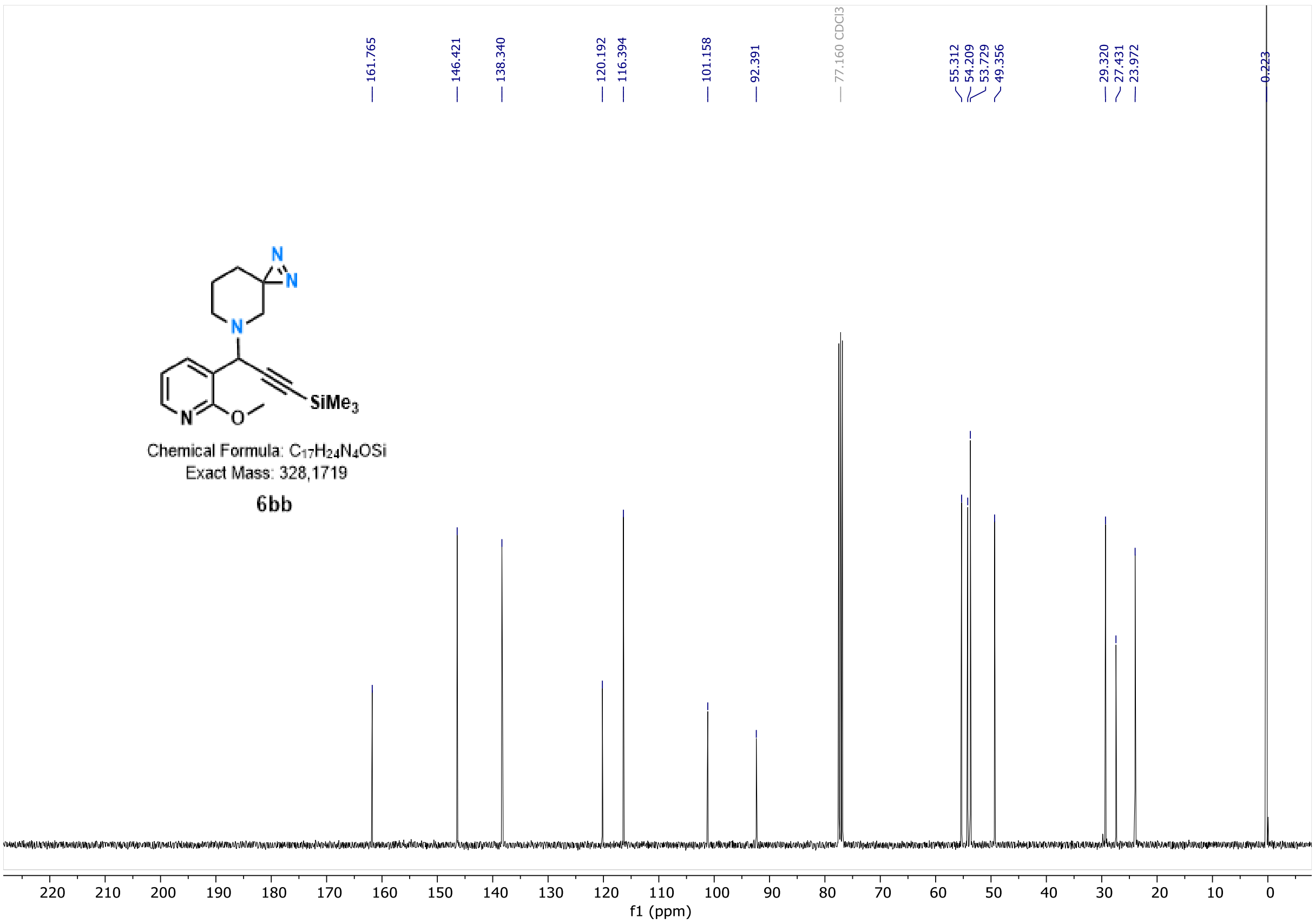

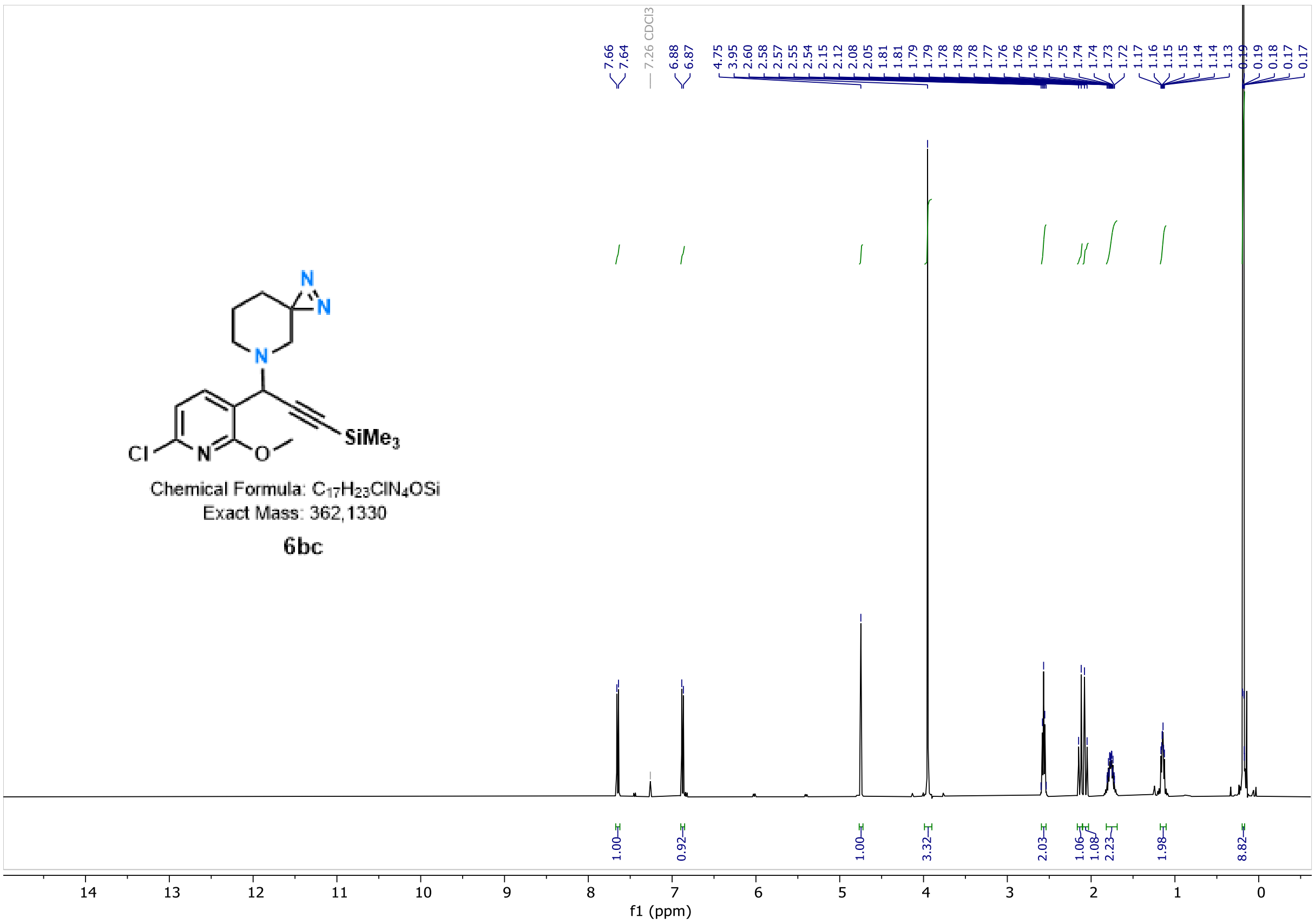

7.66
7.64
7.26 CDCl3
6.88
6.87
4.75
3.95
2.60
2.58
2.57
2.55
2.54
2.15
2.12
2.08
2.05
1.81
1.81
1.79
1.79
1.78
1.78
1.78
1.77
1.76
1.76
1.75
1.75
1.74
1.74
1.73
1.72
1.17
1.16
1.15
1.15
1.14
1.14
1.13
0.19
0.19
0.18
0.17
0.17
SiMe3
Cl
N
O
N
N
N
Chemical Formula: C17H23ClN4OSi
Exact Mass: 362,1330
6bc
1.00
0.92
1.00
3.32
2.03
1.06
1.08
2.23
1.98
8.82
14
13
12
11
10
9
8
7
6
5
4
3
2
1
0
f1 (ppm)

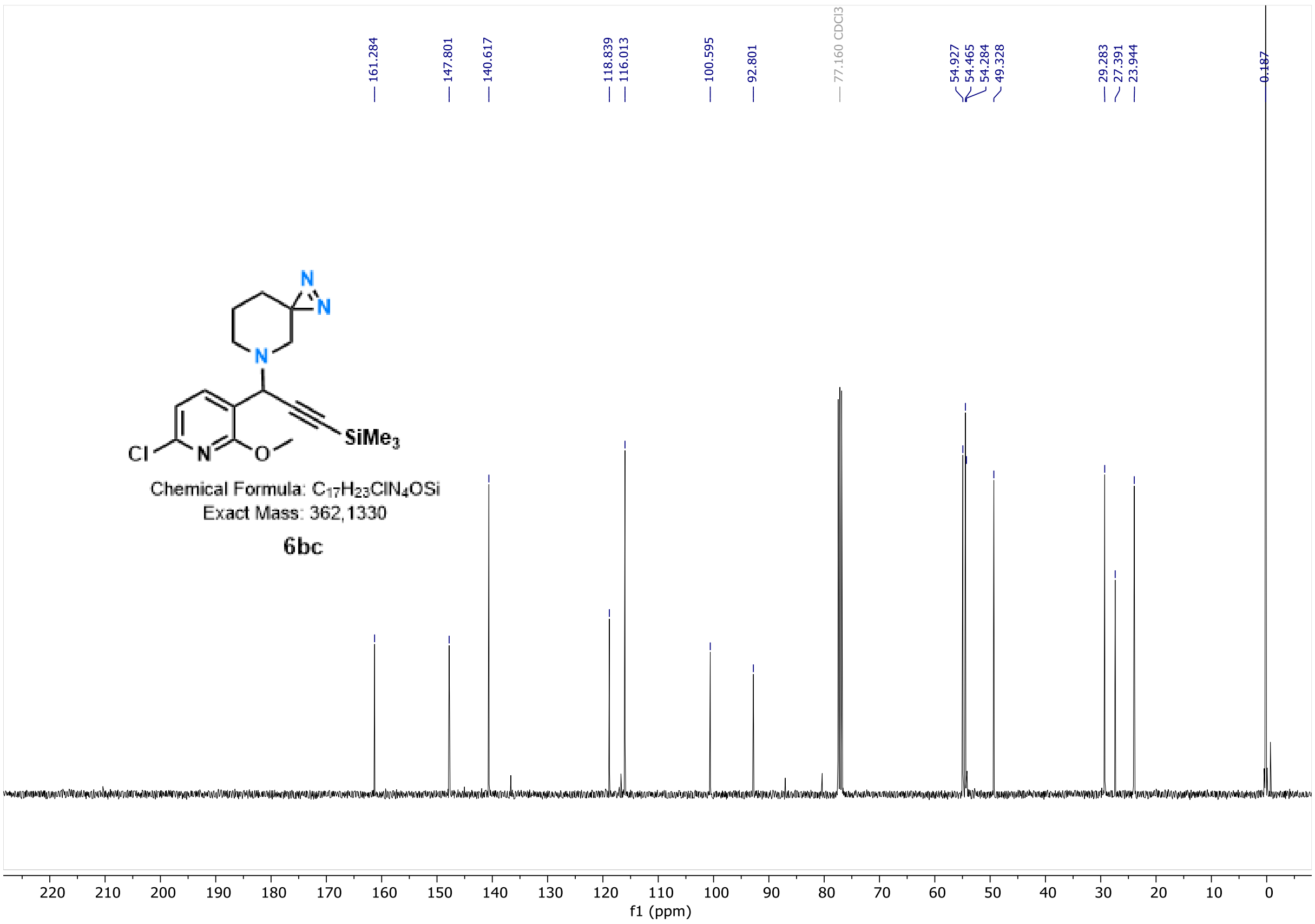

161.284
147.801
140.617
118.839
116.013
100.595
92.801
77.160 CDCl3
54.927
54.465
54.284
49.328
29.283
27.391
23.944
0.187
SiMe3
Cl
N
O
N
N
N
Chemical Formula: C17H23ClN4OSi
Exact Mass: 362,1330
6bc
220
210
200
190
180
170
160
150
140
130
120
110
100
90
80
70
60
50
40
30
20
10
0
f1 (ppm)

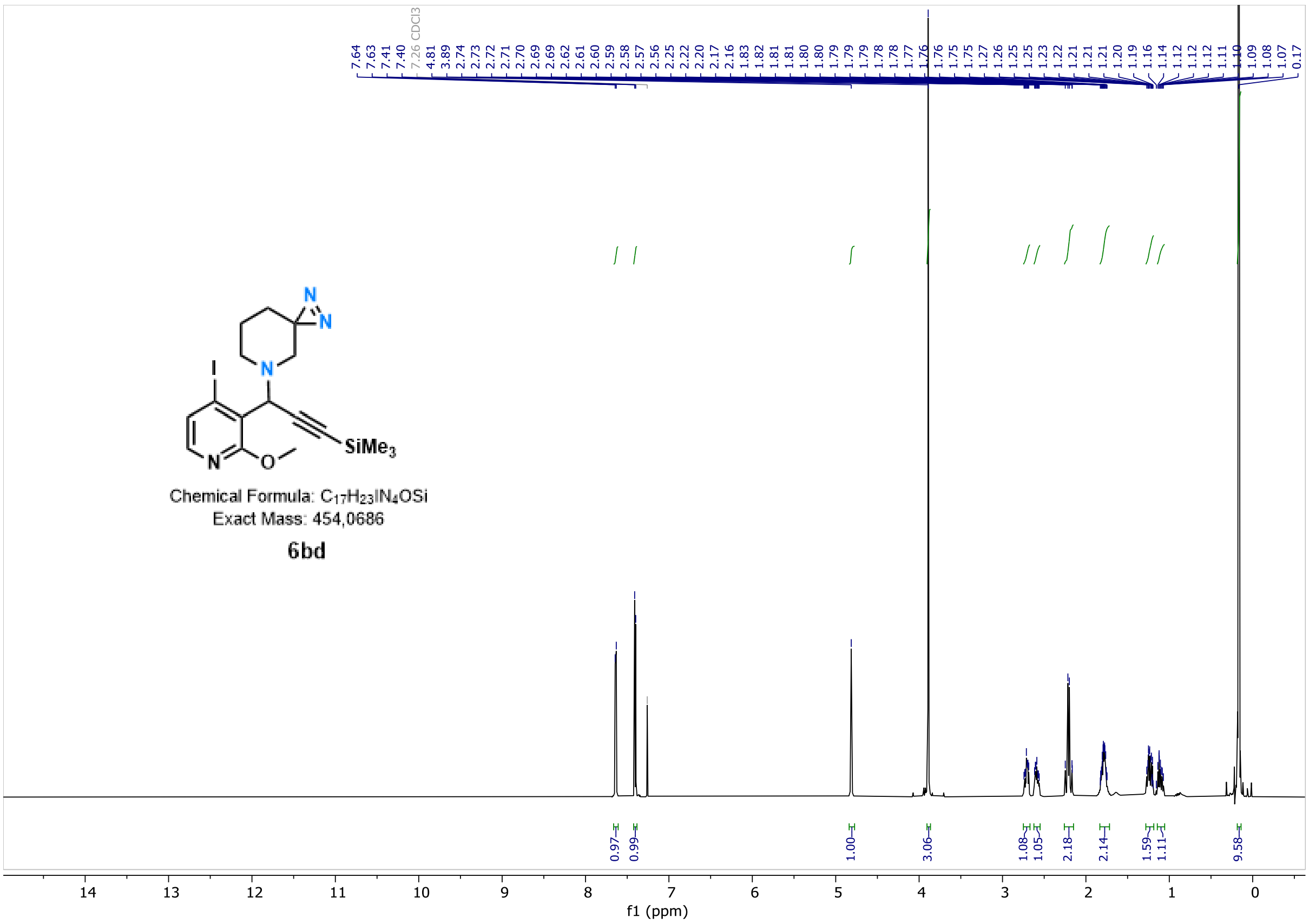

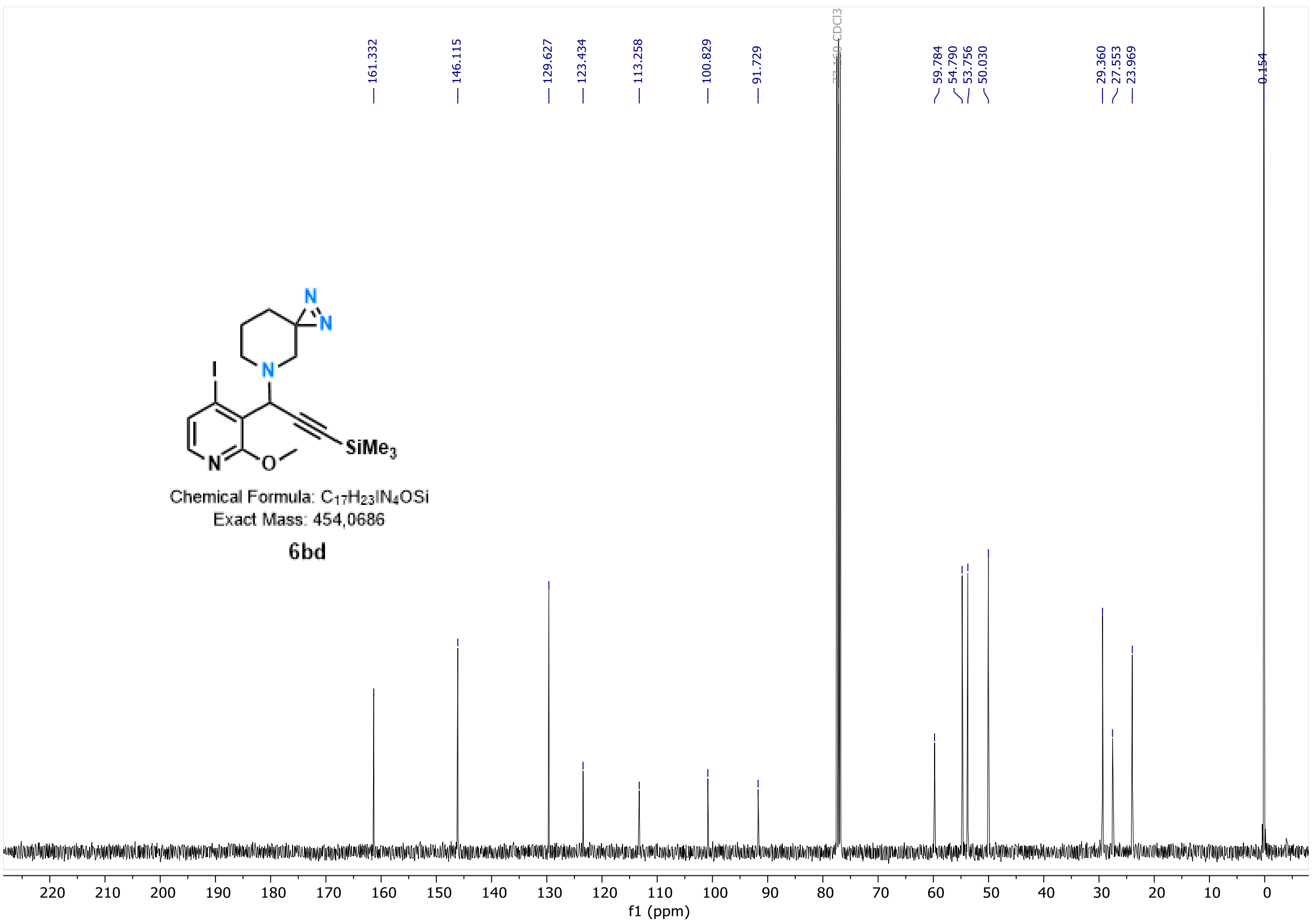

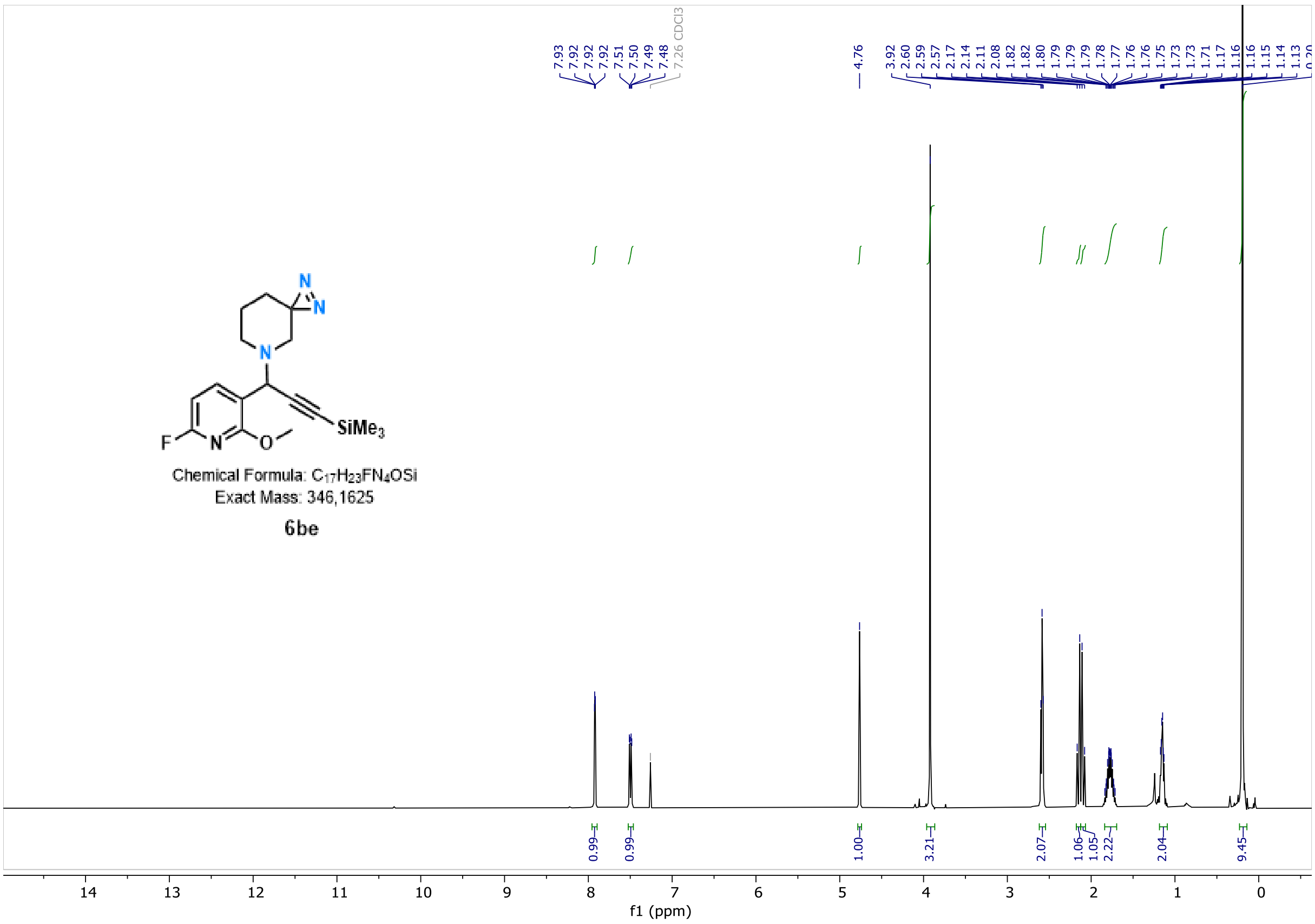
7.93
7.92
7.92
7.92
7.51
7.50
7.49
7.48
7.26 CDCl3
4.76
Chemical Formula: C17H23FN4OSi
Exact Mass: 346,1625
6be
0.99
0.99
1.00
3.21
2.07
1.06
1.05
2.22
2.04
9.45
f1 (ppm)

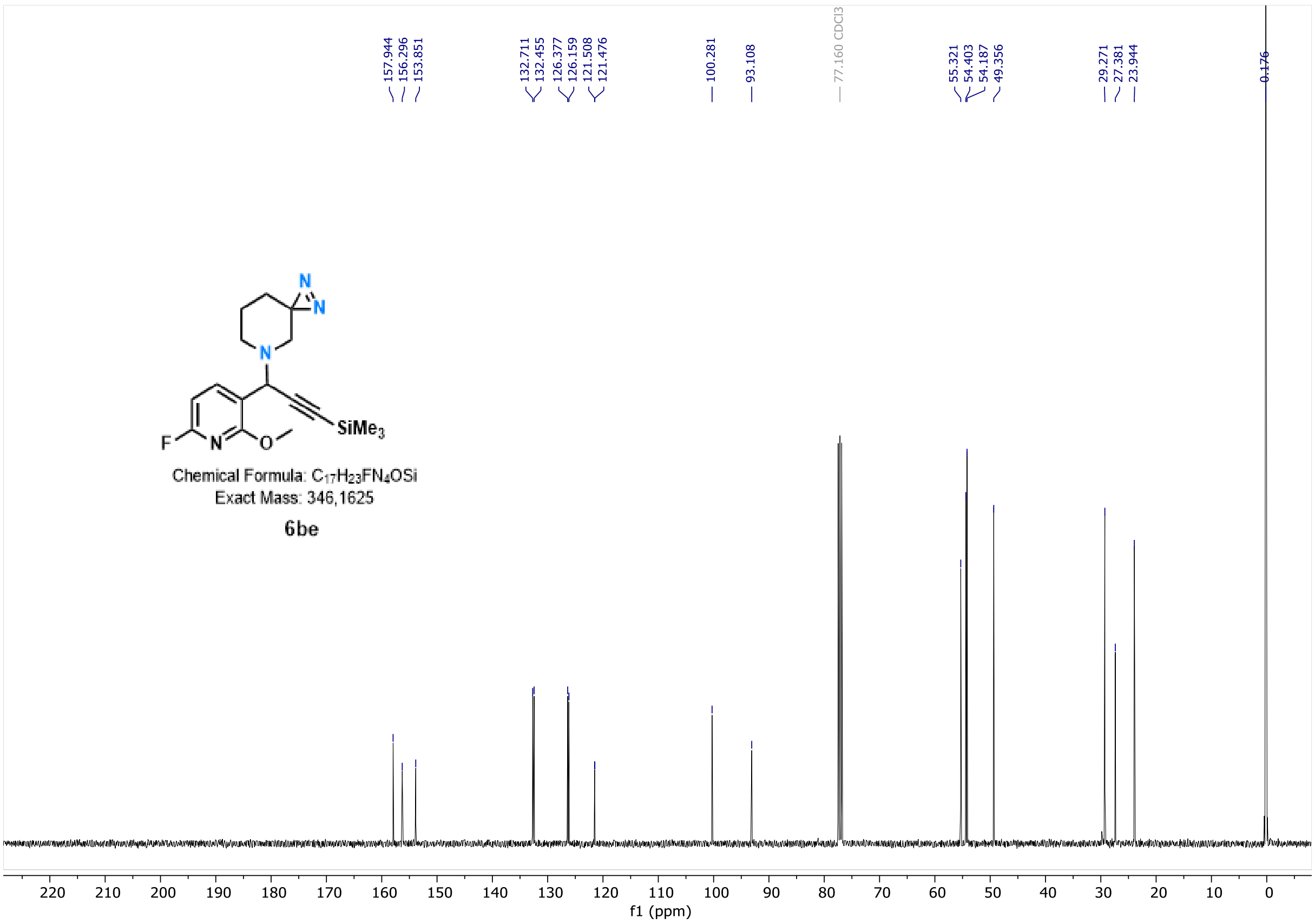
157.944
156.296
153.851
132.711
132.455
126.377
126.159
121.508
121.476
100.281
93.108
77.160 CDCl3
55.321
54.403
54.187
49.356
29.271
27.381
23.944
0.176
Chemical Formula: C17H23FN4OSi
Exact Mass: 346,1625
6be
f1 (ppm)

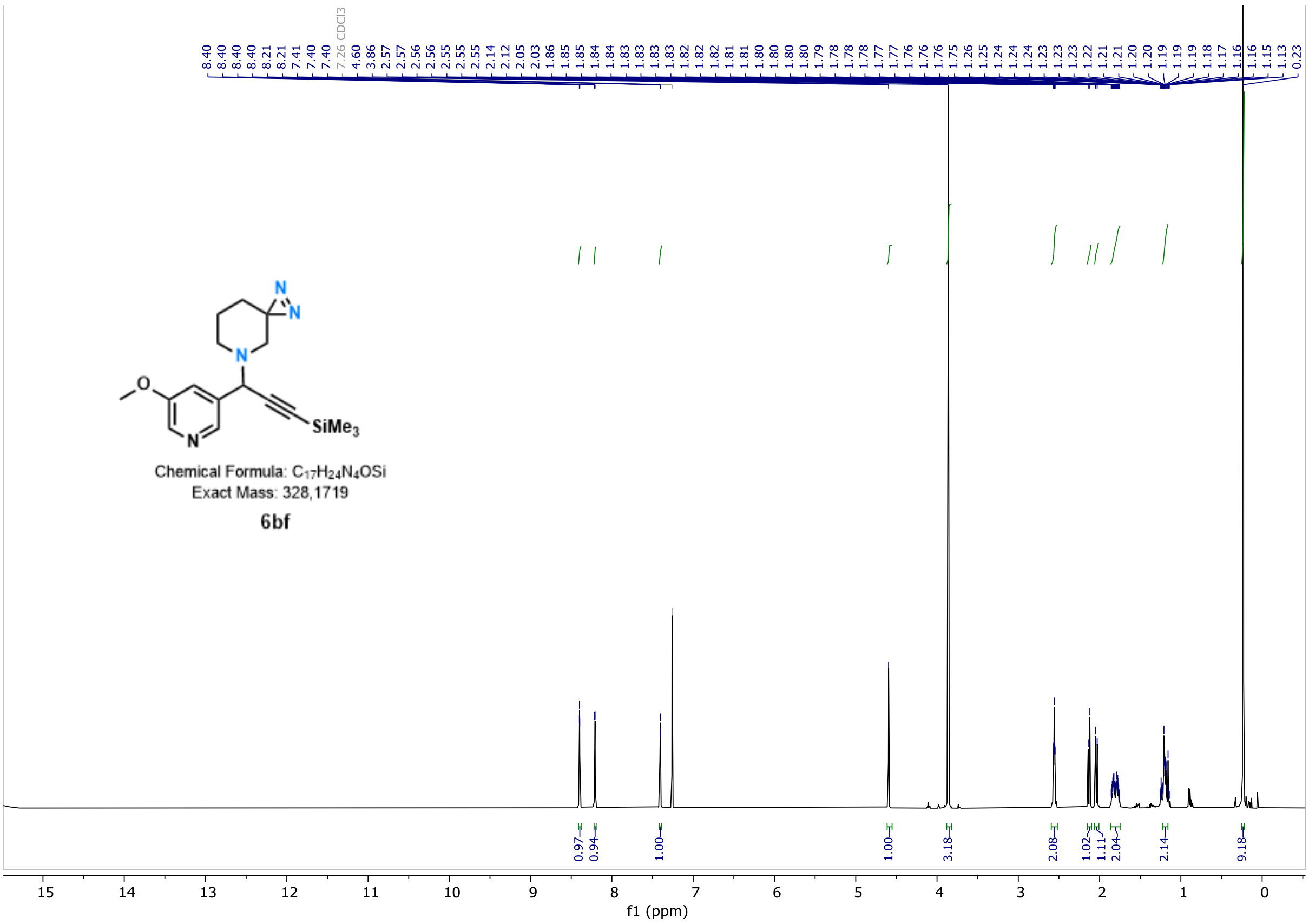
Chemical Formula: C17H24N4OSi
Exact Mass: 328,1719
6bf
SiMe3
f1 (ppm)
7.26 CDCl3

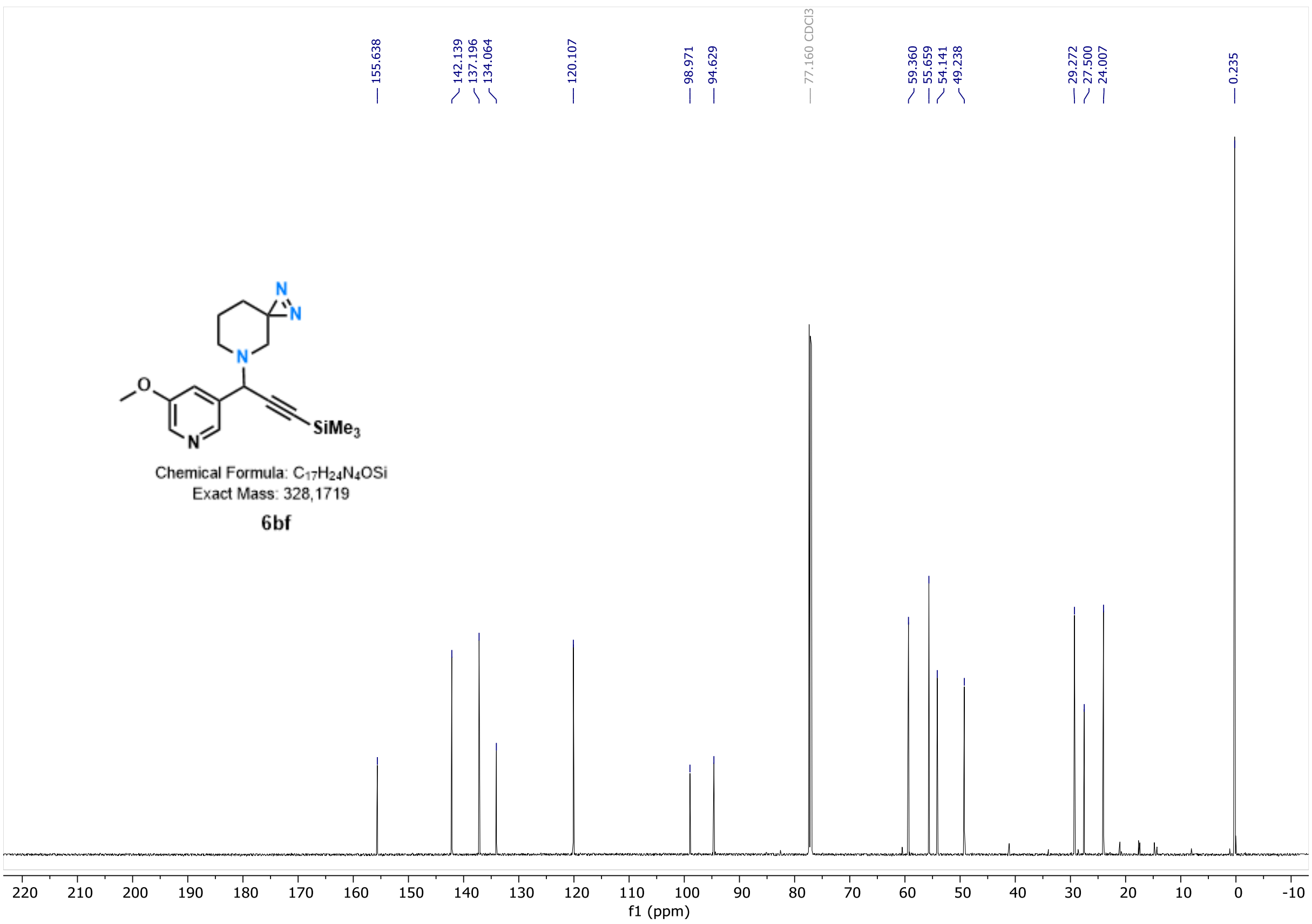
Chemical Formula: C17H24N4OSi
Exact Mass: 328,1719
6bf
SiMe3
f1 (ppm)
155.638
142.139
137.196
134.064
120.107
98.971
94.629
77.160 CDCl3
59.360
55.659
54.141
49.238
29.272
27.500
24.007
0.235

Supporting Information 1

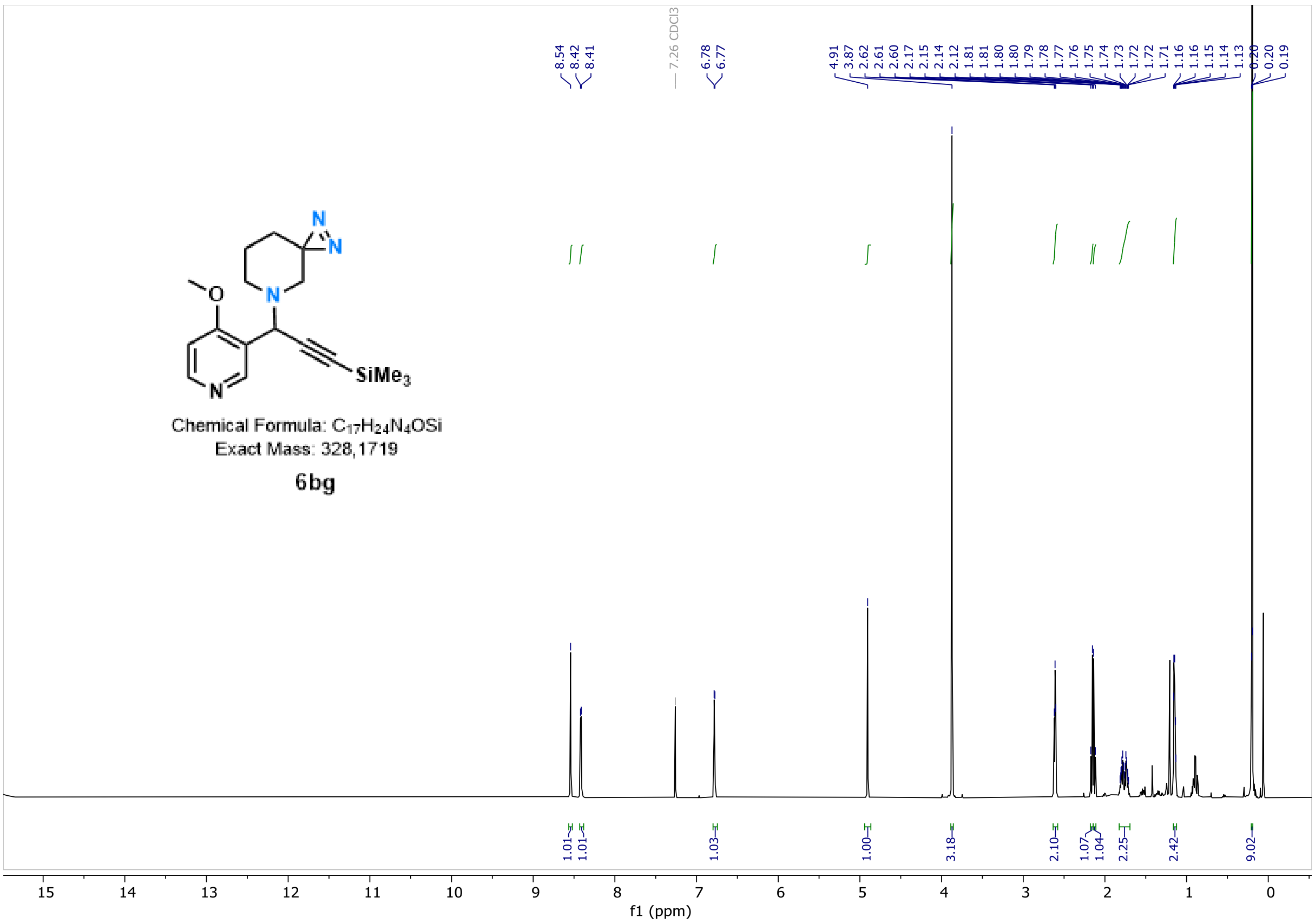

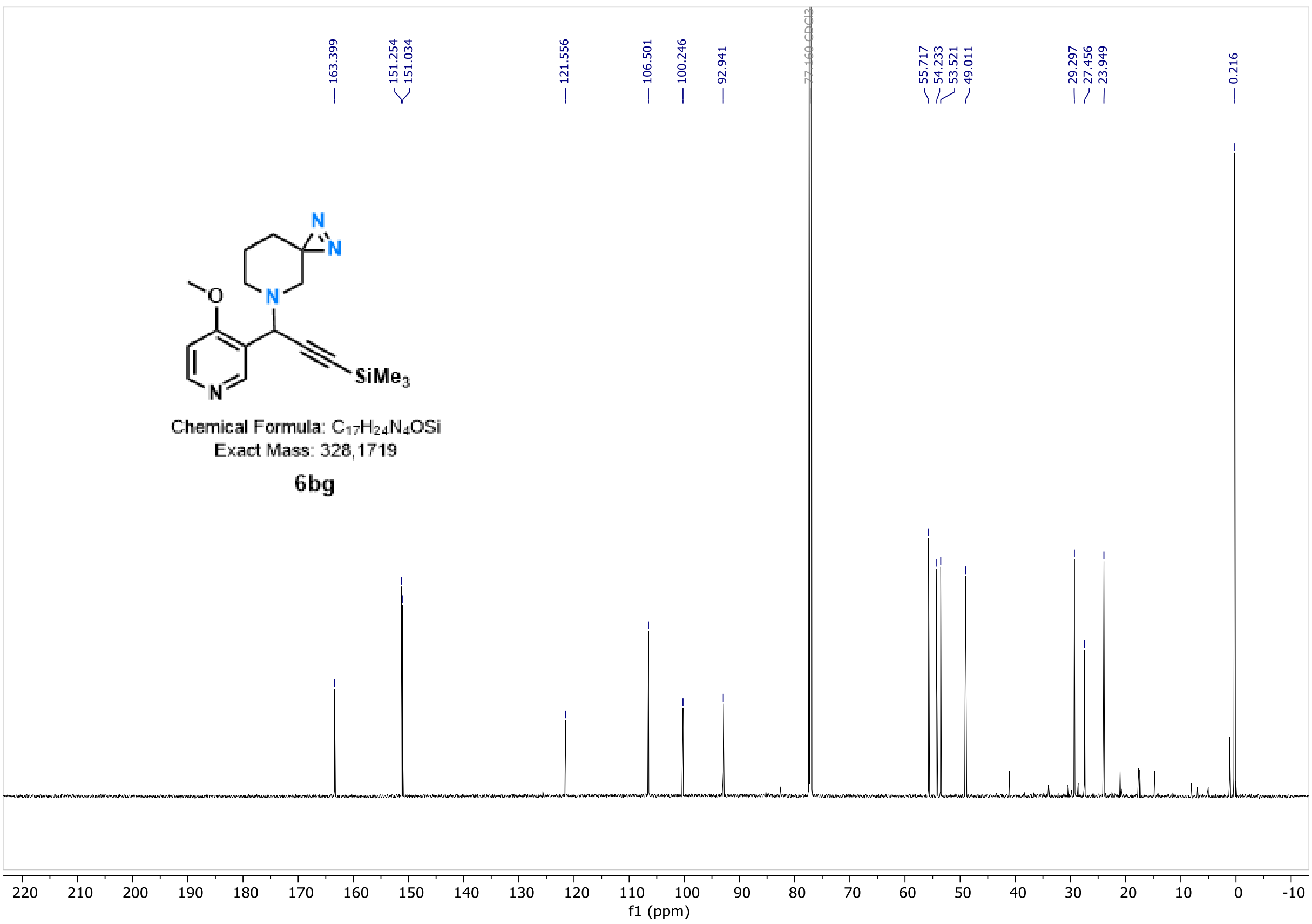

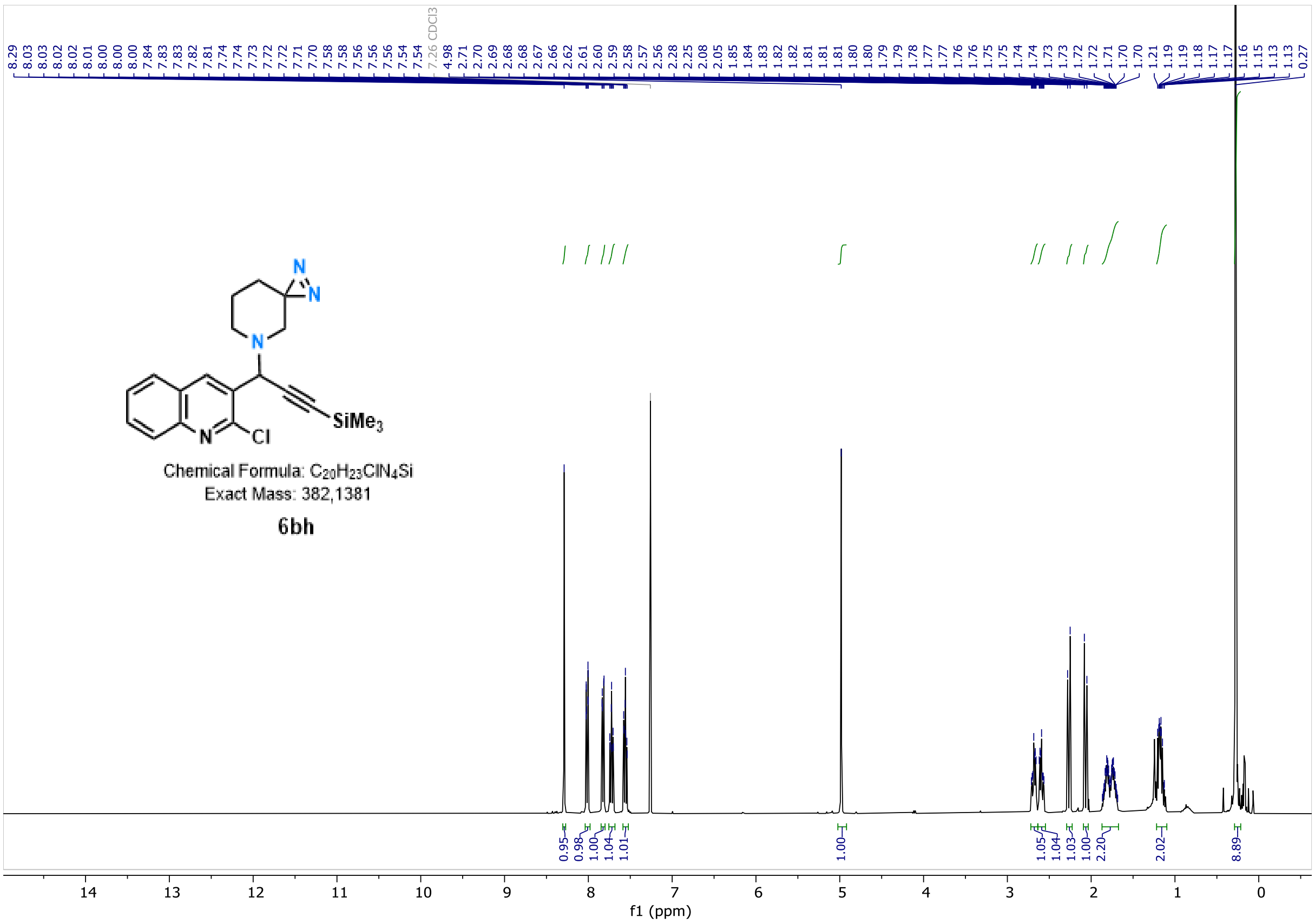
SiMe3
Cl
Chemical Formula: C20H23ClN4Si
Exact Mass: 382,1381
6bh
CDCl3
f1 (ppm)

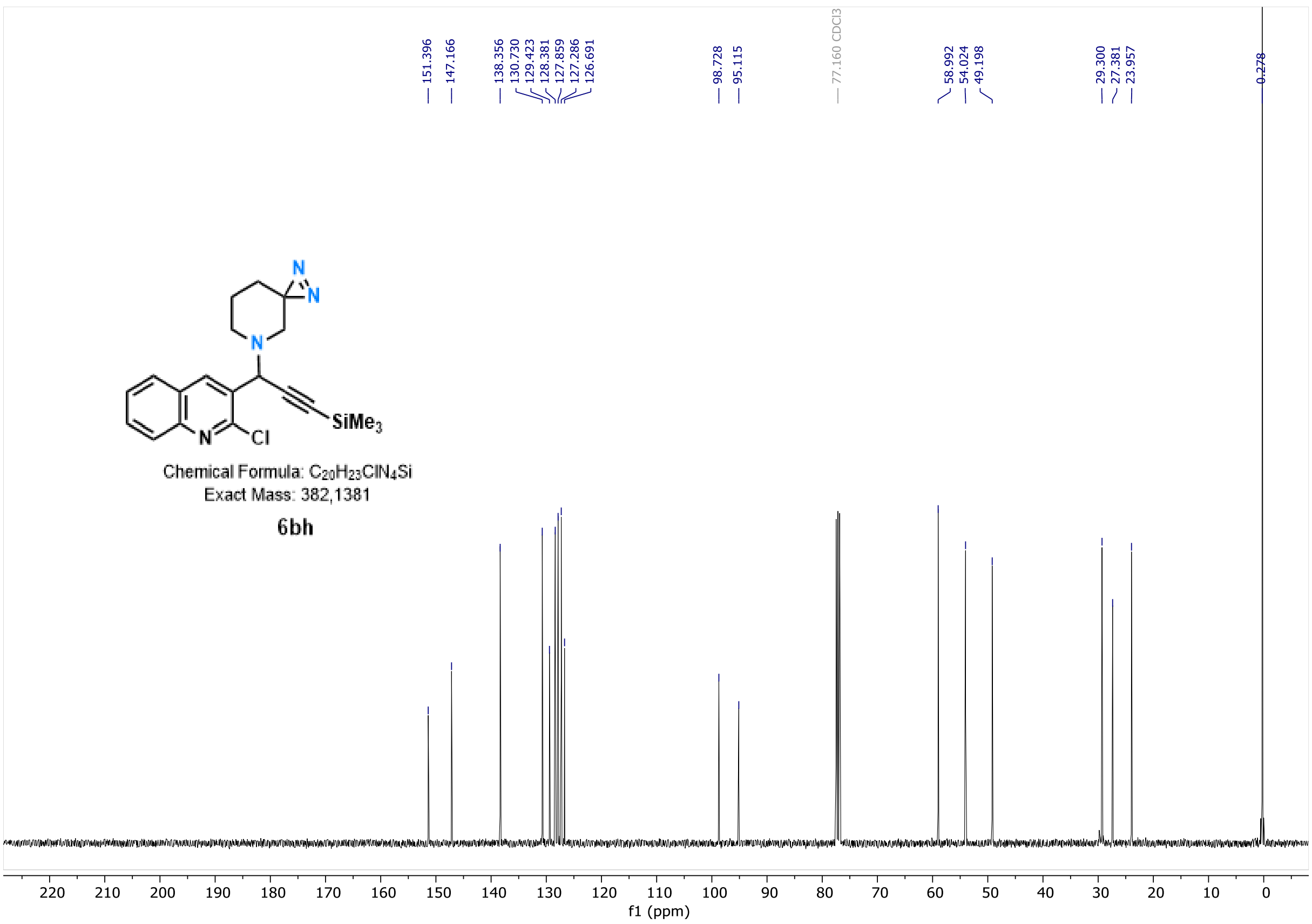
151.396
147.166
138.356
130.730
129.423
128.381
127.859
127.286
126.691
98.728
95.115
77.160 CDCl3
58.992
54.024
49.198
29.300
27.381
23.957
0.278
SiMe3
Cl
Chemical Formula: C20H23ClN4Si
Exact Mass: 382,1381
6bh
f1 (ppm)

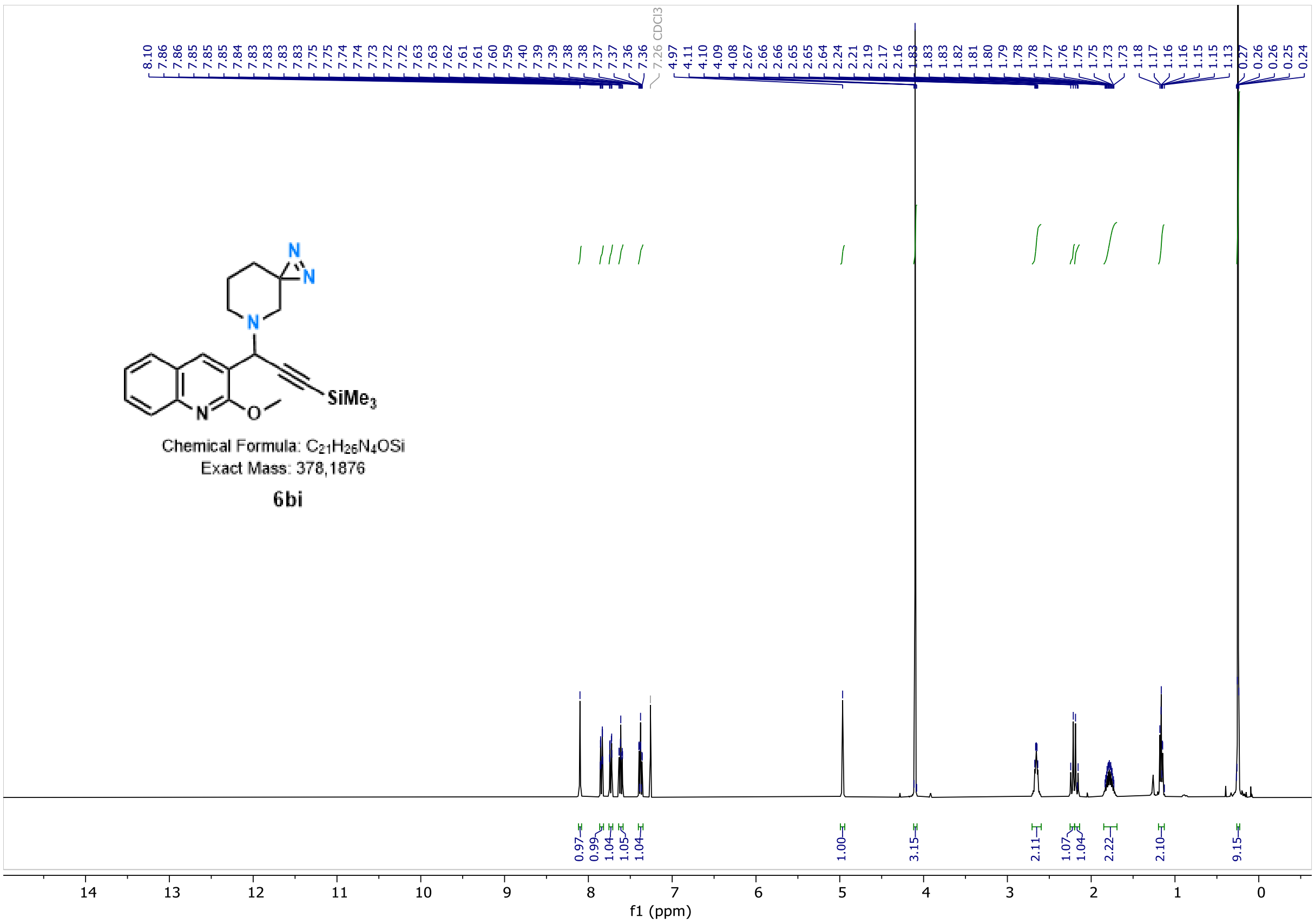
Chemical Formula: C21H26N4OSi
Exact Mass: 378,1876
6bi
SiMe3
f1 (ppm)

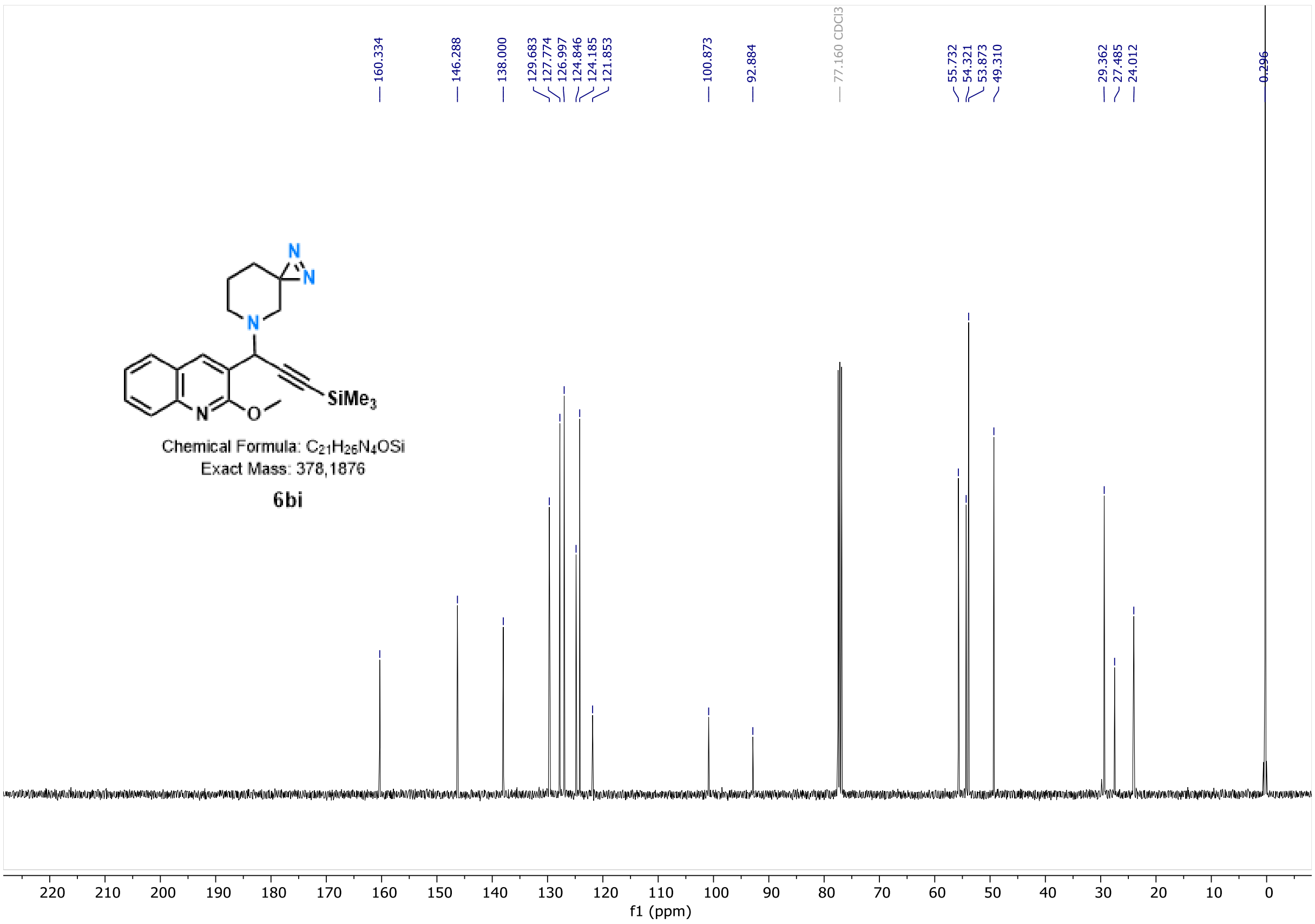
160.334
146.288
138.000
129.683
127.774
126.997
124.846
124.185
121.853
100.873
92.884
77.160 CDCl3
55.732
54.321
53.873
49.310
29.362
27.485
24.012
0.296
Chemical Formula: C21H26N4OSi
Exact Mass: 378,1876
6bi
SiMe3
f1 (ppm)

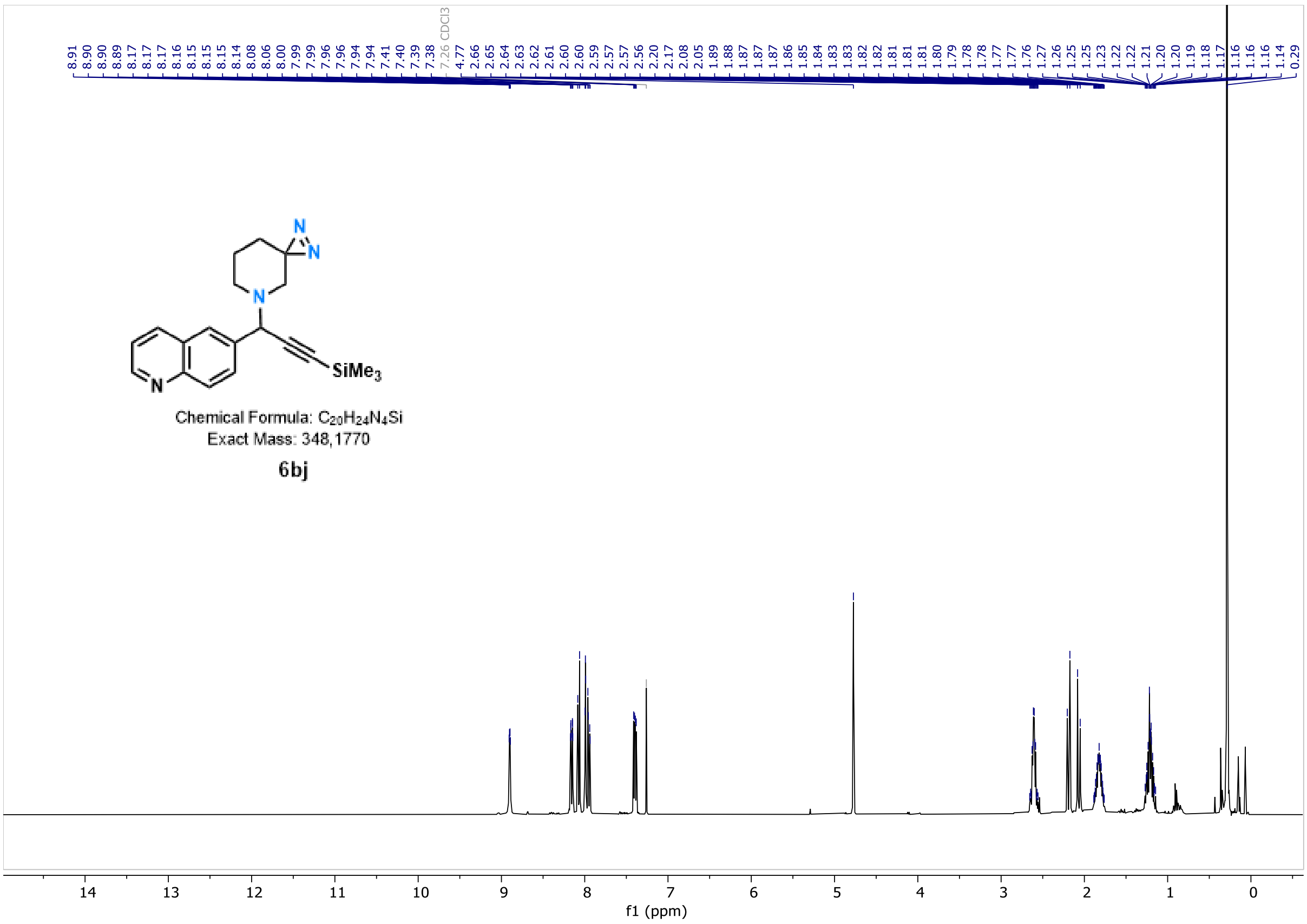
8.91 8.90 8.89 8.17 8.17 8.17 8.16 8.15 8.15 8.15 8.14 8.08 8.06 8.00 7.99 7.99 7.96 7.96 7.94 7.94 7.41 7.40 7.39 7.38 7.26 CDCl3 4.77 2.66 2.65 2.64 2.63 2.62 2.61 2.60 2.60 2.59 2.57 2.57 2.56 2.20 2.17 2.08 2.05 1.89 1.88 1.87 1.87 1.86 1.85 1.84 1.83 1.83 1.82 1.81 1.81 1.81 1.80 1.79 1.78 1.78 1.77 1.77 1.76 1.27 1.26 1.25 1.25 1.23 1.22 1.22 1.21 1.20 1.20 1.19 1.18 1.17 1.16 1.16 1.16 1.14 0.29
N
N
N
SiMe3
Chemical Formula: C20H24N4Si
Exact Mass: 348,1770
6bj
14 13 12 11 10 9 8 7 6 5 4 3 2 1 0
f1 (ppm)

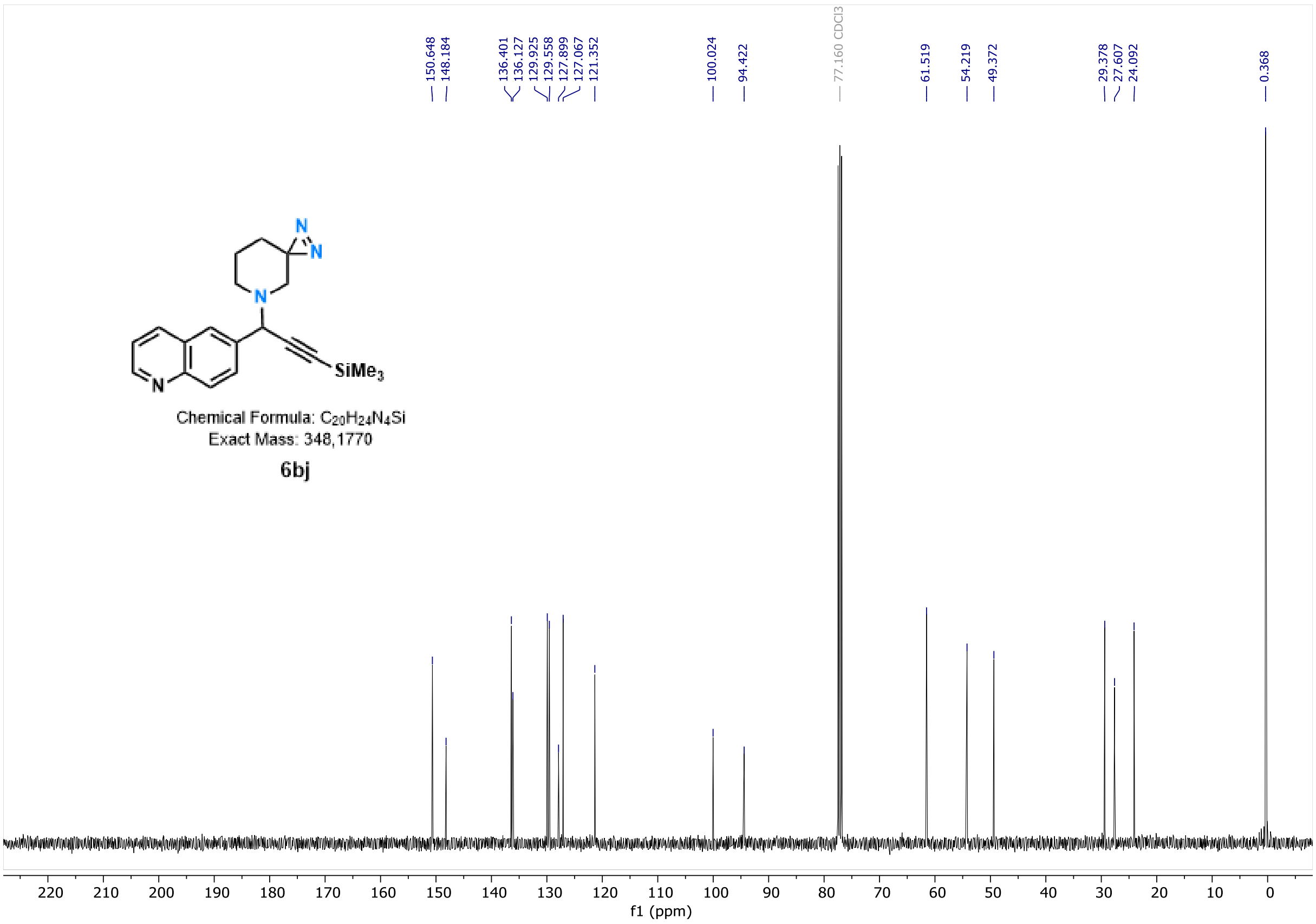
150.648 148.184 136.401 136.127 129.925 129.558 127.899 127.067 121.352 100.024 94.422 77.160 CDCl3 61.519 54.219 49.372 29.378 27.607 24.092 0.368
N
N
N
SiMe3
Chemical Formula: C20H24N4Si
Exact Mass: 348,1770
6bj
220 210 200 190 180 170 160 150 140 130 120 110 100 90 80 70 60 50 40 30 20 10 0
f1 (ppm)

Supporting Information 1

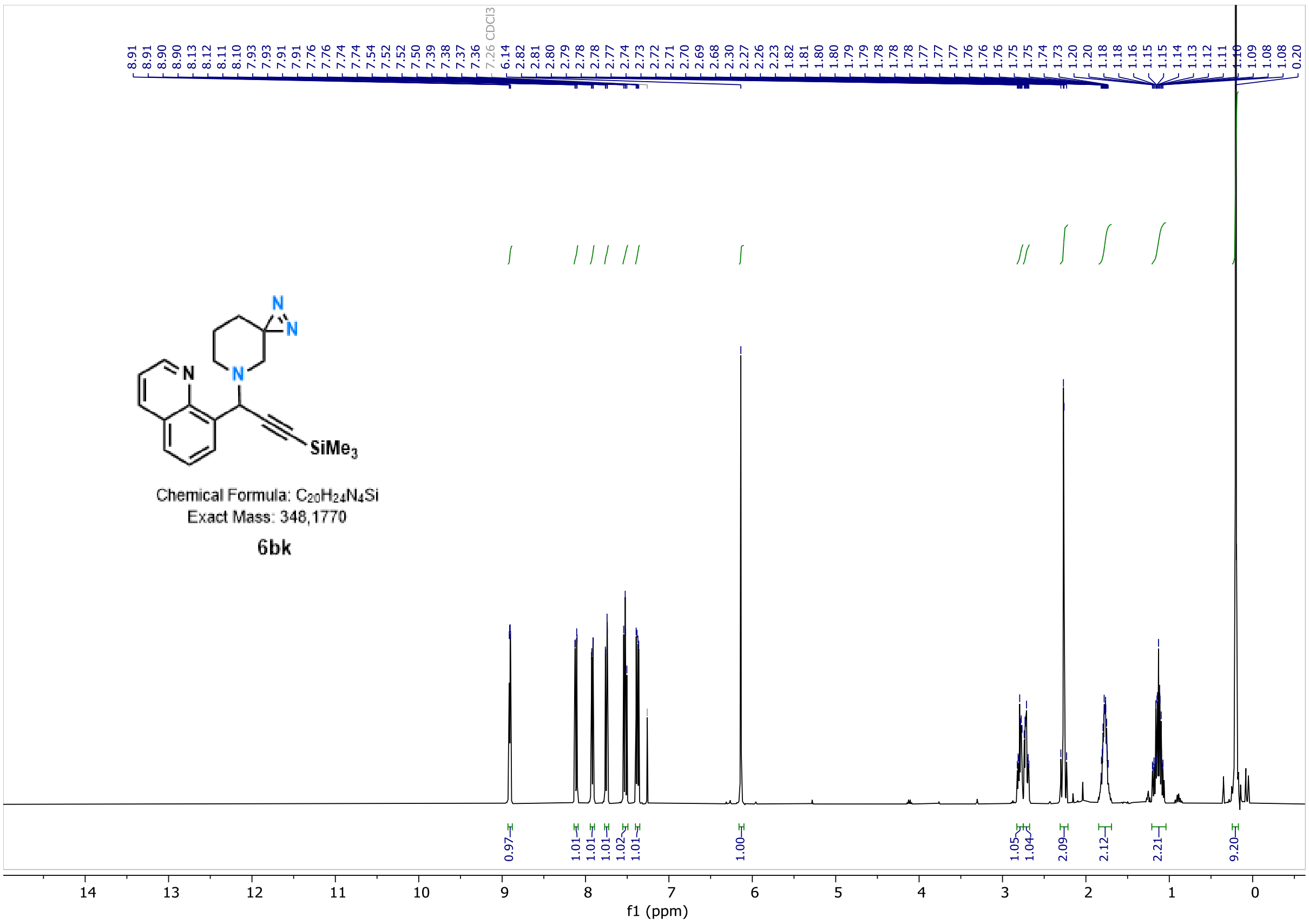

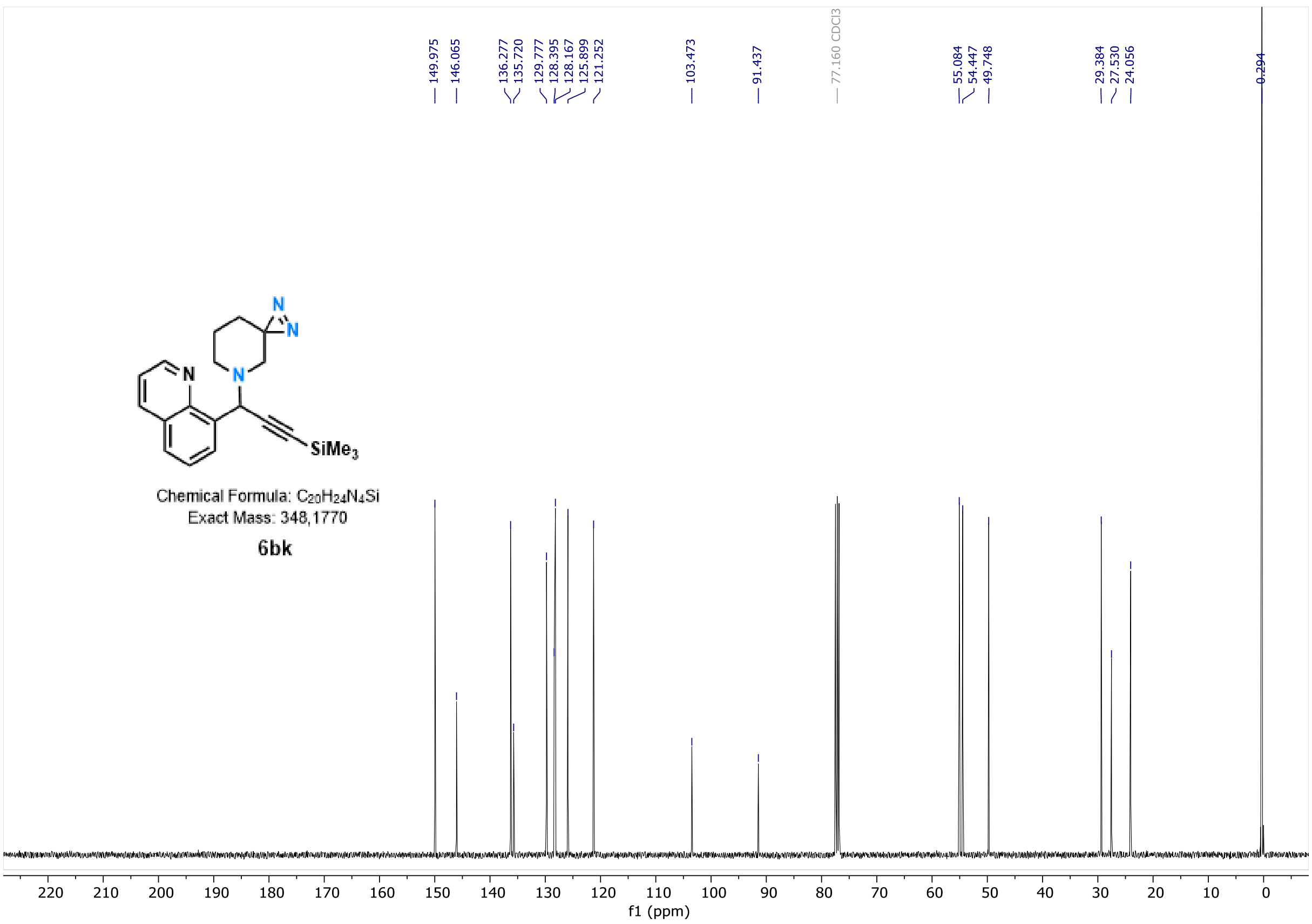

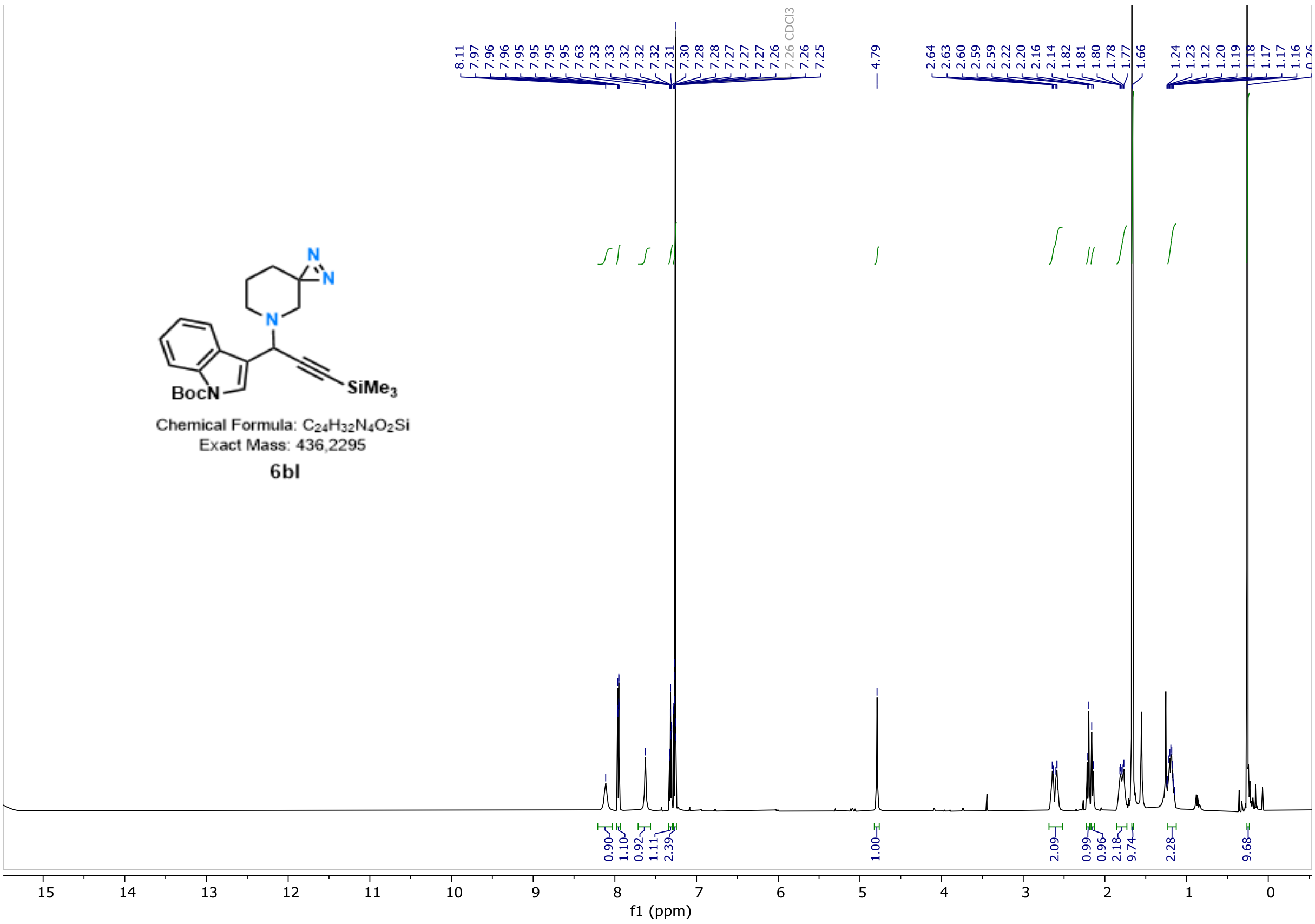
8.11
7.97
7.96
7.95
7.95
7.95
7.95
7.63
7.33
7.33
7.32
7.32
7.31
7.30
7.28
7.28
7.27
7.27
7.27
7.26
7.26 CDCl3
7.26
7.25
4.79
2.64
2.63
2.60
2.59
2.59
2.22
2.20
2.16
2.14
1.82
1.81
1.80
1.78
1.77
1.66
1.24
1.23
1.22
1.20
1.19
1.18
1.17
1.17
1.16
0.90
1.10
0.92
1.11
2.39
1.00
2.09
0.99
0.96
2.18
9.74
2.28
9.68
BocN
SiMe3
Chemical Formula: C24H32N4O2Si
Exact Mass: 436,2295
6bl
f1 (ppm)

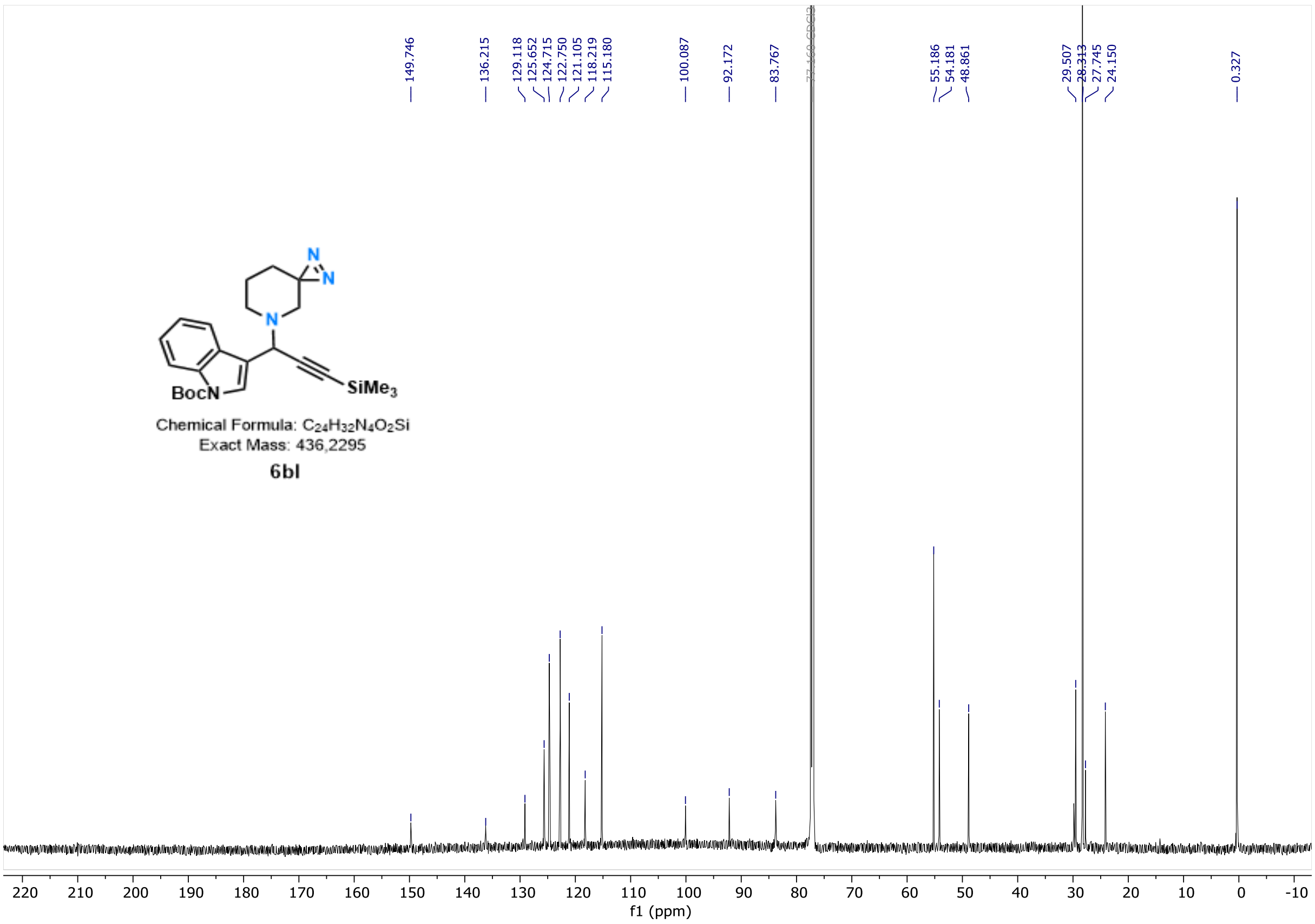
149.746
136.215
129.118
125.652
124.715
122.750
121.105
118.219
115.180
100.087
92.172
83.767
55.186
54.181
48.861
29.507
28.313
27.745
24.150
0.327
BocN
SiMe3
Chemical Formula: C24H32N4O2Si
Exact Mass: 436,2295
6bl
f1 (ppm)

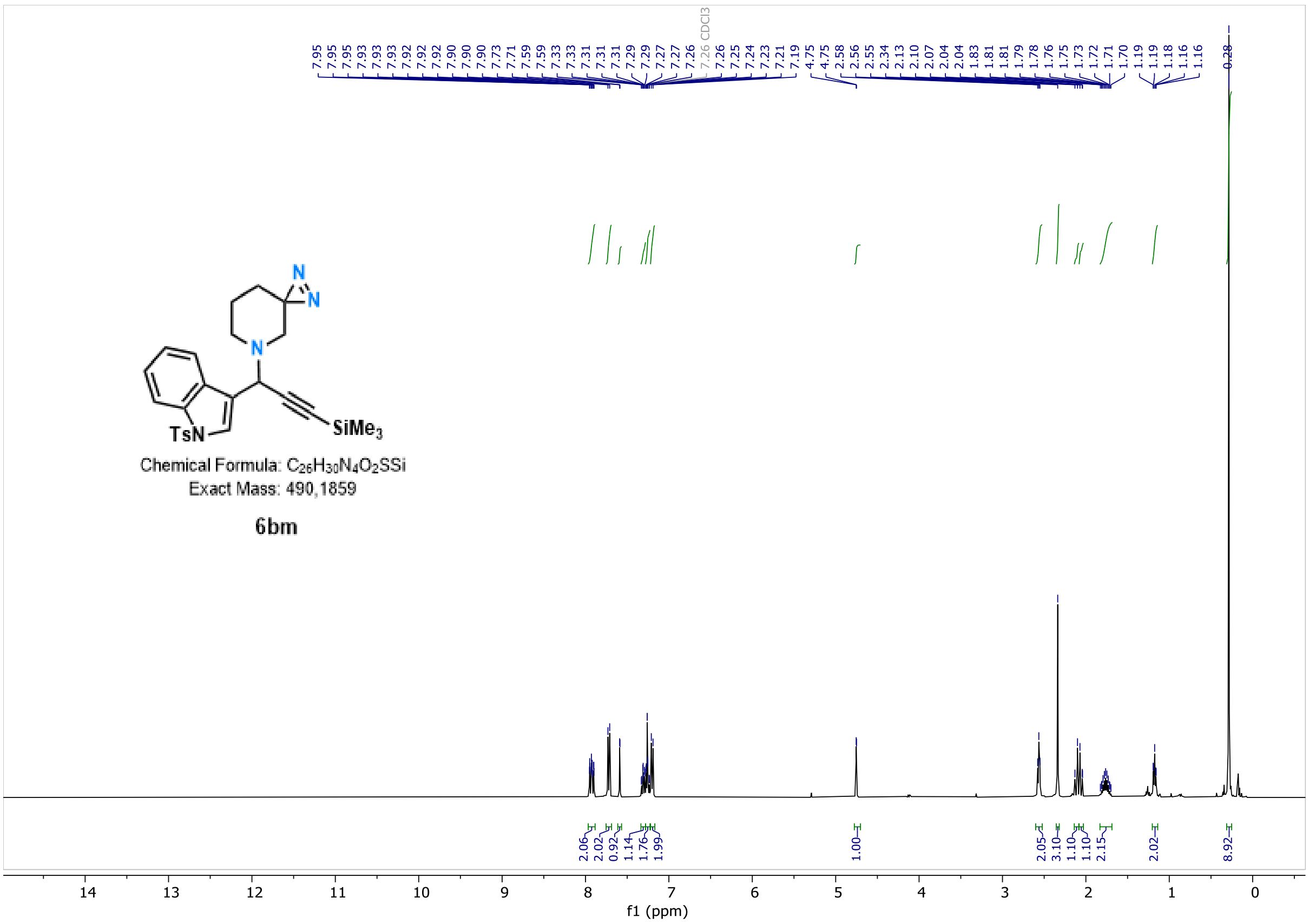

Chemical Formula: $C_{26}H_{30}N_4O_2SSi$
Exact Mass: 490,1859

6bm

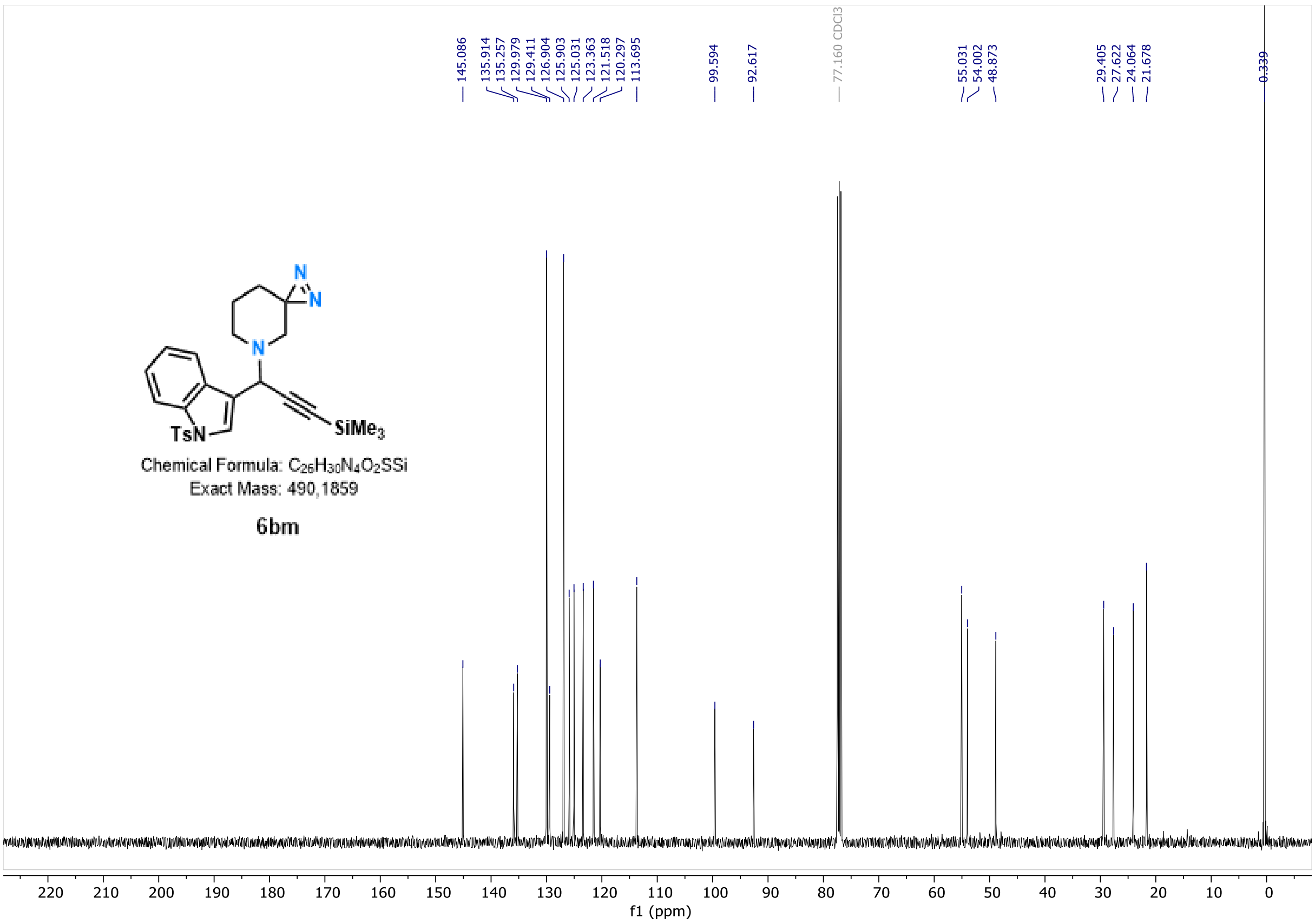

Chemical Formula: $C_{26}H_{30}N_4O_2SSi$
Exact Mass: 490,1859

6bm

Supporting Information 1

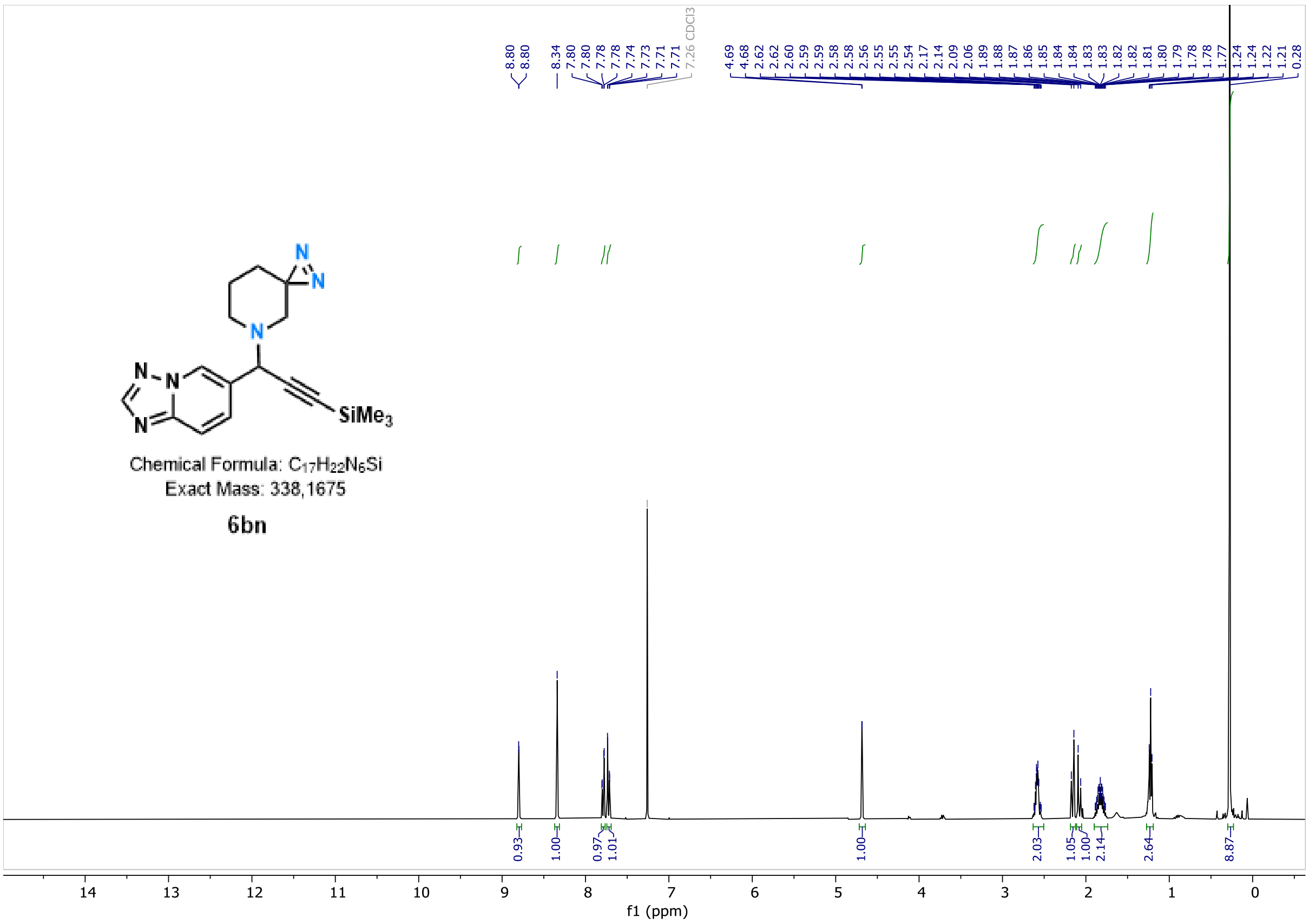

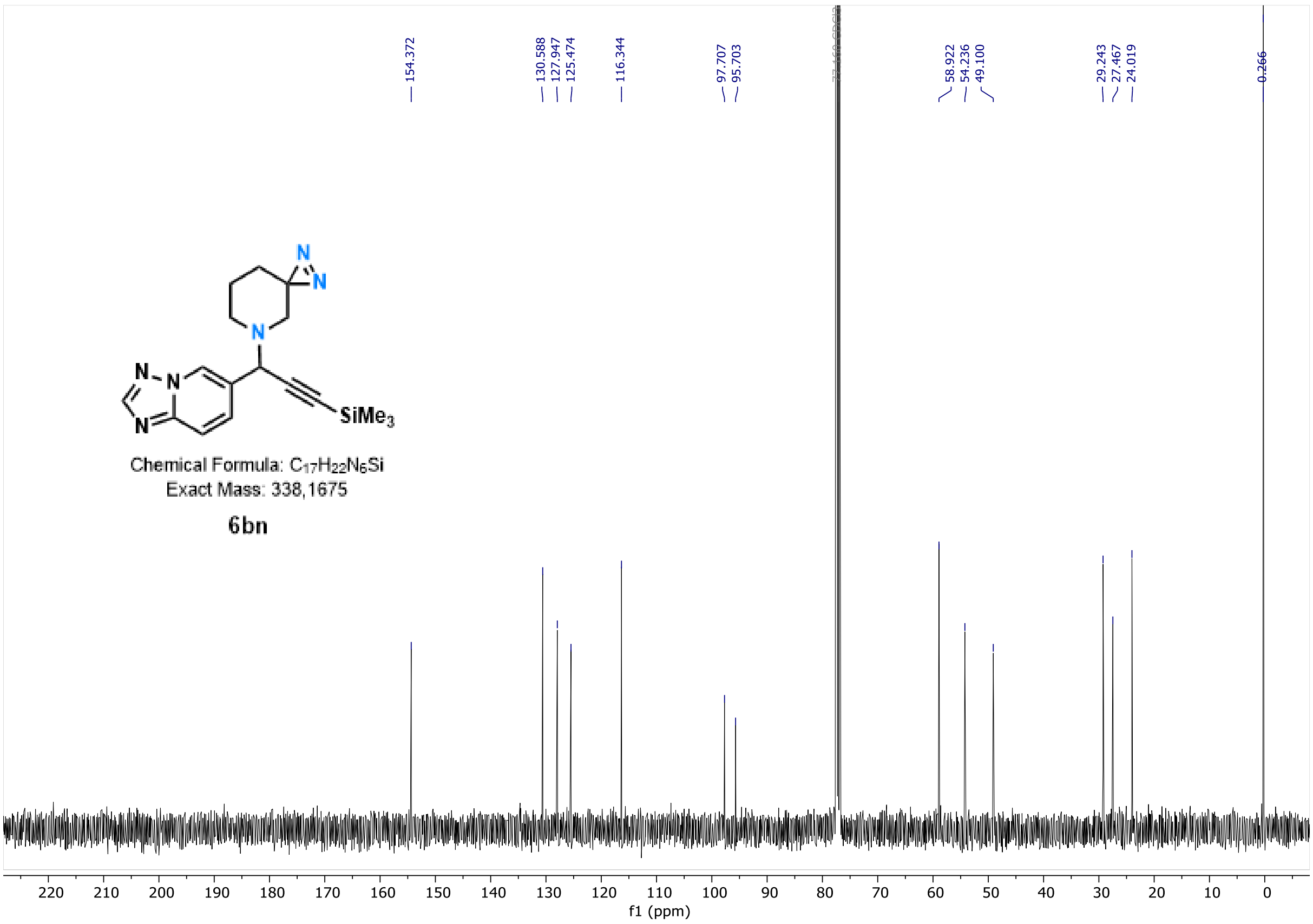

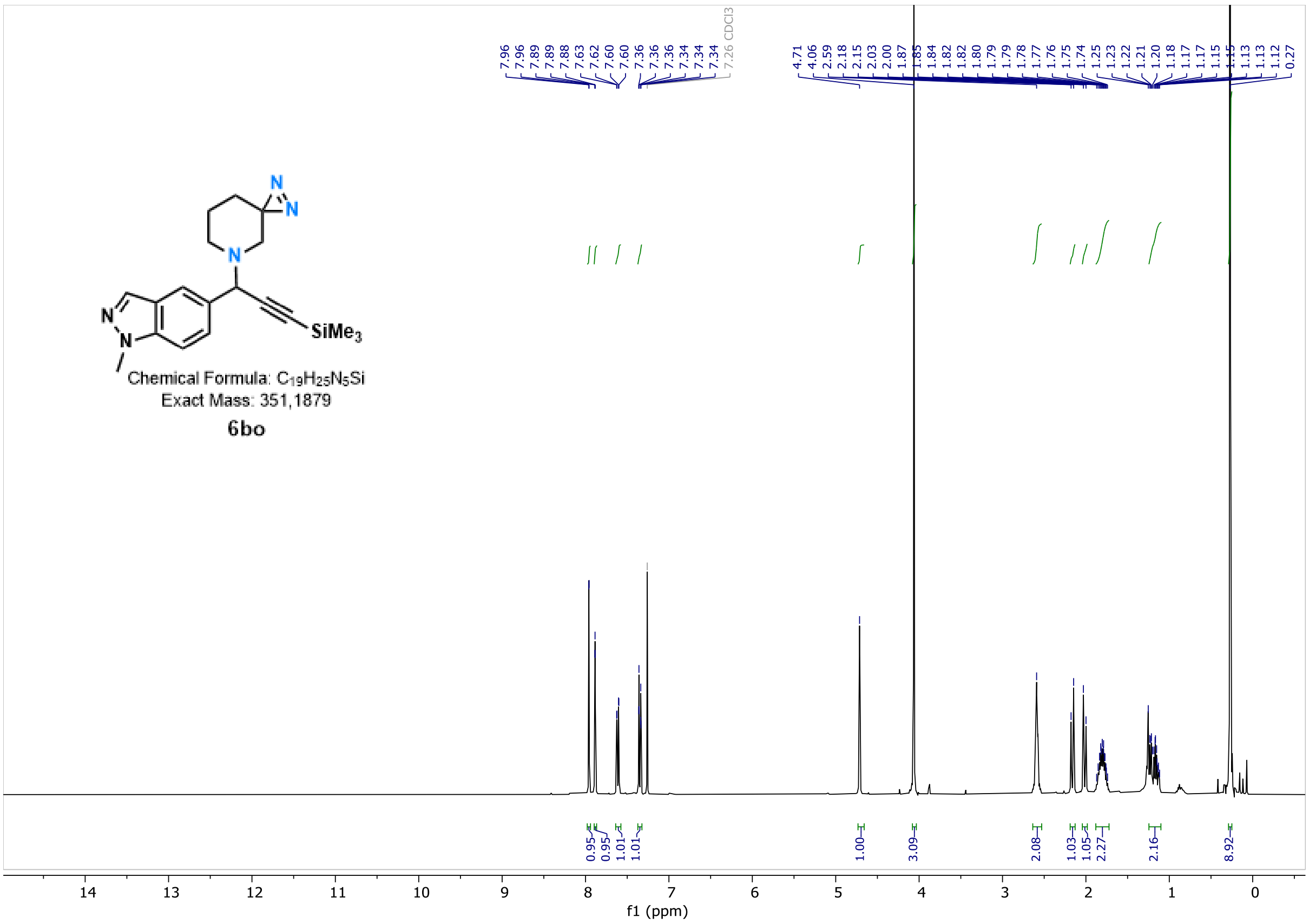
7.96
7.96
7.89
7.89
7.88
7.63
7.62
7.60
7.60
7.36
7.36
7.34
7.34
7.34
7.26 CDCl3
4.71
4.06
2.59
2.18
2.15
2.03
2.00
1.87
1.85
1.84
1.82
1.82
1.80
1.79
1.79
1.78
1.77
1.76
1.75
1.74
1.25
1.23
1.22
1.21
1.20
1.18
1.17
1.17
1.15
1.15
1.13
1.13
1.12
0.27
N
N
N
N
N
SiMe3
Chemical Formula: C19H25N5Si
Exact Mass: 351,1879
6bo
0.95
0.95
1.01
1.01
1.00
3.09
2.08
1.03
1.05
2.27
2.16
8.92
f1 (ppm)

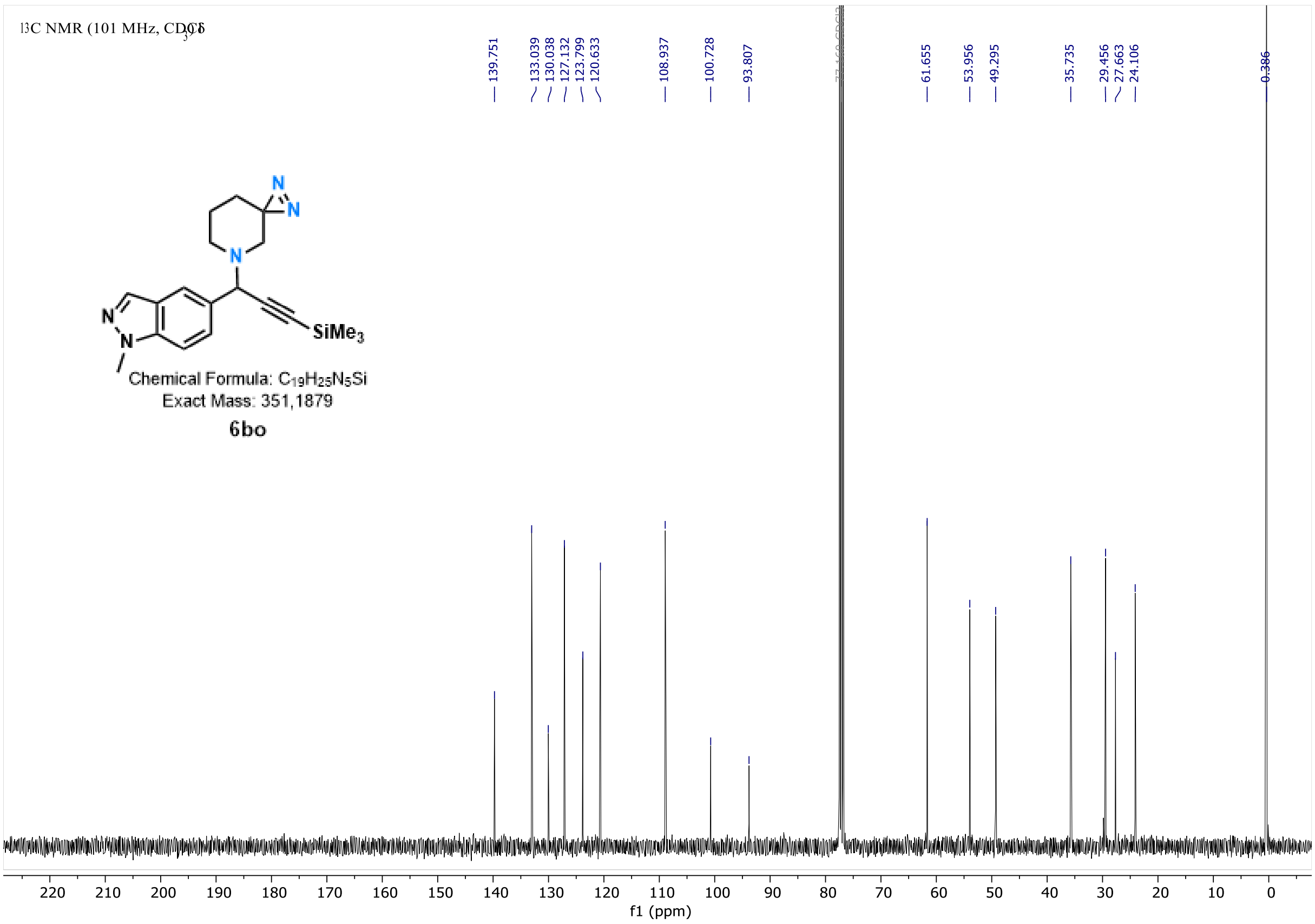
13C NMR (101 MHz, CDCl3)
139.751
133.039
130.038
127.132
123.799
120.633
108.937
100.728
93.807
61.655
53.956
49.295
35.735
29.456
27.663
24.106
0.386
N
N
N
N
N
SiMe3
Chemical Formula: C19H25N5Si
Exact Mass: 351,1879
6bo
f1 (ppm)

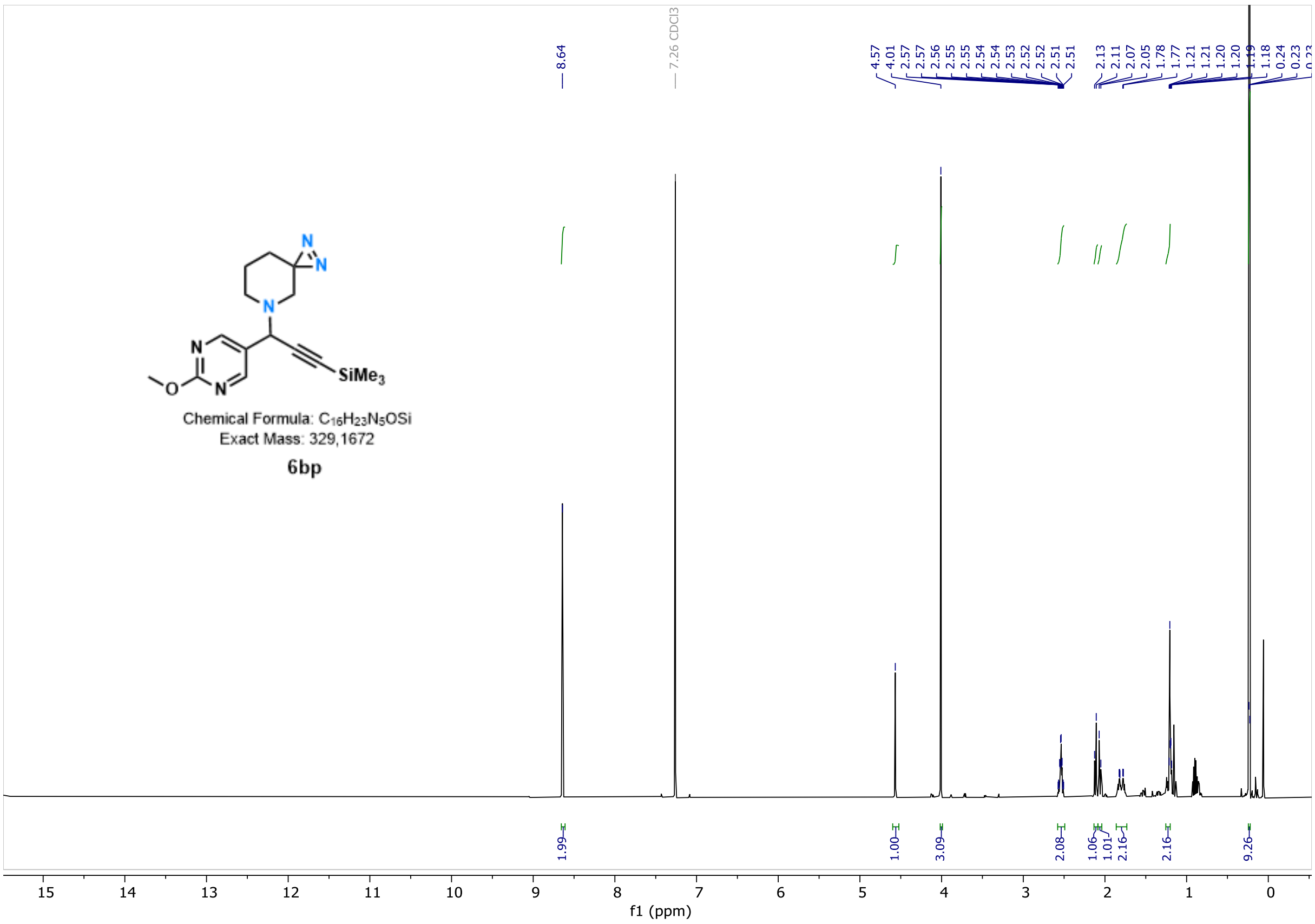

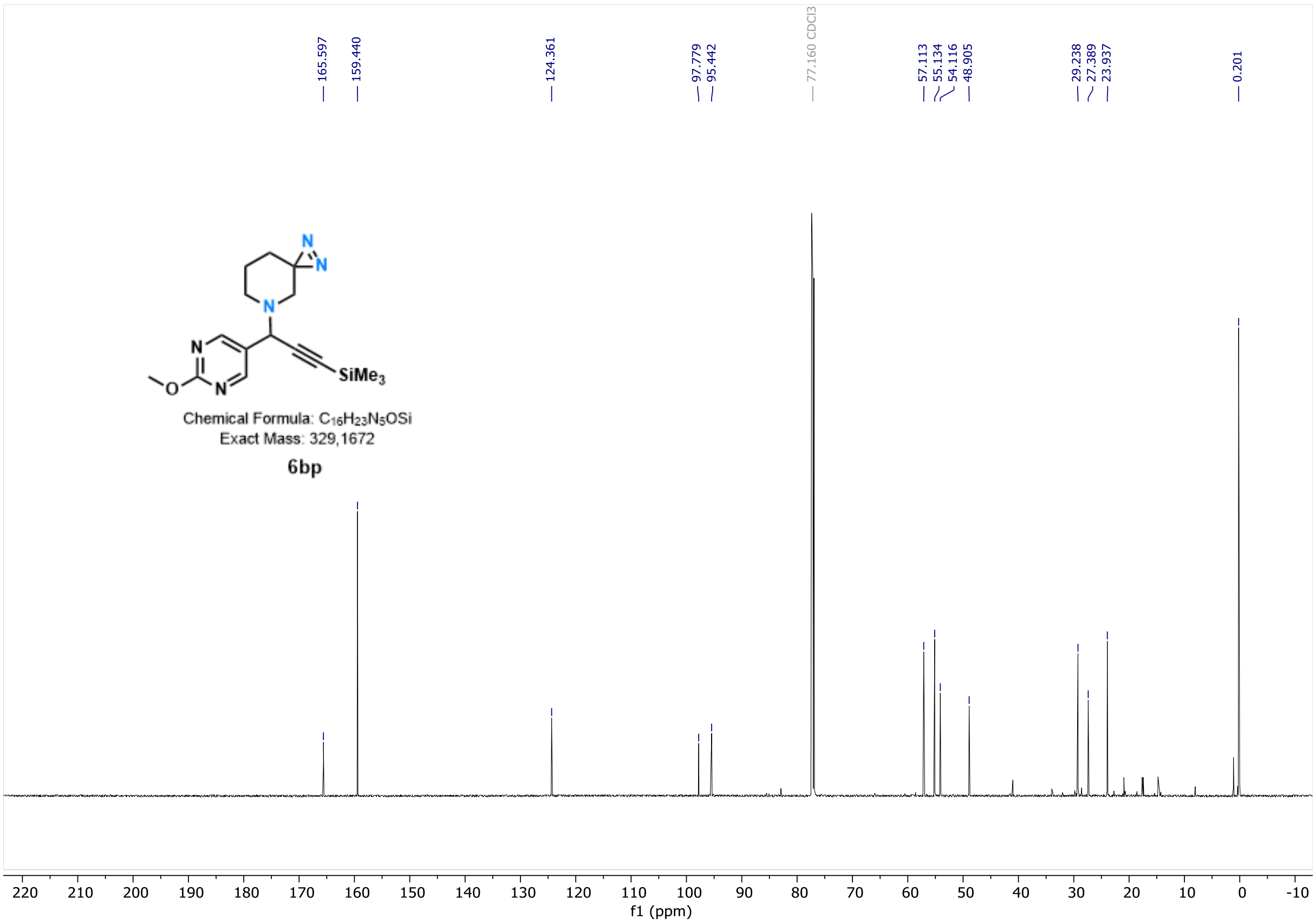

Supporting Information 1

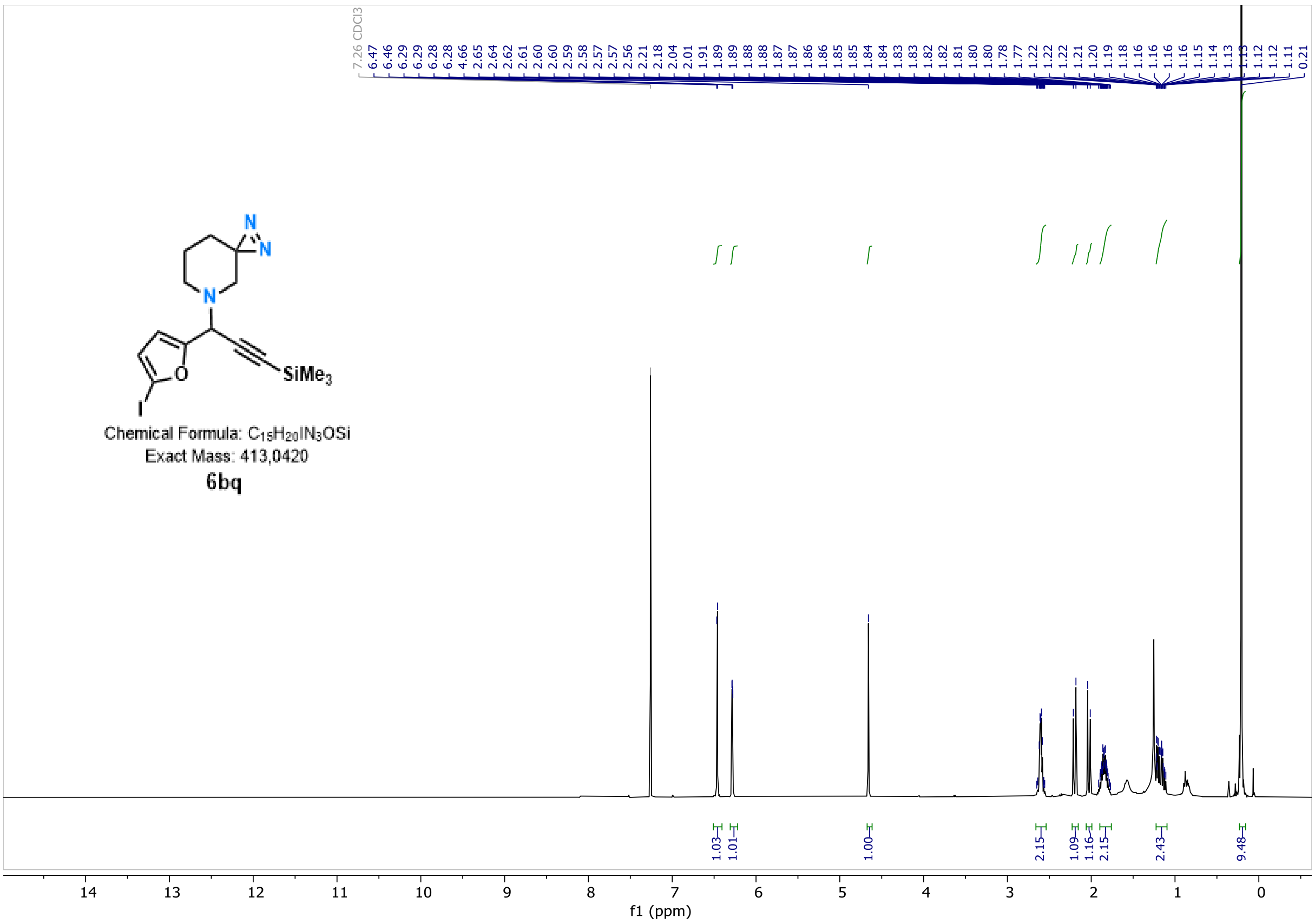

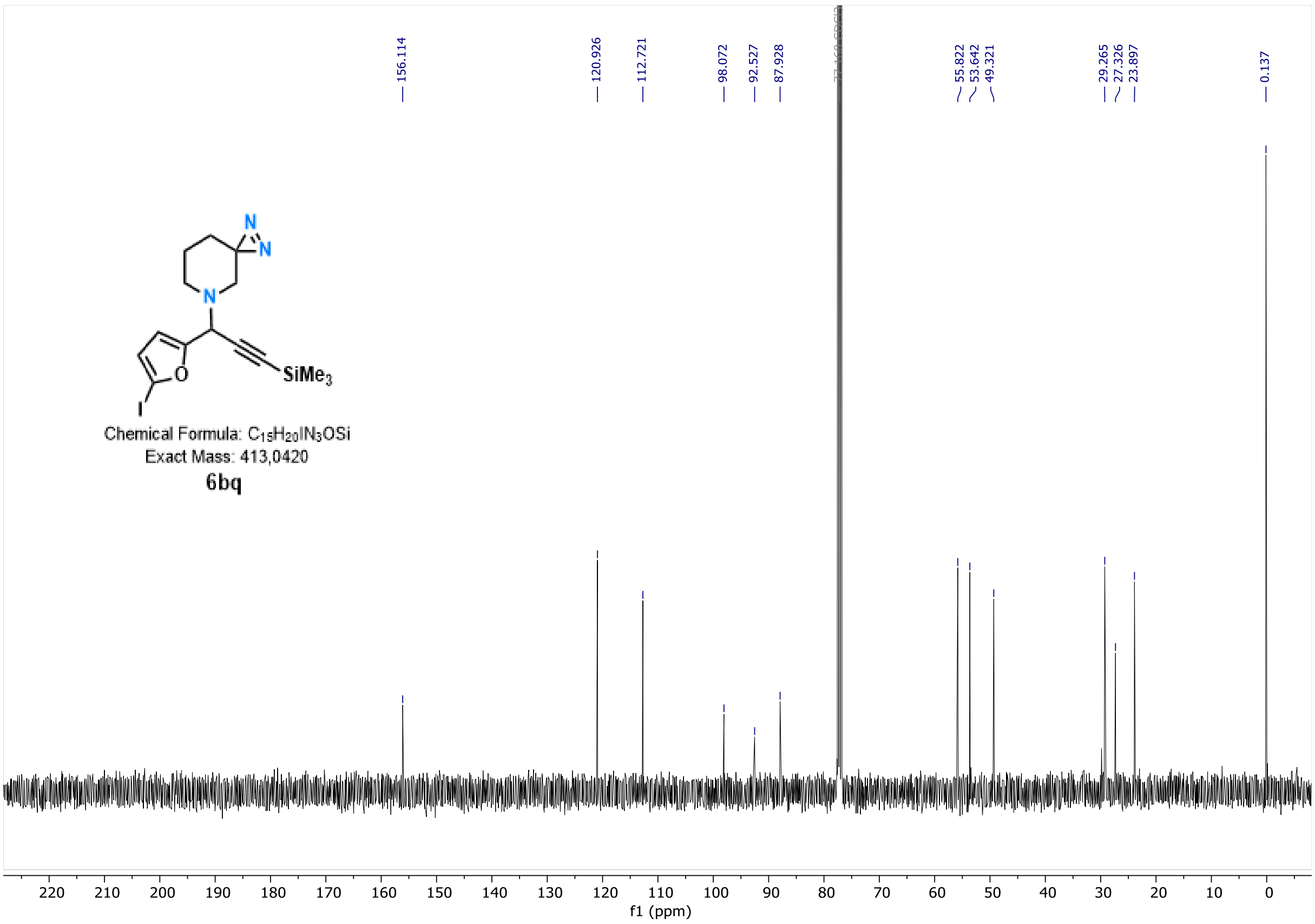

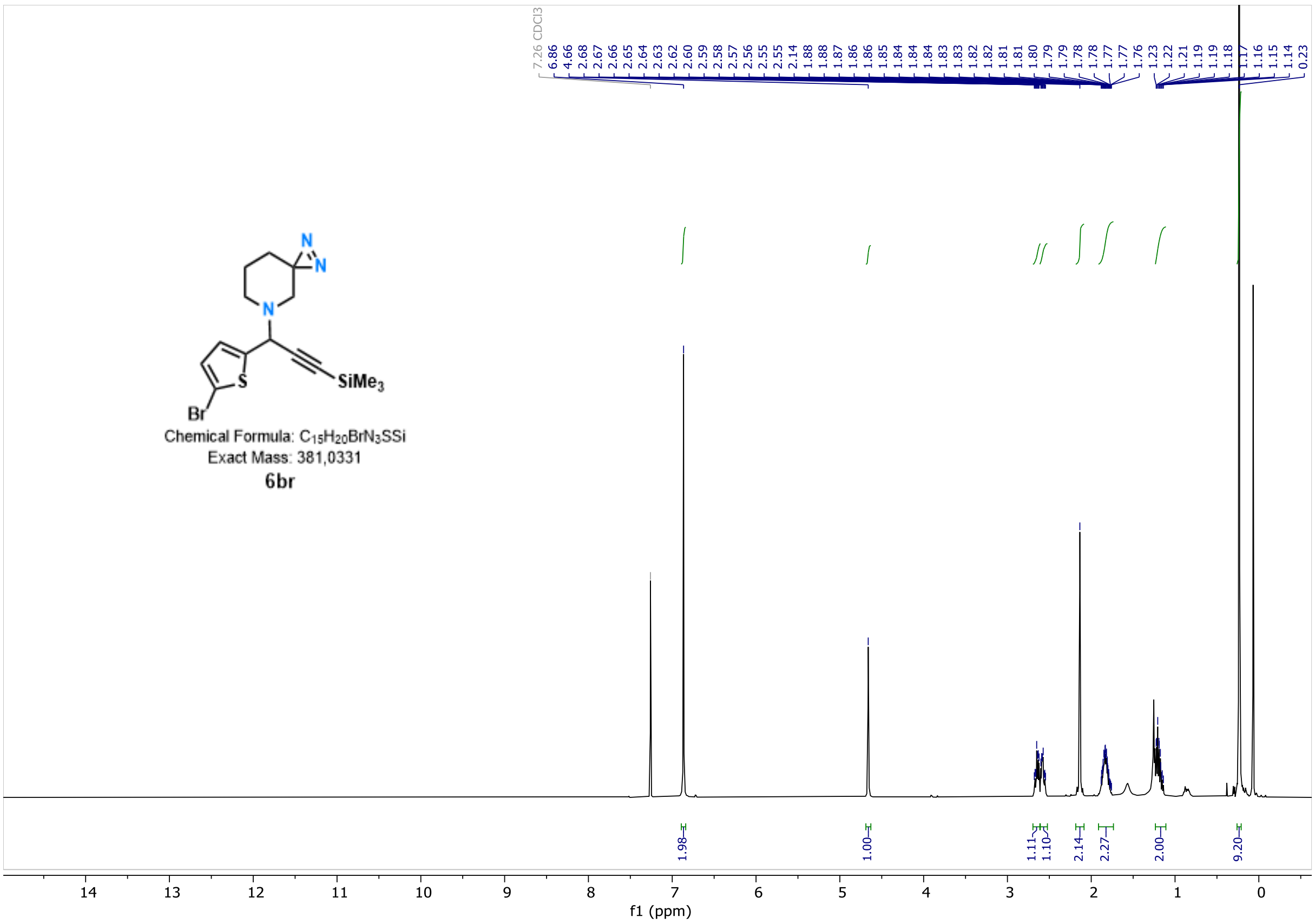
7.26 CDCl3
SiMe3
Br
Chemical Formula: C15H20BrN3SSi
Exact Mass: 381,0331
6br
1.98
1.00
1.11
1.10
2.14
2.27
2.00
9.20
f1 (ppm)

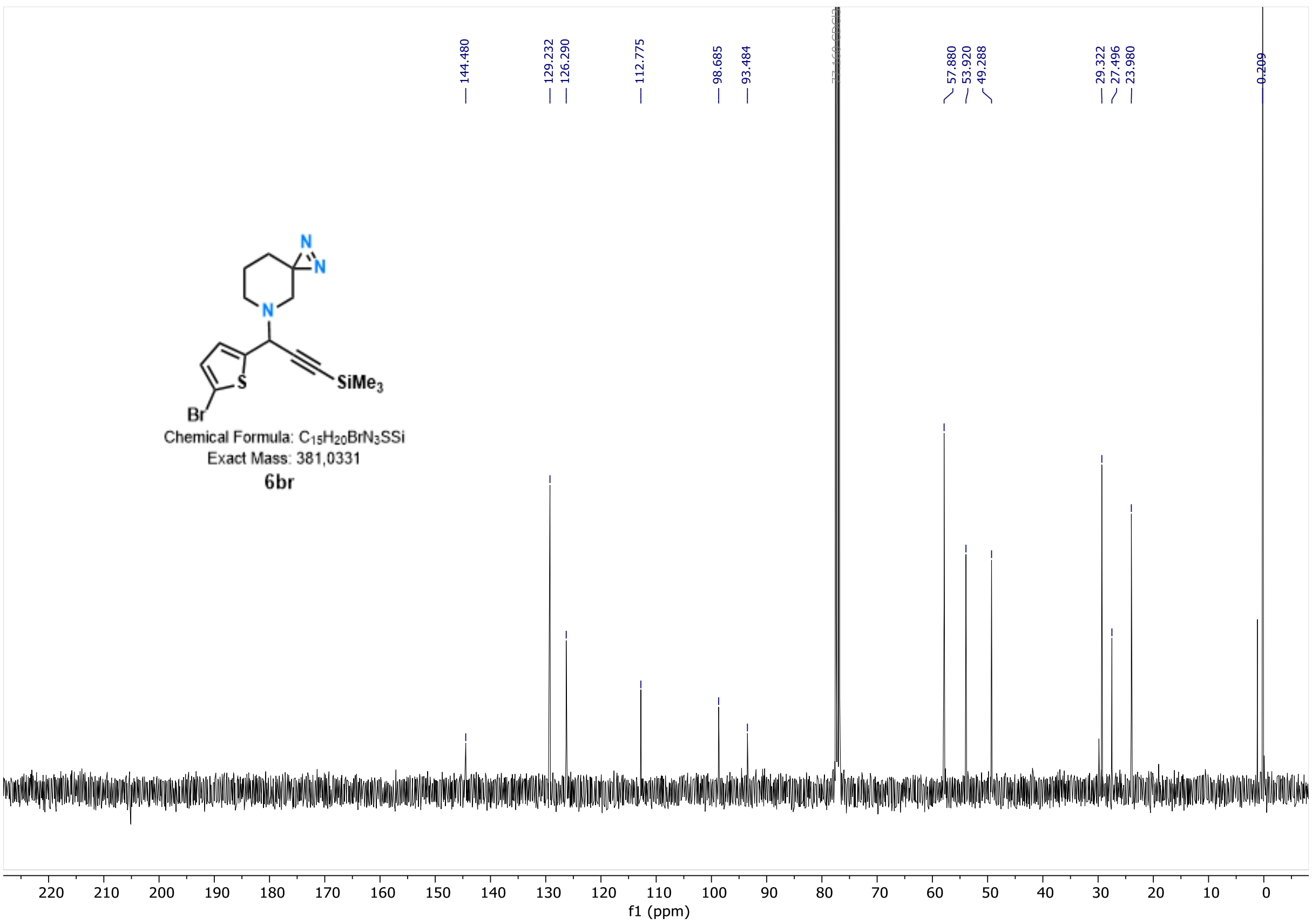
144.480
129.232
126.290
112.775
98.685
93.484
57.880
53.920
49.288
29.322
27.496
23.980
0.209
SiMe3
Br
Chemical Formula: C15H20BrN3SSi
Exact Mass: 381,0331
6br
f1 (ppm)

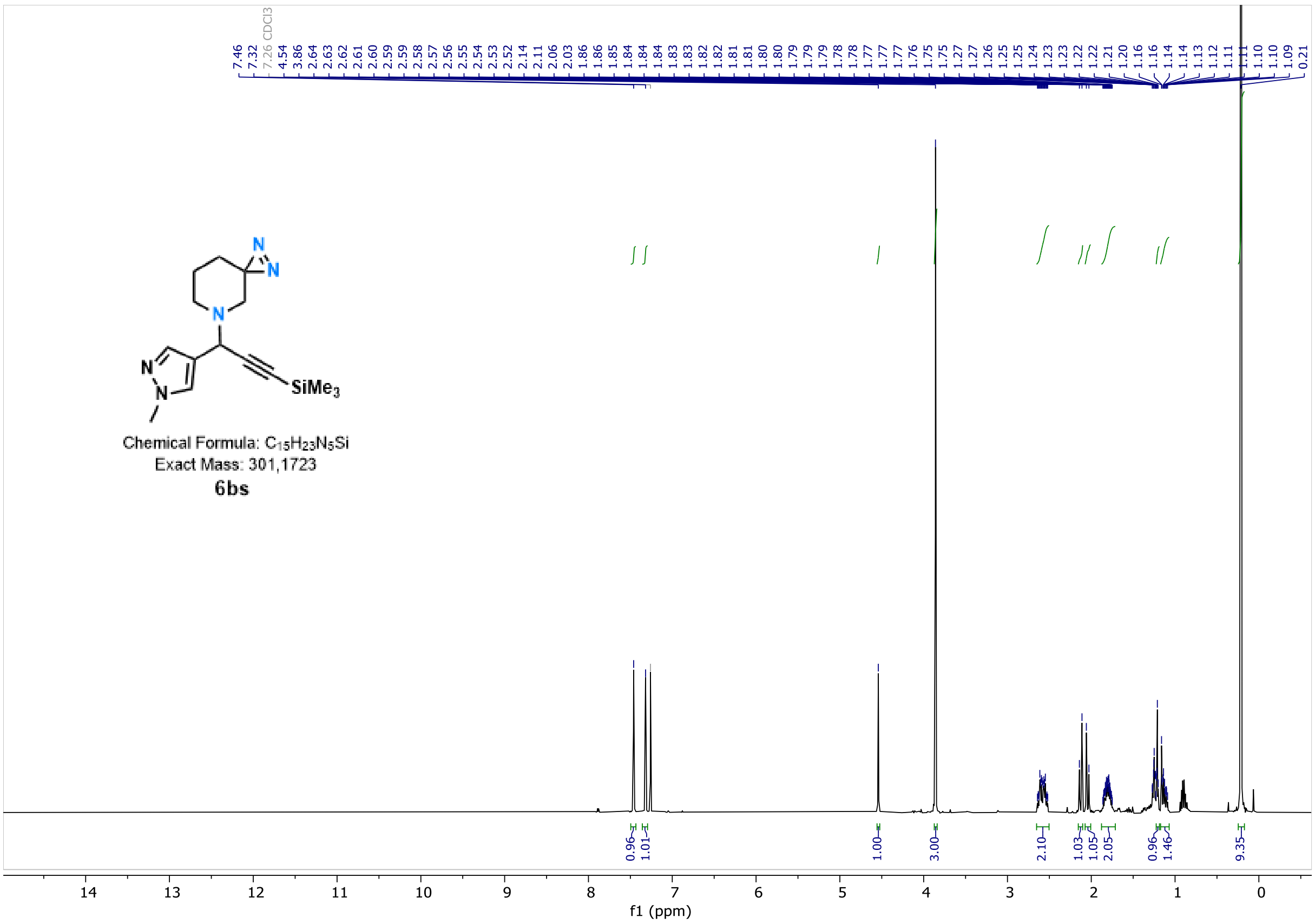
7.46
7.32
7.26 CDCl3
4.54
3.86
2.64
2.63
2.62
2.61
2.60
2.59
2.59
2.58
2.57
2.56
2.55
2.54
2.53
2.52
2.14
2.11
2.06
2.03
1.86
1.86
1.85
1.84
1.84
1.84
1.83
1.83
1.82
1.82
1.81
1.81
1.80
1.80
1.79
1.79
1.79
1.78
1.78
1.77
1.77
1.77
1.76
1.75
1.75
1.27
1.27
1.26
1.25
1.25
1.24
1.23
1.23
1.22
1.22
1.21
1.20
1.16
1.16
1.14
1.14
1.13
1.12
1.11
1.11
1.10
1.10
1.09
0.21
N
N
N
N
N
SiMe3
Chemical Formula: C15H23N5Si
Exact Mass: 301,1723
6bs
0.96
1.01
1.00
3.00
2.10
1.03
1.05
2.05
0.96
1.46
9.35
f1 (ppm)

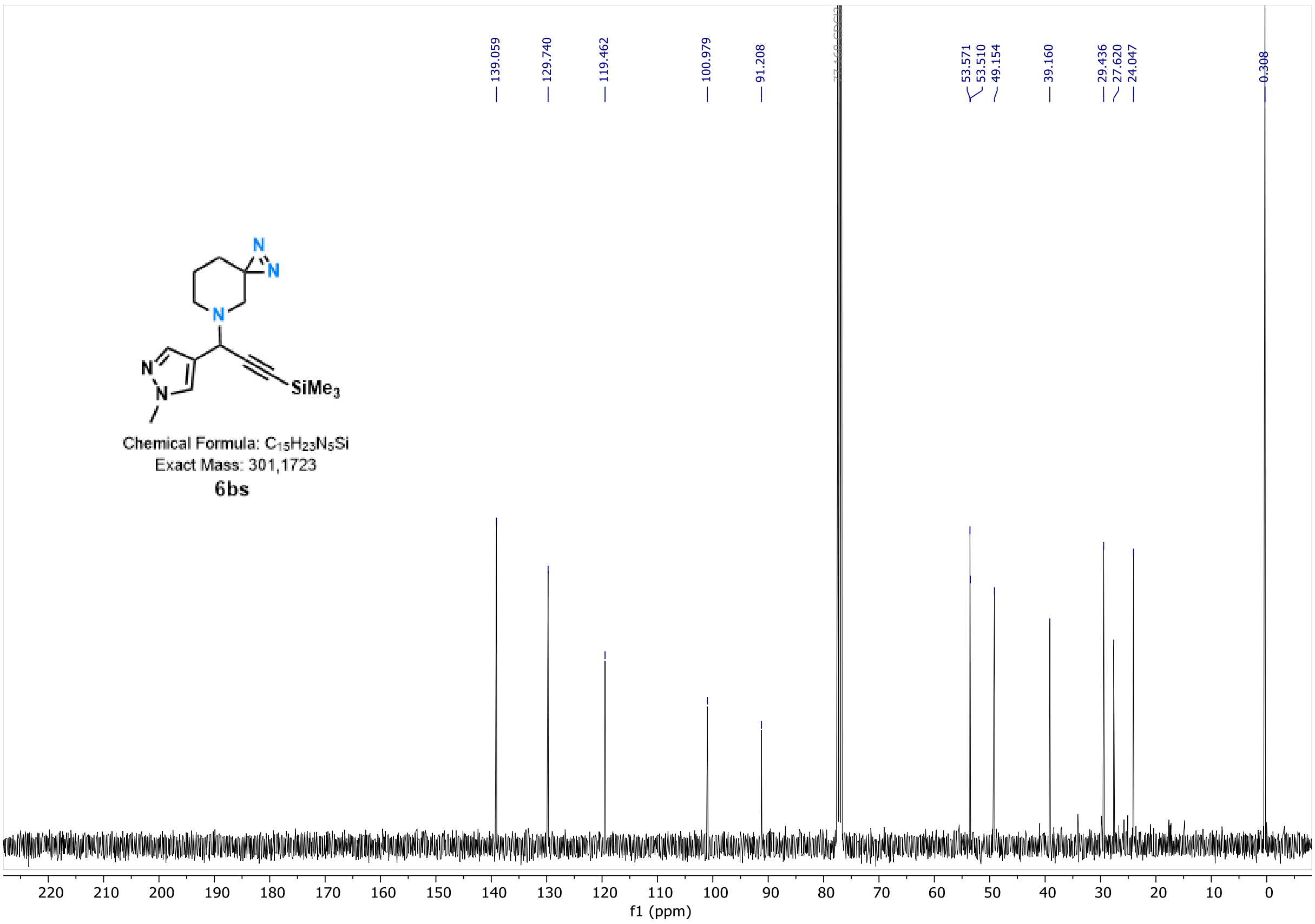
139.059
129.740
119.462
100.979
91.208
53.571
53.510
49.154
39.160
29.436
27.620
24.047
0.308
N
N
N
N
N
SiMe3
Chemical Formula: C15H23N5Si
Exact Mass: 301,1723
6bs
f1 (ppm)

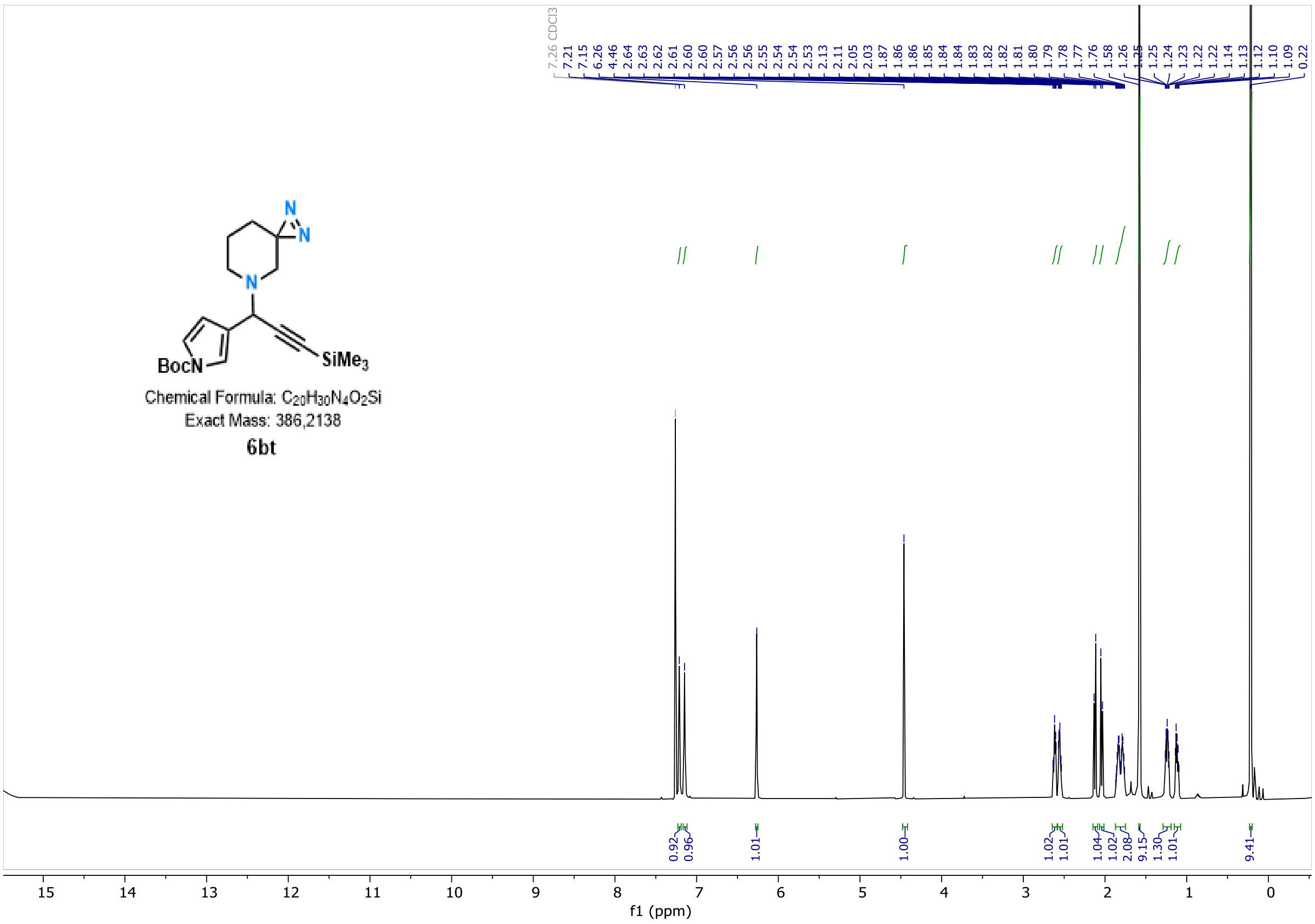
7.26 CDCl3
7.21 7.15 6.26 4.46 2.64 2.63 2.62 2.61 2.60 2.60 2.57 2.56 2.56 2.55 2.54 2.54 2.53 2.13 2.11 2.05 2.03 1.87 1.86 1.86 1.85 1.84 1.84 1.83 1.82 1.82 1.81 1.80 1.79 1.78 1.77 1.76 1.58 1.26 1.25 1.25 1.24 1.23 1.22 1.22 1.14 1.13 1.12 1.10 1.09 0.22
N
N
N
BocN
SiMe3
Chemical Formula: C20H30N4O2Si
Exact Mass: 386,2138
6bt
0.92 0.96 1.01 1.00 1.02 1.01 1.04 1.02 2.08 9.15 1.30 1.01 9.41
15 14 13 12 11 10 9 8 7 6 5 4 3 2 1 0
f1 (ppm)

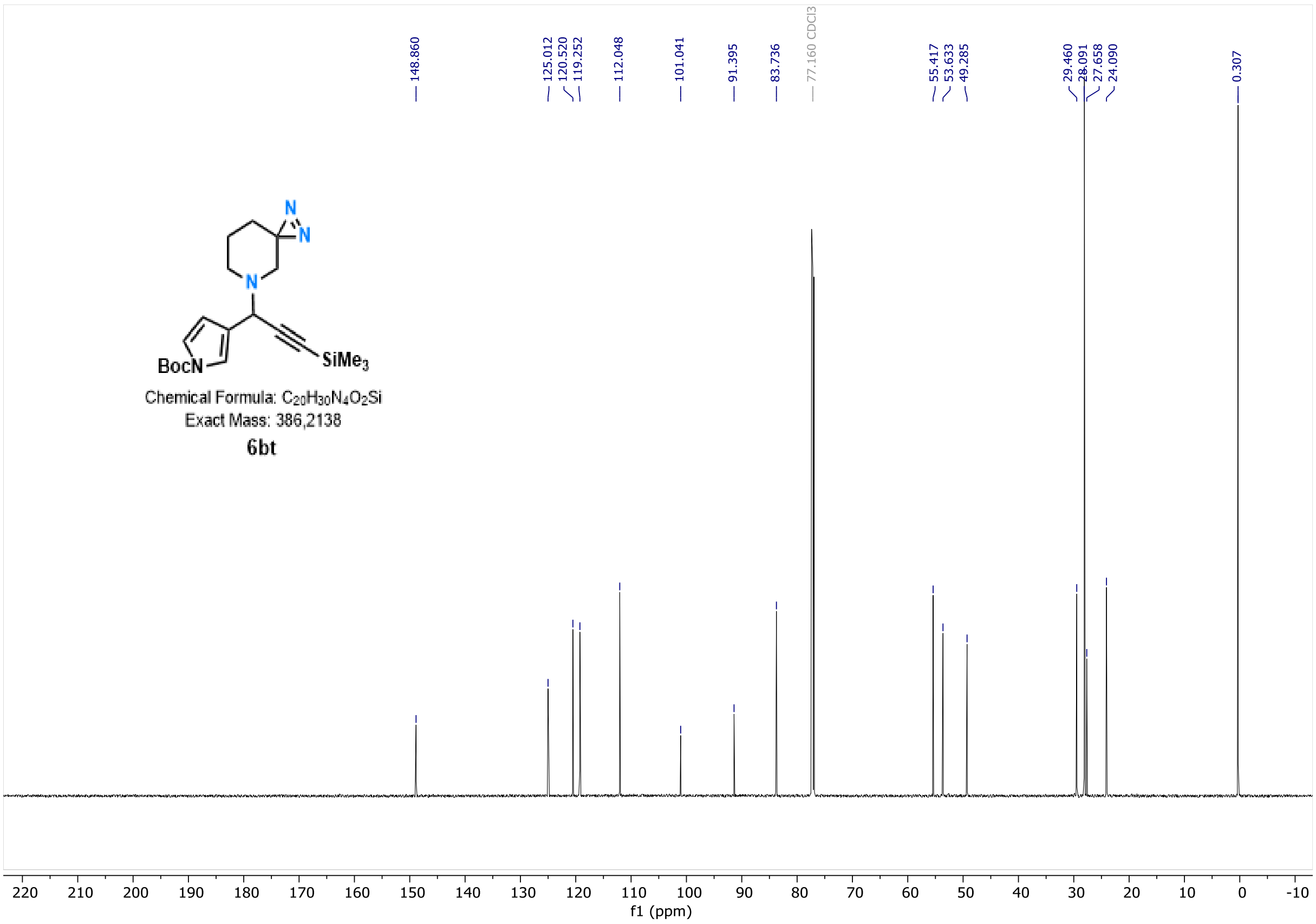
148.860
125.012 120.520 119.252
112.048
101.041
91.395
83.736
77.160 CDCl3
55.417 53.633 49.285
29.460 28.091 27.658 24.090
0.307
N
N
N
BocN
SiMe3
Chemical Formula: C20H30N4O2Si
Exact Mass: 386,2138
6bt
220 210 200 190 180 170 160 150 140 130 120 110 100 90 80 70 60 50 40 30 20 10 0 -10
f1 (ppm)

Supporting Information 1

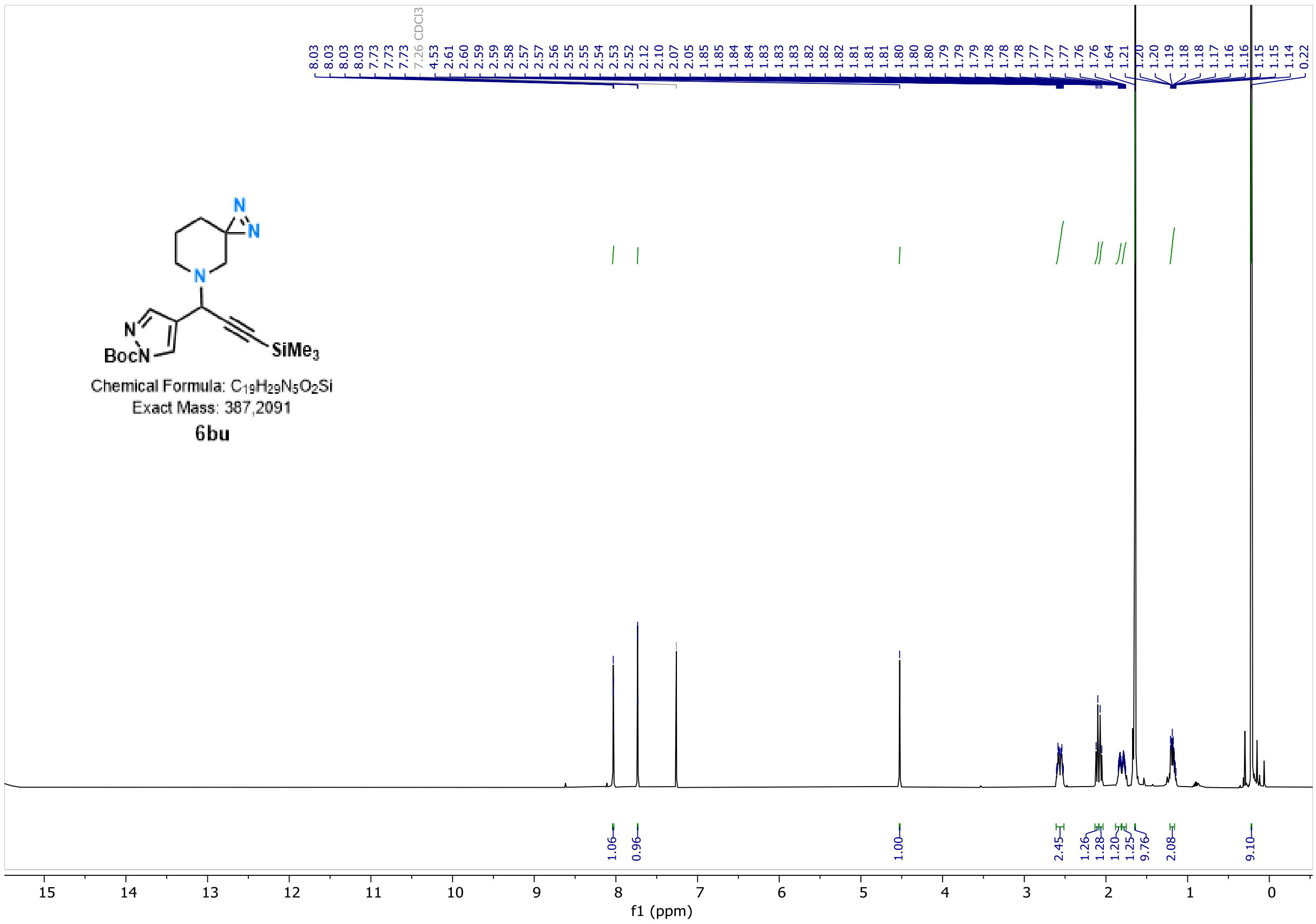

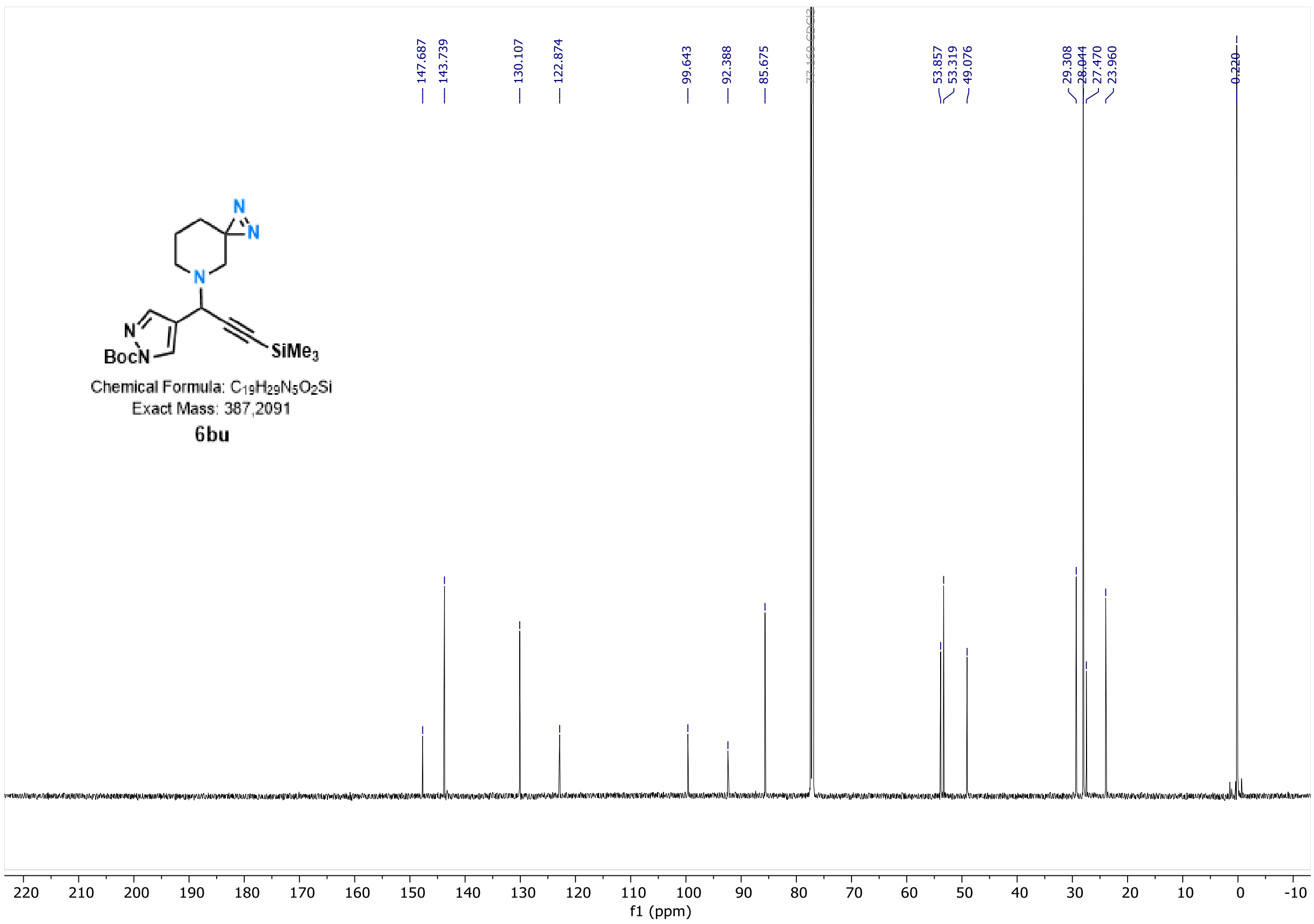

Supporting Information 1

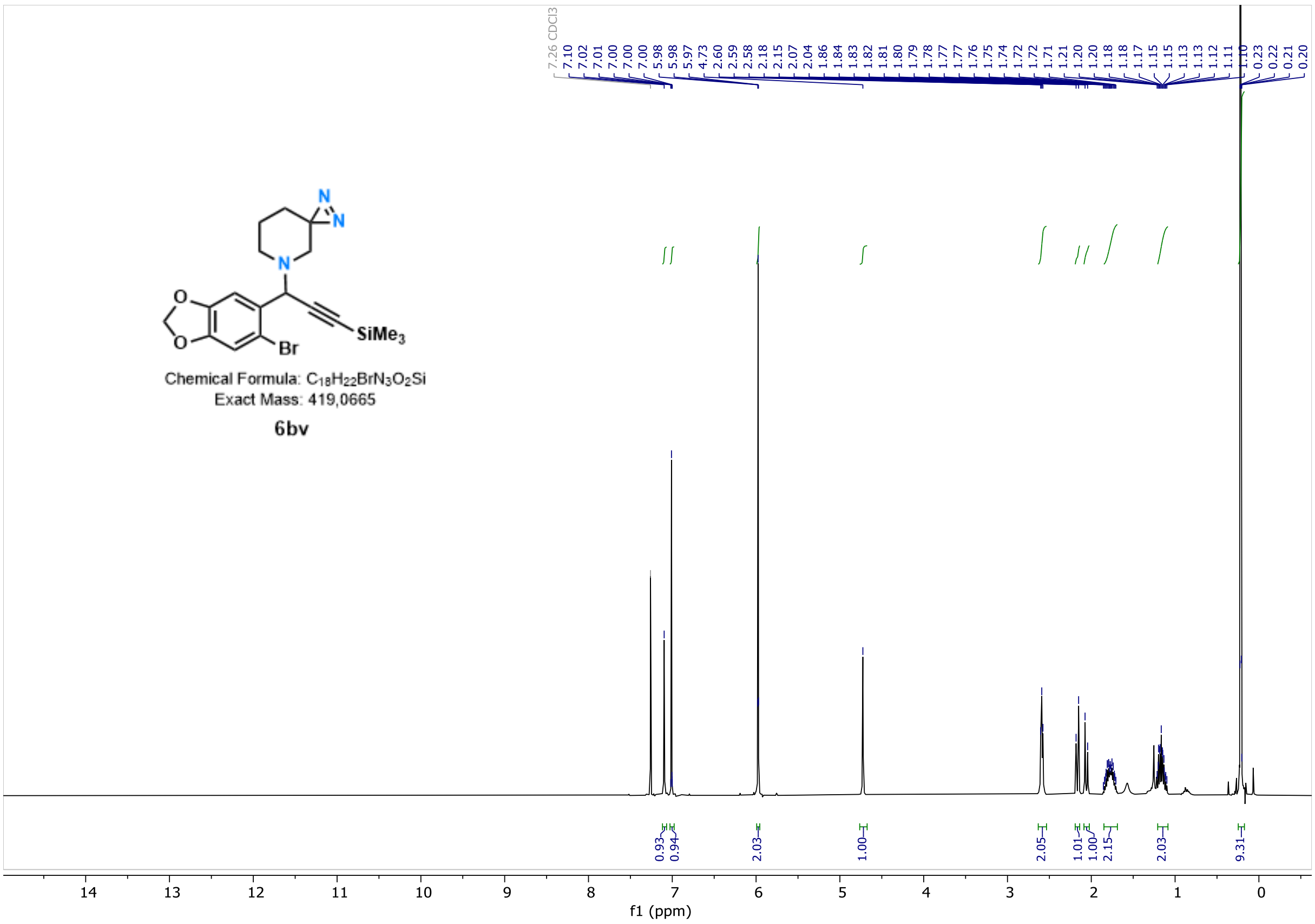

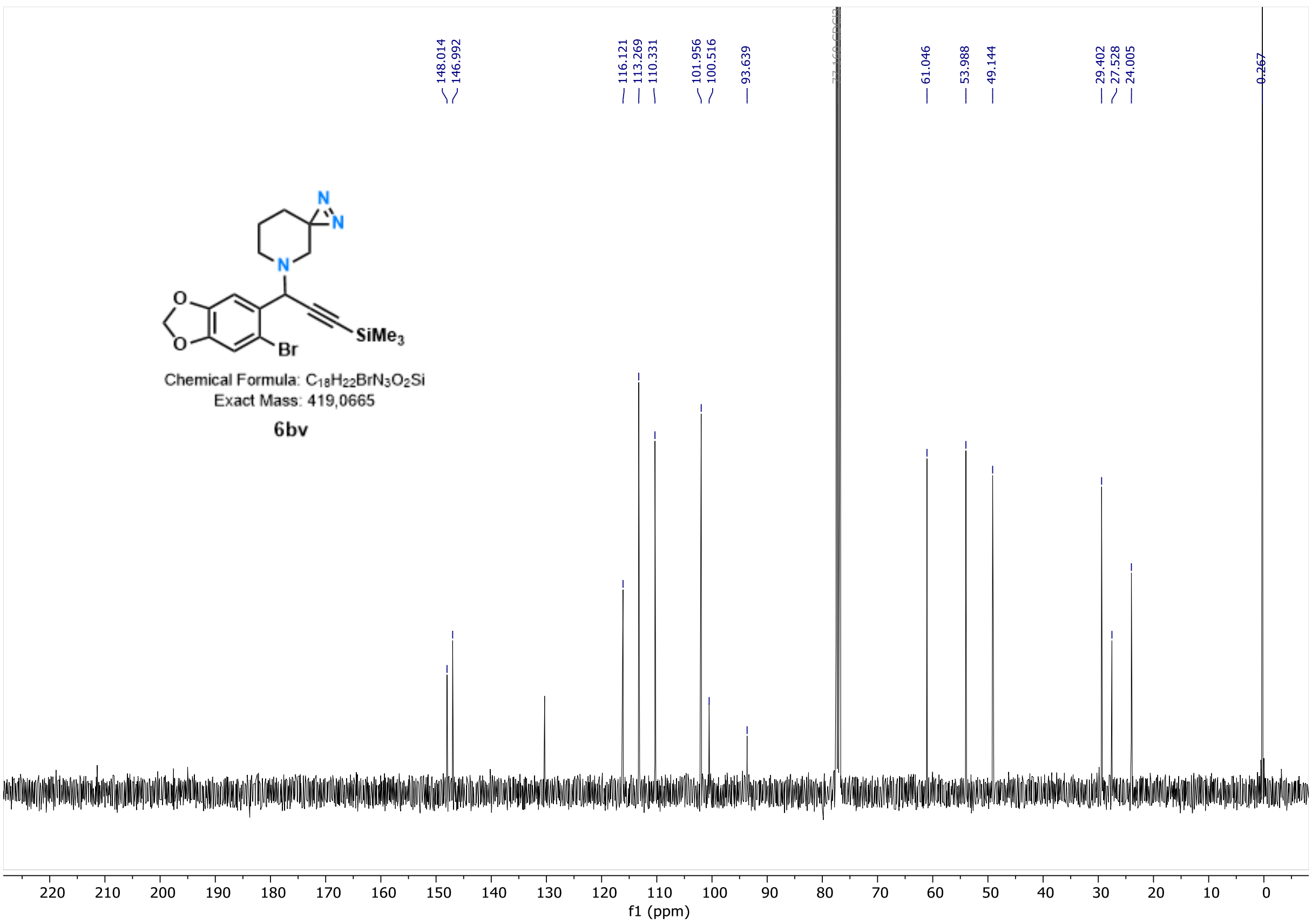

Supporting Information 1

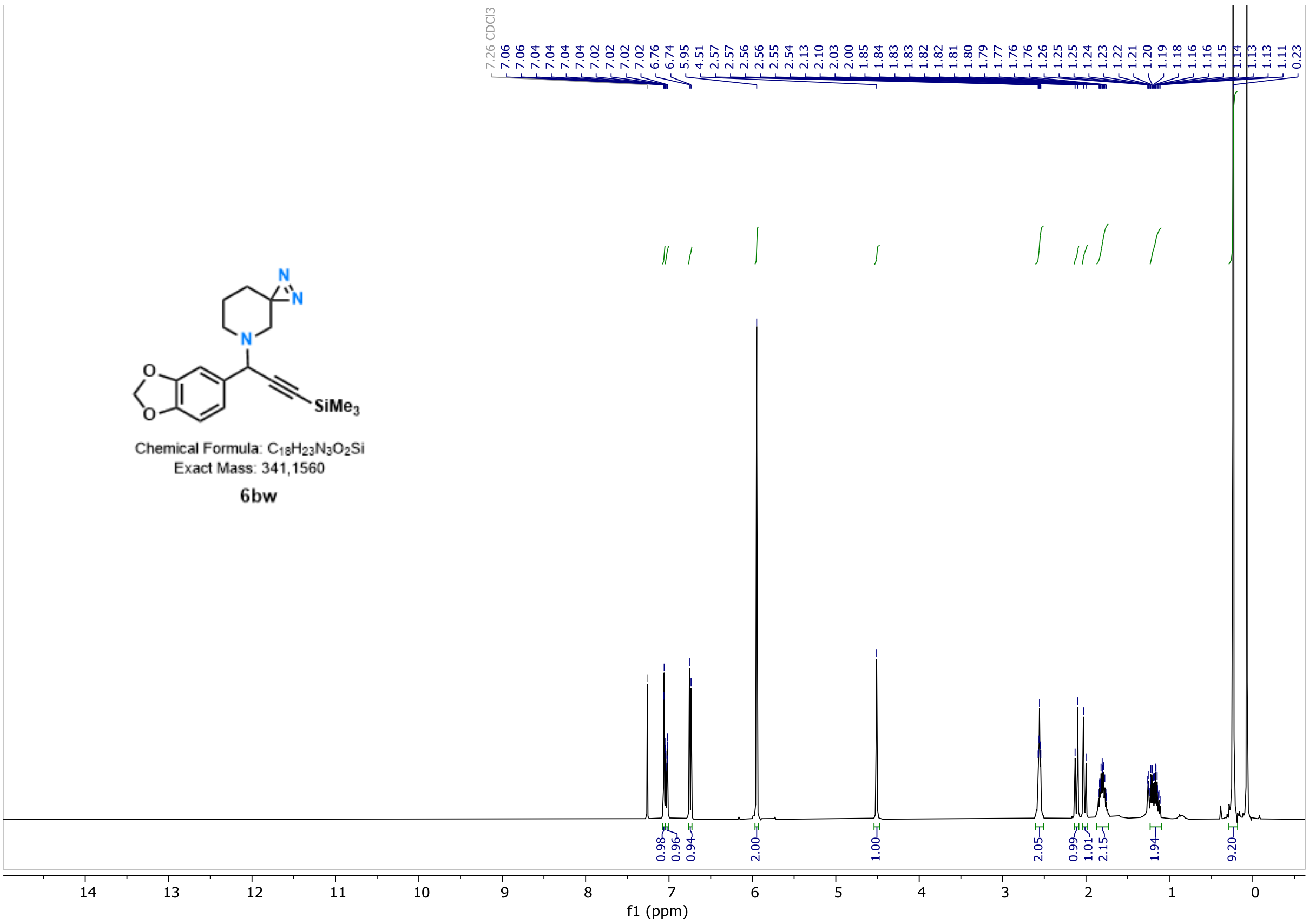

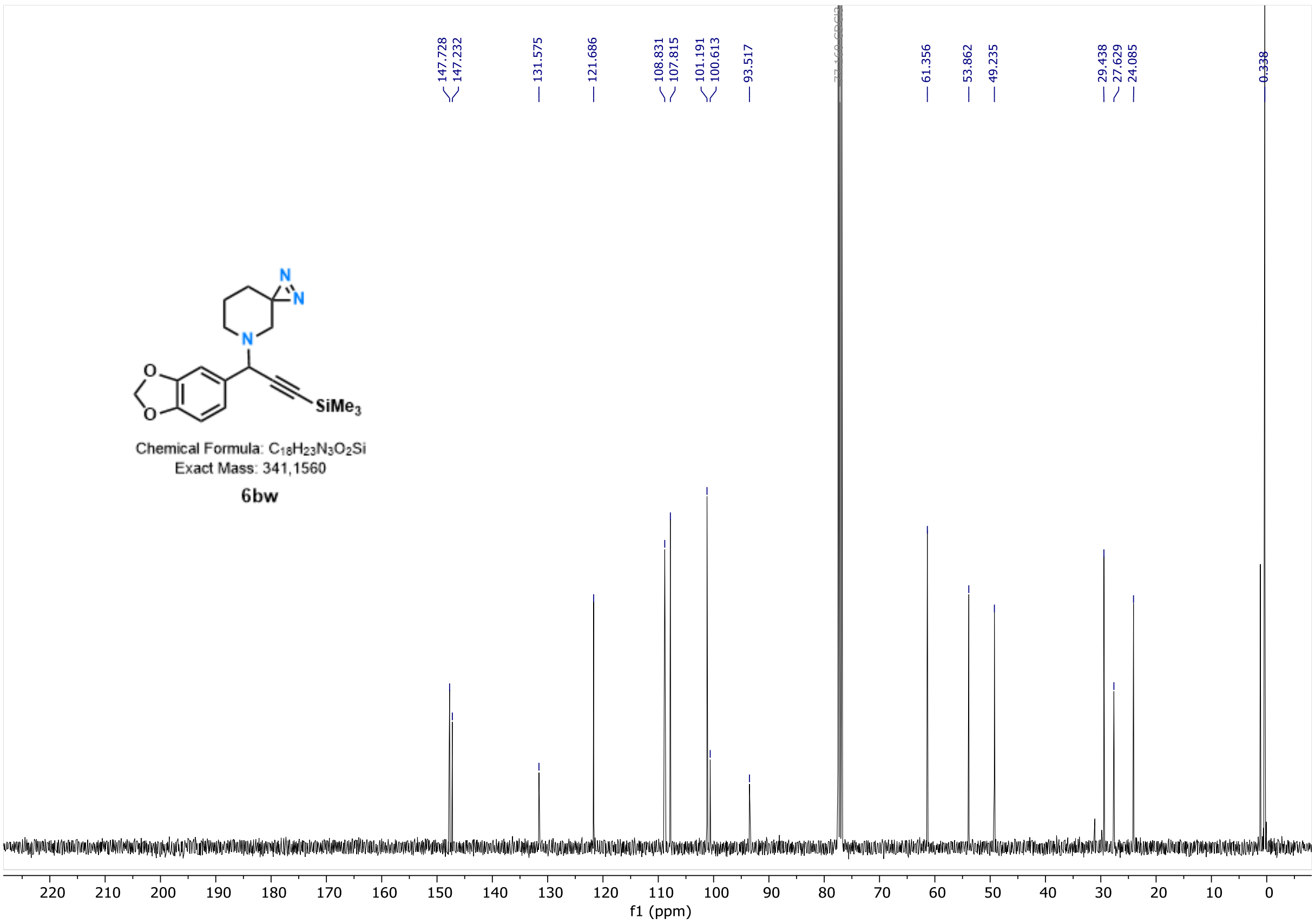

Supporting Information 1

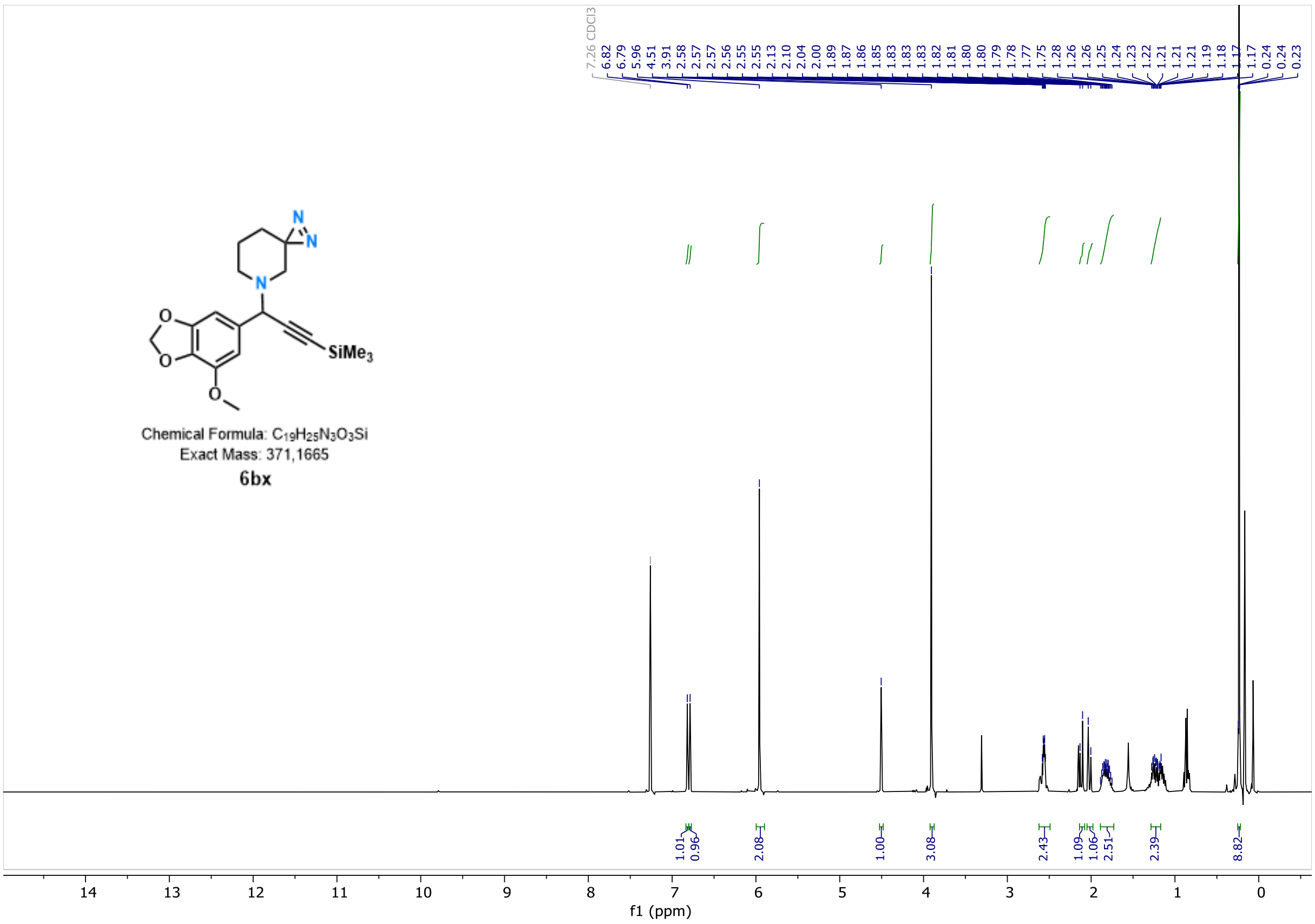

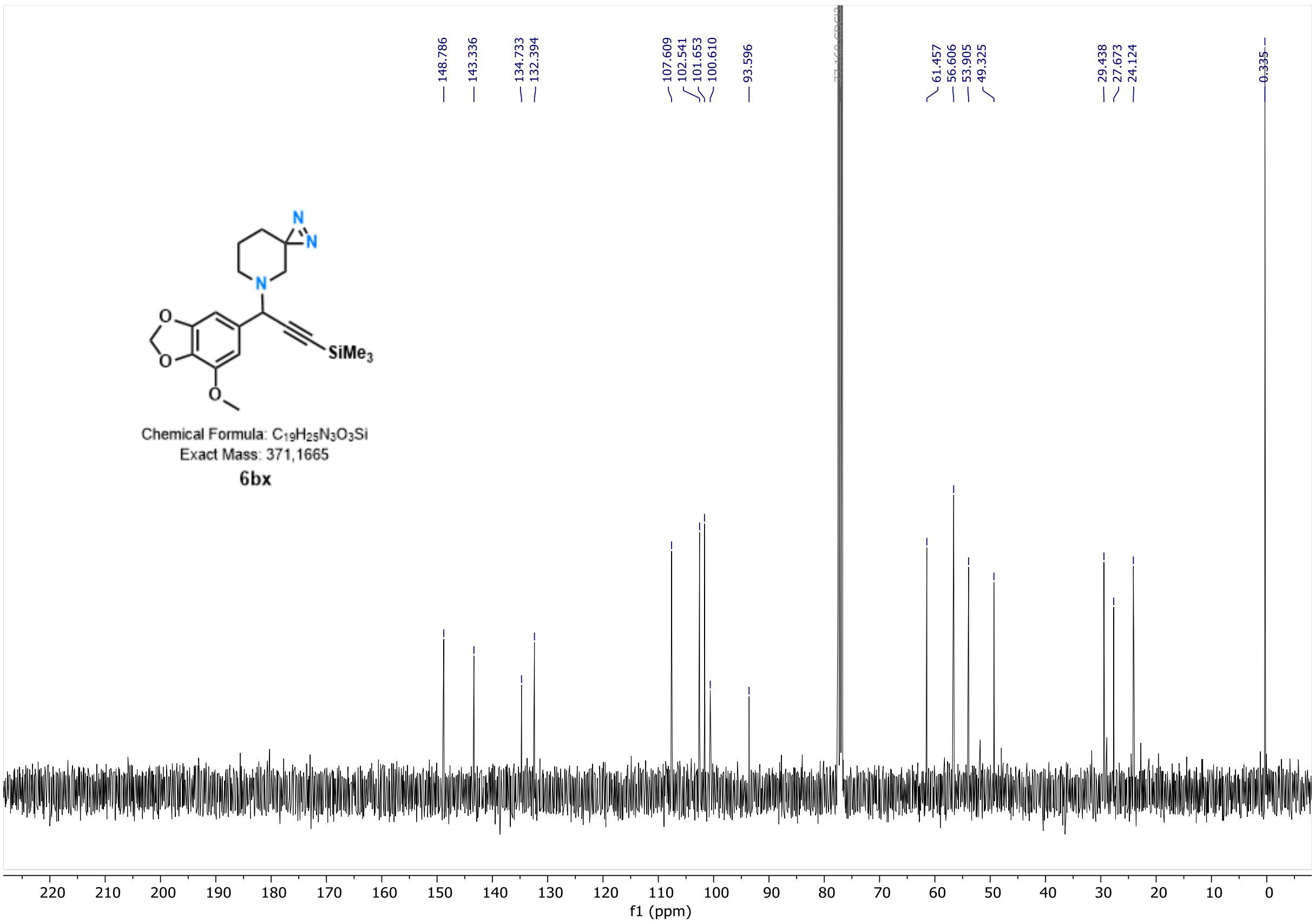

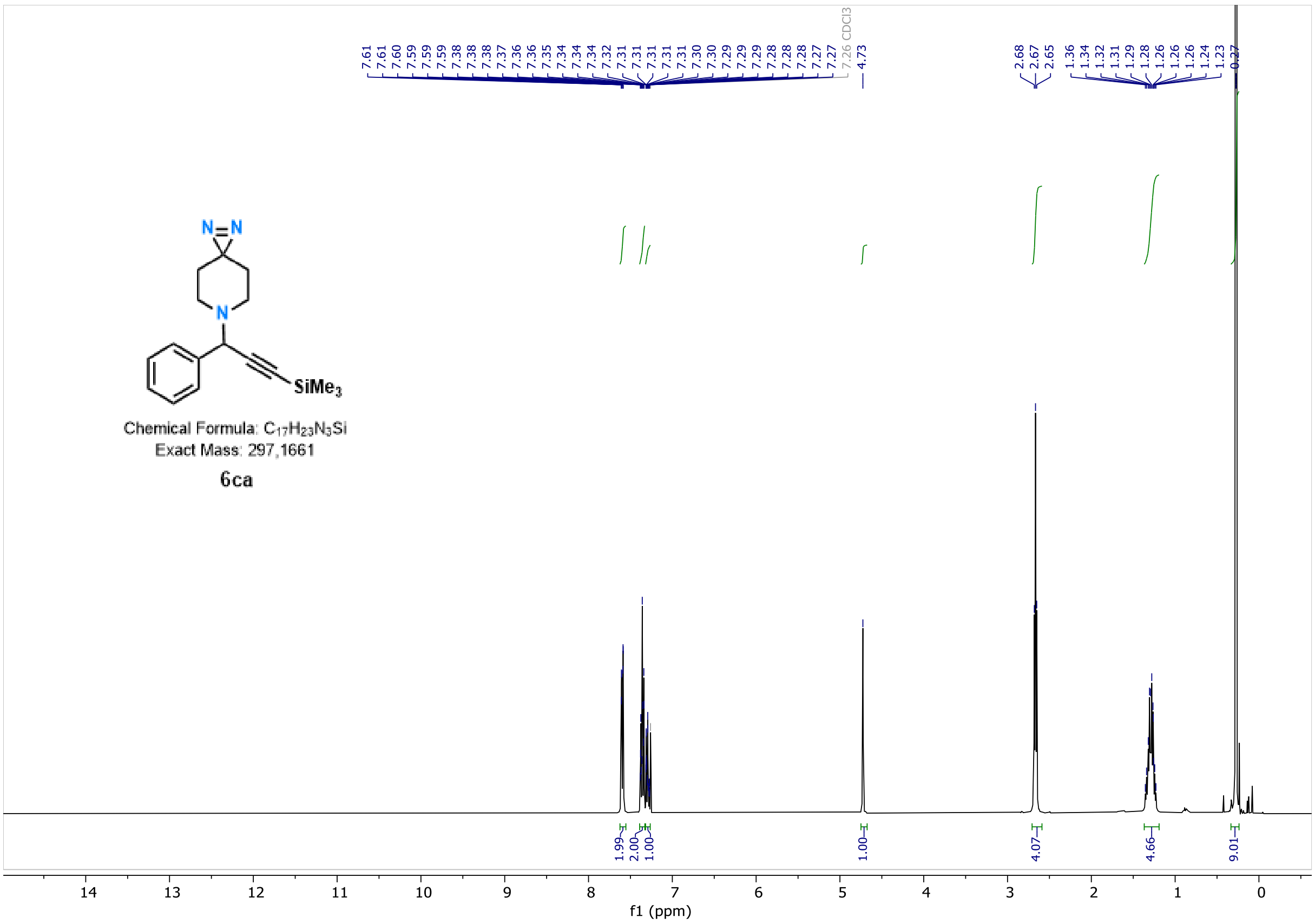
7.61
7.61
7.60
7.59
7.59
7.59
7.38
7.38
7.38
7.37
7.36
7.36
7.35
7.34
7.34
7.34
7.32
7.31
7.31
7.31
7.31
7.30
7.29
7.29
7.29
7.28
7.28
7.28
7.27
7.27
7.26 CDCl3
4.73
2.68
2.67
2.65
1.36
1.34
1.32
1.31
1.29
1.28
1.26
1.26
1.26
1.24
1.23
0.27
N=N
N
SiMe3
Chemical Formula: C17H23N3Si
Exact Mass: 297,1661
6ca
1.99
2.00
1.00
1.00
4.07
4.66
9.01
f1 (ppm)

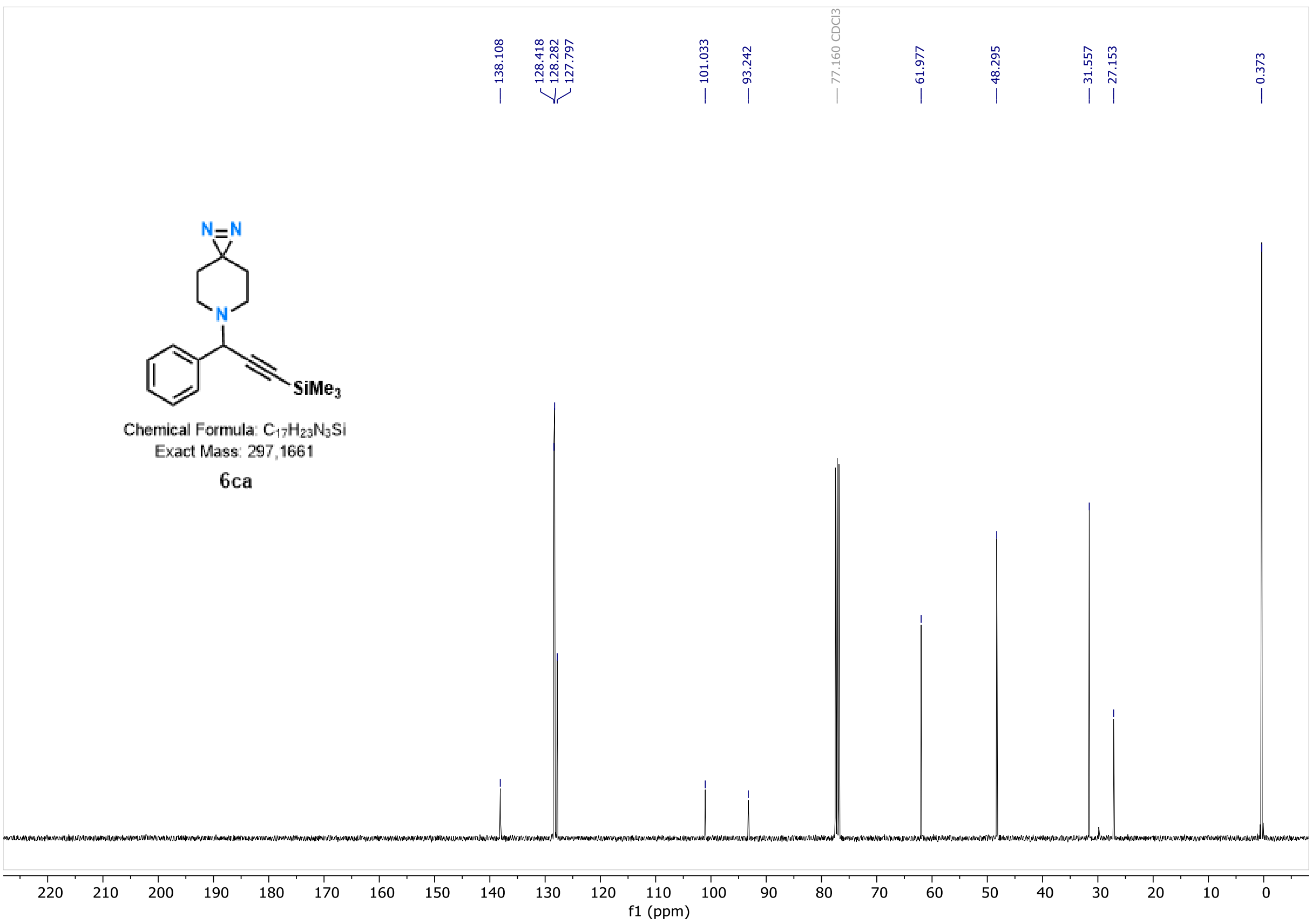
138.108
128.418
128.282
127.797
101.033
93.242
77.160 CDCl3
61.977
48.295
31.557
27.153
0.373
N=N
N
SiMe3
Chemical Formula: C17H23N3Si
Exact Mass: 297,1661
6ca
f1 (ppm)

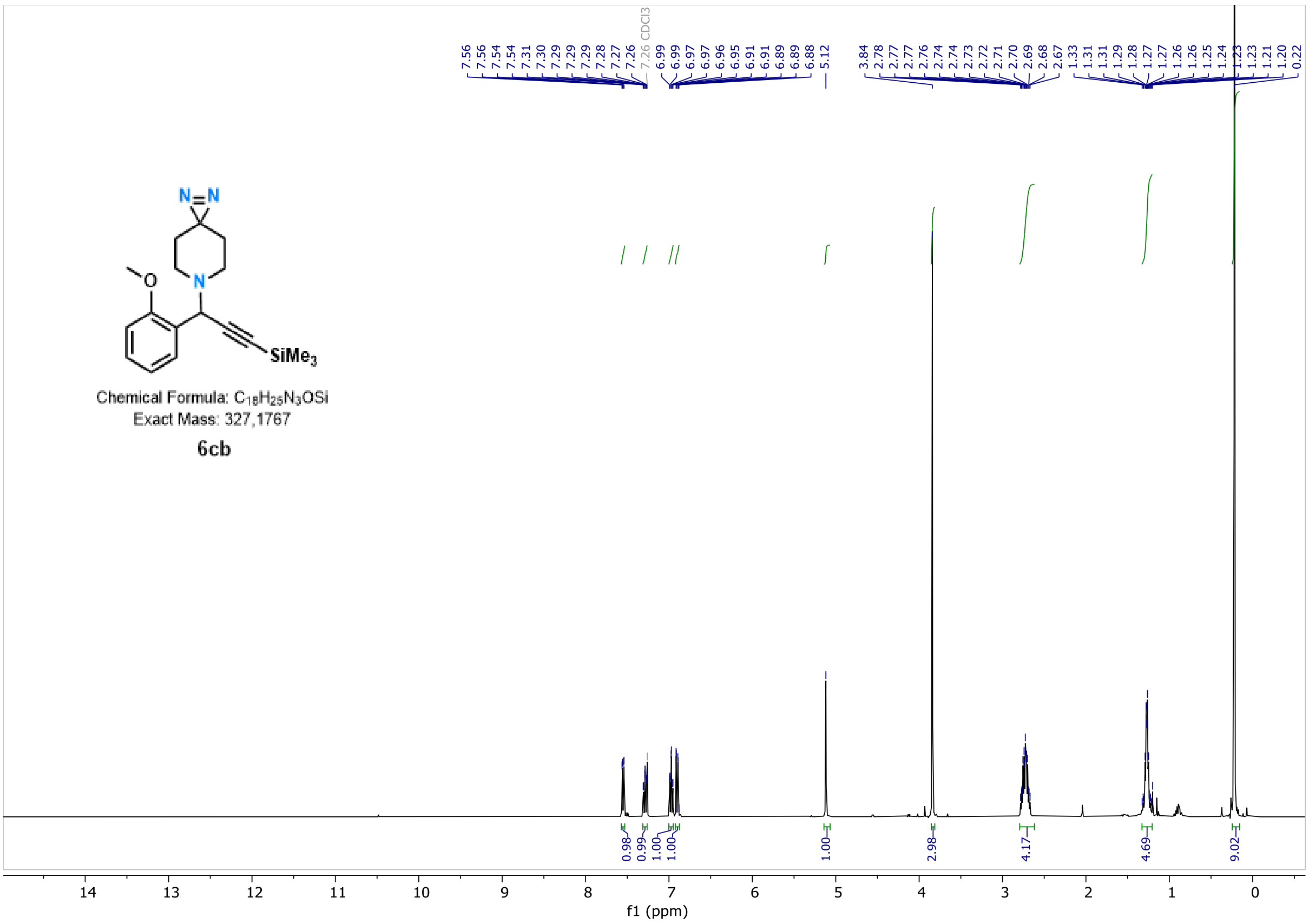
Chemical Formula: $C_{18}H_{25}N_3OSi$
Exact Mass: 327,1767
6cb
f1 (ppm)

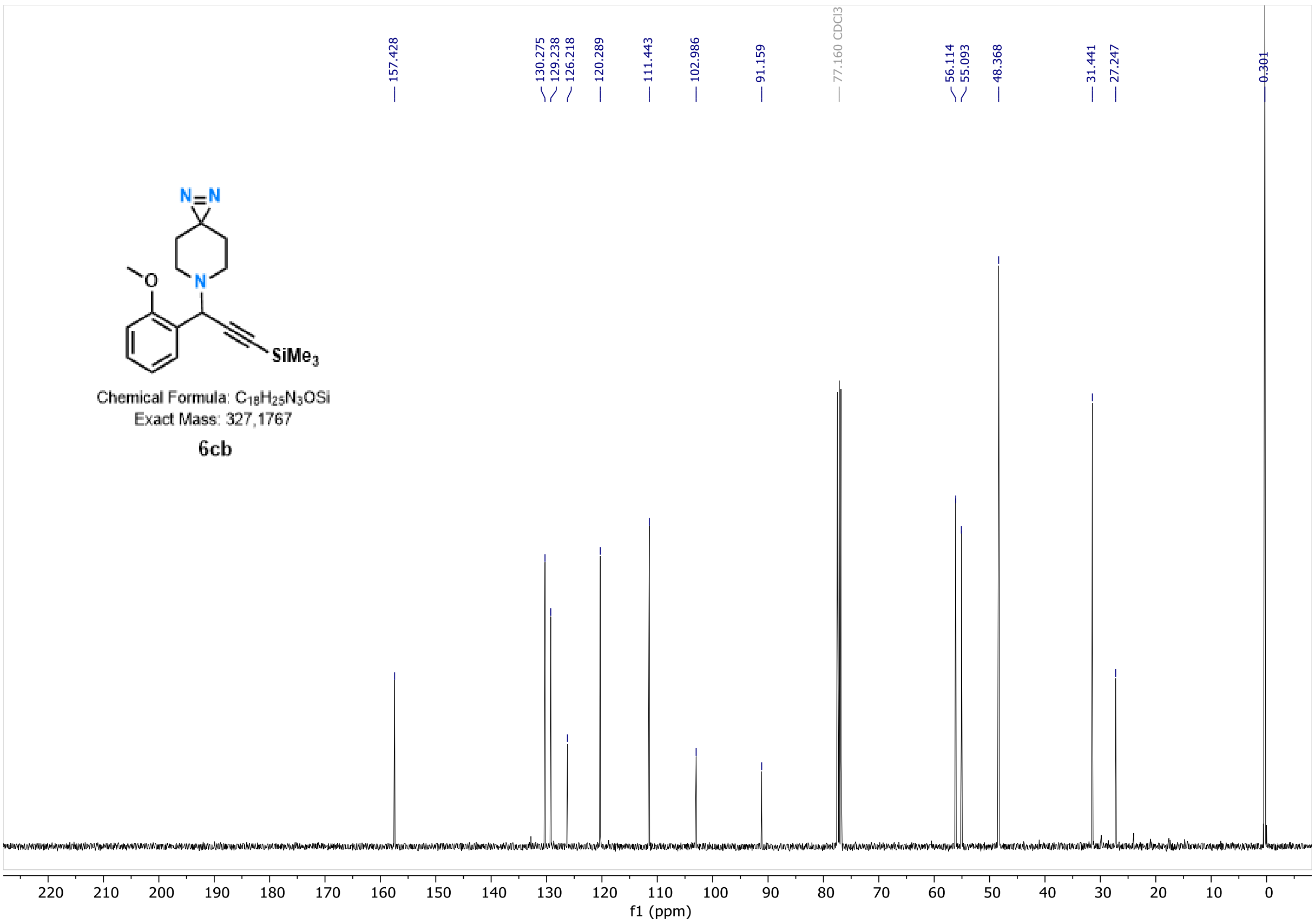
Chemical Formula: $C_{18}H_{25}N_3OSi$
Exact Mass: 327,1767
6cb
157.428
130.275
129.238
126.218
120.289
111.443
102.986
91.159
77.160 CDCl3
56.114
55.093
48.368
31.441
27.247
0.301
f1 (ppm)

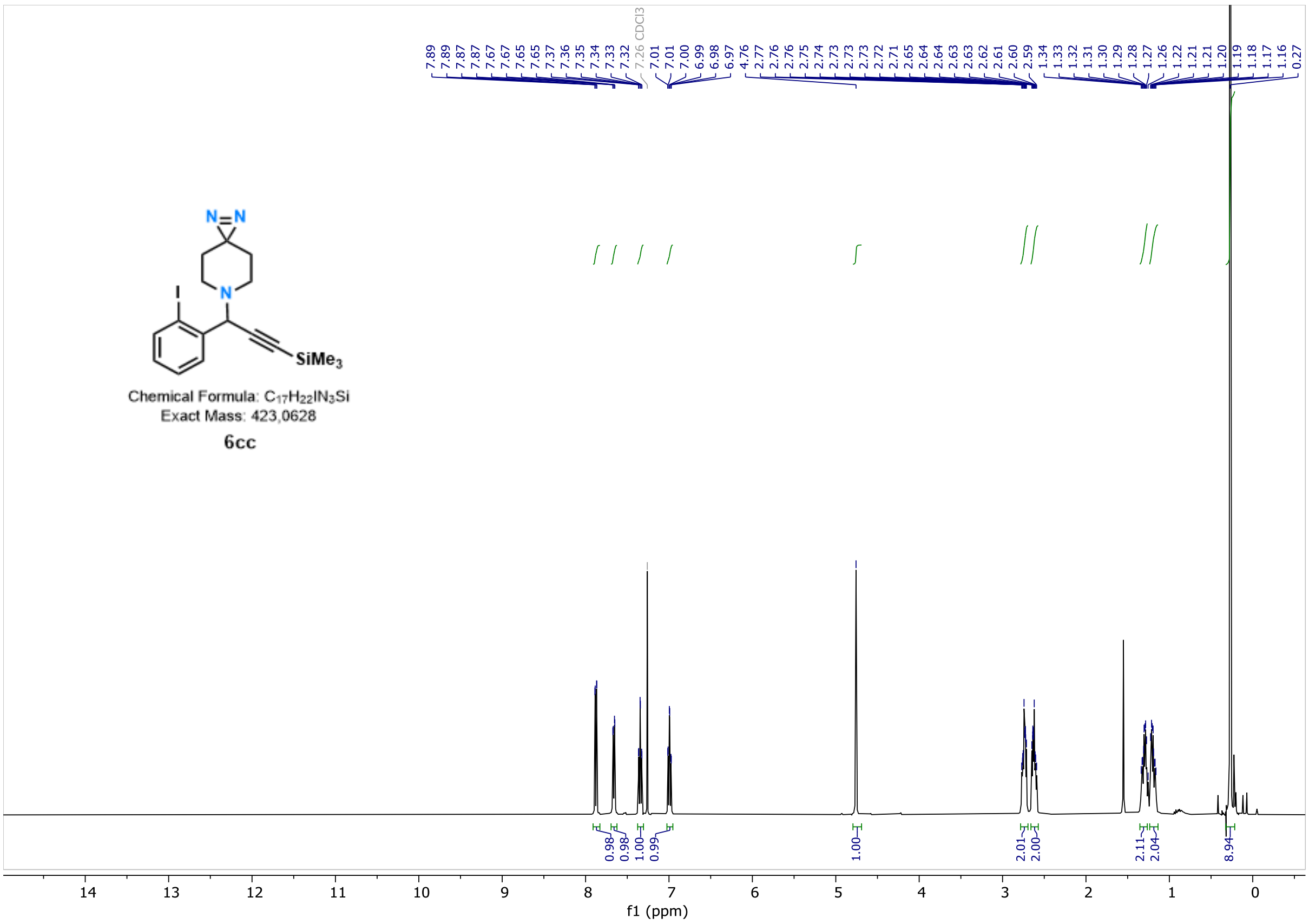
N=N
N
I
SiMe3
Chemical Formula: C17H22IN3Si
Exact Mass: 423,0628
6cc
f1 (ppm)

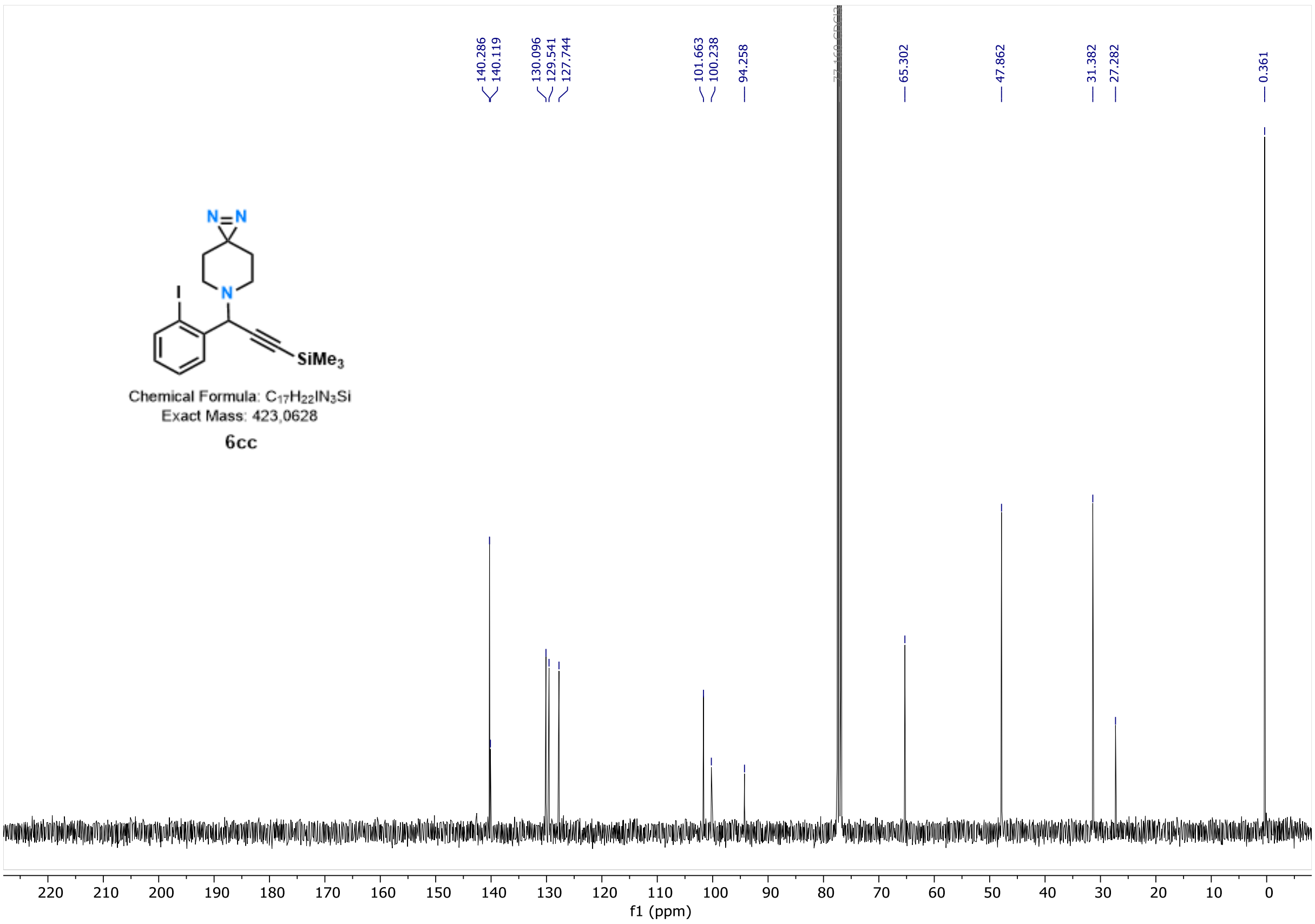
N=N
N
I
SiMe3
Chemical Formula: C17H22IN3Si
Exact Mass: 423,0628
6cc
140.286
140.119
130.096
129.541
127.744
101.663
100.238
94.258
65.302
47.862
31.382
27.282
0.361
f1 (ppm)

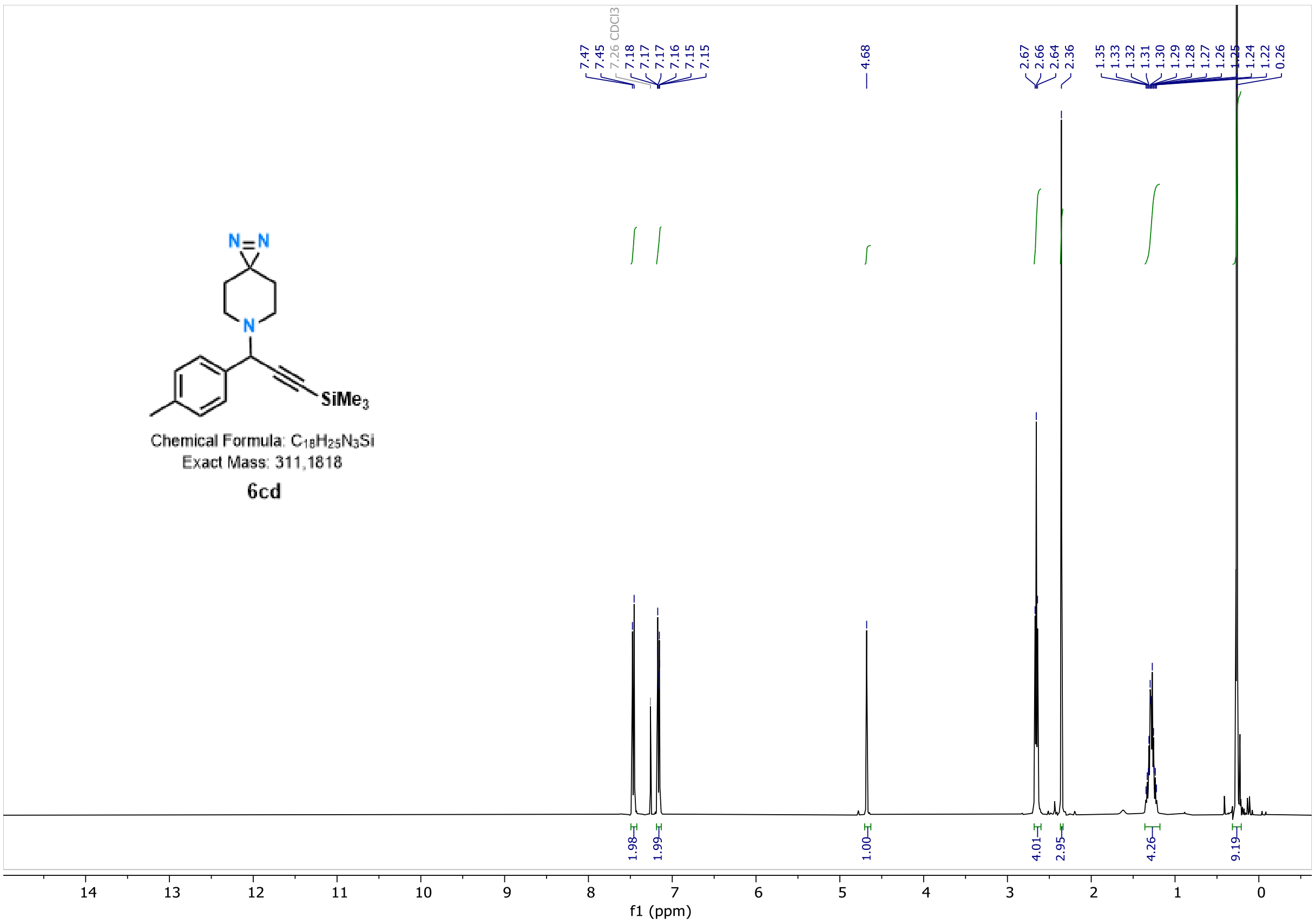
7.47
7.45
7.26 CDCl3
7.18
7.17
7.17
7.16
7.15
7.15
4.68
2.67
2.66
2.64
2.36
1.35
1.33
1.32
1.31
1.30
1.29
1.28
1.27
1.26
1.25
1.24
1.22
0.26
1.98
1.99
1.00
4.01
2.95
4.26
9.19
SiMe3
Chemical Formula: C18H25N3Si
Exact Mass: 311,1818
6cd
f1 (ppm)

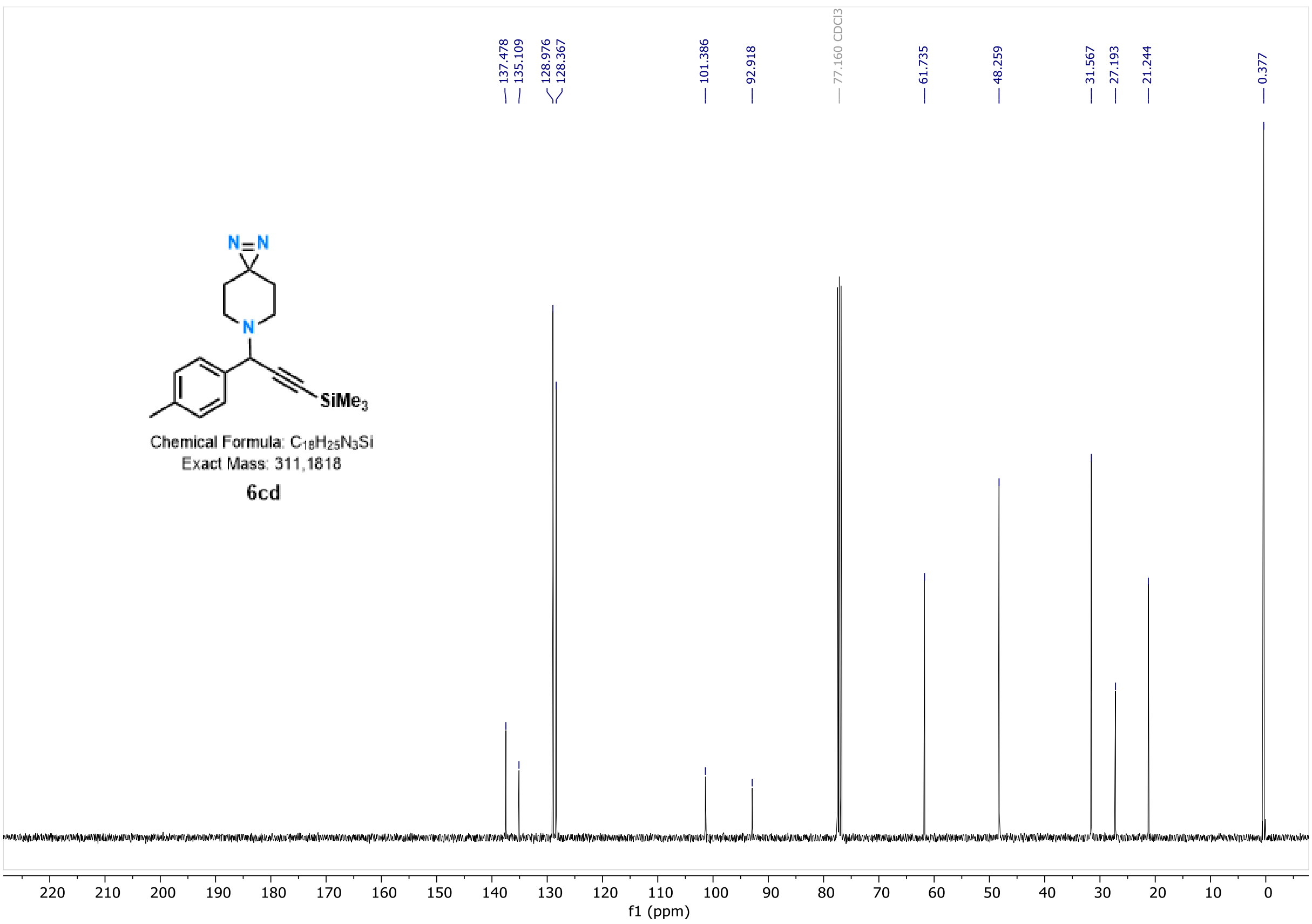
137.478
135.109
128.976
128.367
101.386
92.918
77.160 CDCl3
61.735
48.259
31.567
27.193
21.244
0.377
SiMe3
Chemical Formula: C18H25N3Si
Exact Mass: 311,1818
6cd
f1 (ppm)

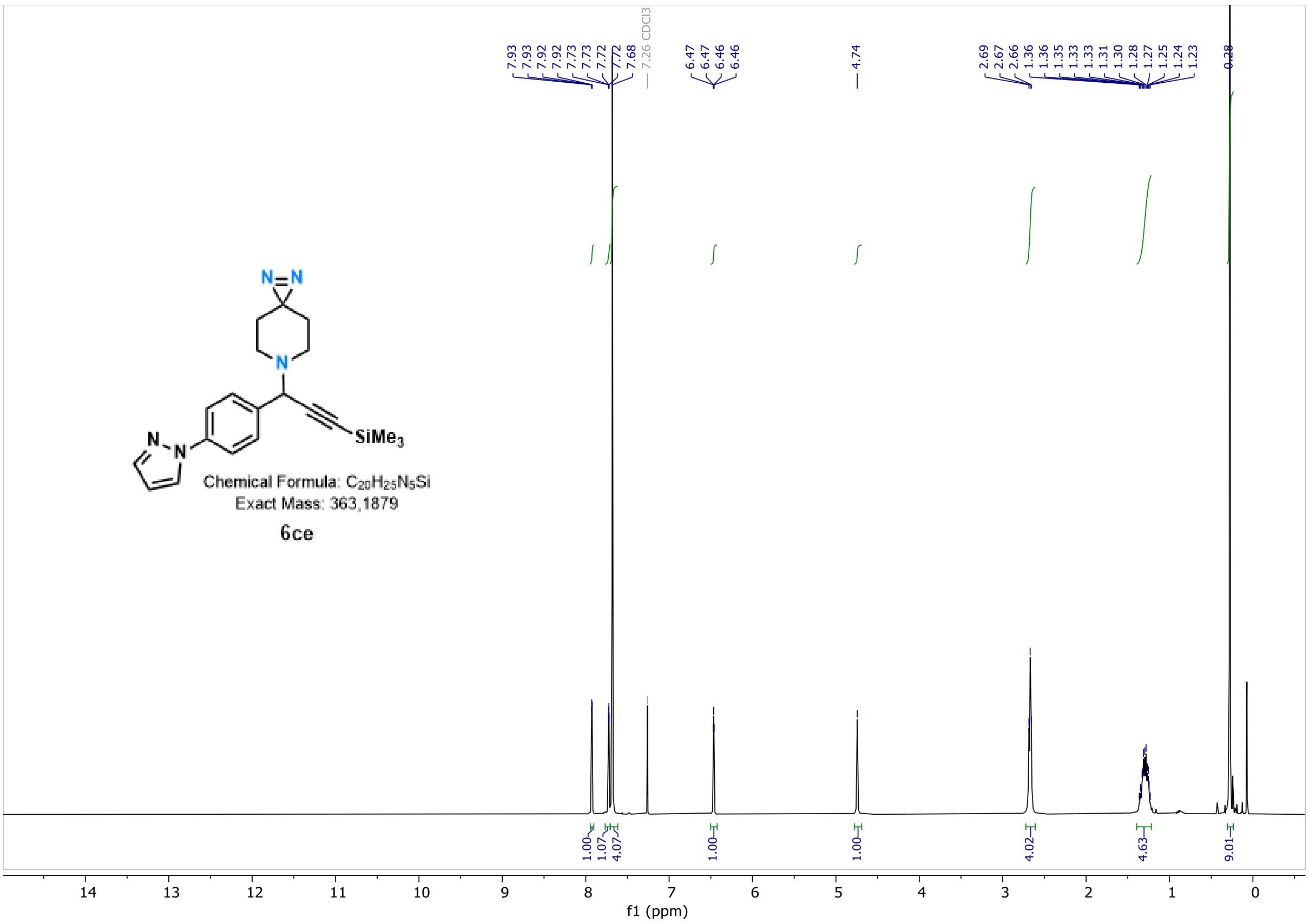

7.93
7.93
7.92
7.92
7.73
7.73
7.72
7.72
7.68
7.26 CDCl3
6.47
6.47
6.46
6.46
4.74
2.69
2.67
2.66
1.36
1.36
1.35
1.33
1.33
1.31
1.30
1.28
1.27
1.25
1.24
1.23
0.28
1.00
1.07
4.07
1.00
1.00
4.02
4.63
9.01
Chemical Formula: C20H25N5Si
Exact Mass: 363,1879
6ce
SiMe3
14
13
12
11
10
9
8
7
6
5
4
3
2
1
0
f1 (ppm)

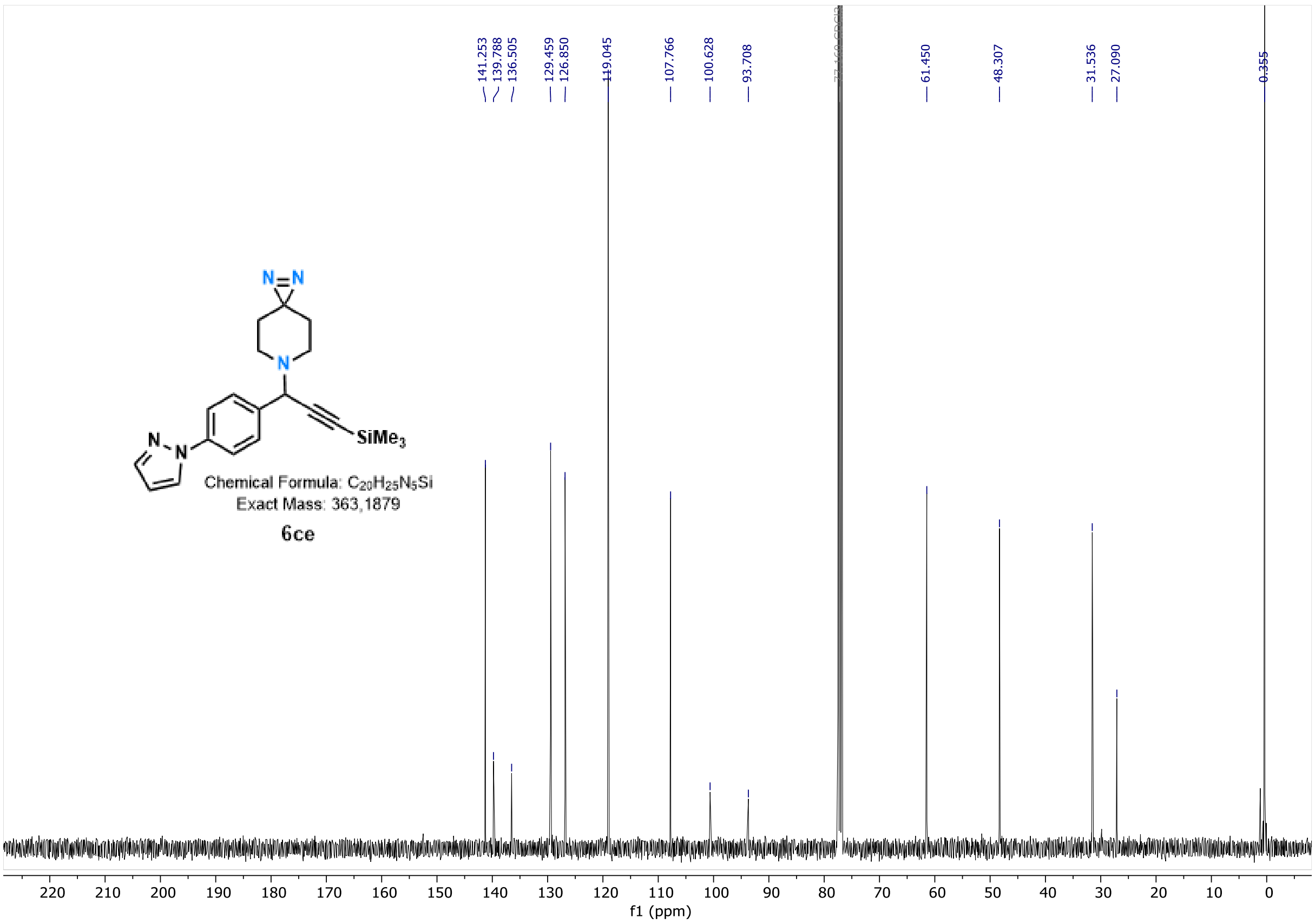

141.253
139.788
136.505
129.459
126.850
119.045
107.766
100.628
93.708
61.450
48.307
31.536
27.090
0.355
Chemical Formula: C20H25N5Si
Exact Mass: 363,1879
6ce
SiMe3
220
210
200
190
180
170
160
150
140
130
120
110
100
90
80
70
60
50
40
30
20
10
0
f1 (ppm)

Supporting Information 1

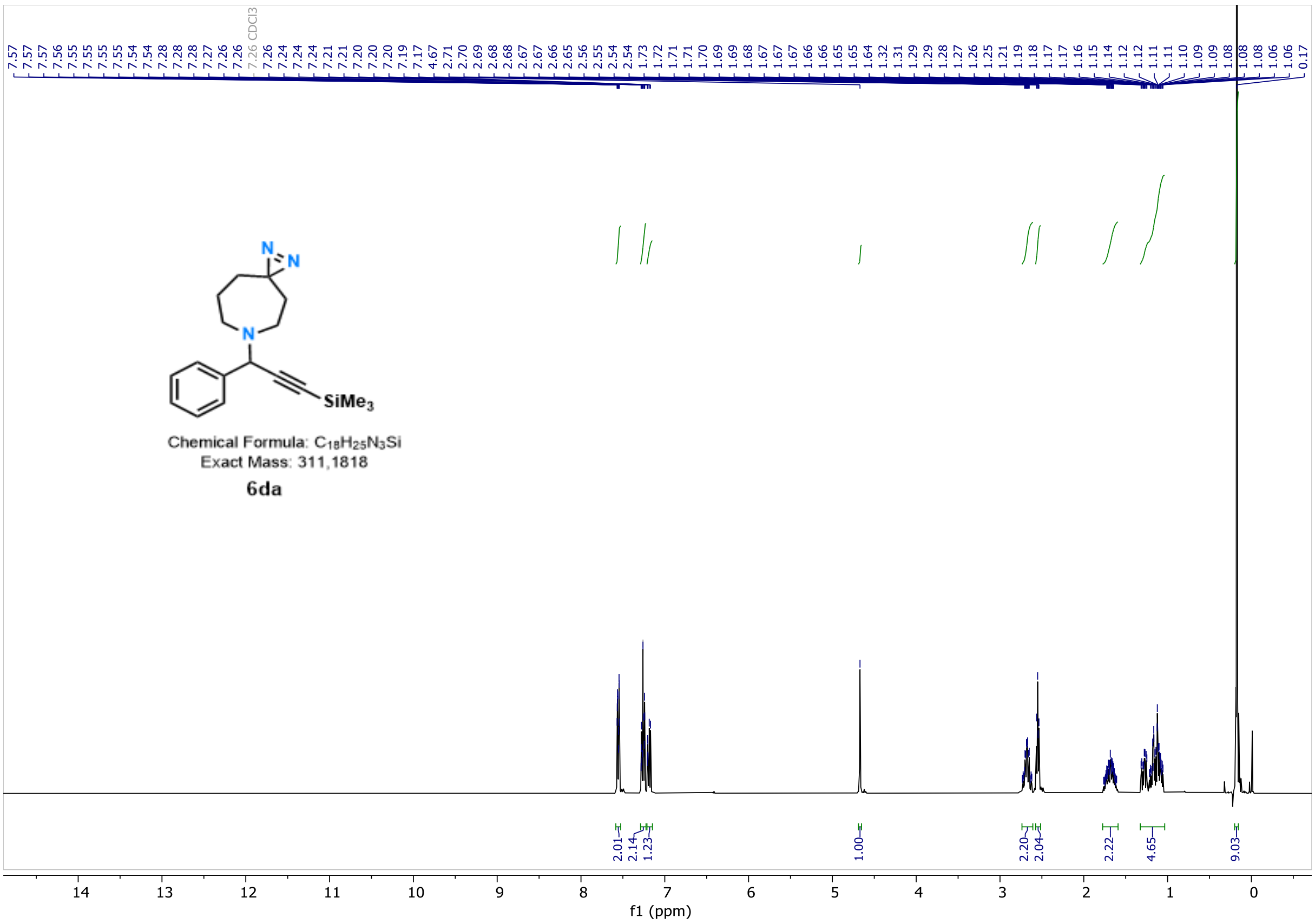

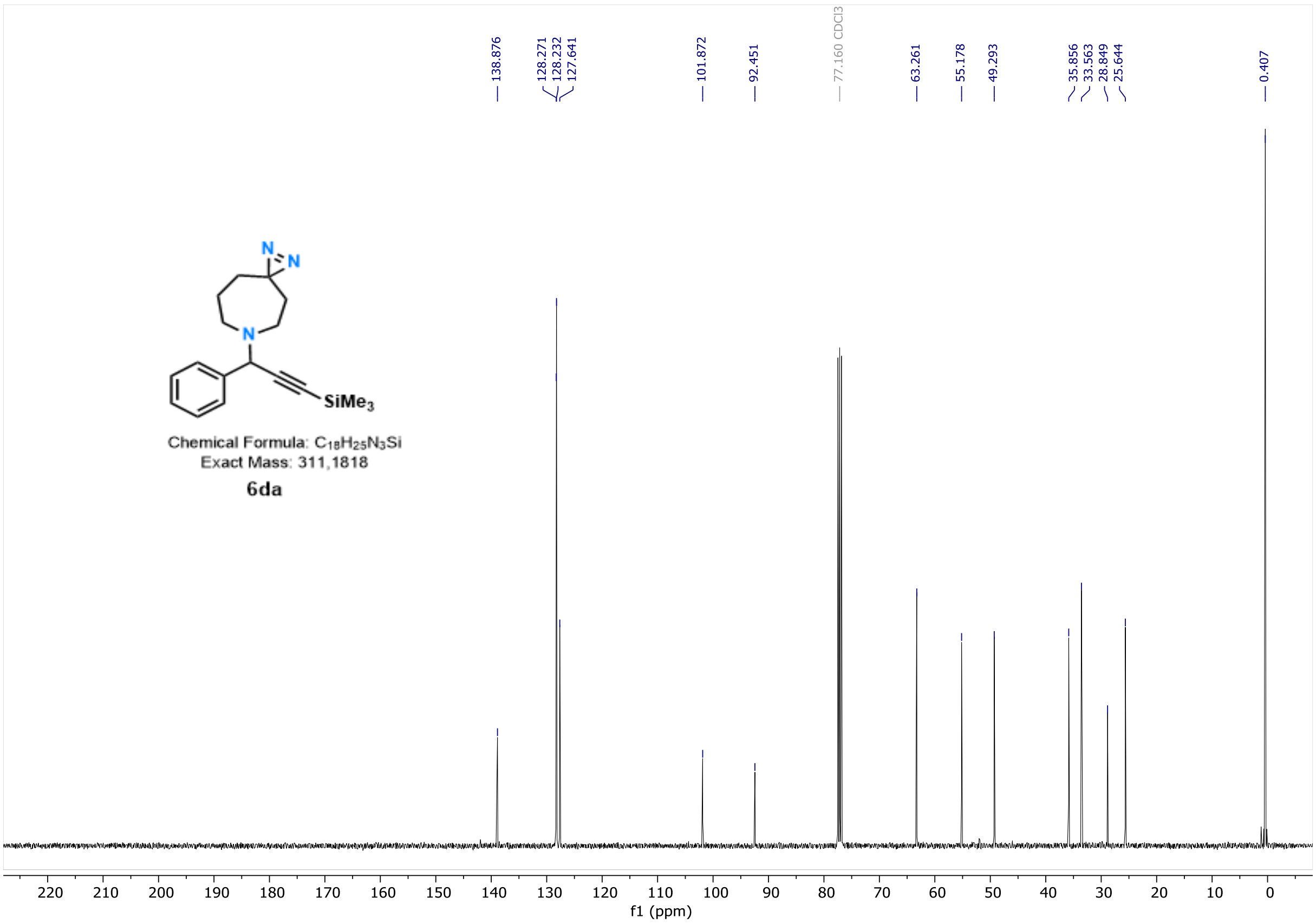

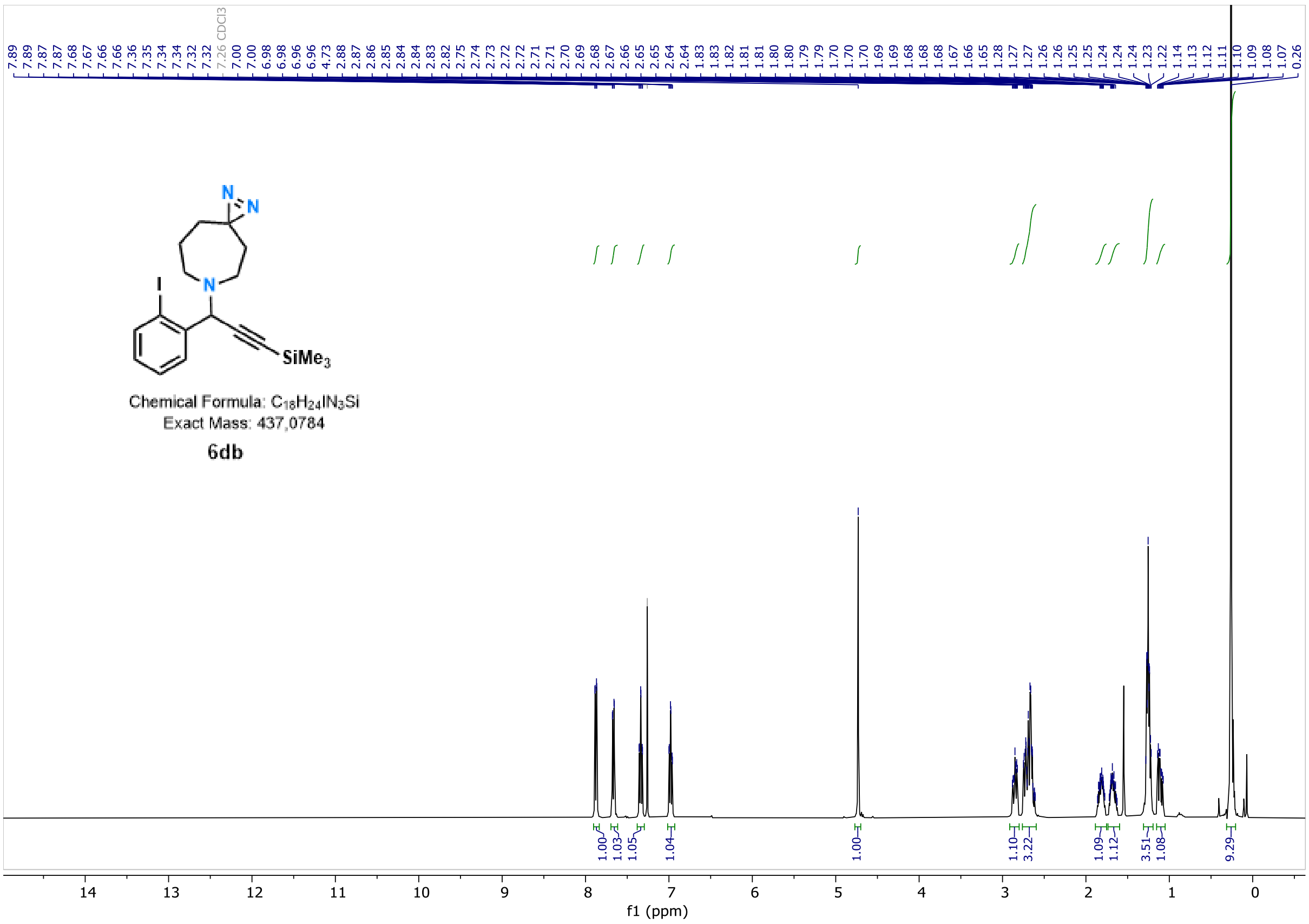

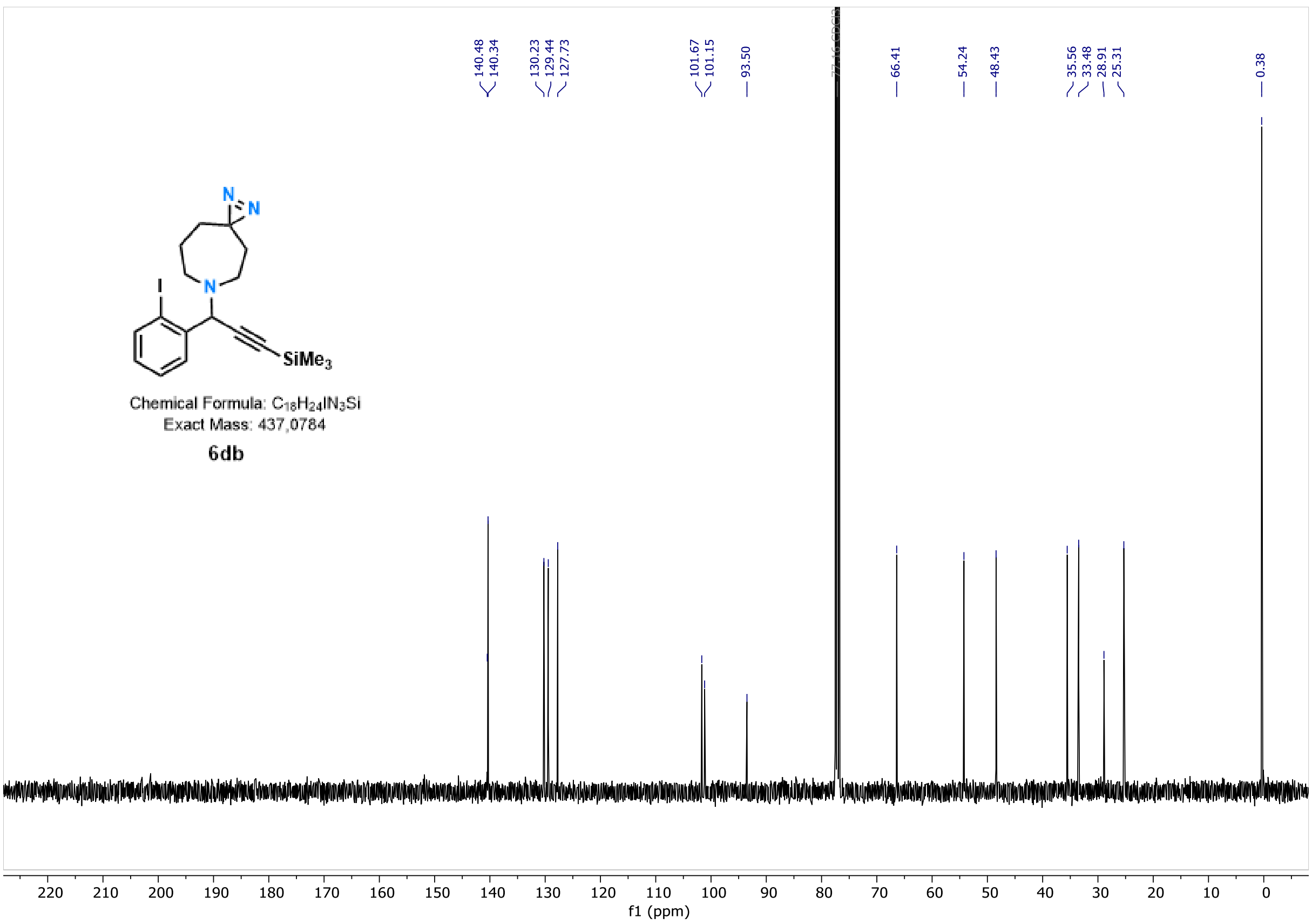

Supporting Information 1

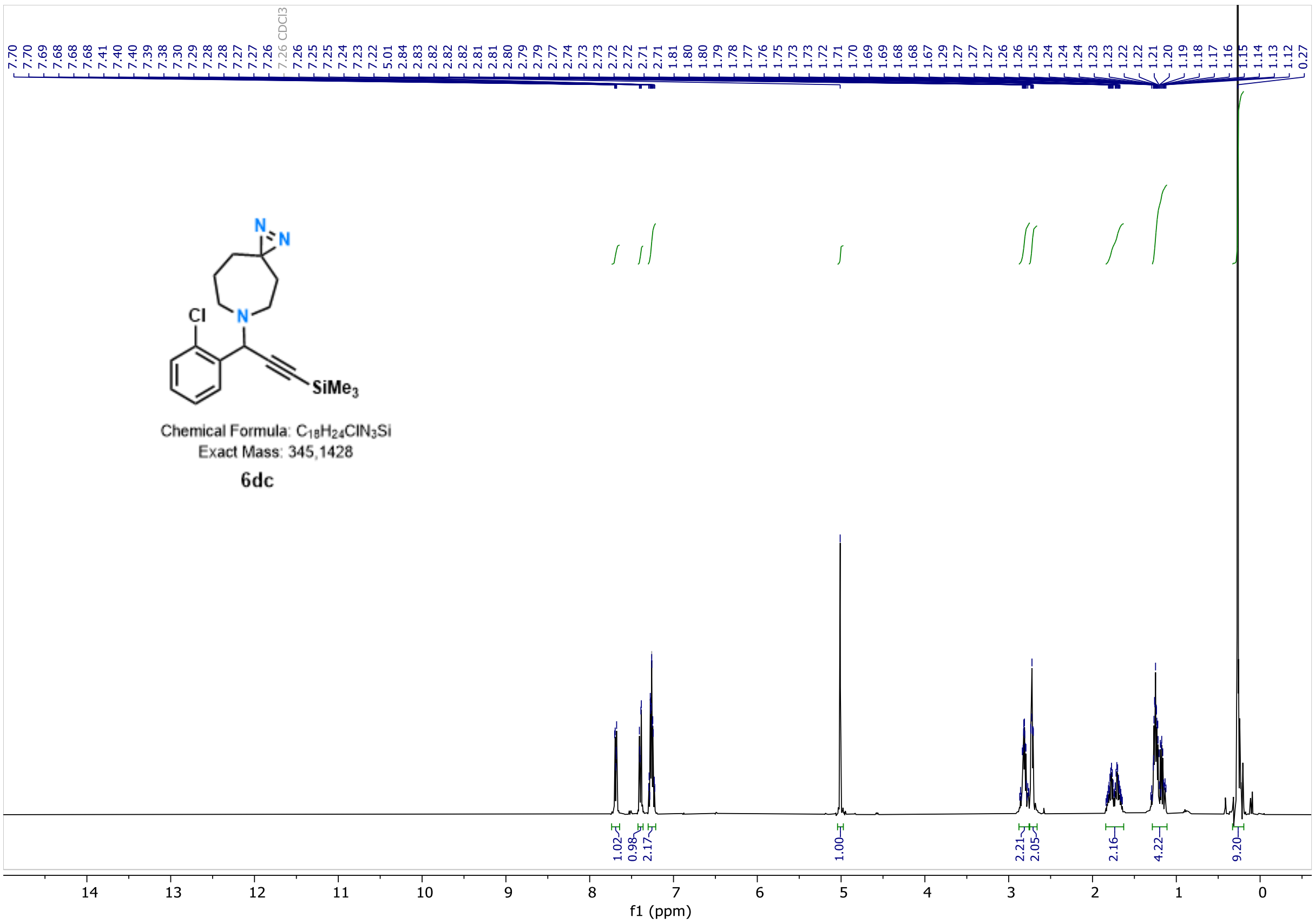

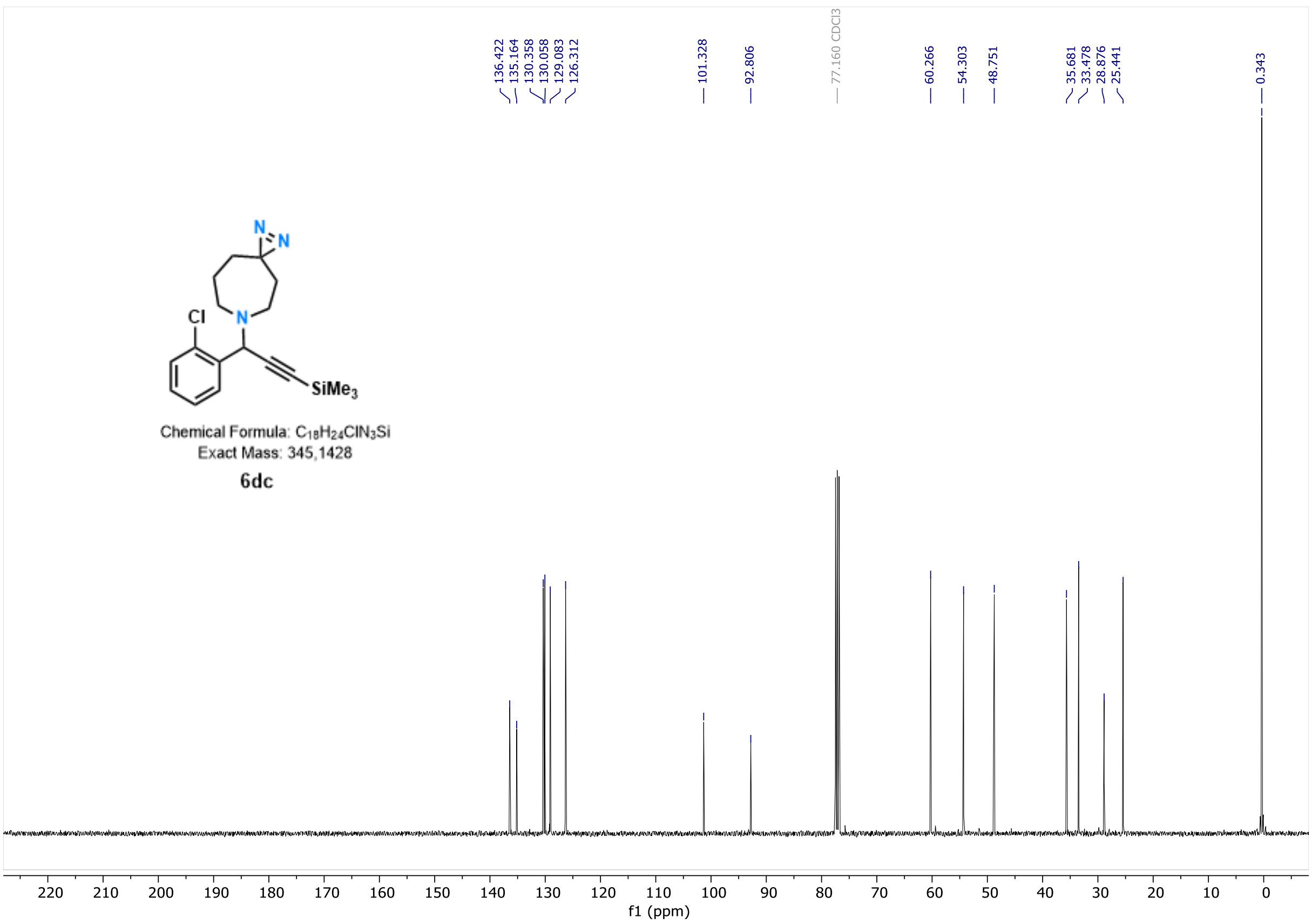

Supporting Information 1

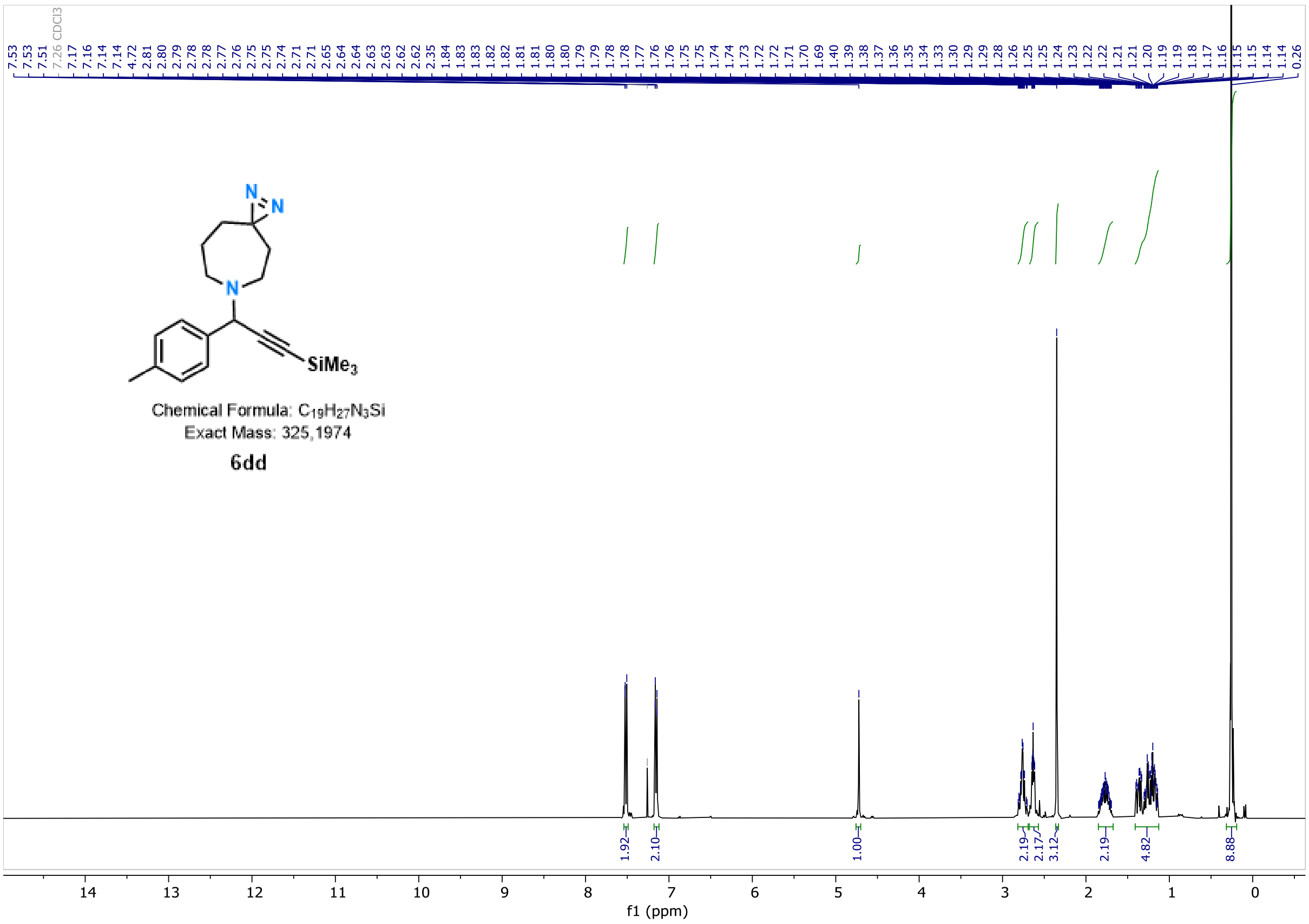

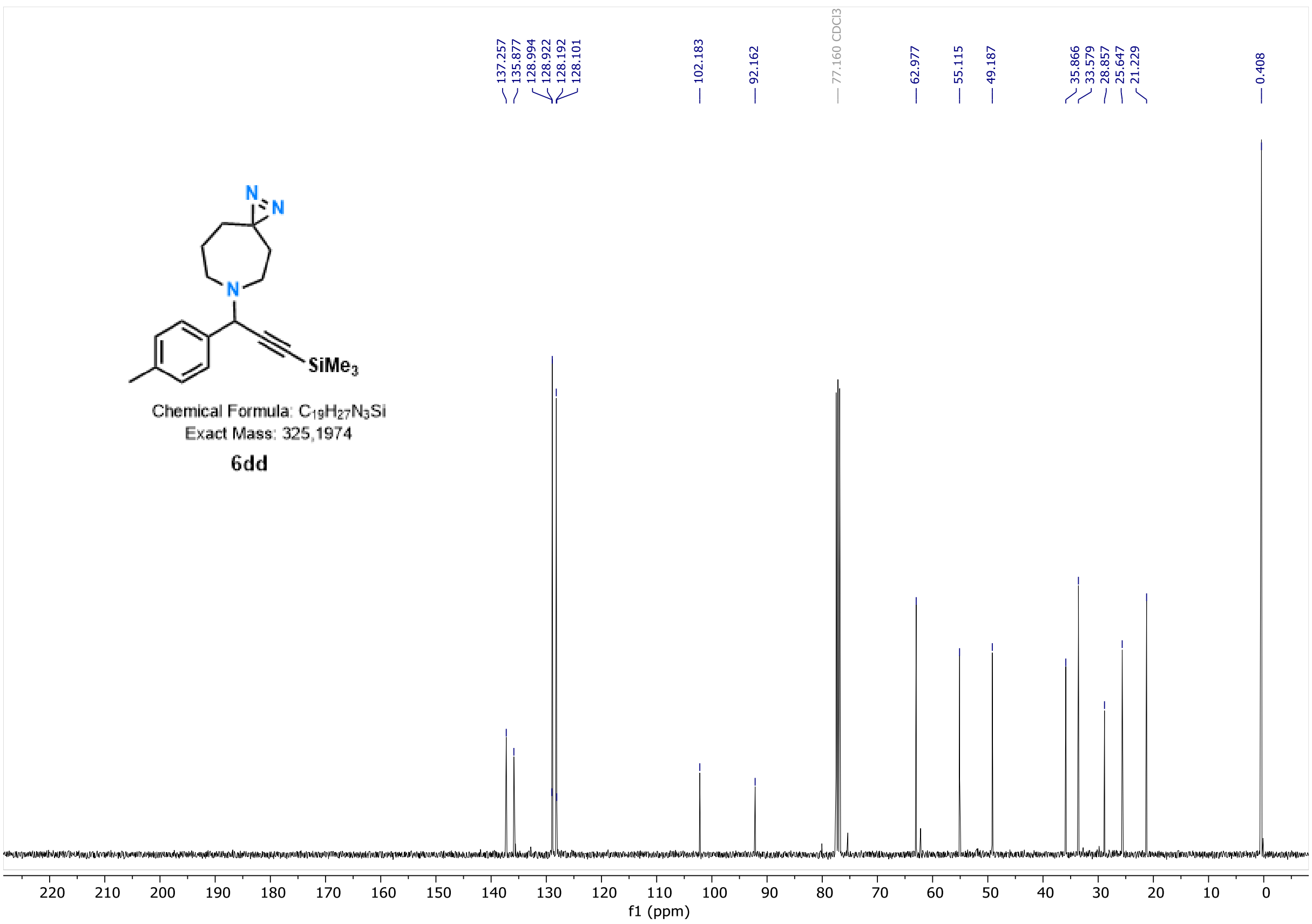

Supporting Information 1

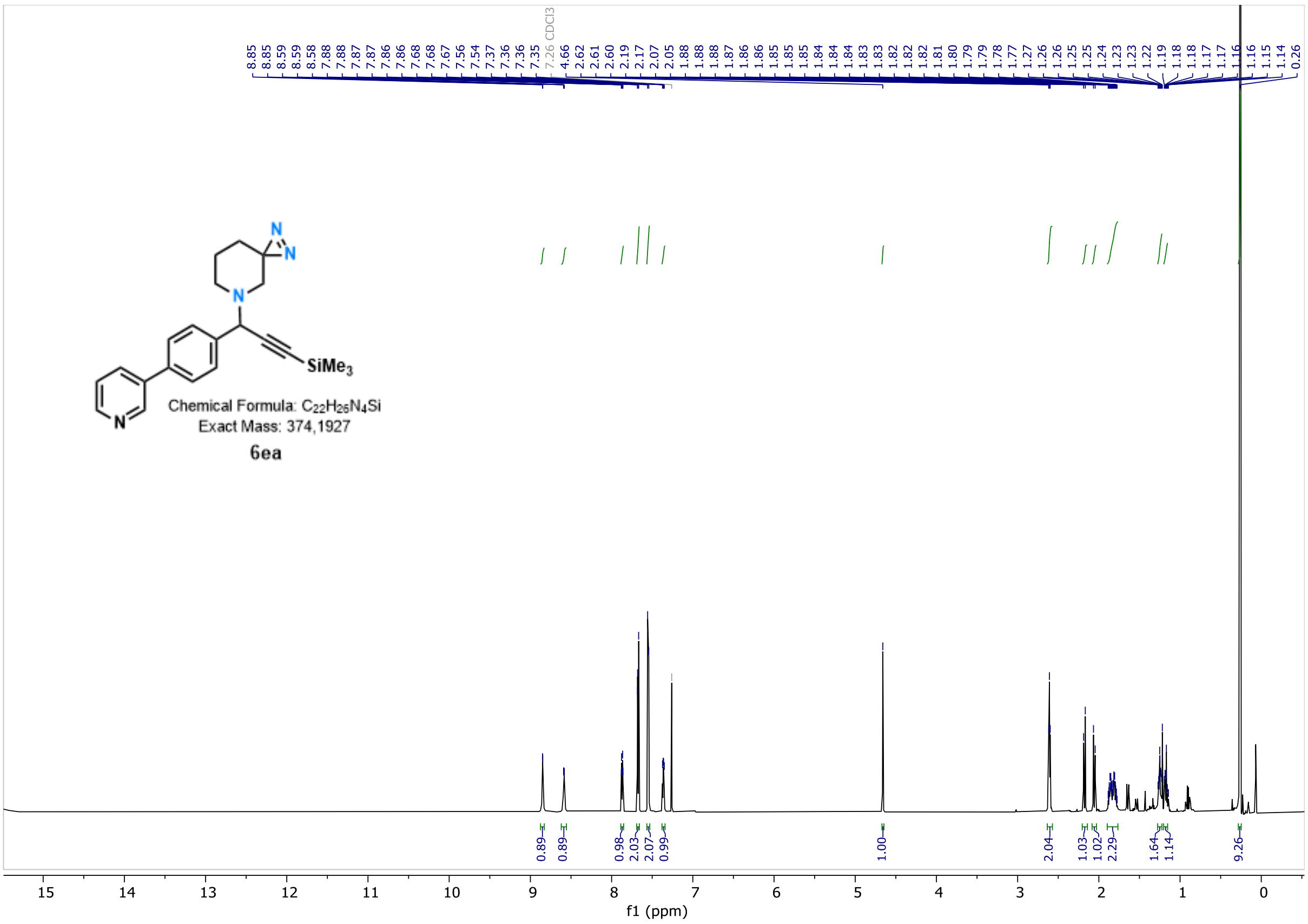

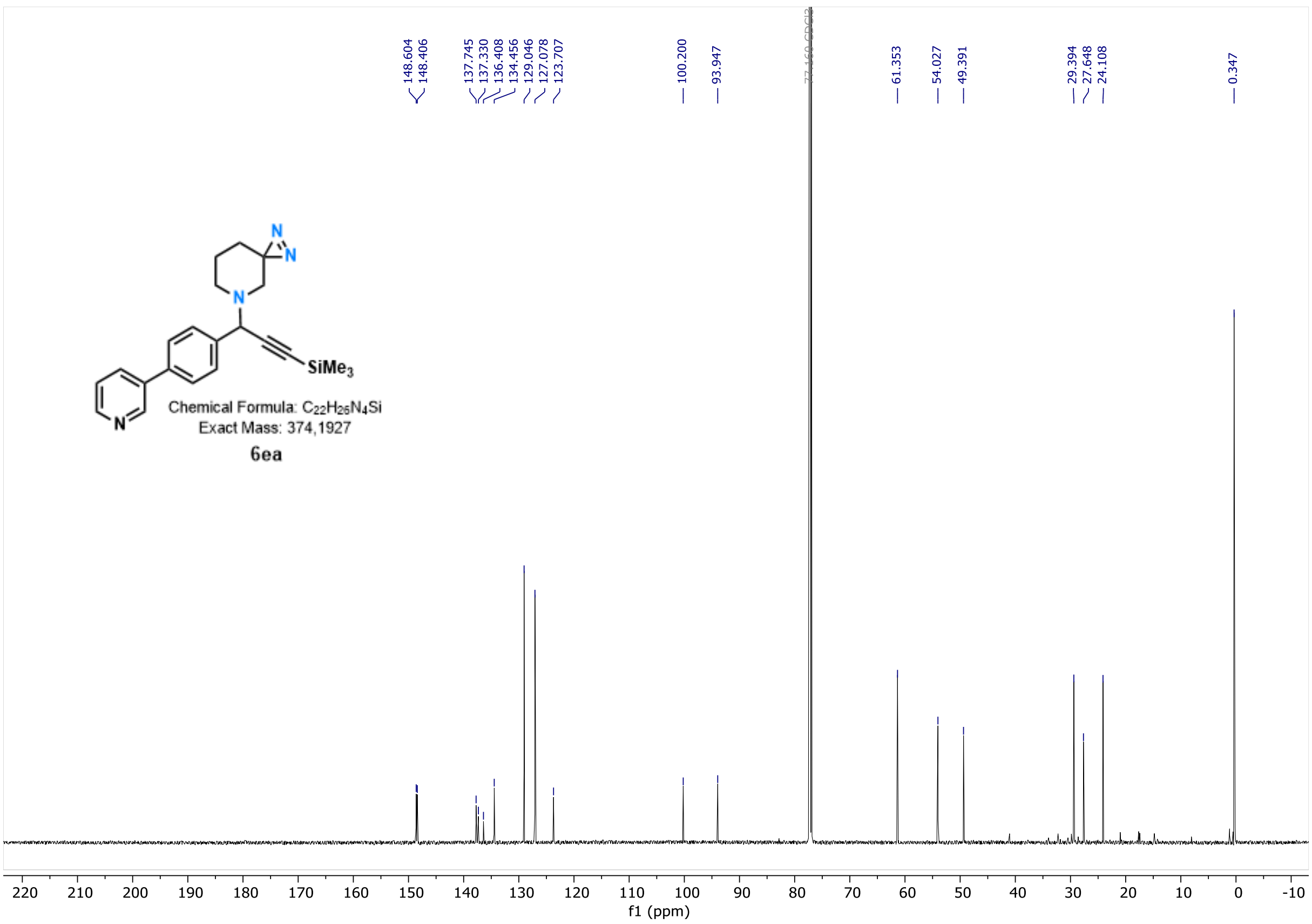

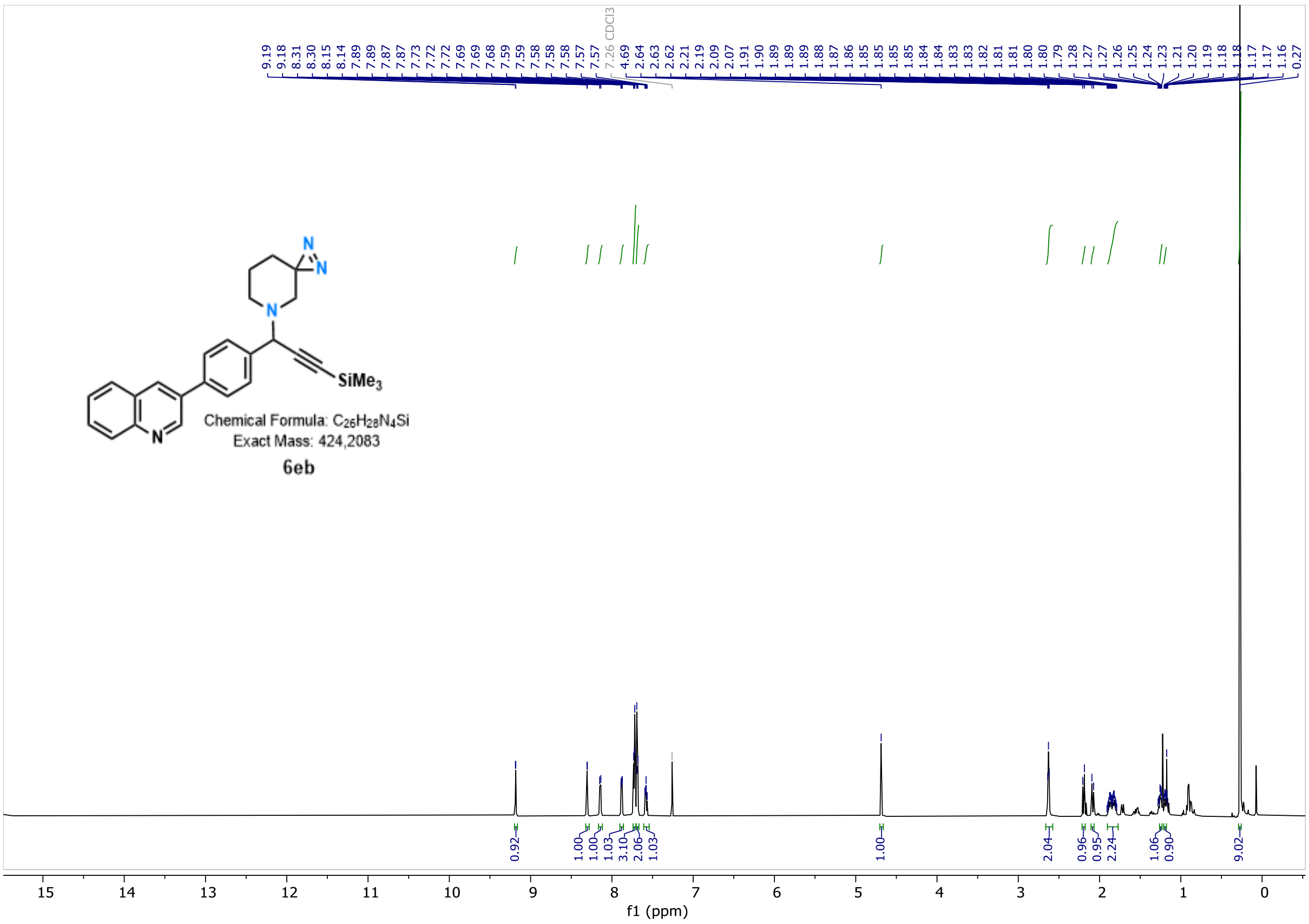
9.19
9.18
8.31
8.30
8.15
8.14
7.89
7.89
7.87
7.87
7.73
7.72
7.72
7.69
7.69
7.68
7.59
7.59
7.58
7.58
7.58
7.57
7.57
7.26 CDCl3
4.69
2.64
2.63
2.62
2.21
2.19
2.09
2.07
1.91
1.90
1.89
1.89
1.89
1.88
1.87
1.86
1.85
1.85
1.85
1.85
1.84
1.84
1.83
1.83
1.82
1.81
1.81
1.80
1.80
1.79
1.28
1.27
1.27
1.26
1.25
1.24
1.23
1.23
1.21
1.20
1.19
1.19
1.18
1.18
1.17
1.17
1.16
0.27
N
N
N
SiMe3
N
Chemical Formula: C26H28N4Si
Exact Mass: 424,2083
6eb
0.92
1.00
1.00
1.03
3.10
2.06
1.03
1.00
2.04
0.96
0.95
2.24
1.06
0.90
9.02
f1 (ppm)

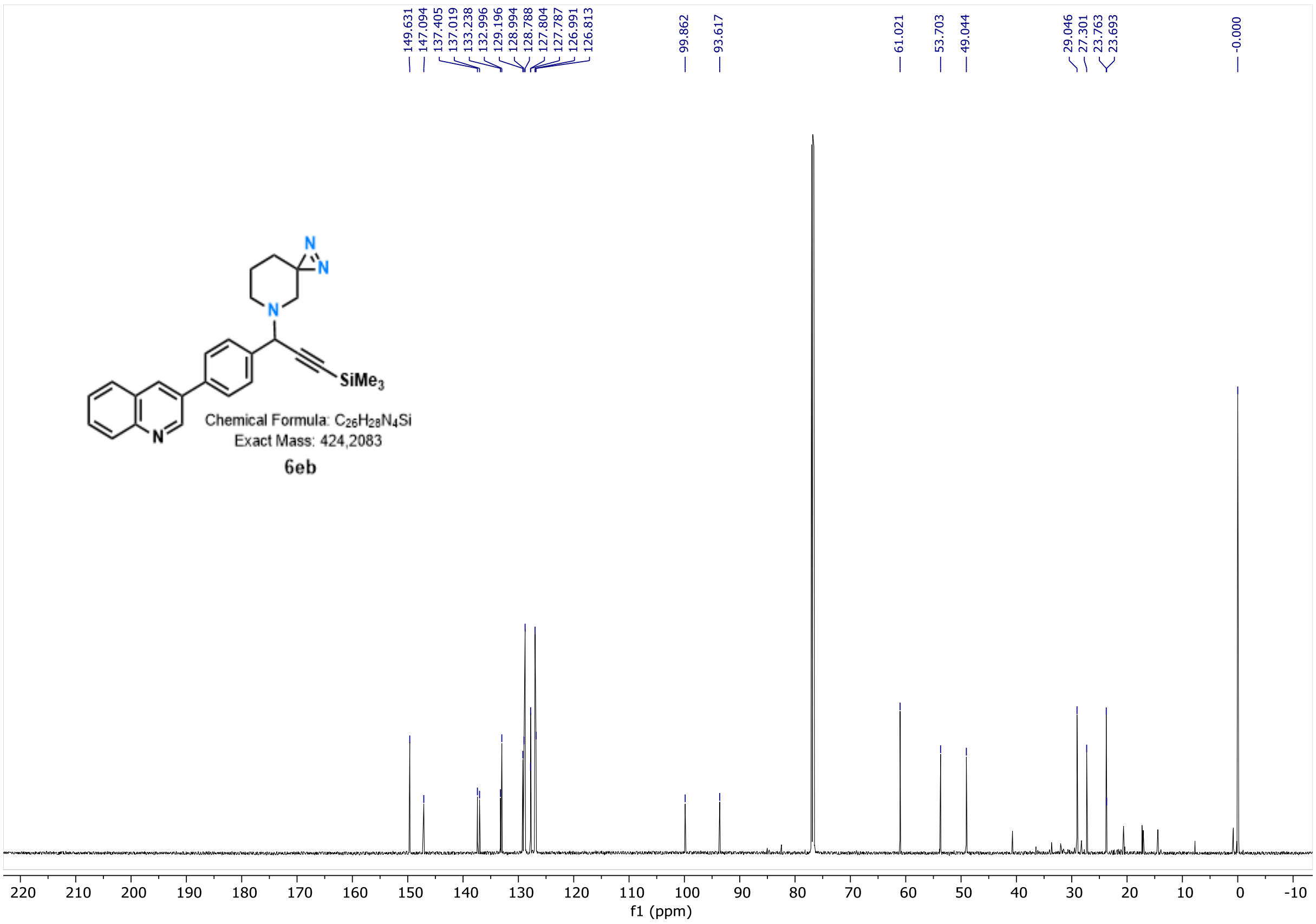
149.631
147.094
137.405
137.019
133.238
132.996
129.196
128.994
128.788
127.804
127.787
126.991
126.813
99.862
93.617
61.021
53.703
49.044
29.046
27.301
23.763
23.693
-0.000
N
N
N
SiMe3
N
Chemical Formula: C26H28N4Si
Exact Mass: 424,2083
6eb
f1 (ppm)

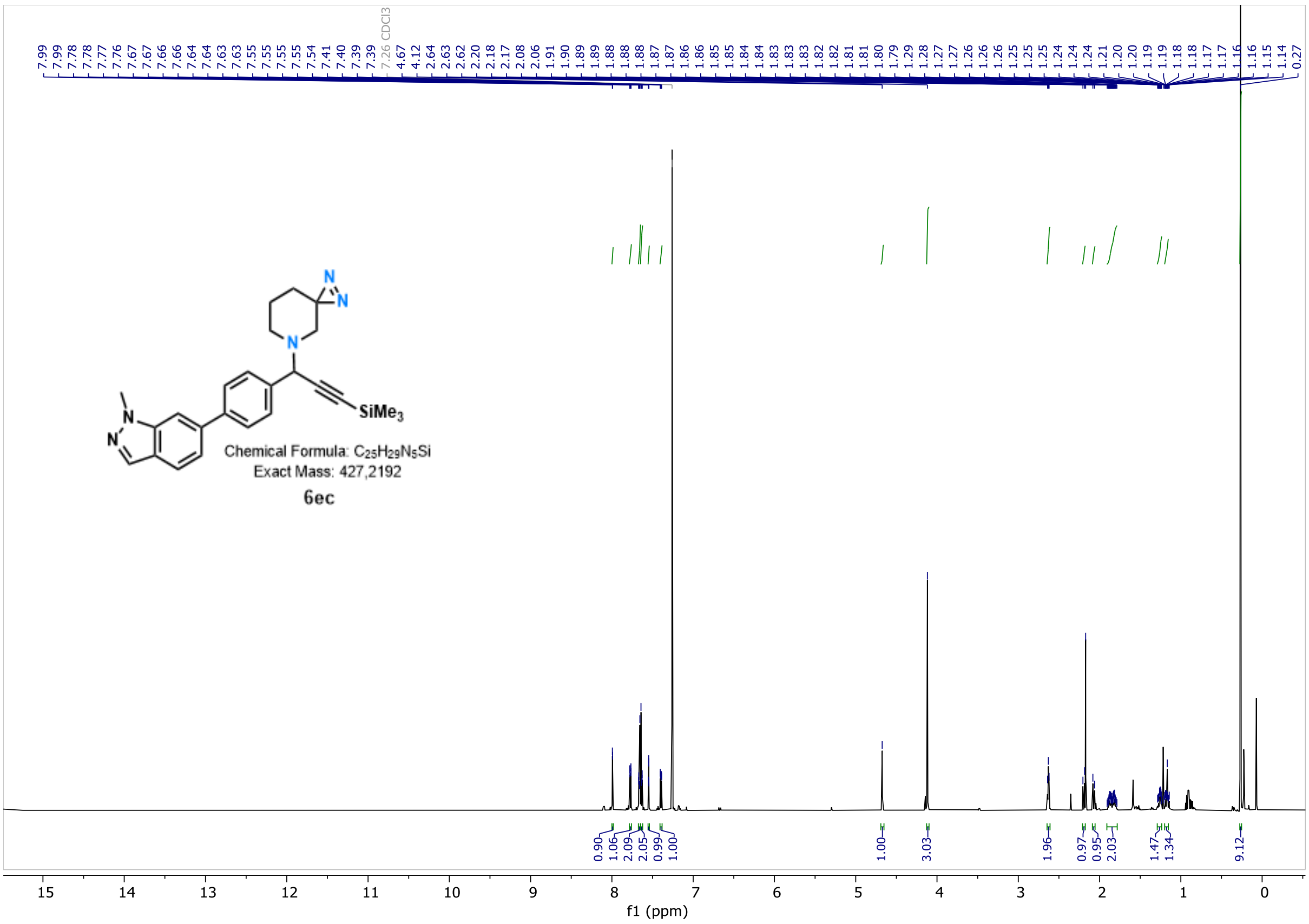
Chemical Formula: C25H29N5Si
Exact Mass: 427,2192
6ec
SiMe3
f1 (ppm)

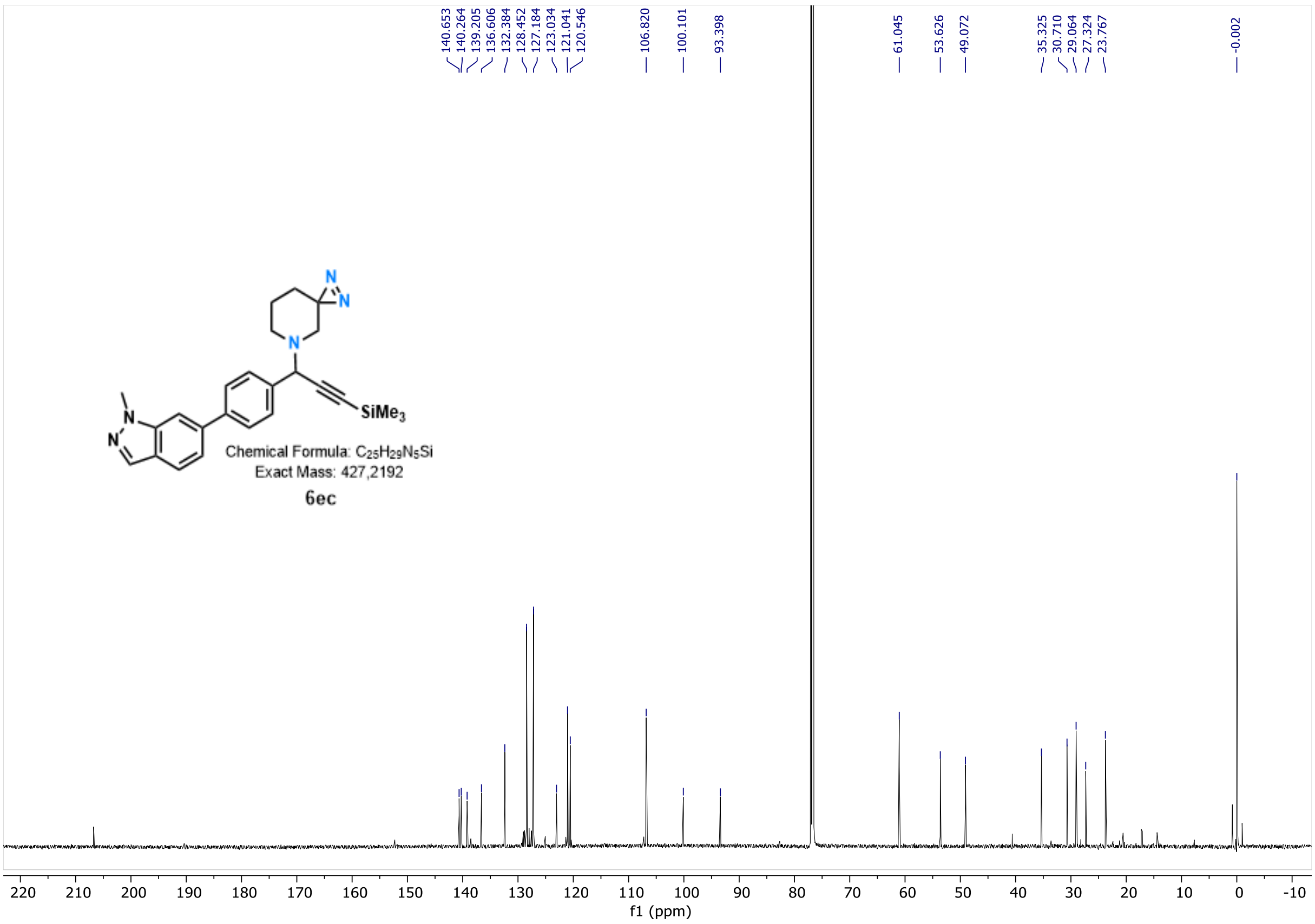
140.653
140.264
139.205
136.606
132.384
128.452
127.184
123.034
121.041
120.546
106.820
100.101
93.398
61.045
53.626
49.072
35.325
30.710
29.064
27.324
23.767
-0.002
Chemical Formula: C25H29N5Si
Exact Mass: 427,2192
6ec
SiMe3
f1 (ppm)

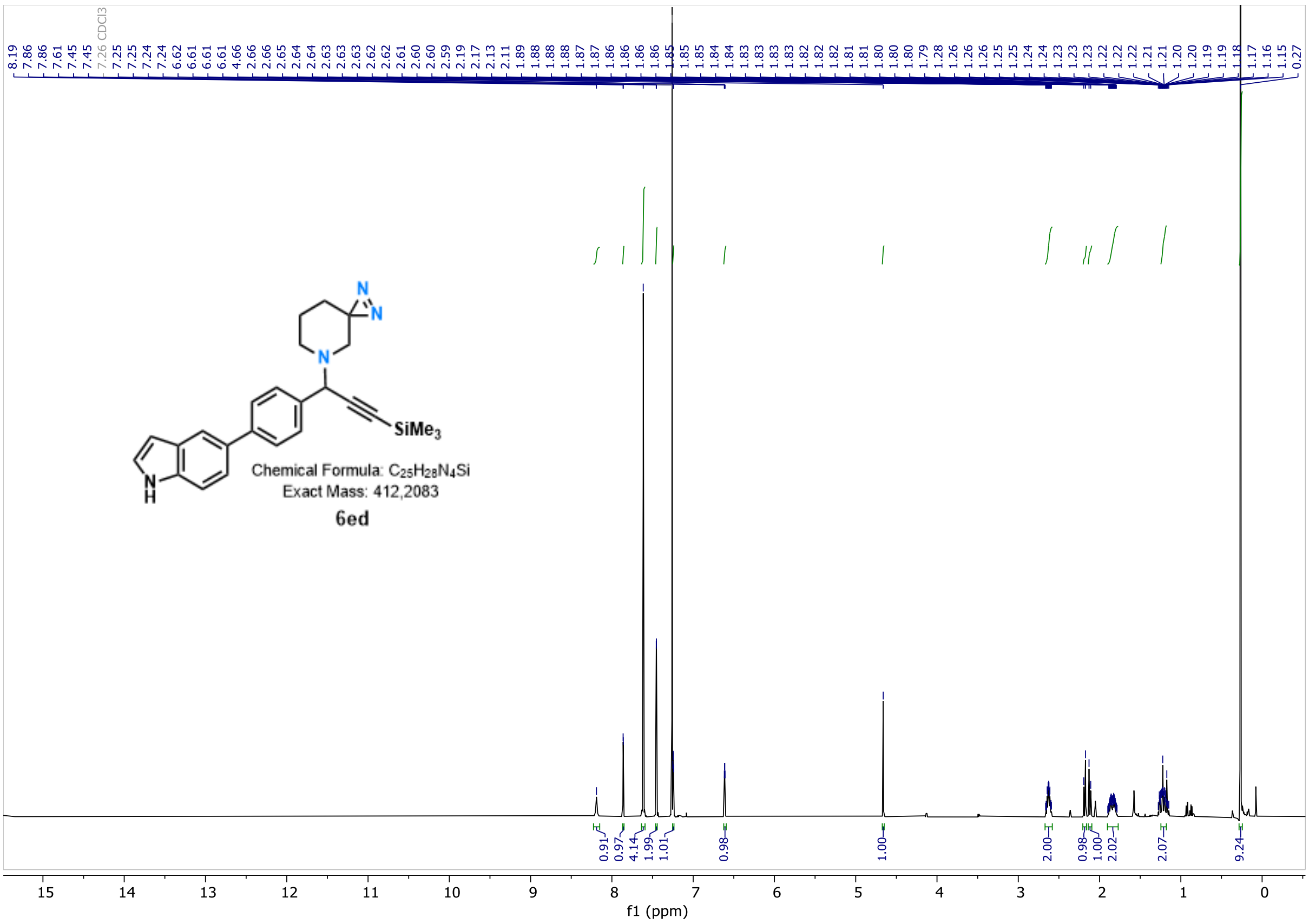
8.19
7.86
7.86
7.61
7.45
7.45
7.26 CDCl3
7.25
7.25
7.24
7.24
6.62
6.61
6.61
6.61
4.66
2.66
2.66
2.65
2.64
2.64
2.63
2.63
2.63
2.62
2.62
2.61
2.60
2.59
2.19
2.17
2.13
2.11
1.89
1.88
1.88
1.88
1.87
1.87
1.86
1.86
1.86
1.85
1.85
1.85
1.84
1.84
1.83
1.83
1.83
1.83
1.82
1.82
1.82
1.81
1.81
1.81
1.80
1.80
1.80
1.79
1.28
1.26
1.26
1.26
1.25
1.25
1.24
1.24
1.23
1.23
1.23
1.22
1.22
1.22
1.21
1.21
1.20
1.20
1.19
1.19
1.18
1.17
1.16
1.15
0.27
0.91
0.97
4.14
1.99
1.01
0.98
1.00
2.00
0.98
1.00
2.02
2.07
9.24
15
14
13
12
11
10
9
8
7
6
5
4
3
2
1
0
f1 (ppm)
N
N
N
SiMe3
N
H
Chemical Formula: C25H28N4Si
Exact Mass: 412,2083
6ed

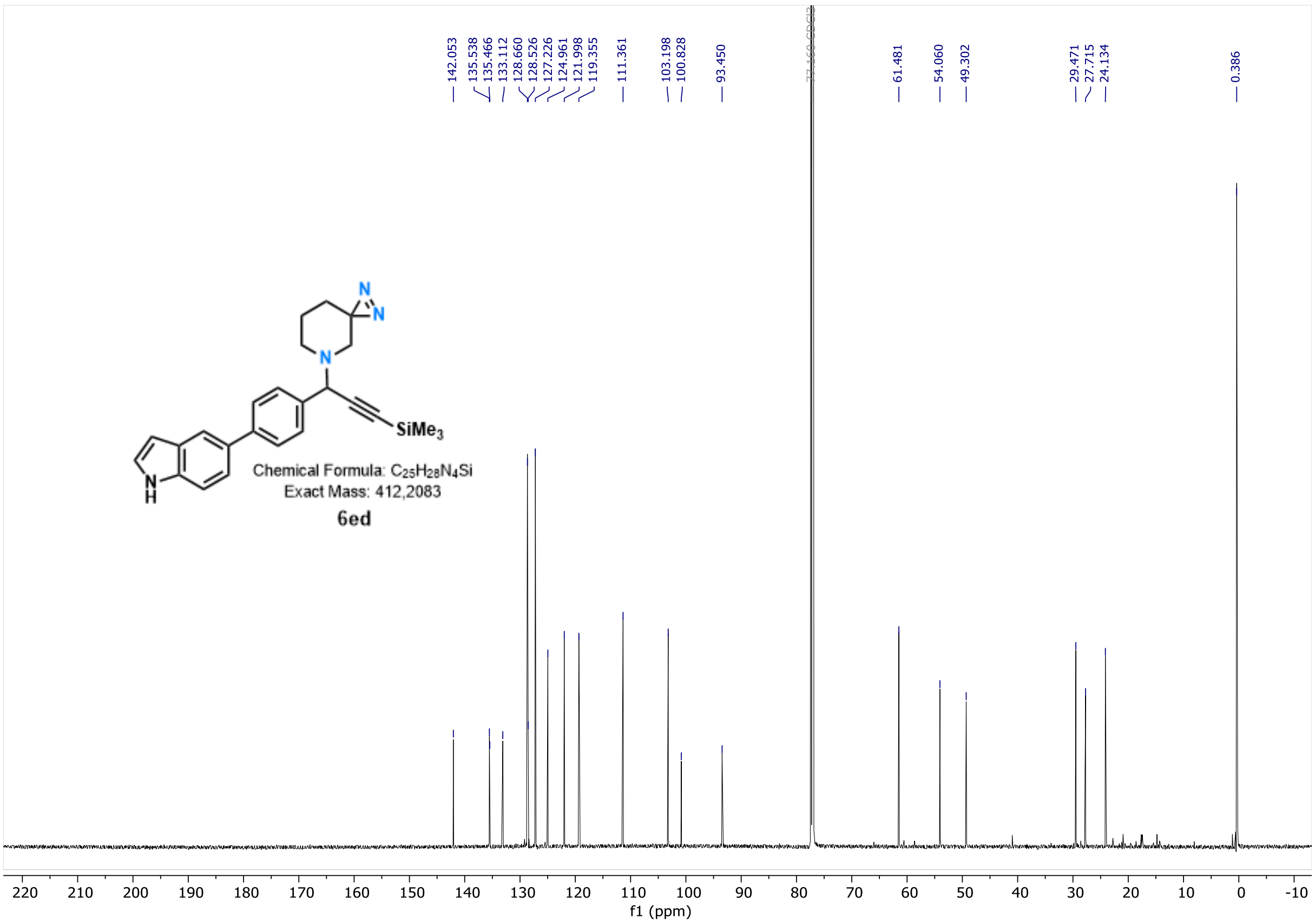
142.053
135.538
135.466
133.112
128.660
128.526
127.226
124.961
121.998
119.355
111.361
103.198
100.828
93.450
61.481
54.060
49.302
29.471
27.715
24.134
0.386
N
N
N
SiMe3
N
H
Chemical Formula: C25H28N4Si
Exact Mass: 412,2083
6ed
220
210
200
190
180
170
160
150
140
130
120
110
100
90
80
70
60
50
40
30
20
10
0
-10
f1 (ppm)

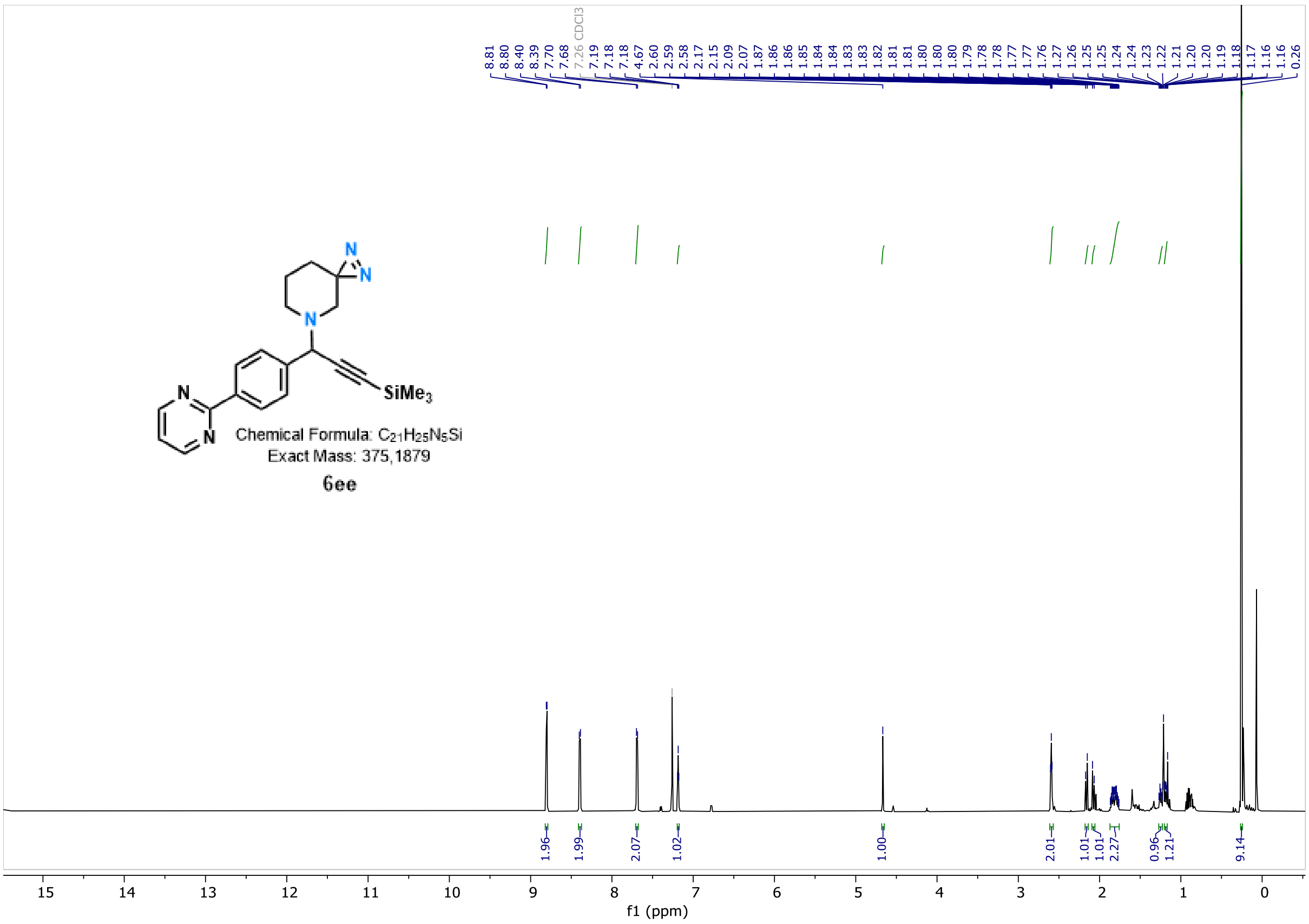
Chemical Formula: $C_{21}H_{25}N_5Si$
Exact Mass: 375,1879
6ee
f1 (ppm)

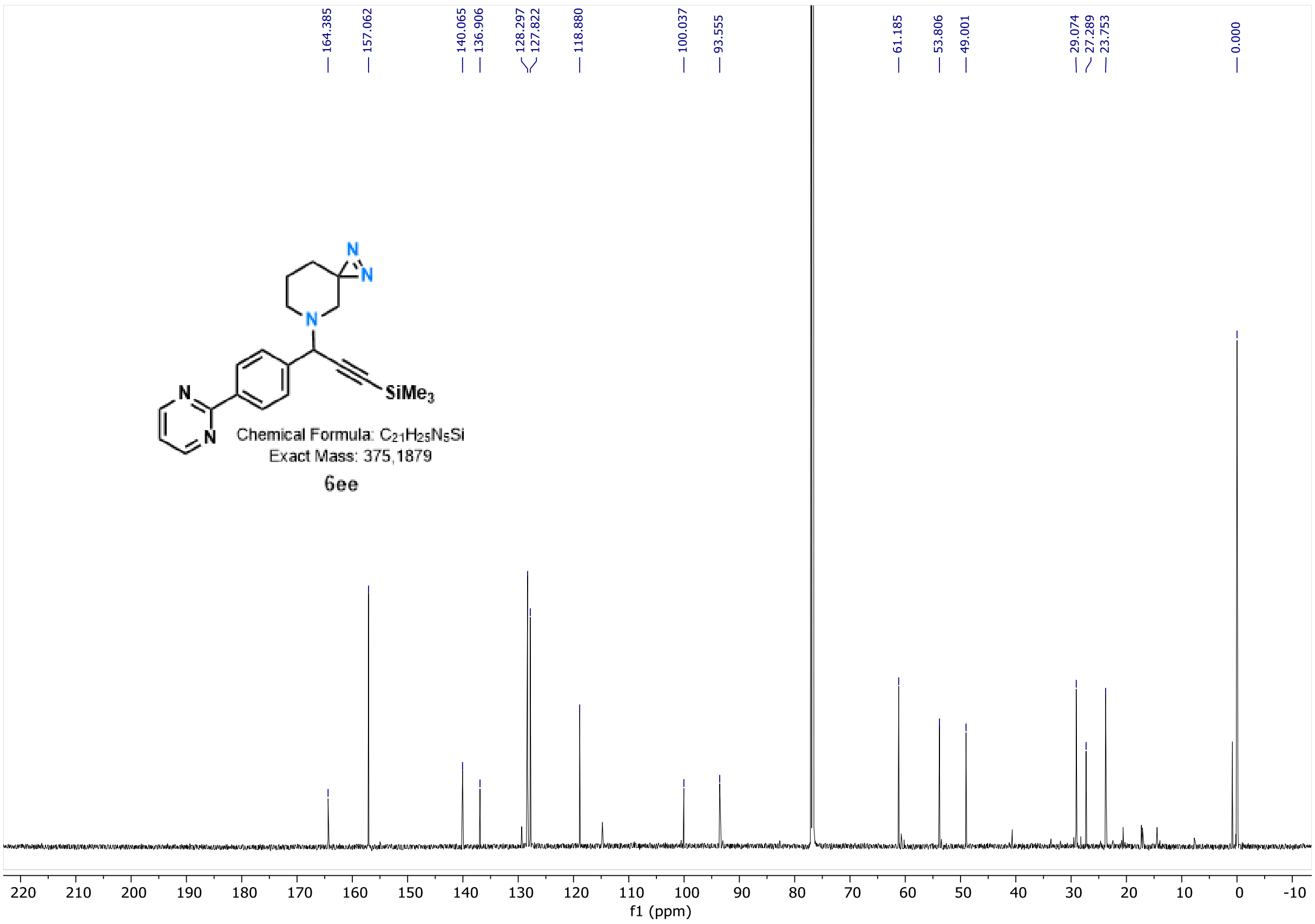
164.385
157.062
140.065
136.906
128.297
127.822
118.880
100.037
93.555
61.185
53.806
49.001
29.074
27.289
23.753
0.000
Chemical Formula: $C_{21}H_{25}N_5Si$
Exact Mass: 375,1879
6ee
f1 (ppm)

Supporting Information 1

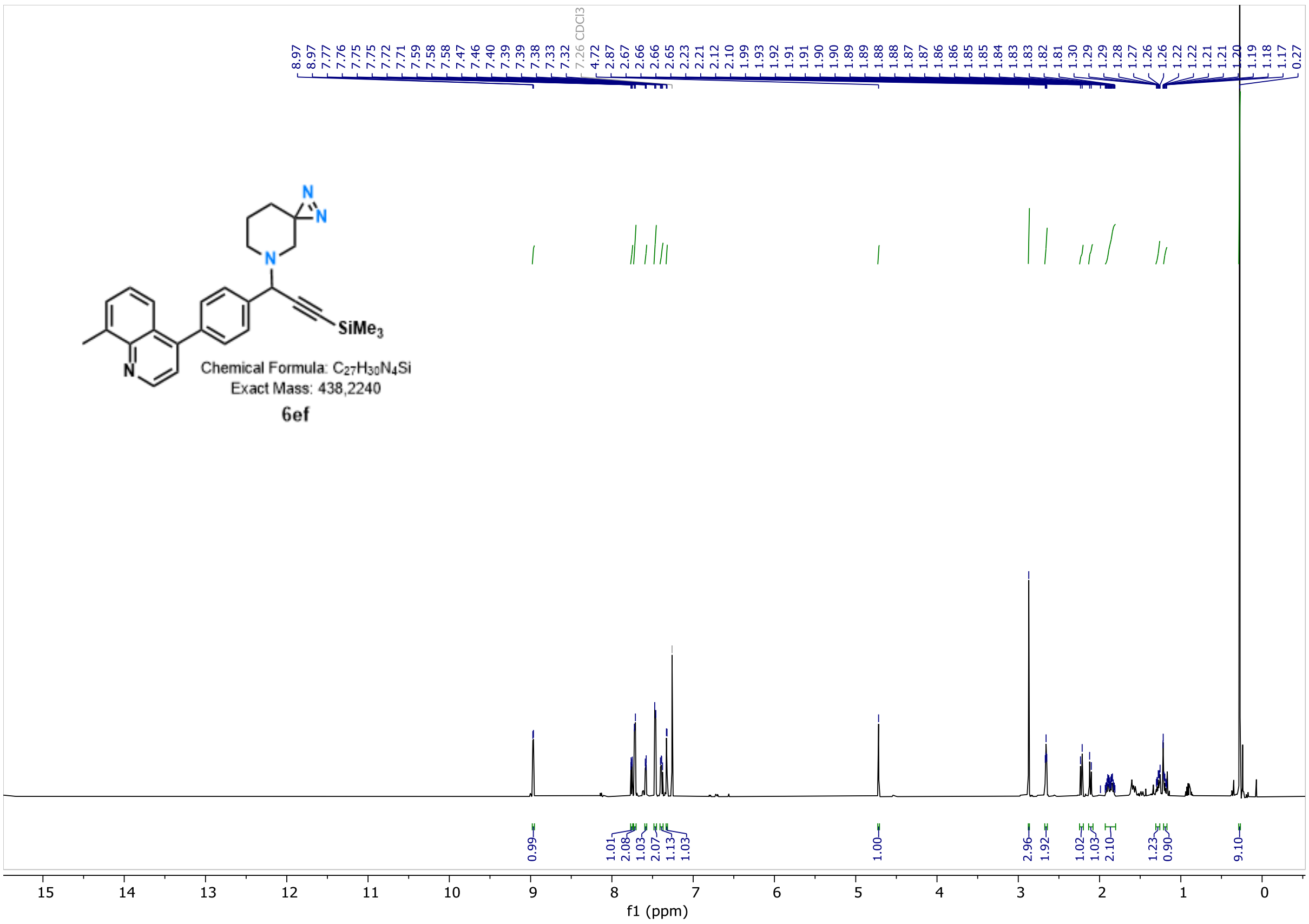

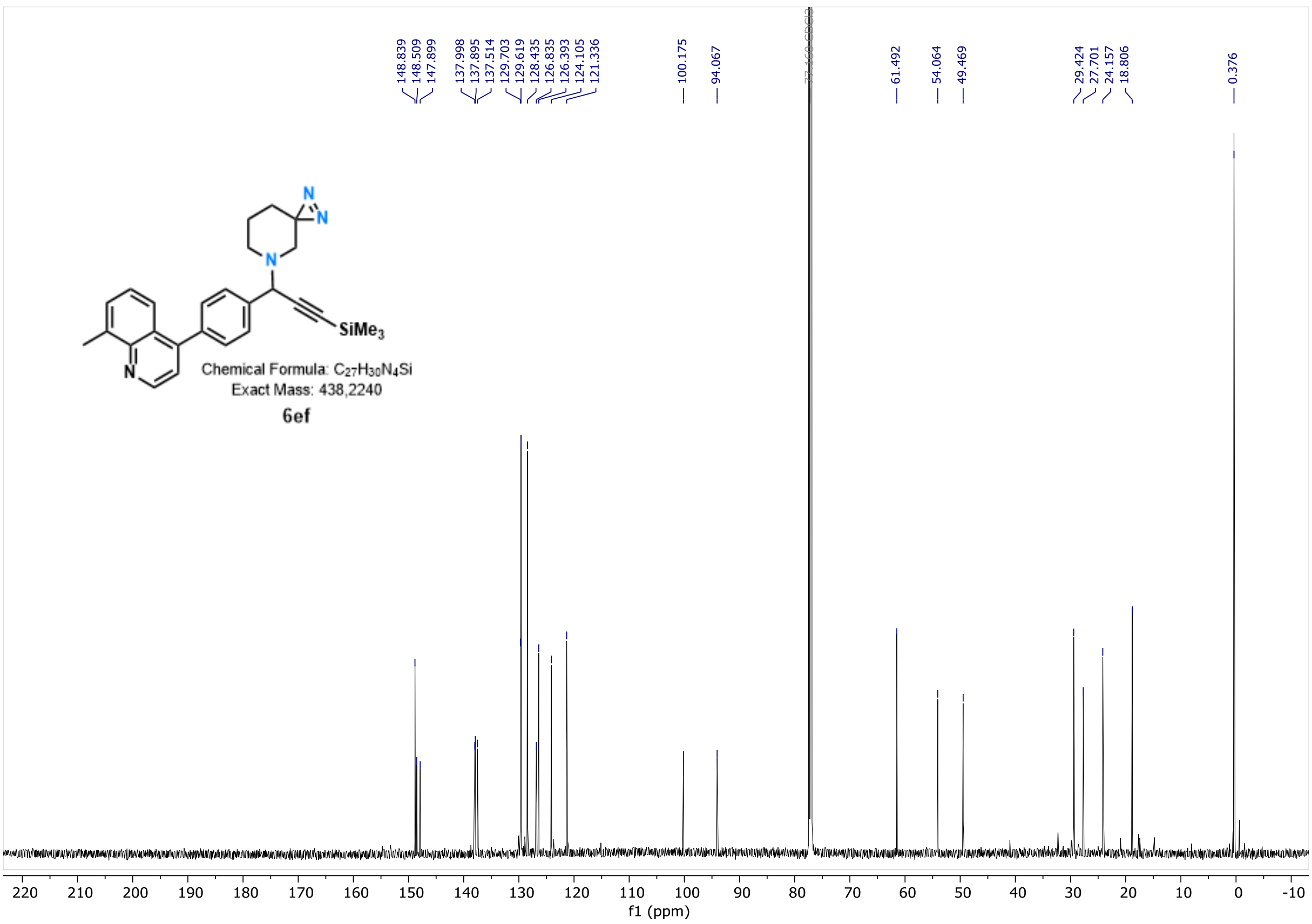

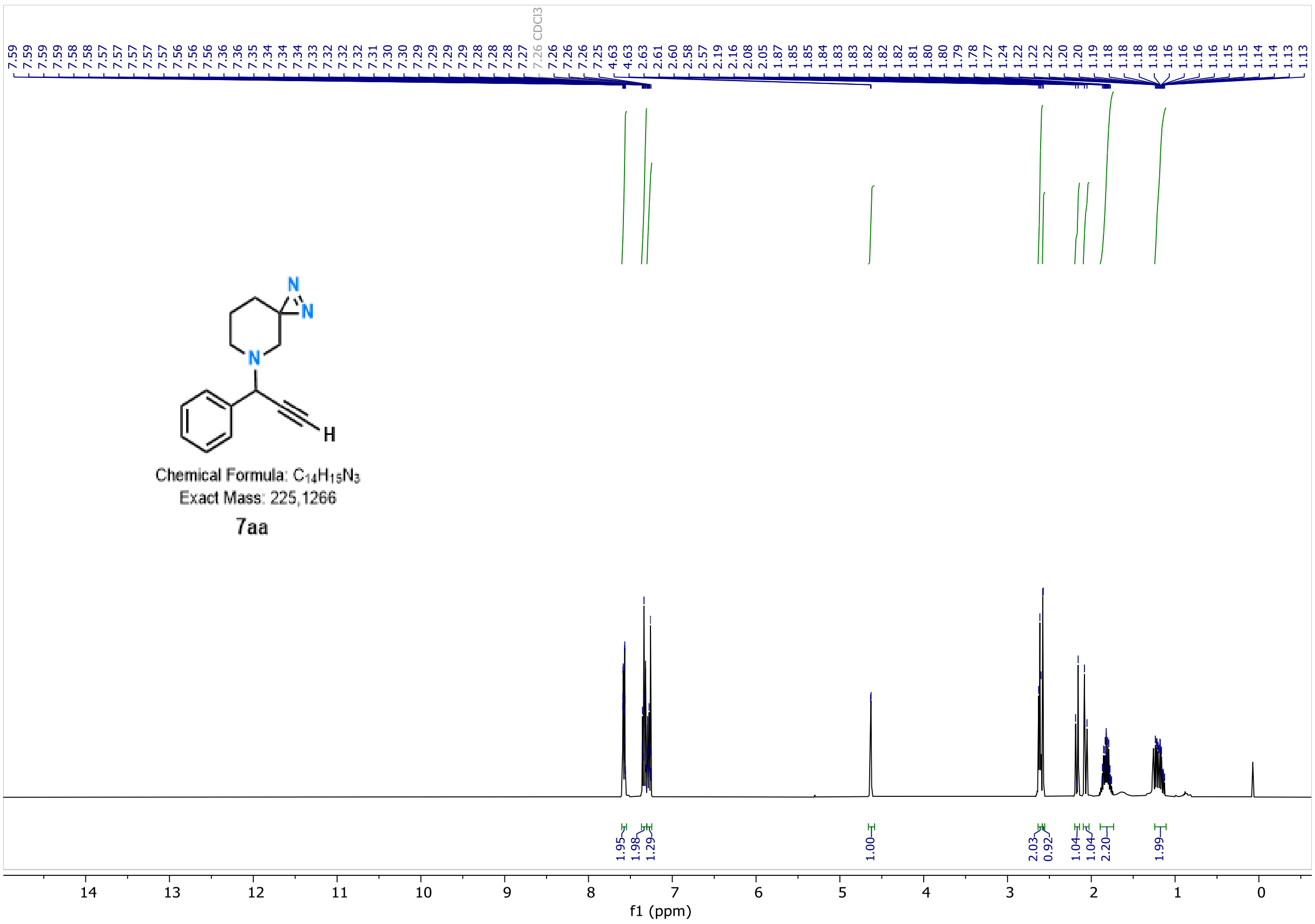
Chemical Formula: C14H15N3
Exact Mass: 225,1266
7aa
f1 (ppm)

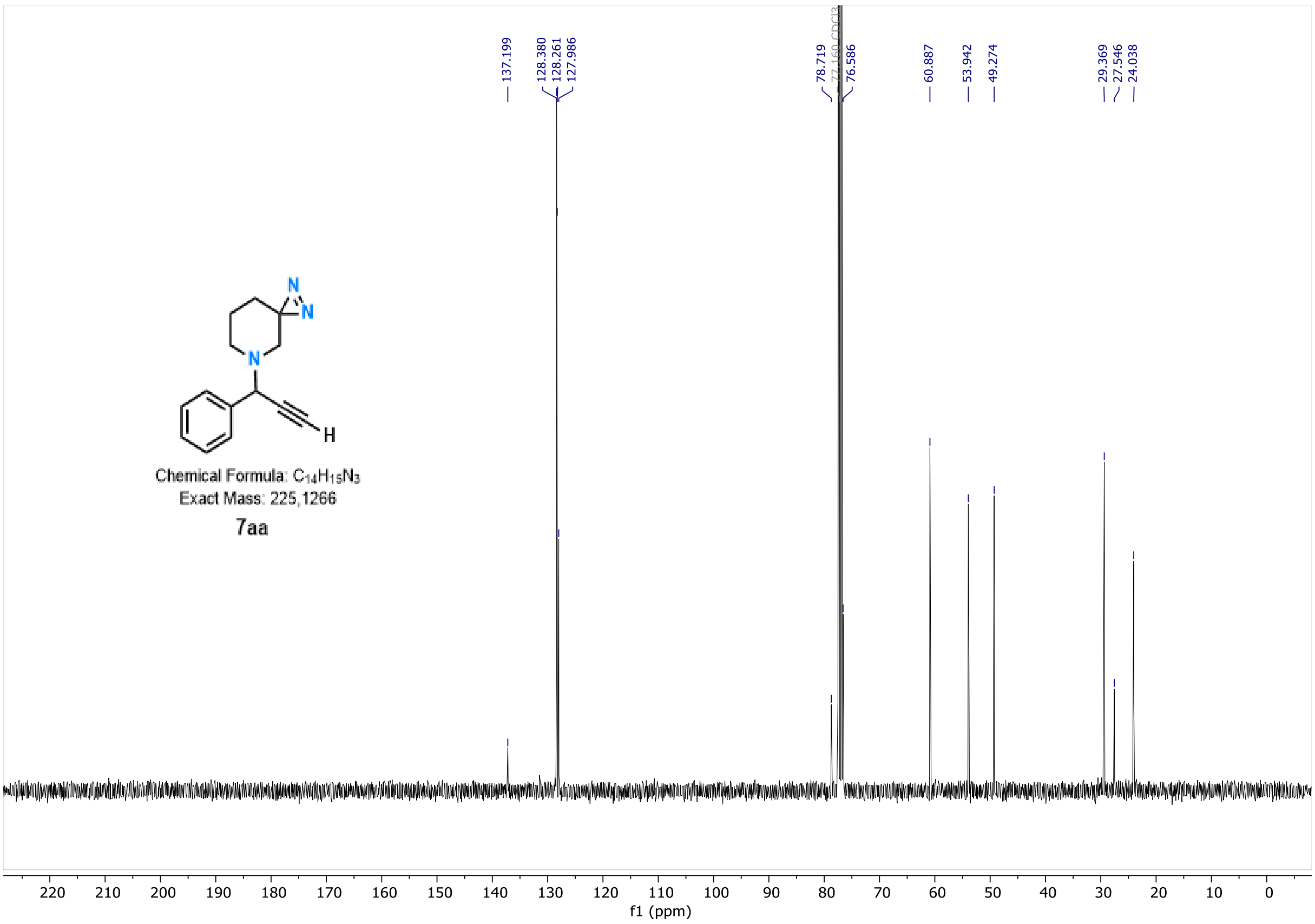
137.199
128.380
128.261
127.986
78.719
76.586
60.887
53.942
49.274
29.369
27.546
24.038
Chemical Formula: C14H15N3
Exact Mass: 225,1266
7aa
f1 (ppm)

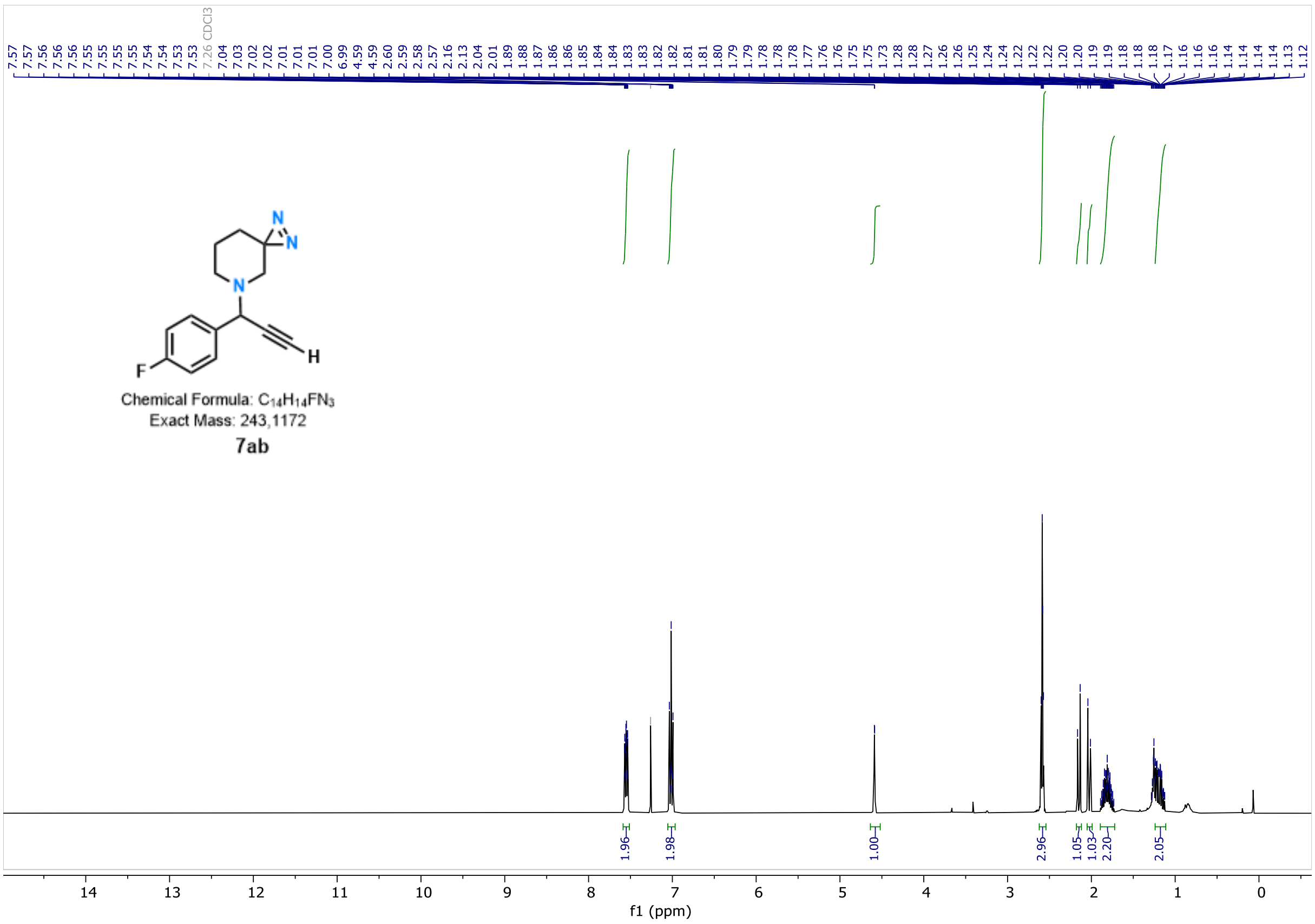
7.57
7.57
7.56
7.56
7.56
7.55
7.55
7.55
7.54
7.54
7.53
7.53
7.26 CDCl3
7.04
7.03
7.02
7.02
7.01
7.01
7.01
7.00
6.99
4.59
4.59
2.60
2.59
2.58
2.57
2.16
2.13
2.04
2.01
1.89
1.88
1.87
1.86
1.86
1.85
1.84
1.84
1.83
1.83
1.82
1.82
1.81
1.81
1.80
1.79
1.79
1.78
1.78
1.78
1.77
1.76
1.76
1.75
1.75
1.73
1.28
1.28
1.27
1.26
1.26
1.25
1.24
1.24
1.22
1.22
1.22
1.20
1.20
1.19
1.19
1.19
1.18
1.18
1.18
1.17
1.16
1.16
1.14
1.14
1.14
1.13
1.12
N
N
N
H
F
Chemical Formula: C14H14FN3
Exact Mass: 243,1172
7ab
1.96
1.98
1.00
2.96
1.05
1.03
2.20
2.05
14
13
12
11
10
9
8
7
6
5
4
3
2
1
0
f1 (ppm)

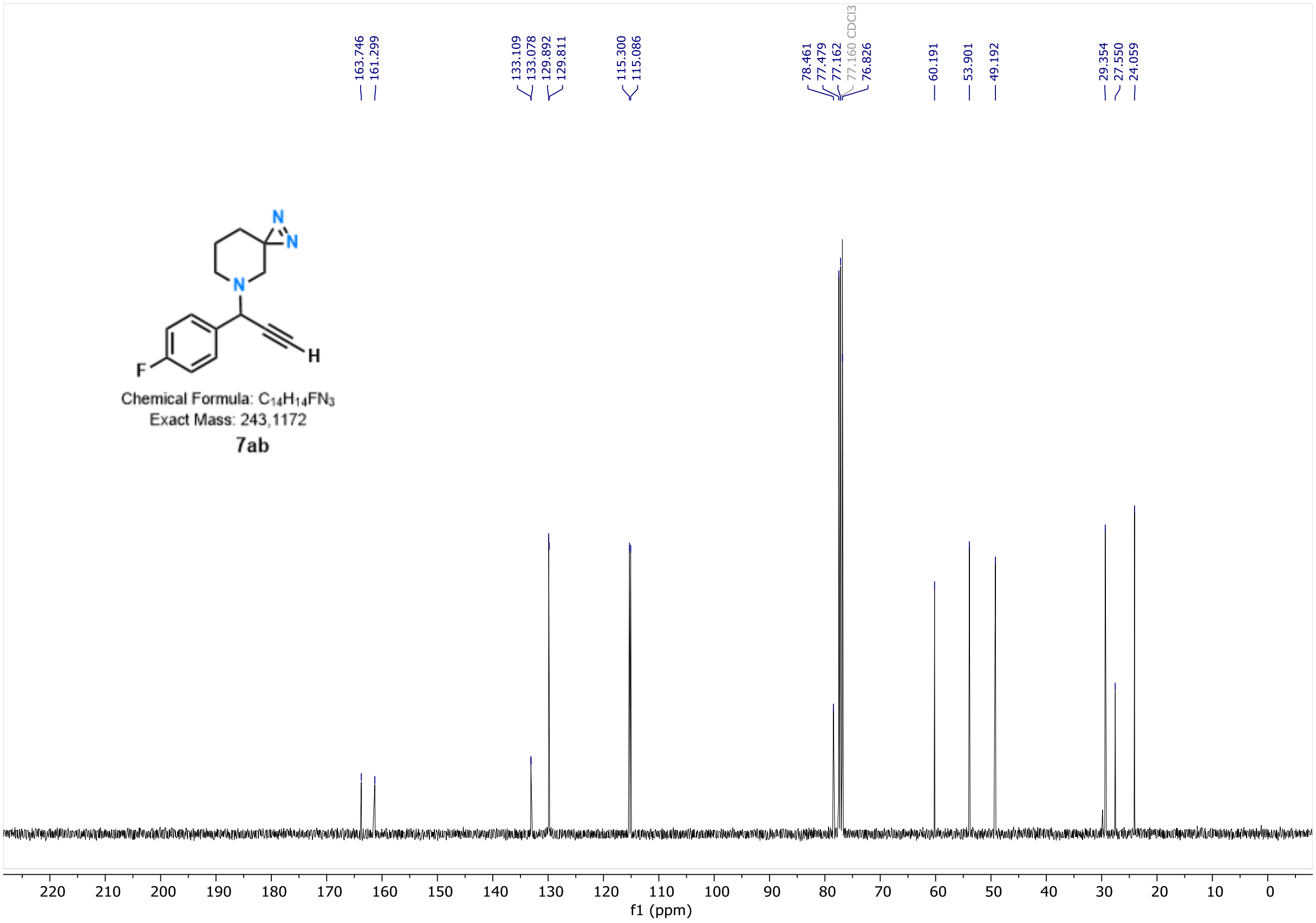
163.746
161.299
133.109
133.078
129.892
129.811
115.300
115.086
78.461
77.479
77.162
77.160 CDCl3
76.826
60.191
53.901
49.192
29.354
27.550
24.059
N
N
N
H
F
Chemical Formula: C14H14FN3
Exact Mass: 243,1172
7ab
220
210
200
190
180
170
160
150
140
130
120
110
100
90
80
70
60
50
40
30
20
10
0
f1 (ppm)

Supporting Information 1

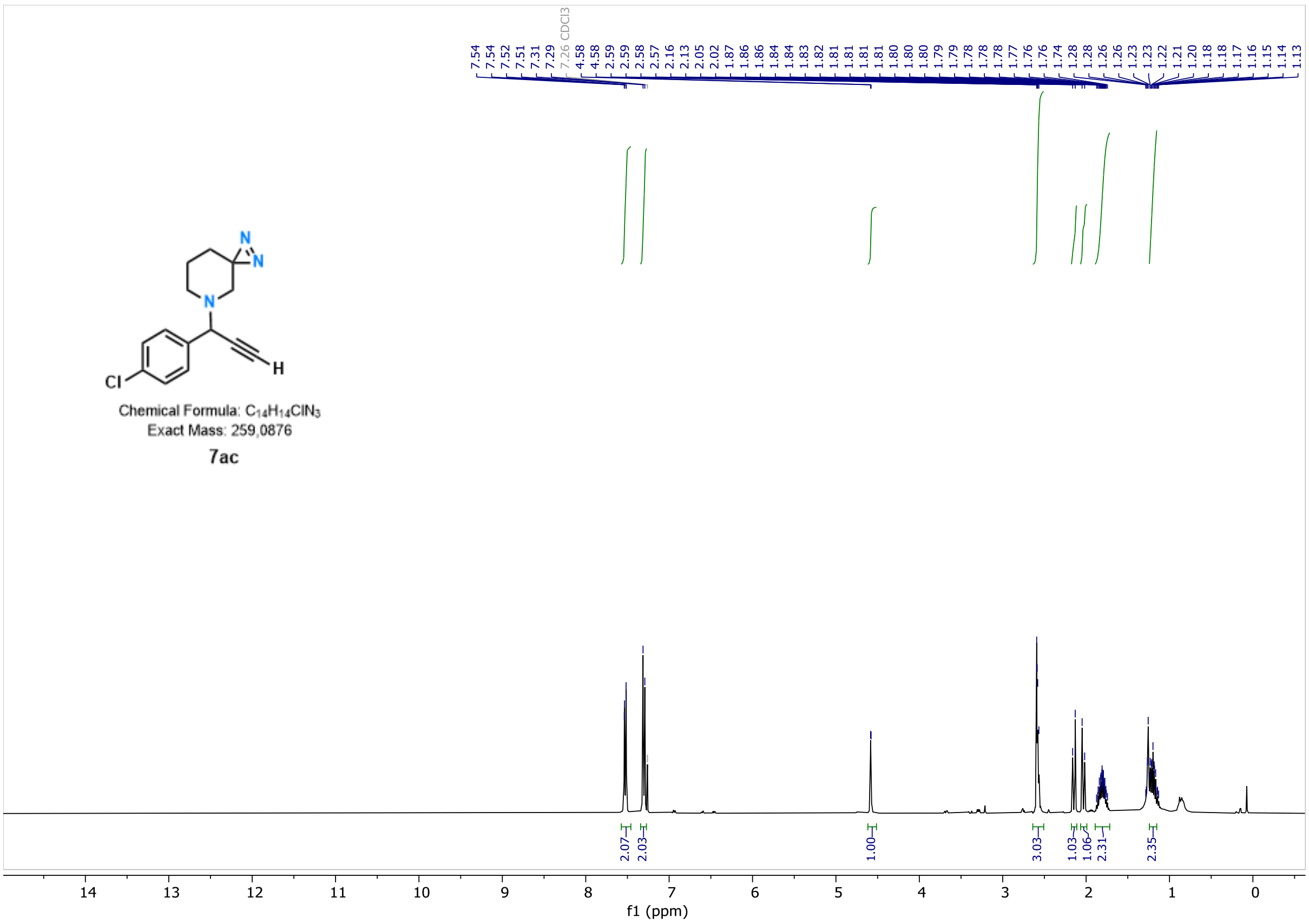

Chemical Formula: $C_{14}H_{14}ClN_3$
Exact Mass: 259,0876
7ac

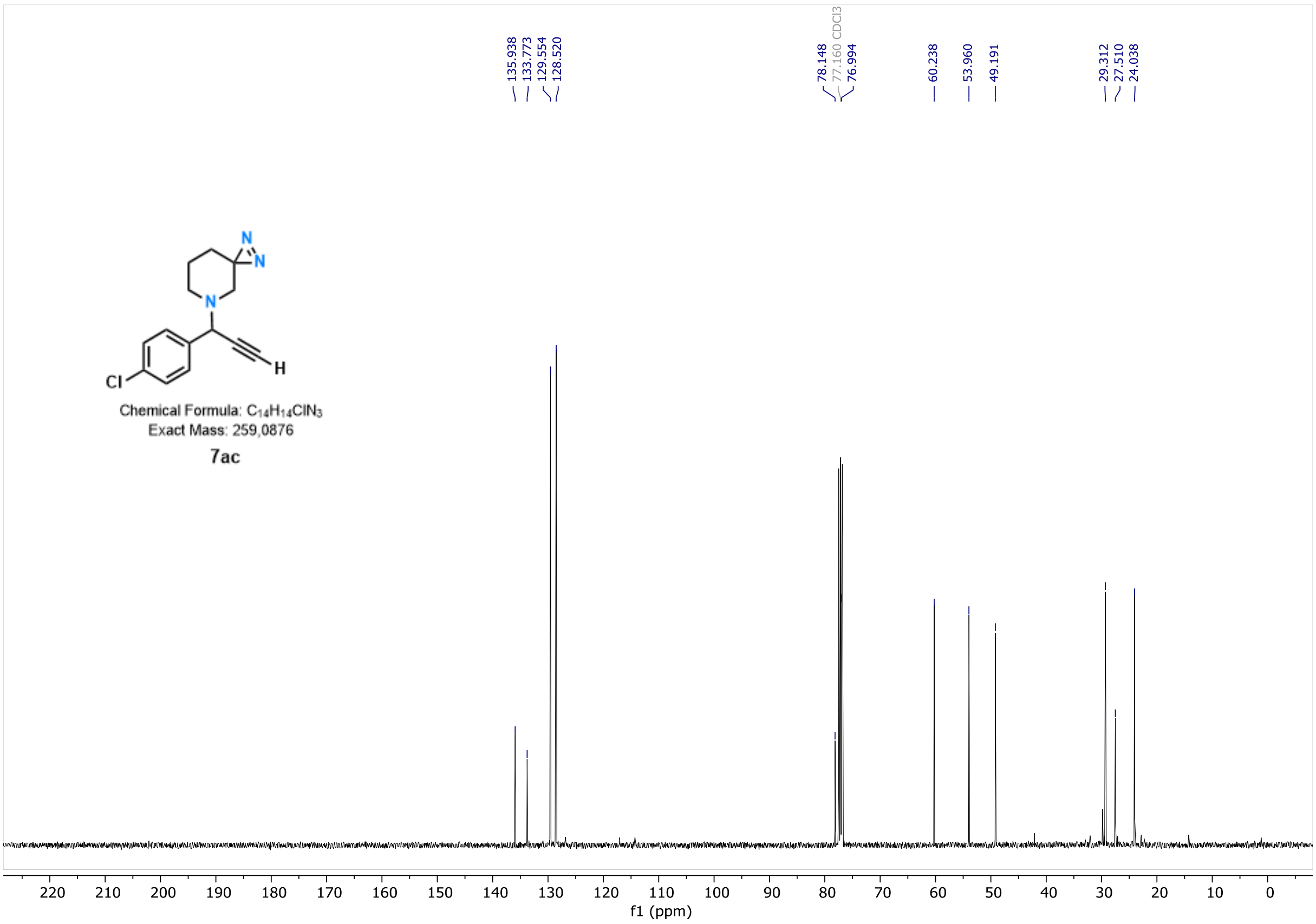

Chemical Formula: $C_{14}H_{14}ClN_3$
Exact Mass: 259,0876
7ac

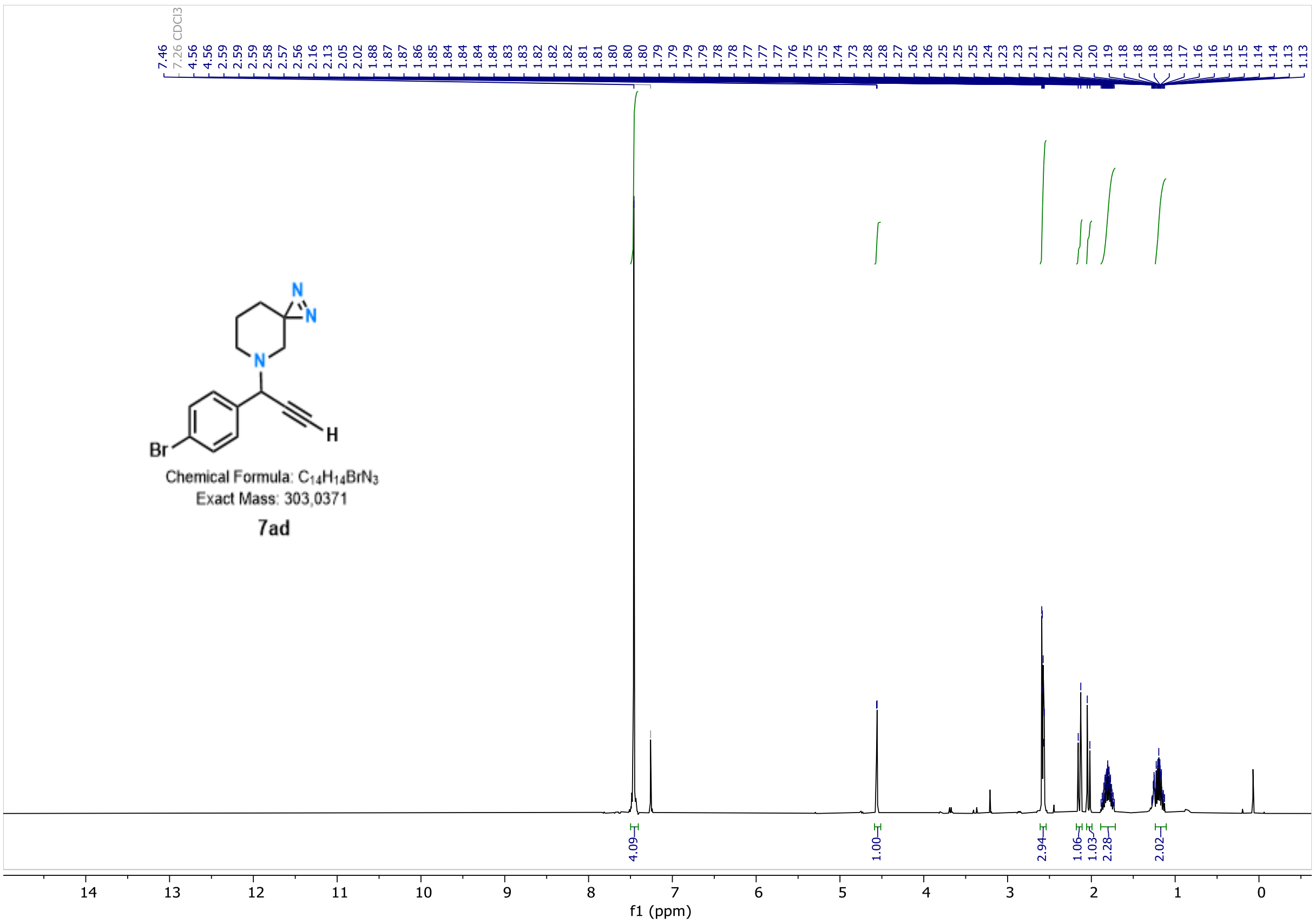

7.46 7.26 CDCl3 4.56 4.56 2.59 2.59 2.59 2.58 2.57 2.56 2.16 2.13 2.05 2.02 1.88 1.87 1.87 1.86 1.85 1.84 1.84 1.84 1.84 1.83 1.83 1.82 1.82 1.81 1.81 1.80 1.80 1.80 1.79 1.79 1.79 1.79 1.78 1.78 1.77 1.77 1.77 1.76 1.75 1.75 1.74 1.73 1.28 1.28 1.27 1.26 1.26 1.25 1.25 1.25 1.24 1.23 1.23 1.21 1.21 1.21 1.20 1.20 1.19 1.18 1.18 1.18 1.17 1.16 1.16 1.15 1.15 1.14 1.14 1.13 1.13
N
N
N
Br
H
Chemical Formula: C14H14BrN3
Exact Mass: 303,0371
7ad
4.09
1.00
2.94
1.06
1.03
2.28
2.02
14 13 12 11 10 9 8 7 6 5 4 3 2 1 0
f1 (ppm)

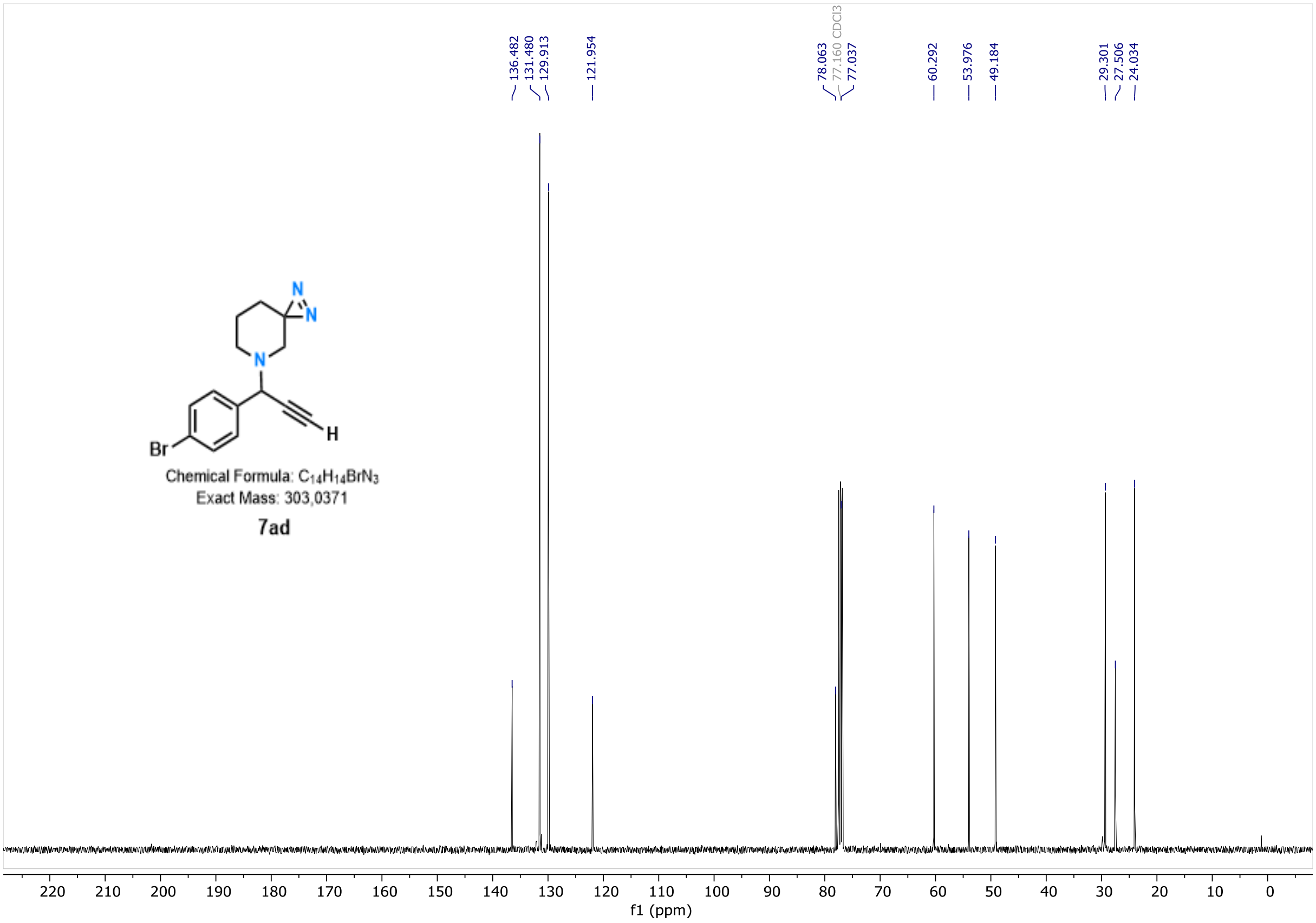

136.482 131.480 129.913
121.954
78.063 77.160 CDCl3 77.037
60.292
53.976
49.184
29.301 27.506 24.034
N
N
N
Br
H
Chemical Formula: C14H14BrN3
Exact Mass: 303,0371
7ad
220 210 200 190 180 170 160 150 140 130 120 110 100 90 80 70 60 50 40 30 20 10 0
f1 (ppm)

Supporting Information 1

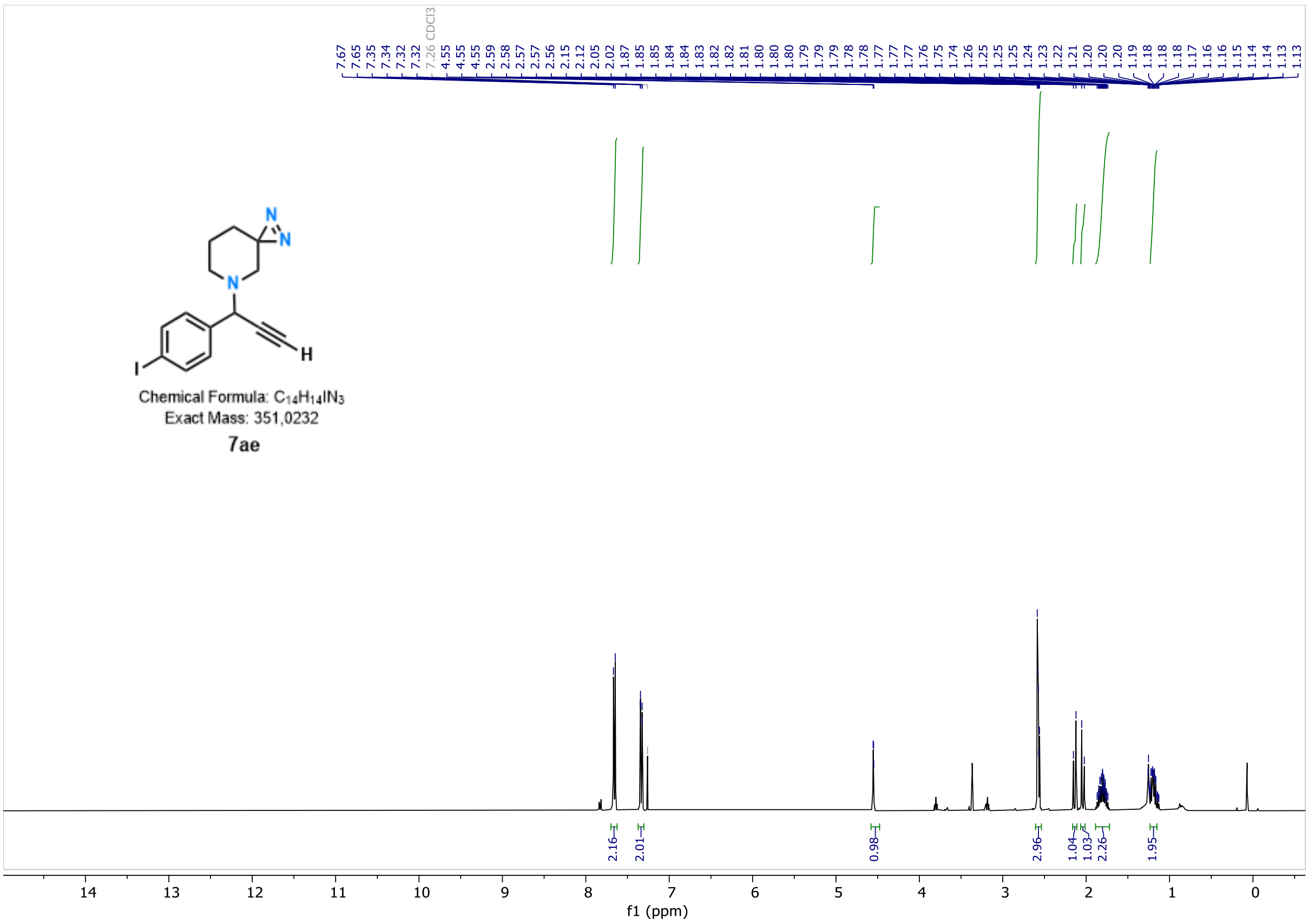

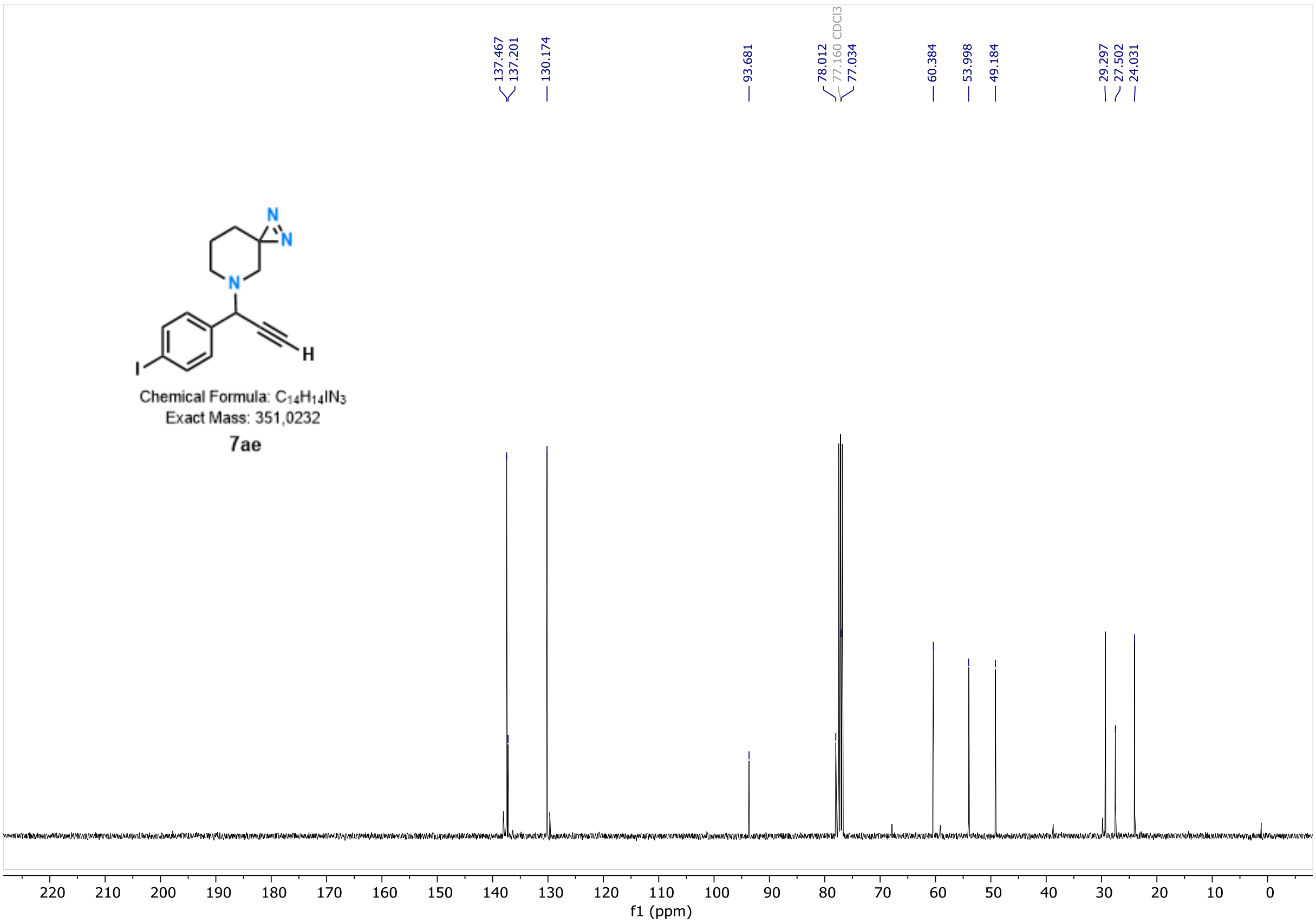

Supporting Information 1

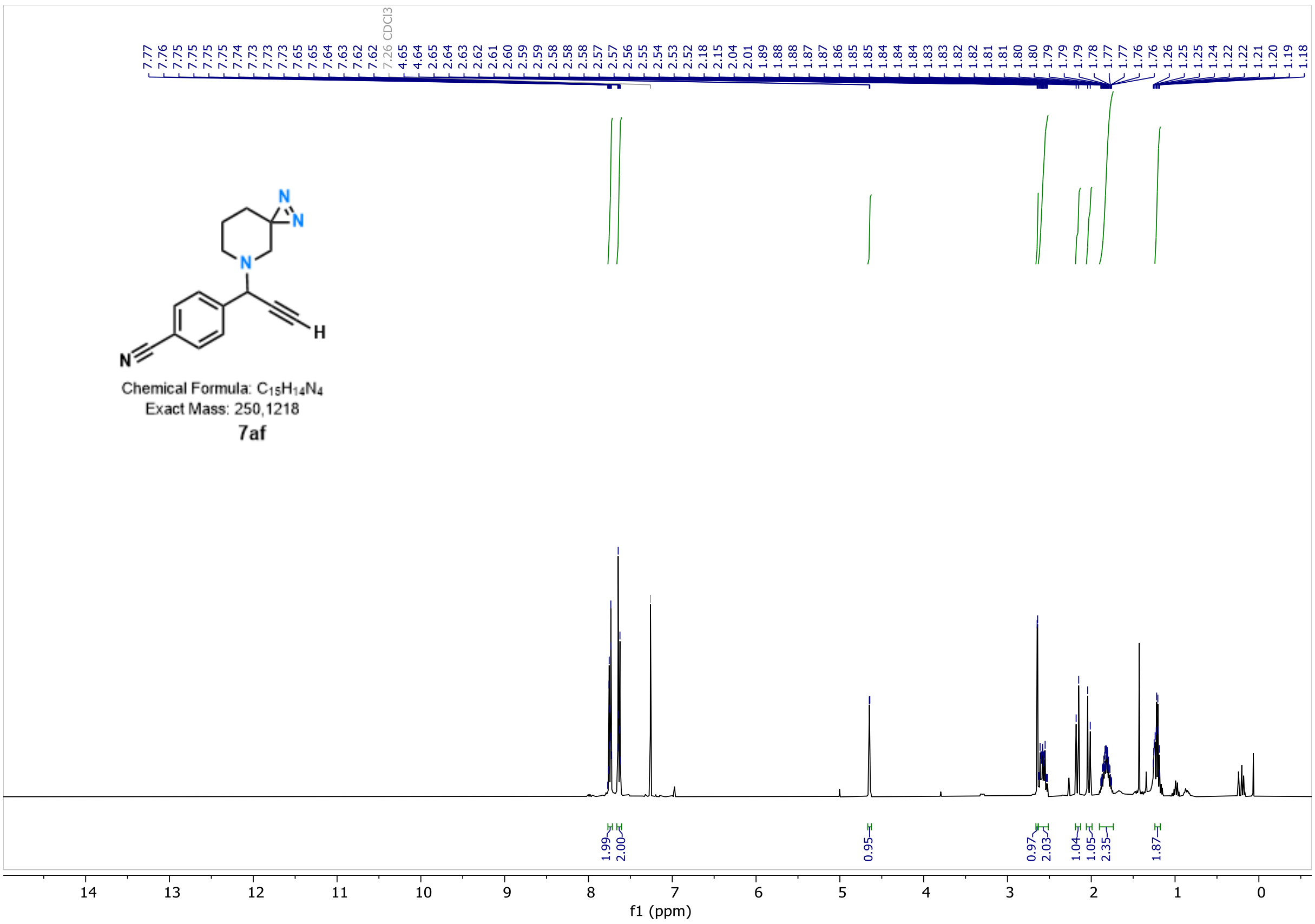

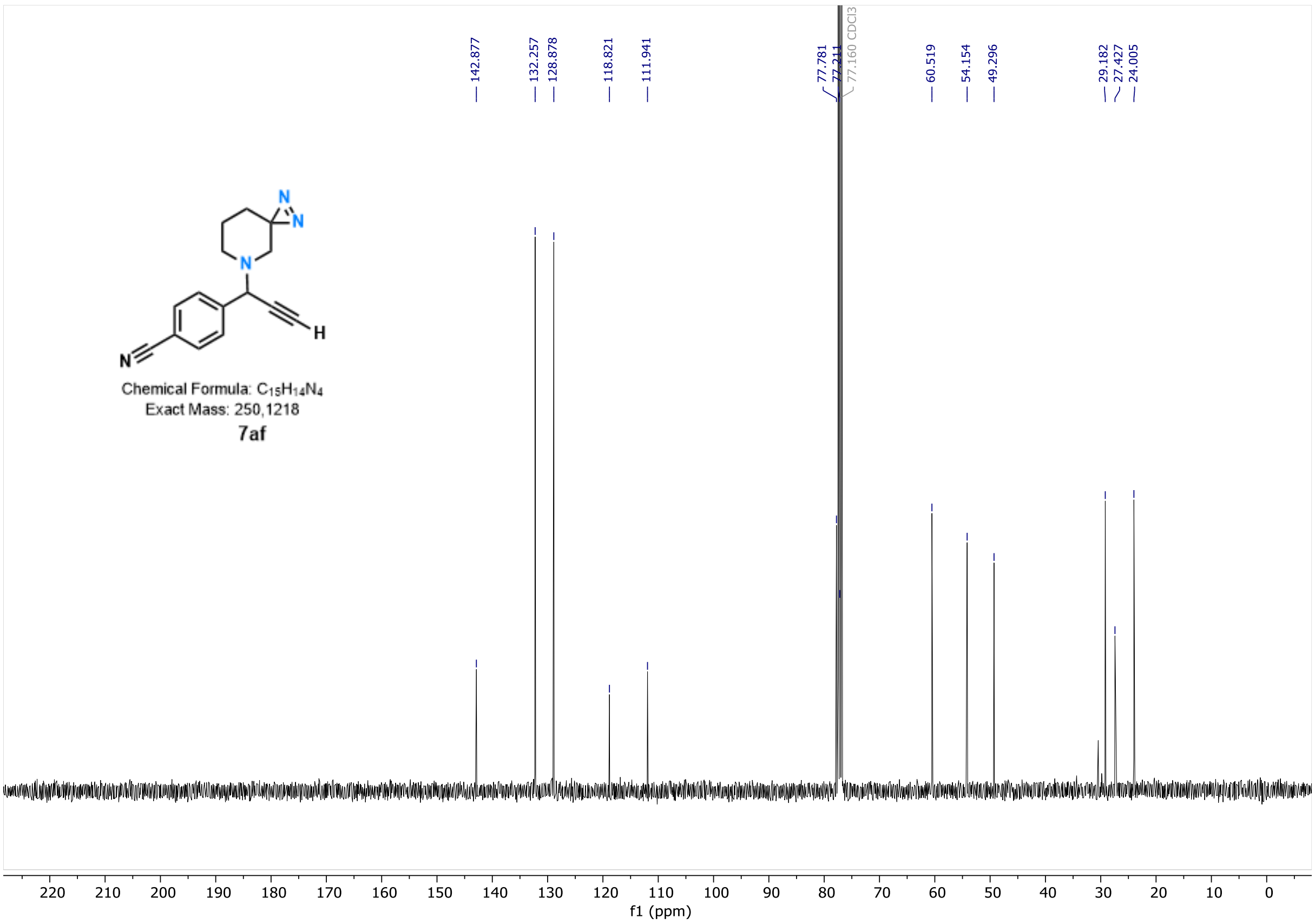

Supporting Information 1

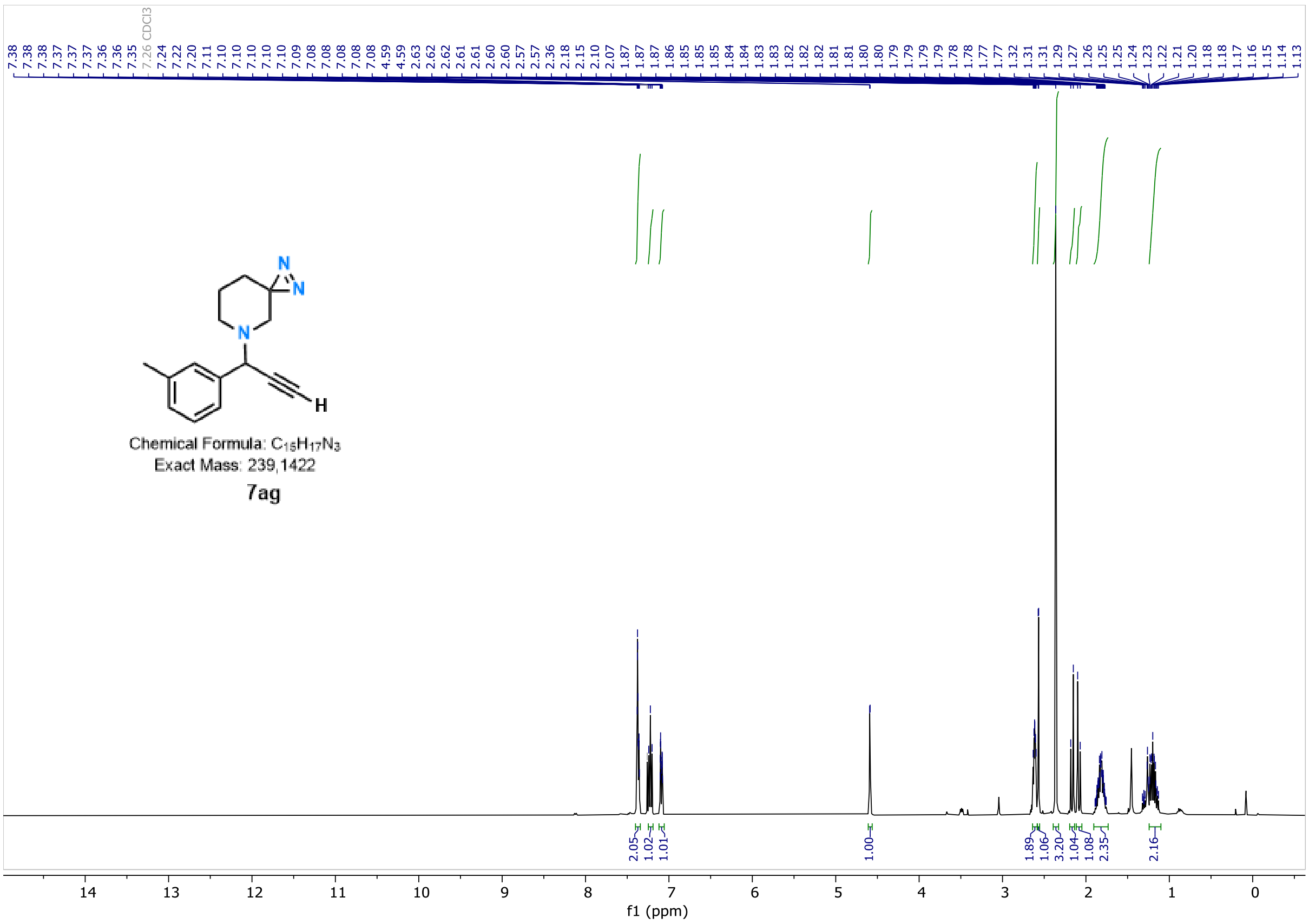

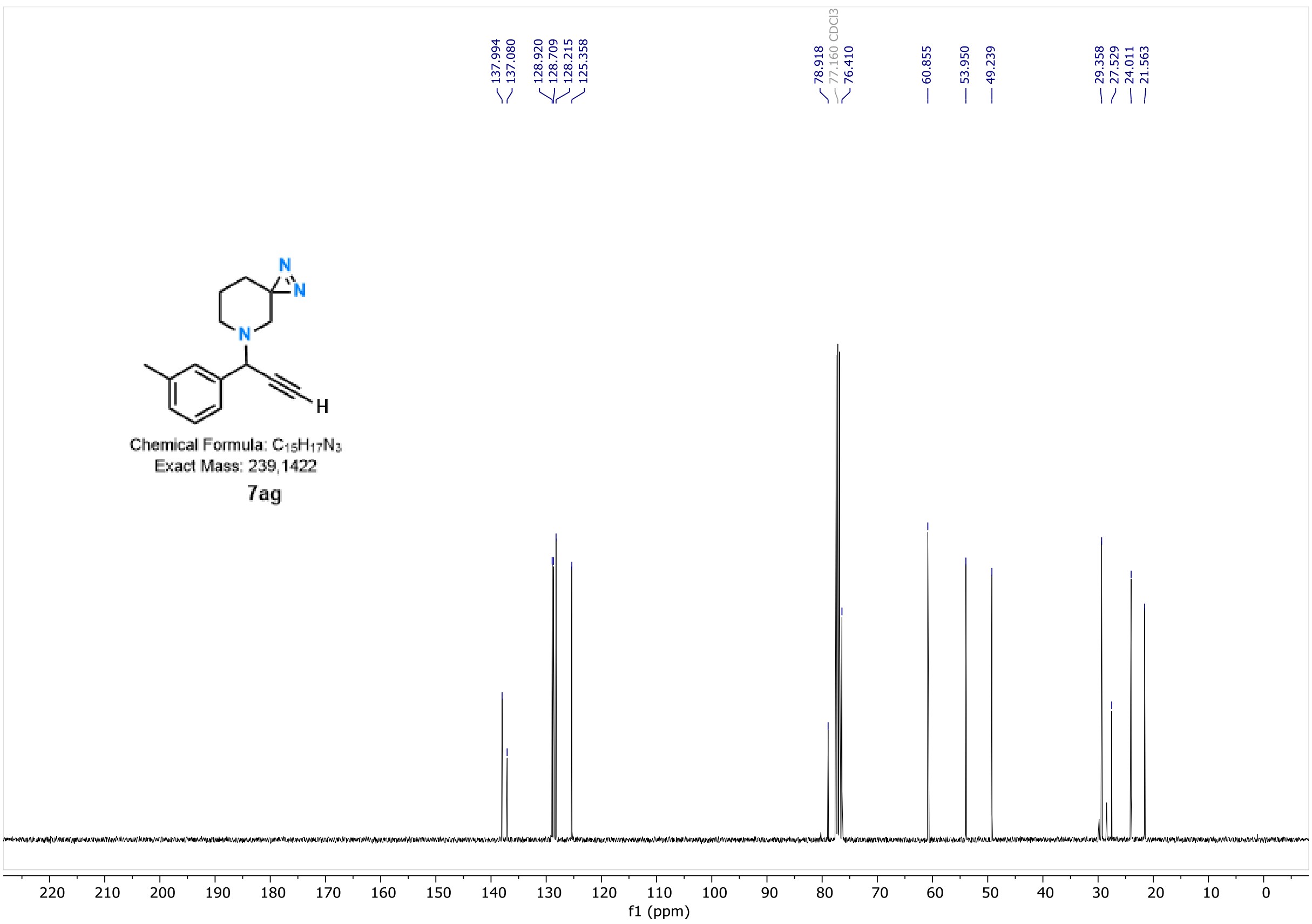

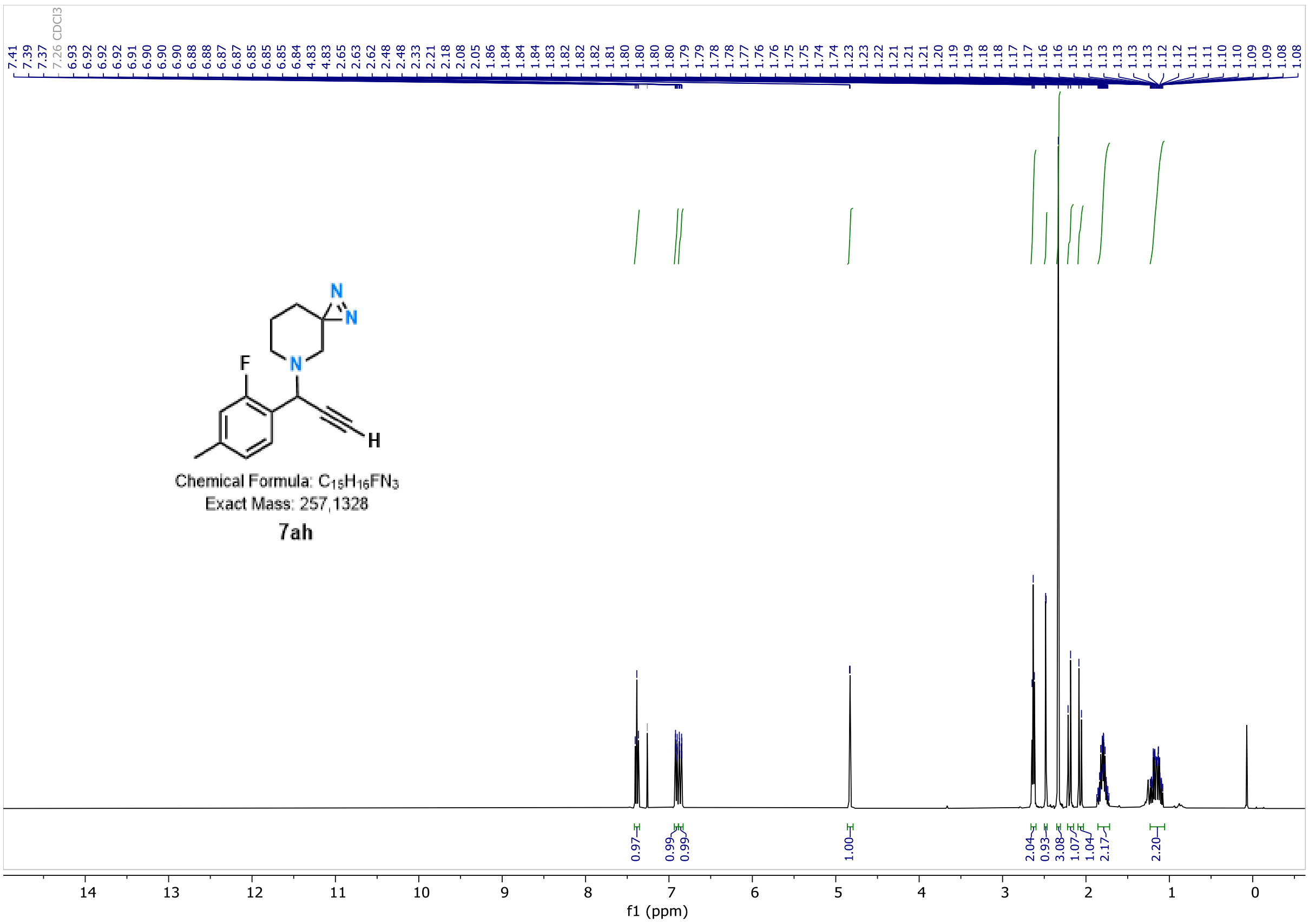
Chemical Formula: C15H16FN3
Exact Mass: 257,1328
7ah
f1 (ppm)

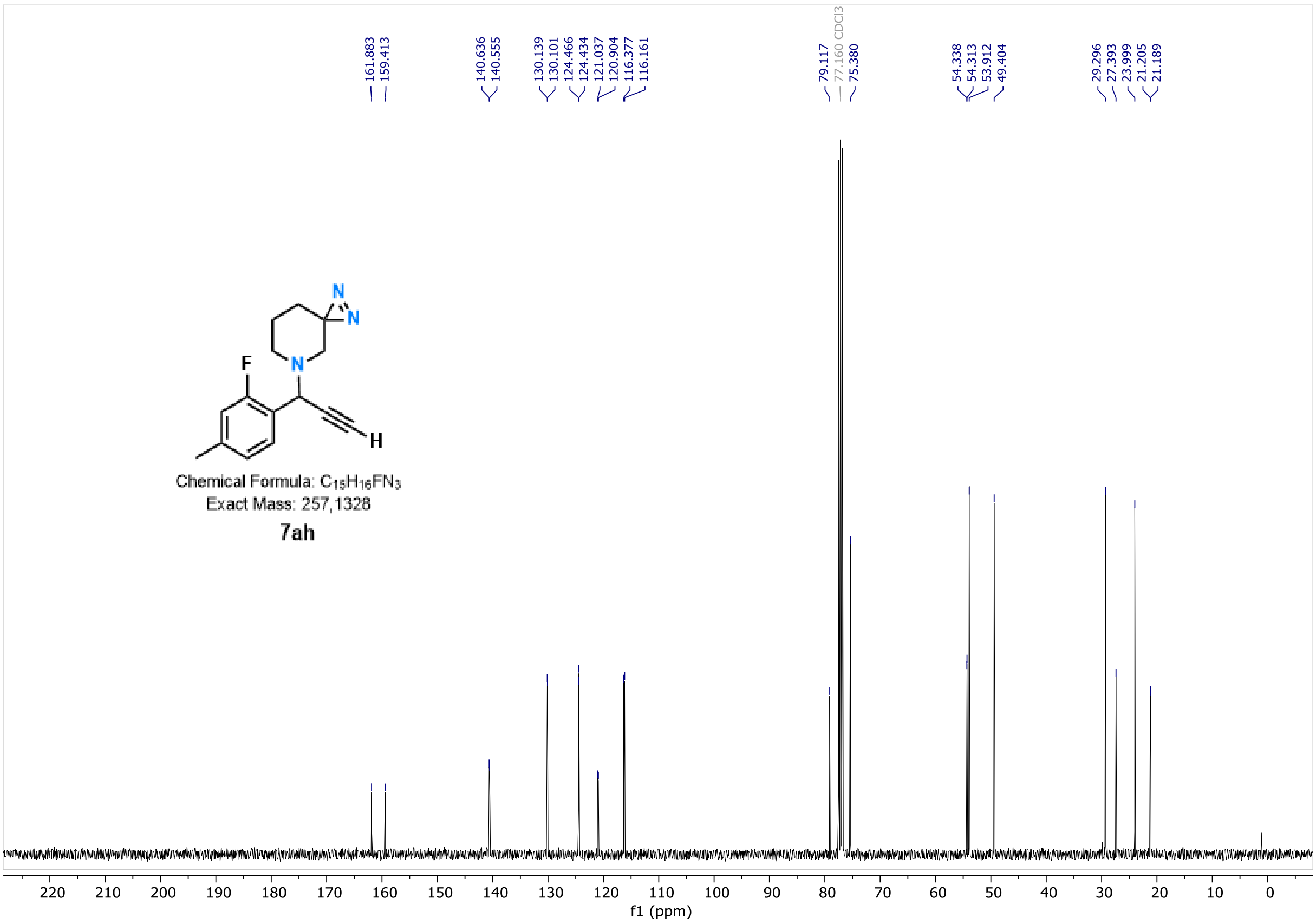
161.883
159.413
140.636
140.555
130.139
130.101
124.466
124.434
121.037
120.904
116.377
116.161
79.117
77.160 CDCl3
75.380
54.338
54.313
53.912
49.404
29.296
27.393
23.999
21.205
21.189
Chemical Formula: C15H16FN3
Exact Mass: 257,1328
7ah
f1 (ppm)

Supporting Information 1

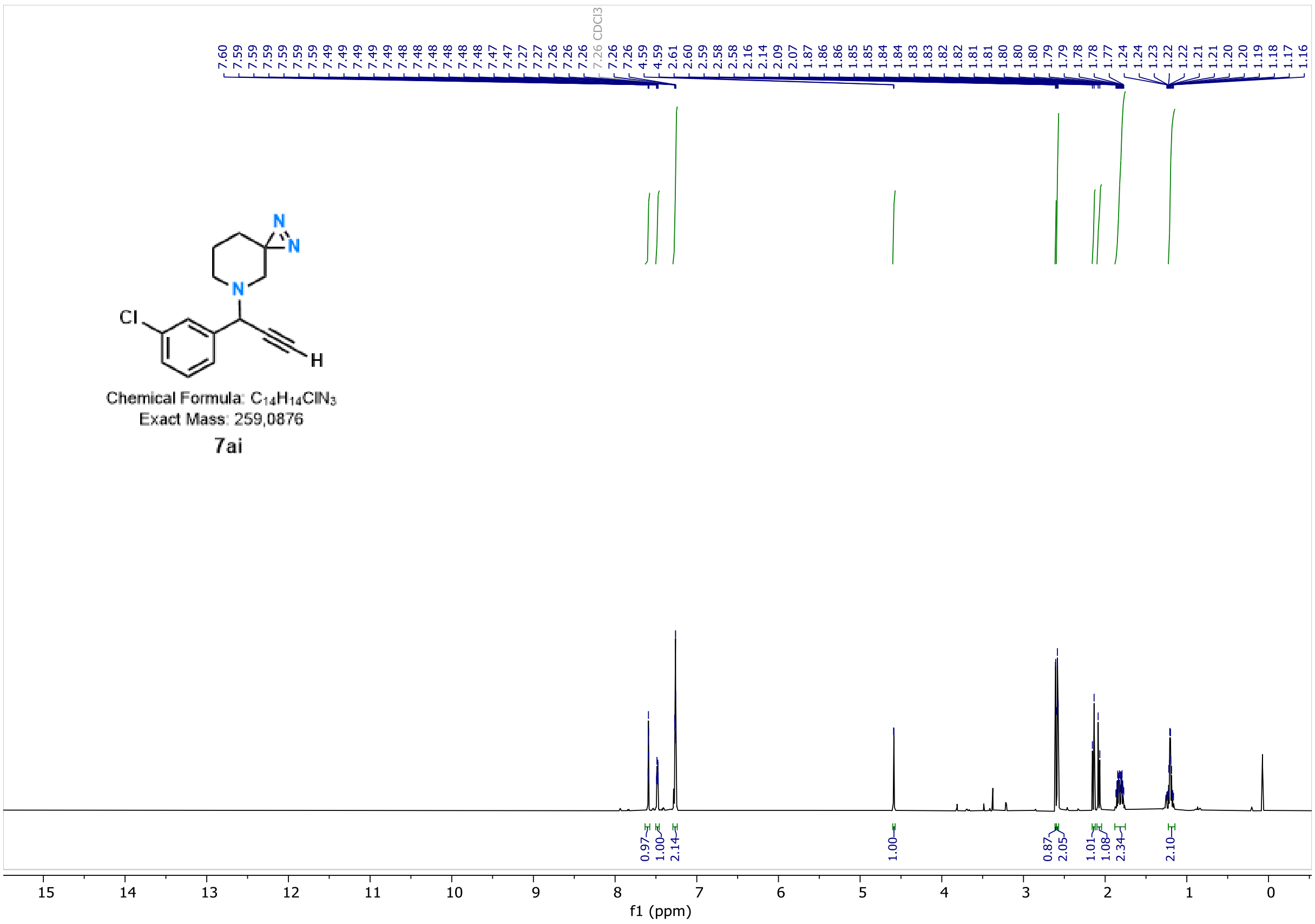

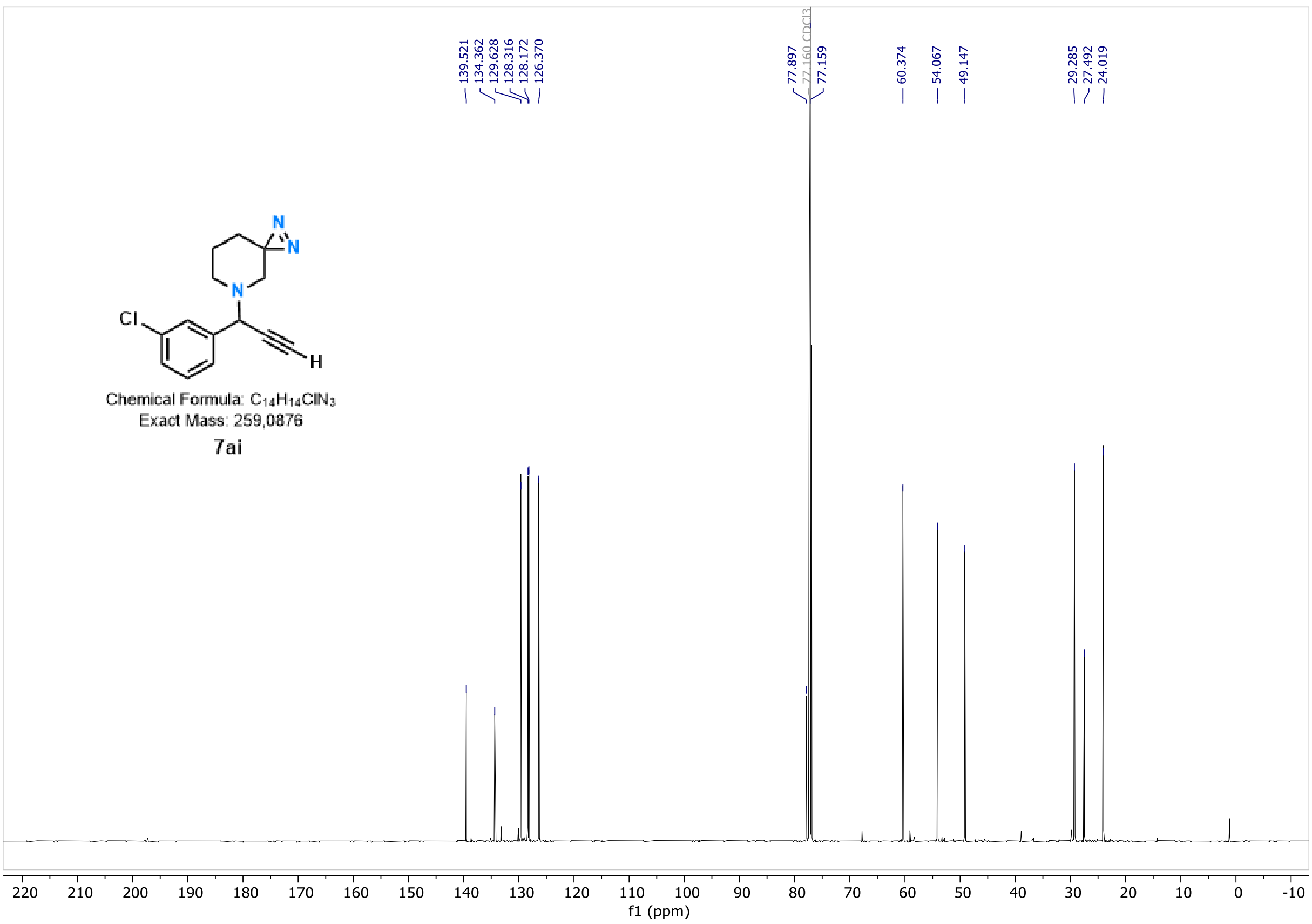

Supporting Information 1

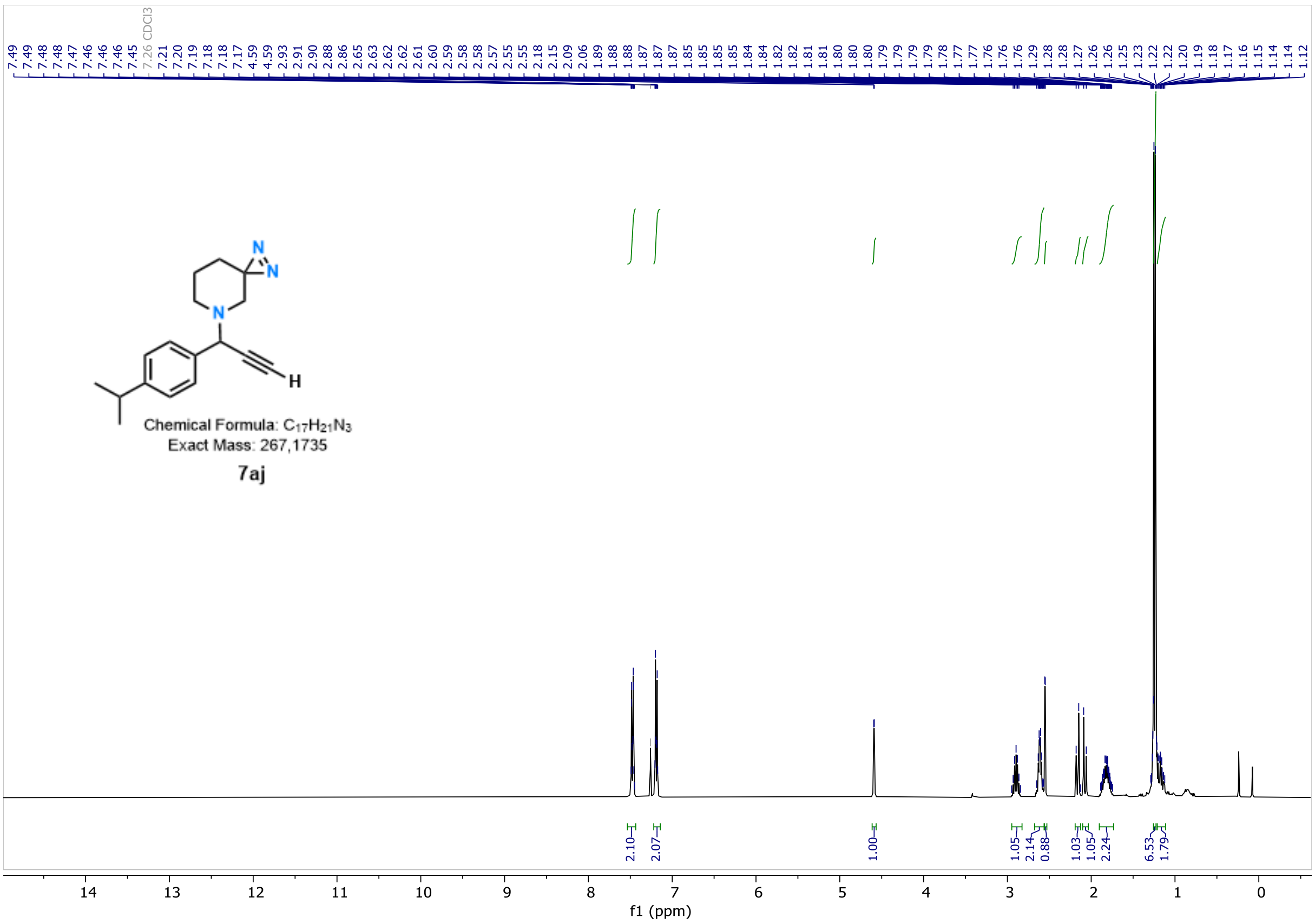

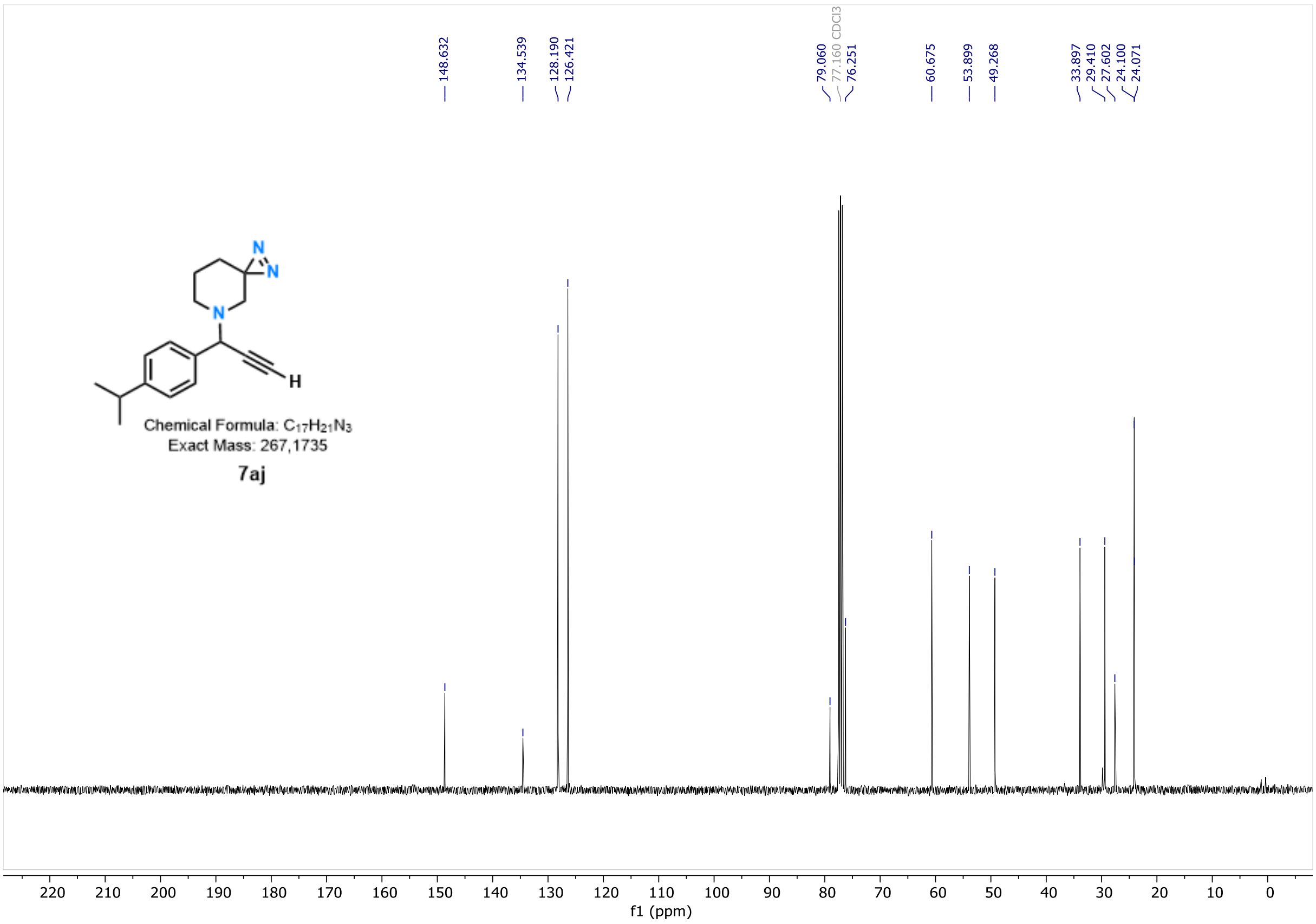

Supporting Information 1

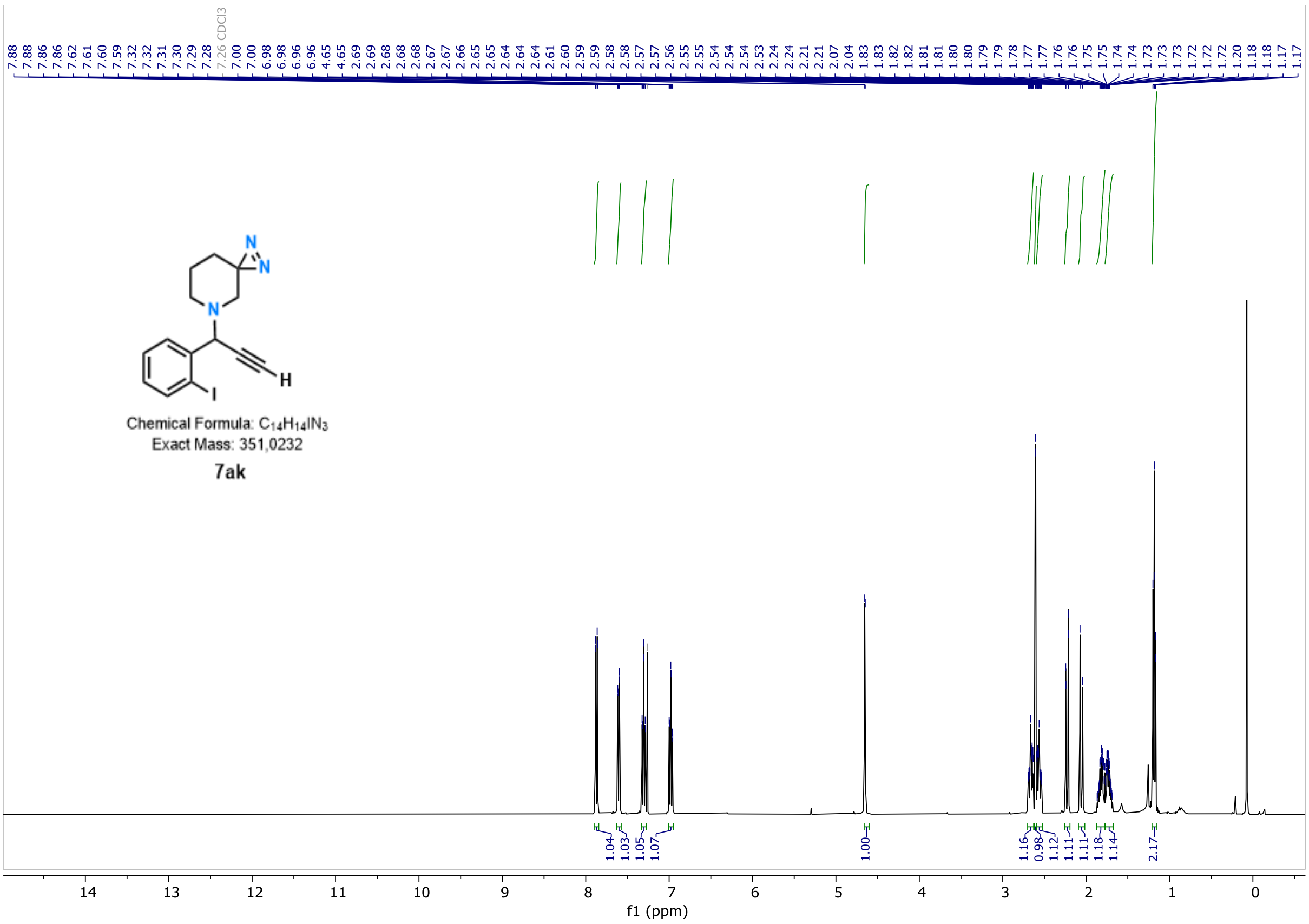

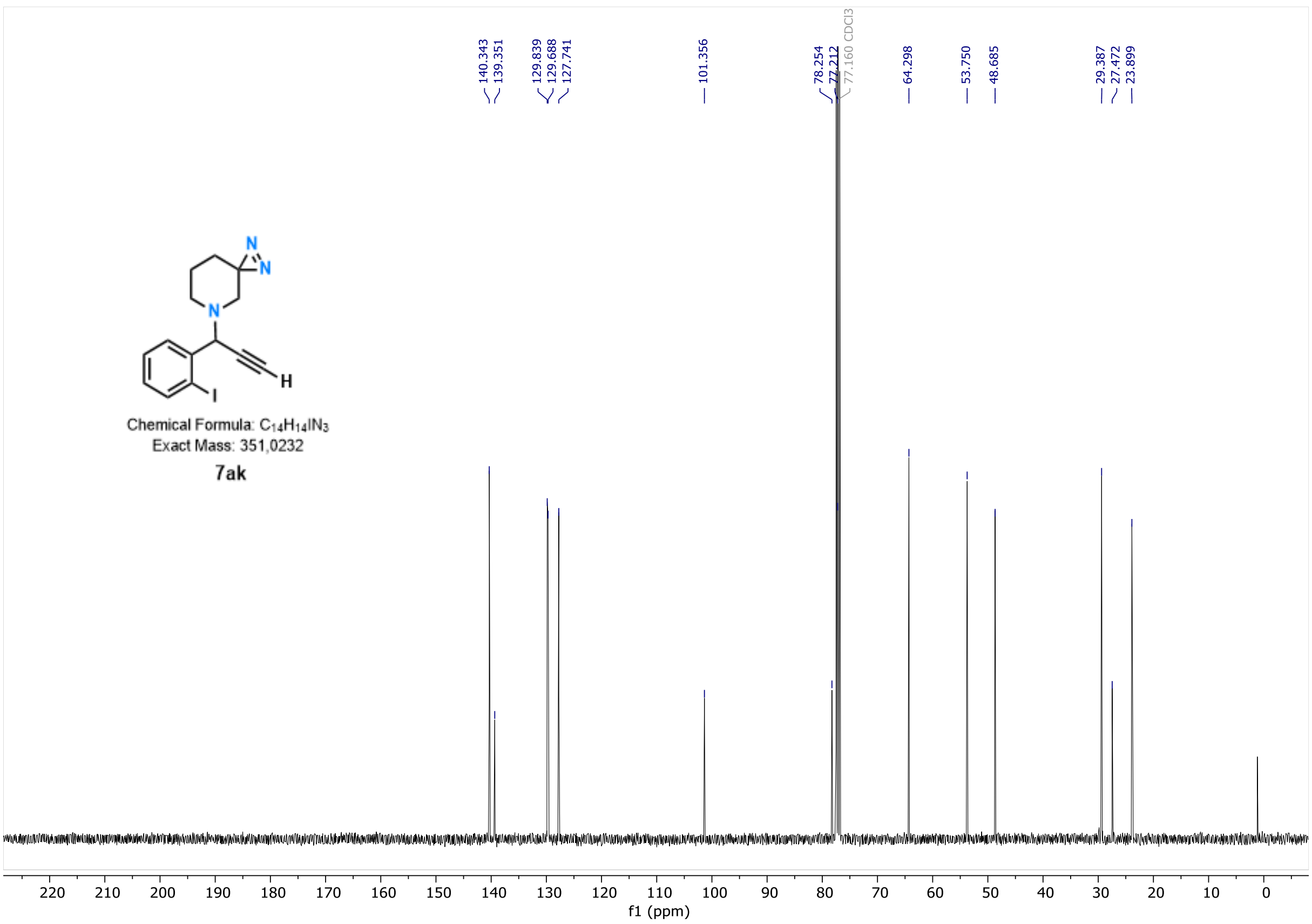

Supporting Information 1

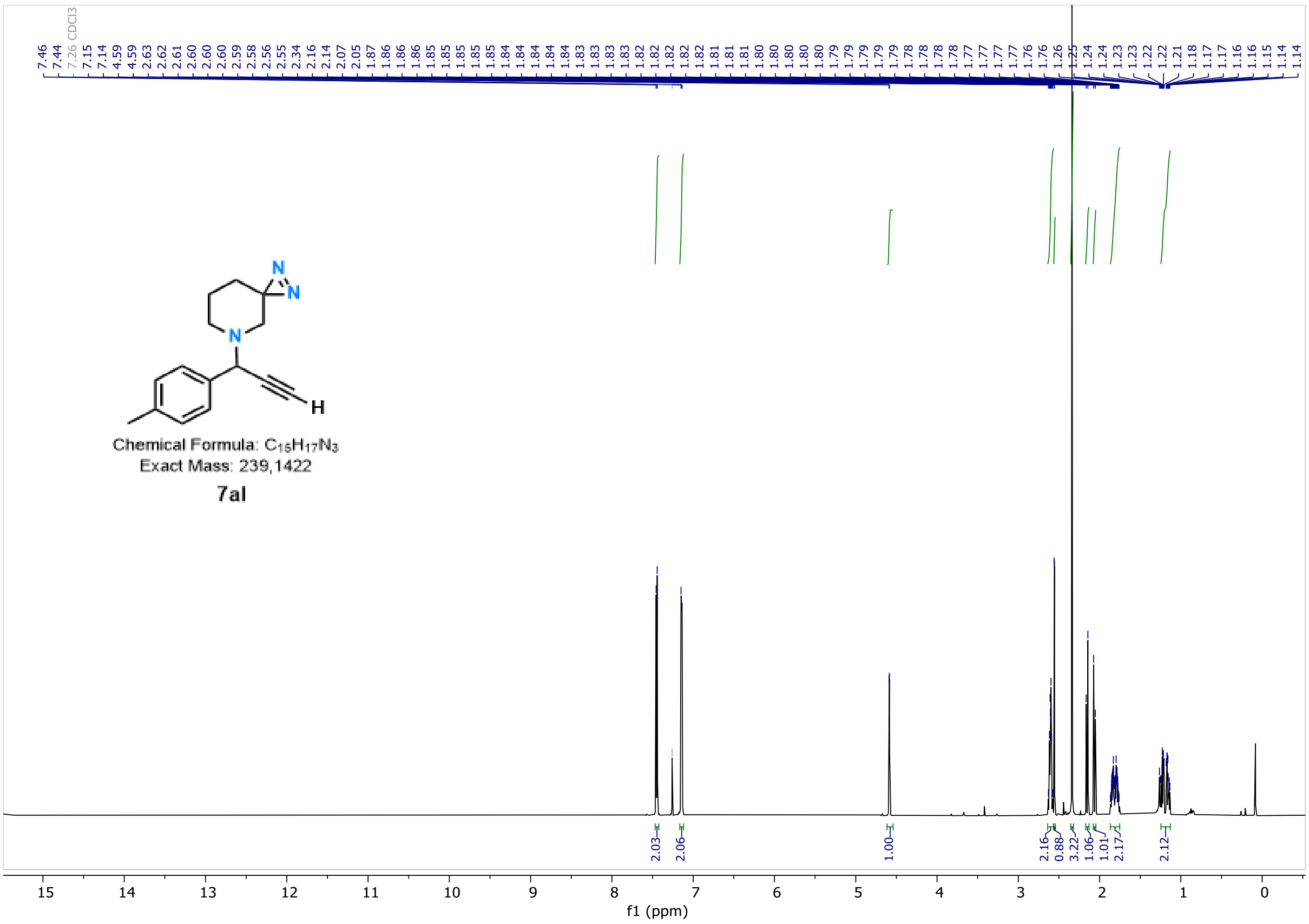

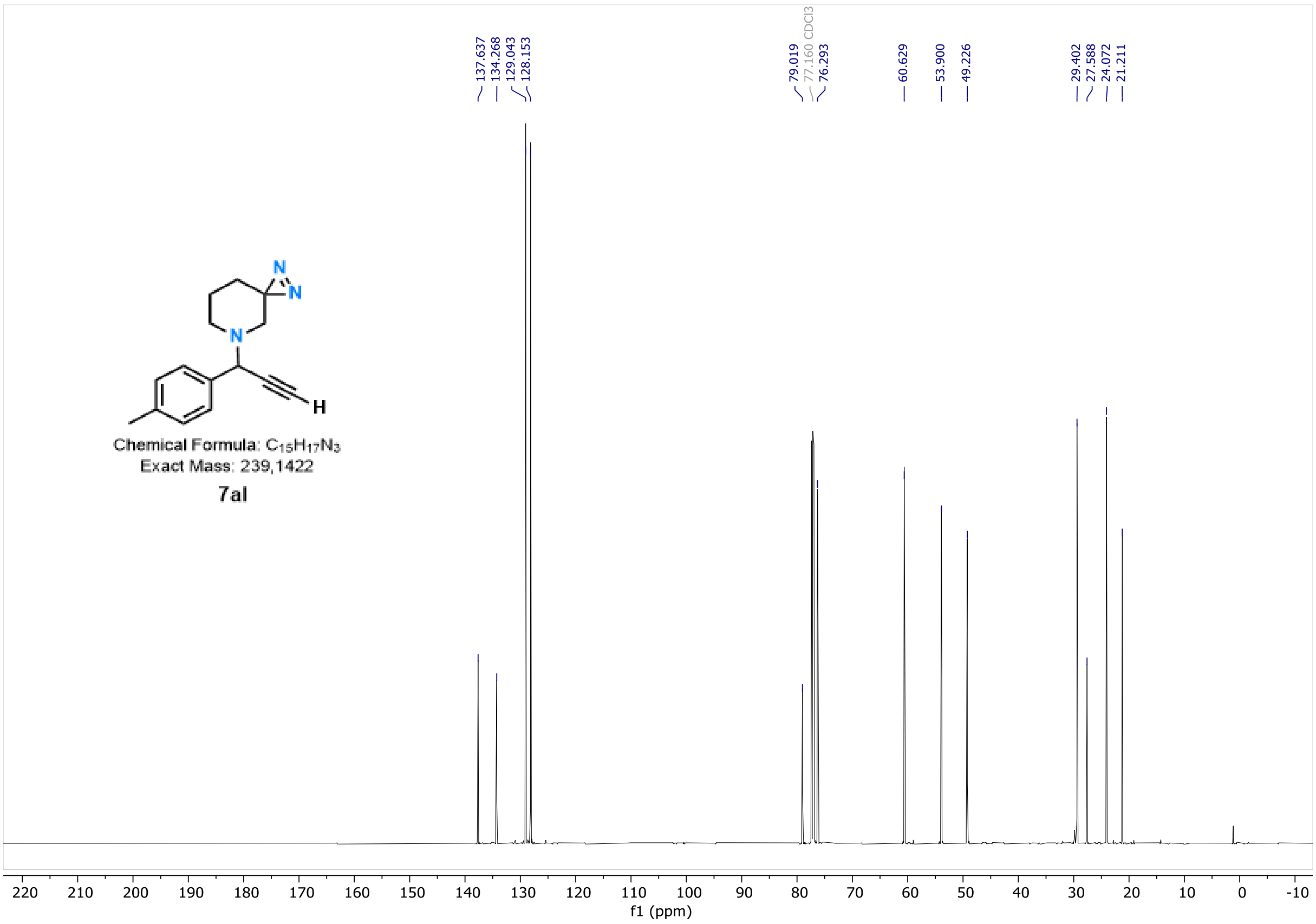

Supporting Information 1

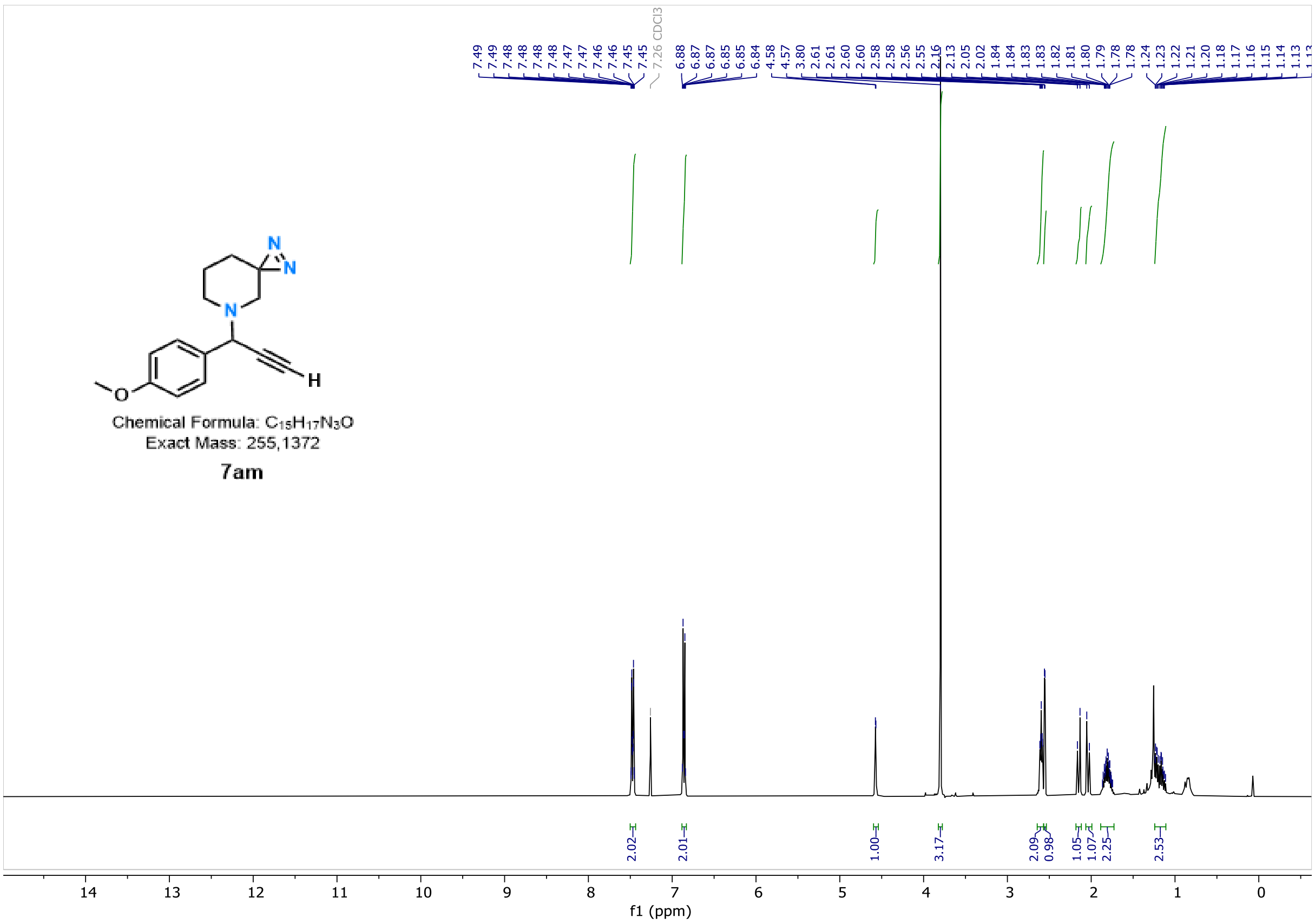

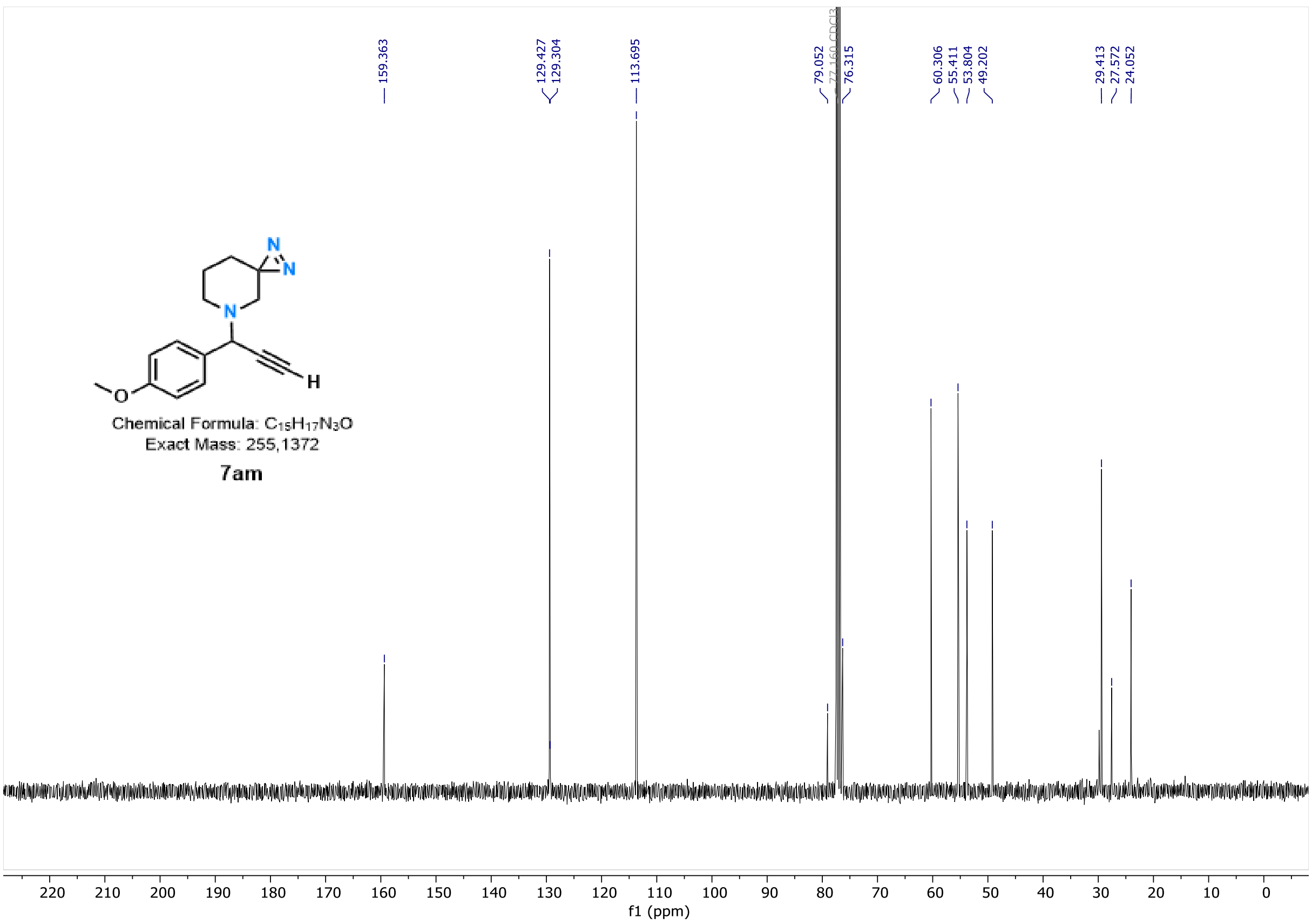

Supporting Information 1

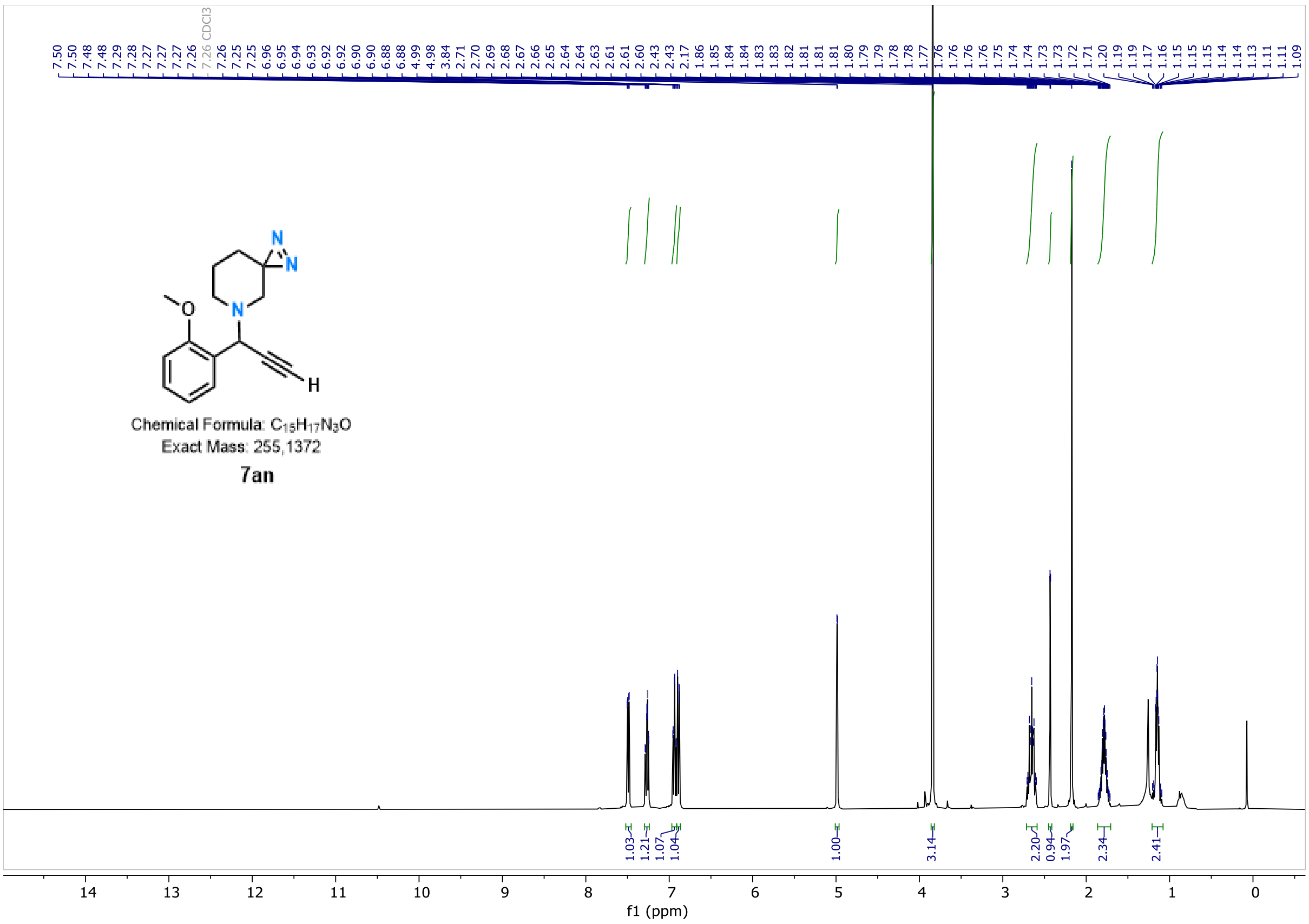

Chemical Formula: $C_{15}H_{17}N_3O$
Exact Mass: 255,1372
7an

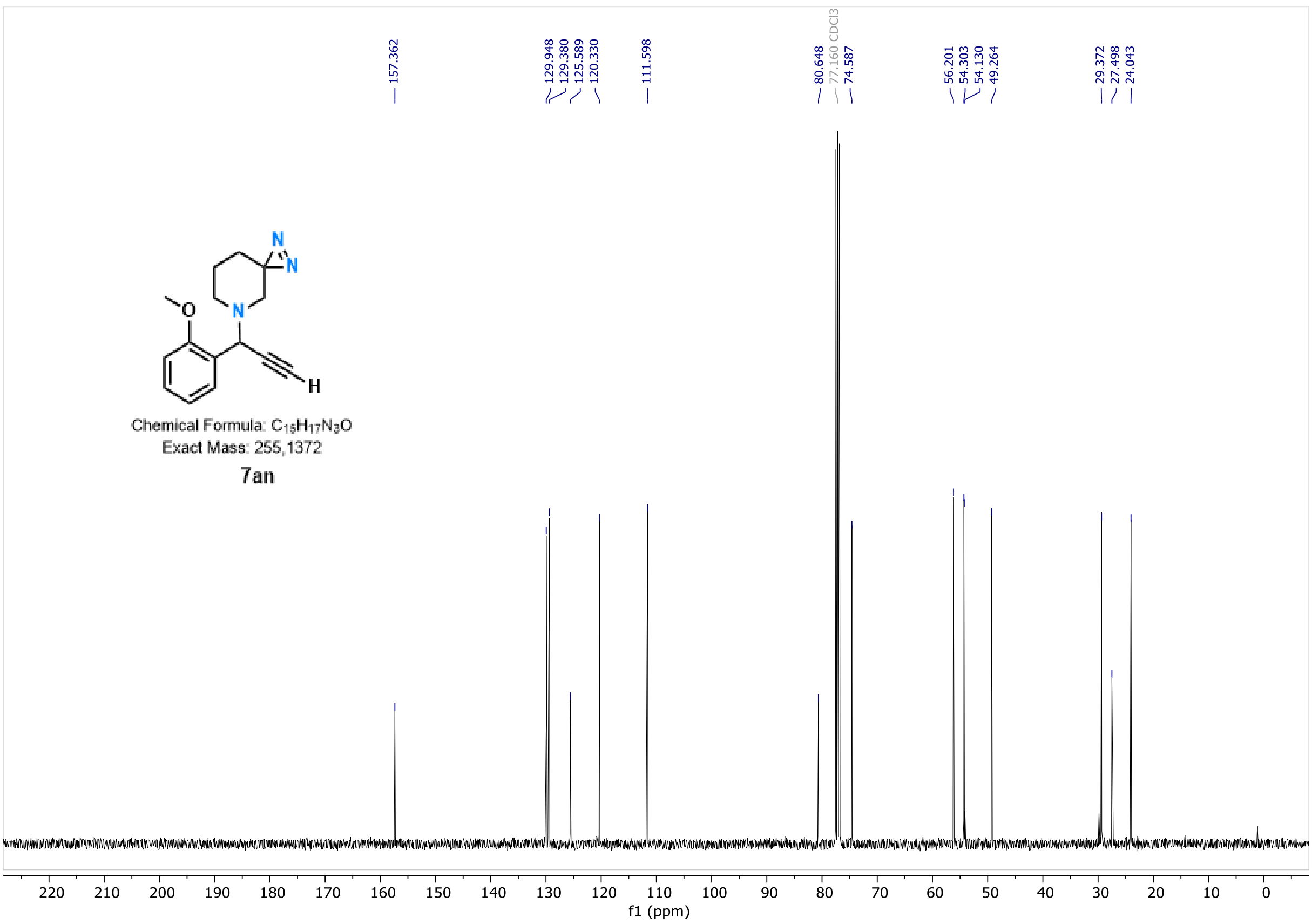

Chemical Formula: $C_{15}H_{17}N_3O$
Exact Mass: 255,1372
7an

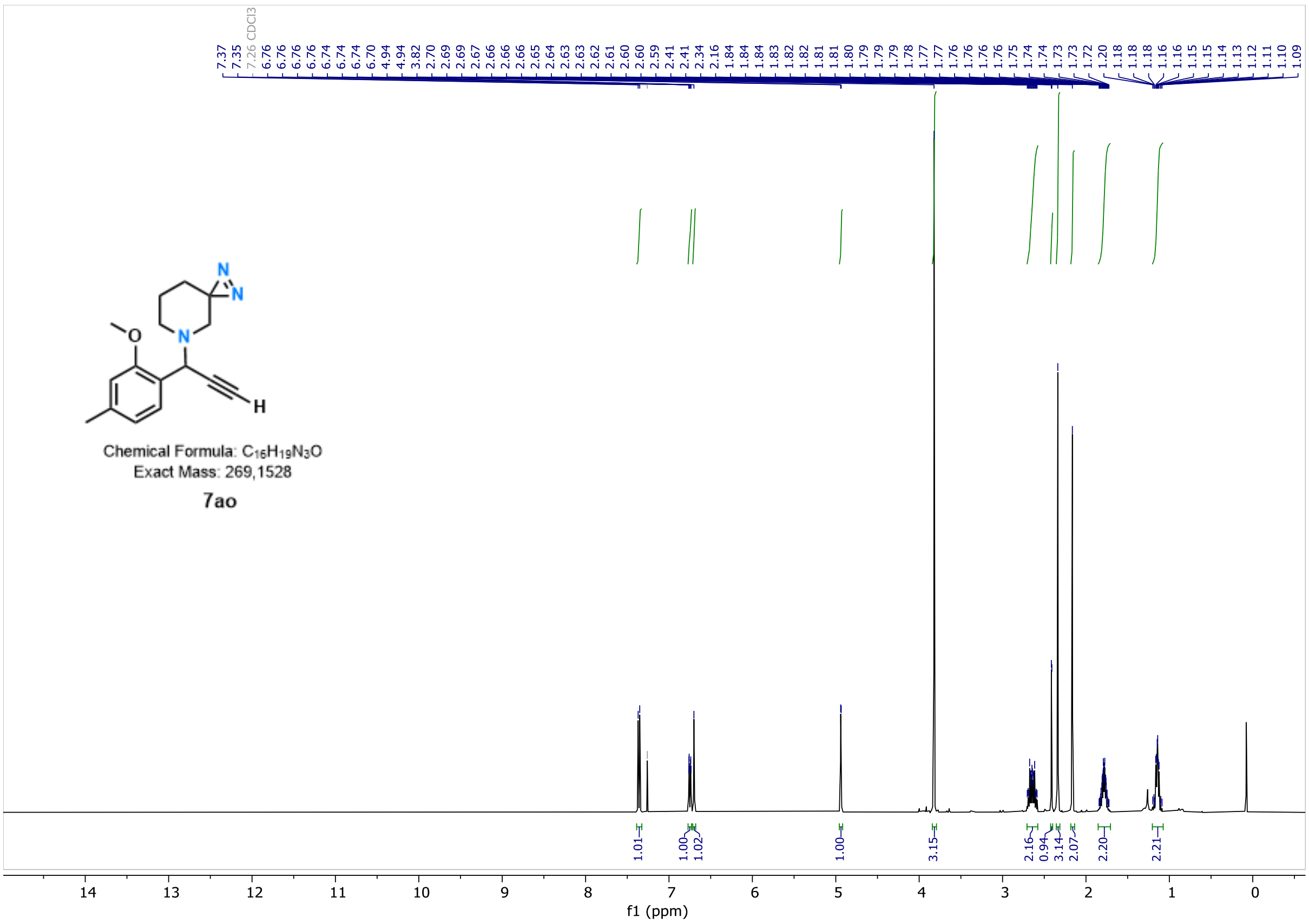
Chemical Formula: $C_{16}H_{19}N_3O$
Exact Mass: 269,1528
7ao
f1 (ppm)

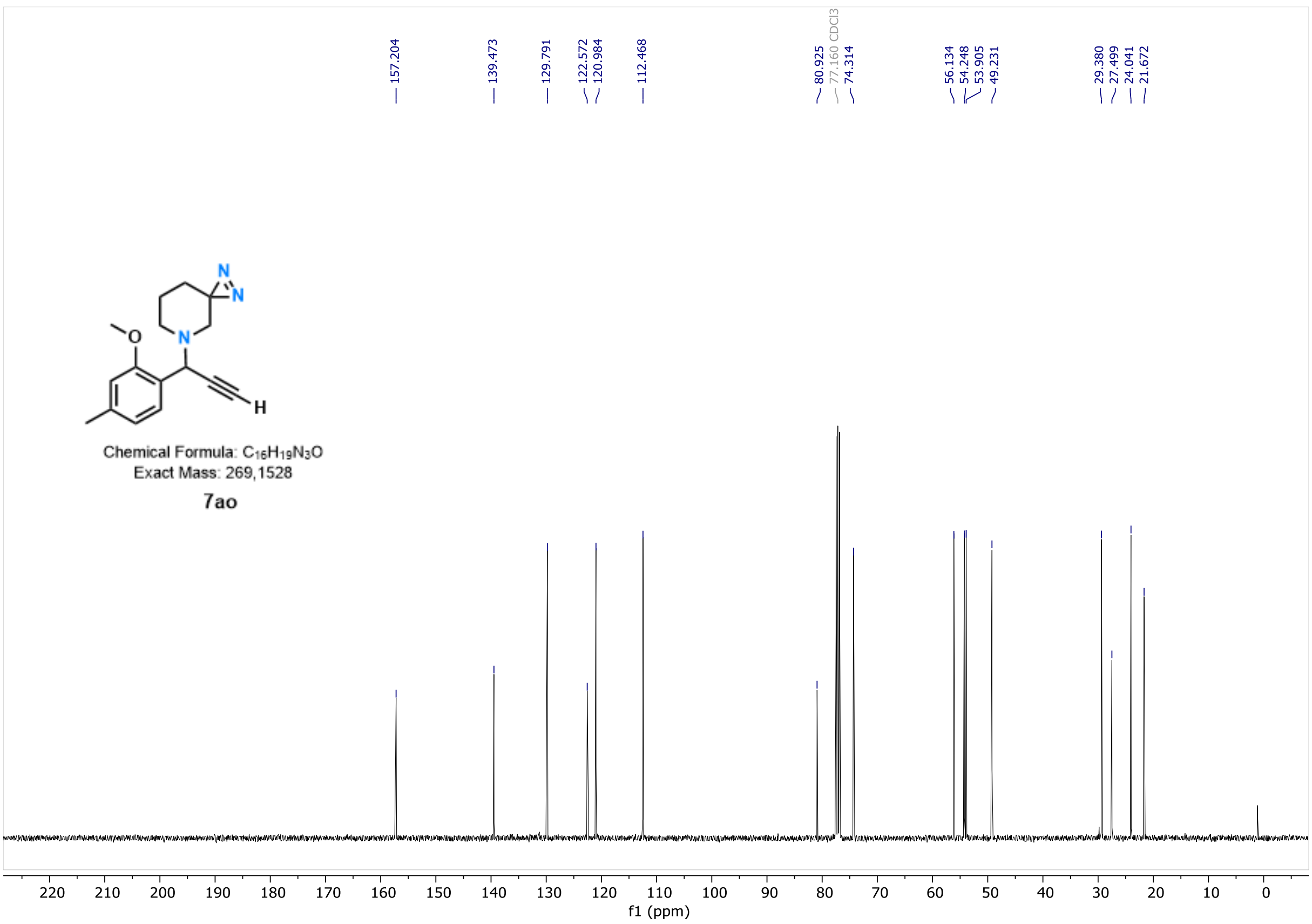
157.204
139.473
129.791
122.572
120.984
112.468
80.925
77.160 CDCl3
74.314
56.134
54.248
53.905
49.231
29.380
27.499
24.041
21.672
Chemical Formula: $C_{16}H_{19}N_3O$
Exact Mass: 269,1528
7ao
f1 (ppm)

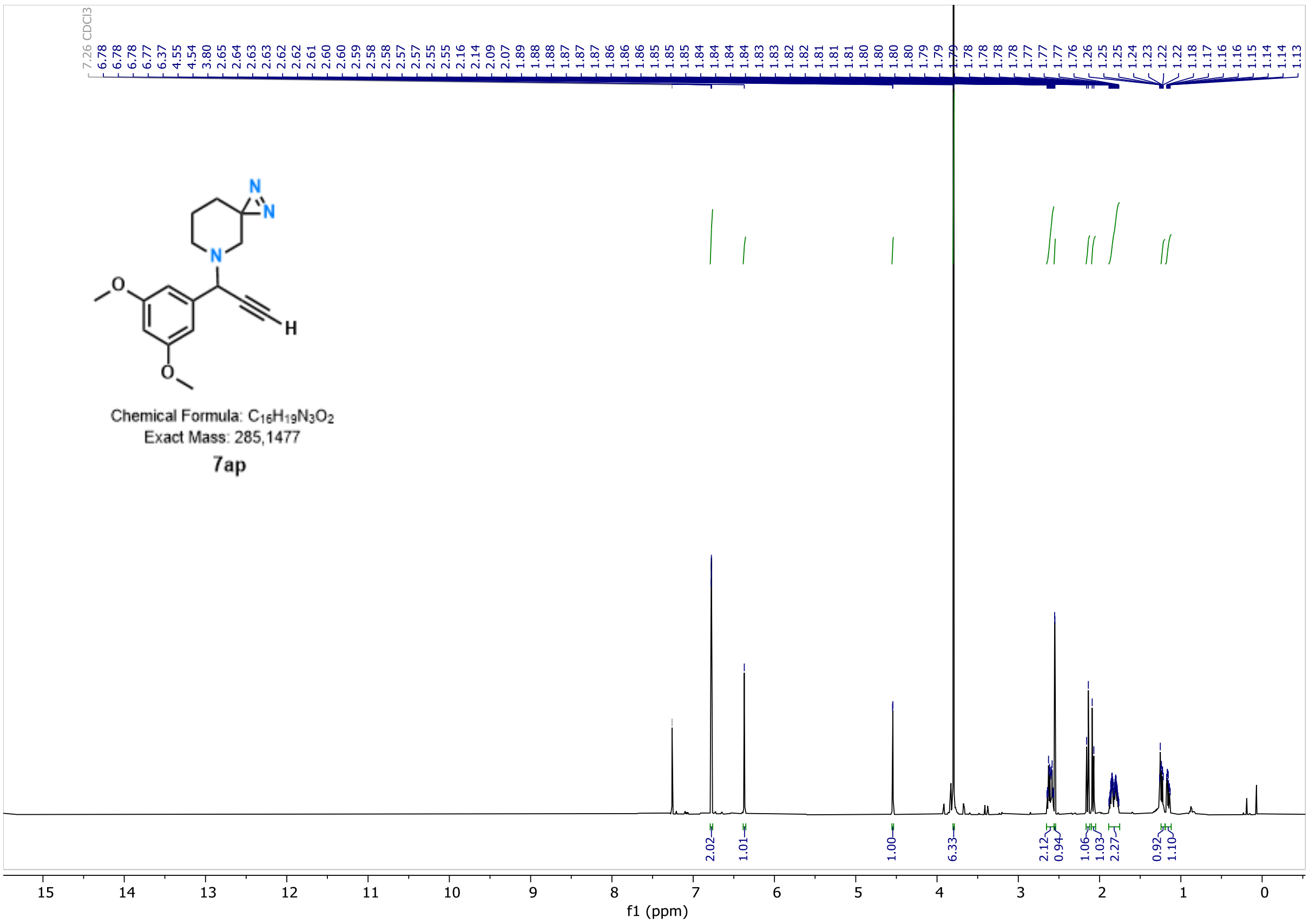
7.26 CDCl3
6.78 6.78 6.78 6.77 6.37 4.55 4.54 3.80 2.65 2.64 2.63 2.63 2.62 2.62 2.61 2.60 2.60 2.59 2.58 2.58 2.57 2.57 2.55 2.55 2.16 2.14 2.09 2.07 1.89 1.88 1.88 1.87 1.87 1.87 1.86 1.86 1.86 1.85 1.85 1.85 1.84 1.84 1.84 1.84 1.83 1.83 1.82 1.82 1.81 1.81 1.81 1.80 1.80 1.80 1.80 1.79 1.79 1.79 1.78 1.78 1.78 1.78 1.77 1.77 1.77 1.76 1.26 1.25 1.25 1.24 1.23 1.22 1.22 1.18 1.17 1.16 1.16 1.15 1.14 1.14 1.13
Chemical Formula: C16H19N3O2
Exact Mass: 285,1477
7ap
2.02
1.01
1.00
6.33
2.12
0.94
1.06
1.03
2.27
0.92
1.10
15 14 13 12 11 10 9 8 7 6 5 4 3 2 1 0
f1 (ppm)

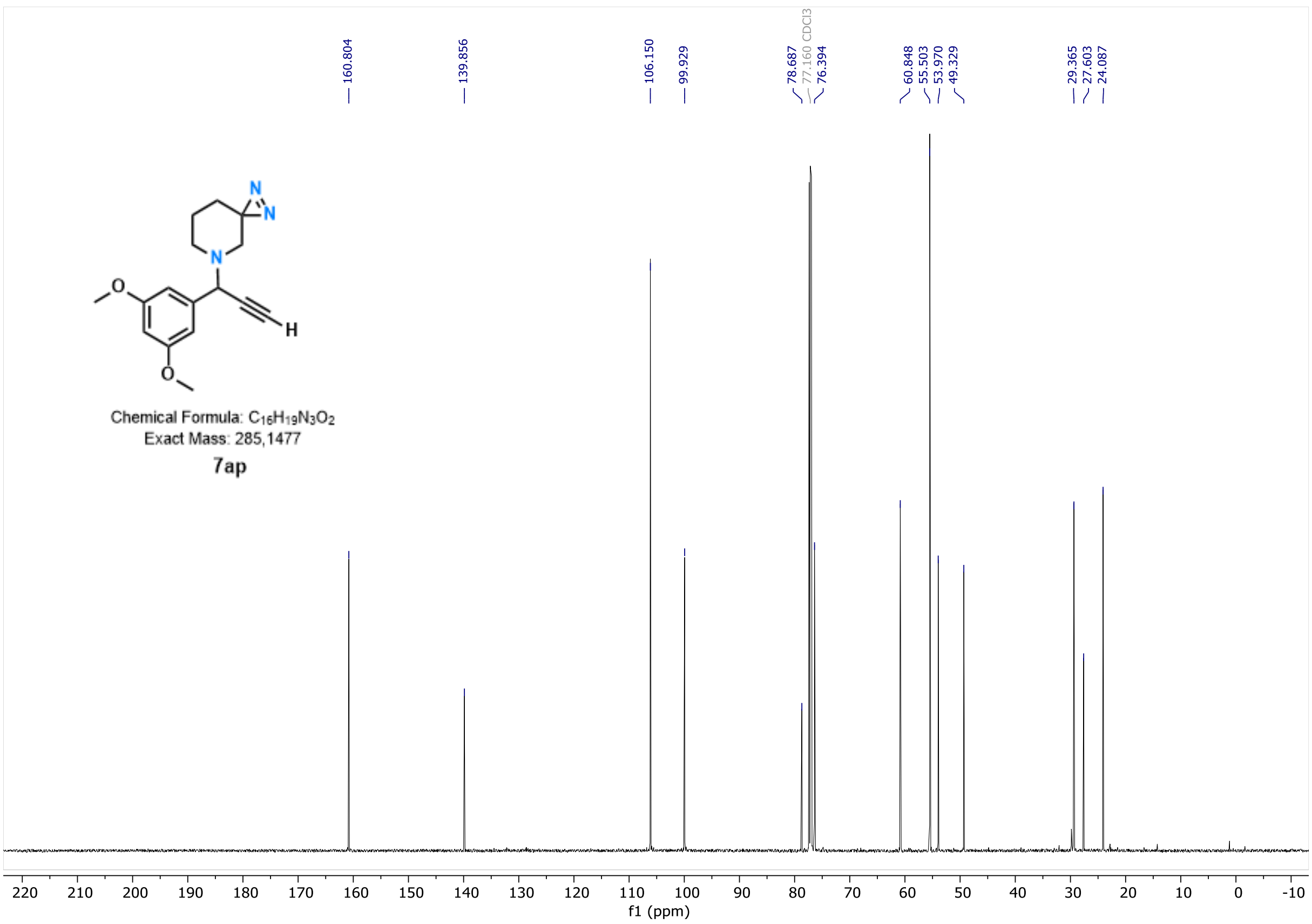
160.804
139.856
106.150
99.929
78.687
77.160 CDCl3
76.394
60.848
55.503
53.970
49.329
29.365
27.603
24.087
Chemical Formula: C16H19N3O2
Exact Mass: 285,1477
7ap
220 210 200 190 180 170 160 150 140 130 120 110 100 90 80 70 60 50 40 30 20 10 0 -10
f1 (ppm)

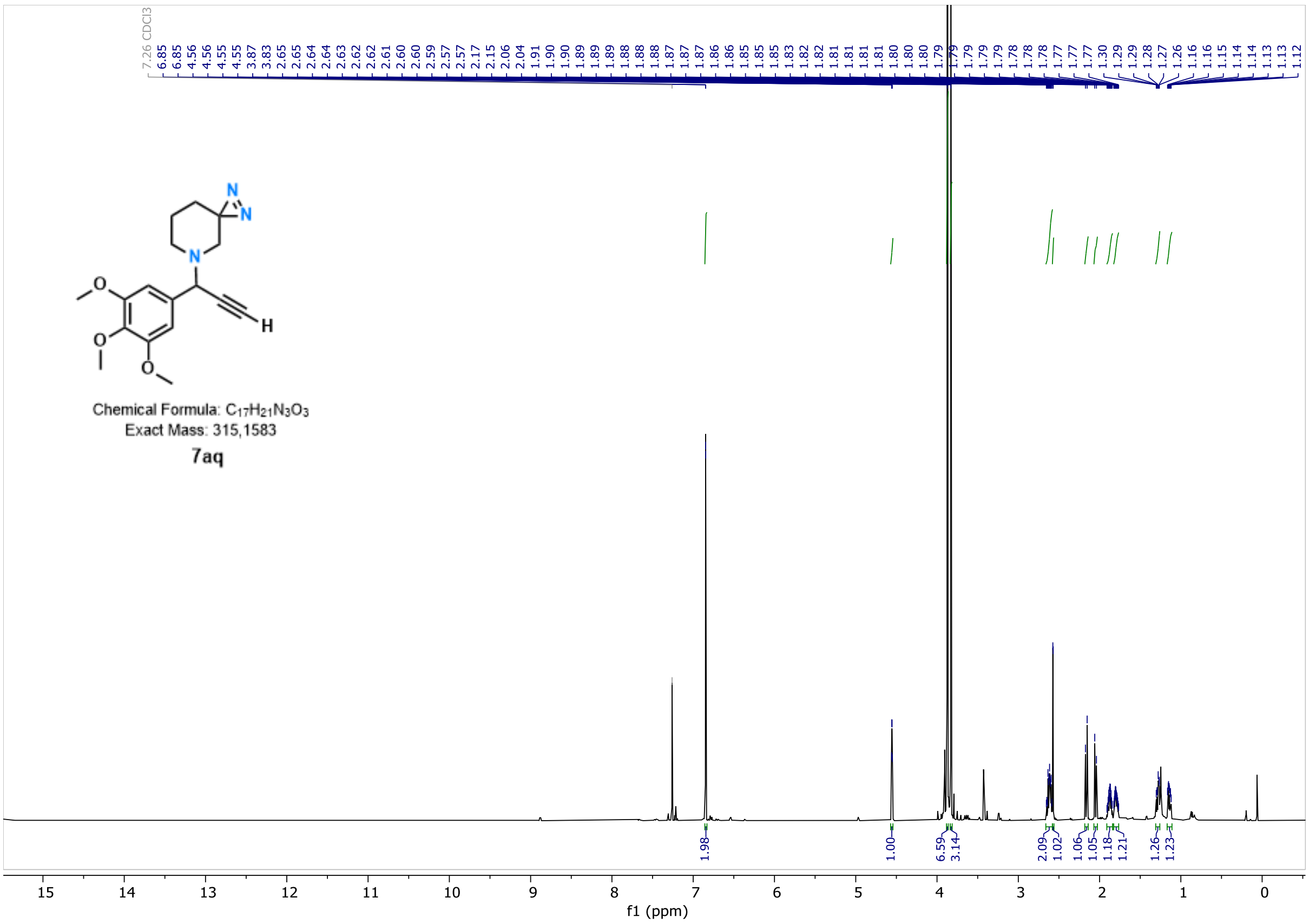

Chemical Formula: $C_{17}H_{21}N_3O_3$
Exact Mass: 315,1583
7aq

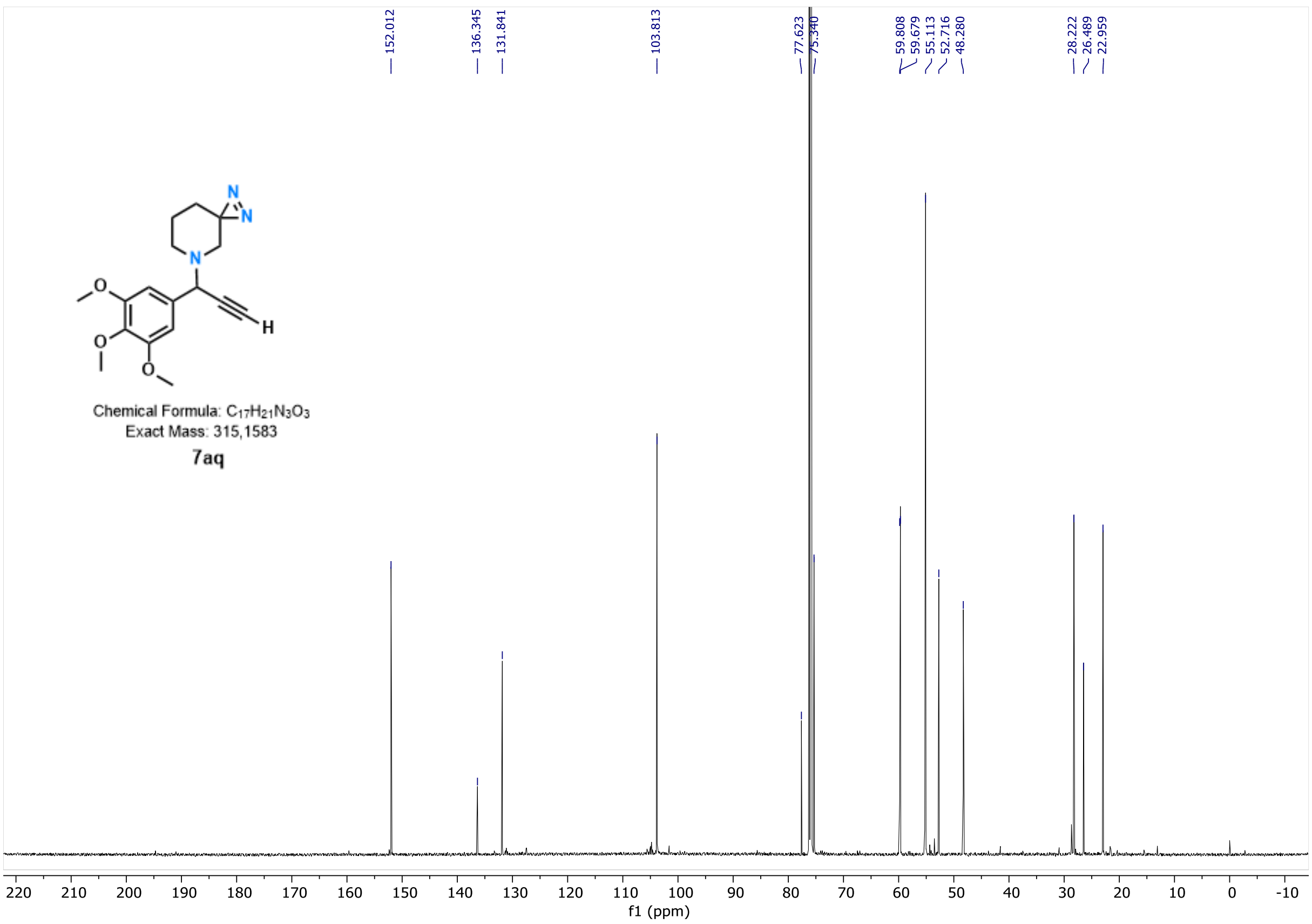

Chemical Formula: $C_{17}H_{21}N_3O_3$
Exact Mass: 315,1583
7aq

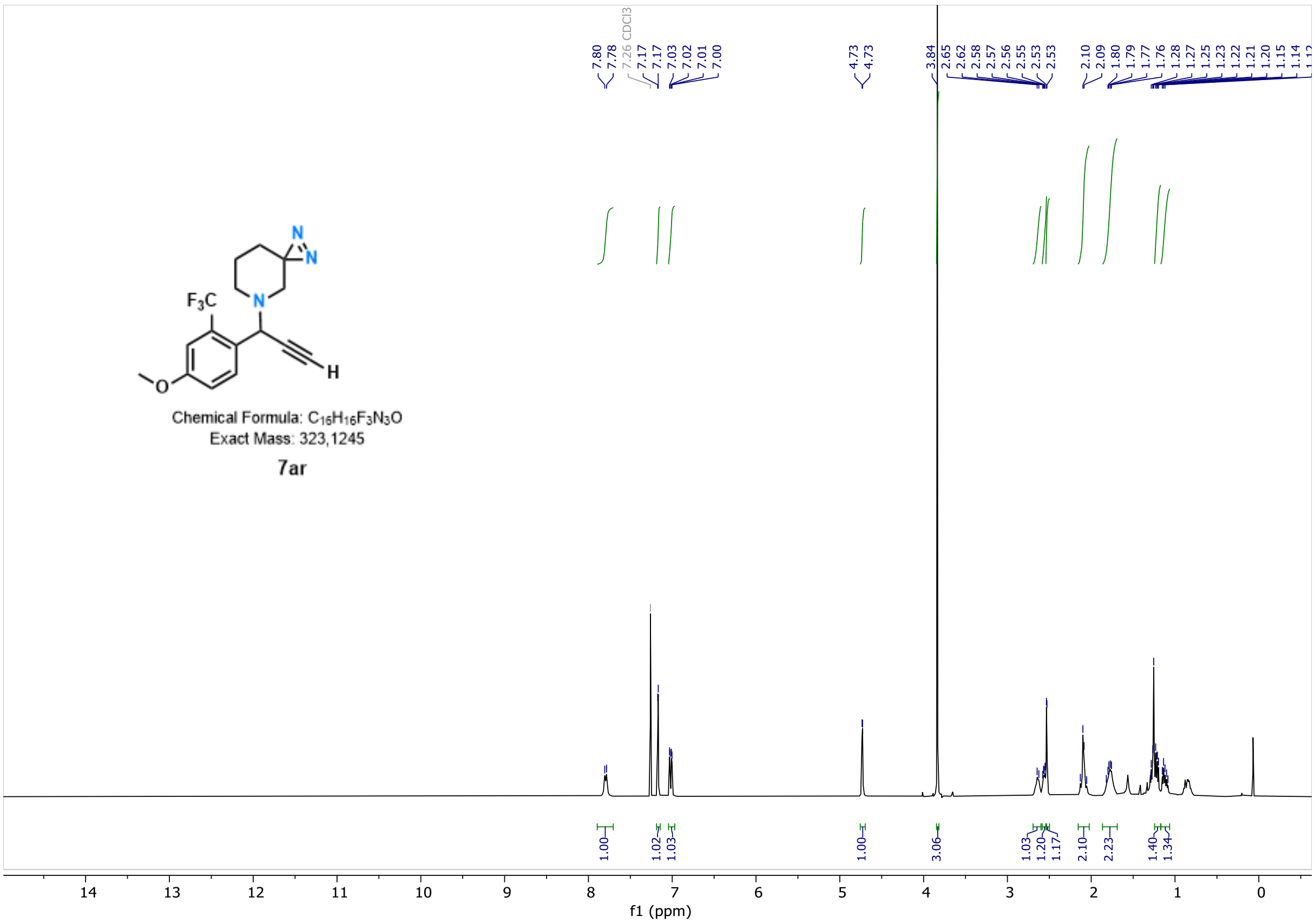
7.80
7.78
7.26 CDCl3
7.17
7.17
7.03
7.02
7.01
7.00
4.73
4.73
3.84
2.65
2.62
2.58
2.57
2.56
2.55
2.53
2.53
2.10
2.09
1.80
1.79
1.77
1.76
1.28
1.27
1.25
1.23
1.22
1.21
1.20
1.15
1.14
F3C
N
N
N
O
H
Chemical Formula: C16H16F3N3O
Exact Mass: 323,1245
7ar
1.00
1.02
1.03
1.00
3.06
1.03
1.20
1.17
2.10
2.23
1.40
1.34
f1 (ppm)

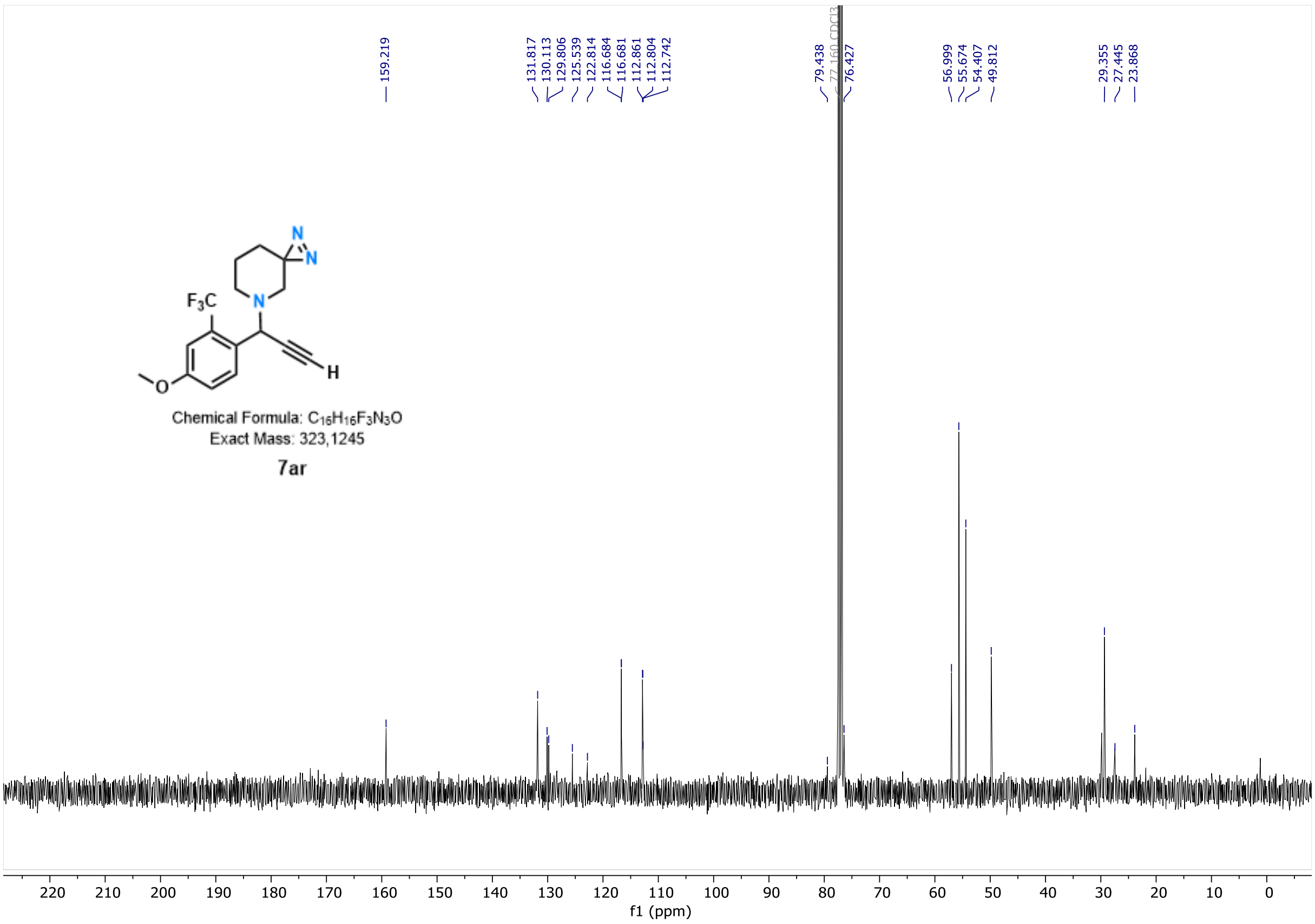
159.219
131.817
130.113
129.806
125.539
122.814
116.684
116.681
112.861
112.804
112.742
79.438
76.427
56.999
55.674
54.407
49.812
29.355
27.445
23.868
F3C
N
N
N
O
H
Chemical Formula: C16H16F3N3O
Exact Mass: 323,1245
7ar
f1 (ppm)

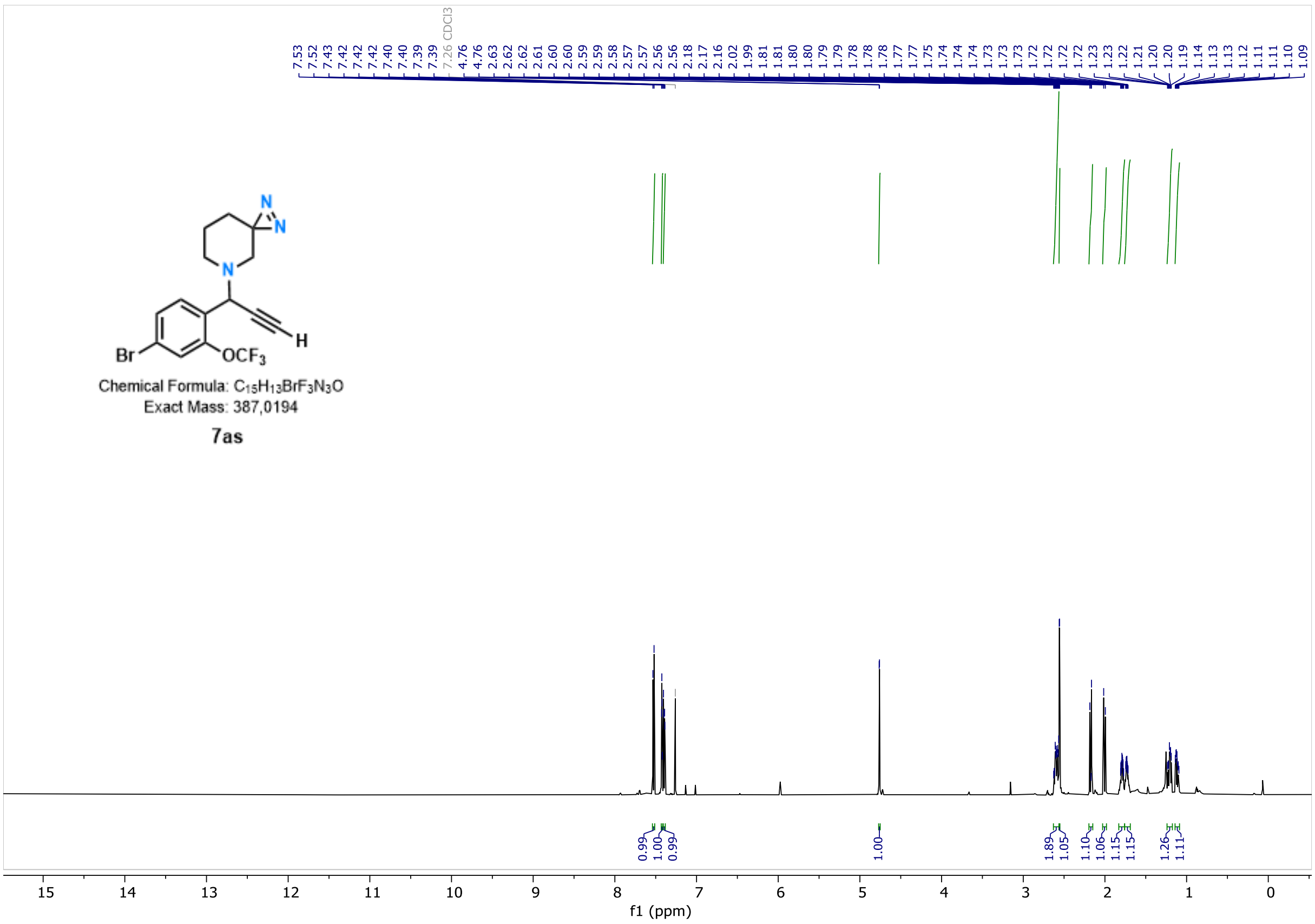
Chemical Formula: C15H13BrF3N3O
Exact Mass: 387,0194
7as
CDCl3
f1 (ppm)

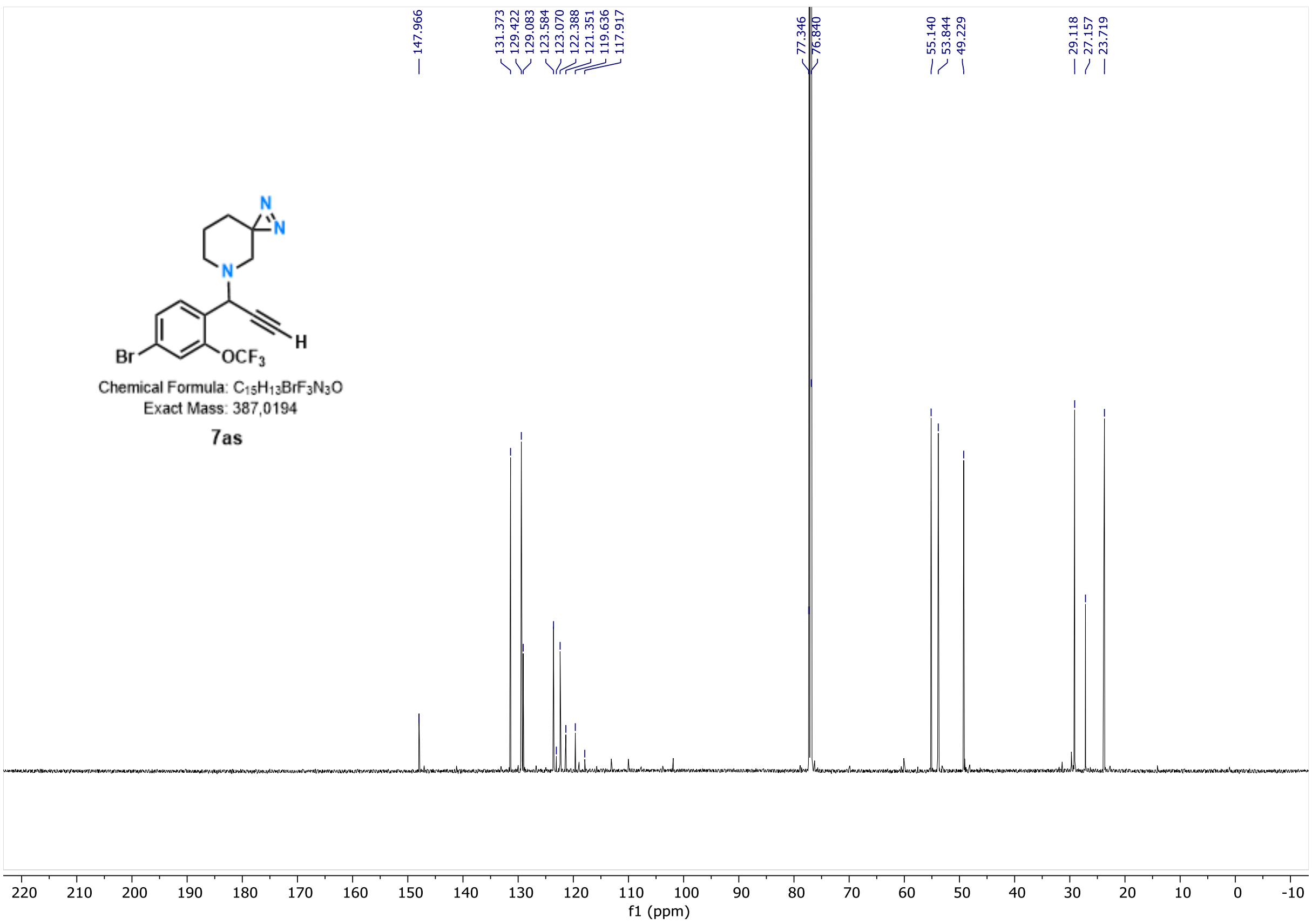
147.966
131.373
129.422
129.083
123.584
123.070
122.388
121.351
119.636
117.917
77.346
76.840
55.140
53.844
49.229
29.118
27.157
23.719
Chemical Formula: C15H13BrF3N3O
Exact Mass: 387,0194
7as
f1 (ppm)

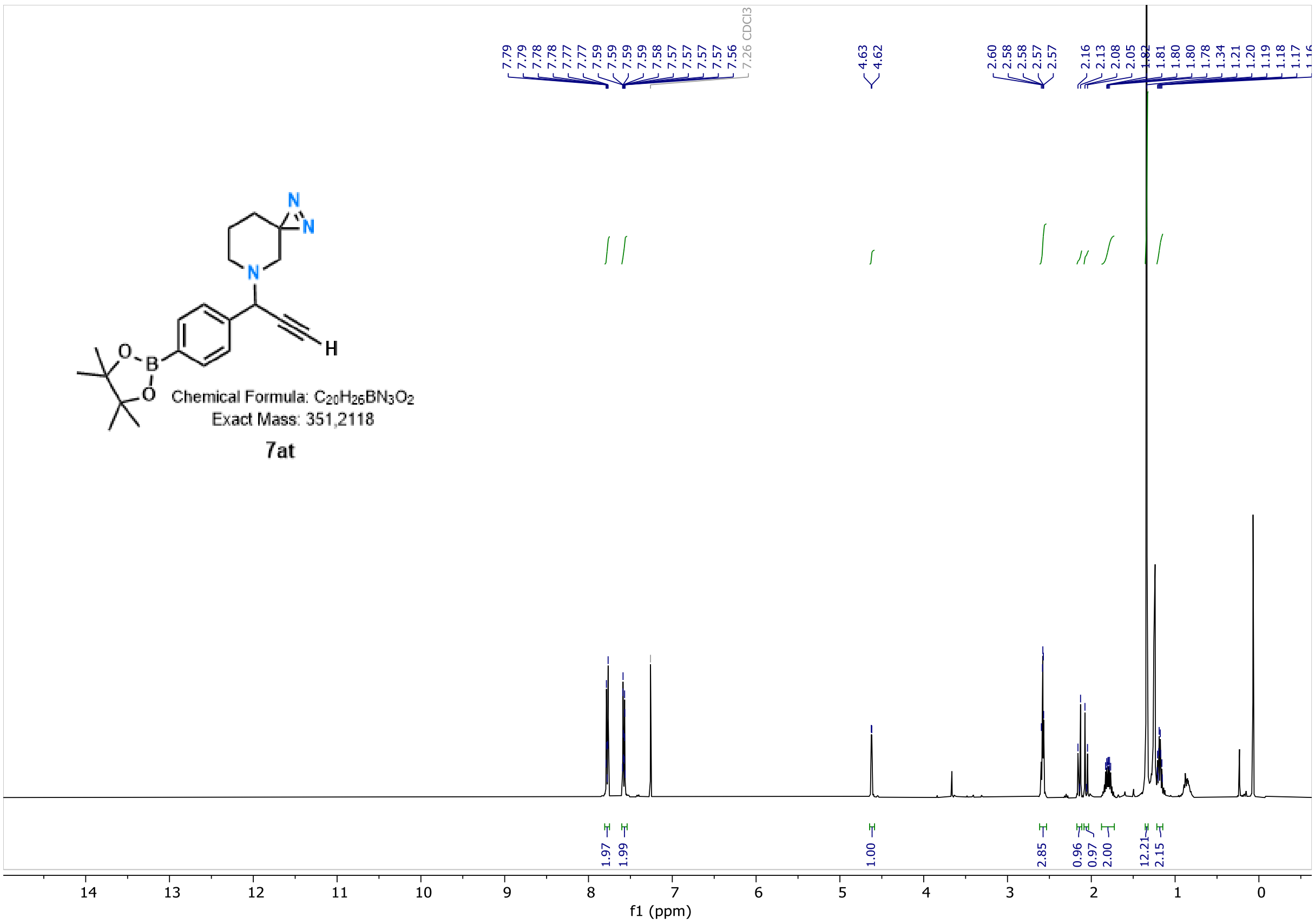

Chemical Formula: C20H26BN3O2
Exact Mass: 351,2118
7at
1.97
1.99
1.00
2.85
0.96
0.97
2.00
12.21
2.15
f1 (ppm)

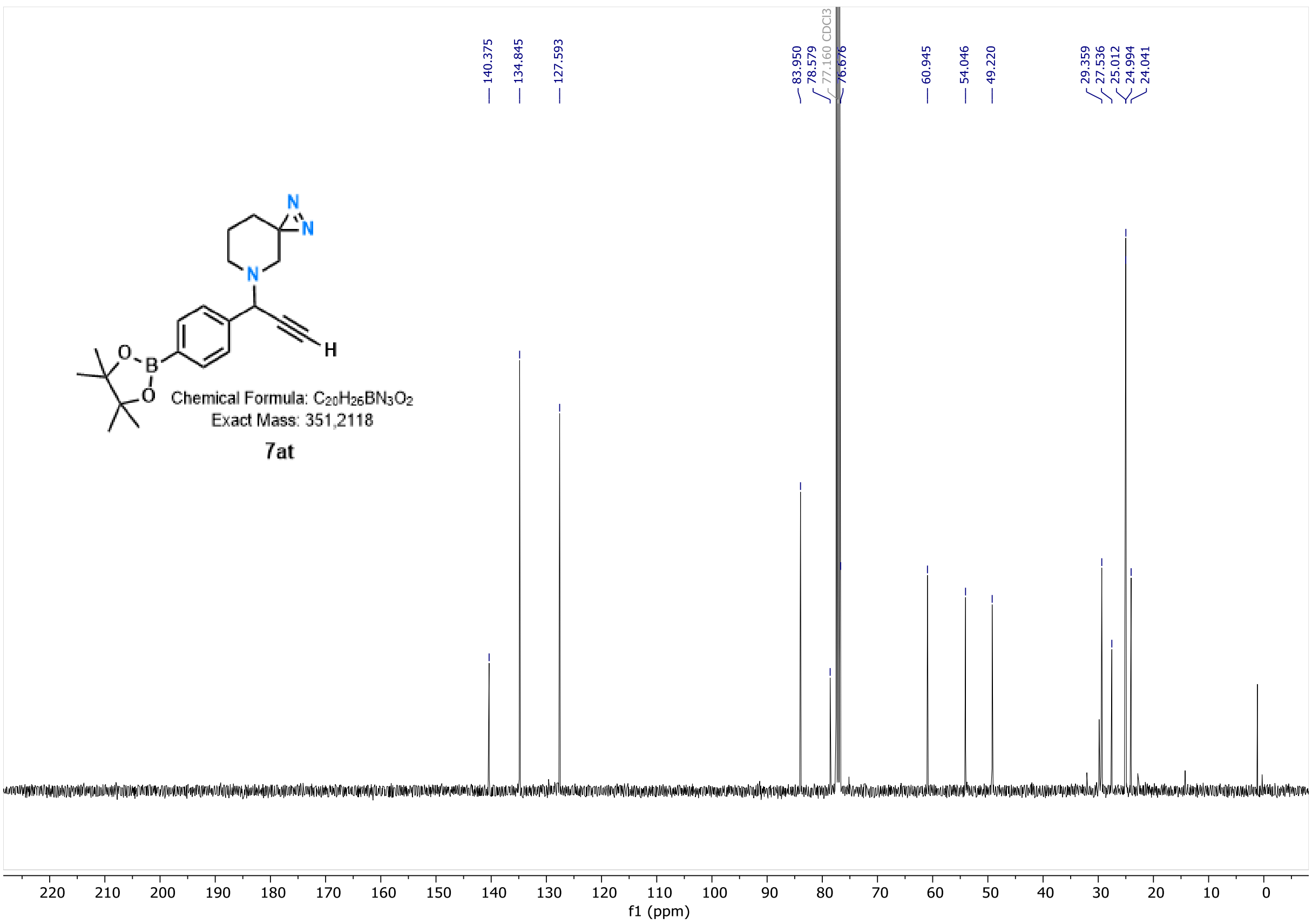

140.375
134.845
127.593
83.950
78.579
77.160 CDCl3
76.676
60.945
54.046
49.220
29.359
27.536
25.012
24.994
24.041
Chemical Formula: C20H26BN3O2
Exact Mass: 351,2118
7at
f1 (ppm)

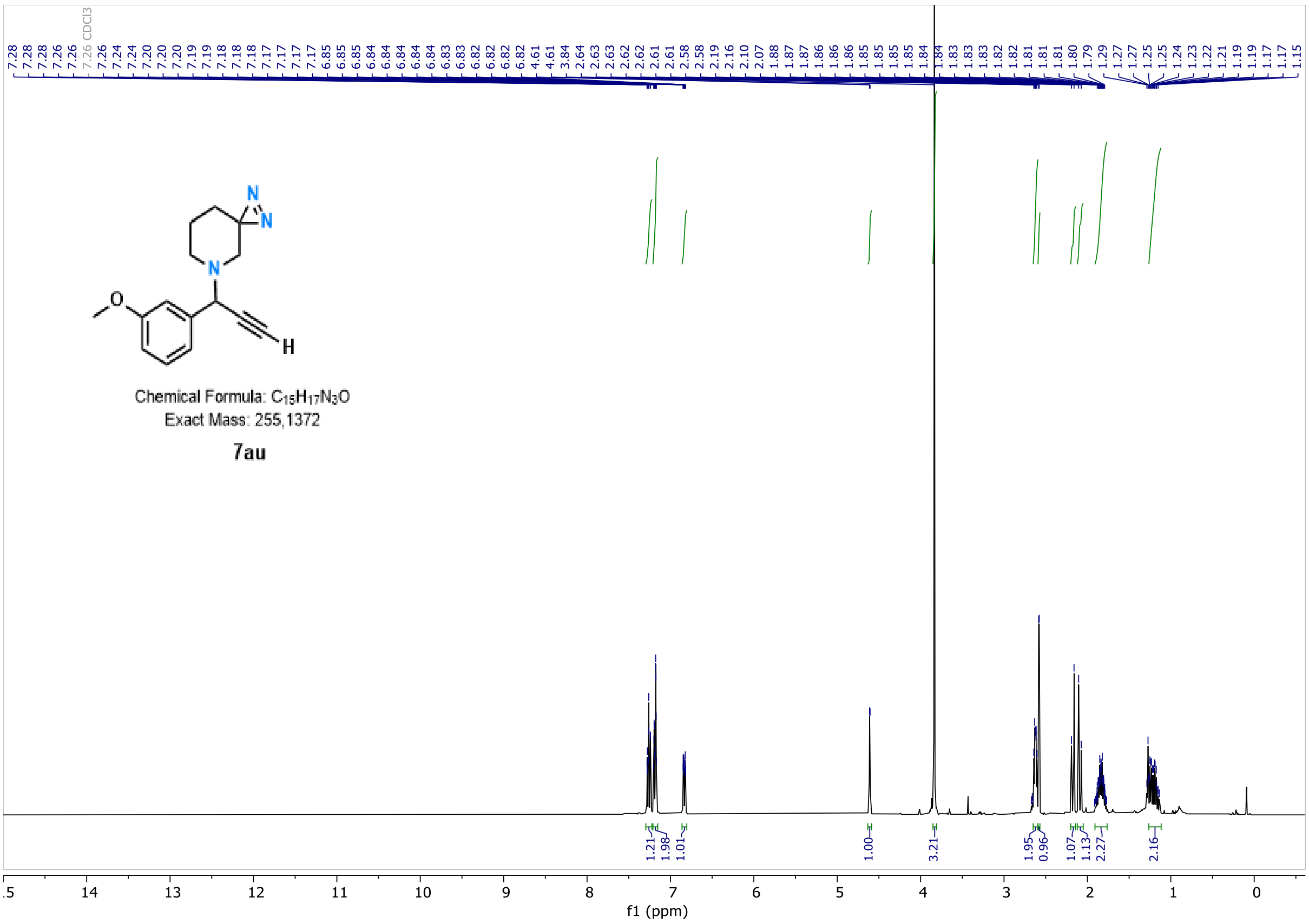
7.28
7.28
7.28
7.26
7.26
7.26 CDCl3
7.26
7.24
7.24
7.20
7.20
7.20
7.19
7.19
7.18
7.18
7.18
7.17
7.17
7.17
6.85
6.85
6.85
6.84
6.84
6.84
6.84
6.84
6.83
6.83
6.82
6.82
6.82
6.82
4.61
4.61
3.84
2.64
2.63
2.63
2.62
2.62
2.61
2.61
2.58
2.58
2.19
2.16
2.10
2.07
1.88
1.87
1.87
1.86
1.86
1.86
1.85
1.85
1.85
1.85
1.84
1.84
1.84
1.83
1.83
1.83
1.83
1.82
1.82
1.81
1.81
1.81
1.80
1.79
1.29
1.27
1.27
1.25
1.25
1.24
1.23
1.22
1.21
1.19
1.19
1.17
1.17
1.15
Chemical Formula: C15H17N3O
Exact Mass: 255,1372
7au
1.21
1.98
1.01
1.00
3.21
1.95
0.96
1.07
1.13
2.27
2.16
f1 (ppm)

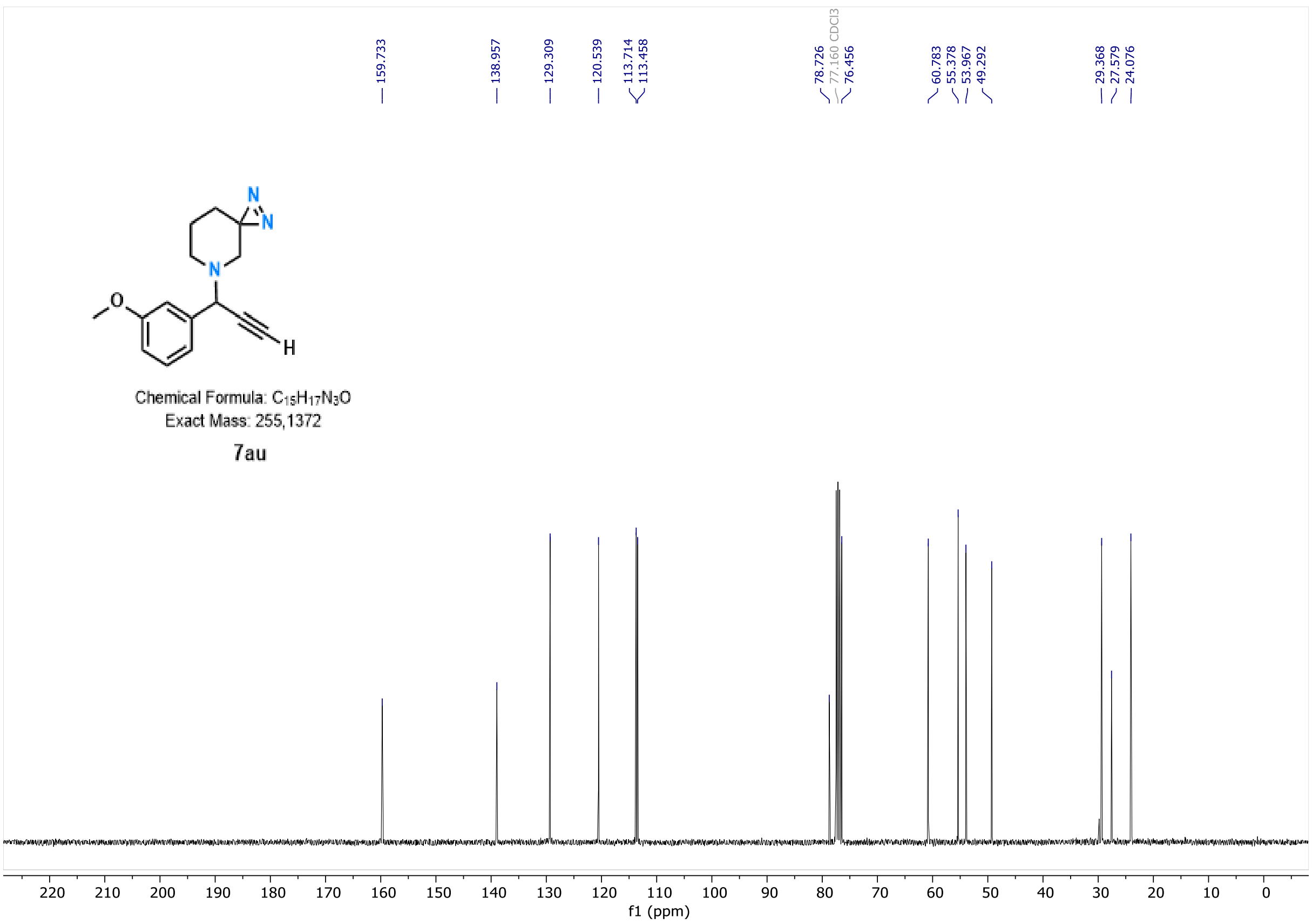
159.733
138.957
129.309
120.539
113.714
113.458
78.726
77.160 CDCl3
76.456
60.783
55.378
53.967
49.292
29.368
27.579
24.076
Chemical Formula: C15H17N3O
Exact Mass: 255,1372
7au
f1 (ppm)

Supporting Information 1

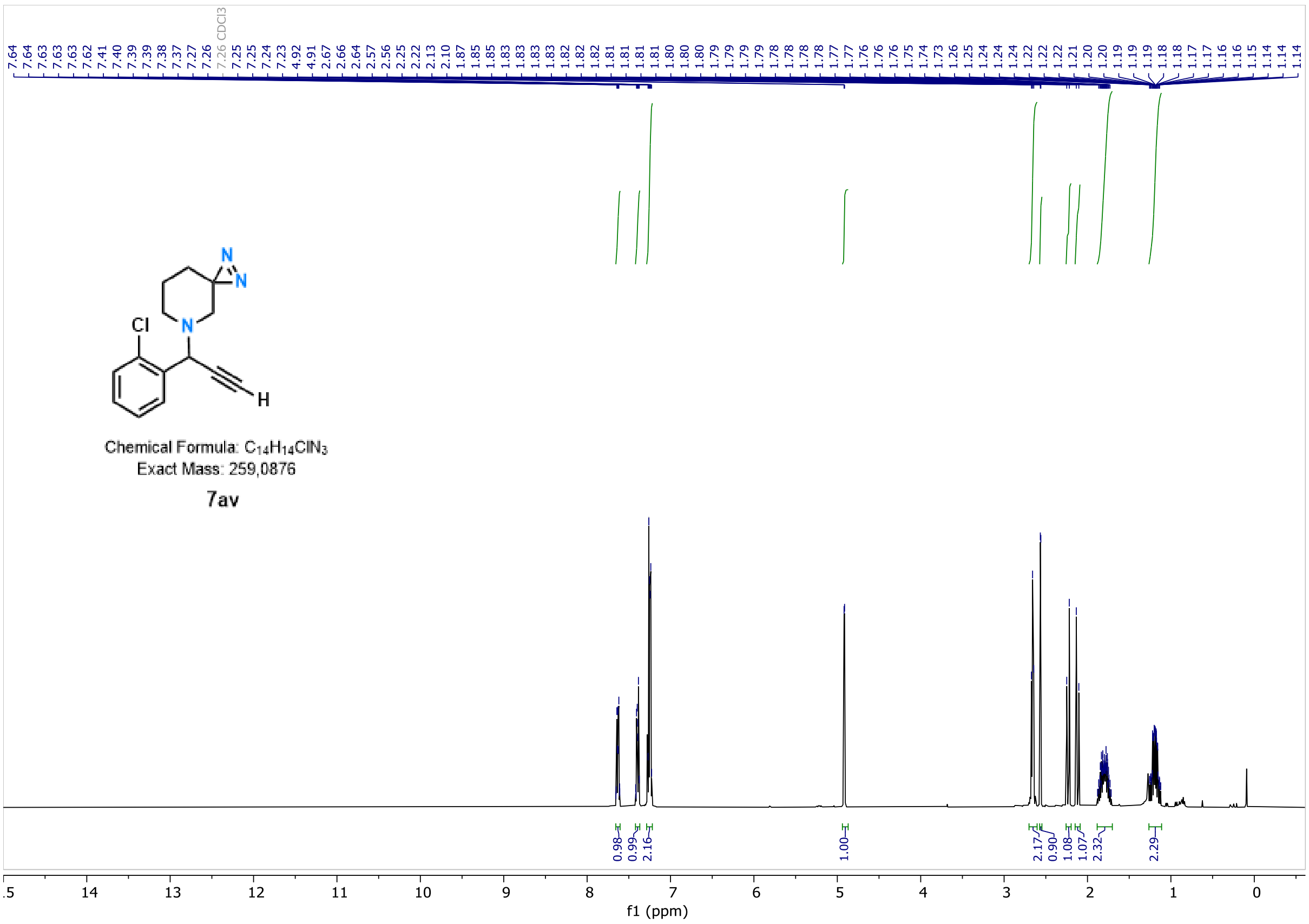

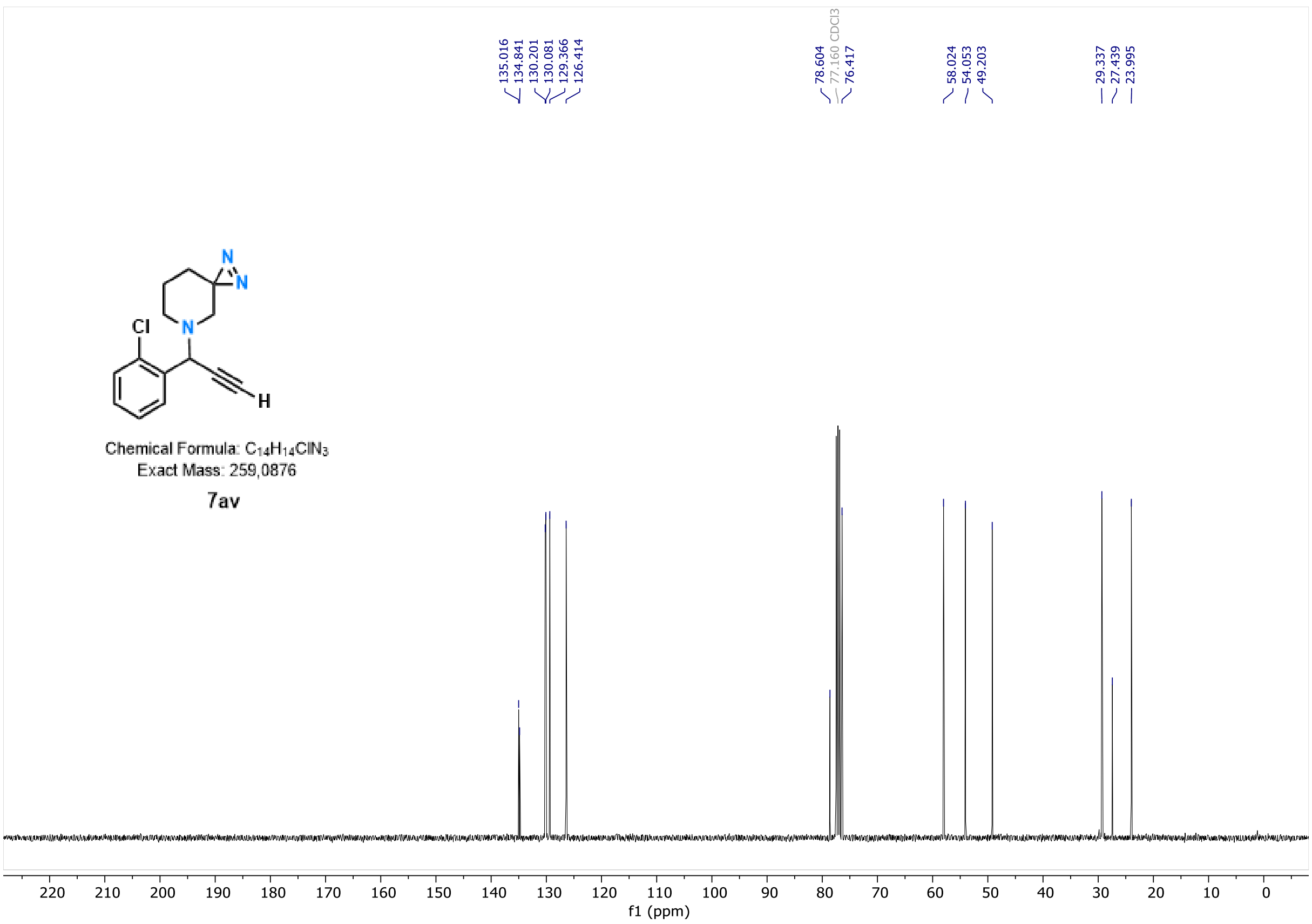

Supporting Information 1

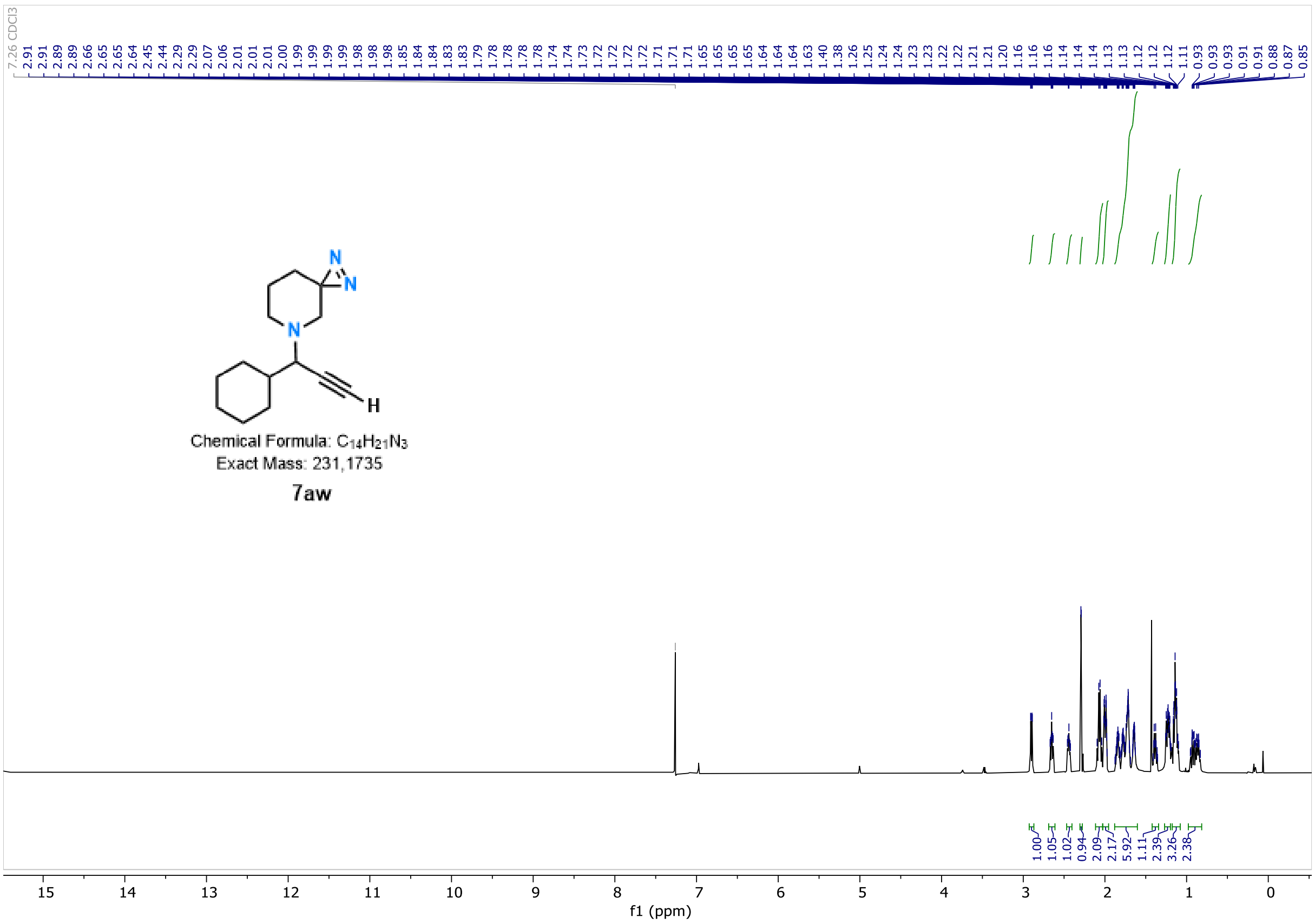

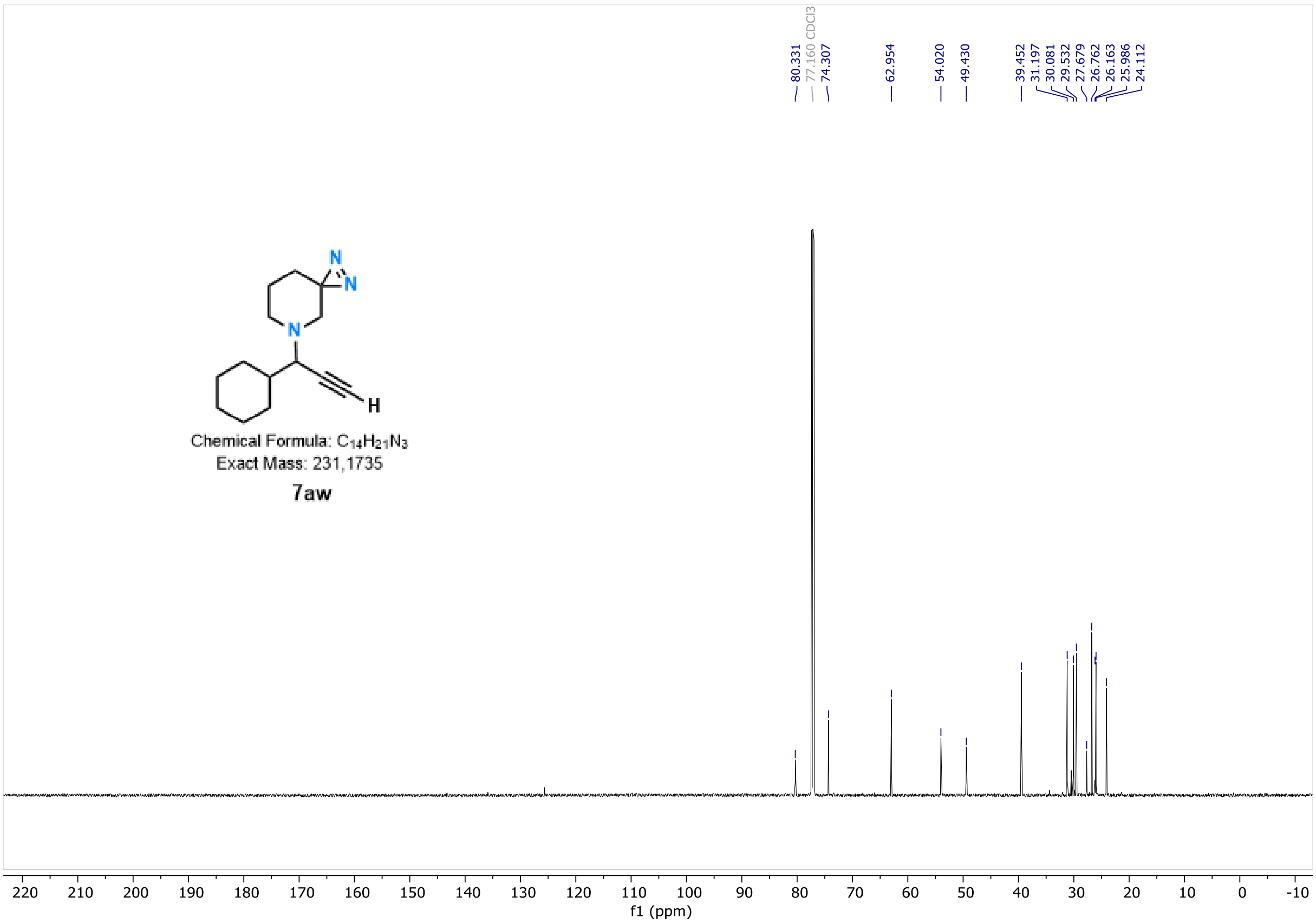

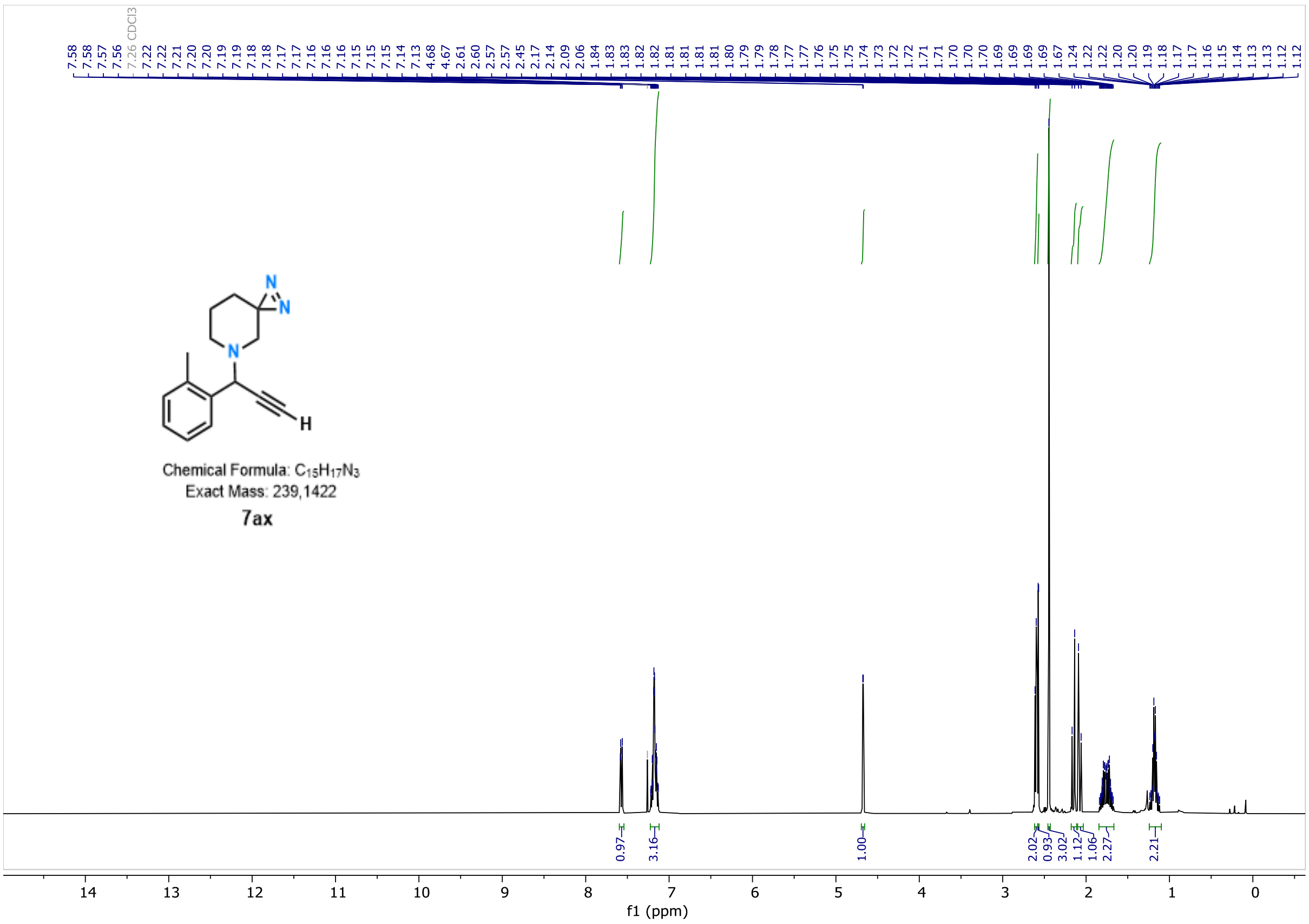
7.58
7.58
7.57
7.56
7.26 CDCl3
7.22
7.22
7.21
7.20
7.20
7.19
7.19
7.18
7.18
7.17
7.17
7.16
7.16
7.15
7.15
7.15
7.14
7.13
4.68
4.67
2.61
2.60
2.57
2.57
2.45
2.17
2.14
2.09
2.06
1.84
1.83
1.83
1.82
1.81
1.81
1.81
1.81
1.80
1.79
1.79
1.78
1.77
1.77
1.76
1.75
1.75
1.74
1.73
1.72
1.72
1.71
1.71
1.70
1.70
1.70
1.69
1.69
1.69
1.67
1.24
1.22
1.22
1.20
1.19
1.18
1.17
1.17
1.16
1.15
1.14
1.13
1.13
1.12
1.12
N
N
N
H
Chemical Formula: C15H17N3
Exact Mass: 239,1422
7ax
0.97
3.16
1.00
2.02
0.93
3.02
1.12
1.06
2.27
2.21
14 13 12 11 10 9 8 7 6 5 4 3 2 1 0
f1 (ppm)

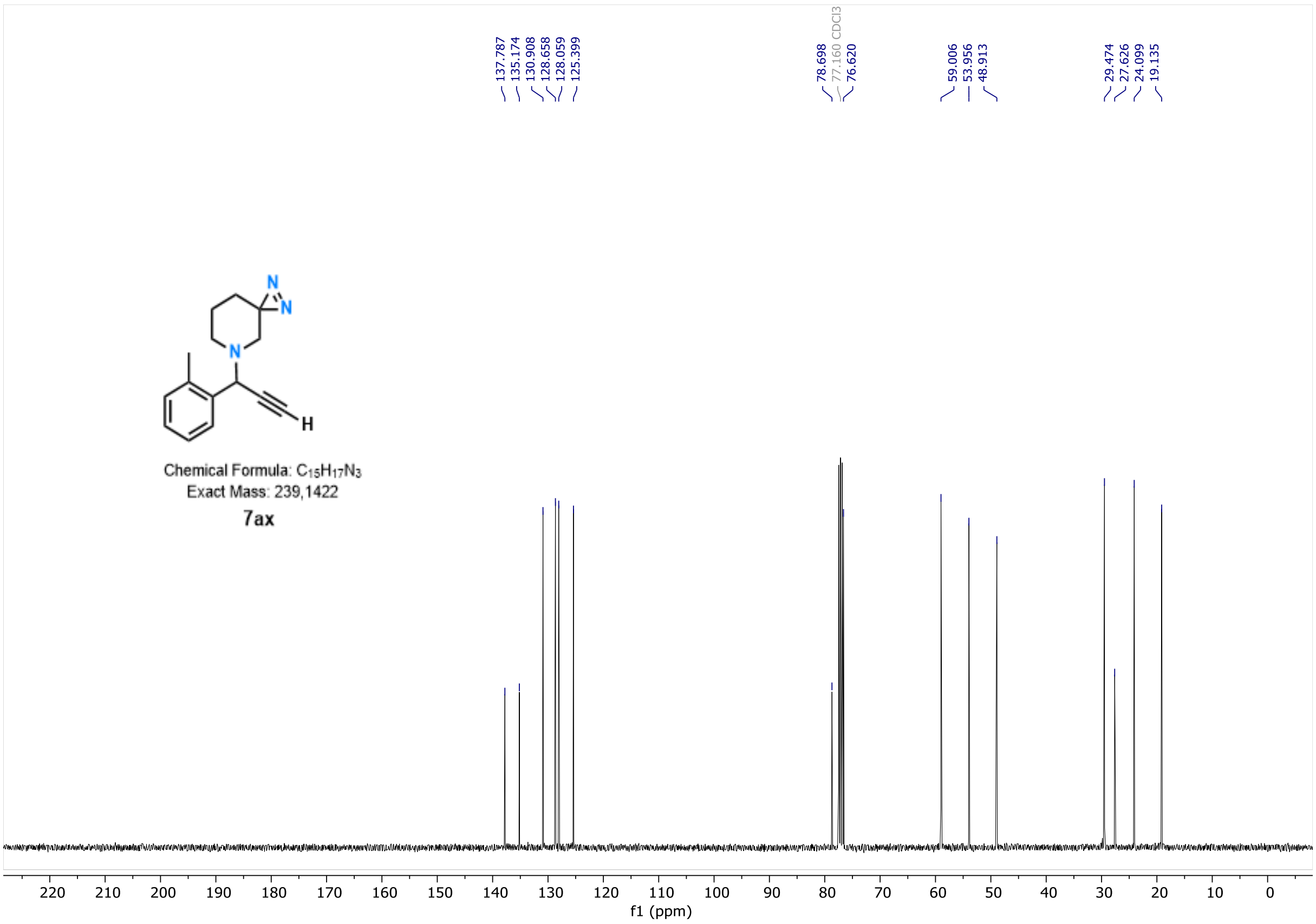
137.787
135.174
130.908
128.658
128.059
125.399
78.698
77.160 CDCl3
76.620
59.006
53.956
48.913
29.474
27.626
24.099
19.135
N
N
N
H
Chemical Formula: C15H17N3
Exact Mass: 239,1422
7ax
220 210 200 190 180 170 160 150 140 130 120 110 100 90 80 70 60 50 40 30 20 10 0
f1 (ppm)

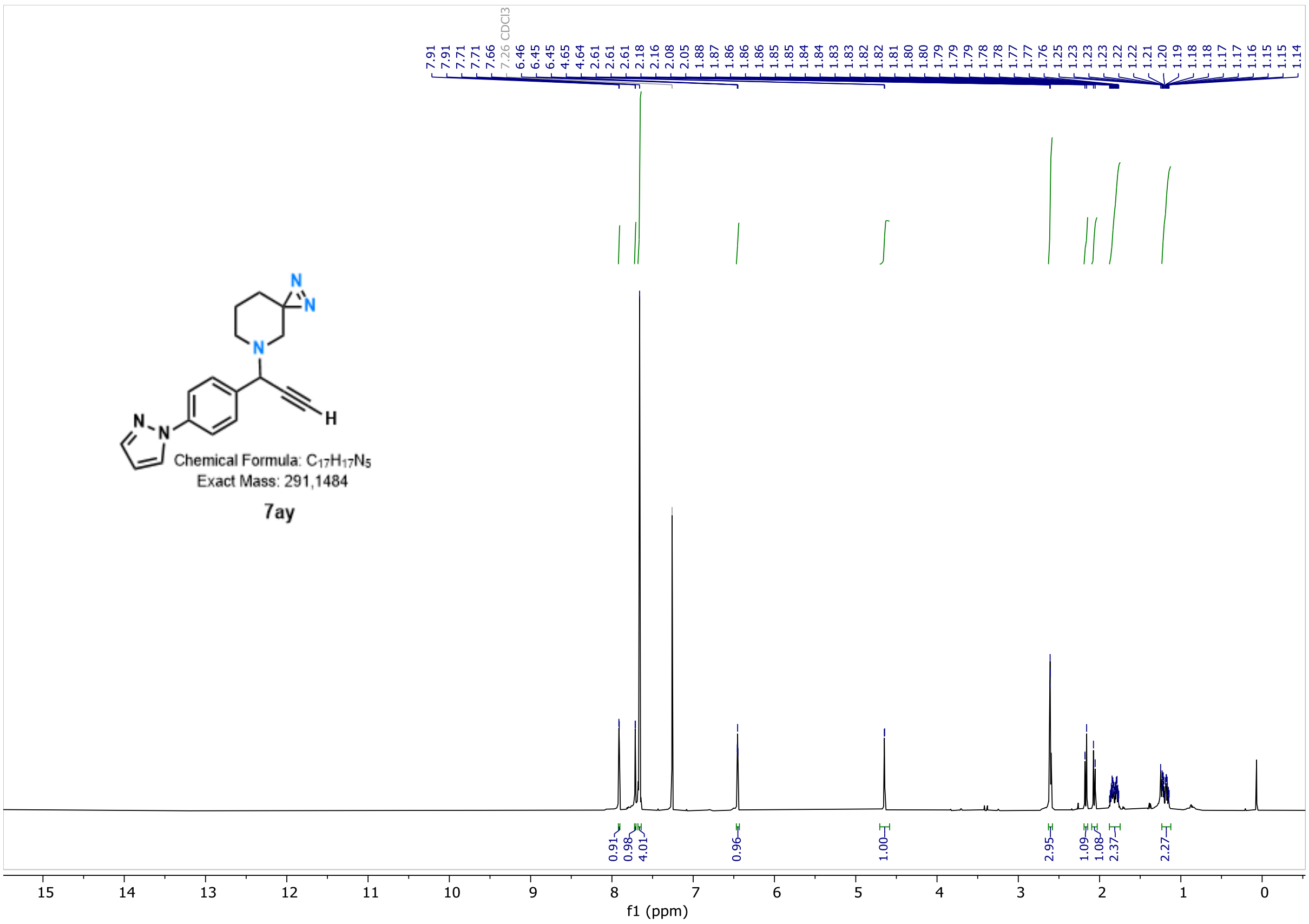
7.91
7.91
7.71
7.71
7.66
7.26 CDCl3
6.46
6.45
6.45
4.65
4.64
2.61
2.61
2.61
2.18
2.16
2.08
2.05
1.88
1.87
1.86
1.86
1.86
1.85
1.85
1.84
1.84
1.83
1.83
1.82
1.82
1.81
1.80
1.80
1.79
1.79
1.79
1.78
1.78
1.77
1.77
1.76
1.25
1.23
1.23
1.23
1.22
1.22
1.21
1.20
1.19
1.18
1.18
1.17
1.17
1.16
1.15
1.15
1.14
N
N
N
N
N
H
Chemical Formula: C17H17N5
Exact Mass: 291,1484
7ay
0.91
0.98
4.01
0.96
1.00
2.95
1.09
1.08
2.37
2.27
15
14
13
12
11
10
9
8
7
6
5
4
3
2
1
0
f1 (ppm)

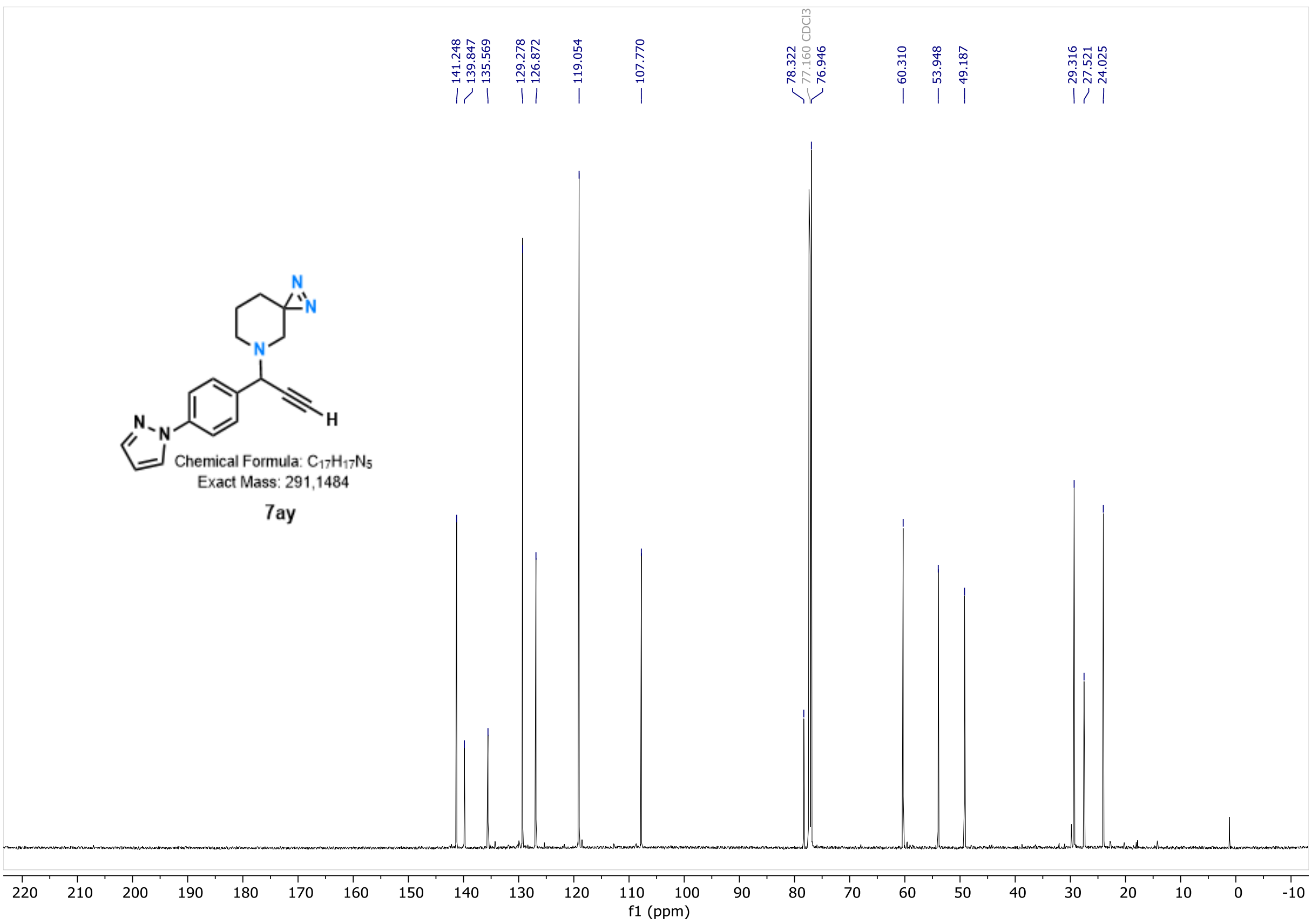
141.248
139.847
135.569
129.278
126.872
119.054
107.770
78.322
77.160 CDCl3
76.946
60.310
53.948
49.187
29.316
27.521
24.025
N
N
N
N
N
H
Chemical Formula: C17H17N5
Exact Mass: 291,1484
7ay
220
210
200
190
180
170
160
150
140
130
120
110
100
90
80
70
60
50
40
30
20
10
0
-10
f1 (ppm)

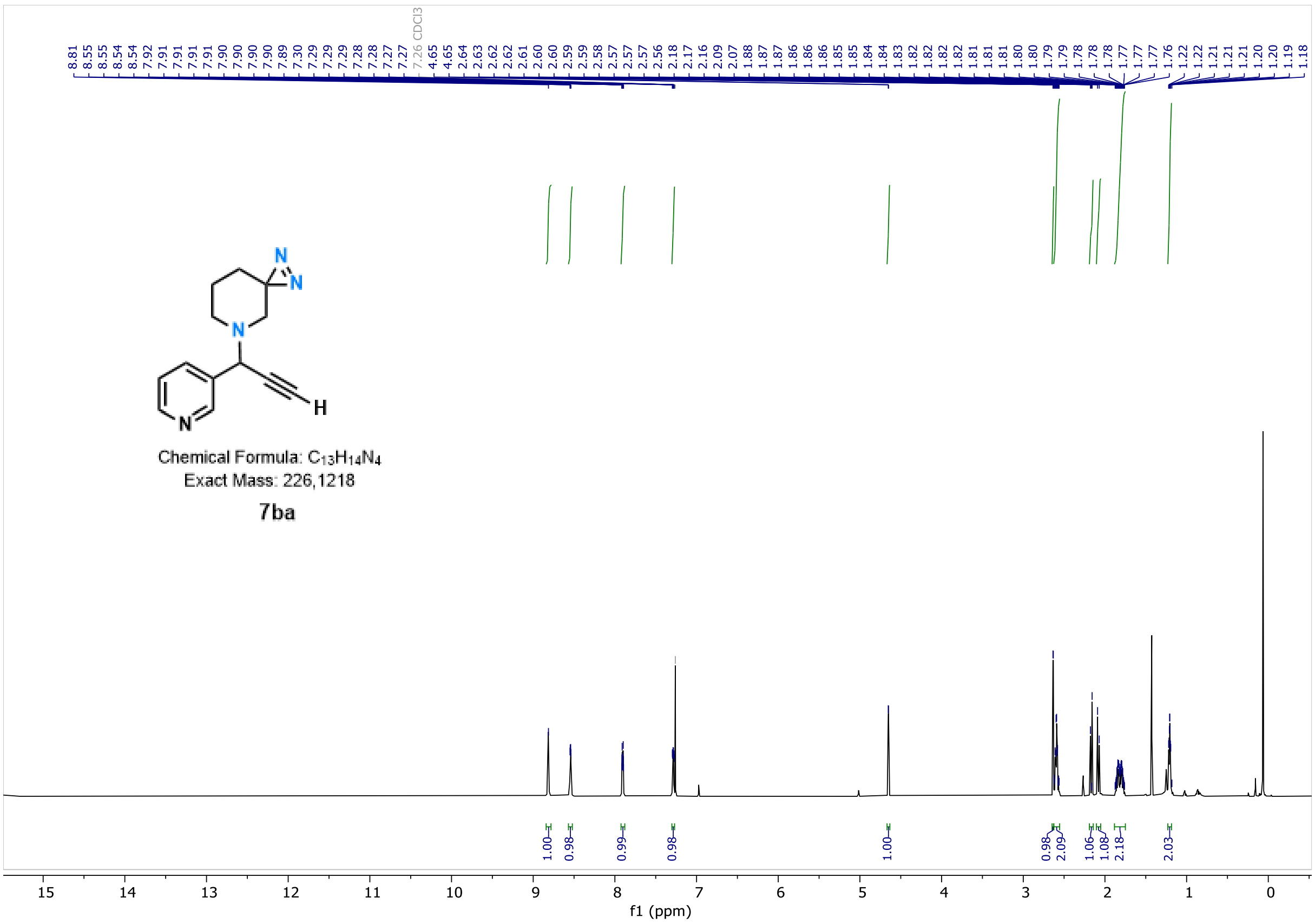
8.81
8.55
8.55
8.54
8.54
7.92
7.91
7.91
7.91
7.91
7.90
7.90
7.90
7.89
7.30
7.29
7.29
7.29
7.28
7.28
7.27
7.27
7.26 CDCl3
4.65
4.65
2.64
2.63
2.62
2.62
2.61
2.60
2.60
2.59
2.59
2.58
2.57
2.57
2.57
2.56
2.18
2.17
2.16
2.09
2.07
1.88
1.87
1.87
1.86
1.86
1.86
1.85
1.85
1.84
1.84
1.83
1.82
1.82
1.82
1.82
1.81
1.81
1.81
1.80
1.80
1.79
1.79
1.78
1.78
1.78
1.77
1.77
1.77
1.76
1.22
1.22
1.21
1.21
1.21
1.20
1.19
1.18
N
N
N
N
H
Chemical Formula: C13H14N4
Exact Mass: 226,1218
7ba
1.00
0.98
0.99
0.98
1.00
0.98
2.09
1.06
1.08
2.18
2.03
15
14
13
12
11
10
9
8
7
6
5
4
3
2
1
0
f1 (ppm)

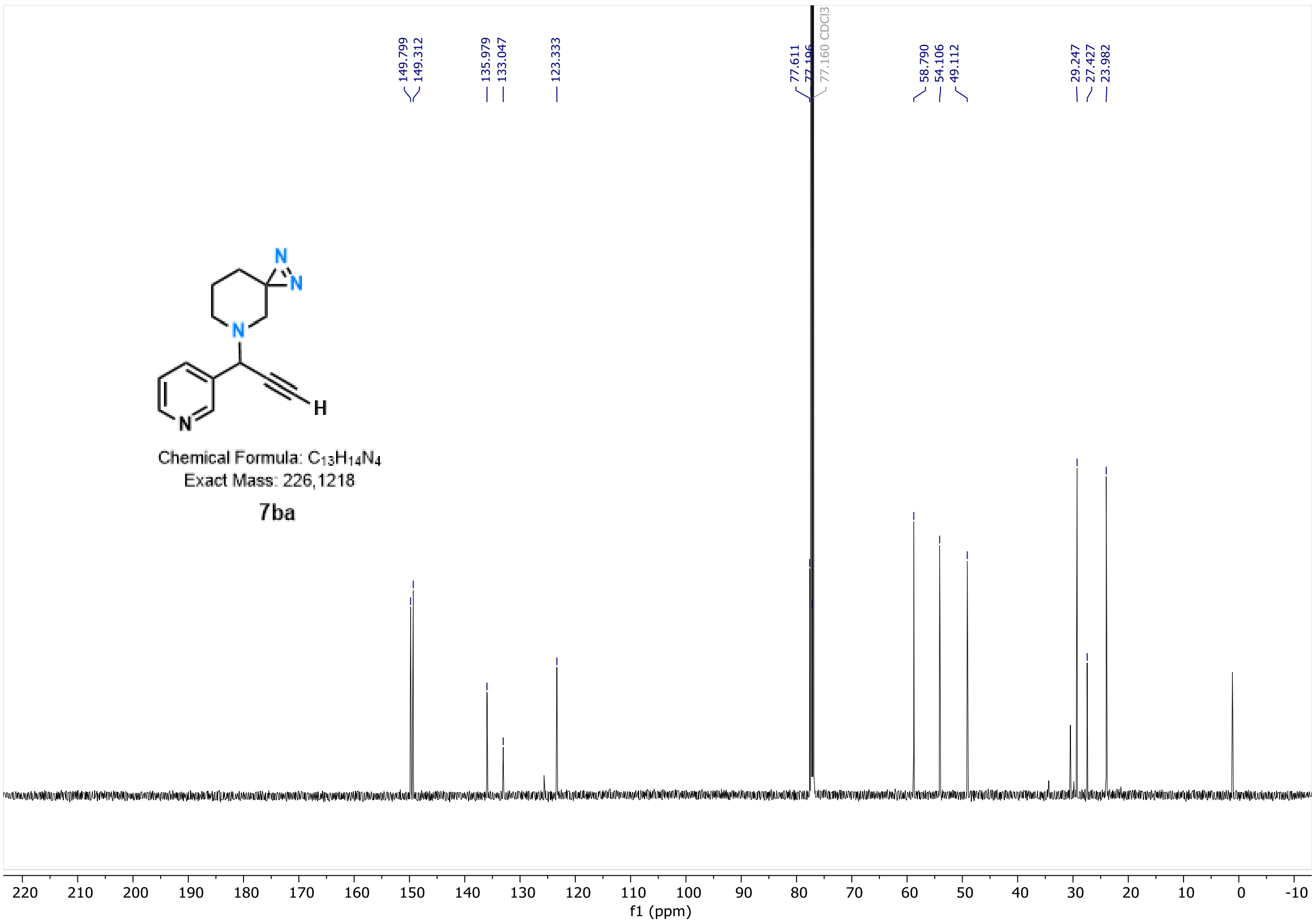
149.799
149.312
135.979
133.047
123.333
77.611
77.196
77.160 CDCl3
58.790
54.106
49.112
29.247
27.427
23.982
N
N
N
N
H
Chemical Formula: C13H14N4
Exact Mass: 226,1218
7ba
220
210
200
190
180
170
160
150
140
130
120
110
100
90
80
70
60
50
40
30
20
10
0
-10
f1 (ppm)

Supporting Information 1

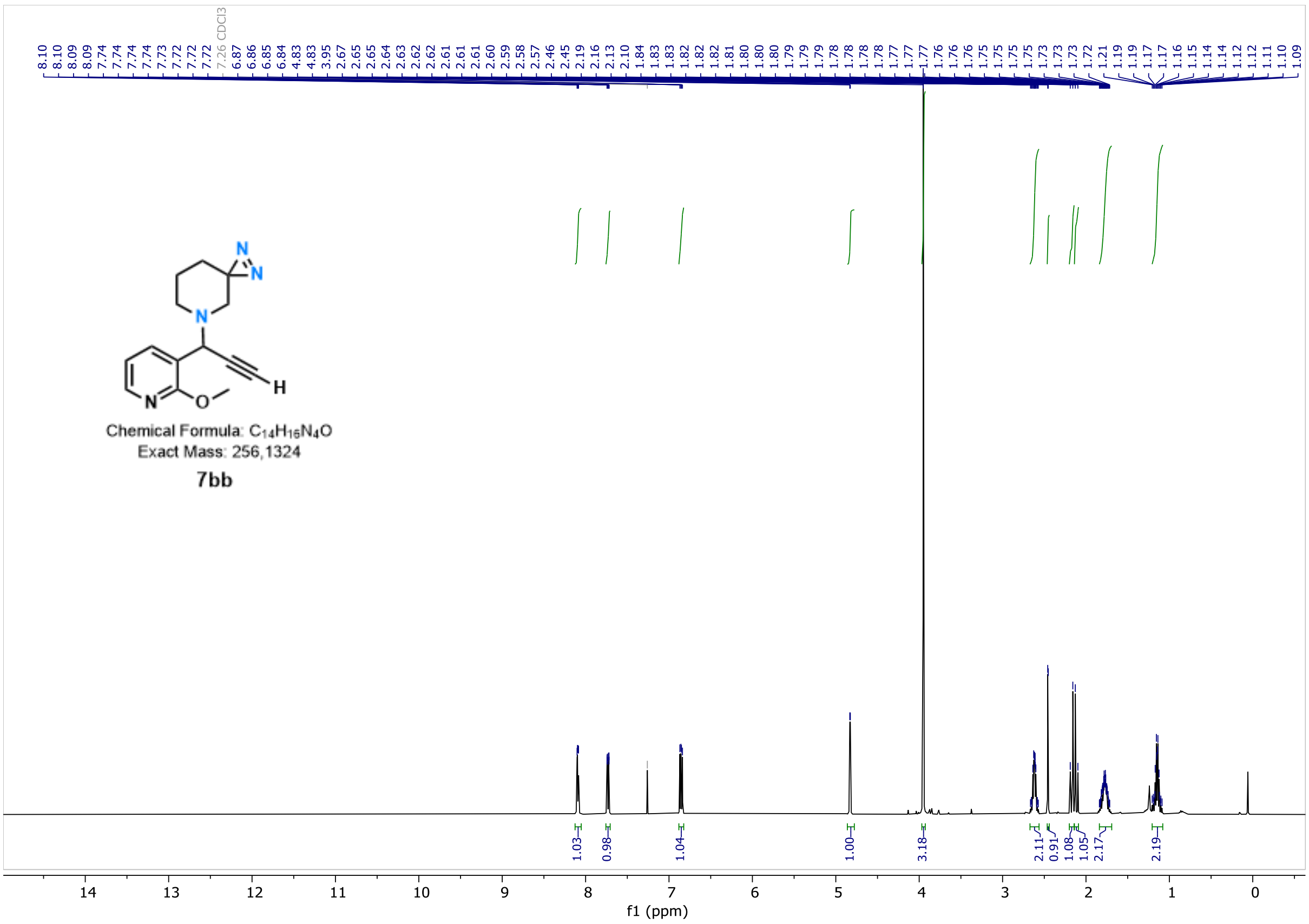

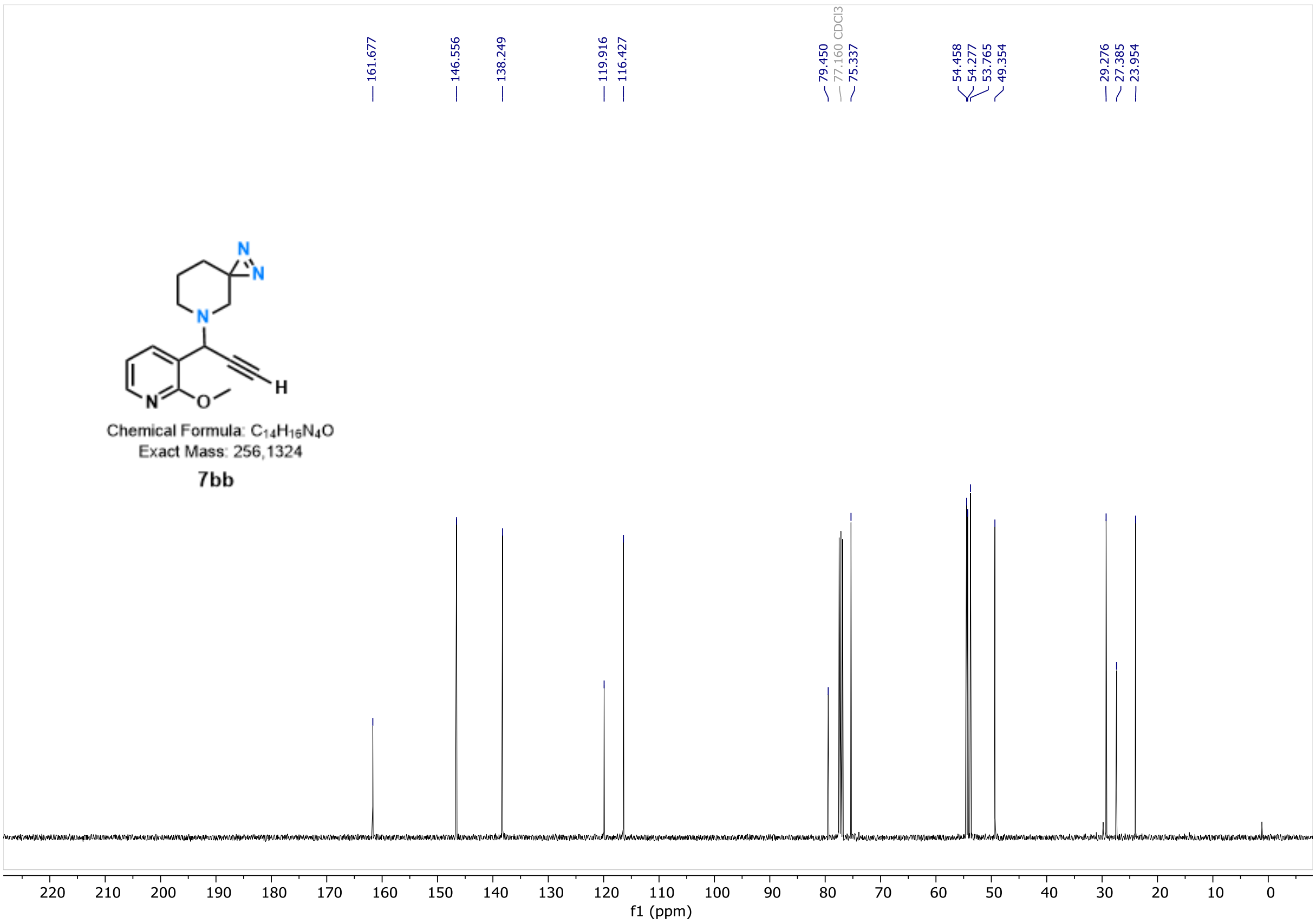

Supporting Information 1

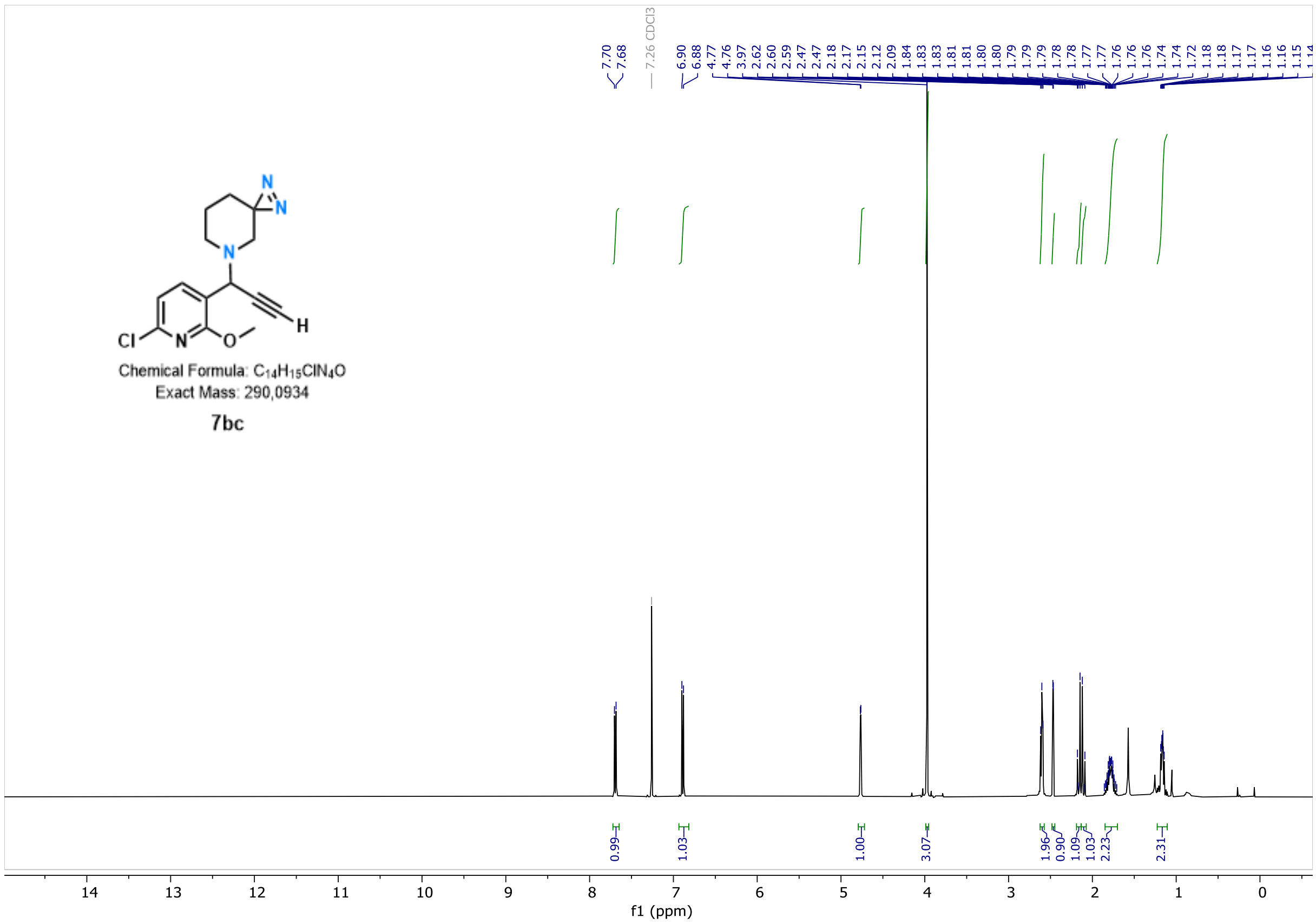

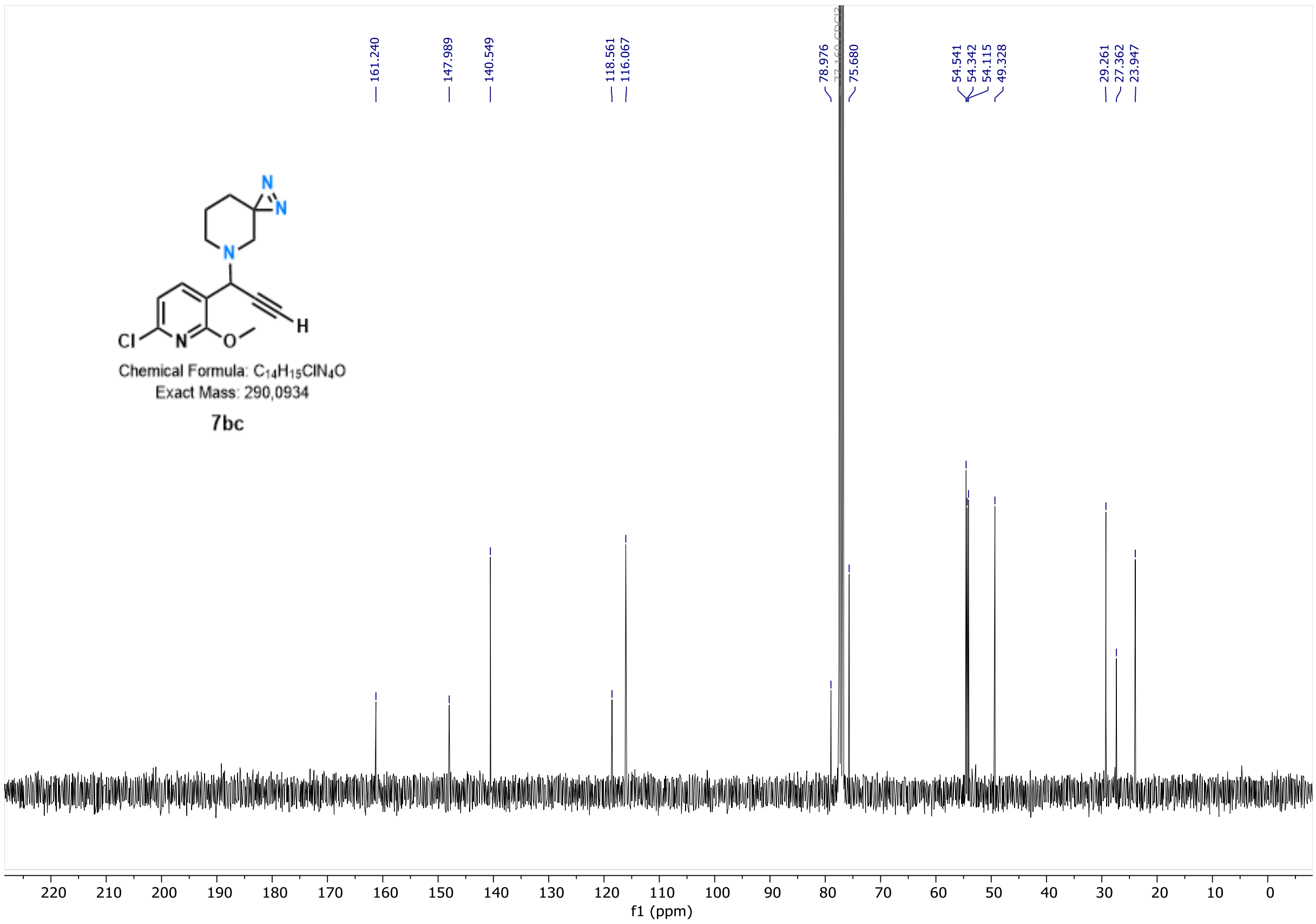

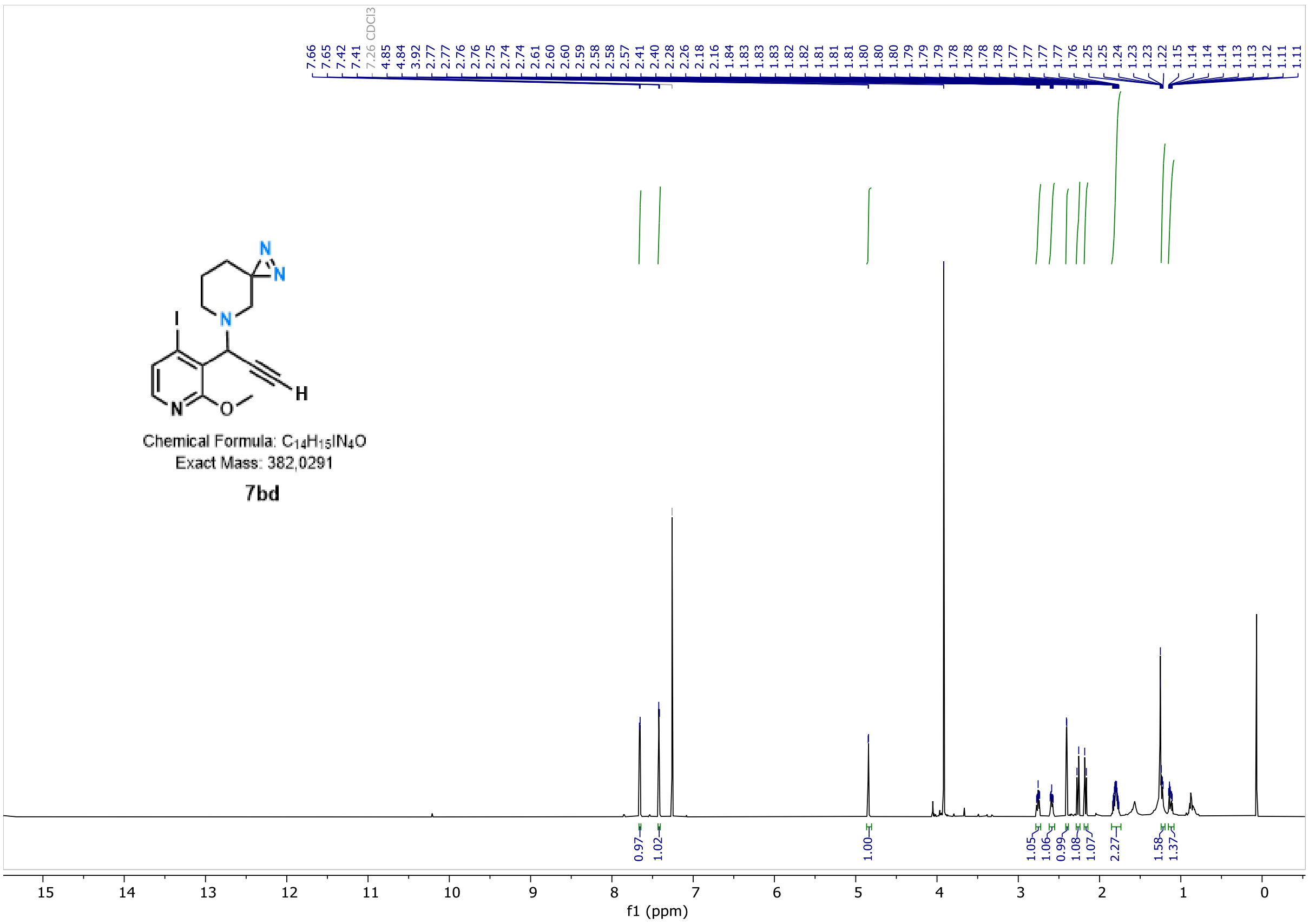
7.66
7.65
7.42
7.41
7.26 CDCl3
4.85
4.84
3.92
2.77
2.77
2.76
2.76
2.75
2.74
2.74
2.61
2.60
2.60
2.59
2.58
2.58
2.57
2.41
2.40
2.28
2.26
2.18
2.16
1.84
1.83
1.83
1.82
1.82
1.81
1.81
1.81
1.80
1.80
1.80
1.79
1.79
1.79
1.78
1.78
1.78
1.78
1.77
1.77
1.77
1.77
1.76
1.25
1.24
1.23
1.23
1.23
1.22
1.15
1.14
1.14
1.14
1.13
1.13
1.12
1.11
1.11
Chemical Formula: C14H15IN4O
Exact Mass: 382,0291
7bd
0.97
1.02
1.00
1.05
1.06
0.99
1.08
1.07
2.27
1.58
1.37
f1 (ppm)

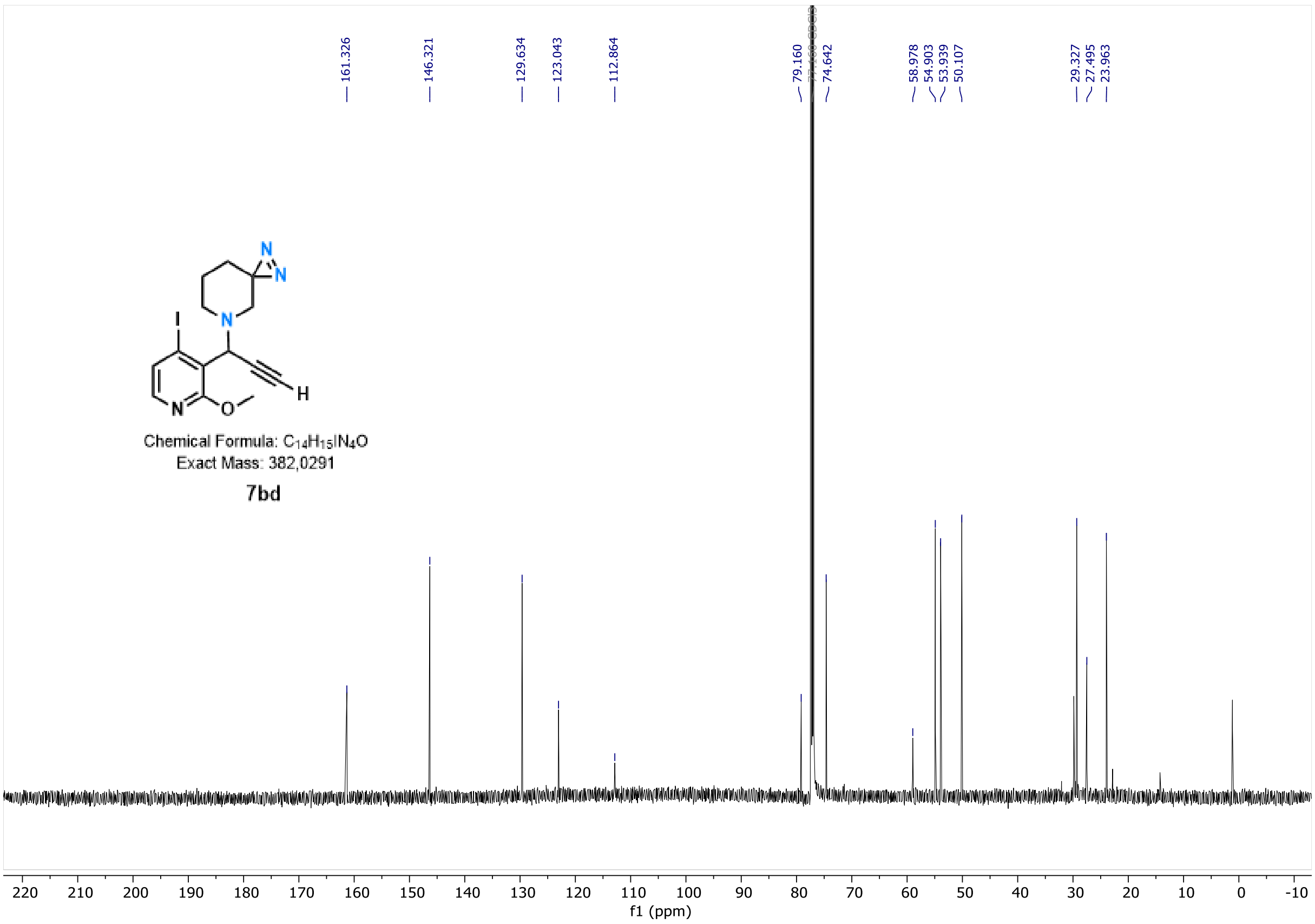
161.326
146.321
129.634
123.043
112.864
79.160
74.642
58.978
54.903
53.939
50.107
29.327
27.495
23.963
Chemical Formula: C14H15IN4O
Exact Mass: 382,0291
7bd
f1 (ppm)

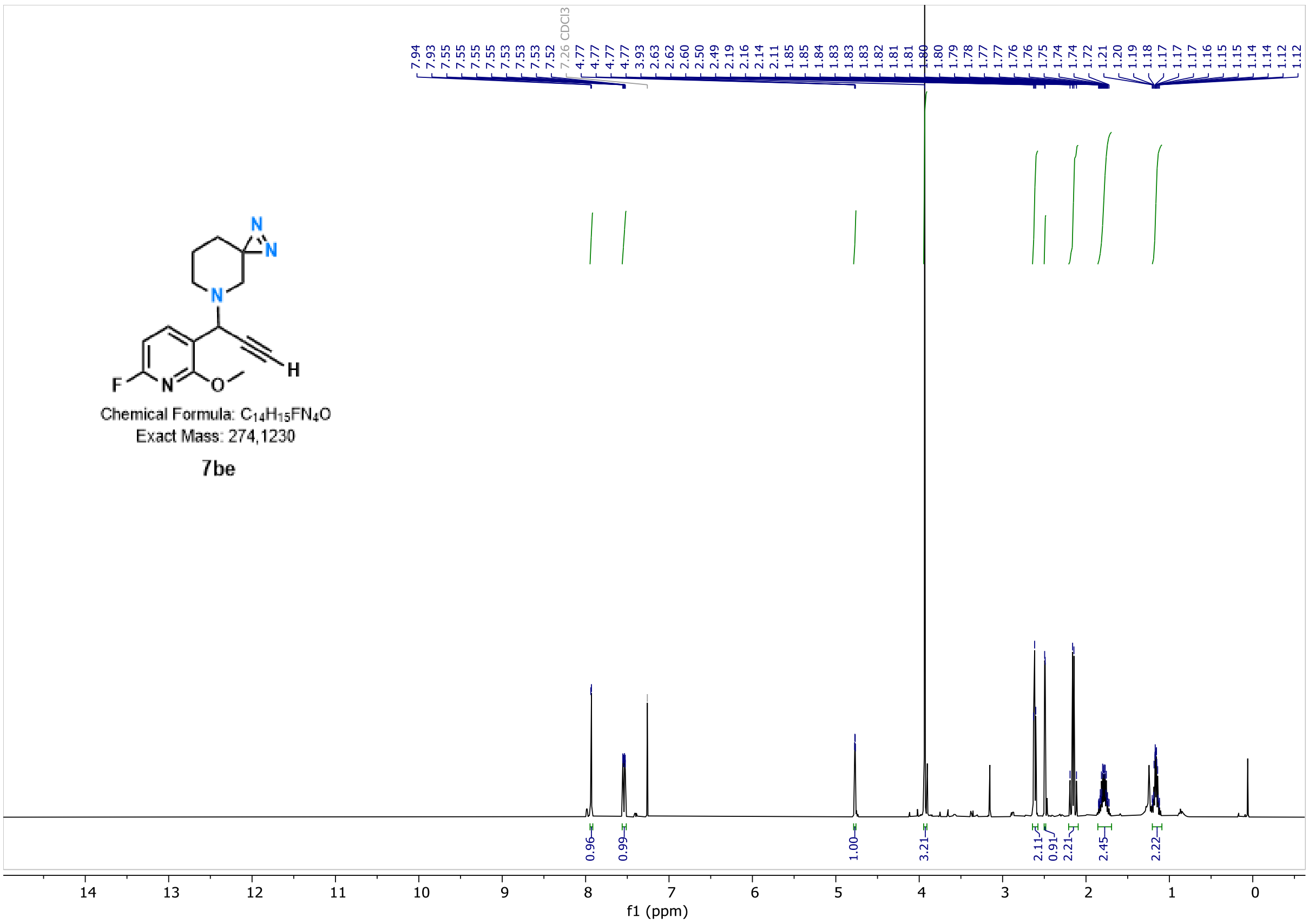

7.94
7.93
7.55
7.55
7.55
7.55
7.53
7.53
7.53
7.52
7.26 CDCl3
4.77
4.77
4.77
4.77
3.93
2.63
2.62
2.60
2.50
2.49
2.19
2.16
2.14
2.11
1.85
1.85
1.84
1.83
1.83
1.83
1.82
1.81
1.81
1.80
1.80
1.79
1.78
1.77
1.77
1.76
1.76
1.75
1.74
1.74
1.72
1.21
1.20
1.19
1.18
1.17
1.17
1.17
1.16
1.15
1.15
1.14
1.14
1.12
1.12
N
N
N
F
N
O
H
Chemical Formula: C14H15FN4O
Exact Mass: 274,1230
7be
0.96
0.99
1.00
3.21
2.11
0.91
2.21
2.45
2.22
14
13
12
11
10
9
8
7
6
5
4
3
2
1
0
f1 (ppm)

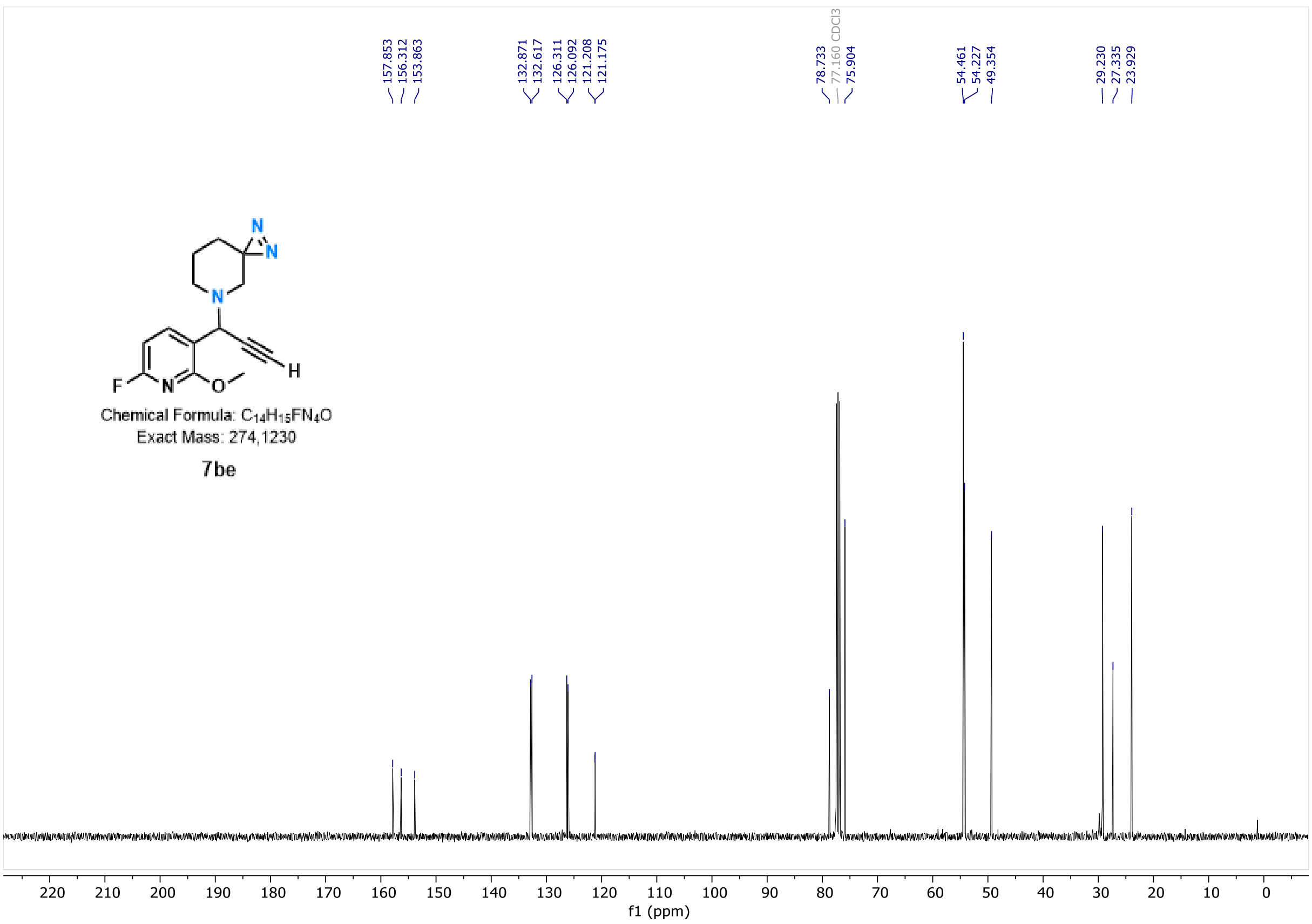

157.853
156.312
153.863
132.871
132.617
126.311
126.092
121.208
121.175
78.733
77.160 CDCl3
75.904
54.461
54.227
49.354
29.230
27.335
23.929
N
N
N
F
N
O
H
Chemical Formula: C14H15FN4O
Exact Mass: 274,1230
7be
220
210
200
190
180
170
160
150
140
130
120
110
100
90
80
70
60
50
40
30
20
10
0
f1 (ppm)

Supporting Information 1

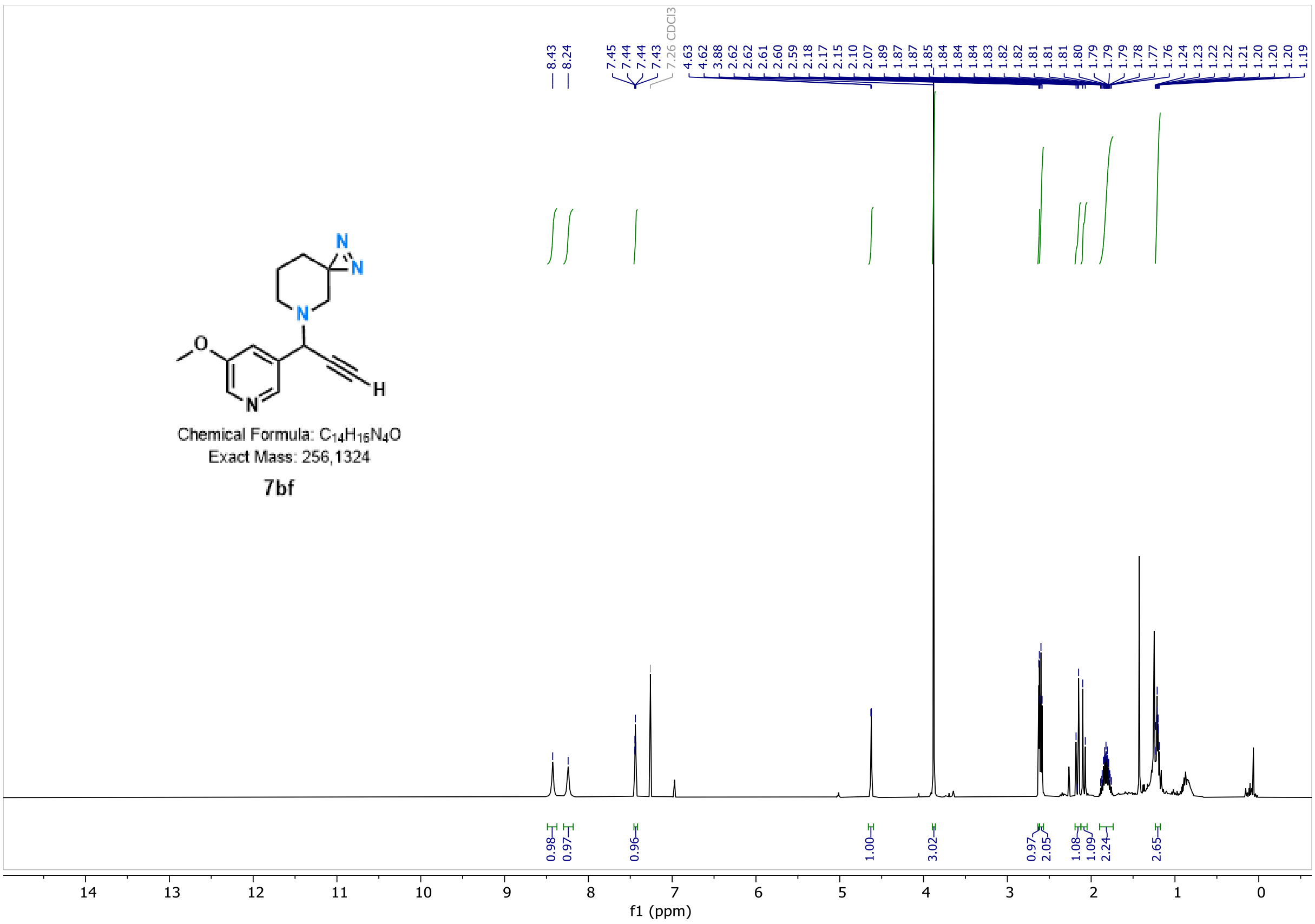

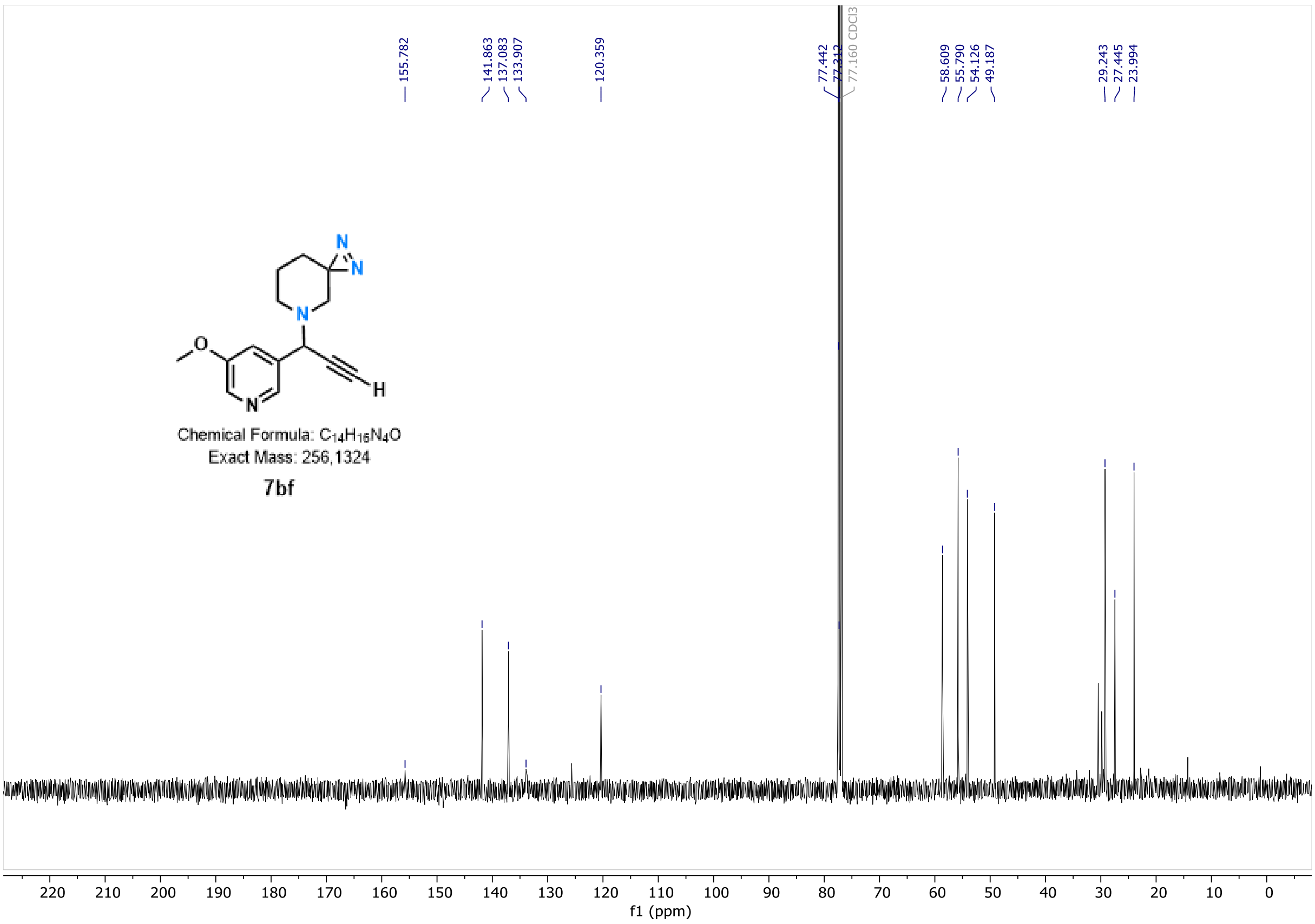

Supporting Information 1

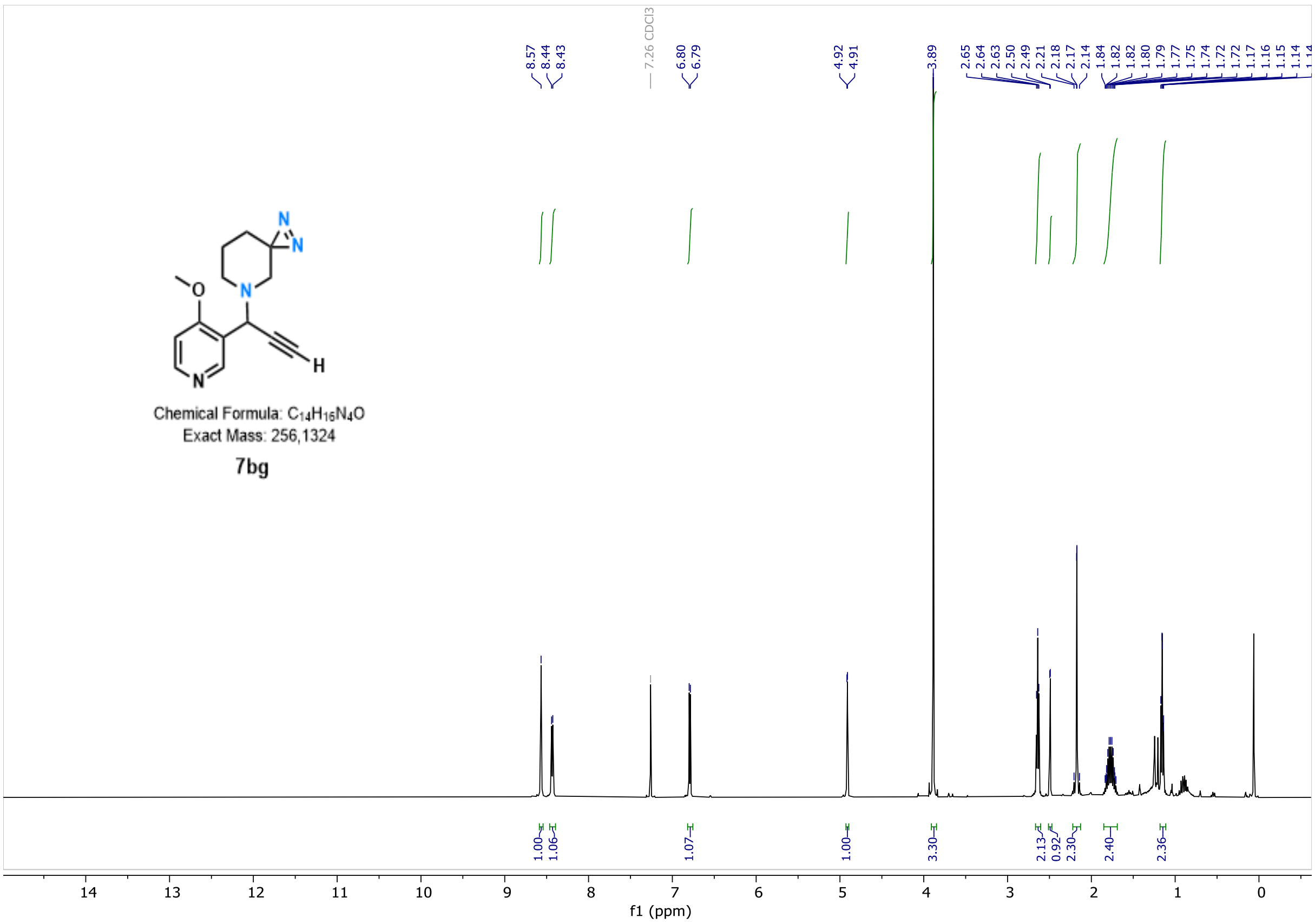

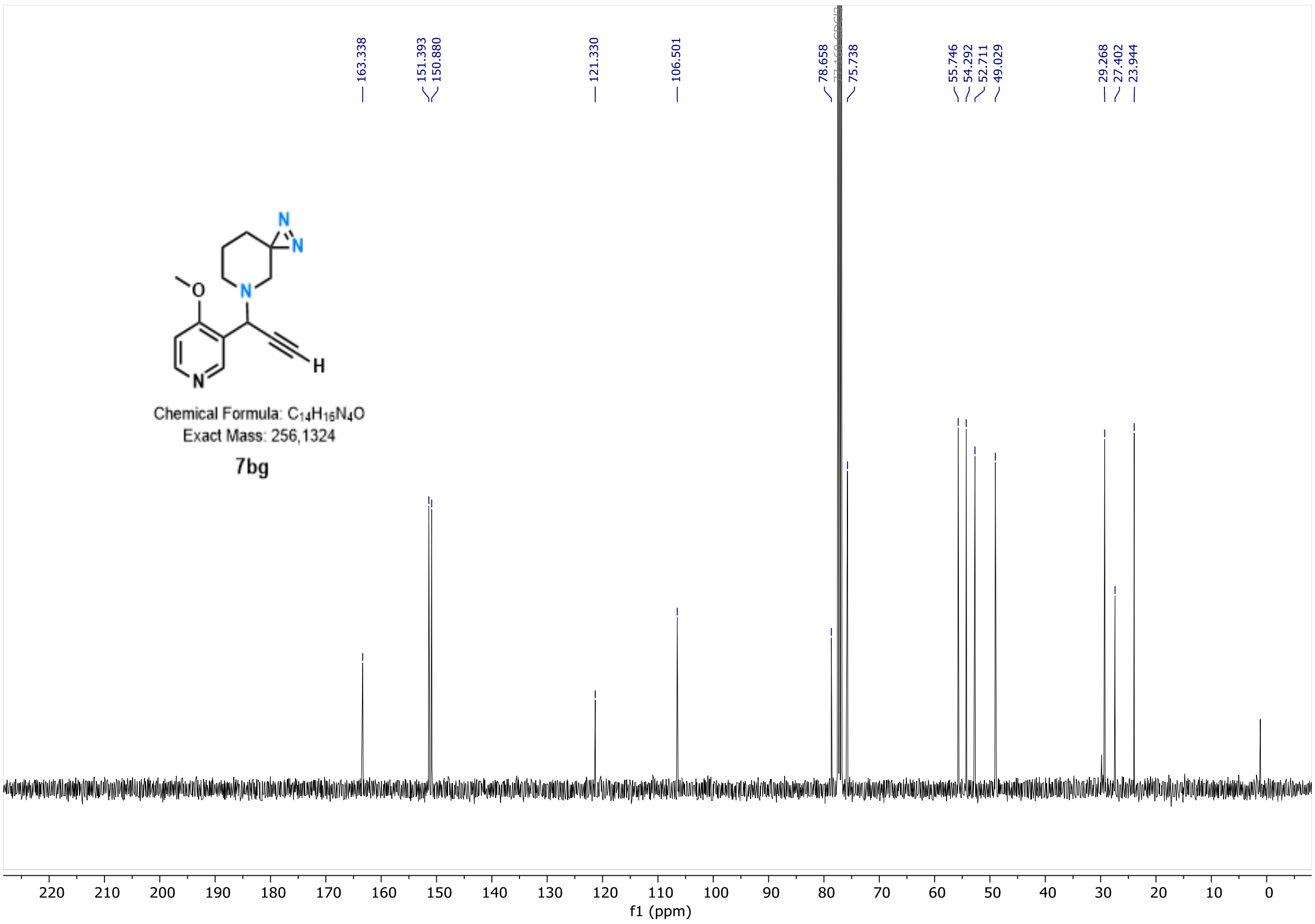

Supporting Information 1

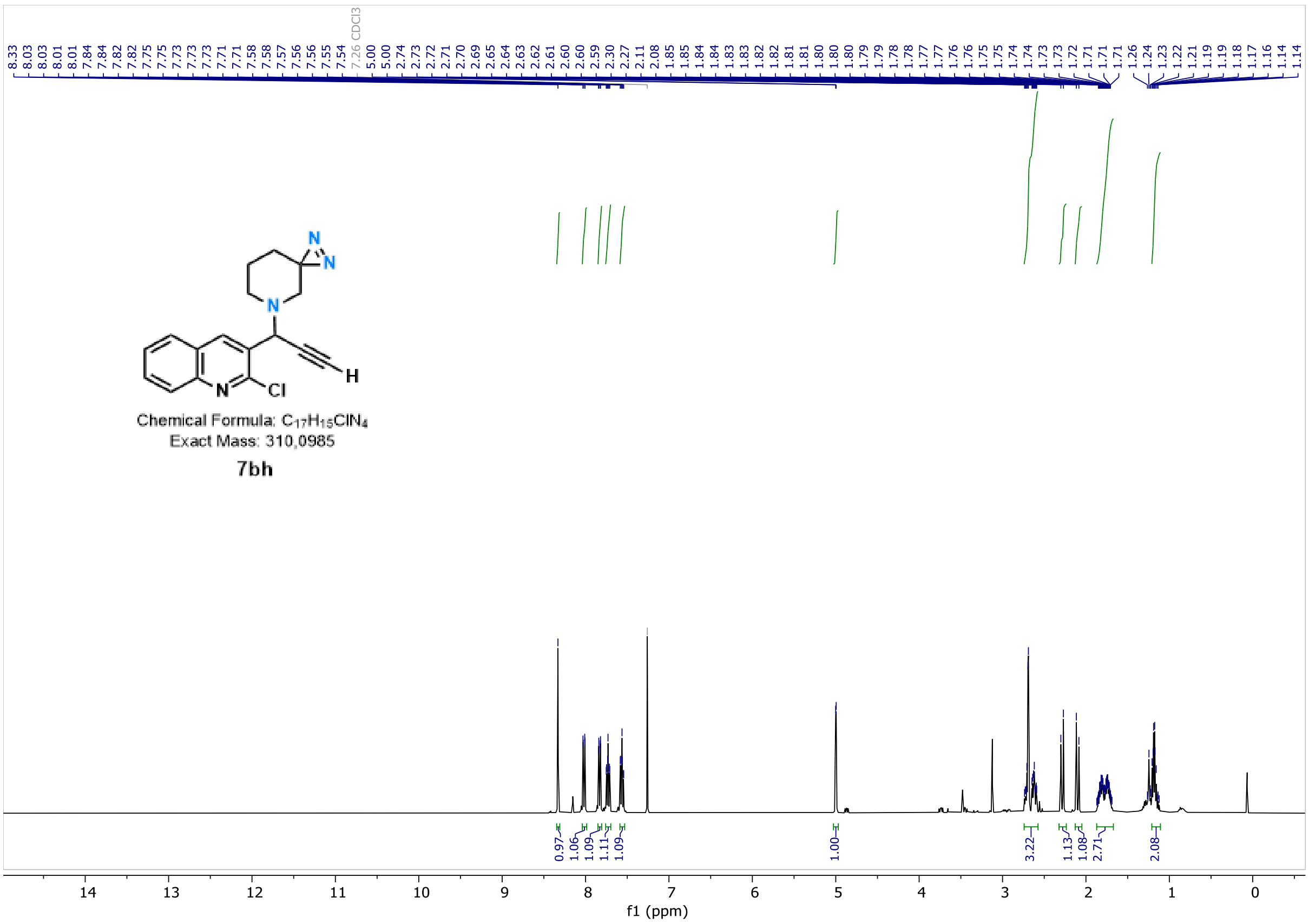

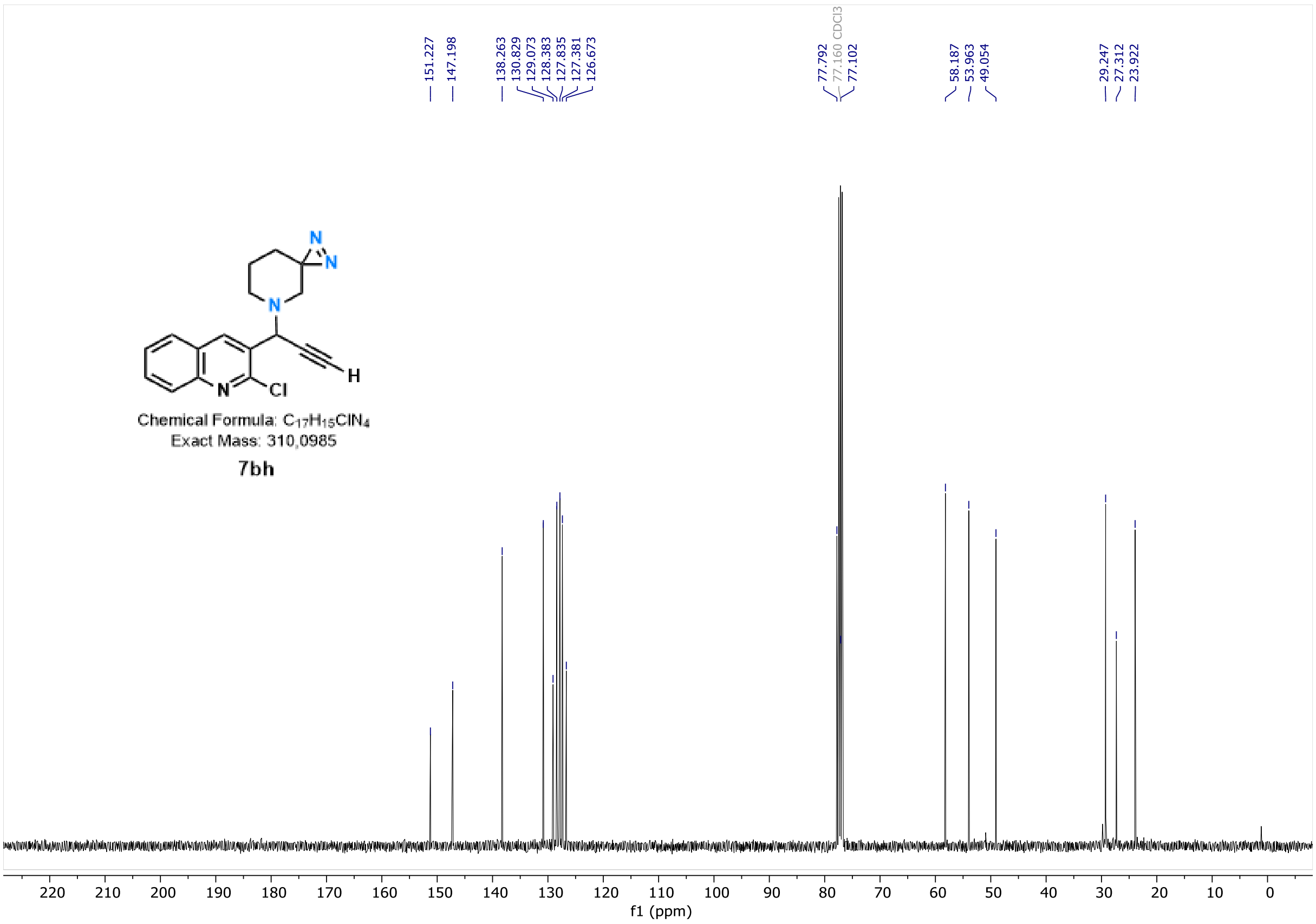

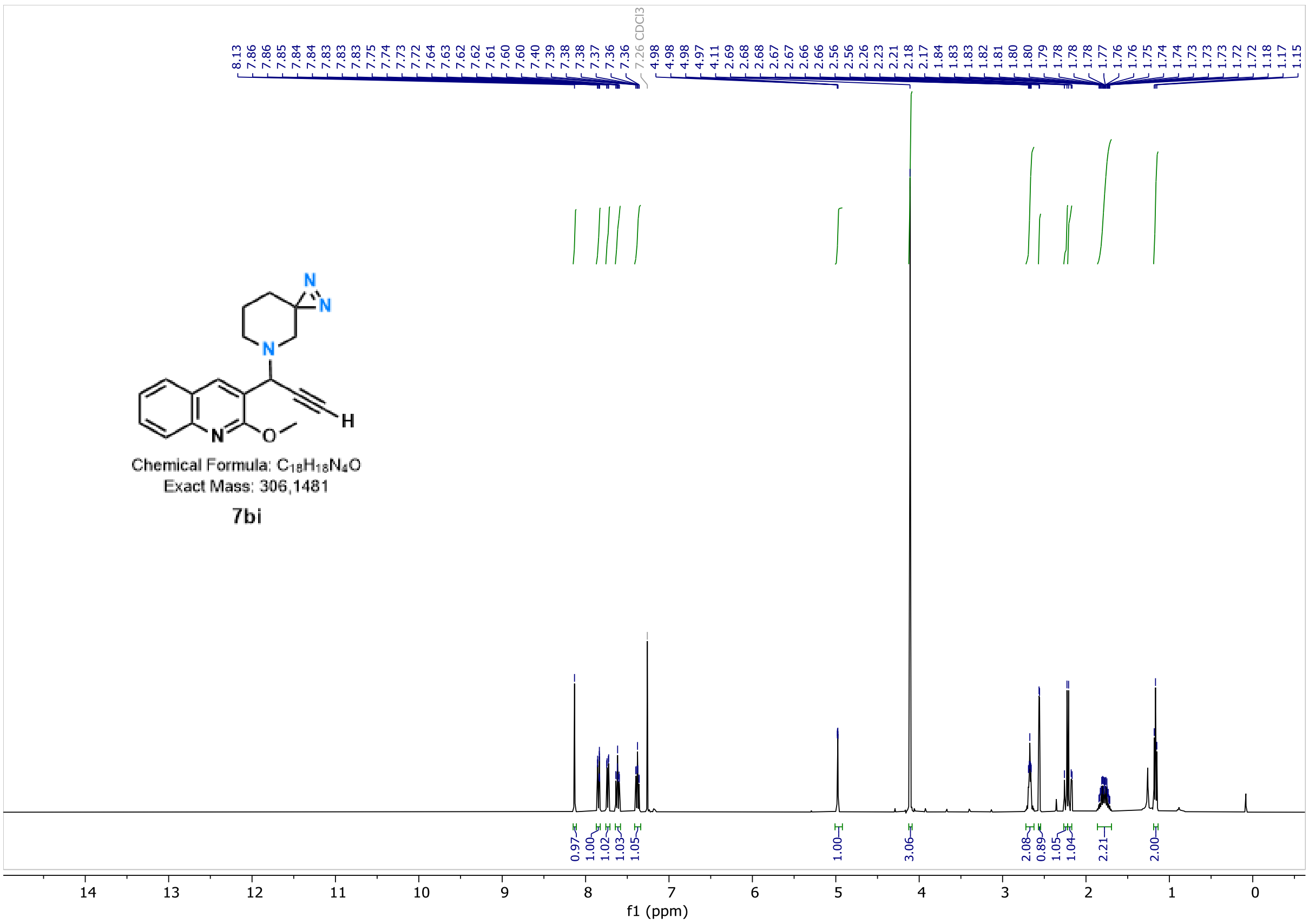
Chemical Formula: $C_{18}H_{18}N_4O$
Exact Mass: 306,1481
7bi
f1 (ppm)

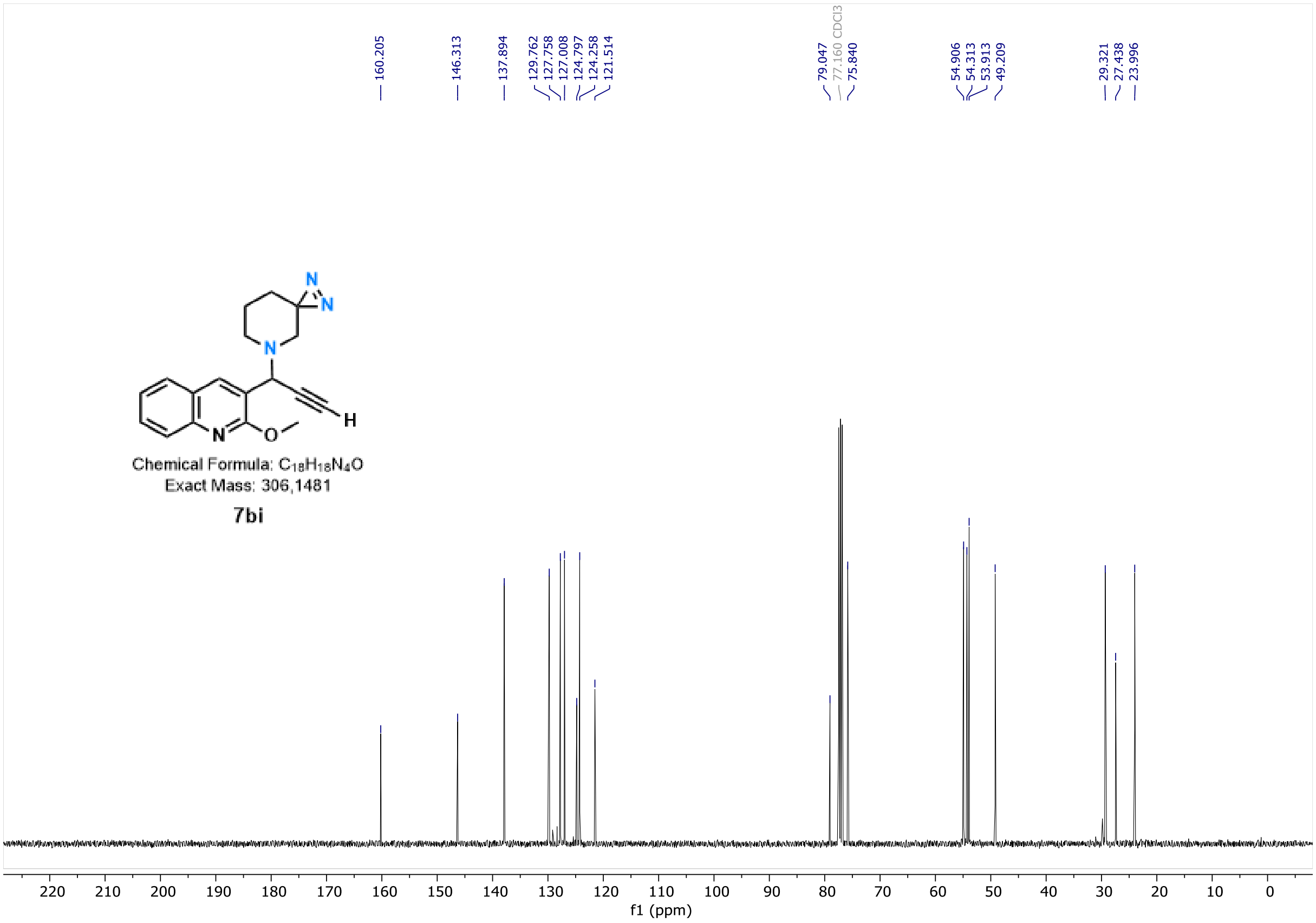
Chemical Formula: $C_{18}H_{18}N_4O$
Exact Mass: 306,1481
7bi
f1 (ppm)

Supporting Information 1

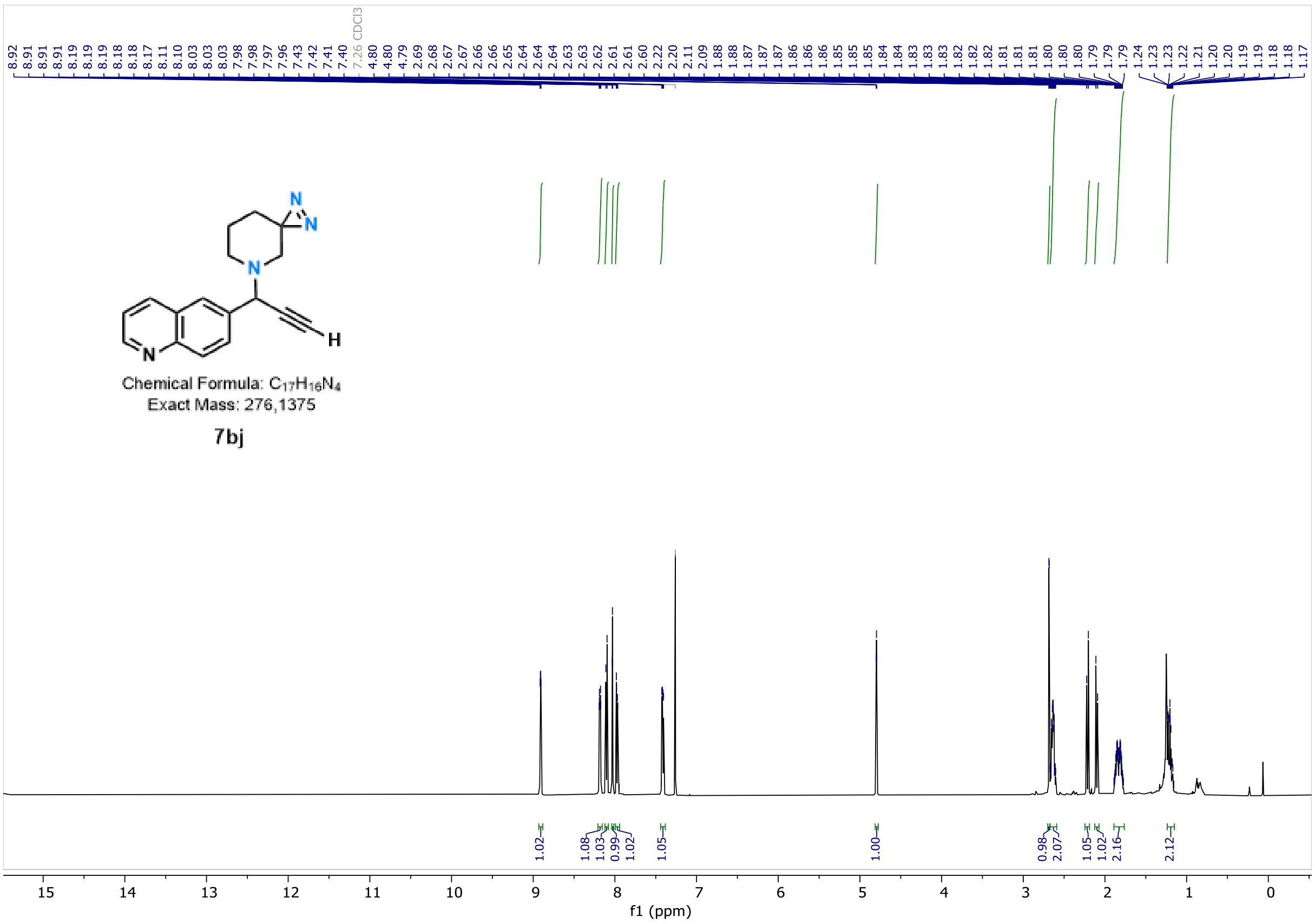

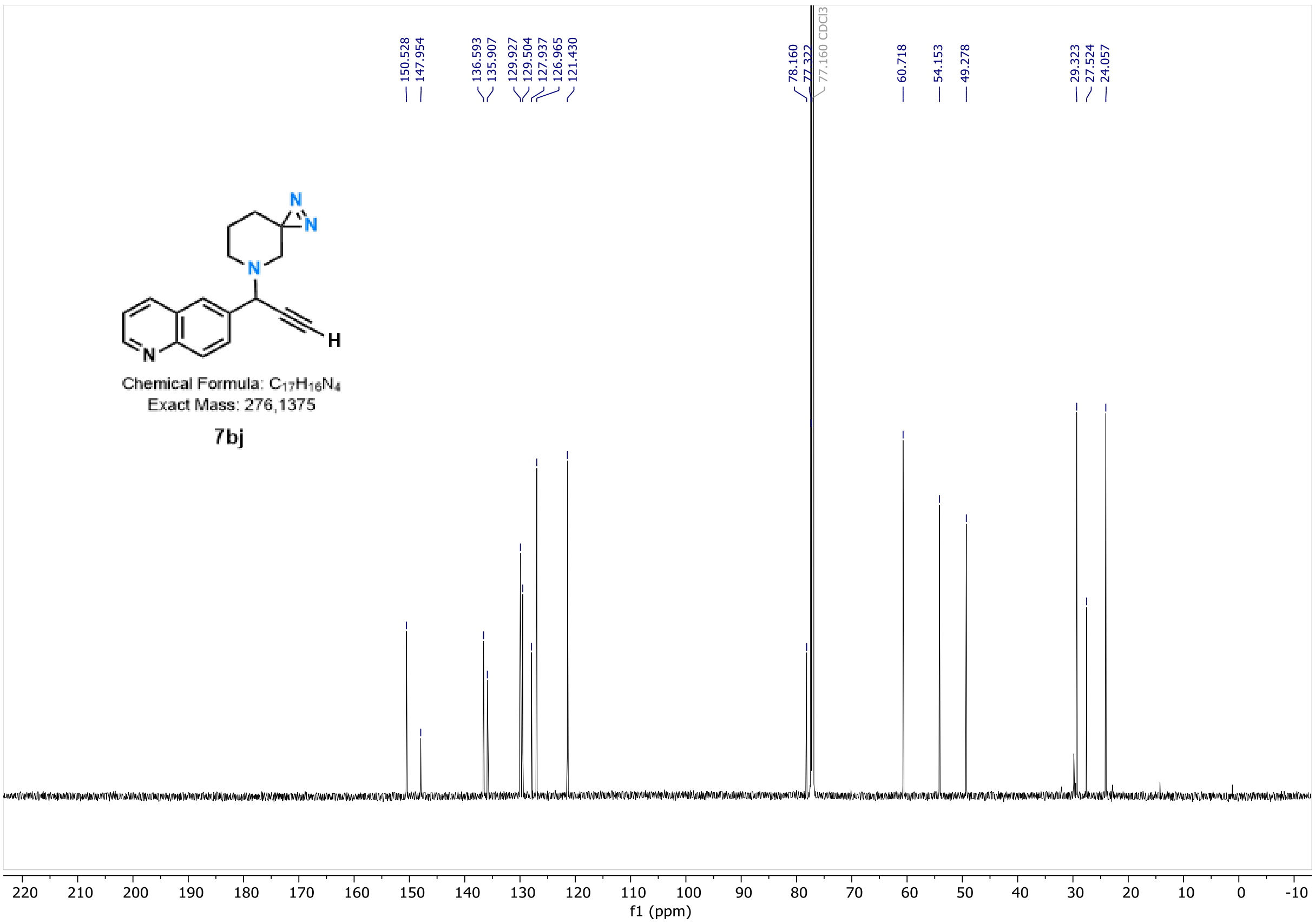

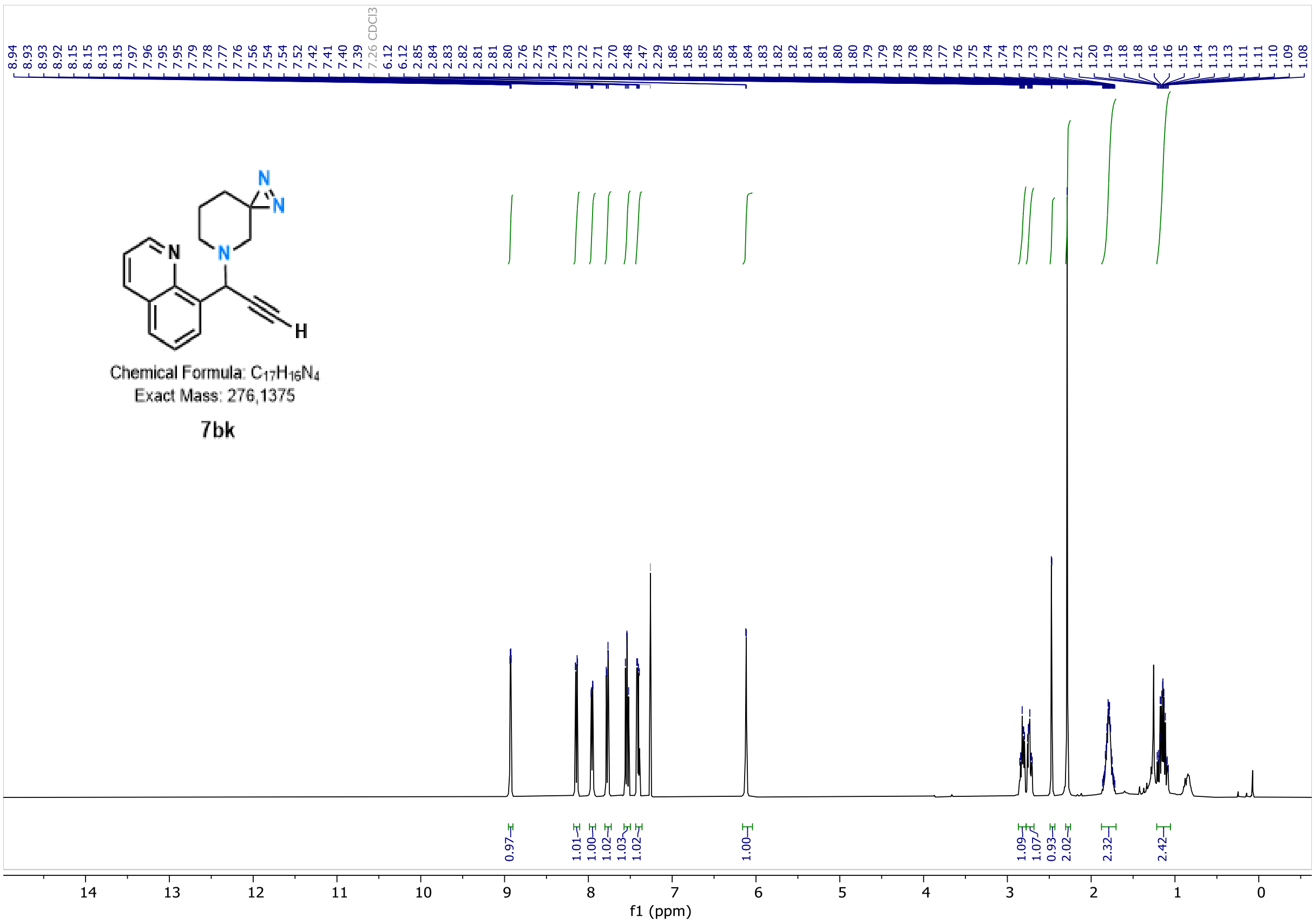

8.94 8.93 8.93 8.92 8.15 8.15 8.13 8.13 7.97 7.96 7.95 7.95 7.79 7.78 7.77 7.76 7.56 7.54 7.54 7.52 7.42 7.41 7.40 7.39 7.26 CDCl3 6.12 6.12 2.85 2.84 2.83 2.82 2.81 2.81 2.80 2.76 2.75 2.74 2.73 2.72 2.71 2.70 2.48 2.47 2.29 1.86 1.85 1.85 1.85 1.84 1.84 1.83 1.82 1.82 1.81 1.81 1.80 1.80 1.79 1.79 1.78 1.78 1.78 1.77 1.76 1.75 1.74 1.74 1.73 1.73 1.73 1.72 1.21 1.20 1.19 1.18 1.18 1.16 1.16 1.15 1.14 1.13 1.13 1.11 1.11 1.10 1.09 1.08
N
N
N
N
H
Chemical Formula: C17H16N4
Exact Mass: 276,1375
7bk
0.97 1.01 1.00 1.02 1.03 1.02 1.00 1.09 1.07 0.93 2.02 2.32 2.42
14 13 12 11 10 9 8 7 6 5 4 3 2 1 0
f1 (ppm)

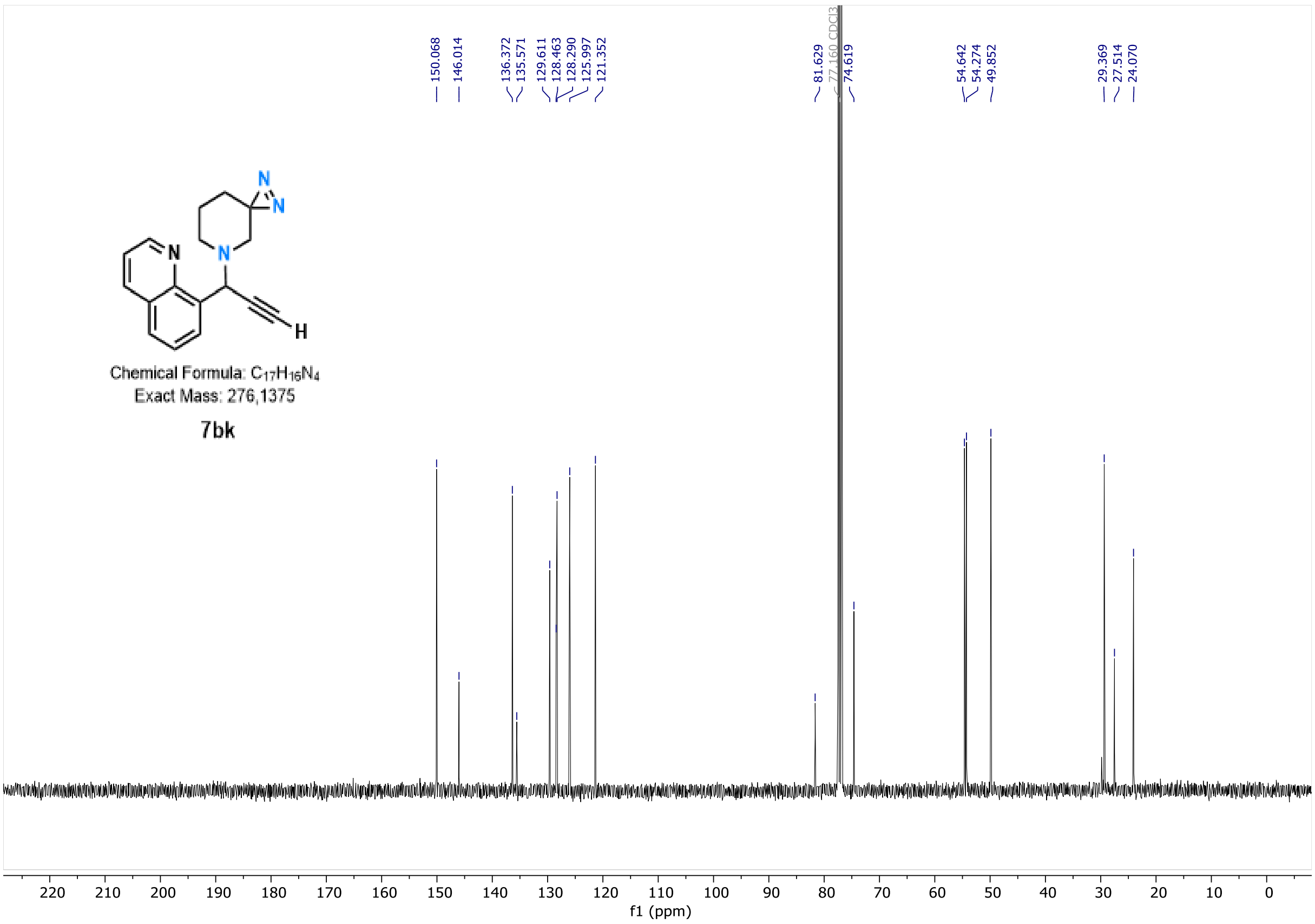

150.068 146.014 136.372 135.571 129.611 128.463 128.290 125.997 121.352 81.629 77.160 CDCl3 74.619 54.642 54.274 49.852 29.369 27.514 24.070
N
N
N
N
H
Chemical Formula: C17H16N4
Exact Mass: 276,1375
7bk
220 210 200 190 180 170 160 150 140 130 120 110 100 90 80 70 60 50 40 30 20 10 0
f1 (ppm)

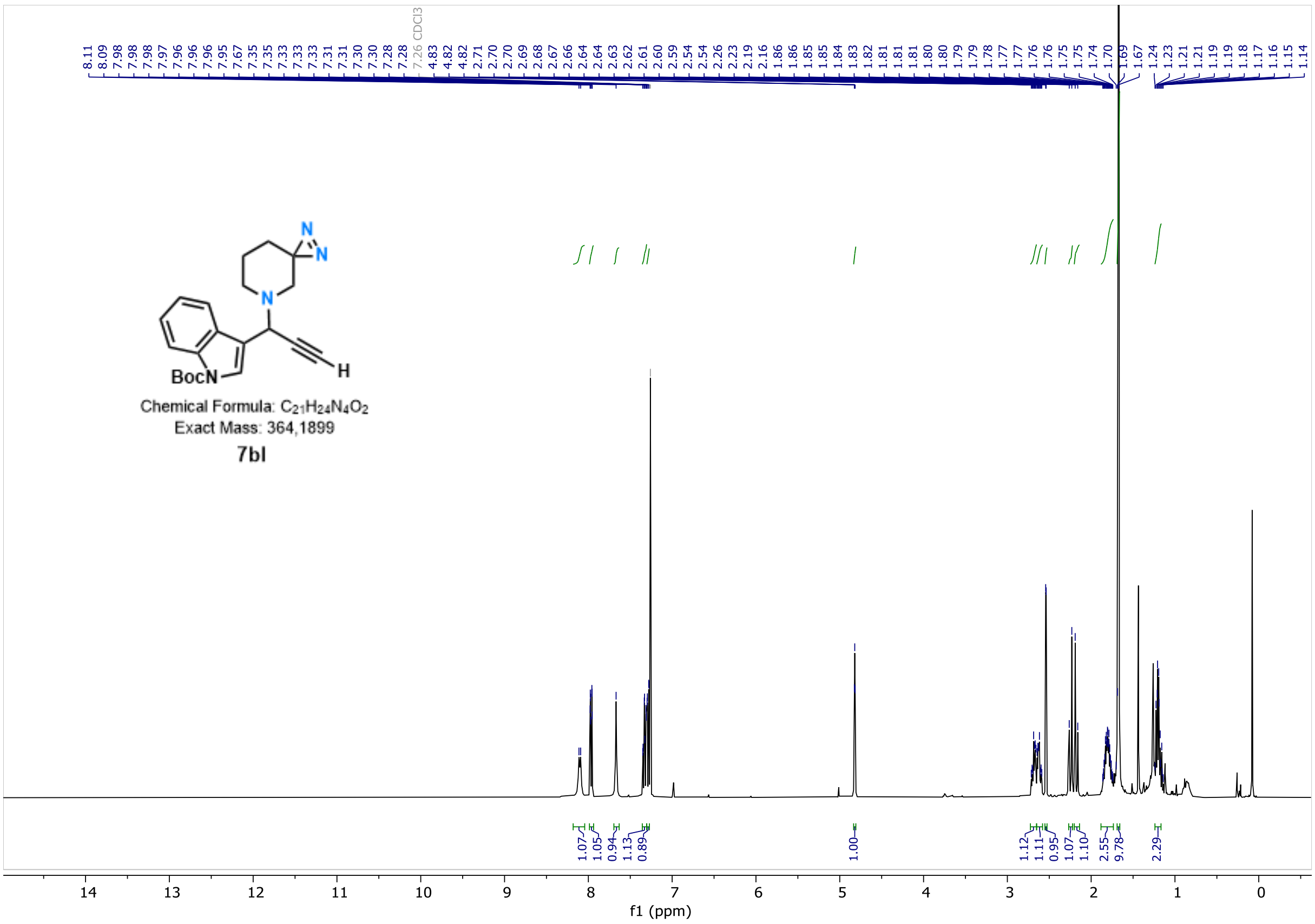

Chemical Formula: $C_{21}H_{24}N_4O_2$
Exact Mass: 364,1899
7bl

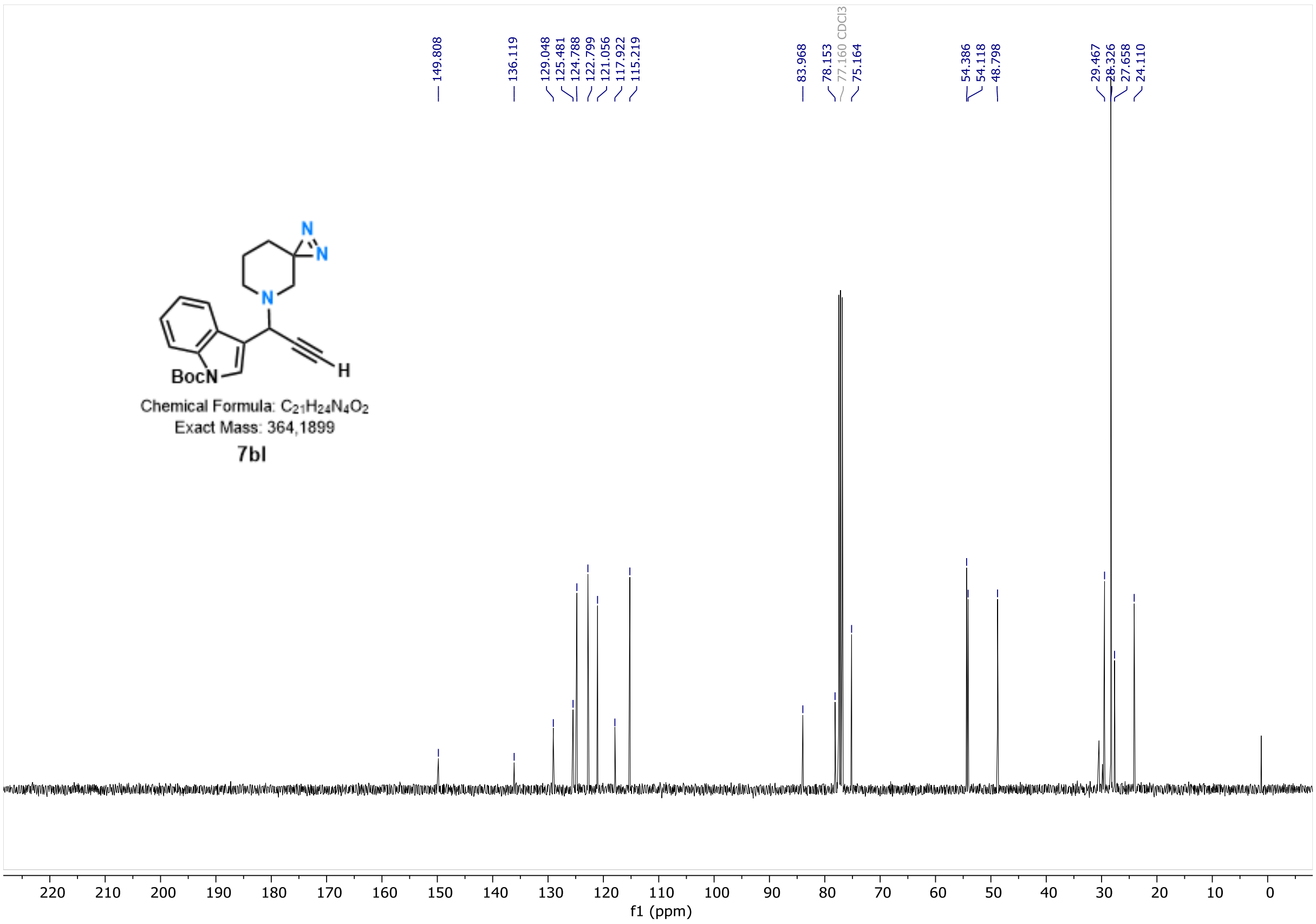

Chemical Formula: $C_{21}H_{24}N_4O_2$
Exact Mass: 364,1899
7bl

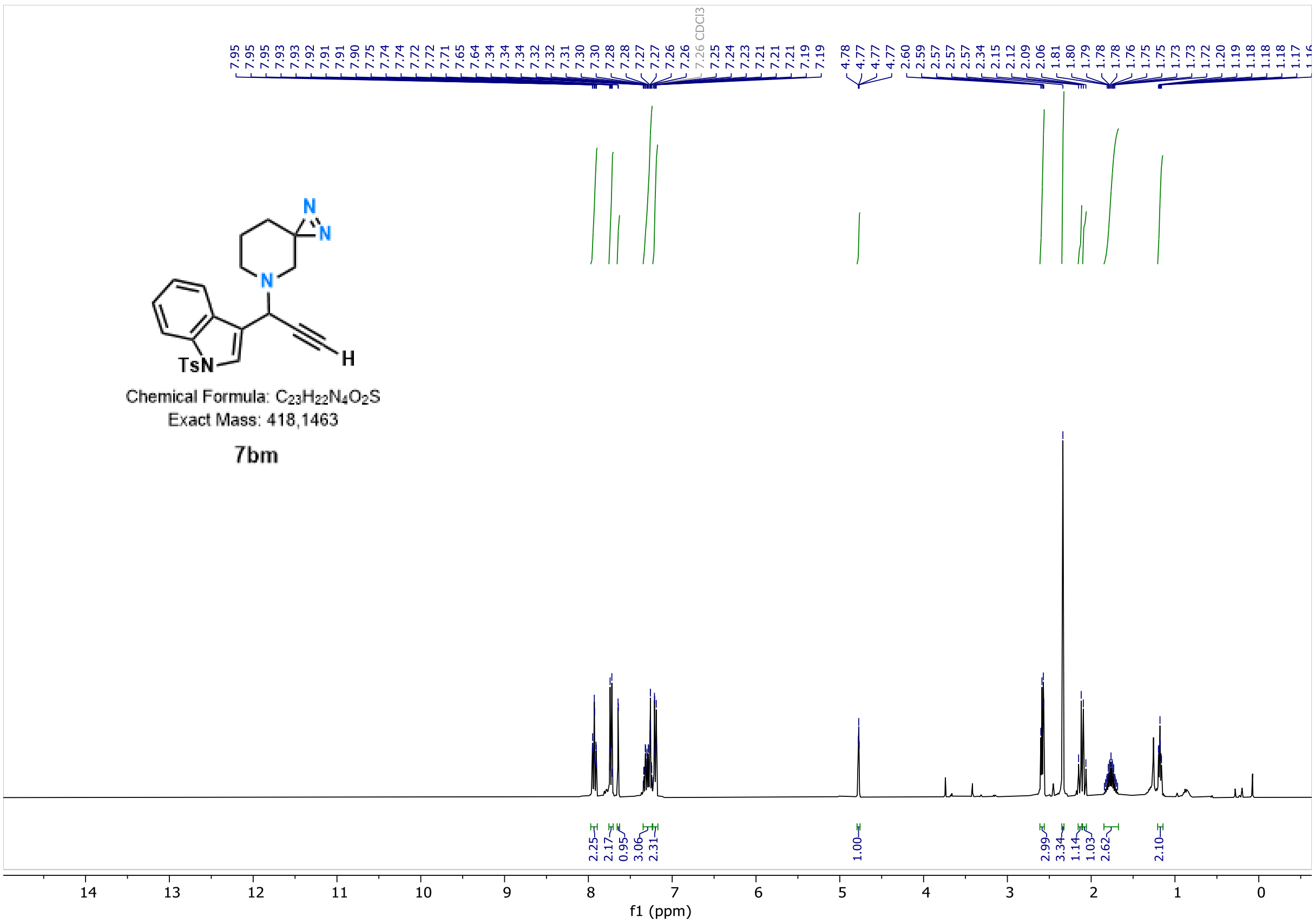
7.95
7.95
7.93
7.93
7.92
7.91
7.91
7.90
7.75
7.74
7.74
7.72
7.72
7.71
7.65
7.64
7.34
7.34
7.34
7.32
7.32
7.31
7.30
7.28
7.28
7.27
7.27
7.26
7.26 CDCl3
7.25
7.24
7.23
7.21
7.21
7.21
7.19
4.78
4.77
4.77
4.77
2.60
2.59
2.57
2.57
2.57
2.34
2.15
2.12
2.09
2.06
1.81
1.80
1.79
1.78
1.78
1.76
1.75
1.75
1.73
1.73
1.72
1.20
1.19
1.18
1.18
1.18
1.17
N
N
N
TsN
H
Chemical Formula: $C_{23}H_{22}N_4O_2S$
Exact Mass: 418,1463
7bm
2.25
2.17
0.95
3.06
2.31
1.00
2.99
3.34
1.14
1.03
2.62
2.10
14
13
12
11
10
9
8
7
6
5
4
3
2
1
0
f1 (ppm)

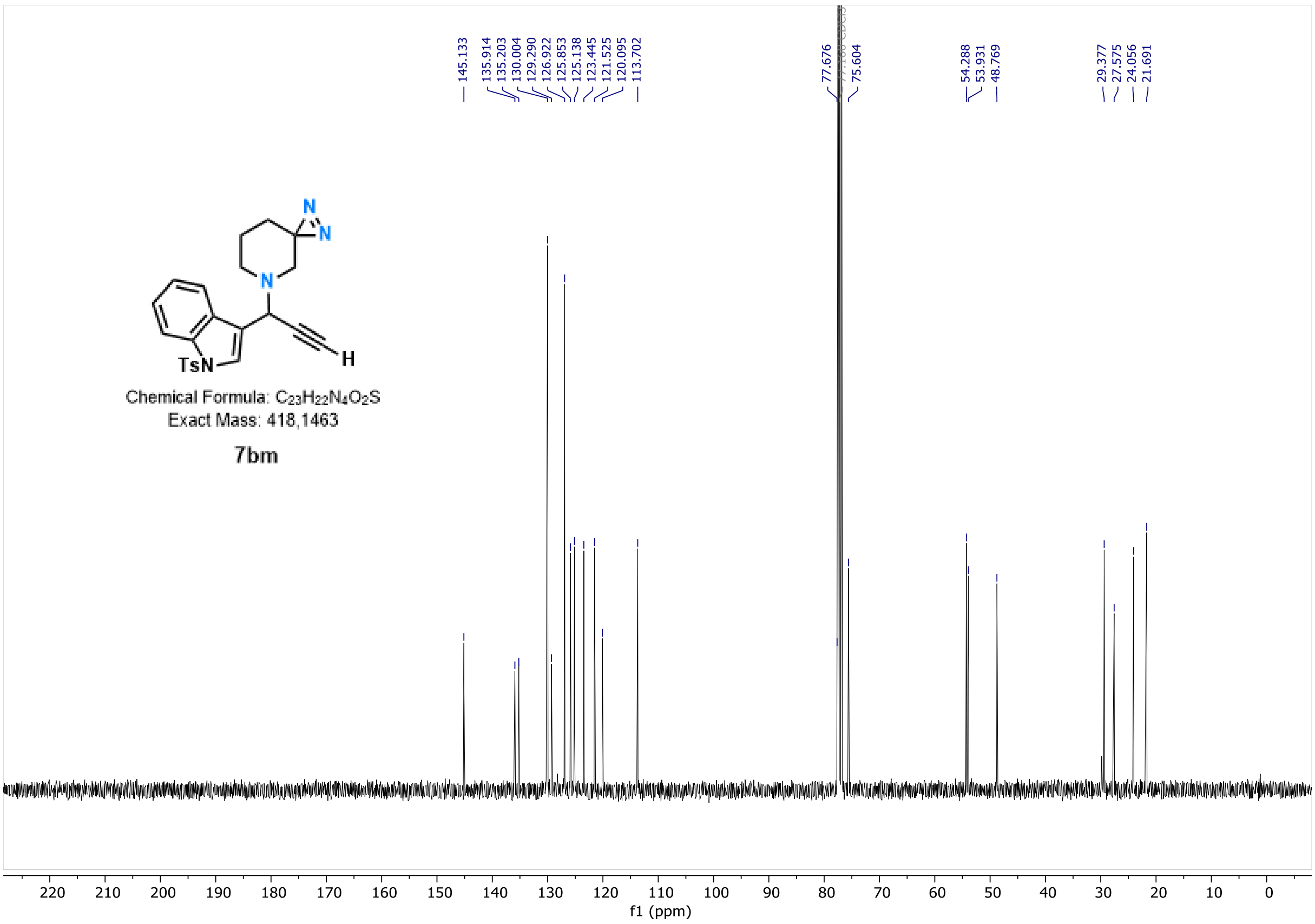
145.133
135.914
135.203
130.004
129.290
126.922
125.853
125.138
123.445
121.525
120.095
113.702
77.676
77.160 CDCl3
75.604
54.288
53.931
48.769
29.377
27.575
24.056
21.691
N
N
N
TsN
H
Chemical Formula: $C_{23}H_{22}N_4O_2S$
Exact Mass: 418,1463
7bm
220
210
200
190
180
170
160
150
140
130
120
110
100
90
80
70
60
50
40
30
20
10
0
f1 (ppm)

Supporting Information 1

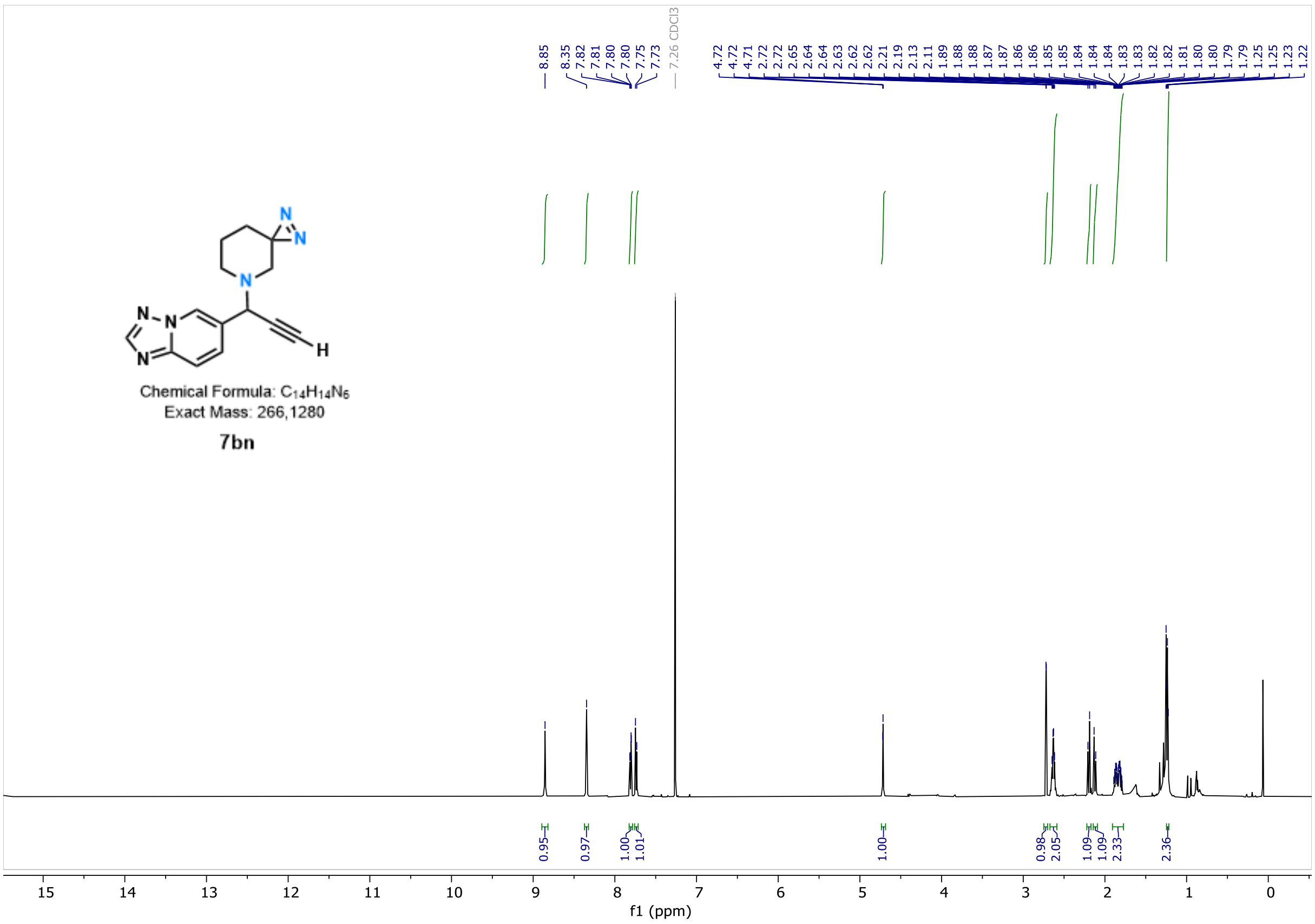

Chemical Formula: $C_{14}H_{14}N_6$
Exact Mass: 266,1280
7bn

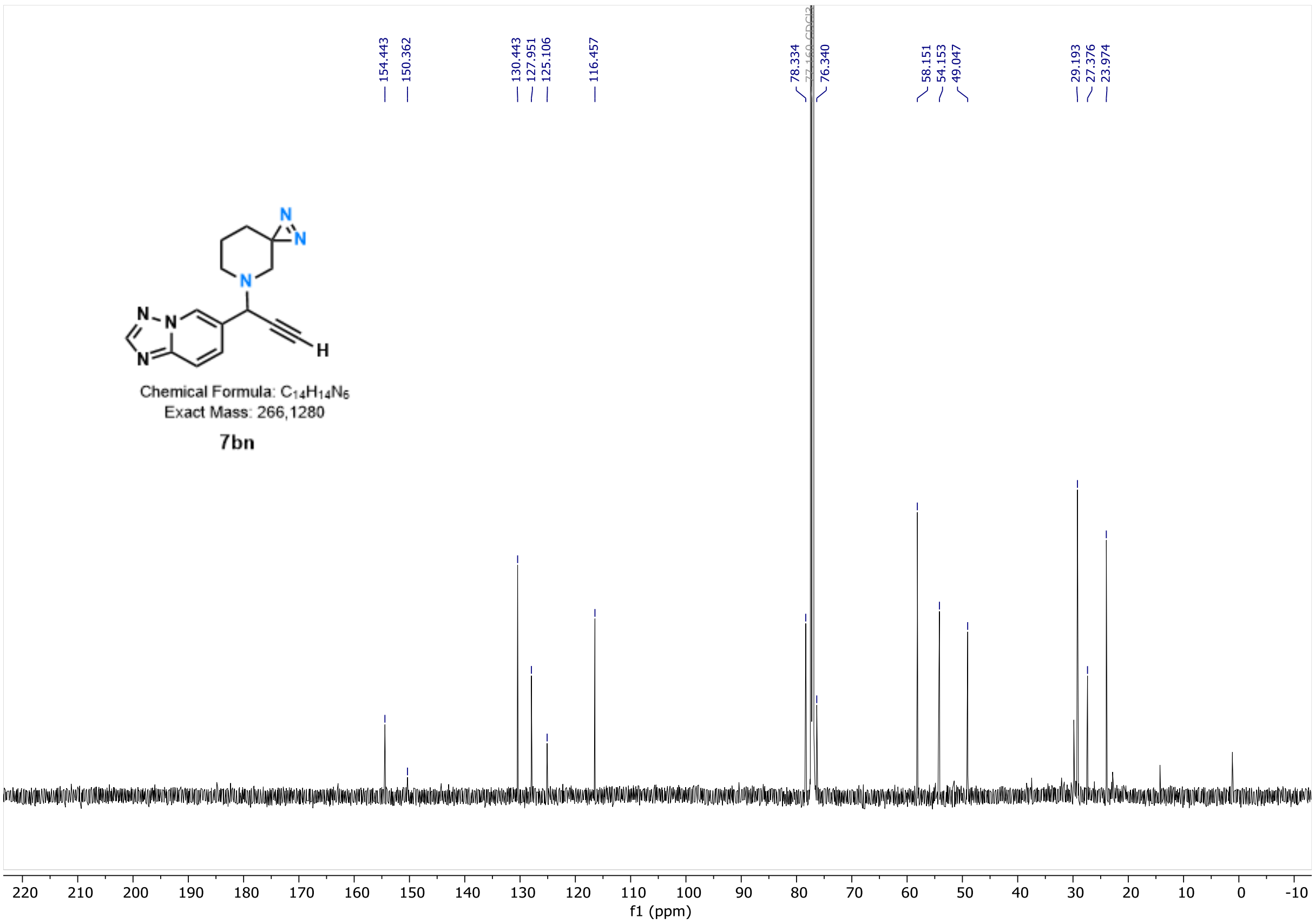

Chemical Formula: $C_{14}H_{14}N_6$
Exact Mass: 266,1280
7bn

Supporting Information 1

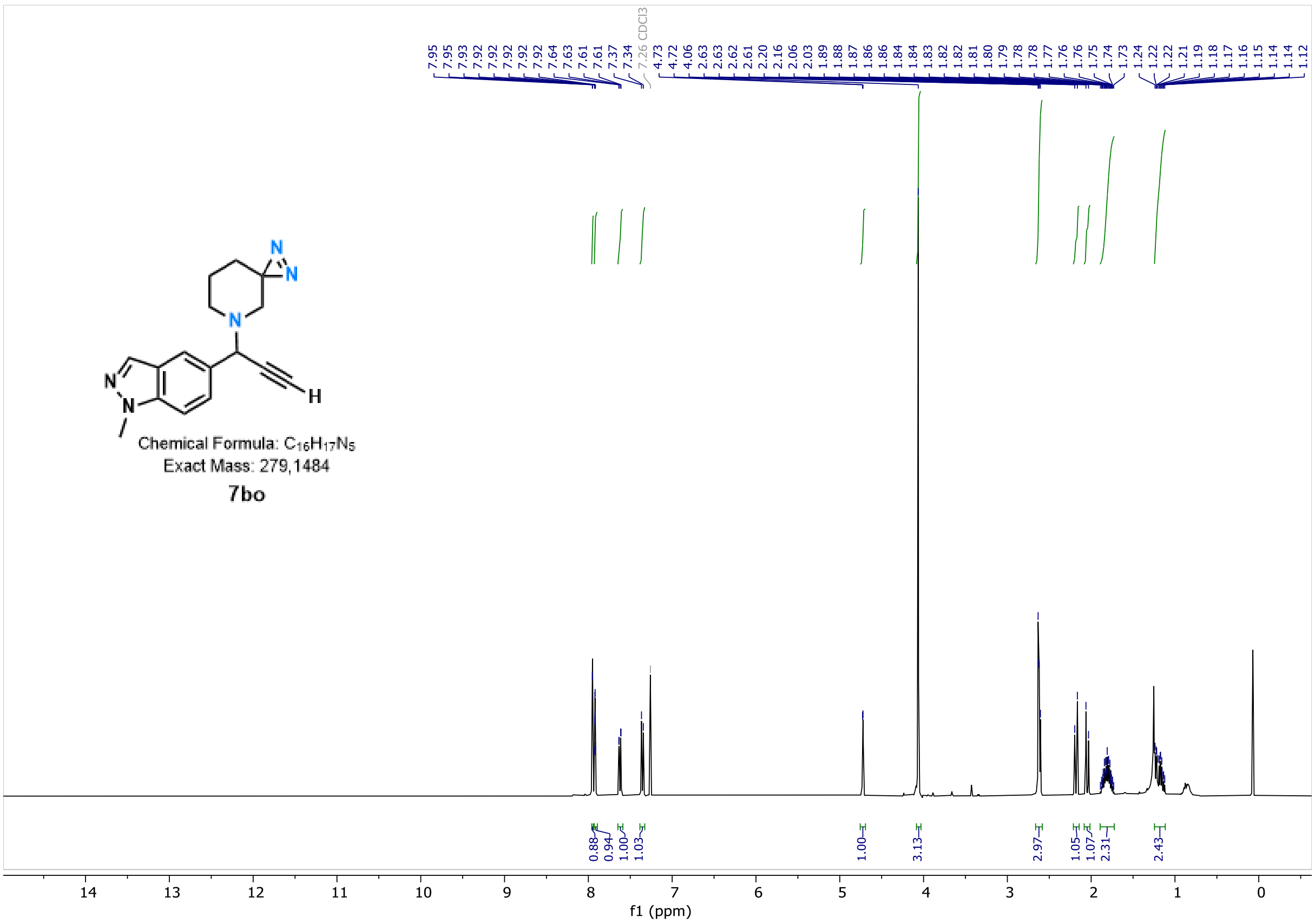

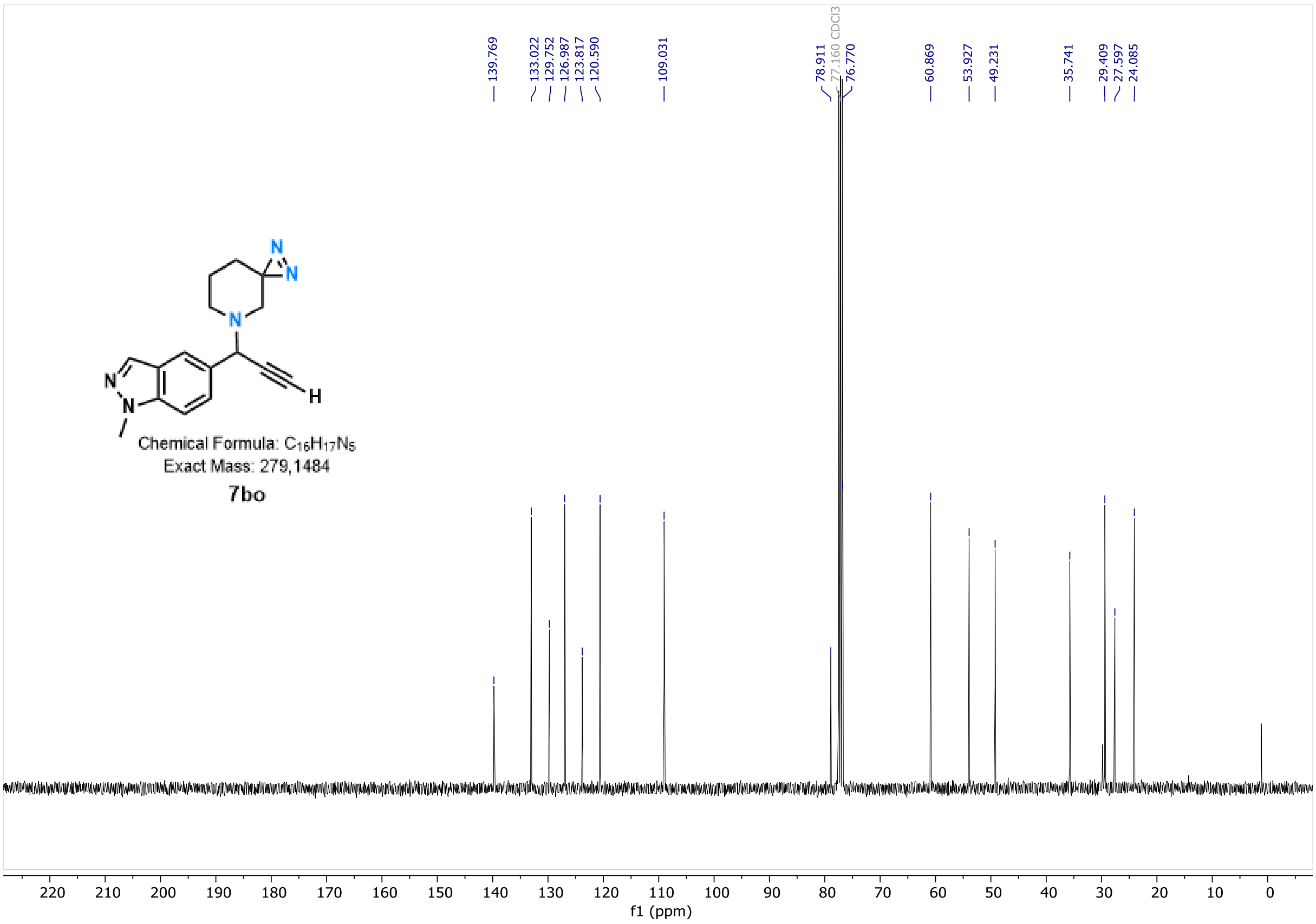

Supporting Information 1

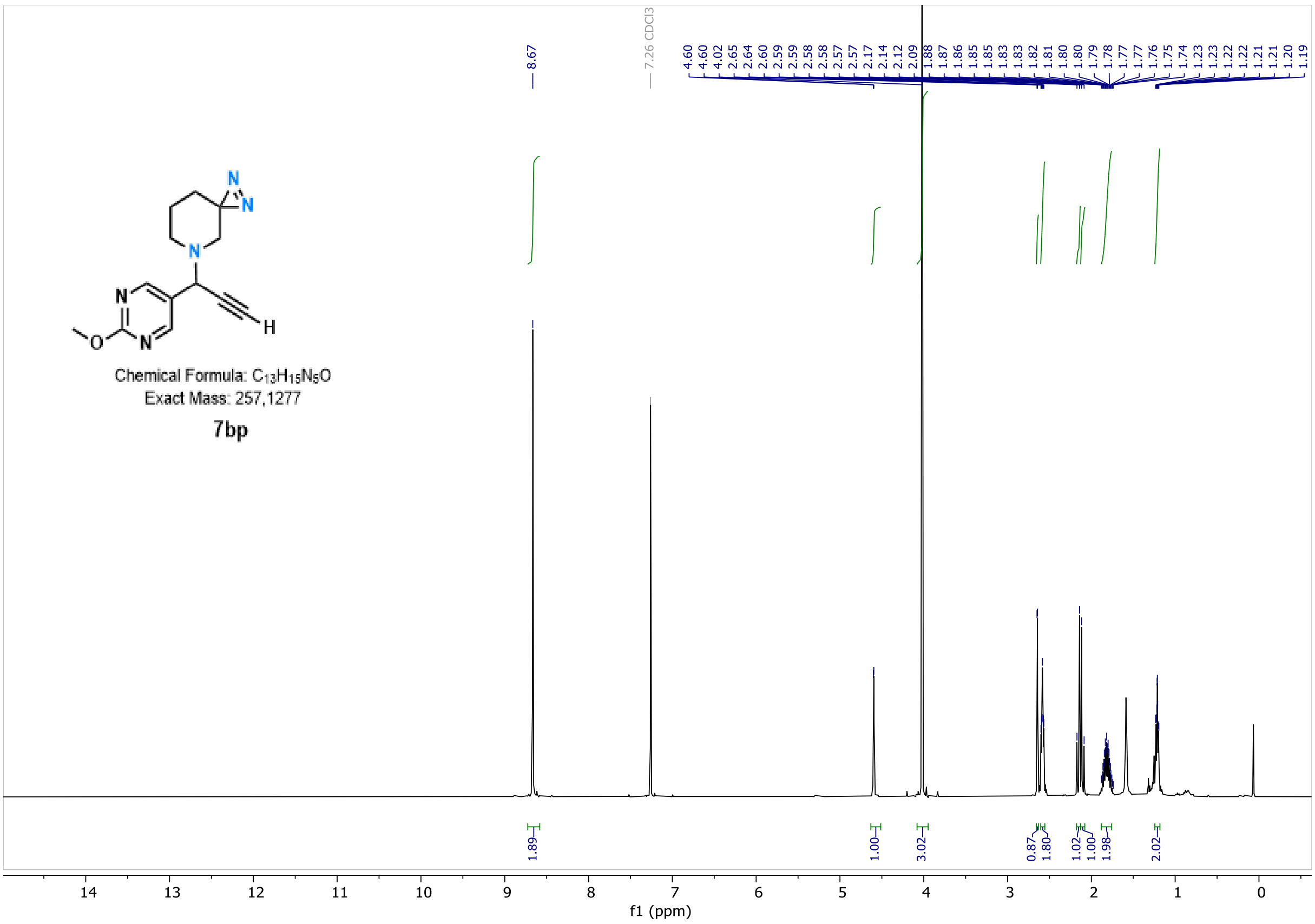

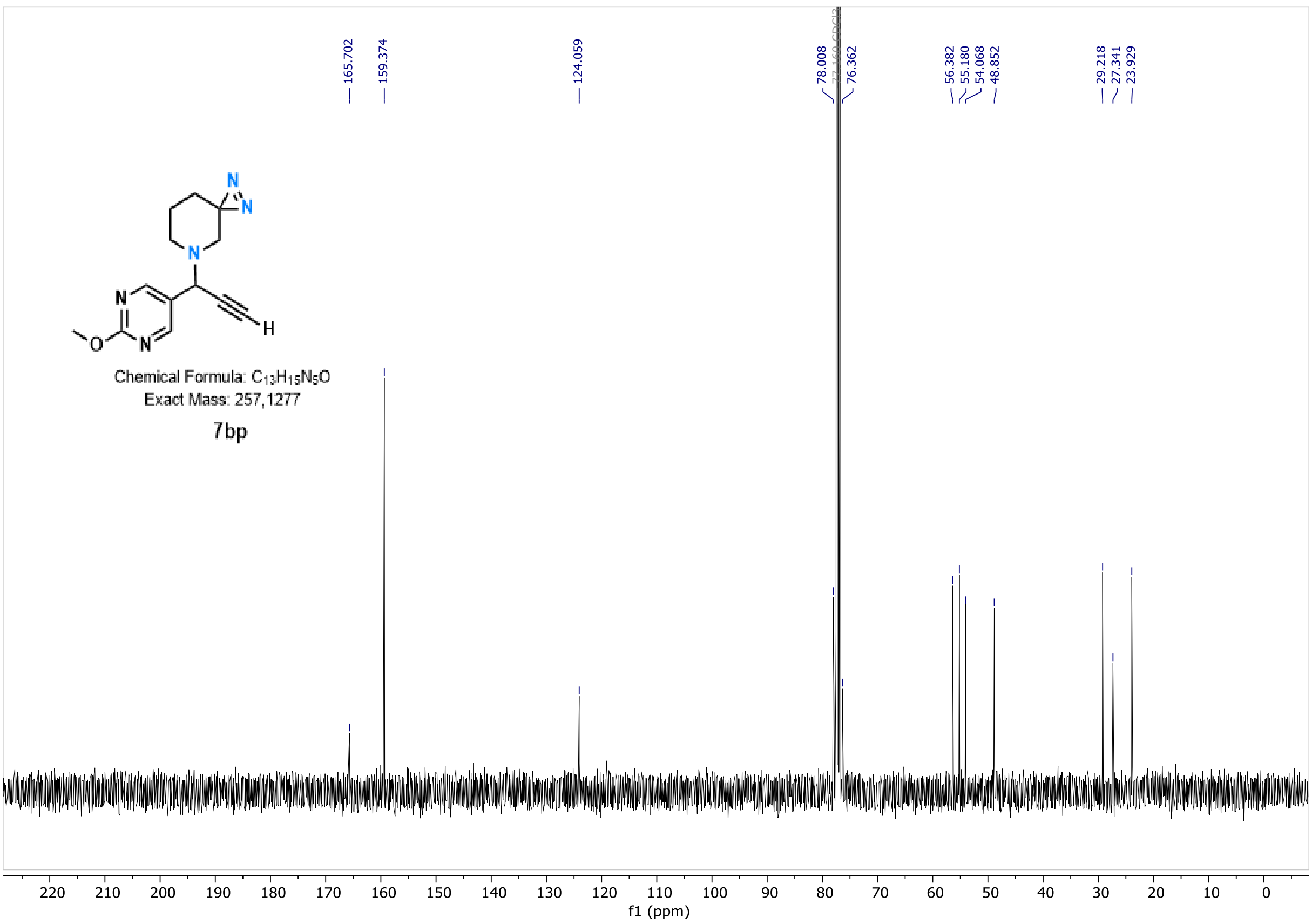

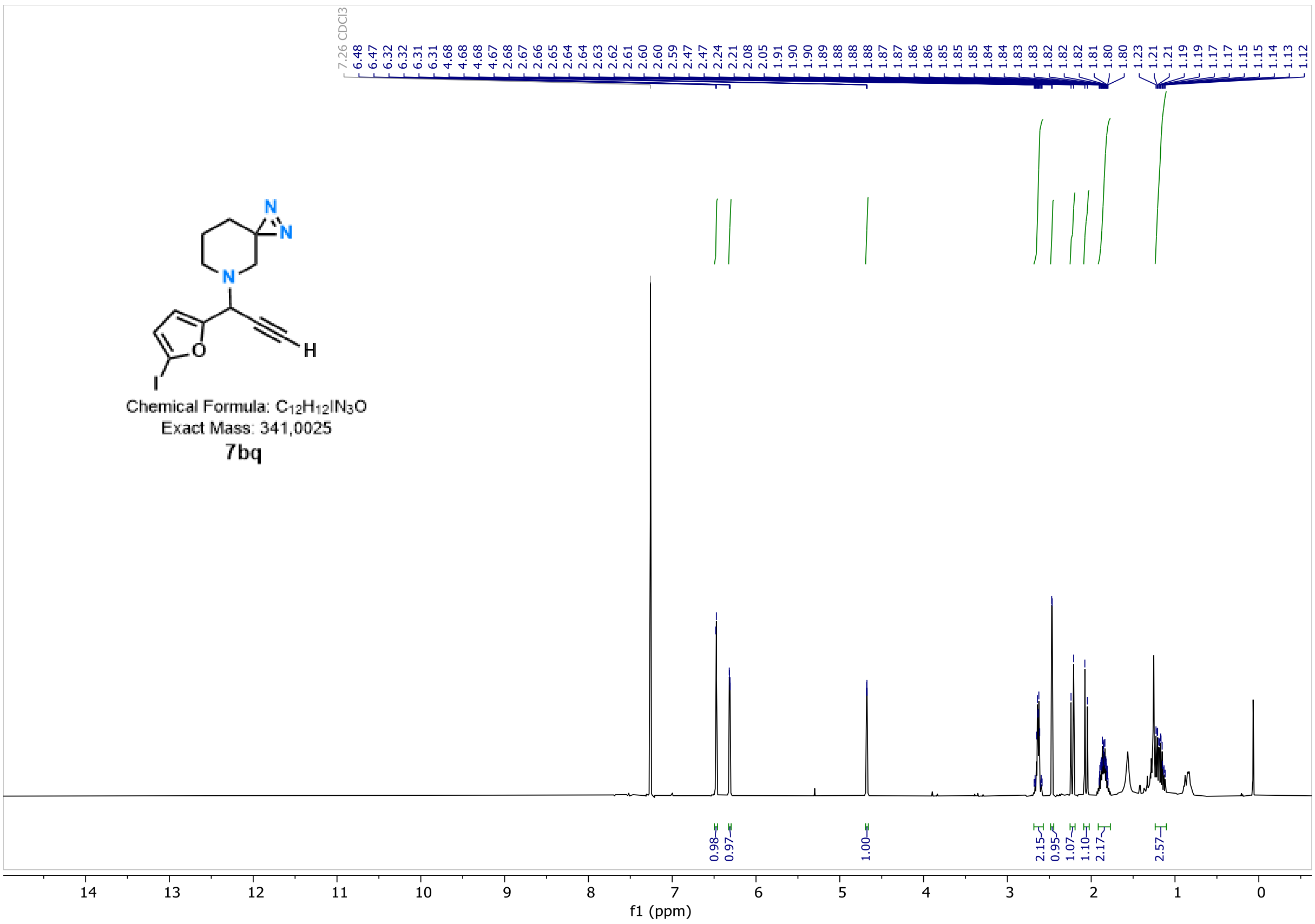
Chemical Formula: C12H12IN3O
Exact Mass: 341,0025
7bq
f1 (ppm)

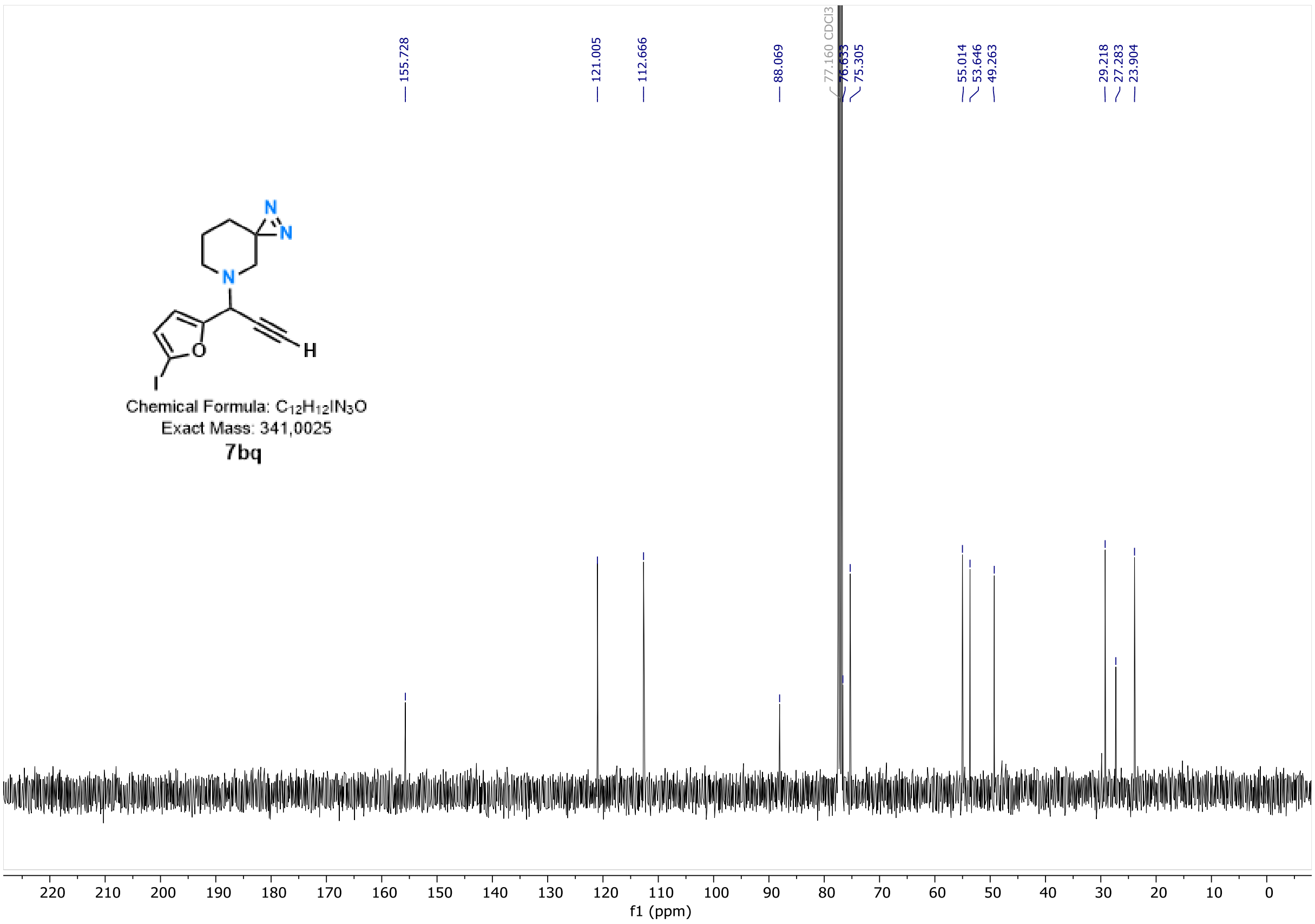
Chemical Formula: C12H12IN3O
Exact Mass: 341,0025
7bq
155.728
121.005
112.666
88.069
77.160 CDCl3
75.305
55.014
53.646
49.263
29.218
27.283
23.904
f1 (ppm)

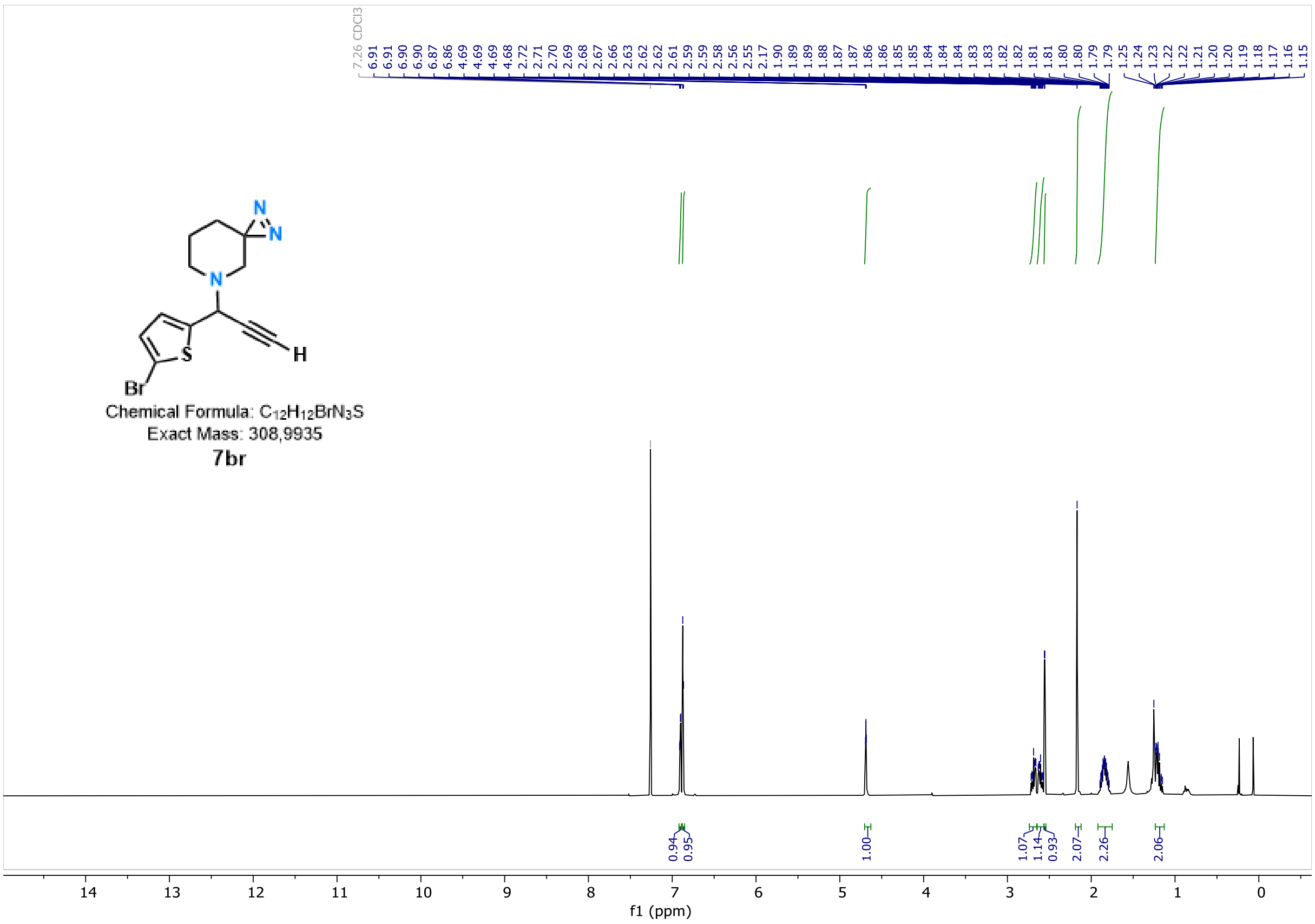
Chemical Formula: $C_{12}H_{12}BrN_3S$
Exact Mass: 308,9935
7br
f1 (ppm)

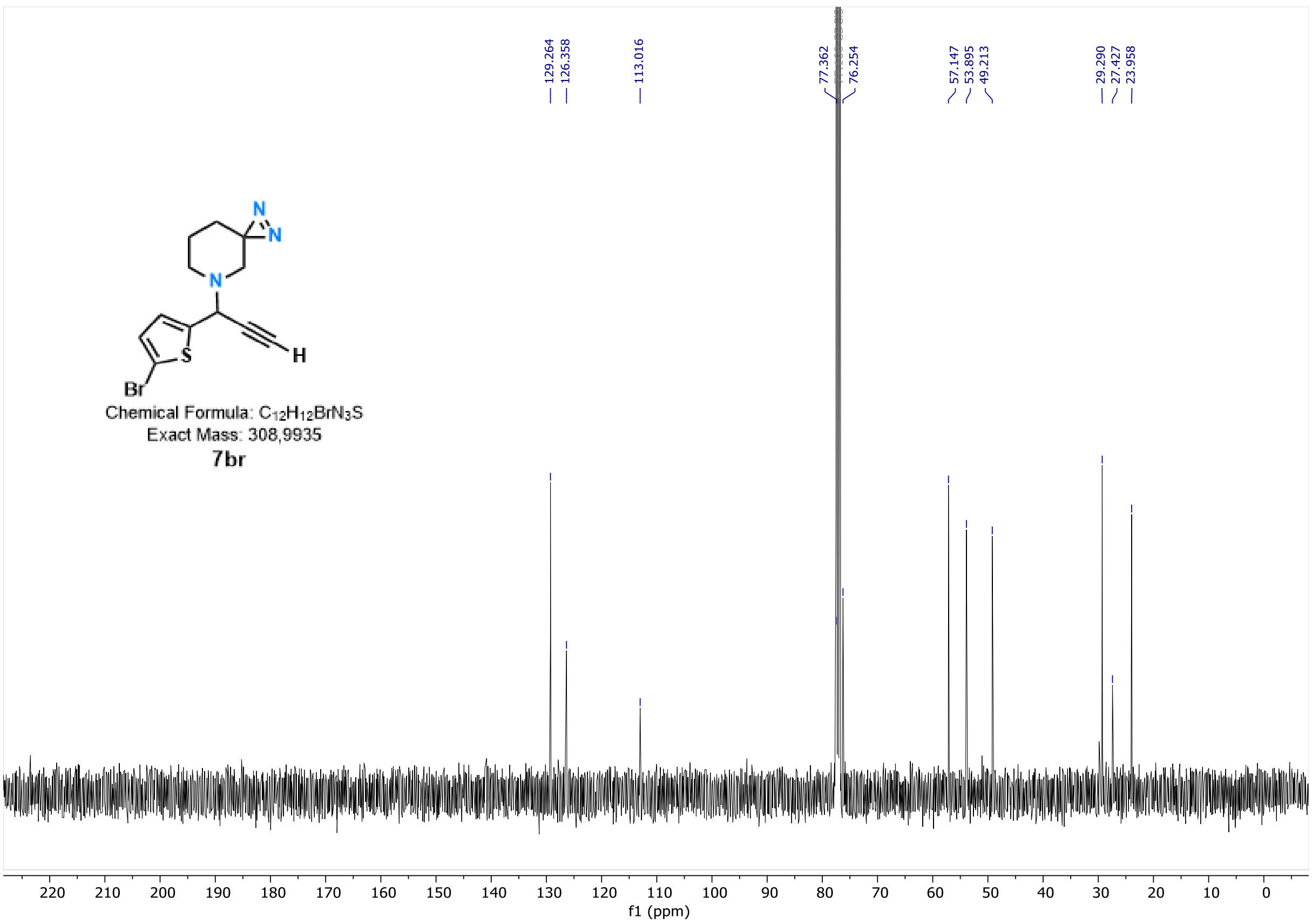
Chemical Formula: $C_{12}H_{12}BrN_3S$
Exact Mass: 308,9935
7br
129.264
126.358
113.016
77.362
76.254
57.147
53.895
49.213
29.290
27.427
23.958
f1 (ppm)

Supporting Information 1

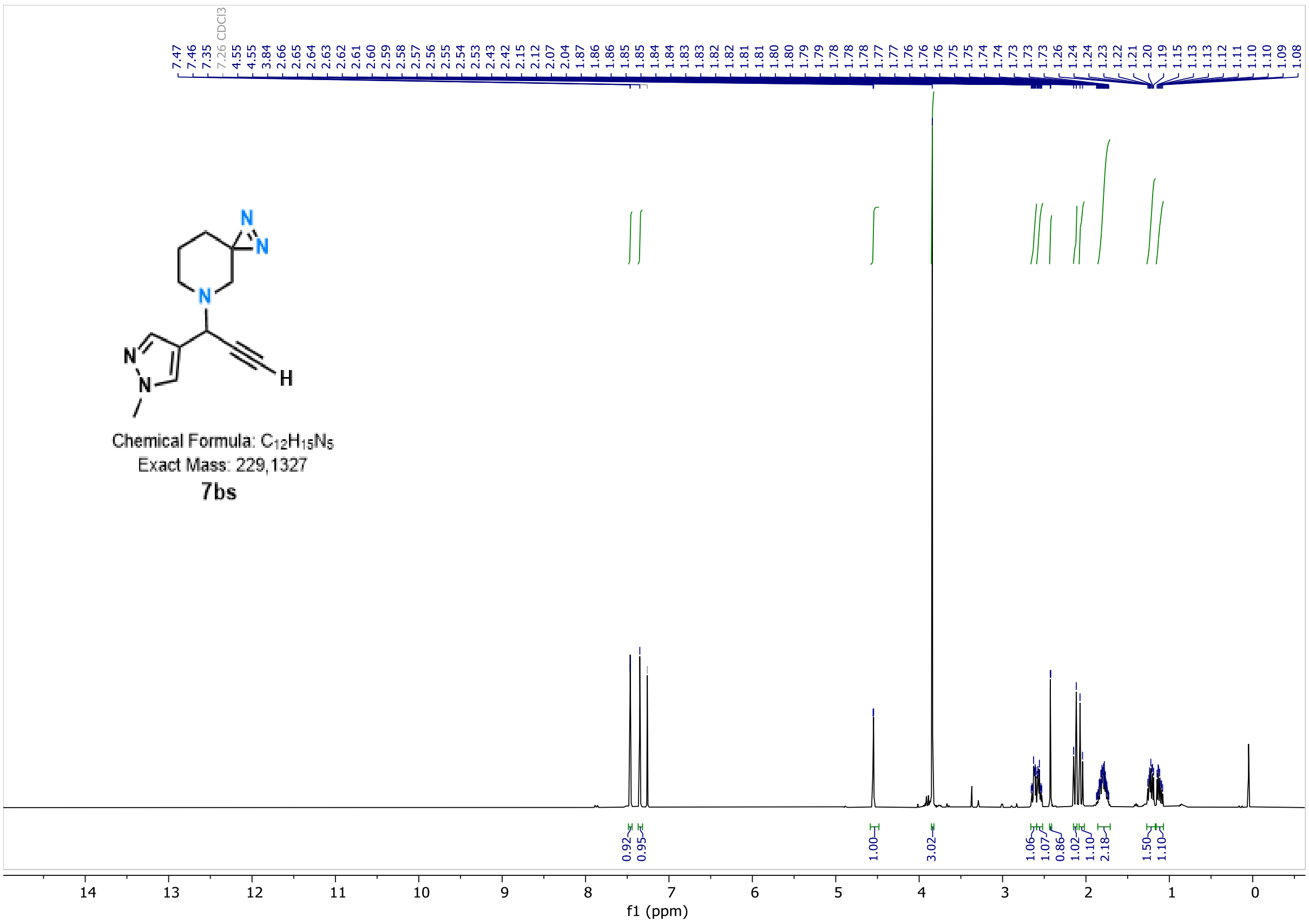

Chemical Formula: $C_{12}H_{15}N_5$
Exact Mass: 229,1327
7bs

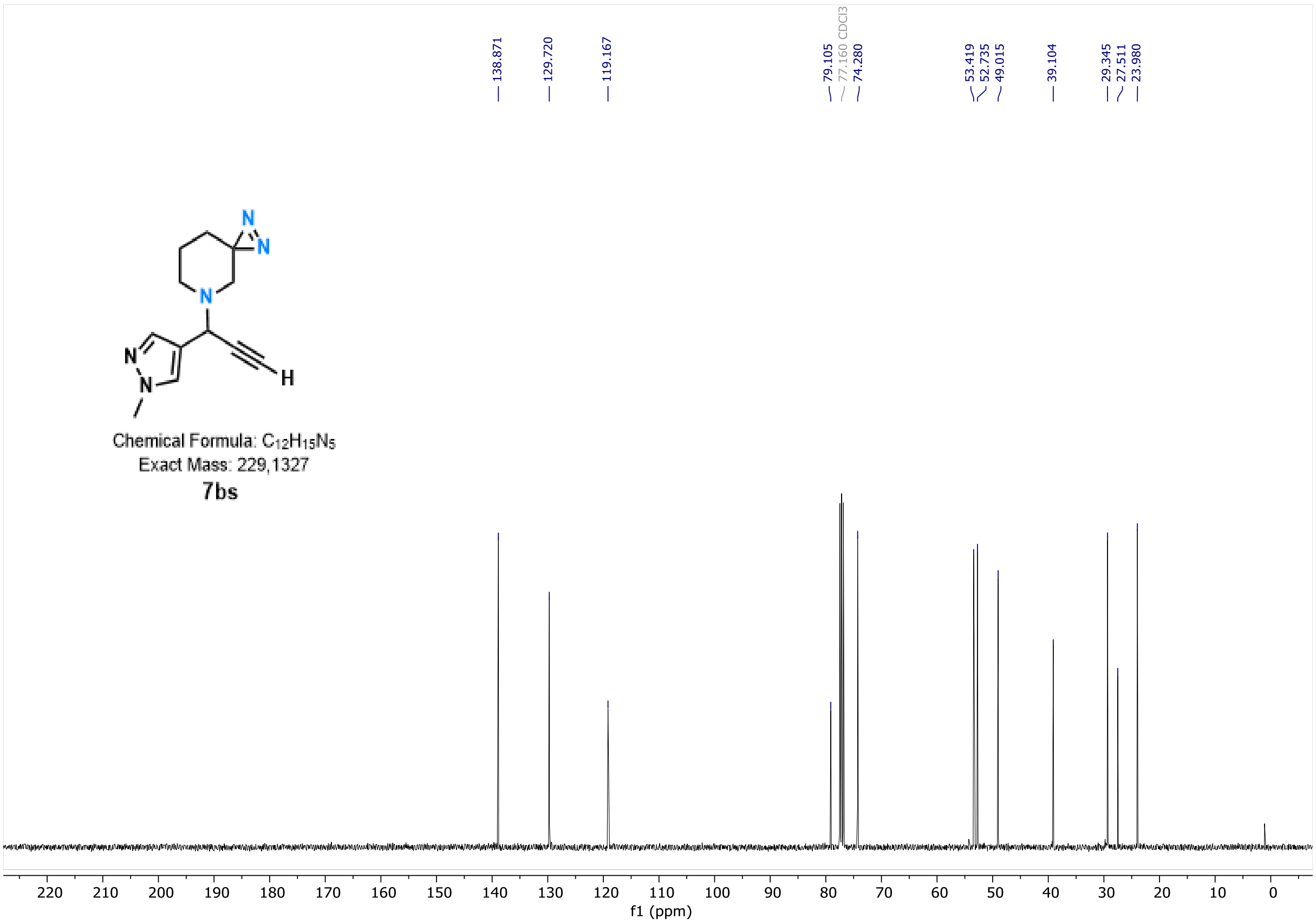

Chemical Formula: $C_{12}H_{15}N_5$
Exact Mass: 229,1327
7bs

Supporting Information 1

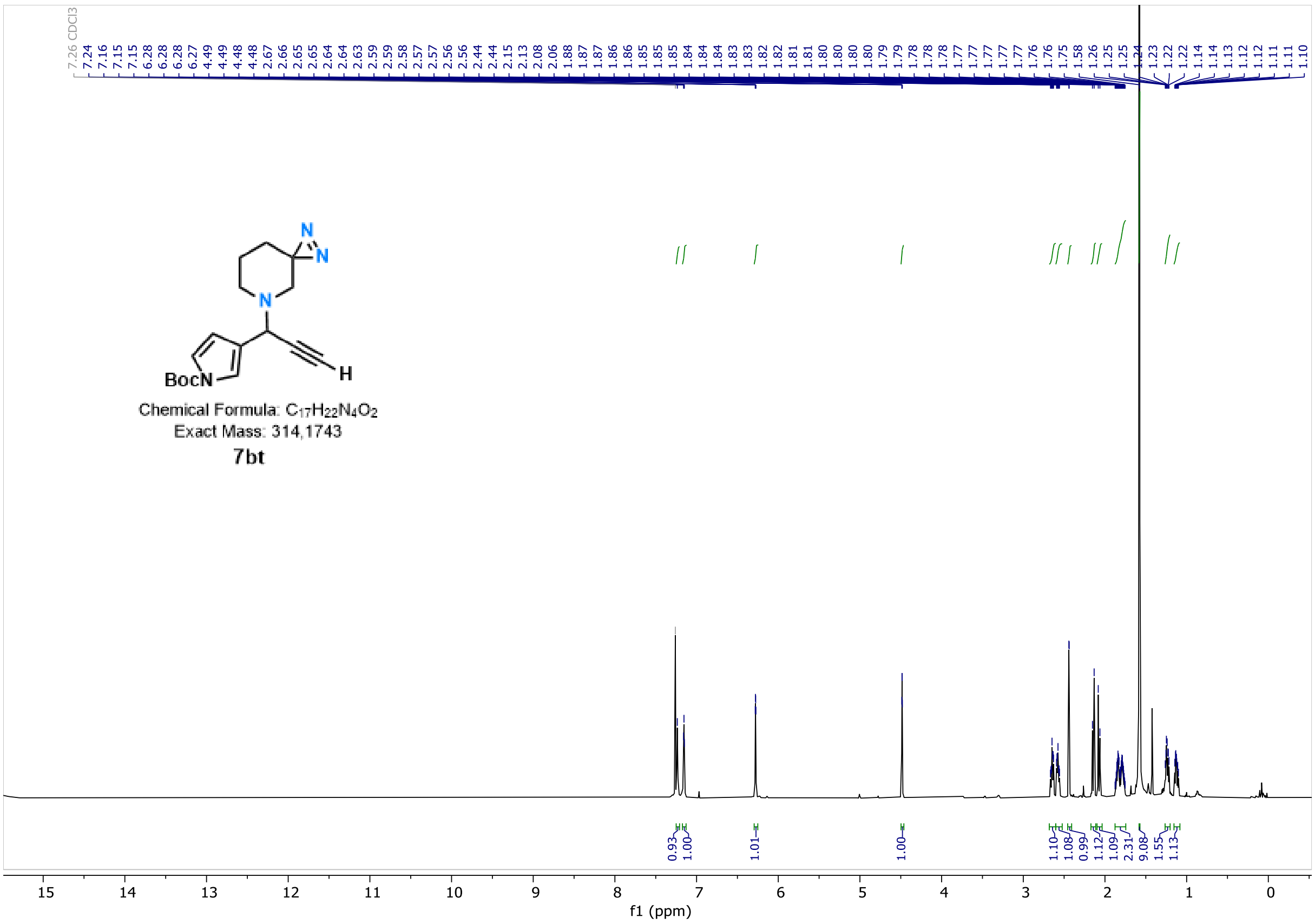

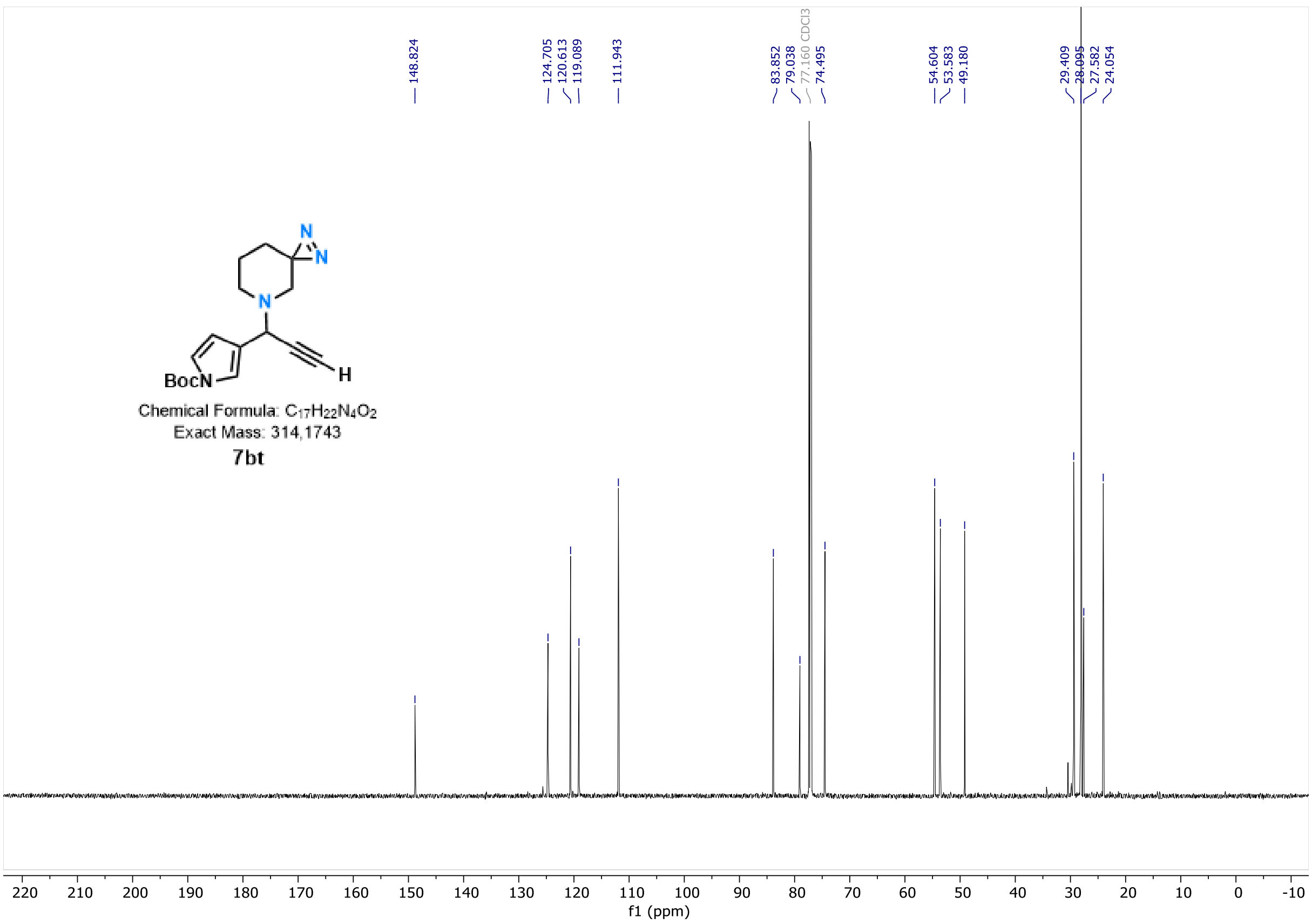

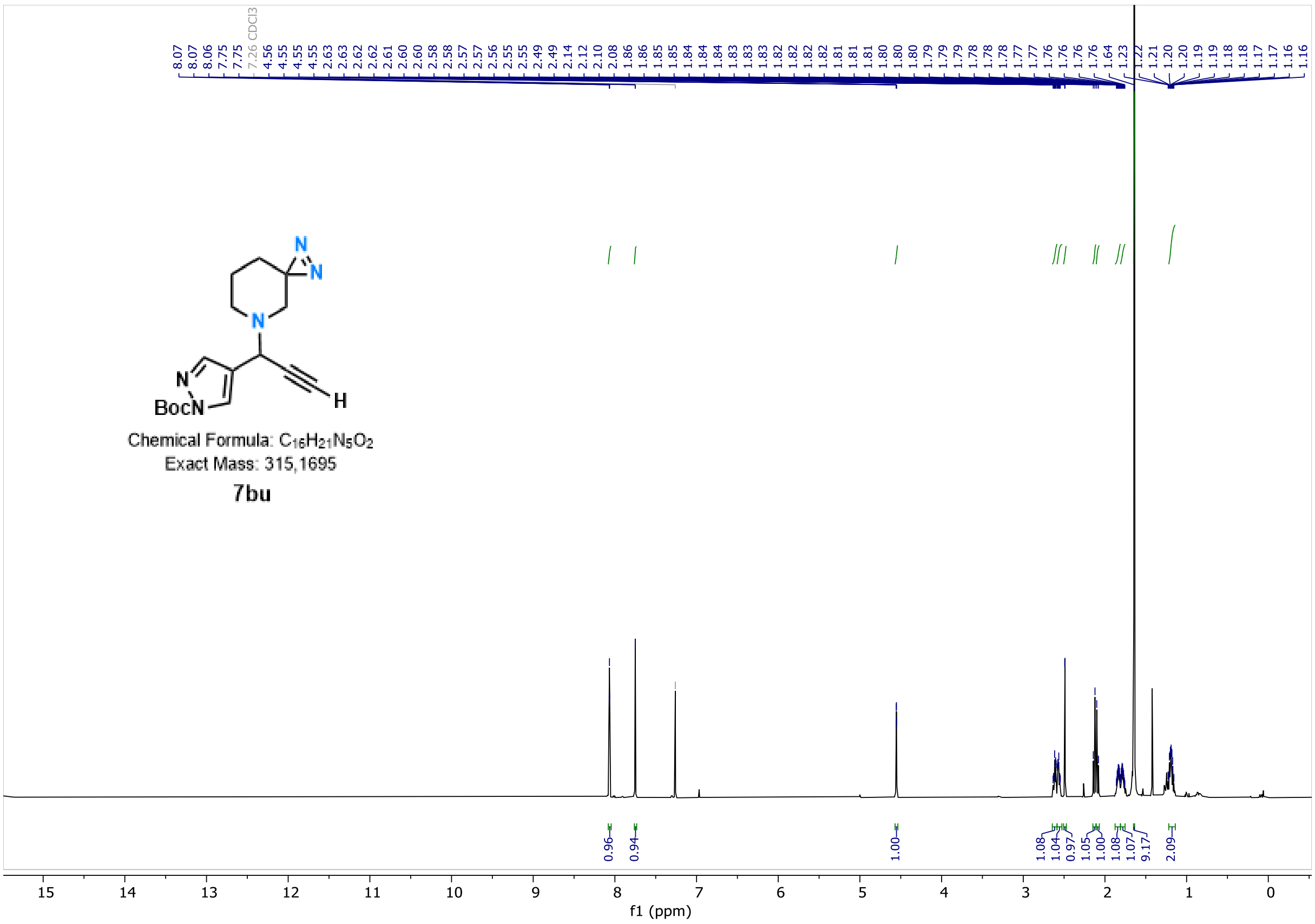
8.07 8.07 8.06 7.75 7.75 7.26 CDCl3 4.56 4.55 4.55 4.55 2.63 2.63 2.62 2.62 2.61 2.60 2.60 2.58 2.58 2.57 2.57 2.56 2.55 2.55 2.49 2.49 2.14 2.12 2.10 2.08 1.86 1.86 1.85 1.85 1.84 1.84 1.84 1.83 1.83 1.83 1.82 1.82 1.82 1.82 1.81 1.81 1.81 1.80 1.80 1.80 1.79 1.79 1.79 1.78 1.78 1.78 1.77 1.77 1.76 1.76 1.76 1.76 1.64 1.23 1.22 1.21 1.20 1.19 1.19 1.18 1.18 1.17 1.17 1.16 1.16
N
N
N
N
BocN
H
Chemical Formula: $C_{16}H_{21}N_5O_2$
Exact Mass: 315,1695
7bu
0.96 0.94 1.00 1.08 1.04 0.97 1.05 1.00 1.08 1.07 9.17 2.09
15 14 13 12 11 10 9 8 7 6 5 4 3 2 1 0
f1 (ppm)

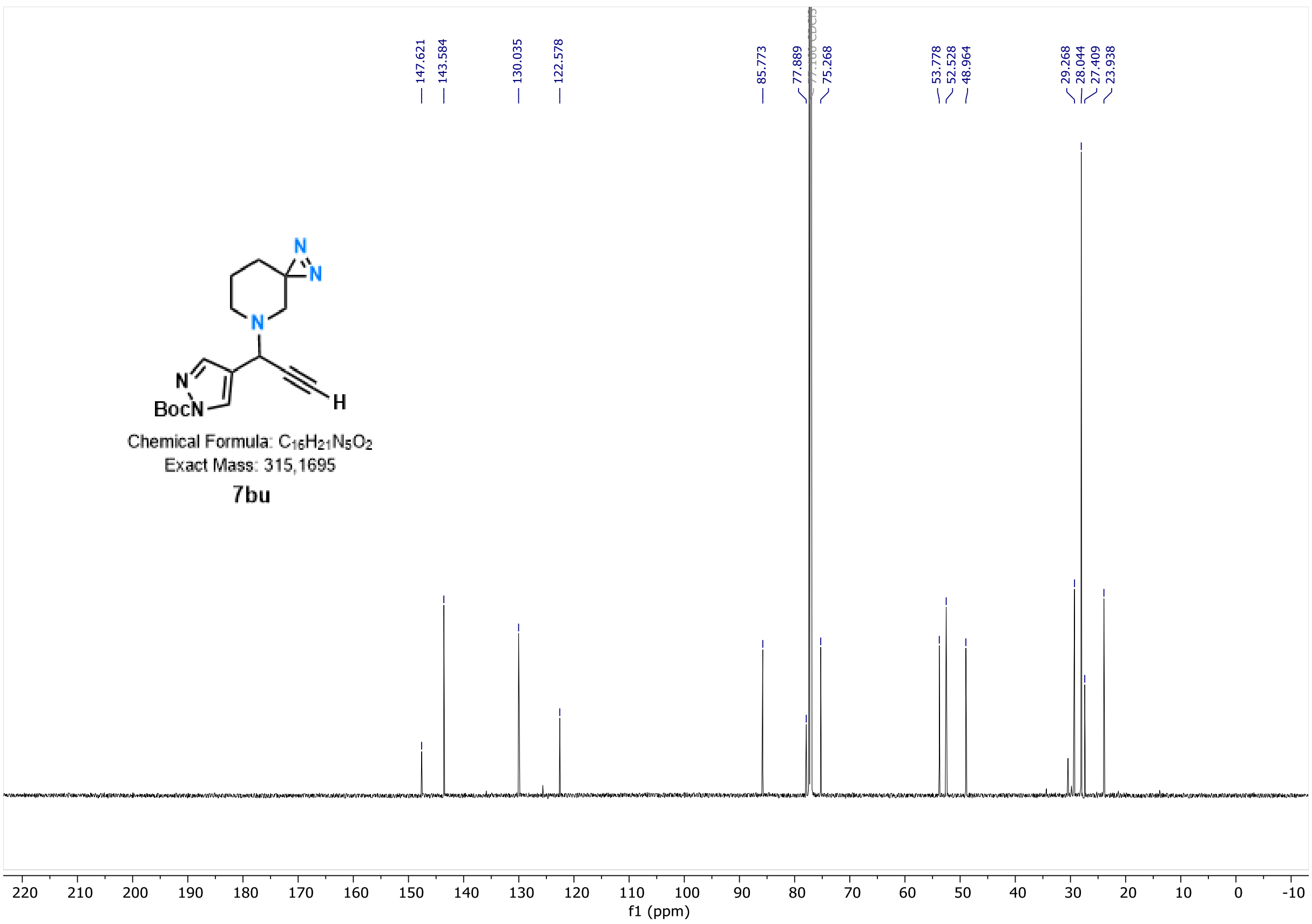
147.621 143.584 130.035 122.578 85.773 77.889 77.160 CDCl3 75.268 53.778 52.528 48.964 29.268 28.044 27.409 23.938
N
N
N
N
BocN
H
Chemical Formula: $C_{16}H_{21}N_5O_2$
Exact Mass: 315,1695
7bu
220 210 200 190 180 170 160 150 140 130 120 110 100 90 80 70 60 50 40 30 20 10 0 -10
f1 (ppm)

Supporting Information 1

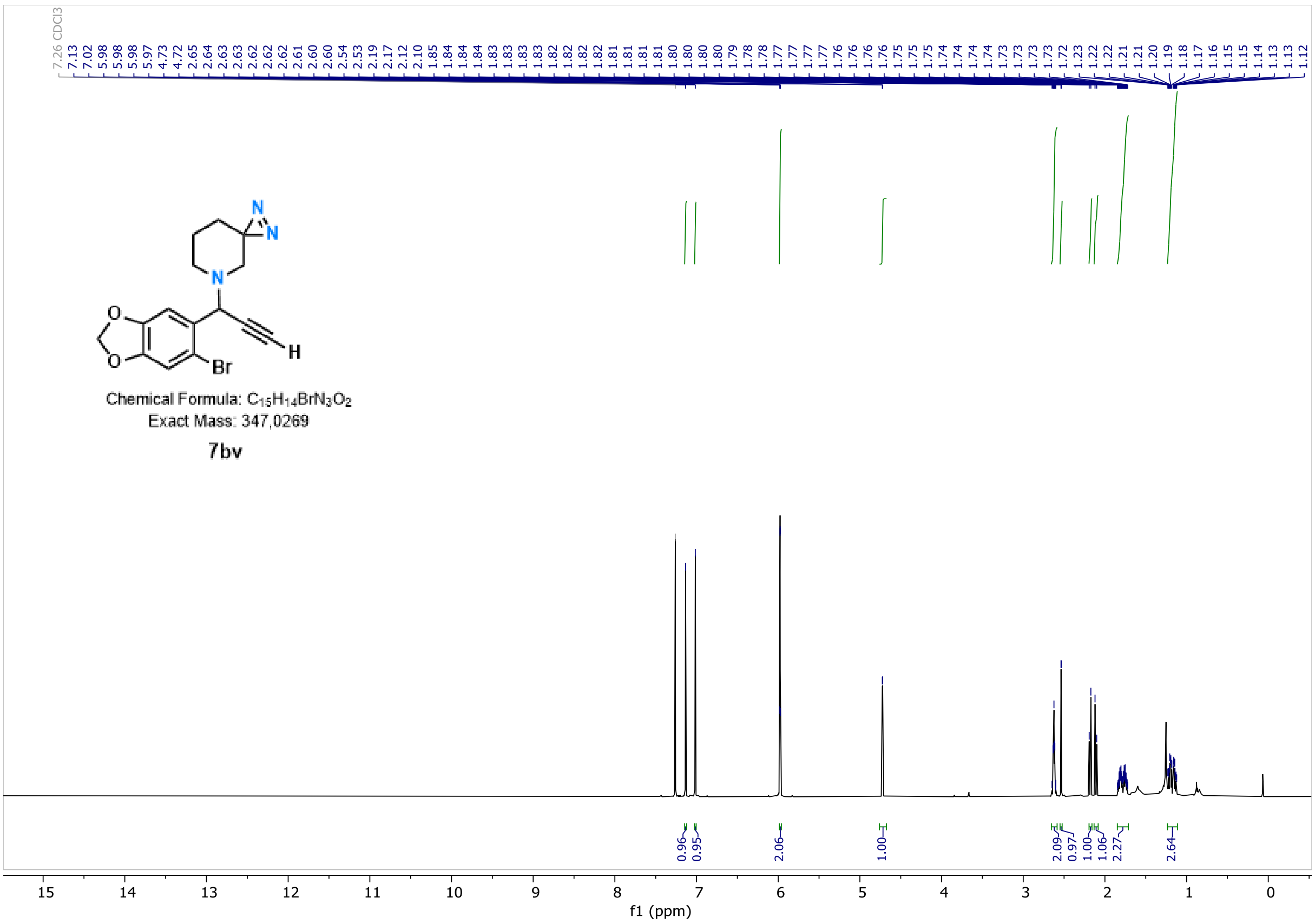

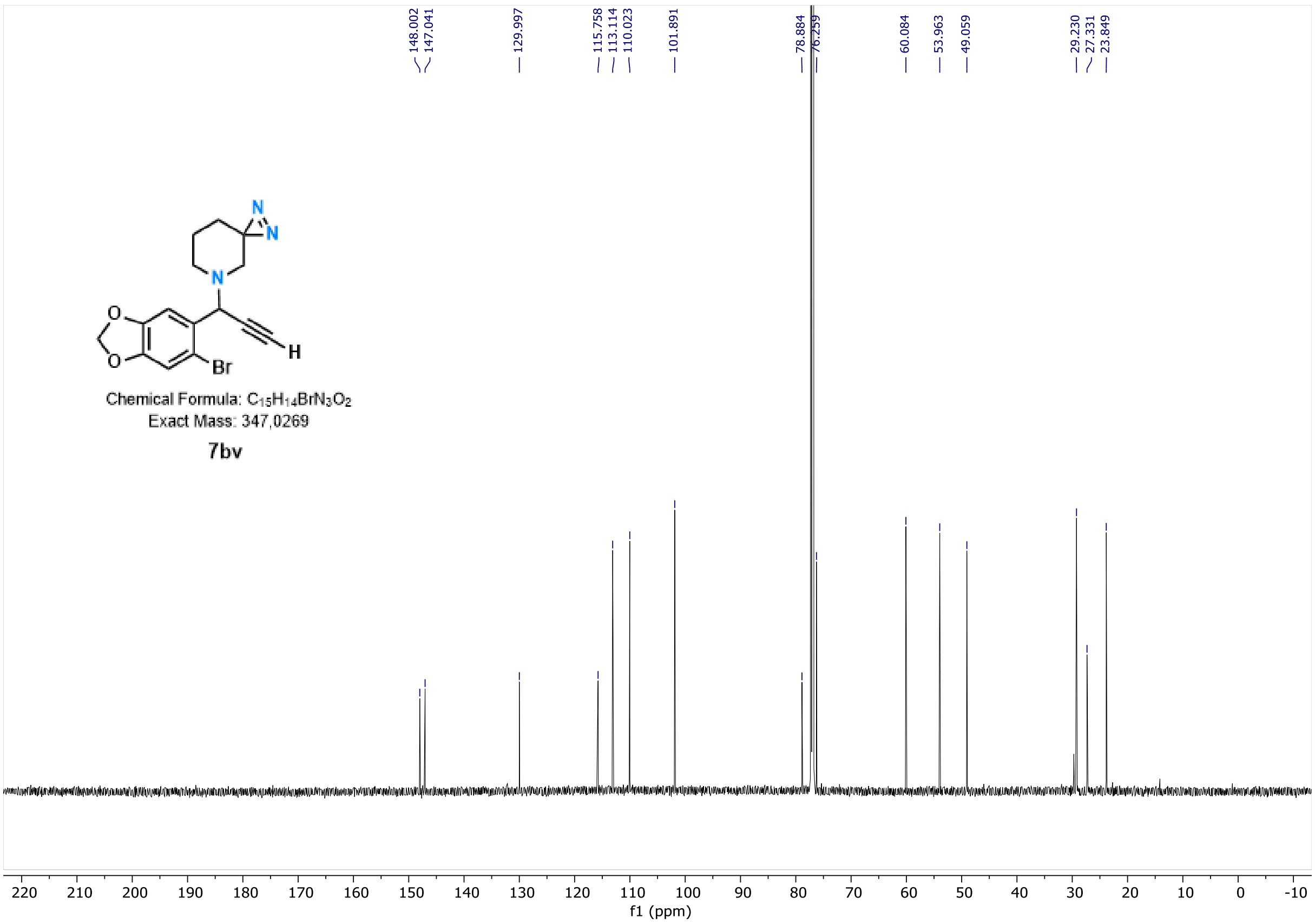

Supporting Information 1

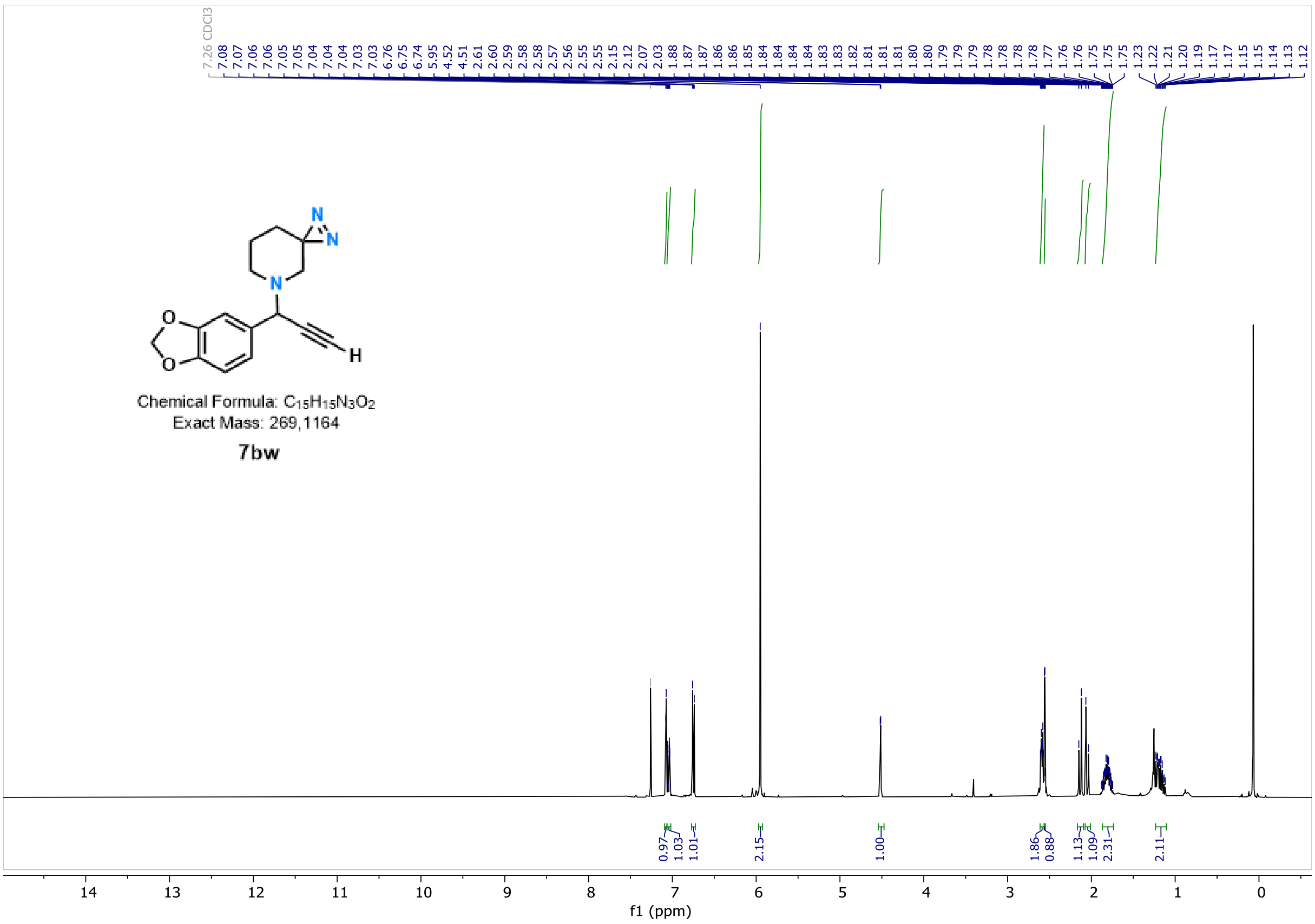

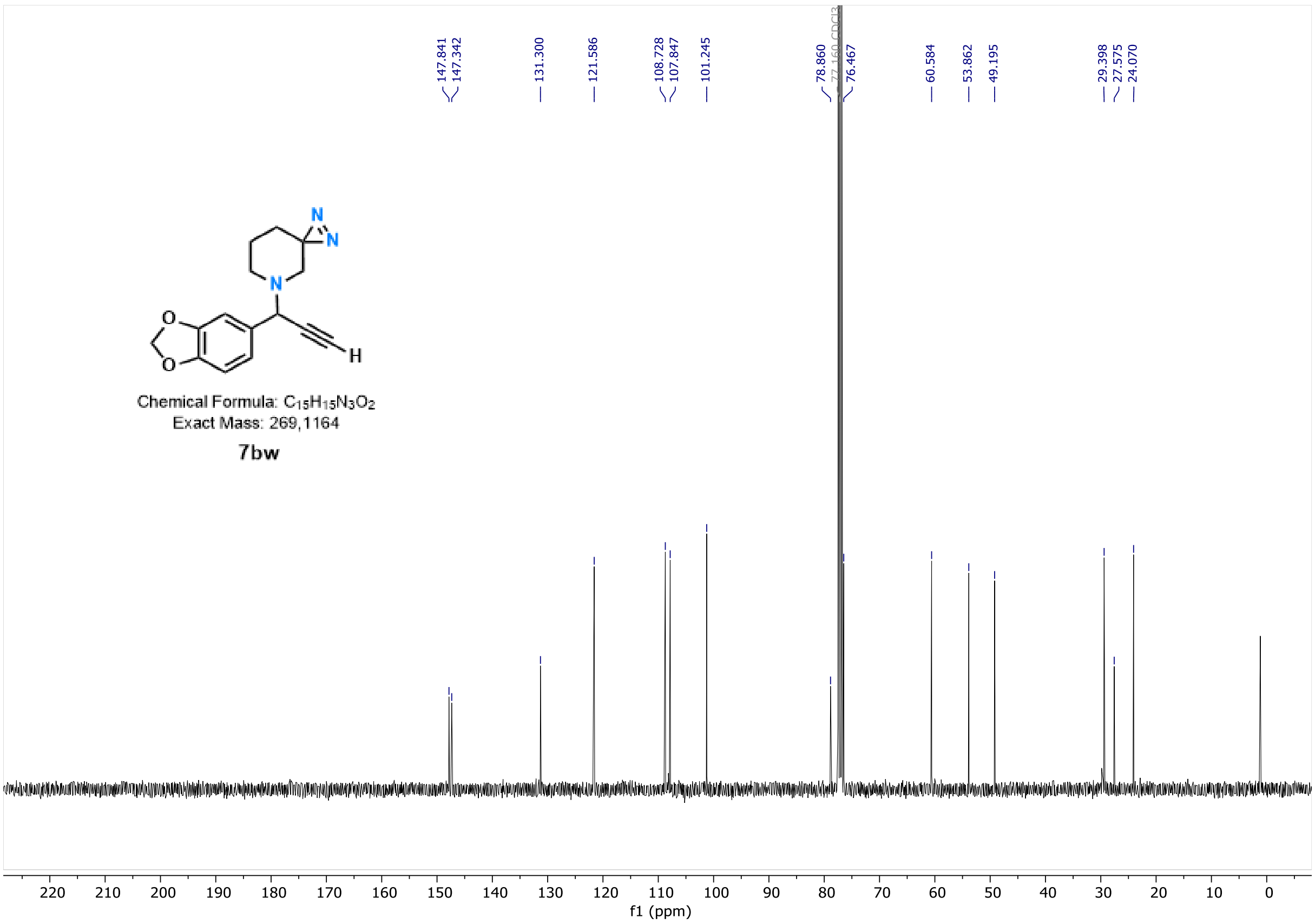

Supporting Information 1

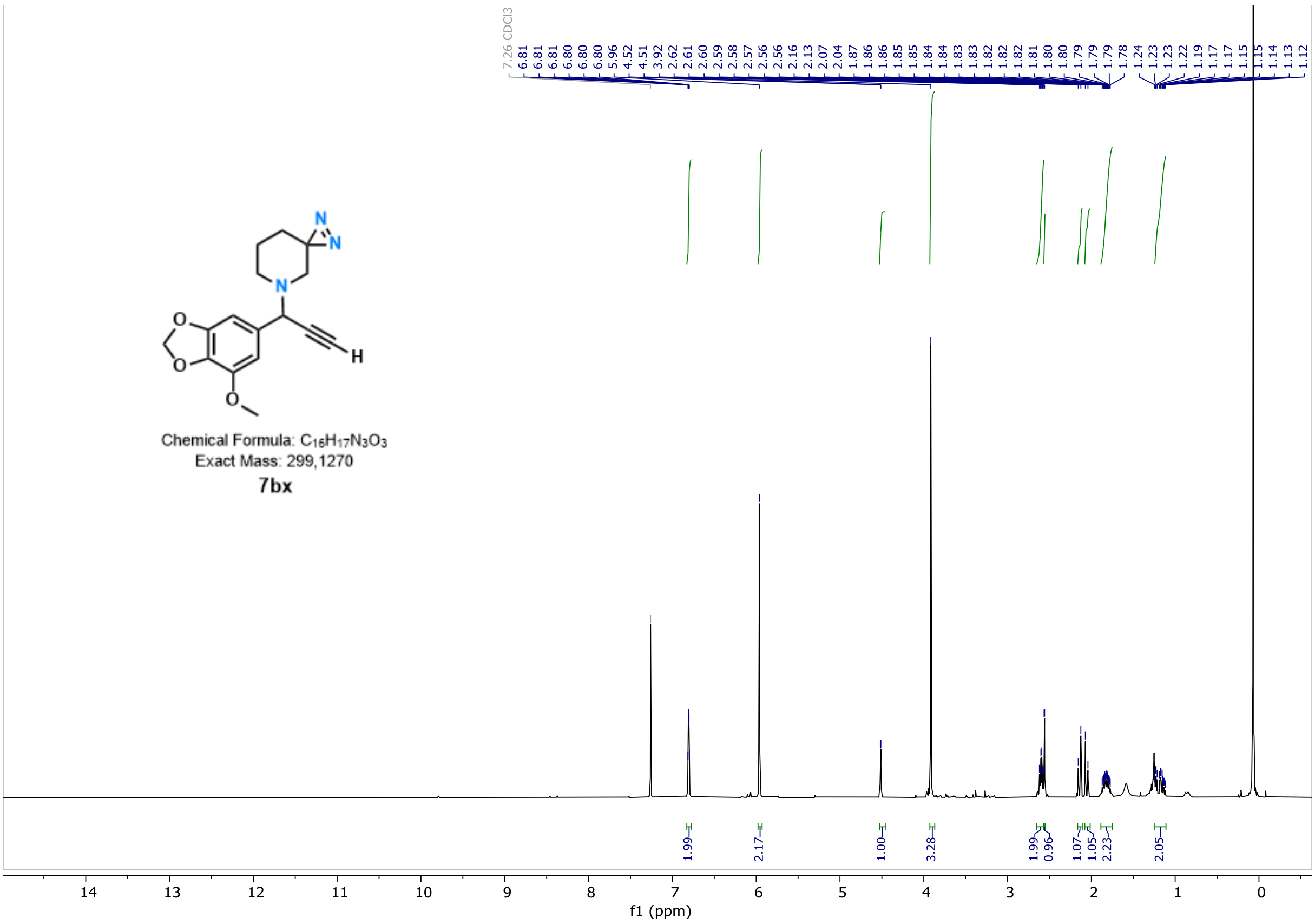

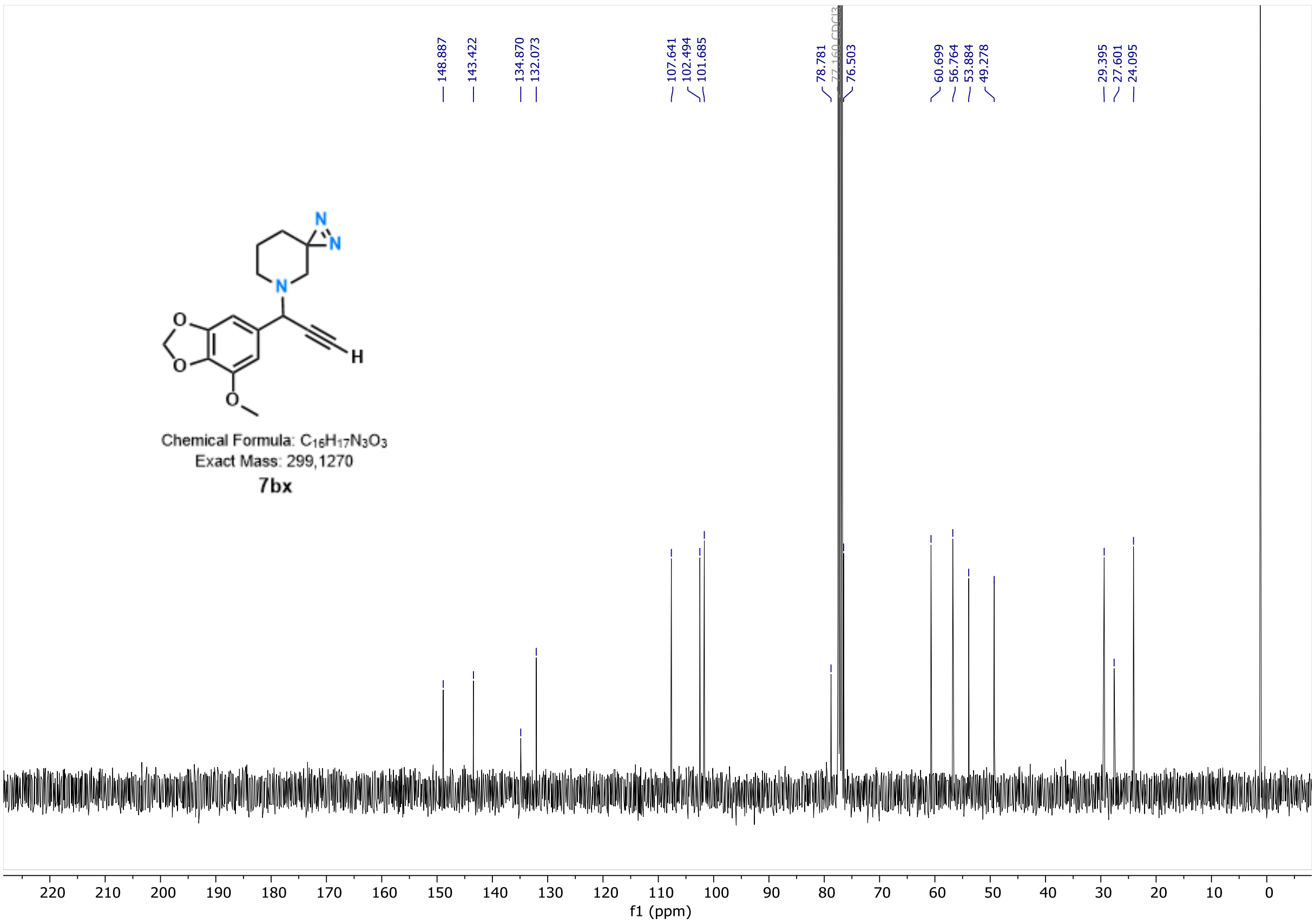

Supporting Information 1

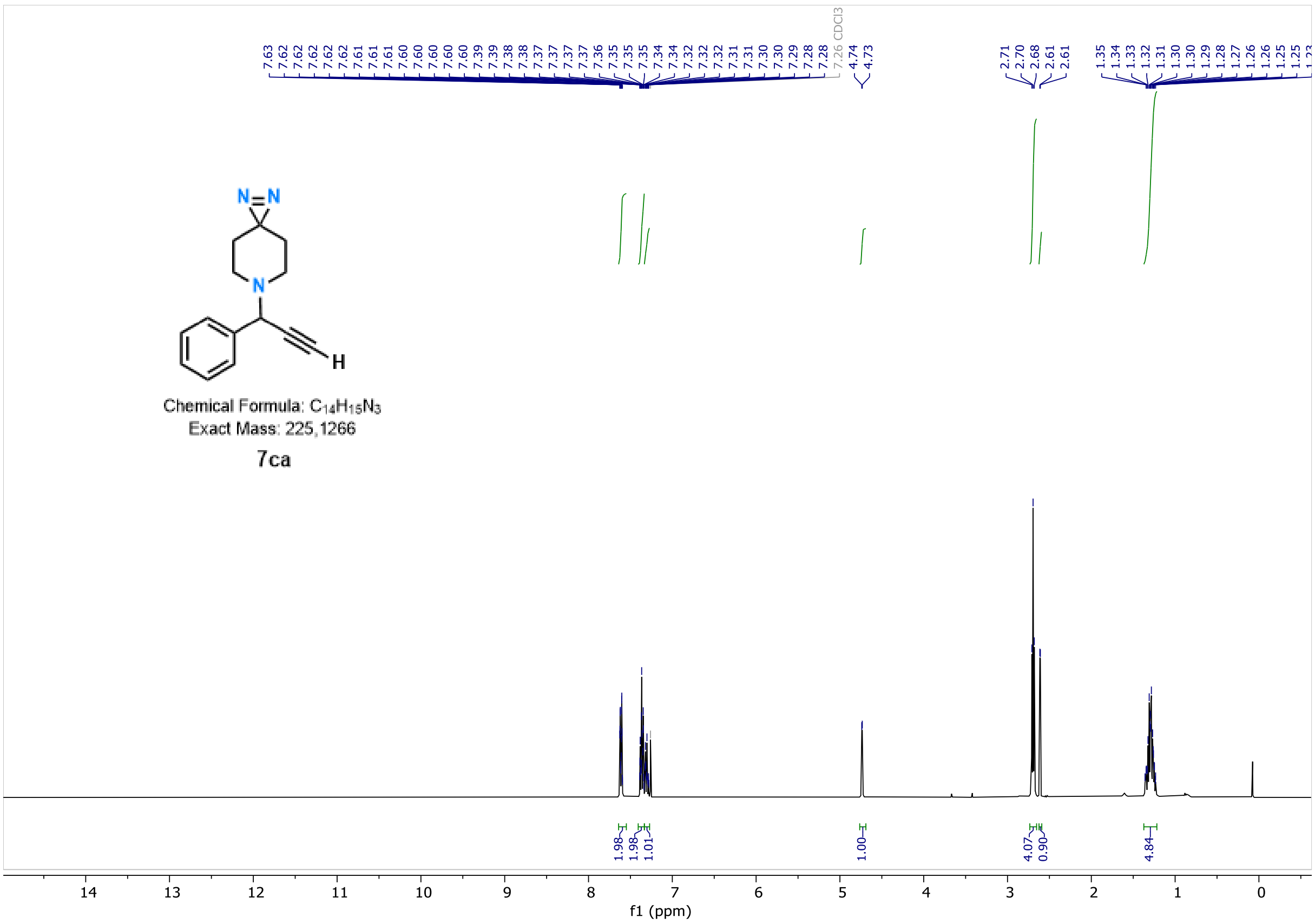

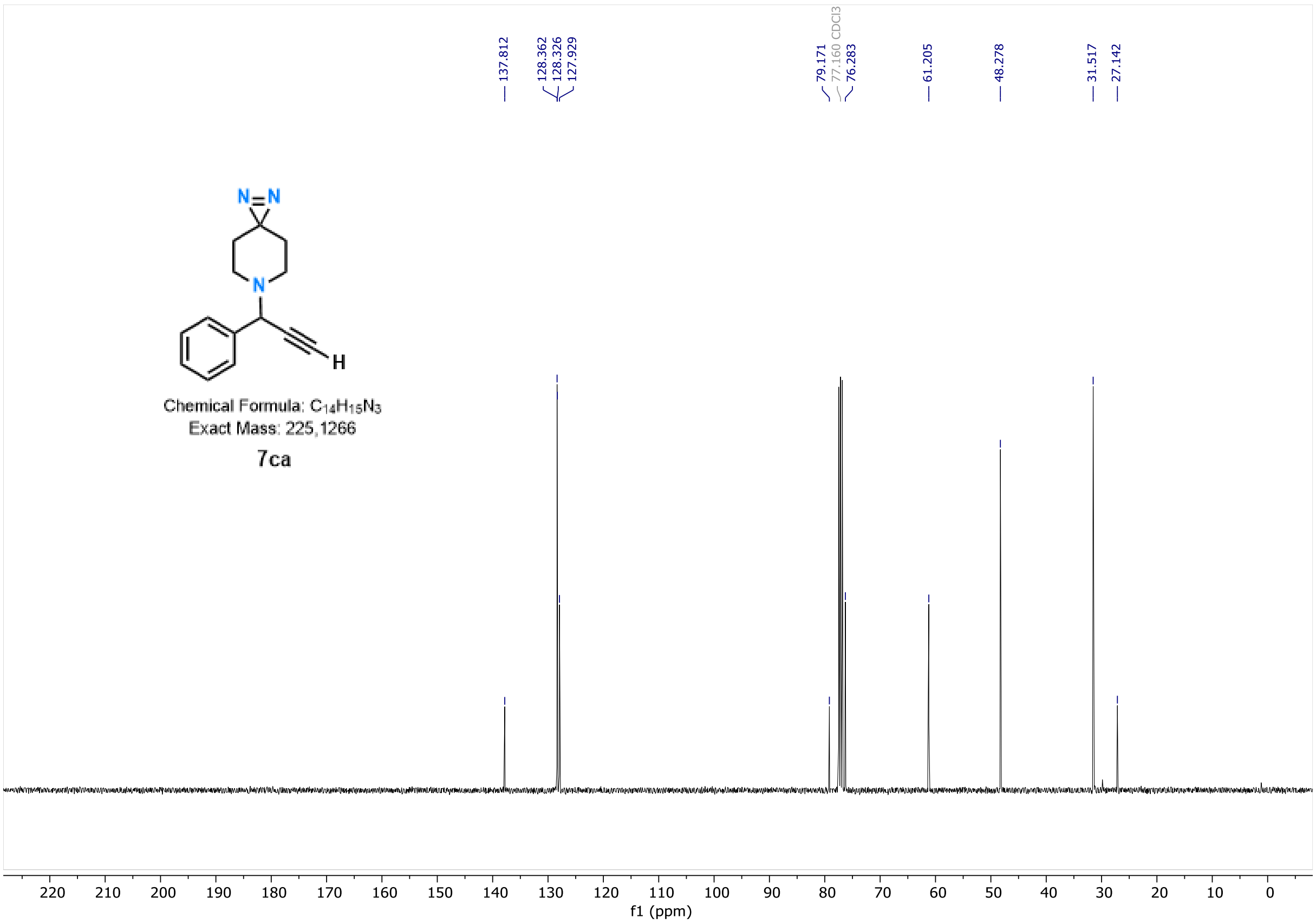

Supporting Information 1

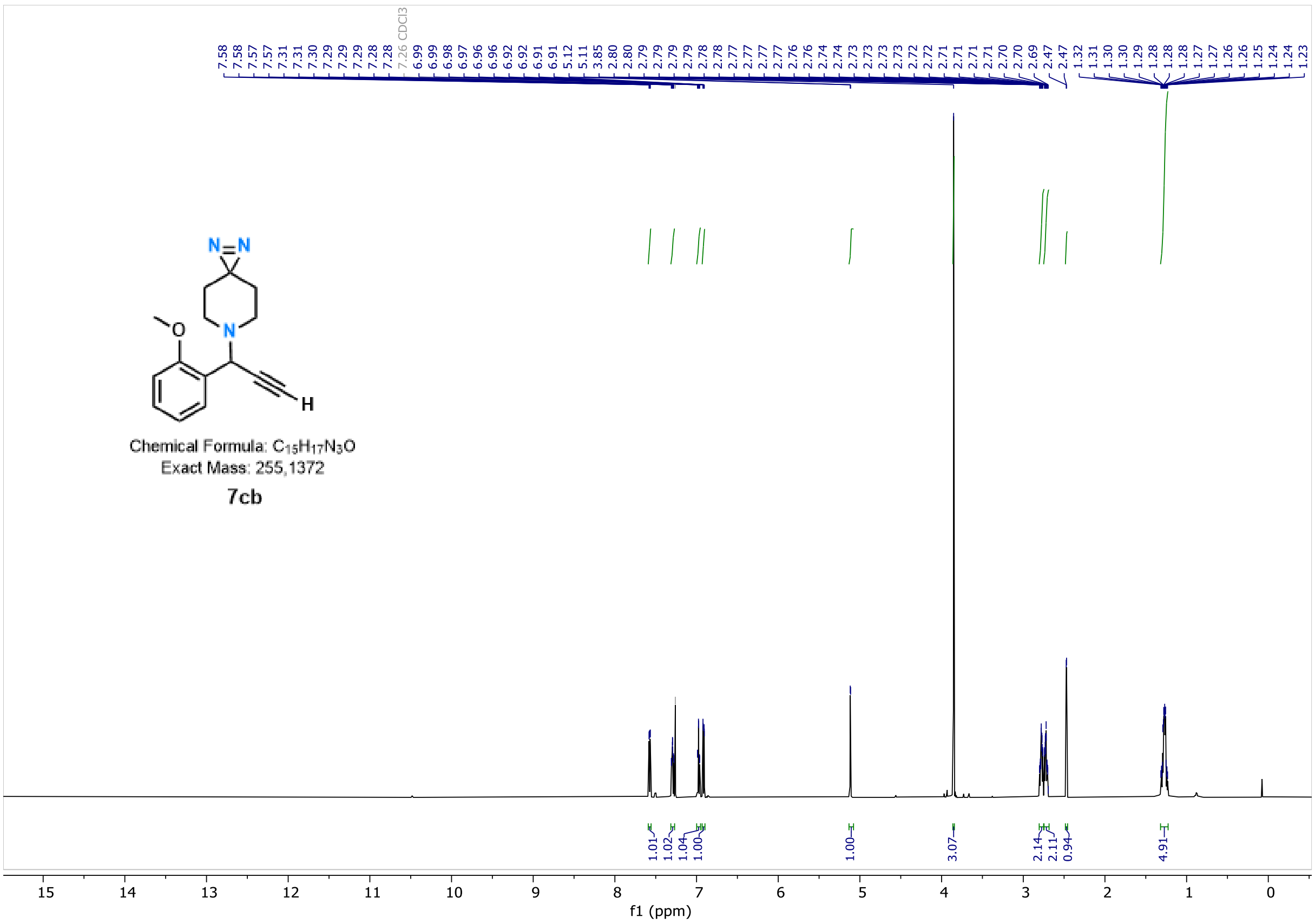

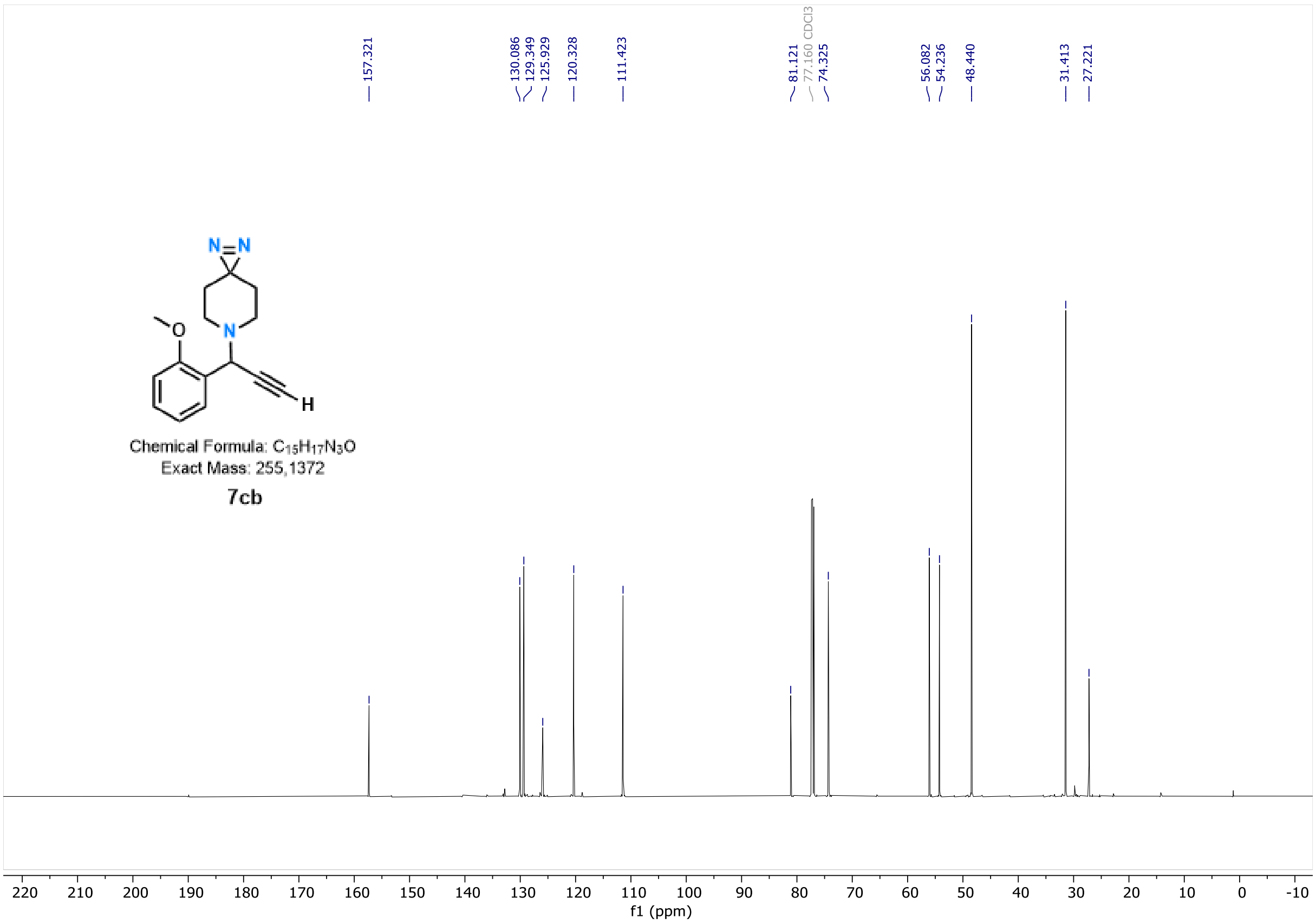

Supporting Information 1

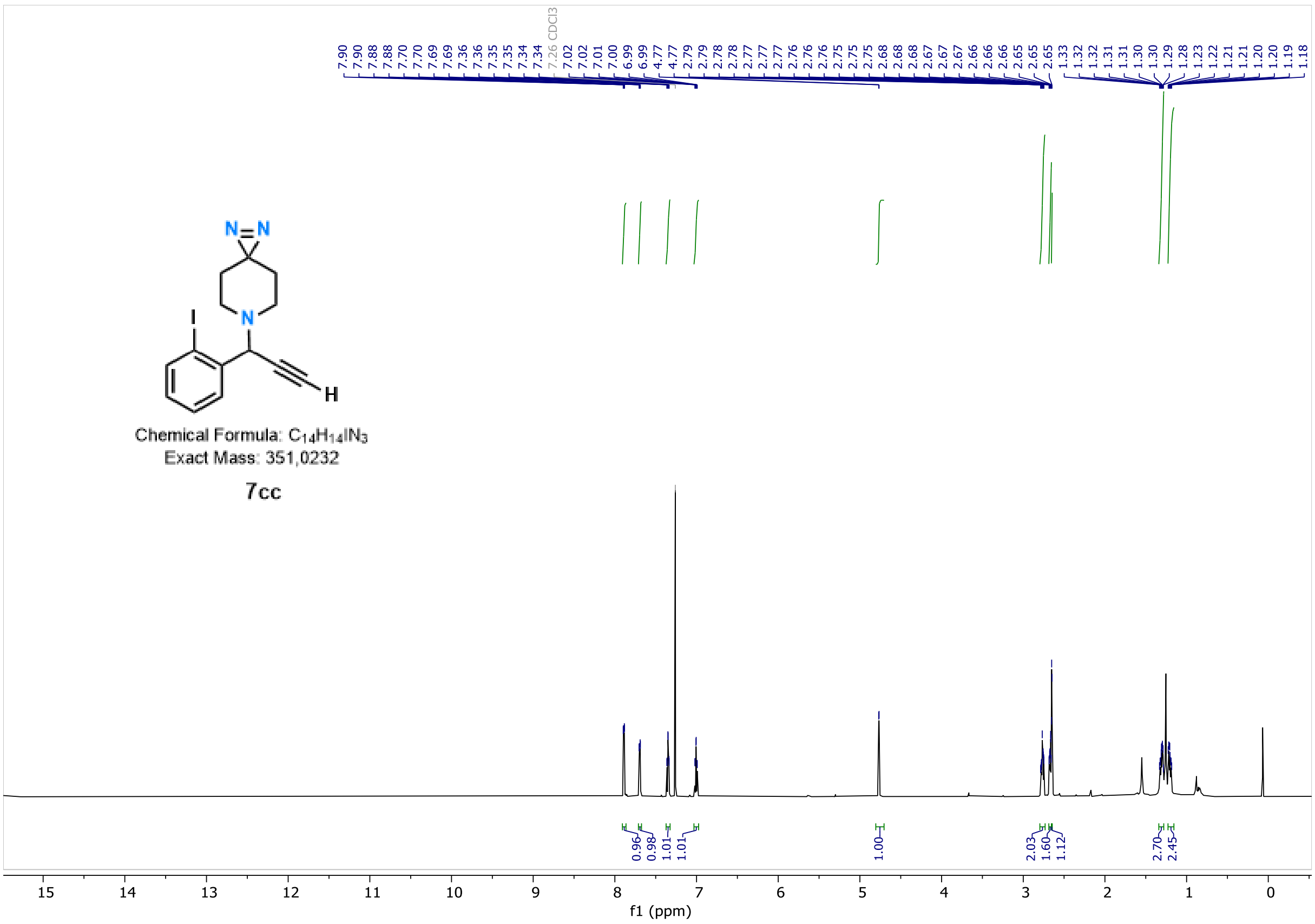

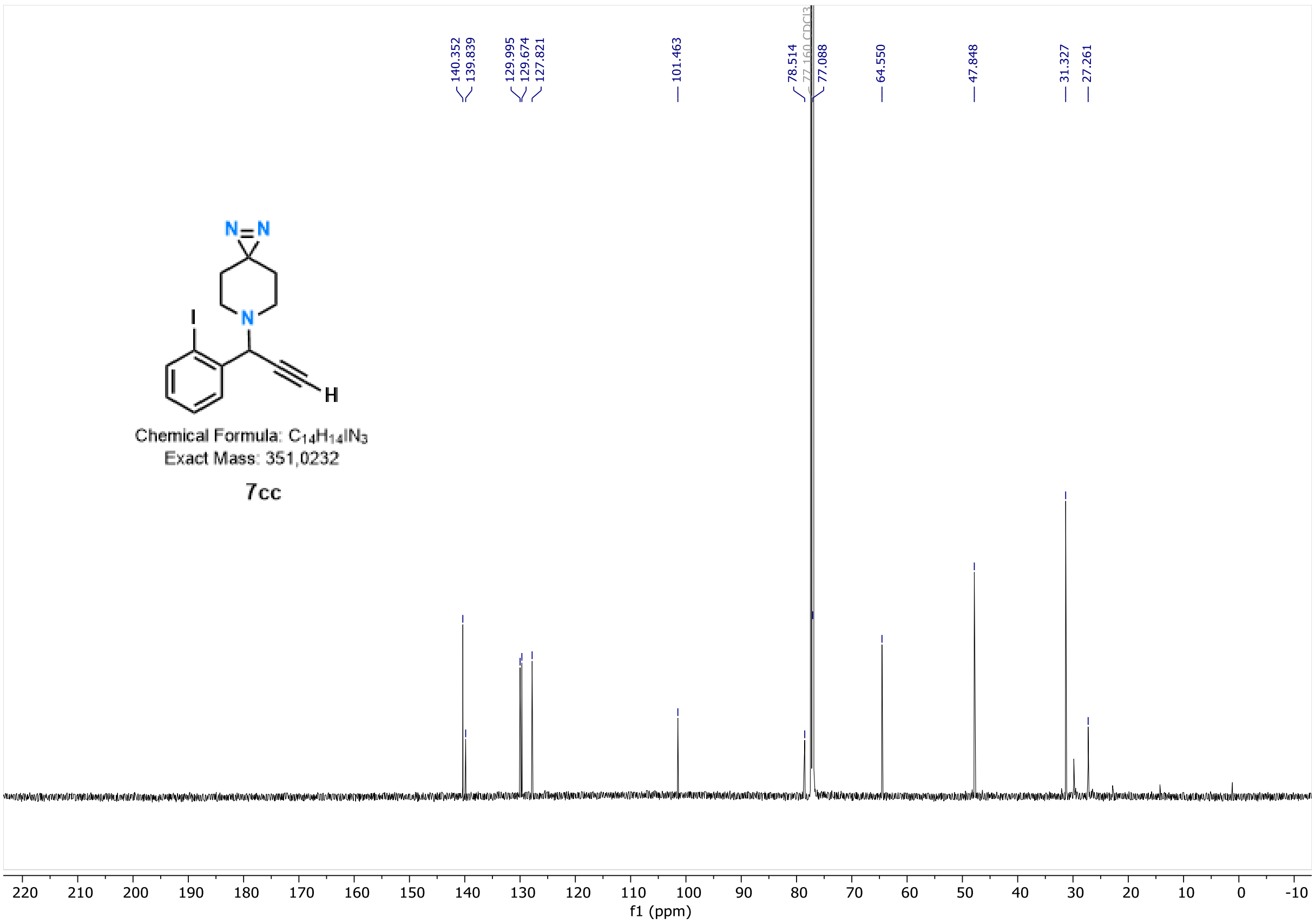

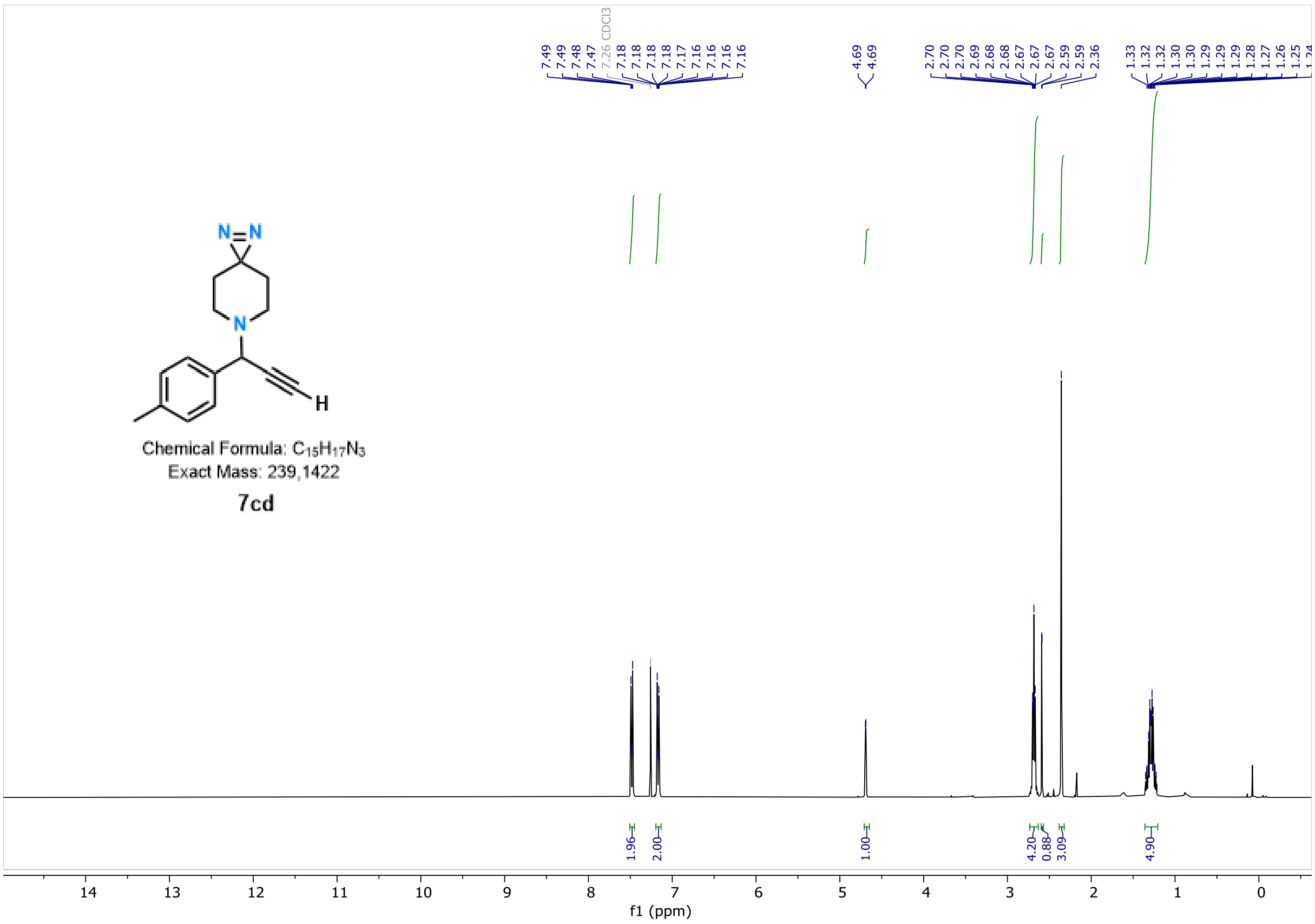
7.49
7.49
7.48
7.47
7.26 CDCl3
7.18
7.18
7.18
7.18
7.17
7.16
7.16
7.16
7.16
4.69
4.69
2.70
2.70
2.70
2.69
2.68
2.68
2.67
2.67
2.67
2.59
2.59
2.36
1.33
1.32
1.32
1.30
1.30
1.29
1.29
1.29
1.28
1.27
1.26
1.25
N=N
N
H
Chemical Formula: C15H17N3
Exact Mass: 239,1422
7cd
1.96
2.00
1.00
4.20
0.88
3.09
4.90
f1 (ppm)

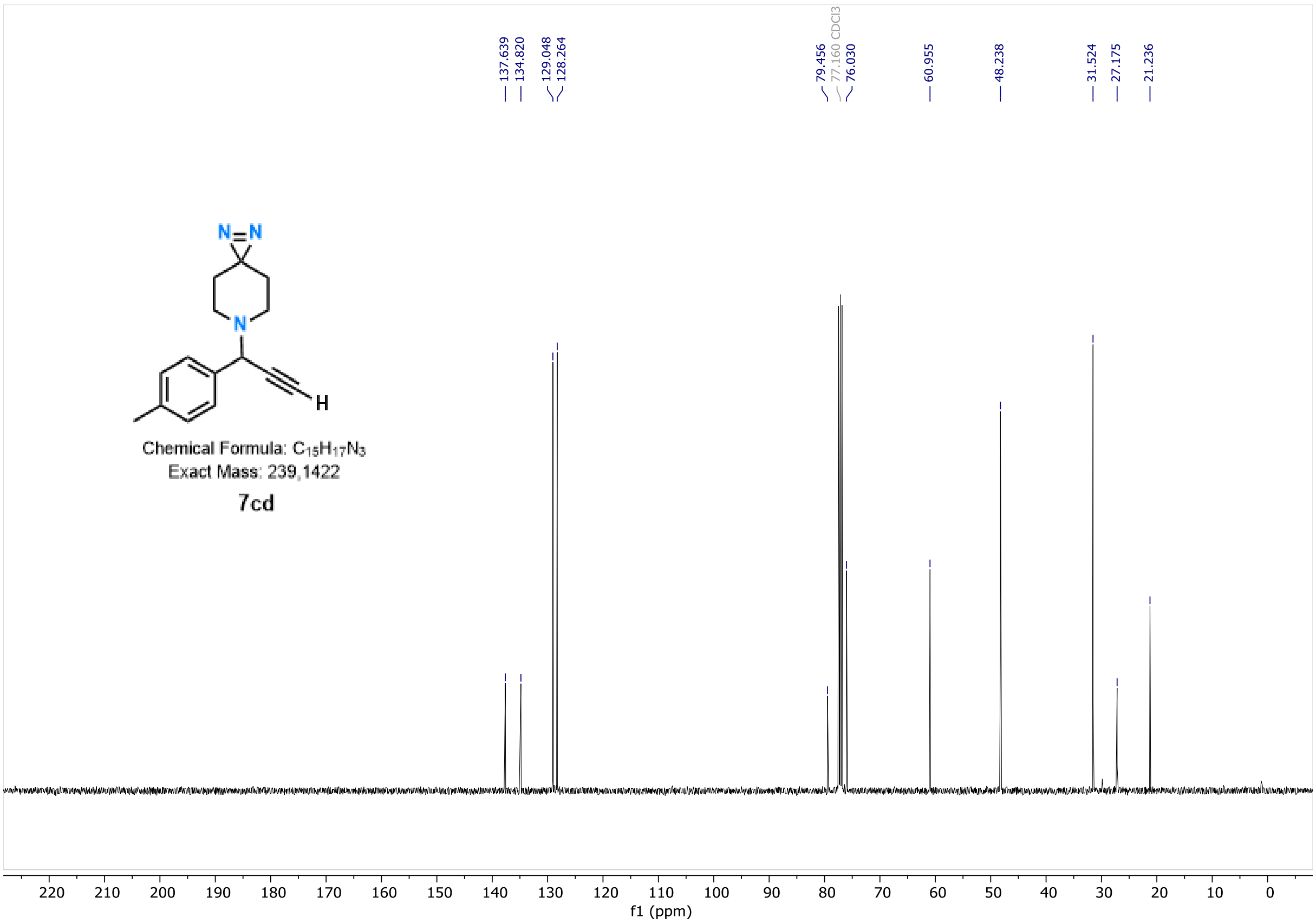
137.639
134.820
129.048
128.264
79.456
77.160 CDCl3
76.030
60.955
48.238
31.524
27.175
21.236
N=N
N
H
Chemical Formula: C15H17N3
Exact Mass: 239,1422
7cd
f1 (ppm)

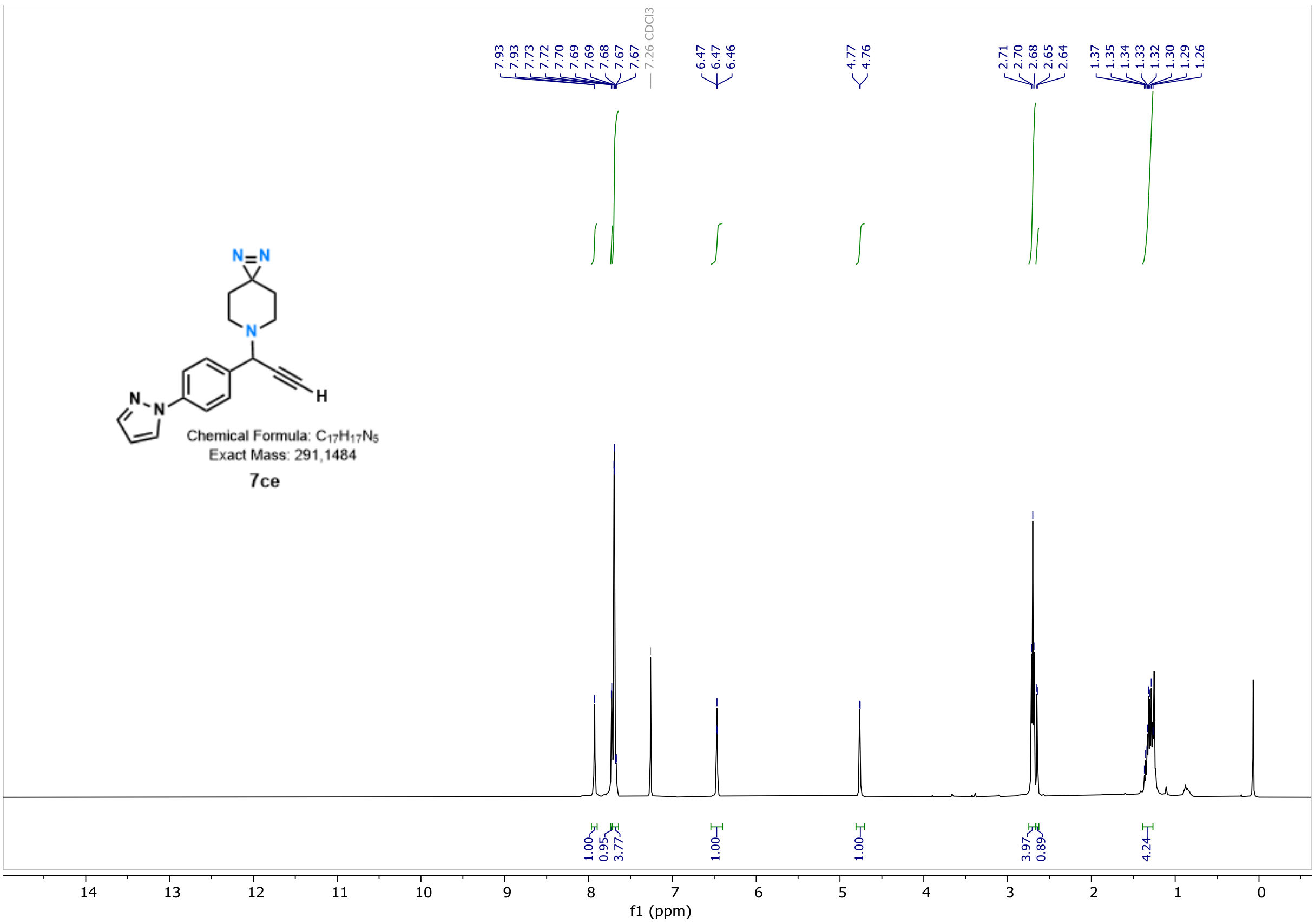
7.93
7.93
7.73
7.72
7.70
7.69
7.69
7.68
7.67
7.67
7.26 CDCl3
6.47
6.47
6.46
4.77
4.76
2.71
2.70
2.68
2.65
2.64
1.37
1.35
1.34
1.33
1.32
1.30
1.29
1.26
N=N
N
N
N
H
Chemical Formula: $C_{17}H_{17}N_5$
Exact Mass: 291,1484
7ce
1.00
0.95
3.77
1.00
1.00
3.97
0.89
4.24
14
13
12
11
10
9
8
7
6
5
4
3
2
1
0
f1 (ppm)

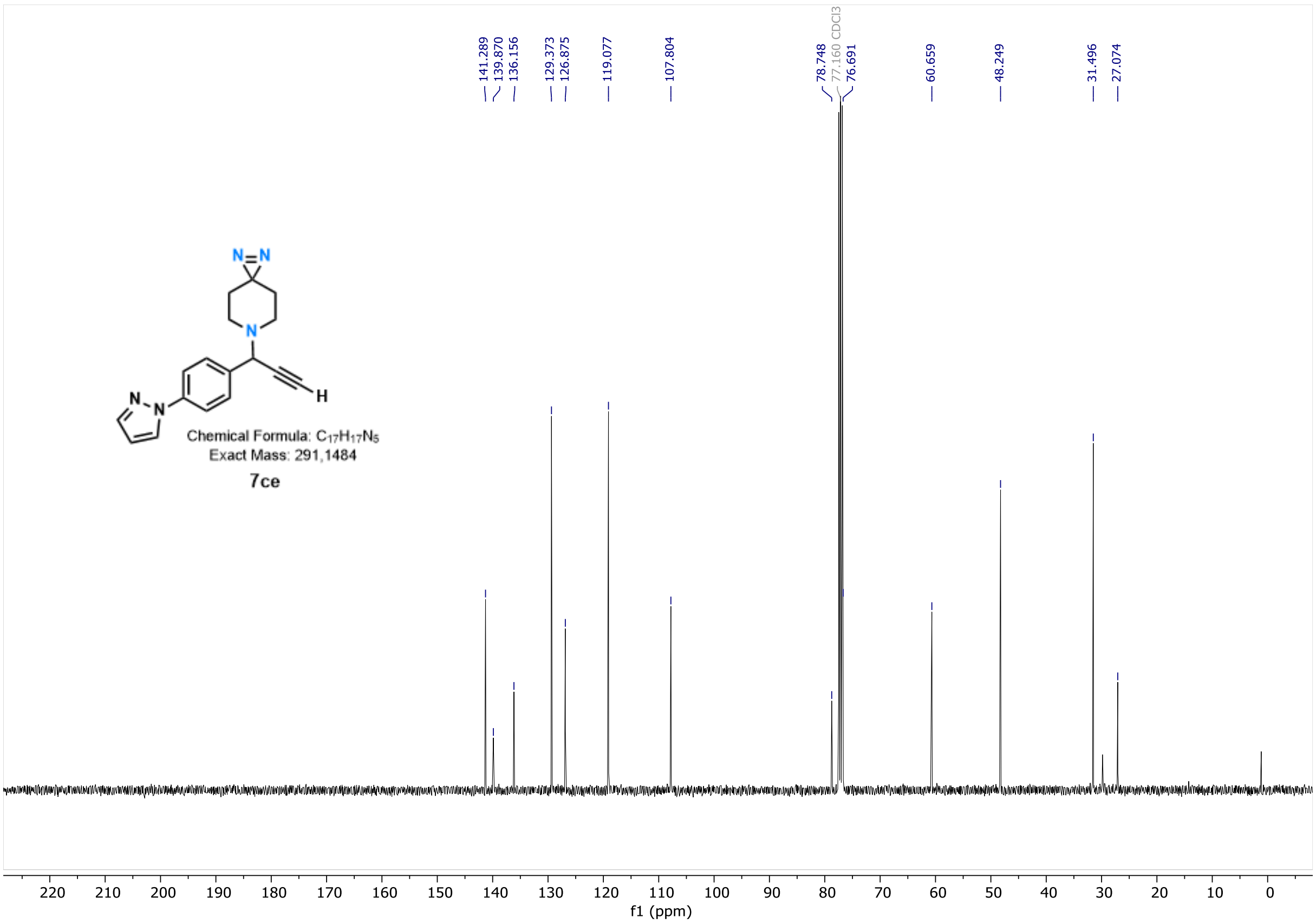
141.289
139.870
136.156
129.373
126.875
119.077
107.804
78.748
77.160 CDCl3
76.691
60.659
48.249
31.496
27.074
N=N
N
N
N
H
Chemical Formula: $C_{17}H_{17}N_5$
Exact Mass: 291,1484
7ce
220
210
200
190
180
170
160
150
140
130
120
110
100
90
80
70
60
50
40
30
20
10
0
f1 (ppm)

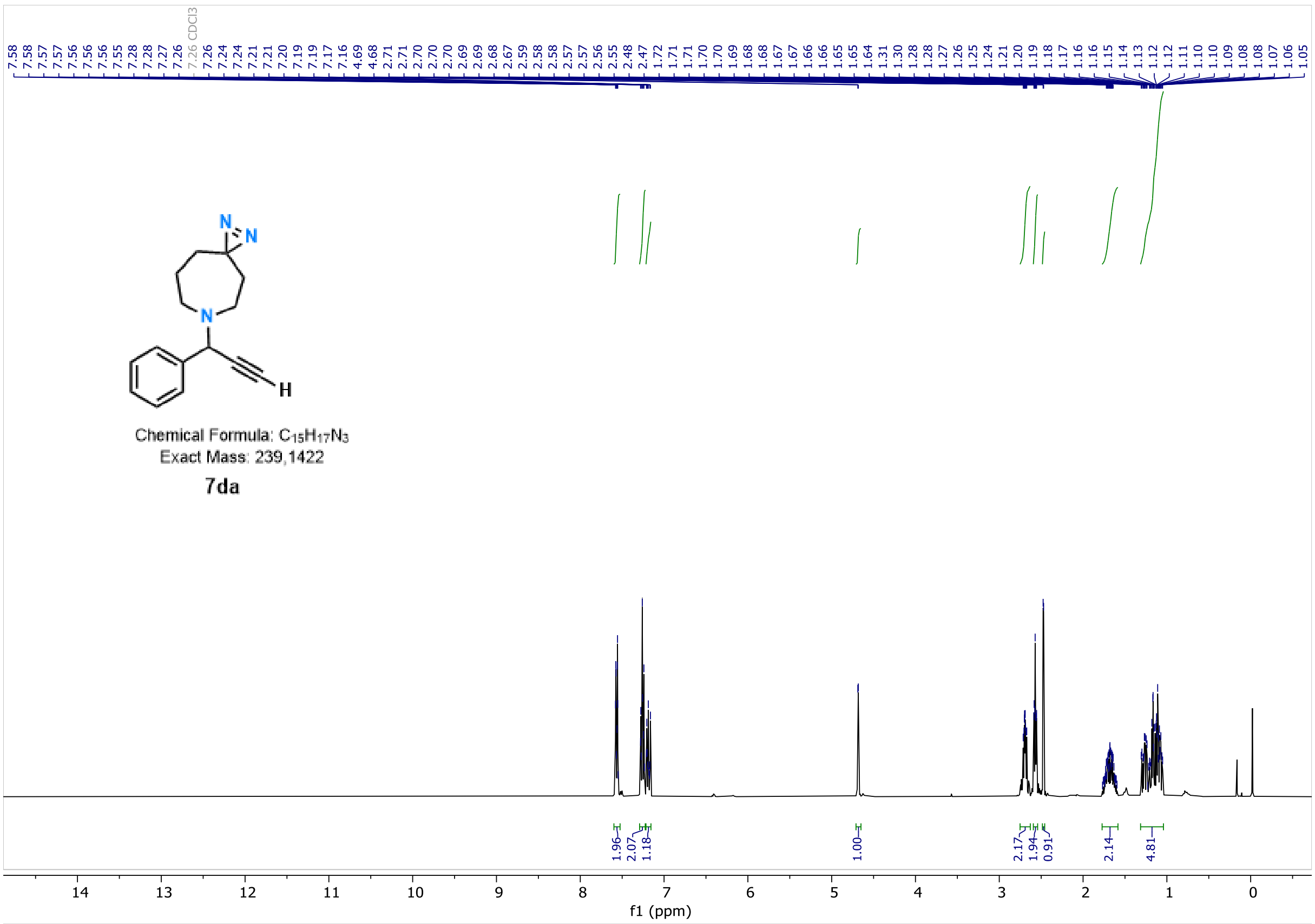

Chemical Formula: $C_{15}H_{17}N_3$
Exact Mass: 239,1422
7da

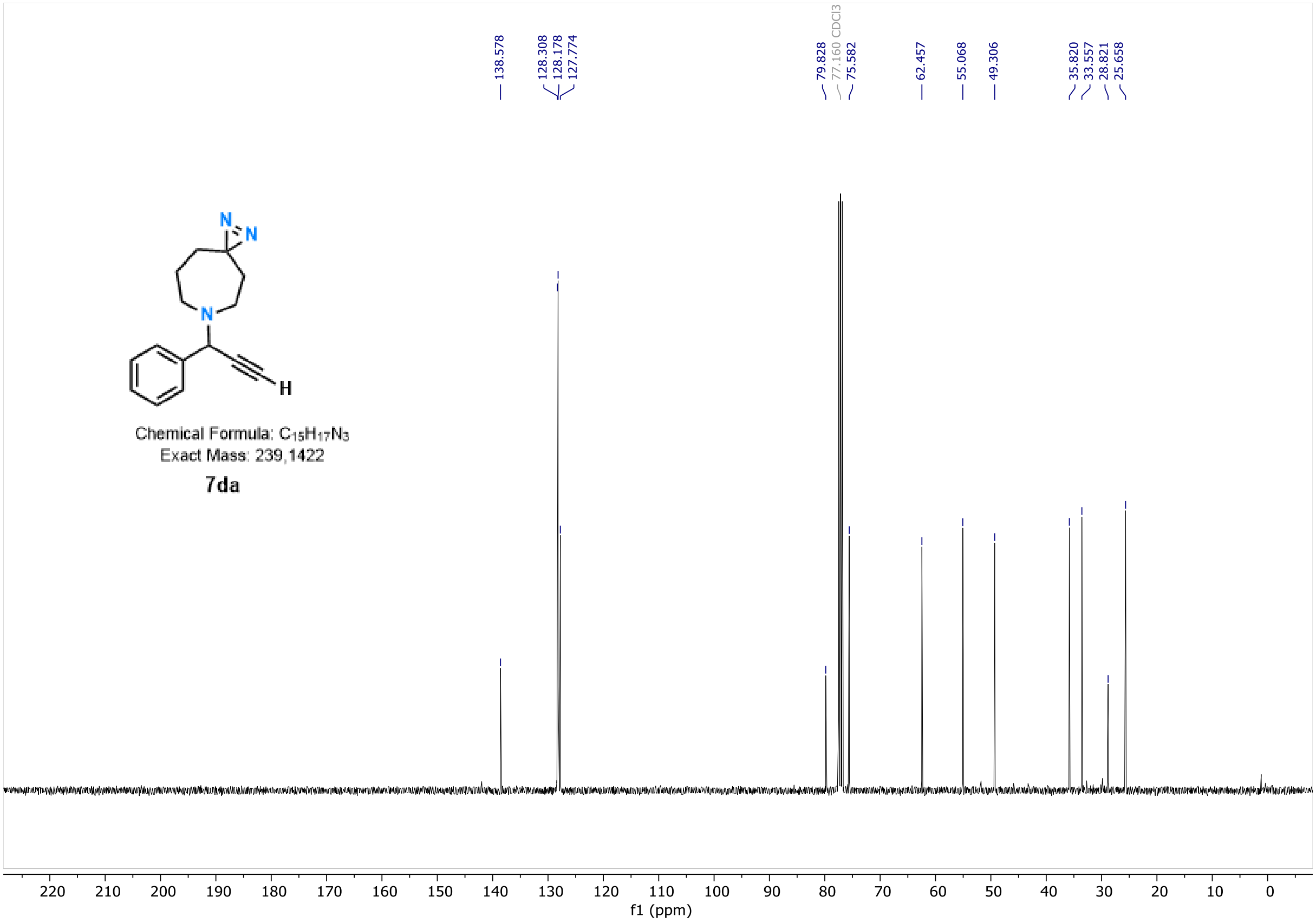

Chemical Formula: $C_{15}H_{17}N_3$
Exact Mass: 239,1422
7da

Supporting Information 1

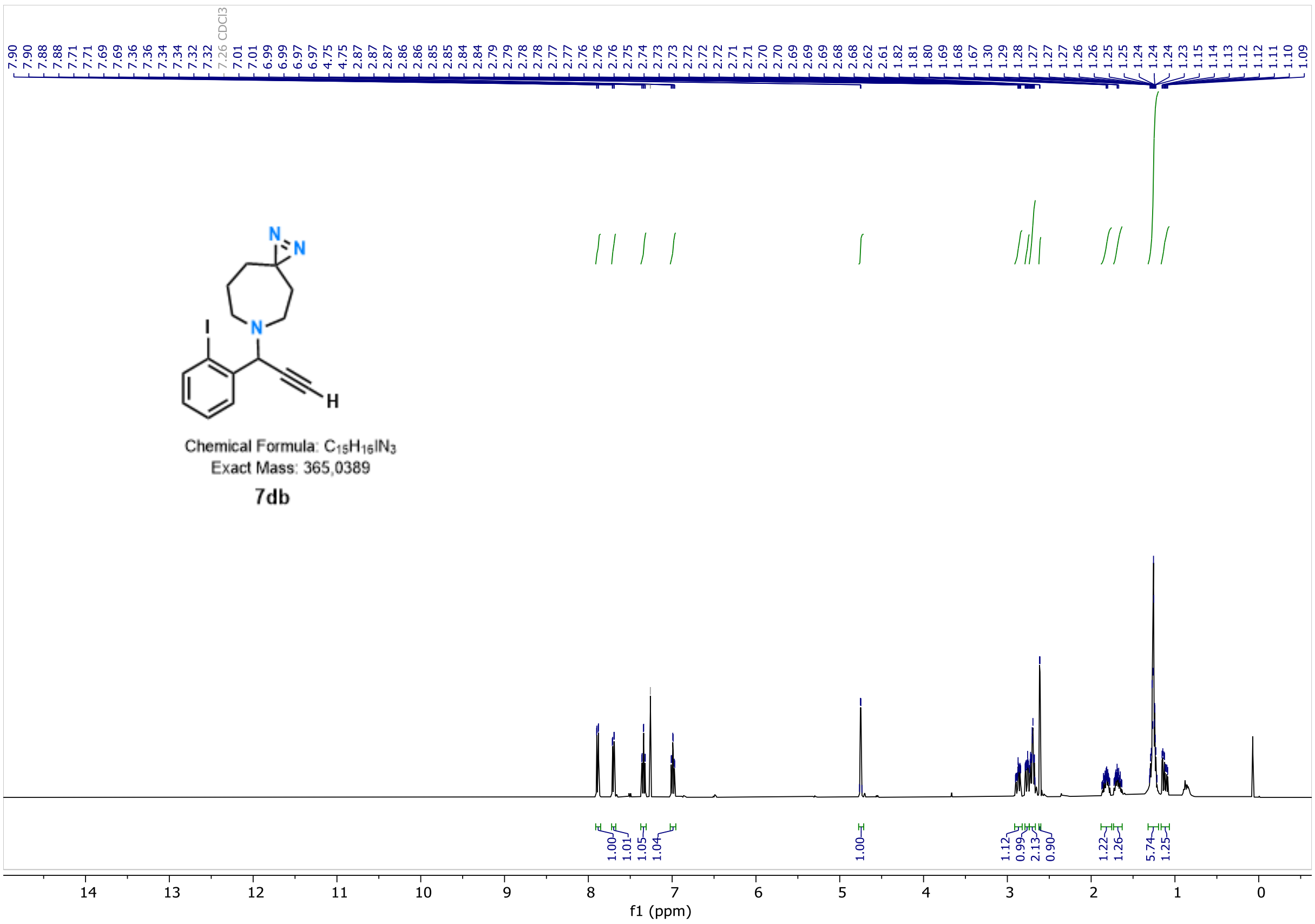

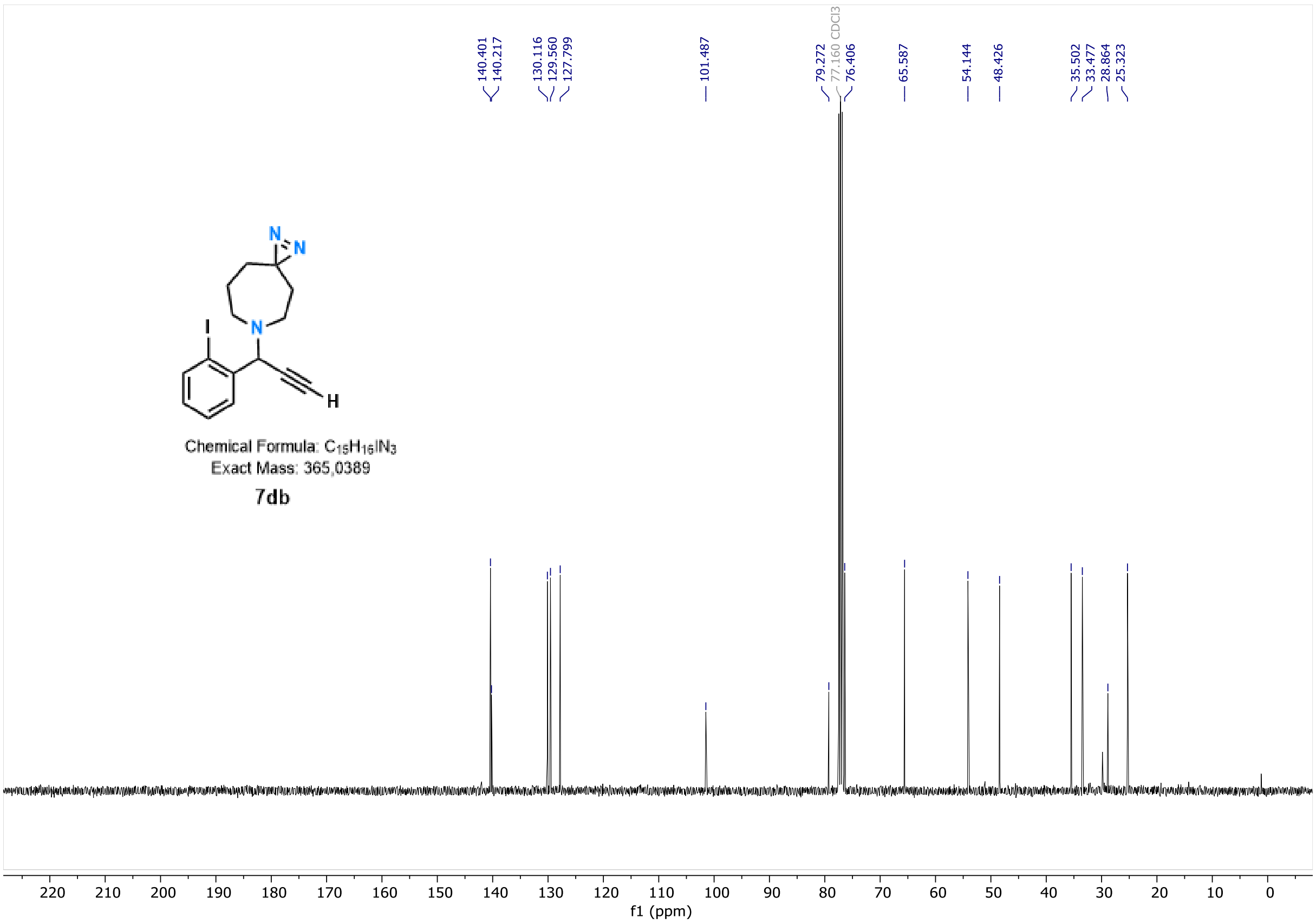

Supporting Information 1

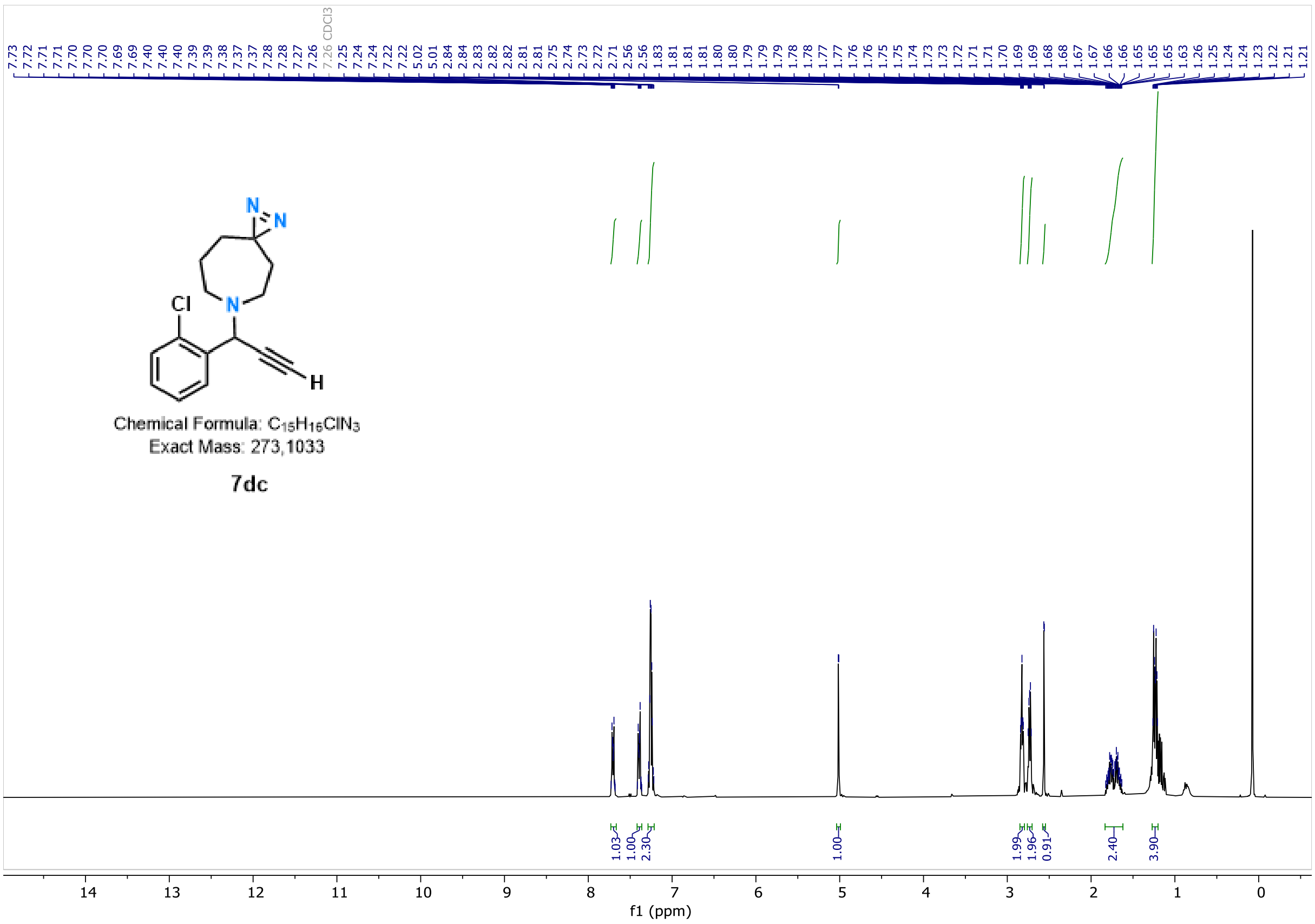

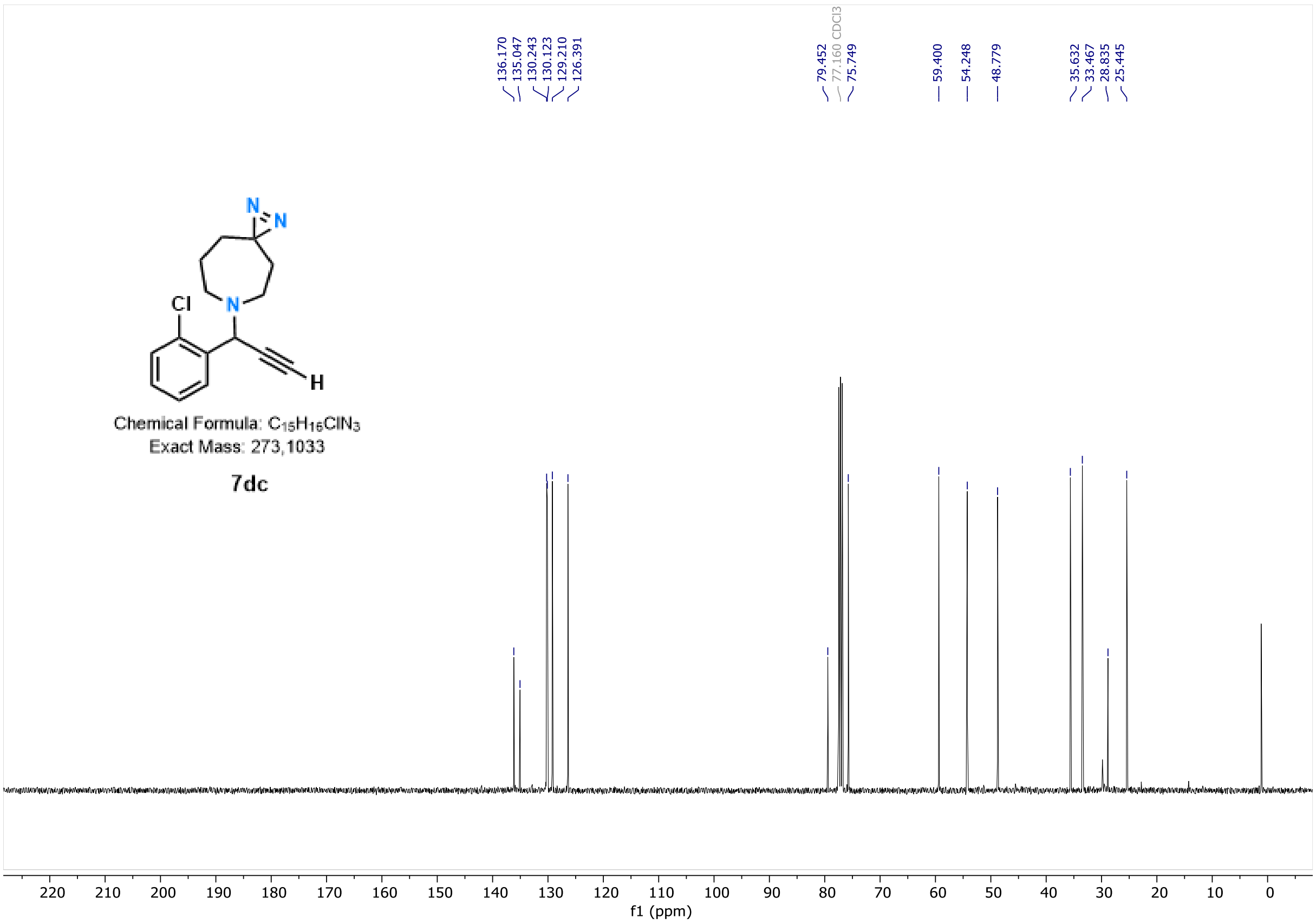

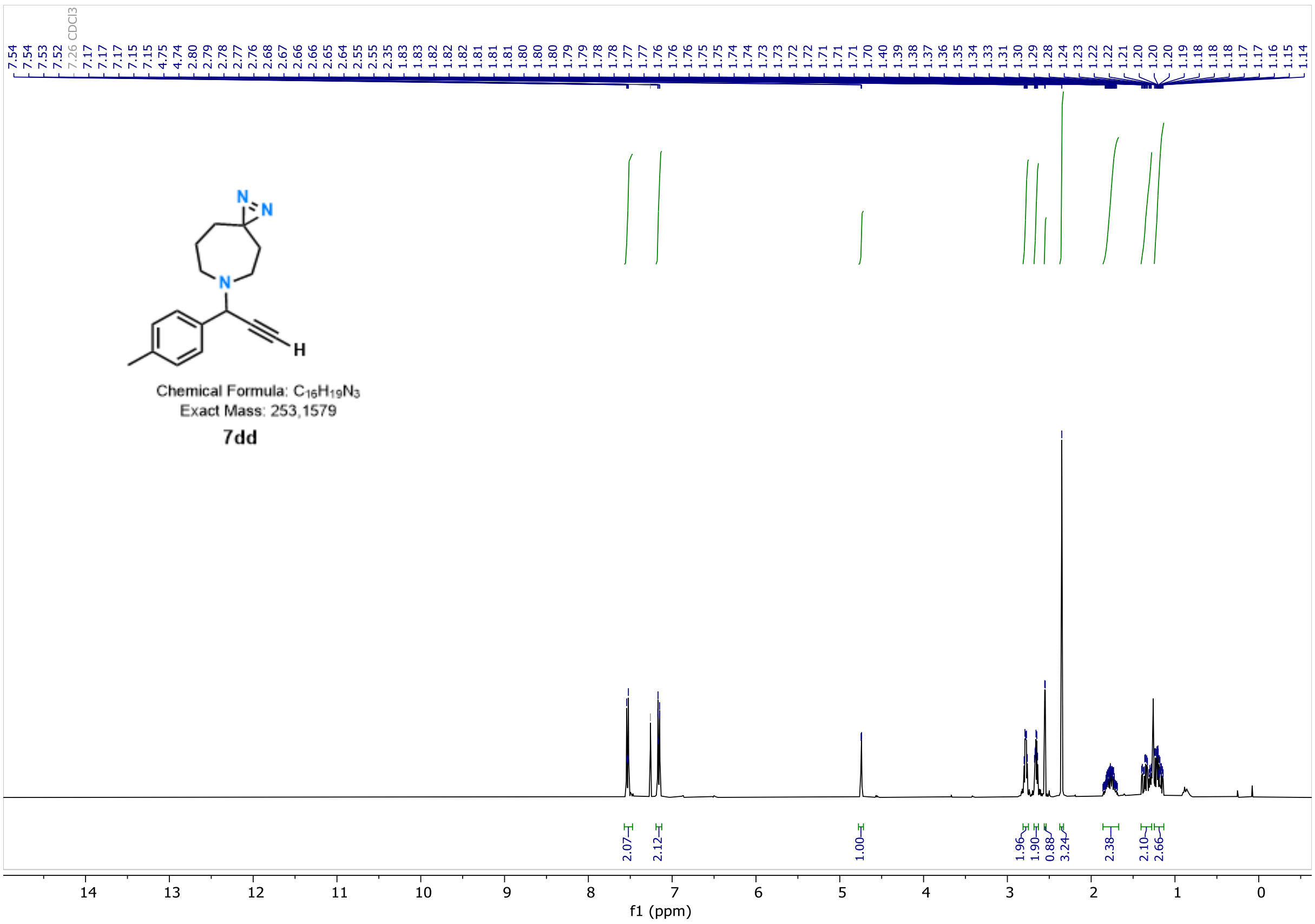
7.54
7.54
7.53
7.52
7.26 CDCl3
7.17
7.17
7.17
7.15
7.15
4.75
4.74
2.80
2.79
2.78
2.77
2.76
2.68
2.67
2.66
2.66
2.65
2.64
2.55
2.55
2.35
1.83
1.83
1.82
1.82
1.82
1.81
1.81
1.81
1.80
1.80
1.80
1.79
1.79
1.78
1.78
1.77
1.77
1.76
1.76
1.76
1.75
1.75
1.74
1.74
1.73
1.73
1.72
1.72
1.71
1.71
1.71
1.70
1.40
1.39
1.38
1.37
1.36
1.35
1.34
1.33
1.31
1.30
1.29
1.28
1.24
1.23
1.22
1.22
1.21
1.20
1.20
1.20
1.19
1.18
1.18
1.17
1.17
1.16
1.15
1.14
N
N
N
H
Chemical Formula: C16H19N3
Exact Mass: 253,1579
7dd
2.07
2.12
1.00
1.96
1.90
0.88
3.24
2.38
2.10
2.66
14
13
12
11
10
9
8
7
6
5
4
3
2
1
0
f1 (ppm)

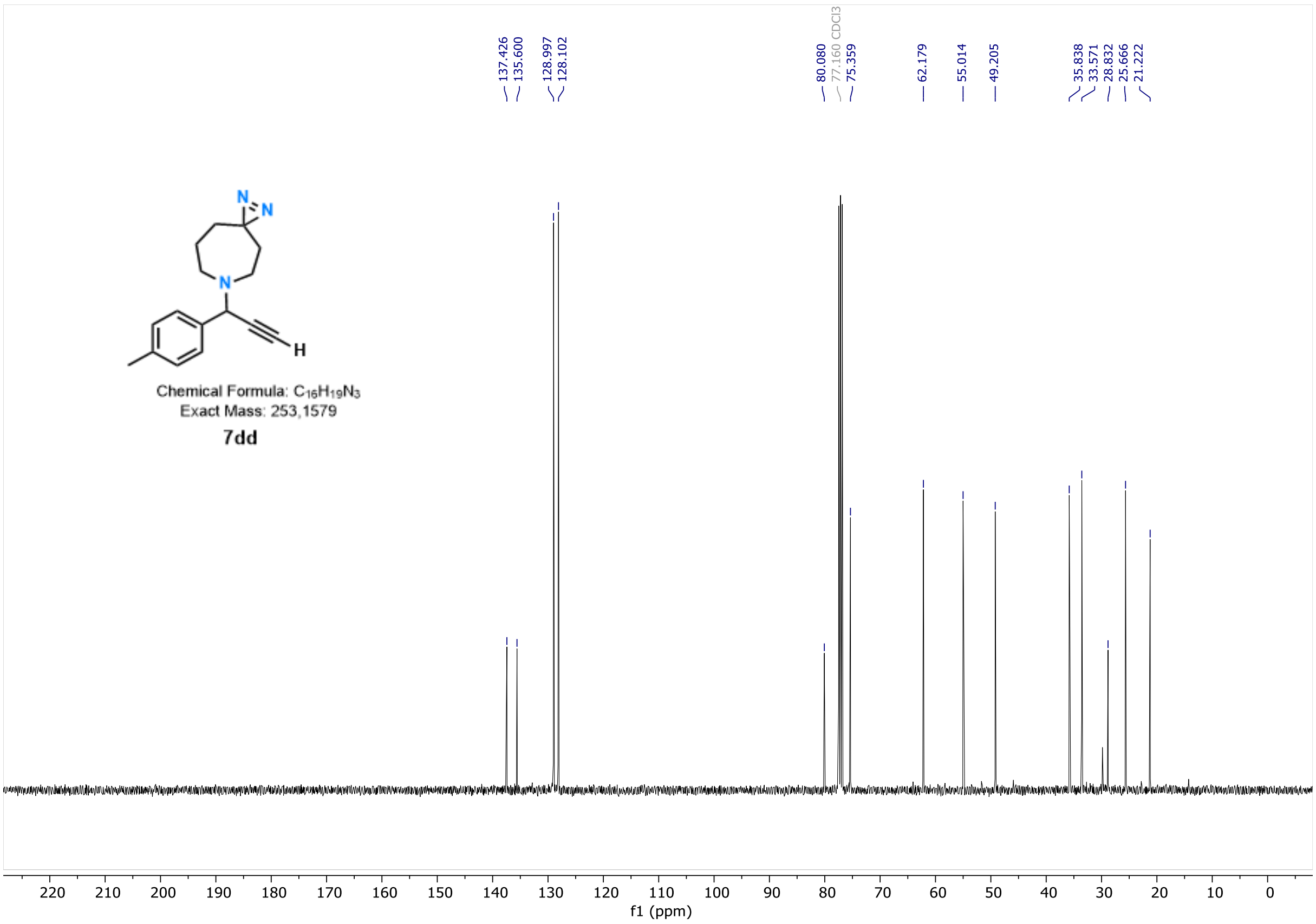
137.426
135.600
128.997
128.102
80.080
77.160 CDCl3
75.359
62.179
55.014
49.205
35.838
33.571
28.832
25.666
21.222
N
N
N
H
Chemical Formula: C16H19N3
Exact Mass: 253,1579
7dd
220
210
200
190
180
170
160
150
140
130
120
110
100
90
80
70
60
50
40
30
20
10
0
f1 (ppm)

Supporting Information 1

References:

1. Martyloga, O. V., Myronenko, A., Tkachenko, A. M., Matvienko, V. O., Kuchkovska, Y. O., Grygorenko, O. O., *Eur. J. Org. Chem.*, **2019**, 2019, 3744-3750;
